# Guidance for data analysis in split-plot designs when parametric assumptions are violated: *F*-statistic, adjusted *F*-tests, and bootstrap-*F*

**DOI:** 10.3389/fpsyg.2026.1787528

**Published:** 2026-08-06

**Authors:** María J. Blanca, Rafael Alarcón, Roser Bono, Jaume Arnau, F. Javier García-Castro, Guillermo Vallejo

**Affiliations:** 1 Department of Psychobiology and Behavioral Sciences Methodology, University of Malaga, Málaga, Spain; 2 Department of Social Psychology and Quantitative Psychology, University of Barcelona, Barcelona, Spain; 3 Institute of Neurosciences, University of Barcelona, Barcelona, Spain; 4 Department of Psychology, Universidad Loyola Andalucia, Seville, Spain; 5 Department of Psychology, University of Oviedo, Oviedo, Spain

**Keywords:** anova, bootstrap, Greenhouse-Geisser adjustment, Huynh-Feldt adjustment, repeated measures

## Abstract

**Introduction:**

Data analysis of split-plot designs requires normality, homogeneity of variance, and multisample sphericity. Adjusted *F*-tests and the bootstrap-*F* procedure have been proposed as alternatives to the *F*-statistic when these assumptions are violated. However, simulation studies have not identified the conditions under which these tests are robust.

**Method:**

To analyze the Type I error of the *F*-statistic, Greenhouse-Geisser (*F-GG*) and Huynh-Feldt (*F-HF*) adjustments, and bootstrap-*F* (*B-F*), we performed a simulation study with split-plot designs, manipulating the distribution shape, epsilon values, sample size, coefficient of sample size variation, degree of heterogeneity, and pairing of variance with group sample size.

**Results:**

With balanced designs, the results show that for the group effect the *F*-statistic and *B-F* are valid choices under assumption violations. For time and interaction effects, under non-sphericity, *F-GG* and *F-HF* maintain Type I error close to 5% with moderate violation of normality; both of them remain robust with $\hat{\varepsilon }\geq$ 0.70 for larger violations of normality. With unbalanced designs, the behavior of all these statistics depends on the variables manipulated.

**Discussion:**

The paper identifies the conditions under which each procedure can be used. Overall, *B-F* was the most robust procedure in the majority of conditions. It can be used under moderate violations of normality, homogeneity, and sphericity with *N* > 20. Under more severe violations of these assumptions, larger sample size, *N* > 80, is needed to achieve robustness.

## Introduction

The split-plot design is a mixed design that incorporates both a between-subjects or grouping factor and a within-subject factor, in which the dependent variable is registered in all participants under all conditions of the within-subject factor, but only under one level of the grouping factor. When the design involves repeated measures over several periods of time, it is conceived as longitudinal. Under the general linear model (GLM), the conventional statistical procedure is repeated measures analysis of variance (ANOVA), which uses the *F*-statistic to test the statistical significance associated with the null hypothesis corresponding to the different effects. This design yields two main effects, namely group and within-subject factor effects (hereinafter, time effect), as well as the interaction effect between them. For a valid statistical decision, this test requires fulfillment of the assumptions of normality, homogeneity of variance, and multisample sphericity (Huynh, 1978; Kirk, 2013). The latter assumption implies both homogeneity of the variance-covariance matrices within each level of the between-subjects factor, and sphericity in the common variance-covariance matrix. That is, there must be sphericity for the within-subject factor for each level of the between-subjects factor.

Monte Carlo simulation studies are commonly used to examine the impact of assumption violation on the performance of statistical procedures, understood in terms of Type I error and power. Type I error is generally interpreted using Bradley (1978) liberal criterion (e.g., Berkovits et al., 2000; Blanca et al., 2017, 2023a; Keselman et al., 1999; Oberfeld and Franke, 2013; Vallejo et al., 2006), according to which a procedure is considered robust if the Type I error rate is between 2.5% and 7.5% for a nominal alpha of 5%. The procedure is considered liberal when the Type I error rate is above 7.5%, and conservative when it is below 2.5%. The results of all the studies cited below have been interpreted according to this criterion.

Most simulation studies performed with split-plot designs have focused on the performance of procedures for time and group × time interaction effects. The group effect has typically been analyzed in studies involving one-way designs, and the empirical evidence suggests that the *F*-statistic for this effect is not affected by non-normality (Black et al., 2010; Blanca et al., 2017; Lantz, 2013; Patrick, 2007; Schmider et al., 2010). Regarding the homogeneity assumption, one of the most important variables is the inequality of group sizes. When the homogeneity of variances assumption is satisfied, the *F*-statistic is generally robust with both balanced and unbalanced designs (Lee and Ahn, 2003; Yigit and Gökpinar, 2010). In the case of heterogeneity, it is also robust with a balanced design (Blanca et al., 2018a; Patrick, 2007; Yigit and Gökpinar, 2010). By contrast, with unbalanced designs the robustness of the *F*-statistic depends on the degree of both variance heterogeneity and inequality in group sizes, as well as on the pairing of variance with group size (Lee and Ahn, 2003; Moder, 2010; Blanca et al., 2018a). The ratio of the largest to the smallest variance (variance ratio) is used as a measure of heterogeneity, while the coefficient of sample size variation (Δ*n*) indicates the amount of inequality in group sizes. Pairing is positive when the largest group size is associated with the largest value of variance, whereas it is negative when the largest group size is associated with the smallest variance. In this regard, Blanca et al. (2018b) found that the *F*-statistic tended to show liberal behavior with negative pairing and a variance ratio greater than 1.5, with this behavior becoming more pronounced with increases in the variance ratio (5 and 9) and inequality of group sizes (Δ*n* = 0.33 and 0.50). By contrast, the *F*-statistic tended to be conservative with positive pairing and a variance ratio greater than 2. As in the previous case, this behavior worsened with higher variance ratio and greater inequality of group sizes.

Regarding the time effect and the group × time interaction, Keselman et al. (1996) showed that the *F*-statistic was robust to violations of normality, although it has a tendency to be liberal with highly skewed distributions. However, most studies have found that violation of sphericity has a serious effect on its robustness (Berkovits et al., 2000; Blanca et al., 2023b, 2024; Fernández et al., 2010; Haverkamp and Beauducel, 2017, 2019; Keselman et al., 1996; Rogan et al., 1979; Vallejo et al., 2010). For both effects, the violation of sphericity causes the *F*-statistic to show liberal behavior with equal group sizes (Fernández et al., 2007; Keselman et al., 1996; Keselman and Rogan, 1980; Rogan et al., 1979), while with unequal group sizes its behavior again depends on the type of pairing; with negative pairing the *F*-statistic is liberal, whereas with positive pairing it is conservative (Fernández et al., 2007; Rogan et al., 1979; Vallejo et al., 2001, 2010).

A number of alternatives to the *F*-statistic for sphericity violations have been proposed, namely adjusted *F*-tests, multivariate analysis, non-parametric procedures, linear mixed models, robust statistics, and bootstrap methods (Arnau et al., 2012, 2013; Blanca et al., 2023b, 2024, 2025; Livacic-Rojas et al., 2010; Sheskin, 2003; Wilcox, 2022). Of these, adjusted *F*-tests are the most widely used as they can be implemented in most statistical packages. These procedures make the test more restrictive (Box, 1954) by reducing the degrees of freedom of each effect by a multiplicative factor called epsilon (ε). The value of ε represents the degree of deviation from sphericity, and it ranges from its lower limit (1/*K*−1) to 1 (Geisser and Greenhouse, 1958), with *K* being the number of repeated measures. When sphericity is satisfied, the value of ε is equal to or close to 1, whereas when it is violated, ε is further from 1 and close to its lower limit. The two most widely known and used adjusted *F*-tests are the Greenhouse-Geisser (*F-GG*; Box, 1954; Geisser and Greenhouse, 1958; Greenhouse and Geisser, 1959) and Huynh-Feldt (*F-HF;* Huynh and Feldt, 1976) adjustments, in which the estimators of ε are referred to as $\hat{\varepsilon }$ and $\tilde{\varepsilon }$, respectively.

There is extensive empirical evidence from simulation studies on the effect of assumption violations on the behavior of these adjusted *F*-tests for time and interaction effects. Regarding the time effect, and in balanced designs, both *F-GG* and *F-HF* have been found to maintain adequate control of Type I error in the presence of sphericity violation, different variance ratios between groups (e.g., 1:1:1, 1:1.5:2, and 1:3:5), and different variance-covariance structures for both normal and non-normal data (Algina, 1994; Algina and Oshima, 1994; Keselman and Keselman, 1990; Keselman et al., 1995, 1999; Rogan et al., 1979). Both procedures have been recommended when the groups have equal sample sizes (Keselman et al., 1995; Rogan et al., 1979).

In designs with unequal group sizes, the robustness of adjusted *F*-tests for the time effect depends on the degree of heterogeneity of variances, pairing of variances with group size, and the coefficient of variation of group size. If pairing is positive, adjusted *F*-tests are conservative, whereas if it is negative they are liberal; these behaviors become more pronounced with higher coefficients of variation of group size and smaller sample size (Algina, 1994; Algina and Oshima, 1994, 1995; Keselman et al., 1995, 1999). Overall, the adjusted *F*-tests are not recommended when there is heterogeneity of variance.

For interaction effects the results obtained are in line with the time effects. In balanced designs, adjusted *F*-tests show adequate control of Type I error with normal and non-normal data under violation of homogeneity of variances and sphericity violation (Keselman and Keselman, 1990; Keselman et al., 1995, 1999; Rogan et al., 1979). In unbalanced designs, their behavior tends to be liberal if pairing is negative (Algina, 1994; Algina and Oshima, 1994; Keselman and Keselman, 1990; Keselman et al., 1995, 1999), and conservative if pairing is positive, except with a low coefficient of variation of group size and $\hat{\varepsilon }$ ≤ 0.75, in which case they are robust (Algina, 1994; Keselman and Keselman, 1990; Keselman et al., 1995). These results indicate that neither of the adjusted *F*-tests may be suitable alternative procedures under heterogeneity and unequal group size, leading some authors (Keselman and Keselman, 1990) to recommend balanced designs.

Another proposed alternative to ANOVA and adjusted *F*-tests when parametric assumptions are violated is the bootstrap method, which consists in performing a random resampling of centered data with replacement, generating a set of *B* bootstrap samples. The *F*-statistic is computed in each sample, thereby creating its empirical distribution (Wilcox, 2003). The bootstrap approach was developed by Efron (1979) as a compromise between parametric and non-parametric methods. This method does not require fulfillment of parametric assumptions and allows researchers to obtain more accurate estimators in situations of assumption violations (Berkovits et al., 2000; Keselman et al., 2000; Vallejo et al., 2002). Berkovits et al. (2000) developed bootstrap-*F* (*B-F*) and assessed its robustness through a Monte Carlo simulation study in a one-way design with four repeated measures. They manipulated different sample sizes (from 10 to 60), ε values (from 0.48 to 1), and different values of skewness (γ_1_) and kurtosis (γ_2_) so as to generate non-normal distributions corresponding to slight (γ_1_ = 1, γ_2_ = 0.75), moderate (γ_1_ = 1.75, γ_2_ = 3.00), and severe deviations from normality (γ_1_ = 3.00, γ_2_ = 21.00). The results showed that under simultaneous violations of normality and sphericity, *B-F* is robust, even with small sample sizes, although it may become conservative in some scenarios with ε = 1. Similar results have recently been reported by Blanca et al. (2025) for one-way designs under a wider variety of scenarios of non-normality and non-sphericity, although it was also suggested that the sample size should be larger than 20–25 with severe violations of parametric assumptions.

Vallejo et al. (2006) described the bootstrap procedure for split-plot designs. These authors studied the behavior of *B-F* in both balanced and unbalanced 3 × 4 split-plot designs, manipulating the total sample size (*N* = 30, 45, and 60), coefficients of sample size variation (Δ*n* = 1 and 0.33), degree of heterogeneity of the variance-covariance matrix (1:1:1, 1:3:5), type of pairing (null, positive, and negative), ε values (ε = 0.50, 0.70, and 1), and distribution shape (same distributions as Berkovits et al., 2000). Regarding the group effect, the results showed that *B-F* was generally robust, except in two conditions of extreme violation in which it showed conservative behavior: (a) *N* = 30, positive pairing, severe deviation from normality (γ_1_ = 3.00, γ_2_ = 21.00), and severe deviation from sphericity (ε = 0.50); and (b) *N* = 30, positive pairing, severe non-normality, while the covariance matrices differed across groups and were not proportional, with different levels of sphericity across groups (ε = 1, 0.75, and 0.50). For the time effect, the results showed that *B-F* adequately controlled Type 1 error with normal data. However, *B-F* was conservative with severe deviation from normality and the sphericity assumption, regardless of the type of pairing. Regarding the interaction effect, *B-F* was again robust with normal distributions. With non-normal distributions, *B-F* was conservative with severe deviation from normality and ε = 0.70 and 1. In a subsequent study, Vallejo et al. (2010) reported adequate control of Type I error for *B-F* with a 3 × 4 split-plot design (balanced and unbalanced), *N* = 30 and 45, an ε value of 0.50, equal and unequal variance-covariance matrices (1:3:5 and 1:5:9), and normal and non-normal distributions (double exponential, exponential, and lognormal). Overall, the empirical evidence indicates that *B-F* is robust to a wide variety of situations in which the assumptions of split-plot designs are violated, and hence it can be considered a suitable alternative for analysis in these situations.

In general, studies show that the *F*-statistic is sensitive to homogeneity and sphericity assumptions and that *F*-adjusted tests may be a good alternative for time and interaction effects in balanced designs. However, in unbalanced designs, adjusted *F*-tests do not correct for the effect of non-sphericity and they can be conservative or liberal, depending on the conditions of heterogeneity of variances and group sample sizes. The conclusion, therefore, is that they are not suitable alternatives in unbalanced designs. However, simulation studies have not identified the conditions under which these tests could be used with guarantees of robustness, nor do they clarify how the non-normality of data influences Type I error under the different scenarios of non-sphericity and heterogeneity. This contrasts with actual practice in research, insofar as adjusted *F*-tests are systematically used in split-plot designs without considering the results of simulation studies. As for *B-F*, this procedure seems to show adequate performance and it can be considered a promising alternative. However, Monte Carlo studies are very limited, and its behavior needs to be analyzed in a larger number of scenarios.

The aim of the present study is to provide new evidence on the behavior of the *F*-statistic, adjusted *F*-tests, and *B-F* with split-plot designs in the presence of assumption violations, clarifying the conditions in which each of these procedures may be suitable. Simulation studies with split-plot designs have frequently used 3 × 4 designs (e.g., Algina, 1994; Algina and Oshima, 1994; Keselman and Keselman, 1990; Rogan et al., 1979; Vallejo et al., 2006, 2010). However, research practice reveals that the most frequently used number of levels is 2 for the grouping factor and 2 or 3 for the within-subject factor (Bono et al., 2021; Goedert et al., 2013). Therefore, we conduct an exhaustive Monte Carlo simulation study with a 2x3 split-plot design and 5,250 conditions, manipulating the following variables: Distribution shape (normal, as well as slight, moderate, severe, and extreme deviation from normality), epsilon values (from $\hat{\varepsilon }$ = 0.50 to 1), total sample size (*N* from 20 to 200), coefficient of group size variation (Δ*n* = 0, 0.16, 0.33, and 0.50), ratio of the variance-covariance matrices between the two groups (1:1, 1:1.5, 1:2, and 1:5), and type of pairing of variance with group size (null, positive, and negative). Our goal is to identify the conditions under which the *F*-statistic, *F-GG, F-HF*, and *B-F* can be used in 2 × 3 designs, providing guidelines for applied researchers regarding the use of these procedures.

## Materials and methods

A Monte Carlo simulation study was conducted using the PROC GLM module of SAS 9.4, together with the interactive matrix language (IML) module. A series of *ad hoc* macros were created for data generation. Normal data were generated using the Cholesky transformation of the variance-covariance matrix, while non-normal data were generated using Fleishman (1978) procedure. To simulate data with different degrees of sphericity violation, a series of unstructured variance-covariance matrices were generated with different values of $\hat{\varepsilon }$.

The variables manipulated were as follows:

1. Shape of the distribution: The normal distribution and four non-normal distributions were used, with different values of skewness and kurtosis coefficients. Non-normal distributions were grouped into slight (γ_1_ = 0.4, γ_2_ = 0.8), moderate (γ_1_ = 1, γ_2_ = 1.5), severe (γ_1_ = 1.41, γ_2_ = 3), and extreme (γ_1_ = 2.31, γ_2_ = 8) deviation from normality. Blanca et al. (2013) found that 80% of real data showed skewness and kurtosis values ranging between −1.25 and 1.25. Distributions with slight and moderate deviations from normality were selected based on this finding. Distributions with severe and extreme deviations were included to represent larger departures, which have typically been used in simulation studies involving repeated measures (Blanca et al., 2023a, 2024, 2025).
2. Epsilon values ($\hat{\varepsilon }$): The values of $\hat{\varepsilon }$ were 0.50, 0.60, 0.70, 0.80, 0.90, and 1.
3. Total sample size: Seven sample sizes were considered, including small, medium, and large samples: 20, 40, 60, 80, 100, 150, and 200.
4. Coefficient of sample size variation (Δ*n*). This represents the amount of inequality in group sizes and was calculated by dividing the standard deviation of the group size by its mean. The values of Δ*n* were 0 (balanced design), 0.16 (low value), 0.33 (medium value), and 0.50 (high value).
5. Ratio of the variance-covariance matrices between the two groups (variance ratio, VR): (a) homogeneity of variance, 1:1; (b) heterogeneity of variance, 1:1.5, 1:2, and 1:5. In this scenario, the variance-covariance matrix of the second group could be, respectively, 1.5, 2, or 5 times larger than that of the first group.
6. Pairing of variance with group sample size. The pairings used were: null when there are equal group sizes; positive when the largest group size is associated with the largest value of variance; and negative when the largest group size is associated with the smallest variance.

Five thousand replications were performed for each of the conditions. We recorded the Type I error rate of the *F-*statistic, *F-GG, F-HF*, and *B-F*, which represents the proportion of rejection of the null hypothesis when it is true. In line with other researchers and to facilitate comparison between studies, Bradley (1978) liberal criterion was used to evaluate robustness, and hence a procedure was considered robust if the Type I error rate was between 2.5% and 7.5% for a nominal alpha of 5%. A procedure was considered liberal when the Type I error rate was above 7.5%, and conservative when it was below 2.5%.

## Results

### Balanced designs

#### Group effect

**Figure 1:**
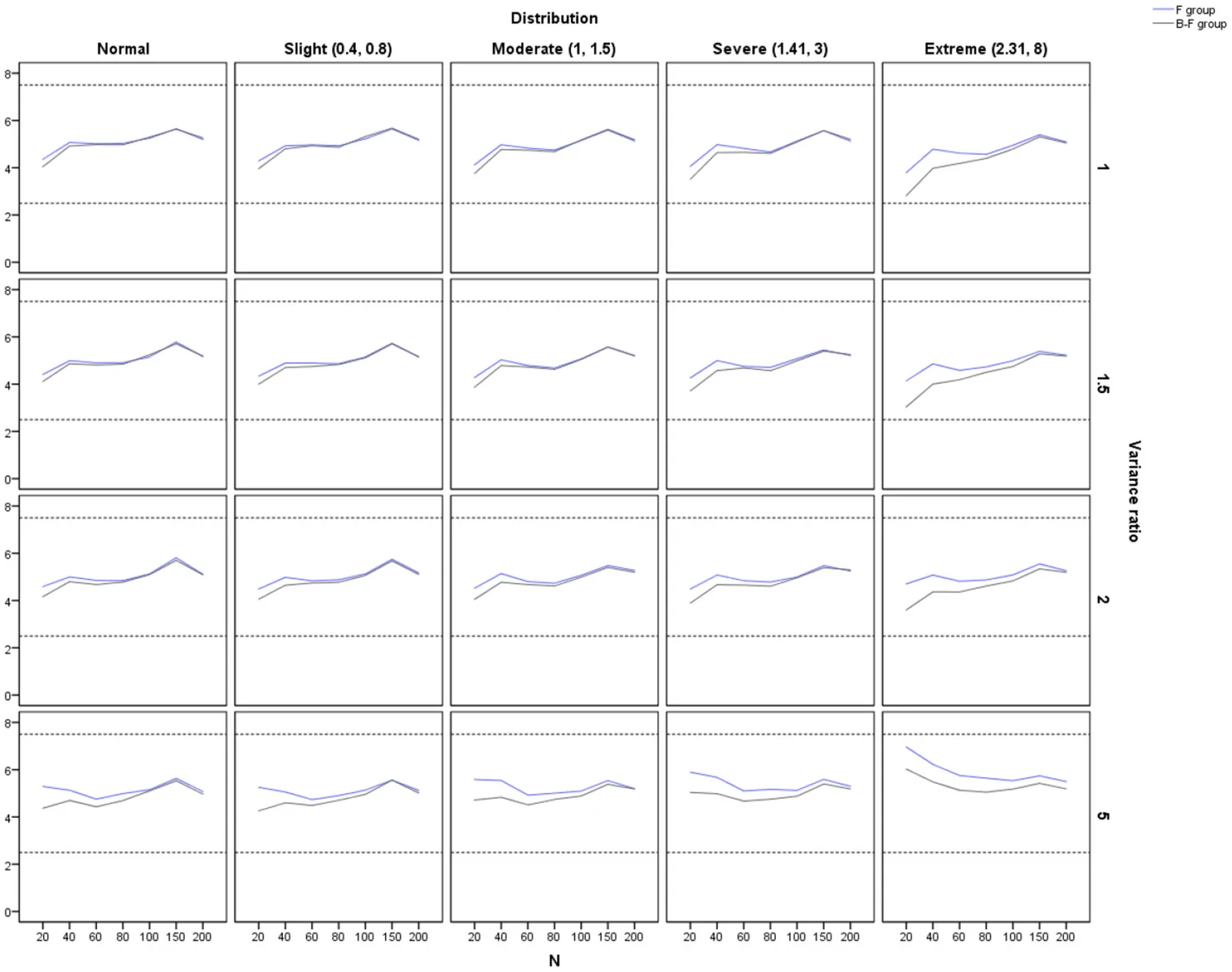
Type I error rates (in percentages) for the group effect in balanced designs for the *F*-statistic and *B-F*, by distribution (in parenthesis: skewness, γ_1_, and kurtosis, γ_2_, coefficients), variance ratio, and total sample size.

Figure 1 shows the results for balanced designs and the group effect (840 conditions). Type I error rates for the *F*-statistic and *B-F* are always within the bounds [2.5%, 7.5%], indicated by dashed lines. Specifically, Type I errors for these procedures are between 3.58% and 7.32% (*M* = 5.07, *SD* = 0.47) and between 2.60% and 6.36% (*M* = 4.83, *SD* = 0.56), respectively. Therefore, both the *F*-statistic and *B-F* control the Type I error rate under violations of normality and heterogeneity of variances in the conditions manipulated here.

#### Time effect

**Figure 2:**
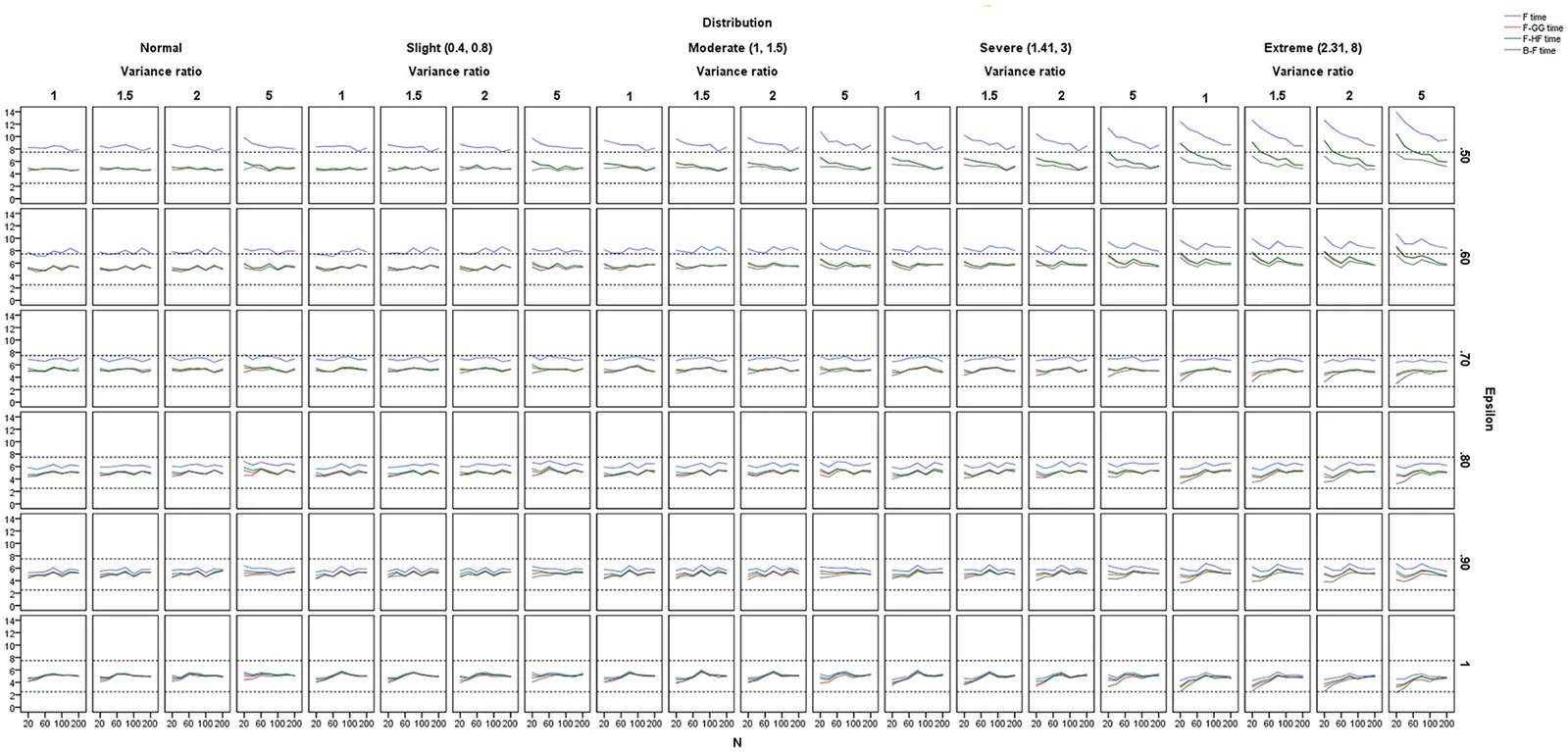
Type I error rates (in percentages) for the time effect in balanced designs for the *F*-statistic, *F-GG, F-HF*, and *B-F* by distribution (in parenthesis: skewness, γ_1_, and kurtosis, γ_2_, coefficients), epsilon values, variance ratio, and total sample size.

Results for the time effect are shown in Figure 2. With $\hat{\varepsilon }$ = 0.50 and 0.60, the *F*-statistic is liberal, with Type I error rates as high as 13.92%. With $\hat{\varepsilon }$ = 0.70, the *F*-statistic is close to 7.5%, and with $\hat{\varepsilon }$ ≥ 0.80 it controls Type I error. With $\hat{\varepsilon }$ ≤ 0.60, *F-GG* and *F-HF* tend to be liberal with severe and extreme deviation from normality and small sample size. With $\hat{\varepsilon }\geq$ 0.70, both statistics are robust and close to 5%. *F-GG* and *F-HF* get closer to 5% than does the *F*-statistic as $\hat{\varepsilon }$ departs from 1. *B-F* is robust in practically all cases, except with $\hat{\varepsilon }$ = 1, a variance ratio of 5, and *N* = 20, in which case it becomes conservative. Type I error rates for *B-F* range from 2.38% to 7.26% (*M* = 5.01, *SD* = 0.56).

#### Group × time interaction

**Figure 3:**
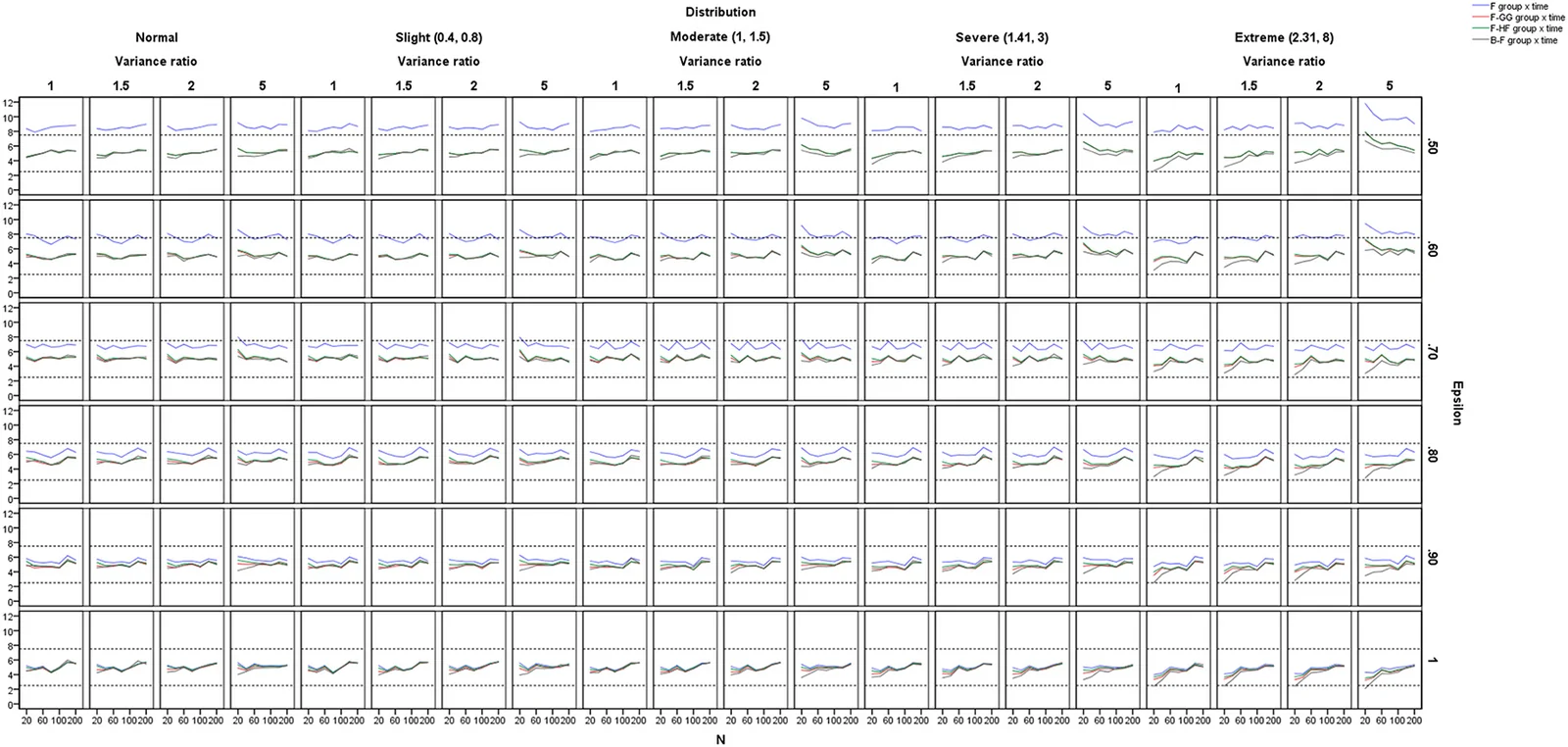
Type I error rates (in percentages) for the interaction effect in balanced designs for the *F*-statistic, *F-GG, F-HF*, and *B-F* by distribution (in parenthesis: skewness, γ_1_, and kurtosis, γ_2_, coefficients), epsilon values, variance ratio and total sample size.

Figure 3 shows the results for the group × time interaction. The interaction effect of the *F*-statistic is affected mainly by the violation of sphericity, with a maximum Type I error of 11.80%. It exhibits a tendency toward liberality with $\hat{\varepsilon }$ ≤ 0.70. However, *F-GG* and *F-HF* are robust in all cases, except with $\hat{\varepsilon }$ = 0.50, extreme deviation from normality, a variance ratio of 5, and *N* = 20. Type I error rates for these two procedures range from 3.24% to 7.86% (*M* = 4.96, *SD* = 0.45), and from 3.52% to 7.90% (*M* = 5.05, *SD* = 0.43), respectively. *B-F* is also robust, except with $\hat{\varepsilon }$ = 1, extreme violation of normality, and *N* = 20, in which case it becomes conservative. Type I error rates for *B-F* range from 2.10% to 6.70% (*M* = 4.81, *SD* = 0.58).

### Unbalanced designs with homogeneity of variance

#### Group effect

**Figure 4:**
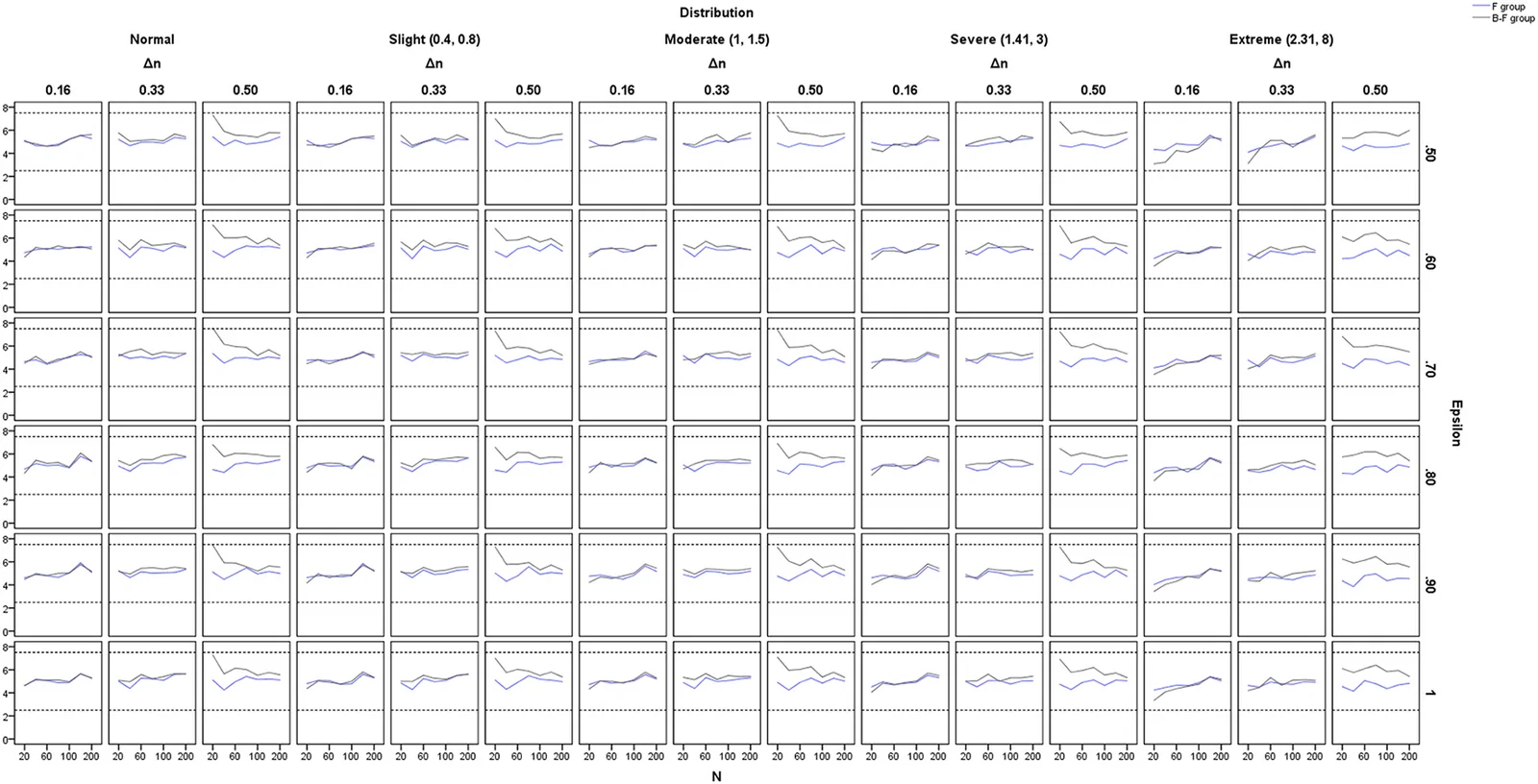
Type I error rates (in percentages) for the group effect in unbalanced designs with a variance ratio equal to 1 (homogeneity of variance) for the *F*-statistic and *B-F* as a function of distribution shape (in parenthesis: skewness, γ_1_, and kurtosis, γ_2_, coefficients), coefficient of sample size variation (Δ*n*), epsilon values, and sample size.

Results for the conditions in which homogeneity of variance is fulfilled (VR = 1; null pairing between variance and sample sizes) are shown in Figure 4 (630 conditions). In this scenario, the *F*-statistic for the group effect is robust in all conditions of non-normality and non-sphericity, with Type I error from 3.86% to 5.92% (*M* = 4.92, *SD* = 0.33). *B-F* is also generally robust, except for some conditions with *N* = 20 when the group sizes are very unequal (Δ*n* = 0.50), in which case it shows a tendency to be liberal and is outperformed by the *F*-statistic. Type I error rates for *B-F* range from 3.10% to 7.54% (*M* = 5.35, *SD* = 0.63).

#### Time effect

**Figure 5:**
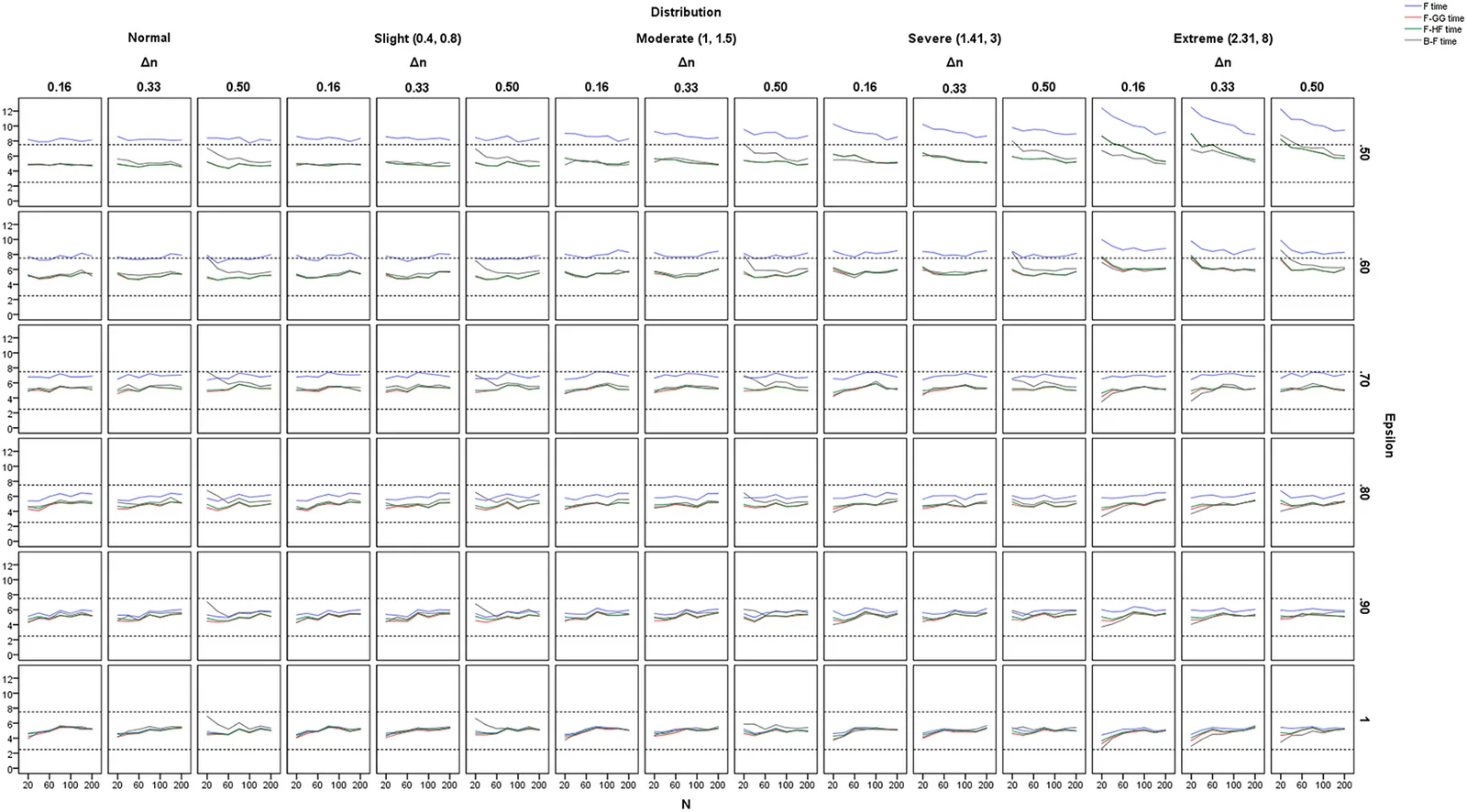
Type I error rates (in percentages) for the time effect in unbalanced designs with a variance ratio = 1 (homogeneity of variance) for the *F*-statistic, *F-GG, F-HF*, and *B-F* as a function of distribution shape (in parenthesis: skewness, γ_1_, and kurtosis, γ_2_, coefficients), coefficient of sample size variation (Δ*n*), epsilon value, and sample size.

Figure 5 shows results for the time effect. The *F*-statistic is liberal for $\hat{\varepsilon }$ ≤ 0.60 and robust with $\hat{\varepsilon }$ ≥ 0.70. *F-GG* and *F-HF* are generally robust, except with extreme deviation from normality and sphericity: $\hat{\varepsilon }$ = 0.50 and *N* ≤ 60, and $\hat{\varepsilon }$ = 0.60 and *N* =20, in which case they are liberal. *B-F* is also generally robust, except with $\hat{\varepsilon }$ ≤ 0.60, very unequal group sizes (Δ*n* = 0.50), and small sample size (*N* ≤ 40), in which case it tends to be liberal. The greater the deviation from normality, the larger the sample size required to achieve robustness (e.g., *N* > 20 for moderate and severe and *N* > 40 for extreme deviation from normality).

#### Group × time interaction

**Figure 6:**
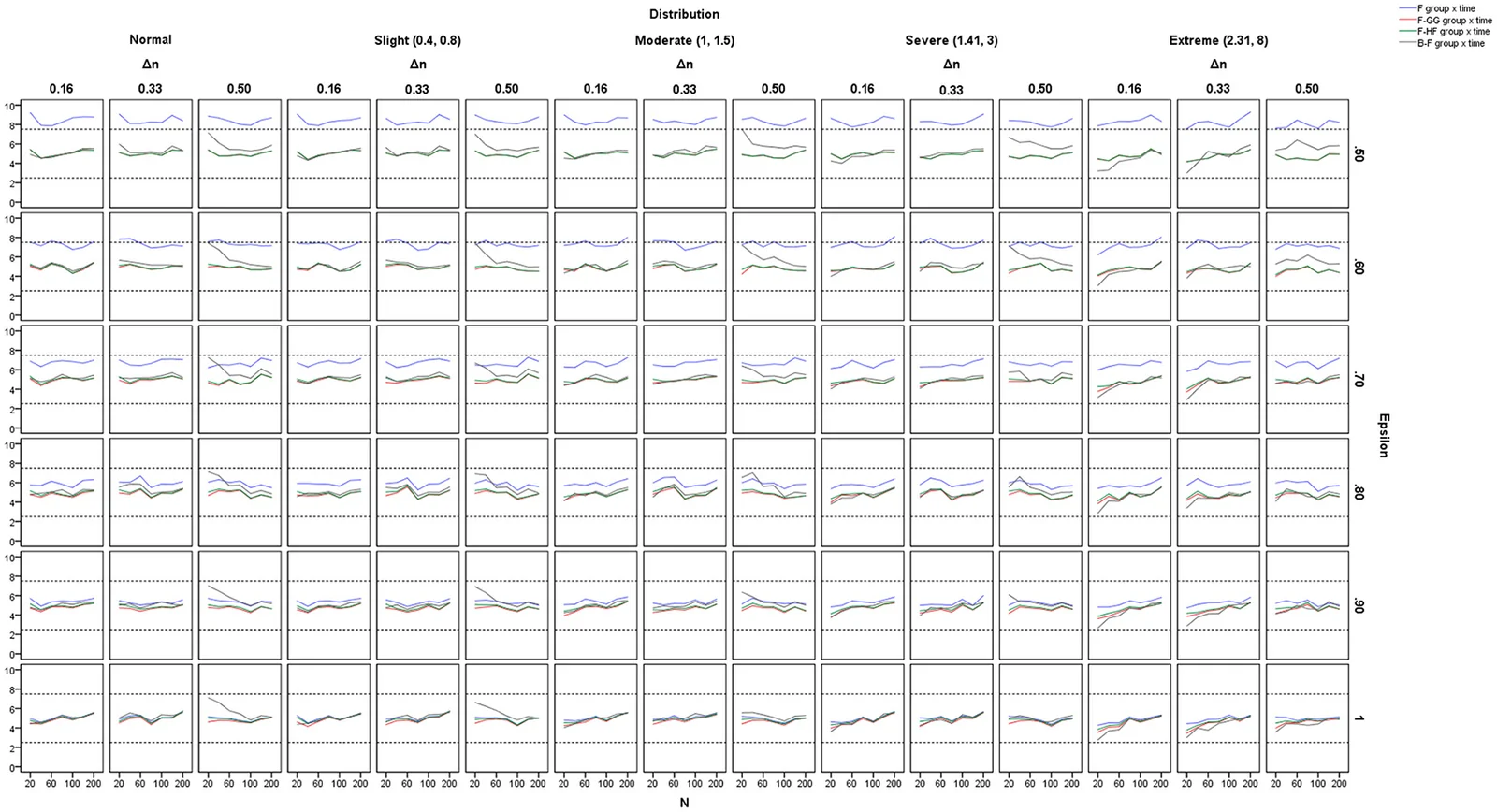
Type I error rates (in percentages) for the group × time interaction in unbalanced designs with a variance ratio = 1 (homogeneity of variance) for the *F*-statistic, *F-GG, F-HF*, and *B-F* as a function of distribution shape (in parenthesis: skewness, γ_1_, and kurtosis, γ_2_, coefficients), coefficient of sample size variation (Δ*n*), epsilon value, and sample size.

Figure 6 displays the results for the group × time interaction, which are in line with those for the time effect. The *F*-statistic for the interaction effect is liberal for $\hat{\varepsilon }$ ≤ 0.60 and robust with $\hat{\varepsilon }$ ≥ 0.70, although it is not until $\hat{\varepsilon }$ = 0.80 that this statistic is shown to be close to 5%. *F-GG* and *F-HF* are robust in all conditions manipulated. Type I error rates for *B-F* are always within the bounds [2.5%, 7.5%], the maximum value reached being 7.48%.

### Unbalanced designs with heterogeneity: positive pairing

#### Group effect

**Figure 7:**
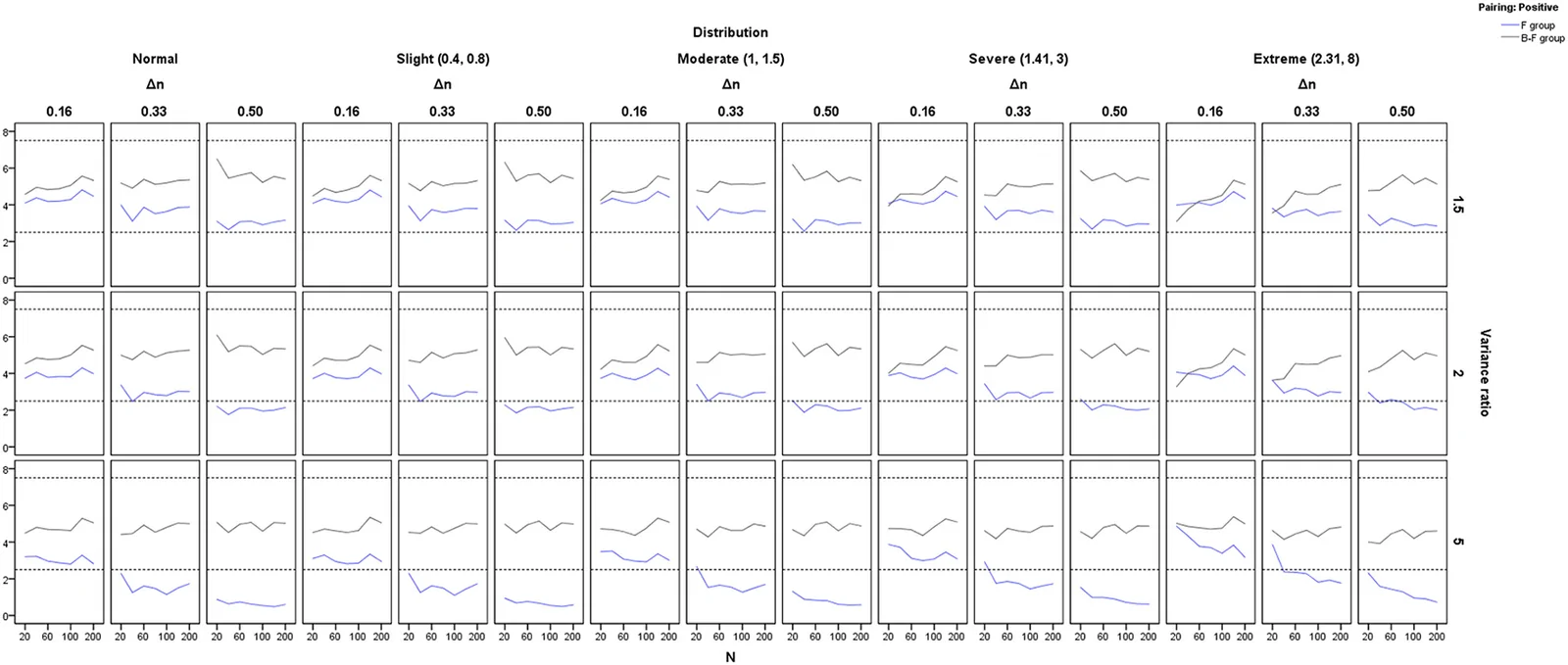
Type I error rates (in percentages) for the group effect in unbalanced designs with positive pairing and variance ratio greater than 1 (heterogeneity of variance) for the *F*-statistic and *B-F* as a function of distribution shape (in parenthesis: skewness, γ_1_, and kurtosis, γ_2_, coefficients), variance ratio, coefficient of sample size variation (Δ*n*), and sample size, across epsilon values.

Table 1 and Figure 7 display the results for the group effect when there is no homogeneity of variance (VR > 1) and the largest group size is associated with the largest value of variance (1890 conditions). We omitted non-sphericity because it is not a relevant variable for group effect, as already seen for the homogeneity condition with unbalanced designs. In this scenario, Type I error shows approximately the same pattern across distribution shapes and sample sizes, with the variables that most affect it being the VR and the inequality of group sizes (Δ*n*). With VR = 1.5, the *F*-statistic is robust in all conditions of Δ*n*. With VR = 2 and 5, it is robust with Δ*n* = 0.16, but is conservative with greater inequality of group sizes. For example, with Δ*n* = 0.50 it may reach a minimum Type I error of 0.40%. *B-F* remains within the bounds [2.5%, 7.5%] to be considered robust, reaching a minimum and maximum Type I error of 2.64% and 6.90%, respectively.

**Table 1:** Descriptive statistics for Type I error (in percentages) for the group effect in unbalanced designs and positive pairing (heterogeneity of variance: variance ratio greater than 1) for the *F*-statistic and *B-F* as a function of variance ratio (VR), and coefficient of sample size variation (Δ*n*).

	VR	Δ*n*	*F*				*B-F*			
Pairing			Min	Max	*M*	SD	Min	Max	*M*	SD
Positive	1.5	0.16	3.68	5.20	4.29	0.29	2.84	5.94	4.80	0.57
		0.33	2.96	4.12	3.64	0.27	2.64	5.68	4.96	0.43
		0.50	*2.46*	3.88	3.01	0.23	4.20	6.90	5.49	0.40
	2	0.16	3.44	4.54	3.93	0.26	3.00	5.88	4.77	0.52
		0.33	*2.24*	3.82	2.95	0.29	3.06	5.52	4.83	0.42
		0.50	*1.58*	3.52	*2.16*	0.26	3.46	6.36	5.22	0.44
	5	0.16	2.68	5.40	3.29	0.48	4.14	5.56	4.82	0.32
		0.33	*0.98*	4.46	*1.81*	0.56	3.94	5.22	4.67	0.29
		0.50	*0.40*	3.06	*0.87*	0.39	3.64	5.48	4.72	0.36
	Total	0.16	2.68	5.40	3.84	0.55	2.84	5.94	4.80	0.48
		0.33	*0.98*	4.46	2.80	0.85	2.64	5.68	4.82	0.40
		0.50	*0.40*	3.88	*2.01*	0.93	3.46	6.90	5.15	0.51

Type I error rates < 2.5% are in italics (conservative). n = 210 conditions for each cell. N = 630 conditions for total cells.

#### Time effect

**Figure 8:**
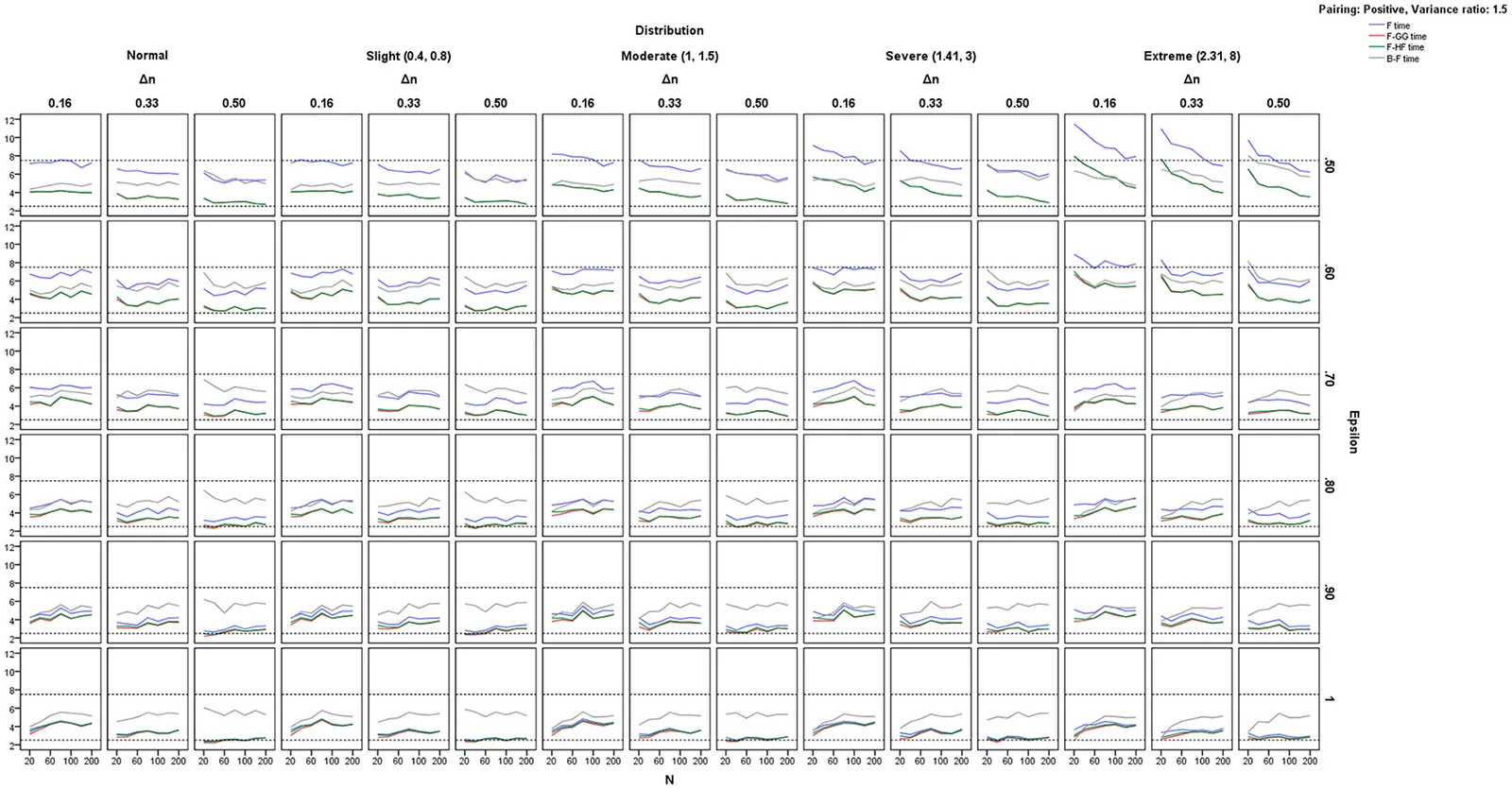
Type I error rates (in percentages) for the time effect in unbalanced designs with positive pairing and variance ratio = 1.5 (heterogeneity of variance) for the *F*-statistic, *F-GG, F-HF*, and *B-F* as a function of distribution shape (in parenthesis: skewness, γ_1_, and kurtosis, γ_2_, coefficients), coefficient of sample size variation (Δ*n*), epsilon value, and sample size.

**Figure 9:**
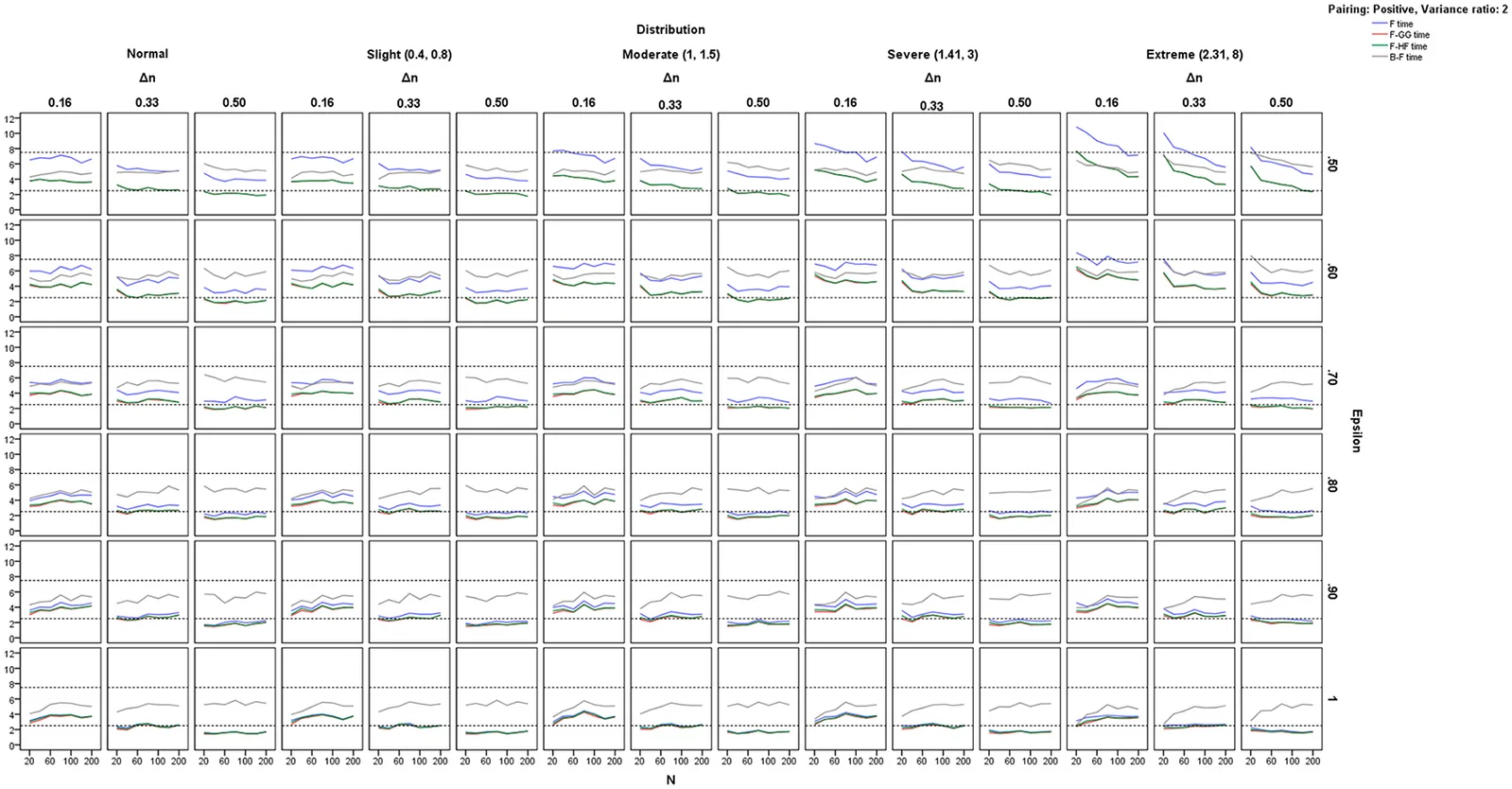
Type I error rates (in percentages) for the time effect in unbalanced designs with positive pairing and variance ratio = 2 (heterogeneity of variance) for the *F*-statistic, *F-GG, F-HF*, and *B-F* as a function of distribution shape (in parenthesis: skewness, γ_1_, and kurtosis, γ_2_, coefficients), coefficient of sample size variation (Δ*n*), epsilon value, and sample size.

**Figure 10:**
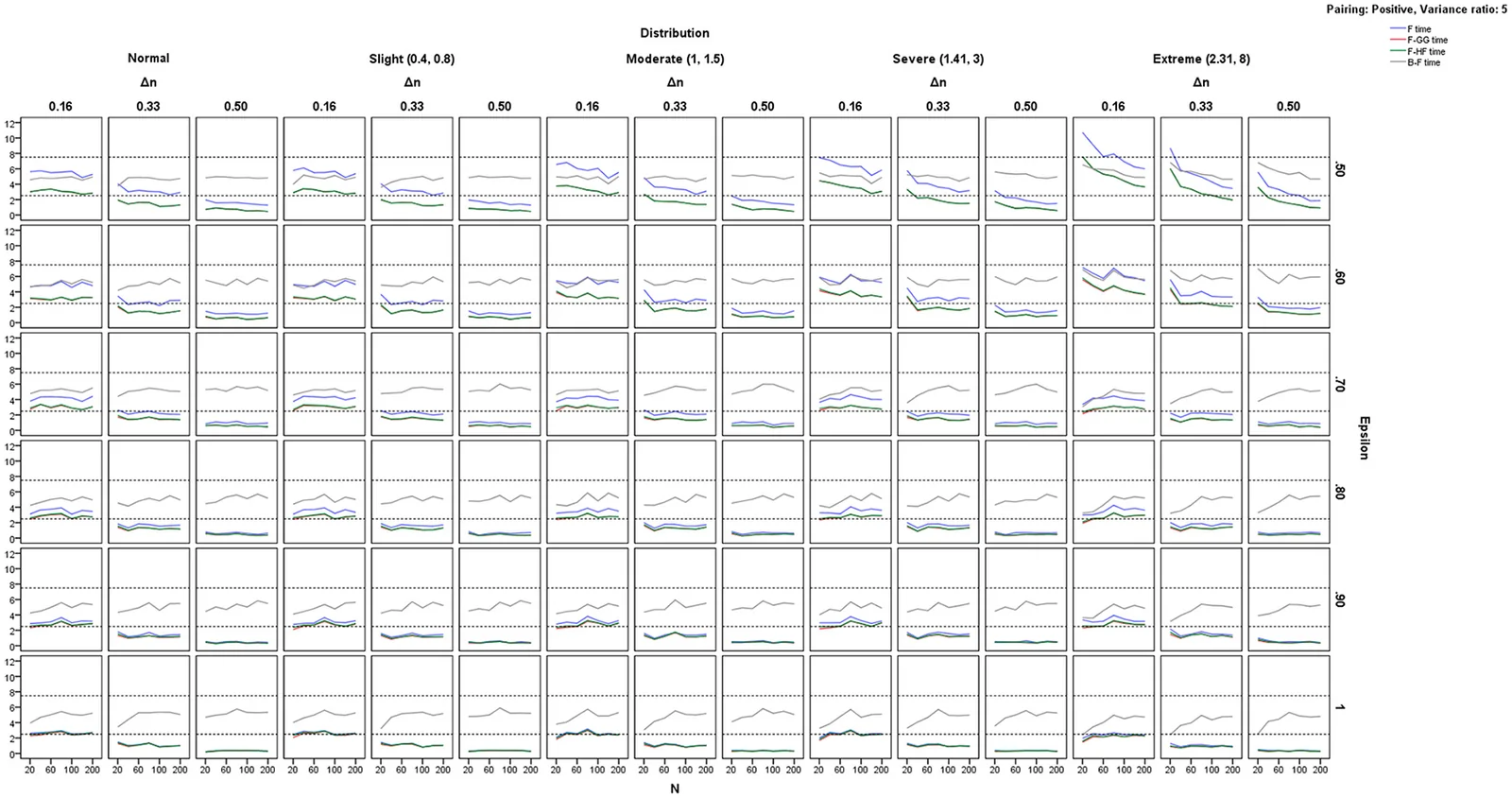
Type I error rates (in percentages) for the time effect in unbalanced designs with positive pairing and variance ratio = 5 (heterogeneity of variance) for the *F*-statistic, *F-GG, F-HF*, and *B-F* as a function of distribution shape (in parenthesis: skewness, γ_1_, and kurtosis, γ_2_, coefficients), coefficient of sample size variation (Δ*n*), epsilon value, and sample size.

Figures 8–10 show results for the time effect. The behavior of the *F*-statistic depends on all the variables manipulated, becoming liberal in some conditions with a low value of $\hat{\varepsilon }$, especially with extreme violation of normality, and conservative with high values of $\hat{\varepsilon }$. Overall, it is robust for all distributions and VRs for medium values of $\hat{\varepsilon }$ and Δ*n* = 0.16. By contrast, *F-GG* and *F-HF* show conservative behavior as non-normality, the variance ratio, inequality of group sizes, and the $\hat{\varepsilon }$ value increase. With VR = 5, these procedures are only robust with a low value of $\hat{\varepsilon }$ and Δ*n* = 0.16, except with extreme deviation and *N* = 20. Regarding *B-F*, it generally shows robust behavior for the time effect with positive pairing, although it may become liberal in some conditions with Δ*n* = 0.50, and conservative with a high value of $\hat{\varepsilon }$. As a general rule, it is robust in all conditions with *N* > 20.

#### Group × time interaction

**Figure 11:**
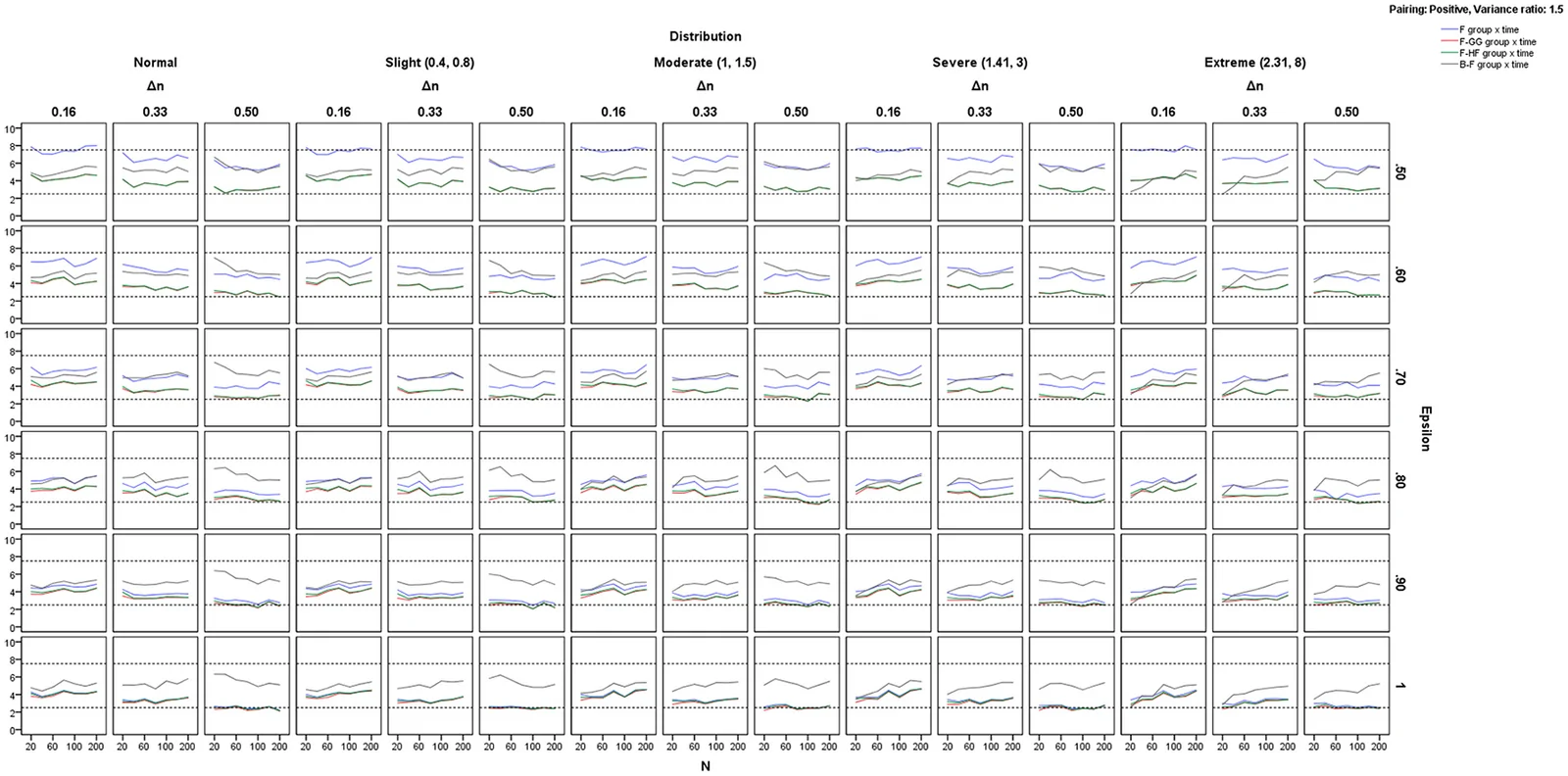
Type I error rates (in percentages) for the group × time interaction in unbalanced designs with positive pairing and variance ratio = 1.5 (heterogeneity of variance) for the *F*-statistic, *F-GG, F-HF*, and *B-F* as a function of distribution shape (in parenthesis: skewness, γ_1_, and kurtosis, γ_2_, coefficients), coefficient of sample size variation (Δ*n*), epsilon value, and sample size.

**Figure 12:**
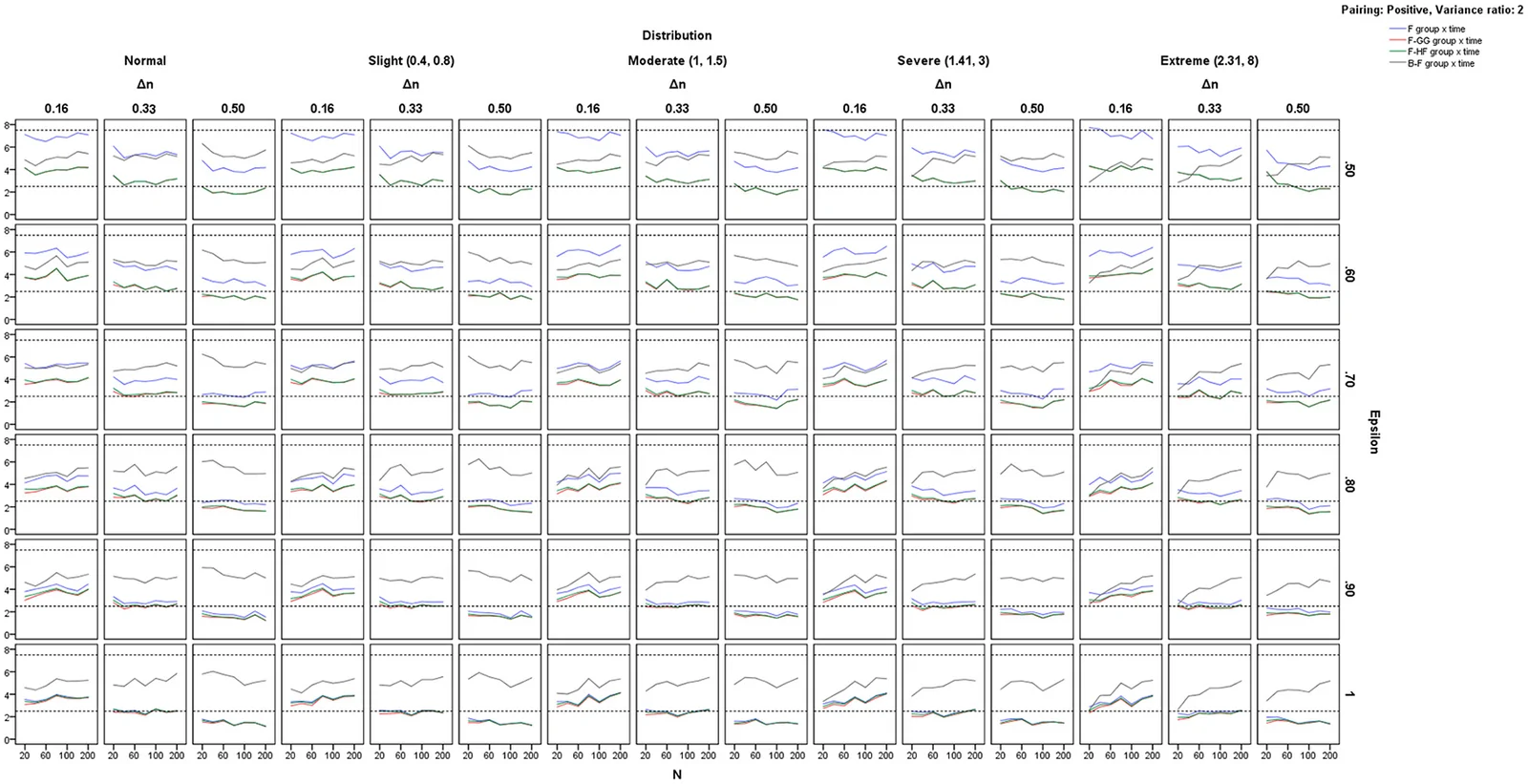
Type I error rates (in percentages) for the group × time interaction in unbalanced designs with positive pairing and variance ratio = 2 (heterogeneity of variance) for the *F*-statistic, *F-GG, F-HF*, and *B-F* as a function of distribution shape (in parenthesis: skewness, γ_1_, and kurtosis, γ_2_, coefficients), coefficient of sample size variation (Δ*n*), epsilon value, and sample size.

**Figure 13:**
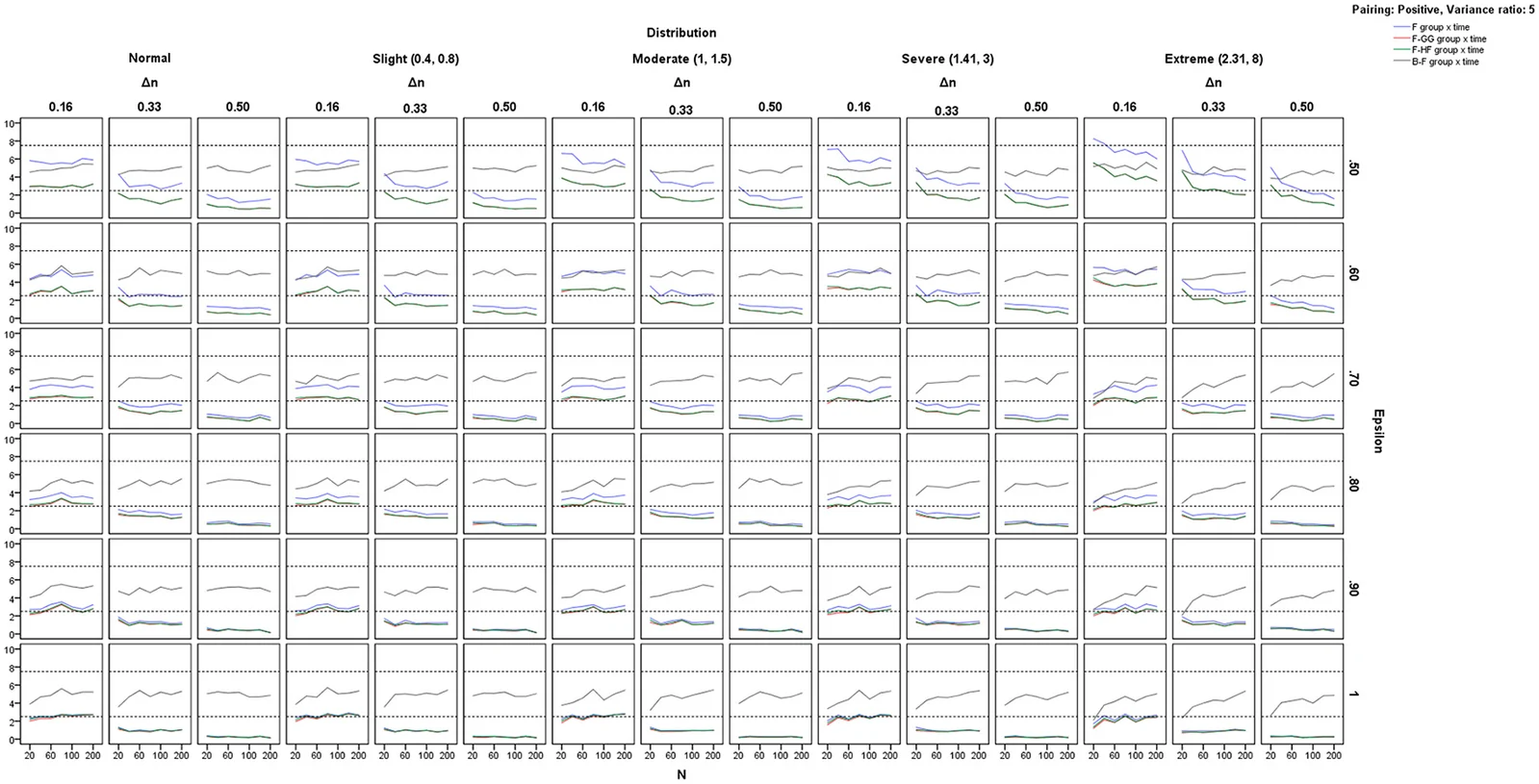
Type I error rates (in percentages) for the group × time interaction in unbalanced designs with positive pairing and variance ratio = 5 (heterogeneity of variance) for the *F*-statistic, *F-GG, F-HF*, and *B-F* as a function of distribution shape (in parenthesis: skewness, γ_1_, and kurtosis, γ_2_, coefficients), coefficient of sample size variation (Δ*n*), epsilon value, and sample size.

Figures 11–13 show the results for the group × time interaction. The *F*-statistic shows liberal behavior in some conditions with the lowest value of $\hat{\varepsilon }$, and a tendency to be conservative with higher values of $\hat{\varepsilon }$, becoming more conservative with higher VR, greater deviation from normality, and greater group inequality. As for the time effect, it is generally robust for all distributions and VRs for medium values of $\hat{\varepsilon }$ and Δ*n* = 0.16. *F-GG* and *F-HF* are robust under some scenarios with Δ*n* = 0.16–0.33, and tend to be conservative as non-normality, the variance ratio, inequality of group sizes, and the $\hat{\varepsilon }$ value increase. Overall, with VR = 5, *F-GG* and *F-HF* are generally conservative under all distributions, Δ*n*, and sample size conditions. In contrast to these statistics, *B-F* is generally robust with positive pairing for the interaction effect. It is only conservative in a few cases, with $\hat{\varepsilon }$ ≥ 0.90, *N* = 20, and extreme violation of normality. As a general rule, it is robust in all conditions with *N* > 20.

### Unbalanced designs with heterogeneity: negative pairing

#### Group effect

**Figure 14:**
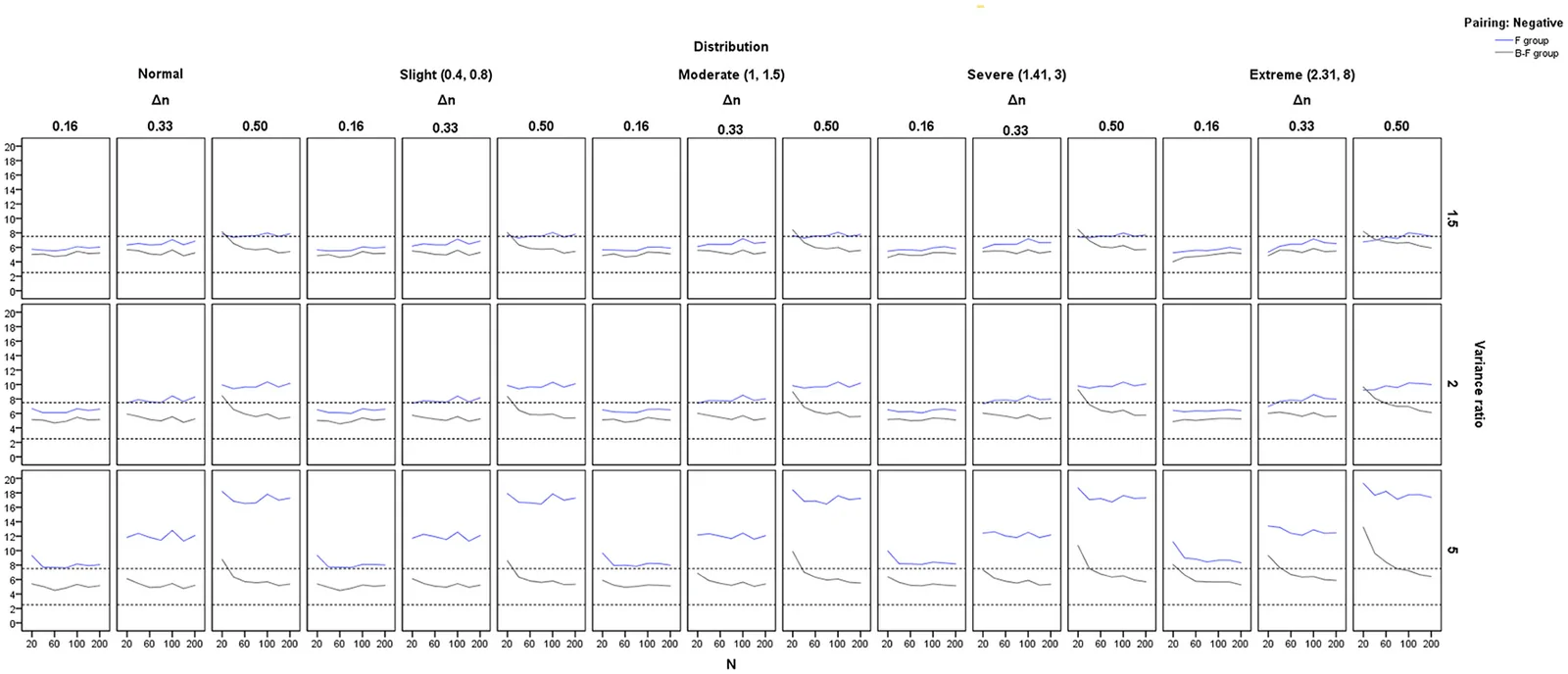
Type I error rates (in percentages) for the group effect in unbalanced designs with negative pairing and variance ratio greater than 1 (heterogeneity of variance) for the *F*-statistic and *B-F* as a function of distribution shape (in parenthesis: skewness, γ_1_, and kurtosis, γ_2_, coefficients), variance ratio, coefficient of sample size variation (Δ*n*), and sample size, across epsilon values.

Table 2 and Figure 14 show the results when there is no homogeneity of variance (VR > 1) and the largest group size is associated with the smallest value of variance (1890 conditions). Type I error of the *F*-statistic is more affected under a high variance ratio and large inequality of group sizes. As can be observed in Table 2, with VR = 1.5 and 2, it is only robust with Δ*n* = 0.16. With VR = 5, it is liberal in all conditions of Δ*n*, reaching a Type I error as large as 19.70%. Conversely, *B-F* is robust with VR = 1.5 and 2, with Δ*n* = 0.16 and 0.33, and becomes liberal with Δ*n* = 0.50 and small sample sizes (*N* = 20). With VR = 5, it tends to be liberal as the deviation from normality and inequality of group sizes increase, especially with small sample size. In this case, *B-F* reaches robustness with *N* > 20 for Δ*n* = 0.16, *N* > 60 for Δ*n* = 0.33, and *N* > 80 for Δ*n* = 0.50. Its maximum Type I error is 13.78%.

**Table 2:** Descriptive statistics for Type I error (in percentages) for the group effect in unbalanced designs and negative pairing (heterogeneity of variance: variance ratio greater than 1) for the *F*-statistic and *B-F* as a function of variance ratio (VR), and coefficient of sample size variation (Δ*n*).

	VR	Δ*n*	*F*				*B-F*			
Pairing			Min	Max	*M*	SD	Min	Max	*M*	SD
Negative	1.5	0.16	5.10	6.38	5.74	0.28	3.62	5.76	4.99	0.33
		0.33	5.22	**7.92**	6.51	0.45	4.50	6.28	5.35	0.34
		0.50	6.44	**8.30**	**7.59**	0.34	4.88	**8.94**	6.33	0.94
	2	0.16	5.78	6.98	6.37	0.26	4.08	5.80	5.10	0.28
		0.33	6.72	**9.14**	**7.84**	0.45	4.54	6.90	5.51	0.43
		0.50	**8.74**	**10.64**	**9.82**	0.39	4.96	**10.14**	6.57	1.19
	5	0.16	7.34	**11.80**	**8.38**	0.77	3.98	**8.86**	5.37	0.69
		0.33	**11.08**	**14.38**	**12.16**	0.56	4.56	**10.64**	5.80	0.96
		0.50	**16.06**	**19.70**	**17.35**	0.72	4.80	**13.78**	6.86	1.79
	Total	0.16	5.10	**11.80**	6.83	1.23	3.62	**8.86**	5.15	0.49
		0.33	5.22	**14.38**	**8.84**	2.46	4.50	**10.64**	5.55	0.66
		0.50	6.44	**19.70**	**11.59**	4.21	4.80	**13.78**	6.59	1.37

Type I error rates < 2.5% are in italics (conservative) and >7.5 are in bold (liberal). n = 210 conditions for each cell. N = 630 conditions for total cells.

#### Time effect

**Figure 15:**
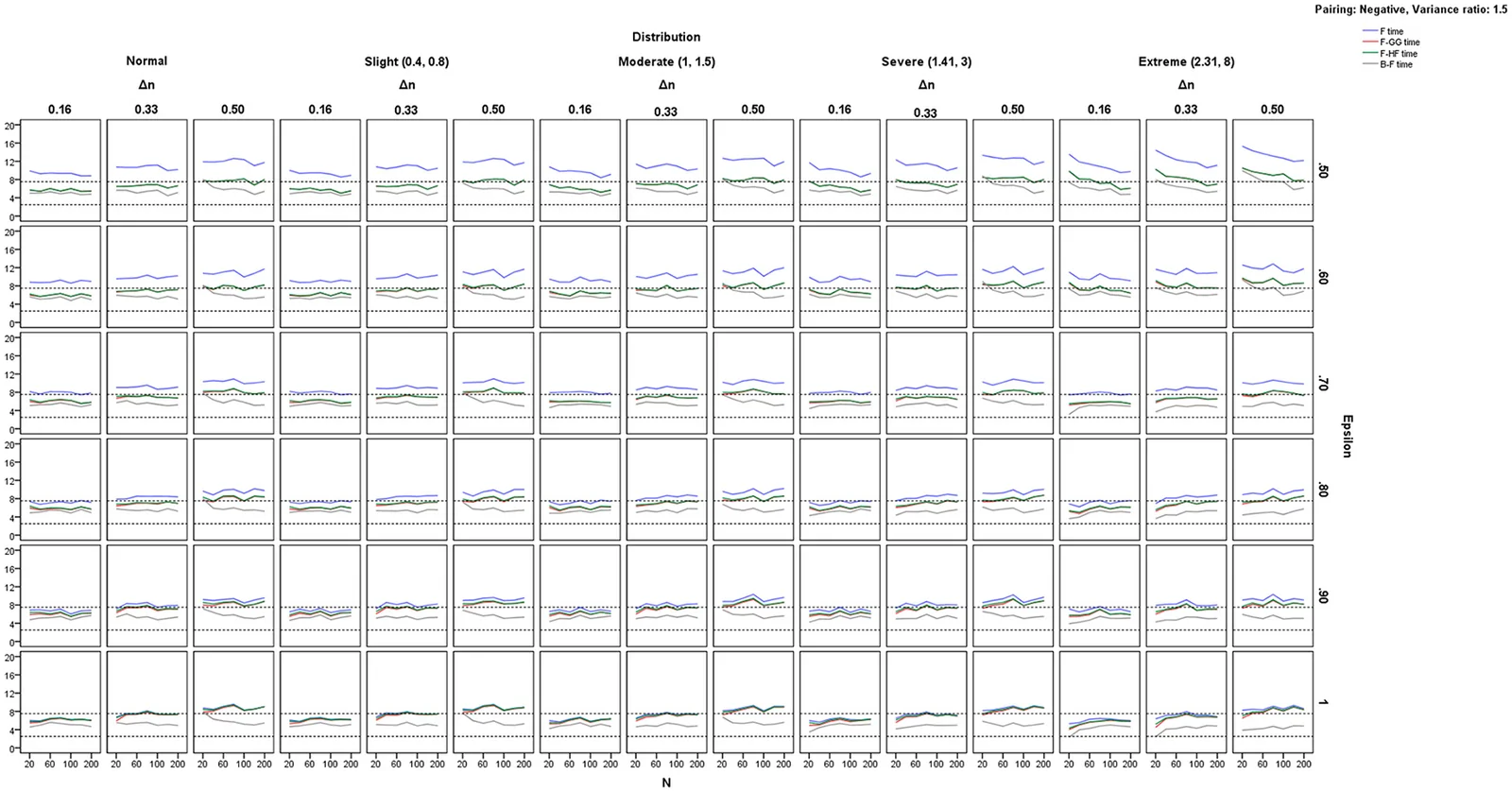
Type I error rates (in percentages) for the time effect in unbalanced designs with negative pairing and variance ratio = 1.5 (heterogeneity of variance) for the *F*-statistic, *F-GG, F-HF*, and *B-F* as a function of distribution shape (in parenthesis: skewness, γ_1_, and kurtosis, γ_2_, coefficients), coefficient of sample size variation (Δ*n*), epsilon value, and sample size.

**Figure 16:**
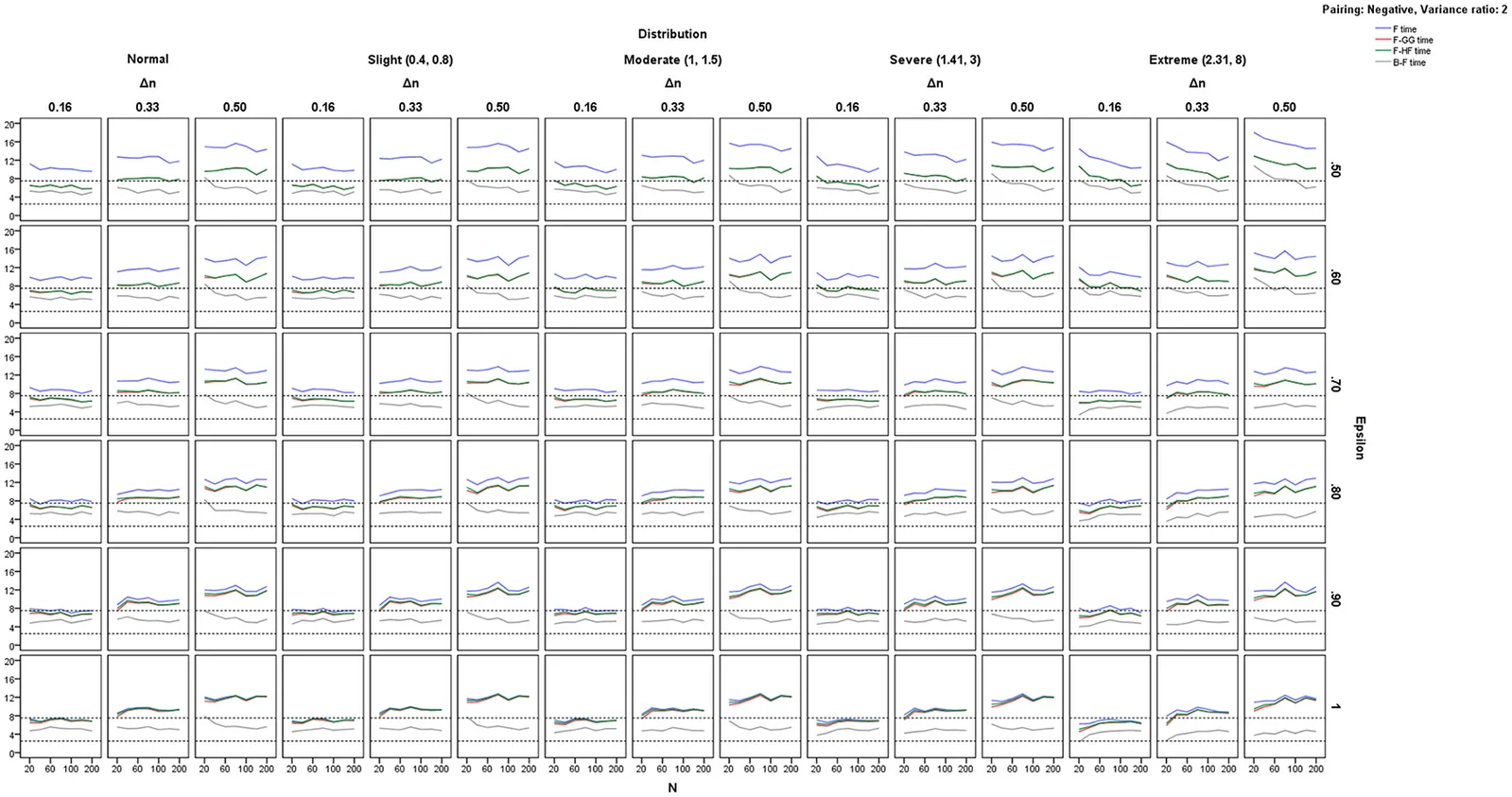
Type I error rates (in percentages) for the time effect in unbalanced designs with negative pairing and variance ratio = 2 (heterogeneity of variance) for the *F*-statistic, *F-GG, F-HF*, and *B-F* as a function of distribution shape (in parenthesis: skewness, γ_1_, and kurtosis, γ_2_, coefficients), coefficient of sample size variation (Δ*n*), epsilon value, and sample size.

**Figure 17:**
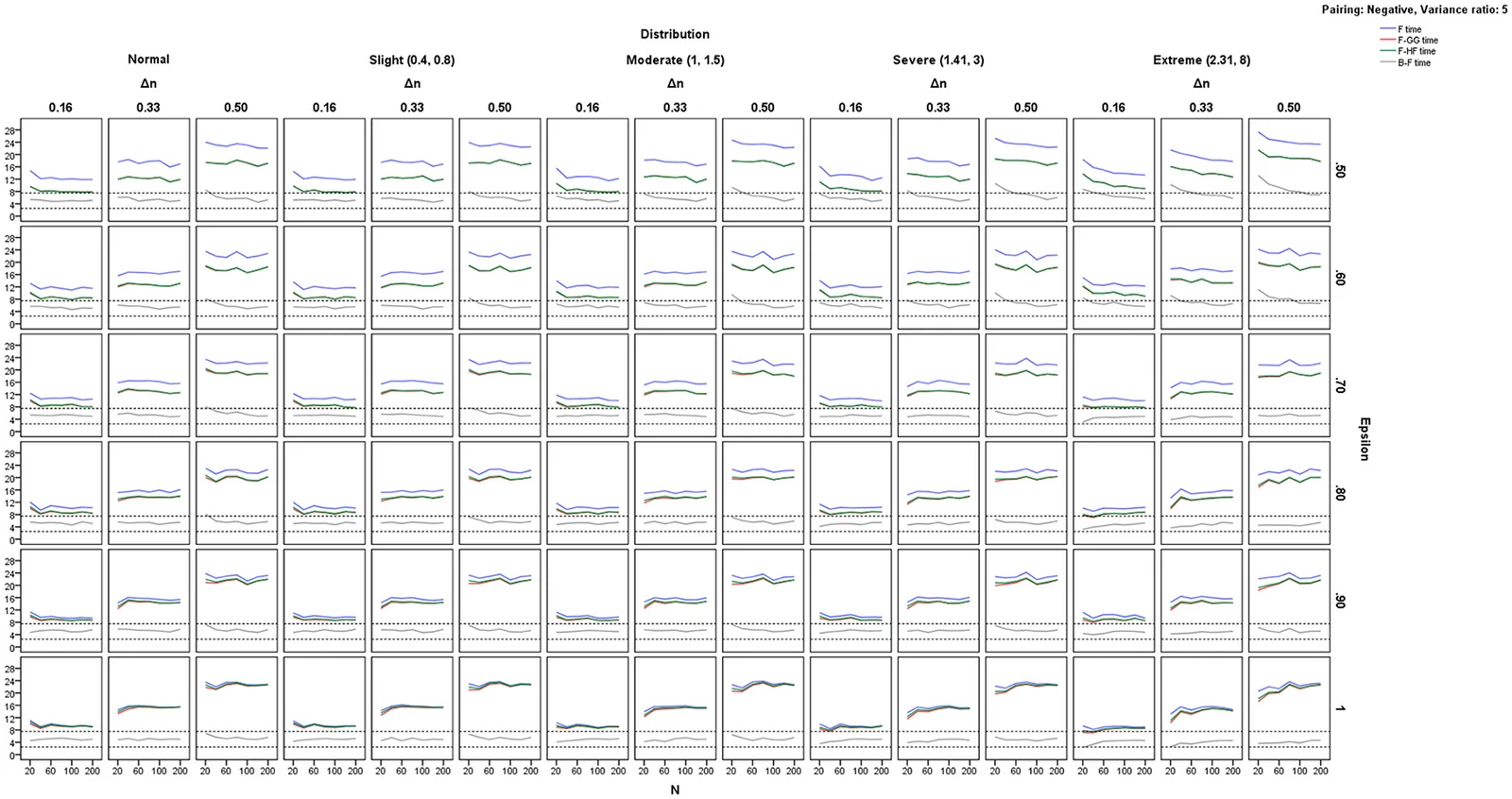
Type I error rates (in percentages) for the time effect in unbalanced designs with negative pairing and variance ratio = 5 (heterogeneity of variance) for the *F*-statistic, *F-GG, F-HF*, and *B-F* as a function of distribution shape (in parenthesis: skewness, γ_1_, and kurtosis, γ_2_, coefficients), coefficient of sample size variation (Δ*n*), epsilon value, and sample size.

Figures 15–17 show the results for the time effect. The *F*-statistic is only robust with VR = 1.5 and with Δ*n* = 0.16 and $\hat{\varepsilon }$ ≥ 0.90 for all distributions, except for extreme deviation, when it is only robust with $\hat{\varepsilon }$ = 1. It is generally liberal for the remaining values of $\hat{\varepsilon }$. Its behavior is more liberal the higher the VR, the higher the Δ*n*, and the greater the violation of normality. Its Type I error rate can reach 27.34%. The same behavior, but less pronounced, is shown by *F-GG* and *F-HF*, which can reach Type I error rates of 23.24% and 23.50%, respectively. These procedures are only robust in some scenarios with VR ≤ 2 and Δ*n* = 0.16. *B-F* also shows a tendency toward liberality in some conditions with low values of $\hat{\varepsilon }$ and small sample size (maximum Type I error of 13.22%). Its behavior worsens with high values of variance ratio, more inequality of group sizes, and greater deviation from normality. As these scenarios worsen, larger sample sizes are required to achieve robustness. There is also a tendency to be conservative with small sample size and $\hat{\varepsilon }$ = 1, especially with extreme deviation from normality.

#### Group × time interaction

**Figure 18:**
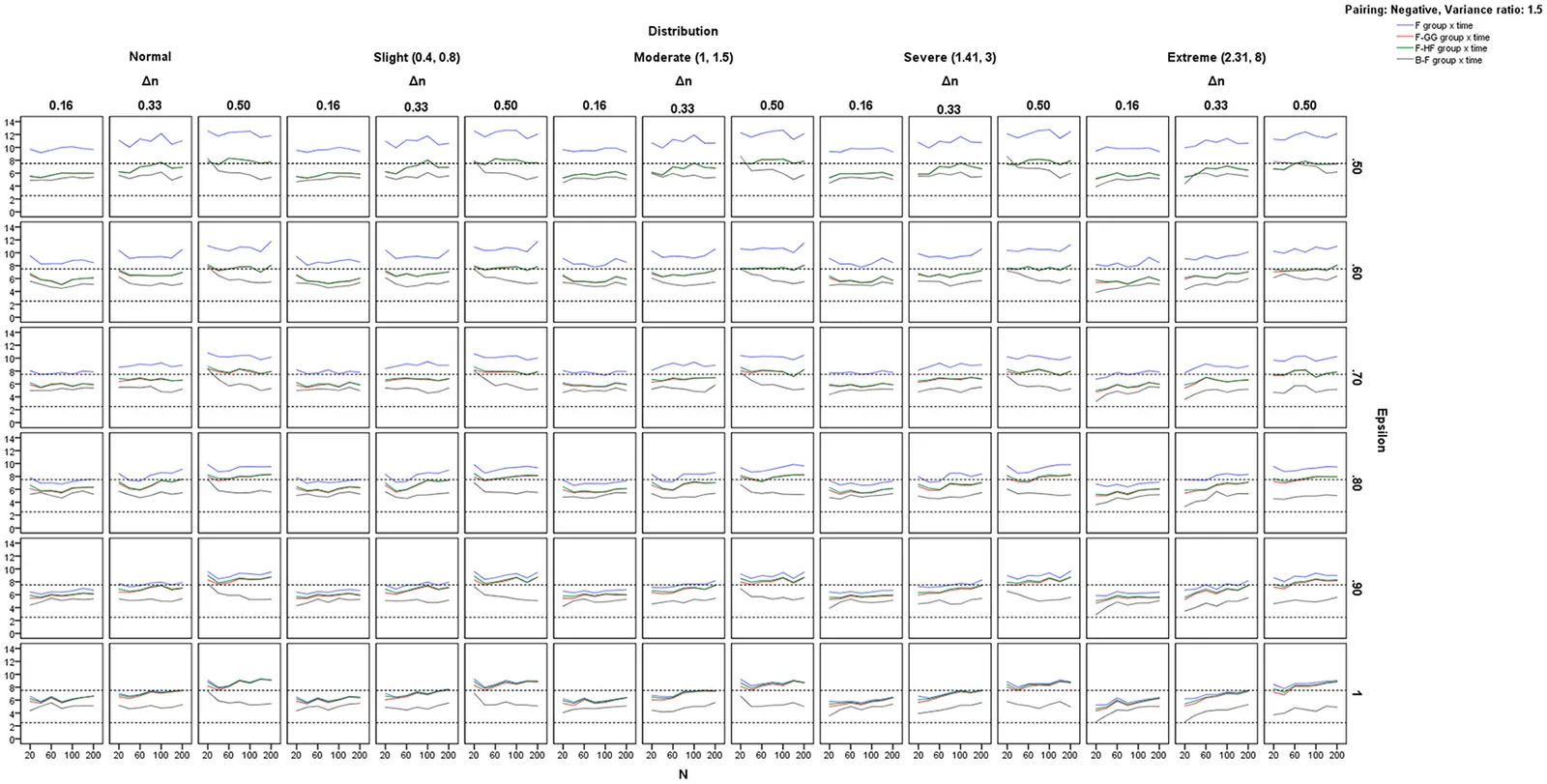
Type I error rates (in percentages) for the group × time interaction in unbalanced designs with negative pairing and variance ratio = 1.5 (heterogeneity of variance) for the *F*-statistic, *F-GG, F-HF*, and *B-F* as a function of distribution shape (in parenthesis: skewness, γ_1_, and kurtosis, γ_2_, coefficients), coefficient of sample size variation (Δ*n*), epsilon value, and sample size.

**Figure 19:**
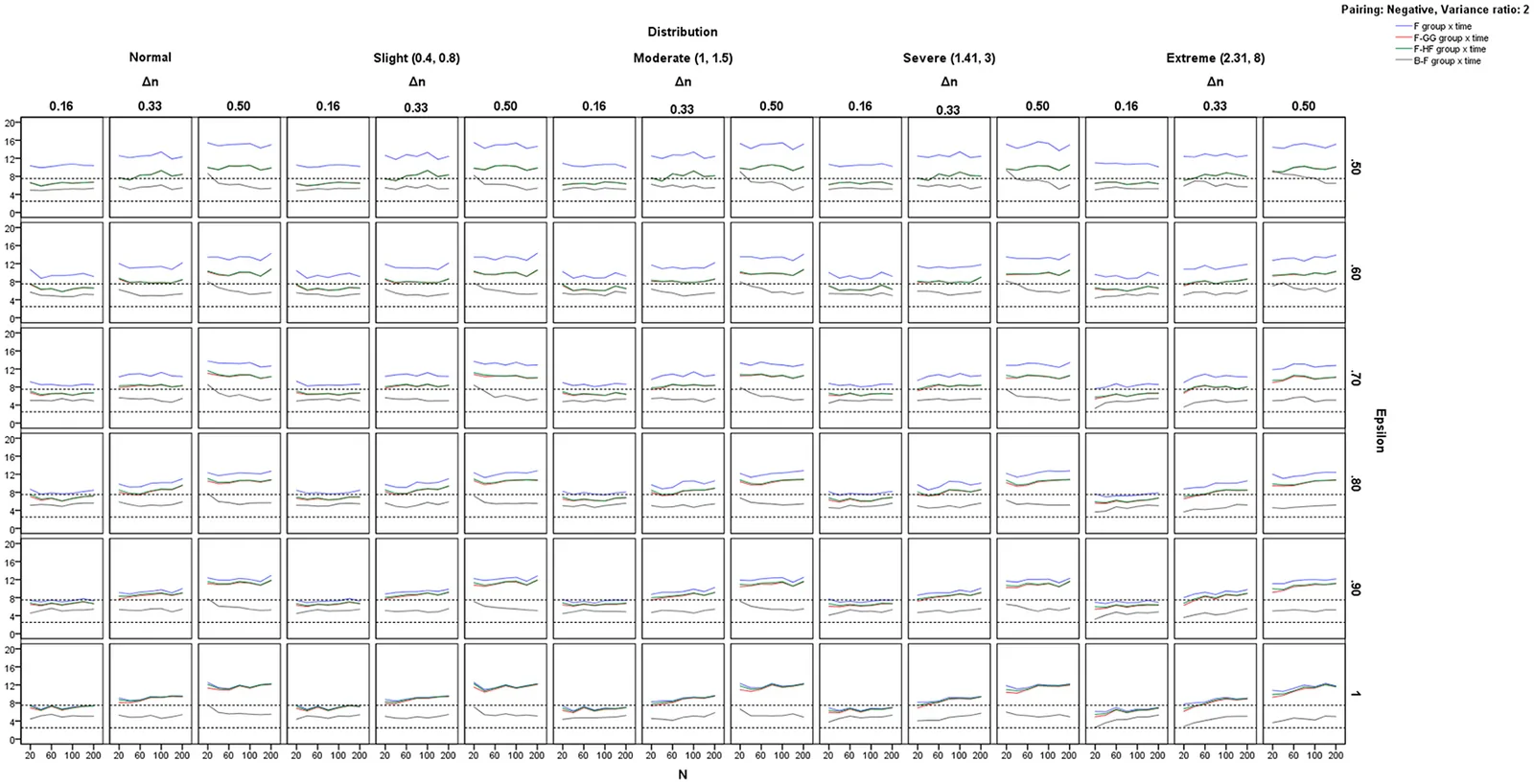
Type I error rates (in percentages) for the group × time interaction in unbalanced designs with negative pairing and variance ratio = 2 (heterogeneity of variance) for the *F*-statistic, *F-GG, F-HF*, and *B-F* as a function of distribution shape (in parenthesis: skewness, γ_1_, and kurtosis, γ_2_, coefficients), coefficient of sample size variation (Δ*n*), epsilon value, and sample size.

**Figure 20:**
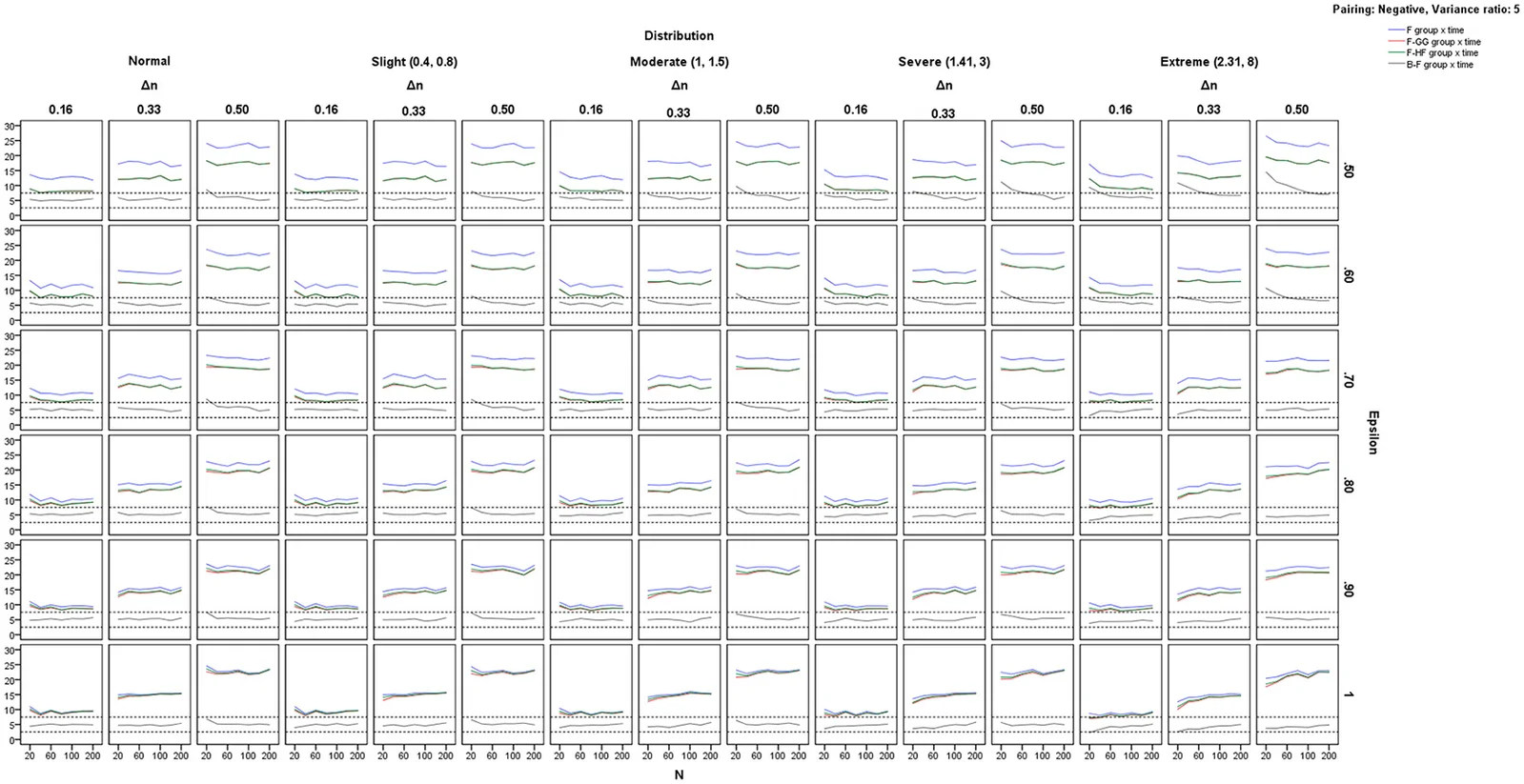
Type I error rates (in percentages) for the group × time interaction in unbalanced designs with negative pairing and variance ratio = 5 (heterogeneity of variance) for the *F*-statistic, *F-GG, F-HF*, and *B-F* as a function of distribution shape (in parenthesis: skewness, γ_1_, and kurtosis, γ_2_, coefficients), coefficient of sample size variation (Δ*n*), epsilon value, and sample size.

Figures 18–20 show the results for the group × time interaction. The *F*-statistic for this interaction is liberal under almost all conditions, especially with more unequal group sizes and higher VRs. Its Type I error can reach 26.54%. It is only robust with VR = 1.5 and with Δ*n* = 0.16 and $\hat{\varepsilon }$ ≥ 0.90 for all distributions. The same tendency toward liberality is shown by *F-GG* and *F-HF*, albeit less pronounced. These procedures can yield Type I error rates of 23.34% and 23.58%, respectively, and they are only robust in some scenarios with VR ≤ 2 and Δ*n* = 0.16. Finally, *B-F* is robust with VR ≤ 2 and Δ*n* = 0.16–0.33 with *N* > 20, showing a tendency toward liberality in some conditions with low values of $\hat{\varepsilon }$, small sample size, and greater inequality of group size. The lower the value of $\hat{\varepsilon }$, the higher the VR, the greater the inequality of group sizes, and the greater the deviation from normality, the larger the sample size that is needed to achieve robustness. For example, with $\hat{\varepsilon }$ = 0.50, VR = 5, and Δ*n* = 0.50 under extreme deviation from normality, *B-F* needs *N* > 60 to reach robustness. Its maximum Type I error is 14.50%.

## Discussion

The aim of this study was to provide new evidence on the behavior of the *F*-statistic, adjusted *F*-tests, and the bootstrap method (*B-F*) in split-plot designs in the presence of parametric assumption violations, clarifying the conditions in which each of them may be a suitable procedure. To this end, we conducted a Monte Carlo simulation study and manipulated the following variables: Distribution shape (normal, as well as slight, moderate, severe, and extreme deviation from normality), sphericity by epsilon values (from $\hat{\varepsilon }$ = 0.50 to 1), total sample size (*N* from 20 to 200), coefficient of group size variation (Δ*n* = 0, 0.16, 0.33, and 0.50), ratio of the variance-covariance matrices between the two groups (1:1, 1:1.5, 1:2, and 1:5), and type of pairing of variance with group sample size (null, positive, and negative). The results are discussed below according to the type of design and the variables manipulated.

### Balanced designs

In balanced designs, and for the group effect, both the *F*-statistic and *B-F* control Type I error under heterogeneity of variance and non-normality, in the conditions studied here. Logically, Type I error is not affected by violation of sphericity, as this is not a relevant assumption for the between-subjects factor. These results are in line with previous research showing the robustness of the *F*-statistic in balanced designs (Blanca et al., 2018b; Patrick, 2007; Yigit and Gökpinar, 2010).

For time and interaction effects, the *F*-statistic is liberal with low epsilon values ($\hat{\varepsilon }$ ≤ 0.70), reflecting the effect of sphericity violation (Blanca et al., 2023b, 2024; Fernández et al., 2007; Keselman et al., 1996; Keselman and Rogan, 1980; Rogan et al., 1979). The *F*-statistic performs worse than *F-GG* and *F-HF*, which are robust, except with severe and extreme normality violation, $\hat{\varepsilon }$ ≤ 0.60, and small samples, in which case they are liberal. Although both adjusted *F*-tests have been recommended when the groups have equal sample sizes (Keselman et al., 1995; Rogan et al., 1979), our results show that, in general, they may be used for $\hat{\varepsilon }$ ≥ 0.60 and *N* > 20. By contrast, *B-F* is robust in almost all conditions, although it tends to be conservative with extreme violation of normality, $\hat{\varepsilon }$ = 1, and *N* = 20. This tendency to be conservative with high values of $\hat{\varepsilon }$ has been reported previously (Berkovits et al., 2000; Blanca et al., 2025; Vallejo et al., 2006).

### Unbalanced designs with homogeneity of variance-covariance matrices

For the group effect, and in unbalanced designs (Δ*n* > 0) when the homogeneity assumption is satisfied, the *F*-statistic is robust and is unaffected by non-normality and non-sphericity. Other studies have concluded the same (Lee and Ahn, 2003; Yigit and Gökpinar, 2010), which is logical since the homogeneity assumption is satisfied. In these cases, *B-F* is also generally robust, although it requires *N* > 20 if there are very unequal group sizes (Δ*n* = 0.50).

For time and interaction effects, the *F*-statistic is liberal for $\hat{\varepsilon }$ ≤ 0.60 and robust with $\hat{\varepsilon }$ ≥ 0.70, although its Type I error is closer to 5% when $\hat{\varepsilon }$ ≥ 0.80. *F-GG* and *F-HF* are generally robust, except with extreme deviation from both normality and sphericity that they are liberal. In these cases, for both time and interaction effects, we need *N* > 60 if $\hat{\varepsilon }$ = 0.50, and *N* > 20 if $\hat{\varepsilon }$ = 0.60. *B-F* shows a similar behavior, and overall it requires *N* > 40 if $\hat{\varepsilon }$ ≤ 0.60.

### Unbalanced designs with heterogeneity of variance-covariance matrices and positive pairing

When the homogeneity assumption is not satisfied, the behavior of the *F*-statistic for the group effect depends on the pairing between variances and group sizes. With positive pairing, the *F*-statistic tends to be conservative if the group sizes are very unequal, with the situation getting worse the greater the heterogeneity of variance. By contrast, *B-F* remains within the bounds [2.5%, 7.5%] to be considered robust.

For time and interaction effects, the *F*-statistic tends to be liberal in some conditions with the lowest value of $\hat{\varepsilon }$, although there is a tendency for it to be conservative with higher values of $\hat{\varepsilon }$. In general, it is robust to all distributions and VRs for medium values of $\hat{\varepsilon }$ and Δ*n* = 0.16. *F-GG* and *F-HF* tend to be conservative, a tendency that is aggravated with greater heterogeneity of variance, more unequal group sizes, and higher $\hat{\varepsilon }$ values. These results are in line with previous research (Algina, 1994; Algina and Oshima, 1994, 1995; Keselman et al., 1995, 1999). According to our results, *F-GG* and *F-HF* can, as a general rule, be used in all distributions when: (a) VR = 1.5, Δ*n* = 0.16 and 0.33, and *N* > 20; (b) VR = 2, 0.60 ≤ $\hat{\varepsilon }$ ≤ 0.90, and Δ*n* = 0.16; and (c) VR = 5, $\hat{\varepsilon }$ ≤ 0.60, Δ*n* = 0.16, and *N* > 20. As for *B-F*, it generally shows robust behavior, except in several scenarios with extreme deviation from normality, very unequal group sizes (Δ*n* = 0.50), and small sample size (*N* = 20), in which cases it may be liberal with a low value $\hat{\varepsilon }$ or conservative when the value of $\hat{\varepsilon }$ is high. Overall, *B-F* can be used with *N* > 20. These results extend knowledge about the behavior of *B-F*, highlighting its worsening performance with very unequal group sizes. It should be noted that previous research only included a maximum value for the group size coefficient of variation of 0.33 (Vallejo et al., 2006, 2010).

### Unbalanced designs with heterogeneity of variance-covariance matrices and negative pairing

With negative pairing, both the *F*-statistic and *B-F* tend to be liberal for the group effect, reaching Type I error rates of 19.70% and 13.78%, respectively. If group sizes are not very unequal (Δ*n* = 0.16), the *F*-statistic is robust with VR ≤ 2; otherwise, it tends to be liberal, regardless of distribution shape, group size or variance ratio. Regarding *B-F*, the results also indicate a tendency toward liberality, although its behavior is better than that of the *F*-statistic. *B-F* is robust with VR ≤ 2 and Δ*n* ≤ 0.33, and with VR ≤ 2, Δ*n* = 0.50, and *N* > 20. With VR = 5, a large sample size is needed to achieve robustness, depending on the inequality of group sizes. In the worst scenario, *N* > 80 is required to reach robustness.

Regarding time and interaction effects, the *F*-statistic and the two adjusted-*F* tests are generally liberal, with all three reaching Type I error rates above 20%. The *F*-statistic is only an analytic option with VR = 1.5, $\hat{\varepsilon }$ = 1, and Δ*n* = 0.50. The two adjusted-*F* tests are only robust in some conditions with VR ≤ 2 and Δ*n* = 0.16. When there is large inequality in group sizes, none of these three statistics should be used. The tendency to liberality has been previously reported, also highlighting the effect of inequality of group sizes (Algina, 1994; Algina and Oshima, 1994, 1995; Keselman and Keselman, 1990; Keselman et al., 1995, 1999). *B-F* outperforms all these statistics, but consistently shows a tendency toward liberality in some conditions with low values of $\hat{\varepsilon }$, small sample size, and large inequality of group sizes. In these cases, a larger sample size is needed to achieve robustness. As for the group effect, with extreme conditions *B-F* needs *N* > 80 to reach robustness.

## Conclusions: practical recommendations

The results of the present study serve to identify the conditions under which the *F*-statistic, adjusted *F*-tests, and *B-F* can be applied with a guarantee of robustness, as well as those conditions under which robustness is threatened. A guideline to the analysis of 3 × 2 split-plot designs that may be useful to applied researchers is provided below.

Table 3 summarizes the conditions under which the analyzed statistics can be used in balanced designs. For the group effect, the *F*-statistic and *B-F* are valid choices for analysis, regardless of whether the assumptions of normality and homogeneity are satisfied independently or in combination. For time and interaction effects, the *F*-statistic, as expected, is also suitable if sphericity is satisfied, whereas *B-F* would require *N* > 20 if $\hat{\varepsilon }$ = 1. In the event of sphericity violation, *F-GG* and *F-HF* maintain Type I error close to 5% with moderate violation of normality. For distributions with larger deviation from normality, both adjusted *F*-tests can be used with $\hat{\varepsilon }$ ≥ 0.70, but with lower $\hat{\varepsilon }$ values a large sample size is required ($\hat{\varepsilon }$ = 0.50, *N* > 60; $\hat{\varepsilon }$ = 0.60, and *N* > 20). As a rule of thumb, *B-F* can be used to test all three effects in all conditions studied here, provided that *N* > 20.

**Table 3:** Conditions in which the *F*-statistic, adjusted *F*-tests, and *B-F* can be used with balanced designs.

Balanced design, pairing null, all Δ*n*					
VR	Effect	Normal–Moderate (γ_1_ ≤ 1, γ_2_ ≤ 1.5)	Severe (γ_1_ = 1.41, γ_2_ = 3)	Extreme (γ_1_ = 2.31, γ_2_ = 8)	General rule
1, 1.5, 2, 5	G	*F*			*F*
		*B-F*			*B-F*
	T, G × T	*F*, $\hat{\varepsilon }\geq 0.80$		*F*, $\hat{\varepsilon }\geq 0.70$	*F*, $\hat{\varepsilon }\geq 0.80$
		*F-GG, F-HF*	*F-GG, F-HF*, $\hat{\varepsilon }=0.50$, *N*>20	*F-GG, F-HF*, $\hat{\varepsilon }=0.50$, *N*>60	*F-GG, F-HF*, $\hat{\varepsilon }=0.50$, *N*>60
			*F-GG, F-HF*, $\hat{\varepsilon }\geq 0.60$	*F-GG, F-HF*, $\hat{\varepsilon }=0.60$, *N*>20	*F-GG, F-HF*, $\hat{\varepsilon }=0.60$, *N*>20
				*F-GG, F-HF*, $\hat{\varepsilon }\geq 0.70$	*F-GG, F-HF*, $\hat{\varepsilon }\geq 0.70$
		*B-F*	*B-F*	*B-F*, $\hat{\varepsilon }\leq 0.90$	*B-F*, $\hat{\varepsilon }\leq 0.90$
				*B-F*, $\hat{\varepsilon }=1$, *N*>20	*B-F*, $\hat{\varepsilon }=1$, *N*>20

G, group effect; T, time effect; G × T, group × time effect; Δ*n*, coefficient of sample size variation; VR, variance ratio; γ_1_, skewness coefficient; γ_2_, kurtosis coefficient; N, total sample size; $\hat{\varepsilon }$, Greenhouse–Geisser epsilon; F, F-statistic; F-GG, Greenhouse–Geisser adjusted F-test; F-HF, Huynh–Feldt adjusted F-test; B-F, bootstrap-F.

Table 4 summarizes the conditions in which the analyzed statistics can be used in unbalanced designs with homogeneity of variance. The *F*-statistic can be used for the group effect, regardless of non-normality and unequal group sizes, as well as for the time and interaction effect when $\hat{\varepsilon }$ ≥ 0.70. *B-F* may also be a suitable alternative for all three effects, but for the group effect it requires *N* > 20 if there is large inequality of group sizes, while for the time and interaction effects, *N* > 40 is needed if $\hat{\varepsilon }$ ≤ 0.60. As for balanced designs, and in the event of sphericity violation, *F-GG* and *F-HF* maintain Type I error close to 5% with moderate violation of normality. For distributions with larger deviation from normality, both can be used with $\hat{\varepsilon }$ ≥ 0.70, but with lower $\hat{\varepsilon }$ values a large sample size is required ($\hat{\varepsilon }$ = 0.50, *N* > 60; $\hat{\varepsilon }$ = 0.60, *N* > 20).

**Table 4:** Conditions in which the *F*-statistic, adjusted *F*-tests, and *B-F* can be used for unbalanced designs with homogeneity of variance.

Unbalanced design, homogeneity of variance-covariance matrices				
VR	Effect	Normal–Severe (γ_1_ ≤ 1.41, γ_2_ ≤ 3)	Extreme (γ_1_ = 2.31, γ_2_ = 8)	General rule
1	G	*F*		*F*
		*B-F*, Δ*n* = 0.16–0.33		*B-F*, Δ*n* = 0.16–0.33
		*B-F*, Δ*n* = 0.50, *N*>20		*B-F*, Δ*n* = 0.50, *N*>20
	T, G × T	*F*, $\hat{\varepsilon }\geq 0.70$		*F*, $\hat{\varepsilon }\geq 0.70$
		*F-GG, F-HF*	*F-GG, F-HF*, $\hat{\varepsilon }=0.50$, *N*>60	*F-GG, F-HF*, $\hat{\varepsilon }=0.50$, *N*>60
			*F-GG, F-HF*, $\hat{\varepsilon }=0.60$, *N*>20	*F-GG, F-HF*, $\hat{\varepsilon }=0.60$, *N*>20
			*F-GG, F-HF*, $\hat{\varepsilon }\geq 0.70$	*F-GG, F-HF*, $\hat{\varepsilon }\geq 0.70$
		*B-F*, Δ*n* = 0.16–0.33	*B-F*, $\hat{\varepsilon }\leq 0.60$, *N*>40	*B-F*, $\hat{\varepsilon }\leq 0.60$, *N*>40
		*B-F*, Δ*n* = 0.50, *N*>20	*B-F*, $\hat{\varepsilon }\geq 0.70$	*B-F*, $\hat{\varepsilon }\geq 0.70$

G, group effect; T, time effect; G × T, group × time effect; Δ*n*, coefficient of sample size variation; VR, variance ratio; γ_1_, skewness coefficient; γ_2_, kurtosis coefficient; N, total sample size; $\hat{\varepsilon }$, Greenhouse–Geisser epsilon; F, F-statistic; F-GG, Greenhouse–Geisser adjusted F-test; F-HF, Huynh–Feldt adjusted F-test; B-F, bootstrap-F.

Table 5 summarizes the conditions in which the analyzed statistics can be used in unbalanced designs with heterogeneity of variance and positive pairing. For the group effect the *F*-statistic can generally only be used when the groups are of similar size (Δ*n* = 0.16), while for the time and interaction effects, it may, as a rule of thumb, be used with Δ*n* = 0.16 and 0.70 ≤ $\hat{\varepsilon }$ ≤ 0.80. *F-GG* and *F-HF* can, as a general rule, be used for the time and interaction effects in all distributions when: (a) VR = 1.5, Δ*n* = 0.16 and 0.33, and *N* > 20; (b) VR = 2, 0.60 ≤ $\hat{\varepsilon }$ ≤ 0.90, and Δ*n* = 0.16; and (c) VR = 5, $\hat{\varepsilon }$ ≤ 0.60, Δ*n* = 0.16, and *N* > 20. By contrast, *B-F* is the procedure that best controls the Type I error rate, and it can be used for all three effects with *N* > 20. Therefore, it is considered the best choice for analysis in cases where the largest group size has the largest variance value.

**Table 5:** Conditions in which the *F*-statistic, adjusted *F*-tests, and *B-F* can be used for unbalanced designs with heterogeneity of variance and positive pairing.

Unbalanced design, positive pairing				
VR	Effect	Normal–Severe (γ_1_ ≤ 1.41, γ_2_ ≤ 3)	Extreme (γ_1_ = 2.31, γ_2_ = 8)	General rule
1.5+	G	*F*		*F*
		*B-F*		*B-F*
	T, G × T	*F*, $0.70\leq \hat{\varepsilon }\leq 0.80$		*F*, $0.70\leq \hat{\varepsilon }\leq 0.80$
		*F-GG, F-HF*, Δ*n* = 0.16–0.33	*F-GG, F-HF*, $\hat{\varepsilon }=0.50$, *N* > 20	*F-GG, F-HF*, Δ*n* = 0.16–0.33, *N* > 20
			*F-GG, F-HF*, $\hat{\varepsilon }\geq 0.60$, Δ*n* = 0.16–0.33	
			*F-GG, F-HF*, $\hat{\varepsilon }=1$, Δ*n* = 0.16–0.33, *N* > 20	
		*B-F*	*B-F, N* > 20	*B-F, N* > 20
2+	G	*F*, Δ*n* = 0.16		*F*, Δ*n* = 0.16
		*B-F*		*B-F*
	T, G × T	*F*, $0.60\leq \hat{\varepsilon }\leq 0.80$, Δ*n* = 0.16–0.33	*F*, $0.70\leq \hat{\varepsilon }\leq 0.90$, Δ*n* = 0.16–0.33	*F*, $0.60\leq \hat{\varepsilon }\leq 0.80$, Δ*n* = 0.16–0.33
		*F*, $\hat{\varepsilon }\geq 0.90$, Δ*n* = 0.16	*F*, $\hat{\varepsilon }=1$, Δ*n* = 0.16	*F*, $\hat{\varepsilon }\geq 0.90$, Δ*n* = 0.16
		*F-GG, F-HF*, Δ*n* = 0.16	*F-GG, F-HF*, $0.60\leq \hat{\varepsilon }\leq 0.90$, Δ*n* = 0.16	*F-GG, F-HF*, $0.60\leq \hat{\varepsilon }\leq 0.90$, Δ*n* = 0.16
		*B-F*	*B-F, N* > 20	*B-F, N* > 20
5+	G	*F*, Δ*n* = 0.16		*F*, Δ*n* = 0.16
		*B-F*		*B-F*
	T, G × T	*F*, $\hat{\varepsilon }\leq 0.90$, Δ*n* = 0.16	*F*, $0.60\leq \hat{\varepsilon }\leq 0.90$, Δ*n* = 0.16	*F*, $0.60\leq \hat{\varepsilon }\leq 0.90$, Δ*n* = 0.16
		*F-GG, F-HF*, $\hat{\varepsilon }\leq 0.60$, Δ*n* = 0.16	*F-GG, F-HF*, $\hat{\varepsilon }\leq 0.60$, Δ*n* = 0.16, *N* > 20	*F-GG, F-HF*, $\hat{\varepsilon }\leq 0.60$, Δ*n* = 0.16, *N* > 20
		*B-F*	*B-F*, $\hat{\varepsilon }\leq 0.80$	*B-F, N* > 20
			*B-F*, $\hat{\varepsilon }\geq 0.90$, *N* > 20	

G, group effect; T, time effect; G × T, group × time effect; Δ*n*, coefficient of sample size variation; VR, variance ratio; γ_1_, skewness coefficient; γ_2_, kurtosis coefficient; N, total sample size; $\hat{\varepsilon }$, Greenhouse–Geisser epsilon; F, F-statistic; F-GG, Greenhouse–Geisser adjusted F-test; F-HF, Huynh–Feldt adjusted F-test; B-F, bootstrap-F.

In the majority of cases with positive pairing, the *F*-statistic and adjusted *F*-tests tend to be conservative. When a test is conservative, there is a greater probability of accepting the null hypothesis when it is false. Therefore, researchers can be sure in these scenarios that the statistical decision is correct if the null hypothesis is rejected.

Table 6 summarizes the conditions in which the analyzed statistics can be used in unbalanced designs with heterogeneity of variance and negative pairing. The *F*-statistic can only be used for the three effects if the group sizes are very similar (Δ*n* = 0.16), the variance ratio is close to 1.5, and sphericity is fulfilled ($\hat{\varepsilon }$ = 1). Similarly, the two adjusted *F*-tests may only be used with similar group sizes (Δ*n* = 0.16) and: (a) a variance ratio of 1.5 and $\hat{\varepsilon }$ ≥ 0.70; or (b) a variance ratio of 2, 70 ≤ $\hat{\varepsilon }$ ≤ 0.80, and *N* > 20. These two statistics, as well as the *F*-statistic, have serious limitations in conditions of negative pairing with very unequal variances, where they tend to be liberal. In these cases, researchers will more likely reject the null hypothesis when it is in fact true. Therefore, one can only be sure that the statistical decision is correct if the results lead to acceptance of the null hypothesis. Regarding *B-F*, it can be used for all three effects provided the sample size is adjusted based on the degree of inequality of the group sizes and the severity of the violation of both homogeneity and normality. As a rule of thumb, it can be used with: (a) Δ*n* = 0.16, *N* > 20; (b) Δ*n* = 0.33, *N* > 60; and (c) Δ*n* = 0.50, *N* > 80. Therefore, if a sample size greater than 80 is available, *B-F* can be used with guarantees of robustness, regardless of assumption violations.

**Table 6:** Conditions in which the *F*-statistic, adjusted *F*-tests, and *B-F* can be used for unbalanced designs with heterogeneity of variance and negative pairing.

Unbalanced design, negative pairing							
VR	Effect	Normal (γ_1_ = 0, γ_2_ *=* 0)	Slight (γ_1_ = 0.4, γ_2_ *=* 0.8)	Moderate (γ_1_ = 1, γ_2_ *=* 1.5)	Severe (γ_1_ = 1.41, γ_2_ *=* 3)	Extreme (γ_1_ = 2.31, γ_2_ *=* 8)	General rule
1.5–	G	*F, Δn* = 0.16					*F, Δn* = 0.16
		*B-F, Δn* = 0.16–0.33					*B-F, Δn* = 0.16–0.33
		*B-F, Δn* = 0.50, *N* > 20					*B-F, Δn* = 0.50, *N* > 20
	T, GxT	*F*, $\hat{\varepsilon }$ ≥ 0.90 Δ*n* = 0.16				*F*, $\hat{\varepsilon }$ = 1, Δ*n* = 0.16	*F*, $\hat{\varepsilon }$ = 1, Δ*n* = 0.16
		*F-GG, F-HF, Δn* = 0.16			*F-GG, F-HF*, $\hat{\varepsilon }$ ≥ 0.60, Δ*n* = 0.16	*F-GG, F-HF*, $\hat{\varepsilon }$ ≥ 0.70, Δ*n* = 0.16	*F-GG, F-HF*, $\hat{\varepsilon }$ ≥ 0.70, Δ*n* = 0.16
		*B-F, Δn* = 0.16–0.33					*B-F, Δn* = 0.16–0.33
		*B-F, Δn* = 0.50, *N* > 20					*B-F, Δn* = 0.50, *N* > 20
2–	G	*F, Δn* = 0.16					*F, Δn* = 0.16
		*B-F, Δn* = 0.16–0.33				*B-F, Δn* = 0.16–0.33	*B-F, Δn* = 0.16–0.33
		*B-F, Δn* = 0.50, *N* > 20				*B-F, Δn* = 0.50, *N* > 80	*B-F, Δn* = 0.50, *N* > 80
	T, GxT	*F-GG, F-HF, Δn* = 0.16, *N* > 20		*F-GG, F-HF*, $\hat{\varepsilon }$ ≥ 0.70, Δ*n* = 0.16		*F-GG, F-HF*, 0.70 ≤ $\hat{\varepsilon }$ ≤ 0.80, Δ*n* = 0.16	*F-GG, F-HF*, 0.70 ≤ $\hat{\varepsilon }$ ≤ 0.80, Δ*n* = 0.16, *N* > 20
		*B-F, Δn* = 0.16–0.33				*B-F*, $\hat{\varepsilon }$ ≤ 0.60, Δ*n* = 0.16–0.33, *N* > 20	*B-F, Δn* = 0.16–0.33, *N* > 20
		*B-F, Δn* = 0.50, *N* > 20				*B-F*, $\hat{\varepsilon }$ ≤ 0.60, Δ*n* = 0.50, *N* > 80	*B-F, Δn* = 0.50, *N* > 80
			*B-F*, 0.70 ≤ $\hat{\varepsilon }$ ≤ 0.90				
			*B-F*, $\hat{\varepsilon }$ = 1, *N* > 20				
5–	G	*B-F, Δn* = 0.16–0.33		*B-F, Δn* = 0.16–0.33	*B-F, Δn* = 0.16	*B-F, Δn* = 0.16, *N* > 20	*B-F, Δn* = 0.16, *N* > 20
		*B-F, Δn* = 0.50, *N* > 20		*B-F, Δn* = 0.50, *N* > 40	*B-F, Δn* = 0.33, *N* > 20	*B-F, Δn* = 0.33, *N* > 60	*B-F, Δn* = 0.33, *N* > 60
					*B-F, Δn* = 0.50, *N* > 60	*B-F, Δn* = 0.50, *N* > 80	*B-F, Δn* = 0.50, *N* > 80
	T, GxT	*B-F, Δn* = 0.16–0.33		*B-F, Δn* = 0.16–0.33	*B-F, Δn* = 0.16	*B-F*, $\hat{\varepsilon }$ ≤ 0.60, Δ*n* = 0.16–0.33, *N* > 60	*B-F, N* > 60
		*B-F, Δn* = 0.50, *N* > 20		*B-F, Δn* = 0.50, *N* > 40	*B-F, Δn* = 0.33, *N* > 20	*B-F*, 0.70 ≤ $\hat{\varepsilon }$ ≤ 0.90	
					*B-F, Δn* = 0.50, *N* > 60	*B-F*, $\hat{\varepsilon }$ = 1, *N* > 20	

G, group effect; T, time effect; G × T, group × time effect; Δn, coefficient of sample size variation; VR, variance ratio; γ_1_, skewness coefficient; γ_2_, kurtosis coefficient; N, total sample size; $\hat{\varepsilon }$*, Greenhouse-Geisser epsilon; F, F-statistic; F-GG, Greenhouse-Geisser adjusted F-test; F-HF, Huynh-Feldt adjusted F-test; B-F, bootstrap-F*.

In an attempt to further simplify the interpretation of the results, Table 7 presents a concise synthesis of the recommendations derived from Tables 3–6. The table is intended as a practical guide for researchers and summarizes the procedures that performed adequately across the non-normal distributions examined, as well as under the different combinations of assumption violations investigated in the present study.

**Table 7:** Practical recommendations for selecting robust tests in a 2 × 3 split-plot design across the non-normal distributions examined and other assumption violations (see Tables 3–6 for details).

Design type	Effect	Condition	Recommended test(s)
Balanced	G	General recommendation under violation of assumptions	*F, B-F*
	T, G × T	Moderate sphericity violation ($\hat{\varepsilon }$ ≥ 0.80) with homogeneity and heterogeneity	*F*
		Moderate sphericity violation ($\hat{\varepsilon }$ ≥ 0.70) with homogeneity and heterogeneity	*F-GG, F-HF*
		Severe sphericity violation ($\hat{\varepsilon }$ = 0.50) with homogeneity and heterogeneity	*F-GG, F-HF* (*N* > 60)
		Severe sphericity violation ($\hat{\varepsilon }\;=$ 0.60) with homogeneity and heterogeneity	*F-GG, F-HF* (*N* > 20)
		General recommendation under violation of assumptions	*B-F* (*N* > 20)
Unbalanced, homogeneous variances	G	General recommendation	*F*, and *B-F* (*N* > 20)
	T, G × T	Moderate sphericity violation ($\hat{\varepsilon }$ ≥ 0.70)	*F, F-GG, F-HF, B-F*
		Severe sphericity violation ($\hat{\varepsilon }$ = 0.50)	*F-GG, F-HF* (*N* > 60)
		Severe sphericity violation ($\hat{\varepsilon }$ = 0.60)	*F-GG, F-HF* (*N* > 20)
		Severe sphericity violation ($\hat{\varepsilon }$ ≤ 0.60)	*B-F* (*N* > 40)
Unbalanced, heterogeneous variances (positive pairing)	G	Small inequality of group size (Δ*n* = 0.16)	*F, B-F*
		Larger inequality of group size (Δ*n* > 0.16)	*B-F*
	T, G × T	Small/moderate inequality of group size (Δ*n* ≤ 0.33), moderate heterogeneity (VR ≤ 2), and moderate sphericity violation ($\hat{\varepsilon }$ = 0.60-0.80)	*F-GG, F-HF* (*N* > 20)
		Small inequality of group size (Δ*n* = 0.16), high heterogeneity (VR = 5), and severe sphericity violation ($\hat{\varepsilon }$ ≤ 0.60)	*F-GG, F-HF* (*N* > 20)
		General recommendation under violation of assumptions	*B-F* (*N* > 20)
Unbalanced, heterogeneous variances (negative pairing)	G	Small inequality of group size (Δ*n* ≤ 0.16) and moderate heterogeneity (VR ≤ 2)	*F* may be acceptable
		Small and moderate inequality of group size (Δ*n* ≤ 0.33) and moderate heterogeneity (VR ≤ 2)	*B-F*
		Larger inequality of group size (Δ*n* = 0.50) and moderate/high heterogeneity (VR ≥ 2)	*B-F* (*N* > 80)
	T, G × T	Small inequality of group size (Δ*n* ≤ 0.16), low heterogeneity (VR = 1.5), and sphericity satisfied ($\hat{\varepsilon }$ ≈ 1)	*F* may be acceptable
		Small inequality of group size (Δ*n* ≤ 0.16), low heterogeneity (VR = 1.5), and moderate sphericity violation ($\hat{\varepsilon }$ ≥ 0.70)	*F-GG or F-HF* may be acceptable
		Small and moderate inequality of group size (Δ*n* ≤ 0.33) and moderate heterogeneity (VR ≤ 2)	*B-F* (*N* > 20)
		Larger inequality of group size (Δ*n* = 0.50) and high heterogeneity (VR ≥ 2)	*B-F* (*N* > 60 - 80)

G, group effect; T, time effect; G × T, group × time effect; Δn, coefficient of sample size variation; VR, variance ratio; N, total sample size; $\hat{\varepsilon }$*, Greenhouse-Geisser epsilon; F, F-statistic; F-GG, Greenhouse-Geisser adjusted F-test; F-HF, Huynh-Feldt adjusted F-test; B-F, bootstrap-F*.

Finally, it should be highlighted that none of the procedures is robust under all conditions of non-sphericity, non-normality, heterogeneity of the variance, and sample size. All the statistics perform worse with severe simultaneous violations of all three assumptions when the group sizes are very unequal. The advice here is to try to plan the research with group sizes that are as similar as possible. In general, the bootstrap method represents an appropriate analytic alternative in most cases of violation of homogeneity of variances and sphericity, although it generally requires a sample size larger than 20 to control the Type I error rate in the majority of conditions. However, in some scenarios with negative pairing, it requires a larger sample (*N* > 80) to achieve robustness if the group sizes are very unequal.

This study has several limitations that should be noted. First, the results are applicable only to the conditions studied here, namely a design with 2 groups and 3 repeated measures, with skewness and kurtosis up to 2.31 and 8, respectively, with varying degrees of homogeneity and sphericity violations. Future studies could extend these results to other designs of higher order and other deviations from the underlying assumptions. Second, the findings are based solely on Type I error rates; future studies should complement these results by examining statistical power, which would provide a more comprehensive understanding of the performance of the procedures examined. Third, this study focused on a specific set of statistical procedures, and other approaches—such as linear mixed models, multivariate analysis of variance, permutation tests, robust statistics based on trimmed means—were not analyzed. Exploring these additional procedures in future research would help better handle repeated-measures designs in situations in which the underlying assumptions are violated.

## Data Availability

The datasets presented in this study can be found in the University of Malaga online repositoriy: https://hdl.handle.net/10630/38406. The datasets can also be found in the article/Supplementary material.
