# Supplementary material for: Guidance for data analysis in split-plot designs when parametric assumptions are violated: F-statistic, adjusted F-tests, and bootstrap-F
Source: Front Psychol. 2026 Aug 6;17:1787528. doi: 10.3389/fpsyg.2026.1787528 (PMC13490978; doi:10.3389/fpsyg.2026.1787528)
Supplement: Supplementary file 1 [file Data_Sheet_1.pdf]

Type I error for the *F-statistic*, Greenhouse-Geisser (*F-GG*) and Huynh-Feldt (*F-HF*) adjustments, and boostrap-*F* (*B-F*) in split-plot design

Authors: M. J. Blanca, R. Alarcón, R. Bono, J. Arnau, F. J. García-Castro, and G. Vallejo

D: Distribution. Shape of the distribution. The numbers indicate the values of skewness (γ_1) and kurtosis (γ_2), respectively. The normal distribution and four non-normal distributions are used: slight (γ_1 = .4, γ_2 = .8), moderate (γ_1 = 1, γ_2 = 1.5), severe (γ_1 = 1.41, γ_2 = 3), and extreme (γ_1 = 2.31, γ_2 = 8) deviation from normality.

VR: Ratio of the covariance matrices between the two groups: a) homogeneity of variance, 1:1; b) heterogeneity of variance, 1:1.5, 1:2, and 1:5. In this scenario, the covariance matrix of the second group could be, respectively, 1.5, 2 or 5 times larger than that of the first group.

P: Pairing of variance with group sample size. The pairings used were: null (0) when there are equal group sizes; positive (+) when the largest group size is associated with the largest value of variance; and negative (-) when the largest group size is associated with the smallest variance.

N: Total sample size: 20, 40, 60, 80, 100, 150 and 200.

Δn: Coefficient of sample size variation (Δn). Represents the amount of inequality in group sizes and was calculated by dividing the standard deviation of the group size by its mean. The values of Δn were 0 (balanced design), 0.16 (low value), 0.33 (medium value) and 0.50 (high value).

n1, n2: Sample size of group 1 and group 2, respectively.

ε: Greenhouse-Geisser epsilon estimation ($\hat{\varepsilon}$). The values were .50, .60, .70, .80, .90 and 1.

F g: Type I error of the *F*-statistic for group effect.

B-F g: Type I error of boostrap-*F* for group effect.

F t: Type I error of the *F*-statistic for time effect.

F-GG t: Type I error of the Greenhouse-Geisser adjustment for time effect.

F-HF t: Type I error of the Huynh-Feldt adjustment for time effect.

B-F t: Type I error of boostrap-*F* for time effect.

F g x t: Type I error of the *F*-statistic for group x time interaction.

F-GG g x t: Type I error of the Greenhouse-Geisser adjustment for group x time interaction.

F-HF g x t: Type I error of the Huynh-Feldt adjustment for group x time interaction.

B-F g x t: Type I error of boostrap-*F* for group x time interaction.

	D	VR	P	N	Δn	n1	n2	ε	F g	B-F g	F t	F-GG t	F-HF t	B-F t	F g x t	F-GG g x t	F-HF g x t	B-F g x t
1	0, 0	1	0	20	0	10	10	.50	4.60	4.28	8.24	4.88	4.90	4.52	8.36	4.52	4.54	4.44
2	0, 0	1	0	20	0.16	8	12	.50	5.12	5.02	8.20	4.86	4.86	4.78	9.22	5.40	5.42	4.90
3	0, 0	1	0	20	0.33	7	13	.50	5.20	5.76	8.60	4.92	4.92	5.60	9.06	5.10	5.12	5.98
4	0, 0	1	0	20	0.50	5	15	.50	5.42	7.32	8.40	5.20	5.22	7.06	8.86	5.38	5.38	7.14
5	0, 0	1	0	40	0	20	20	.50	4.98	4.66	8.20	4.68	4.68	4.68	7.90	4.80	4.80	4.72
6	0, 0	1	0	40	0.16	17	23	.50	4.66	4.82	7.88	4.88	4.88	4.92	7.92	4.54	4.54	4.54
7	0, 0	1	0	40	0.33	13	27	.50	4.66	5.04	8.08	4.70	4.70	5.40	8.10	4.78	4.78	5.12
8	0, 0	1	0	40	0.50	10	30	.50	4.66	5.90	8.40	4.66	4.66	6.22	8.68	4.76	4.76	6.10
9	0, 0	1	0	60	0	30	30	.50	5.06	4.86	8.12	4.84	4.86	4.84	8.26	5.04	5.04	5.02
10	0, 0	1	0	60	0.16	25	35	.50	4.64	4.60	7.92	4.78	4.78	4.76	7.86	4.72	4.72	4.60
11	0, 0	1	0	60	0.33	20	40	.50	4.96	5.12	8.20	4.54	4.54	4.88	8.10	4.90	4.90	5.08
12	0, 0	1	0	60	0.50	15	45	.50	5.14	5.58	8.24	4.36	4.36	5.54	8.36	4.76	4.78	5.44
13	0, 0	1	0	80	0	40	40	.50	5.12	5.02	8.52	4.86	4.88	4.78	8.58	5.42	5.42	5.48
14	0, 0	1	0	80	0.16	34	46	.50	4.78	4.68	8.36	4.96	4.96	4.98	8.24	4.90	4.90	4.90
15	0, 0	1	0	80	0.33	27	53	.50	4.98	5.18	8.24	4.80	4.80	5.08	8.26	5.04	5.04	5.20
16	0, 0	1	0	80	0.50	20	60	.50	4.80	5.52	8.50	4.98	4.98	5.74	8.02	4.90	4.90	5.42
17	0, 0	1	0	100	0	50	50	.50	5.22	5.08	8.42	4.74	4.74	4.86	8.70	5.06	5.06	5.20
18	0, 0	1	0	100	0.16	42	58	.50	5.24	5.20	8.22	4.76	4.76	4.90	8.70	5.08	5.08	5.14
19	0, 0	1	0	100	0.33	33	67	.50	4.88	5.08	8.22	4.80	4.80	5.02	8.20	4.82	4.82	5.02
20	0, 0	1	0	100	0.50	25	75	.50	4.90	5.40	7.72	4.76	4.78	5.26	7.92	4.72	4.72	5.28
21	0, 0	1	0	150	0	75	75	.50	5.40	5.62	7.72	4.56	4.56	4.50	8.76	5.38	5.38	5.46
22	0, 0	1	0	150	0.16	63	87	.50	5.54	5.56	7.94	4.84	4.84	4.76	8.80	5.40	5.40	5.54
23	0, 0	1	0	150	0.33	50	100	.50	5.38	5.68	8.10	4.92	4.92	5.24	8.94	5.38	5.38	5.78
24	0, 0	1	0	150	0.50	37	113	.50	5.06	5.78	8.22	4.66	4.66	5.12	8.46	5.06	5.06	5.44
25	0, 0	1	0	200	0	100	100	.50	5.40	5.46	7.94	4.60	4.60	4.68	8.84	5.30	5.30	5.32
26	0, 0	1	0	200	0.16	84	116	.50	5.28	5.62	8.16	4.68	4.68	4.80	8.78	5.34	5.34	5.52
27	0, 0	1	0	200	0.33	67	133	.50	5.26	5.42	8.14	4.56	4.56	4.74	8.40	5.32	5.32	5.36
28	0, 0	1	0	200	0.50	50	150	.50	5.42	5.76	8.08	4.74	4.74	5.24	8.70	5.28	5.28	5.86
29	0, 0	1.5	0	20	0	10	10	.50	4.68	4.18	8.46	4.98	4.98	4.66	8.38	4.78	4.80	4.38
30	0, 0	1.5	+	20	0.16	8	12	.50	4.54	4.98	7.16	4.04	4.06	4.36	7.86	4.64	4.68	4.90

	D	VR	P	N	Δn	n1	n2	ε	F g	B-F g	F t	F-GG t	F-HF t	B-F t	F g x t	F-GG g x t	F-HF g x t	B-F g x t
31	0, 0	1.5	-	20	0.16	12	8	.50	5.34	4.82	9.90	5.70	5.74	5.02	9.70	5.50	5.54	4.88
32	0, 0	1.5	+	20	0.33	7	13	.50	3.90	5.24	6.58	3.90	3.90	5.10	7.18	4.16	4.16	5.46
33	0, 0	1.5	-	20	0.33	13	7	.50	6.18	5.72	10.74	6.48	6.48	5.64	11.08	6.22	6.22	5.66
34	0, 0	1.5	+	20	0.50	5	15	.50	3.26	6.62	6.14	3.36	3.38	6.38	6.32	3.30	3.30	6.68
35	0, 0	1.5	-	20	0.50	15	5	.50	7.98	8.14	11.88	7.78	7.82	8.02	12.54	7.78	7.80	8.28
36	0, 0	1.5	0	40	0	20	20	.50	4.96	4.70	8.16	4.80	4.80	4.66	8.18	4.66	4.66	4.42
37	0, 0	1.5	+	40	0.16	17	23	.50	3.92	4.42	7.28	4.10	4.10	4.58	7.06	3.94	3.96	4.46
38	0, 0	1.5	-	40	0.16	23	17	.50	5.34	4.76	9.28	5.40	5.40	5.02	9.18	5.30	5.30	4.96
39	0, 0	1.5	+	40	0.33	13	27	.50	3.06	4.84	6.32	3.32	3.32	5.02	6.08	3.26	3.26	5.04
40	0, 0	1.5	-	40	0.33	27	13	.50	5.76	5.16	10.66	6.50	6.50	5.64	10.02	6.02	6.06	5.16
41	0, 0	1.5	+	40	0.50	10	30	.50	2.76	5.70	5.38	2.86	2.86	5.90	5.48	2.60	2.60	5.84
42	0, 0	1.5	-	40	0.50	30	10	.50	7.56	6.28	11.84	7.52	7.52	6.32	11.74	7.32	7.32	6.36
43	0, 0	1.5	0	60	0	30	30	.50	5.00	4.88	8.44	5.00	5.00	4.94	8.30	5.16	5.16	5.04
44	0, 0	1.5	+	60	0.16	25	35	.50	4.10	4.68	7.24	4.08	4.08	4.80	7.04	4.12	4.12	4.68
45	0, 0	1.5	-	60	0.16	35	25	.50	5.76	5.00	9.42	6.02	6.02	5.32	9.58	5.68	5.68	4.90
46	0, 0	1.5	+	60	0.33	20	40	.50	3.92	5.14	6.40	3.38	3.38	4.80	6.32	3.74	3.74	5.20
47	0, 0	1.5	-	60	0.33	40	20	.50	6.92	5.50	10.66	6.64	6.66	5.04	11.30	6.96	6.96	5.62
48	0, 0	1.5	+	60	0.50	15	45	.50	3.06	5.44	5.04	2.92	2.92	5.24	5.62	2.98	2.98	5.20
49	0, 0	1.5	-	60	0.50	45	15	.50	8.20	6.04	12.02	7.72	7.72	5.78	12.30	8.32	8.32	6.08
50	0, 0	1.5	0	80	0	40	40	.50	5.06	4.84	8.70	4.78	4.78	4.68	8.54	5.06	5.06	5.06
51	0, 0	1.5	+	80	0.16	34	46	.50	4.14	5.00	7.56	4.20	4.20	5.00	7.40	4.24	4.24	5.00
52	0, 0	1.5	-	80	0.16	46	34	.50	5.76	5.02	9.34	5.46	5.48	4.90	10.00	6.04	6.04	5.22
53	0, 0	1.5	+	80	0.33	27	53	.50	3.82	5.10	6.14	3.62	3.62	5.04	6.54	3.62	3.62	5.20
54	0, 0	1.5	-	80	0.33	53	27	.50	7.12	5.44	11.08	6.86	6.88	5.42	10.94	7.20	7.20	5.72
55	0, 0	1.5	+	80	0.50	20	60	.50	3.02	5.34	5.38	2.98	2.98	5.56	5.32	2.90	2.90	5.42
56	0, 0	1.5	-	80	0.50	60	20	.50	8.08	6.14	12.62	7.84	7.84	6.02	12.40	8.14	8.14	6.06
57	0, 0	1.5	0	100	0	50	50	.50	5.22	5.38	8.24	4.84	4.84	4.74	8.46	5.10	5.10	5.14
58	0, 0	1.5	+	100	0.16	42	58	.50	4.44	5.10	7.42	4.06	4.08	4.90	7.34	4.40	4.40	5.30
59	0, 0	1.5	-	100	0.16	58	42	.50	6.30	5.62	9.36	6.00	6.00	5.18	10.10	5.98	5.98	5.42
60	0, 0	1.5	+	100	0.33	33	67	.50	3.62	5.02	6.08	3.42	3.42	4.76	6.28	3.42	3.42	4.94
61	0, 0	1.5	-	100	0.33	67	33	.50	7.84	6.28	11.16	6.86	6.86	5.66	12.14	7.70	7.70	6.16
62	0, 0	1.5	+	100	0.50	25	75	.50	2.70	5.04	5.36	3.02	3.02	5.02	5.18	2.92	2.92	4.92

	D	VR	P	N	Δn	n1	n2	ε	F g	B-F g	F t	F-GG t	F-HF t	B-F t	F g x t	F-GG g x t	F-HF g x t	B-F g x t
63	0, 0	1.5	-	100	0.50	75	25	.50	8.08	5.74	12.38	8.12	8.14	5.76	12.50	7.94	7.94	5.72
64	0, 0	1.5	0	150	0	75	75	.50	5.48	5.46	7.74	4.56	4.56	4.52	8.76	5.50	5.50	5.34
65	0, 0	1.5	+	150	0.16	63	87	.50	4.62	5.40	6.72	4.00	4.00	4.70	7.96	4.74	4.74	5.66
66	0, 0	1.5	-	150	0.16	87	63	.50	6.08	5.36	8.80	5.40	5.40	4.68	9.82	6.00	6.00	5.20
67	0, 0	1.5	+	150	0.33	50	100	.50	3.98	5.58	6.10	3.40	3.42	5.16	6.94	3.88	3.88	5.54
68	0, 0	1.5	-	150	0.33	100	50	.50	6.66	5.10	9.96	6.16	6.16	4.46	10.48	6.78	6.78	4.92
69	0, 0	1.5	+	150	0.50	37	113	.50	2.98	5.40	5.32	2.78	2.78	5.26	5.42	3.12	3.12	5.38
70	0, 0	1.5	-	150	0.50	113	37	.50	7.38	5.02	11.04	6.80	6.80	4.74	11.56	7.52	7.52	5.00
71	0, 0	1.5	0	200	0	100	100	.50	5.32	5.36	8.14	4.68	4.68	4.56	8.98	5.42	5.42	5.40
72	0, 0	1.5	+	200	0.16	84	116	.50	4.70	5.58	7.24	3.98	3.98	4.94	8.00	4.62	4.62	5.56
73	0, 0	1.5	-	200	0.16	116	84	.50	5.90	5.36	8.82	5.48	5.48	4.80	9.66	5.98	5.98	5.38
74	0, 0	1.5	+	200	0.33	67	133	.50	3.98	5.10	6.00	3.28	3.28	4.86	6.58	3.90	3.90	5.06
75	0, 0	1.5	-	200	0.33	133	67	.50	6.98	5.50	10.20	6.64	6.64	5.16	11.02	6.90	6.90	5.50
76	0, 0	1.5	+	200	0.50	50	150	.50	3.18	5.52	5.36	2.70	2.70	4.98	5.86	3.30	3.32	5.66
77	0, 0	1.5	-	200	0.50	150	50	.50	7.50	5.50	11.74	8.00	8.00	5.42	11.82	7.74	7.74	5.34
78	0, 0	2	0	20	0	10	10	.50	4.68	4.28	8.72	5.06	5.06	4.64	8.74	4.94	4.94	4.50
79	0, 0	2	+	20	0.16	8	12	.50	4.08	4.96	6.52	3.76	3.76	4.28	7.12	4.14	4.14	4.86
80	0, 0	2	-	20	0.16	12	8	.50	6.32	4.84	11.22	6.52	6.52	5.32	10.32	6.58	6.58	4.98
81	0, 0	2	+	20	0.33	7	13	.50	3.26	5.16	5.78	3.26	3.26	4.88	6.08	3.44	3.48	5.20
82	0, 0	2	-	20	0.33	13	7	.50	7.66	5.92	12.72	7.68	7.68	6.08	12.56	7.72	7.72	5.78
83	0, 0	2	+	20	0.50	5	15	.50	2.36	6.34	4.78	2.36	2.36	6.02	4.80	2.44	2.44	6.30
84	0, 0	2	-	20	0.50	15	5	.50	10.04	8.46	14.92	9.58	9.58	8.28	15.42	9.94	9.96	8.62
85	0, 0	2	0	40	0	20	20	.50	4.86	4.66	8.38	4.92	4.92	4.70	8.12	4.72	4.72	4.32
86	0, 0	2	+	40	0.16	17	23	.50	3.44	4.26	6.80	3.98	3.98	4.56	6.72	3.52	3.52	4.34
87	0, 0	2	-	40	0.16	23	17	.50	5.88	4.80	9.92	6.22	6.22	5.06	9.92	5.88	5.88	4.86
88	0, 0	2	+	40	0.33	13	27	.50	2.54	4.88	5.28	2.72	2.72	4.94	5.04	2.60	2.64	4.82
89	0, 0	2	-	40	0.33	27	13	.50	7.30	5.04	12.52	8.00	8.00	5.76	12.14	7.20	7.22	5.10
90	0, 0	2	+	40	0.50	10	30	.50	1.92	5.26	4.06	2.02	2.02	5.56	3.88	1.92	1.92	5.50
91	0, 0	2	-	40	0.50	30	10	.50	9.42	6.36	14.76	9.68	9.68	6.28	14.78	9.48	9.48	6.46
92	0, 0	2	0	60	0	30	30	.50	5.08	4.72	8.22	5.10	5.10	4.92	8.30	4.94	4.94	4.84
93	0, 0	2	+	60	0.16	25	35	.50	3.84	4.76	6.72	3.78	3.78	4.76	6.50	3.80	3.82	4.88
94	0, 0	2	-	60	0.16	35	25	.50	6.48	5.08	10.36	6.62	6.62	5.38	10.18	6.30	6.30	5.04

	D	VR	P	N	Δn	n1	n2	ε	F g	B-F g	F t	F-GG t	F-HF t	B-F t	F g x t	F-GG g x t	F-HF g x t	B-F g x t
95	0, 0	2	+	60	0.33	20	40	.50	3.06	4.98	5.42	2.56	2.56	4.88	5.30	2.94	2.94	5.30
96	0, 0	2	-	60	0.33	40	20	.50	8.16	5.68	12.44	8.02	8.04	4.88	12.48	8.28	8.28	5.60
97	0, 0	2	+	60	0.50	15	45	.50	2.12	5.28	3.72	2.16	2.16	5.22	4.16	2.02	2.02	5.14
98	0, 0	2	-	60	0.50	45	15	.50	10.50	6.14	14.72	10.08	10.08	5.86	15.04	10.30	10.30	6.14
99	0, 0	2	0	80	0	40	40	.50	4.94	4.92	8.54	4.74	4.74	4.70	8.36	5.08	5.08	5.02
100	0, 0	2	+	80	0.16	34	46	.50	3.86	4.92	7.14	3.86	3.86	5.02	6.94	3.98	3.98	5.10
101	0, 0	2	-	80	0.16	46	34	.50	6.46	5.18	10.10	6.10	6.10	4.92	10.54	6.64	6.66	5.16
102	0, 0	2	+	80	0.33	27	53	.50	2.96	5.04	5.16	2.90	2.90	4.88	5.44	2.94	2.94	5.14
103	0, 0	2	-	80	0.33	53	27	.50	8.22	5.50	12.78	8.20	8.20	5.36	12.62	8.36	8.36	5.72
104	0, 0	2	+	80	0.50	20	60	.50	2.04	4.98	4.02	2.16	2.16	5.30	3.82	1.82	1.82	5.18
105	0, 0	2	-	80	0.50	60	20	.50	10.20	6.20	15.66	10.32	10.32	6.16	15.14	10.28	10.28	6.24
106	0, 0	2	0	100	0	50	50	.50	5.04	5.18	8.20	4.98	4.98	4.74	8.58	5.08	5.08	5.02
107	0, 0	2	+	100	0.16	42	58	.50	3.90	5.18	6.84	3.64	3.64	4.90	6.84	3.96	3.96	5.04
108	0, 0	2	-	100	0.16	58	42	.50	6.74	5.76	10.08	6.58	6.58	5.18	10.76	6.48	6.50	5.24
109	0, 0	2	+	100	0.33	33	67	.50	2.82	5.02	5.06	2.60	2.60	4.78	5.20	2.66	2.66	4.94
110	0, 0	2	-	100	0.33	67	33	.50	9.10	6.12	12.78	8.14	8.14	5.68	13.42	9.28	9.28	6.08
111	0, 0	2	+	100	0.50	25	75	.50	1.82	4.84	3.96	2.08	2.08	5.00	3.76	1.84	1.84	5.00
112	0, 0	2	-	100	0.50	75	25	.50	10.50	5.94	15.00	10.18	10.20	5.96	15.28	10.44	10.44	5.66
113	0, 0	2	0	150	0	75	75	.50	5.48	5.38	7.72	4.54	4.54	4.62	8.86	5.30	5.30	5.36
114	0, 0	2	+	150	0.16	63	87	.50	4.10	5.60	6.12	3.58	3.58	4.62	7.26	4.20	4.20	5.60
115	0, 0	2	-	150	0.16	87	63	.50	6.62	5.30	9.68	5.80	5.80	4.50	10.44	6.60	6.60	5.18
116	0, 0	2	+	150	0.33	50	100	.50	3.04	5.52	5.00	2.56	2.56	5.02	5.60	3.04	3.04	5.40
117	0, 0	2	-	150	0.33	100	50	.50	7.96	5.12	11.40	7.42	7.42	4.70	11.86	8.06	8.06	5.12
118	0, 0	2	+	150	0.50	37	113	.50	2.06	5.16	3.86	1.88	1.88	5.20	4.14	2.02	2.02	5.28
119	0, 0	2	-	150	0.50	113	37	.50	9.44	5.02	13.82	8.84	8.84	4.72	14.28	9.40	9.42	5.22
120	0, 0	2	0	200	0	100	100	.50	5.38	5.40	8.12	4.68	4.68	4.80	8.92	5.56	5.56	5.54
121	0, 0	2	+	200	0.16	84	116	.50	4.30	5.62	6.64	3.64	3.64	4.80	7.10	4.18	4.18	5.42
122	0, 0	2	-	200	0.16	116	84	.50	6.60	5.26	9.58	5.86	5.86	5.06	10.40	6.70	6.70	5.36
123	0, 0	2	+	200	0.33	67	133	.50	3.00	5.16	5.10	2.60	2.60	5.08	5.34	3.18	3.18	5.16
124	0, 0	2	-	200	0.33	133	67	.50	8.54	5.48	11.78	7.90	7.90	5.14	12.32	8.44	8.44	5.44
125	0, 0	2	+	200	0.50	50	150	.50	2.24	5.50	3.86	1.92	1.92	5.10	4.18	2.36	2.36	5.74
126	0, 0	2	-	200	0.50	150	50	.50	9.90	5.42	14.38	10.00	10.00	5.38	15.00	9.86	9.86	5.38

	D	VR	P	N	Δn	n1	n2	ε	F g	B-F g	F t	F-GG t	F-HF t	B-F t	F g x t	F-GG g x t	F-HF g x t	B-F g x t
127	0, 0	5	0	20	0	10	10	.50	5.56	4.52	9.82	5.86	5.88	4.68	9.18	5.66	5.68	4.60
128	0, 0	5	+	20	0.16	8	12	.50	3.10	4.68	5.62	3.02	3.02	4.58	5.82	2.94	2.96	4.56
129	0, 0	5	-	20	0.16	12	8	.50	8.86	5.32	14.80	9.62	9.64	5.42	13.58	8.84	8.84	5.36
130	0, 0	5	+	20	0.33	7	13	.50	2.16	4.12	4.06	1.94	1.94	3.76	4.32	2.18	2.18	4.28
131	0, 0	5	-	20	0.33	13	7	.50	11.72	5.88	17.60	11.94	11.98	6.12	17.22	12.04	12.06	5.92
132	0, 0	5	+	20	0.50	5	15	.50	.94	5.02	1.96	.72	.72	4.82	2.06	.98	.98	5.00
133	0, 0	5	-	20	0.50	15	5	.50	18.02	8.30	24.02	17.44	17.44	8.50	24.08	18.26	18.26	8.56
134	0, 0	5	0	40	0	20	20	.50	5.06	4.78	8.84	5.38	5.38	5.14	8.56	5.12	5.12	4.66
135	0, 0	5	+	40	0.16	17	23	.50	2.98	4.88	5.74	3.22	3.22	4.82	5.66	3.00	3.00	4.76
136	0, 0	5	-	40	0.16	23	17	.50	7.82	4.72	12.16	8.04	8.04	5.24	12.42	7.58	7.58	4.86
137	0, 0	5	+	40	0.33	13	27	.50	1.46	4.56	3.02	1.42	1.44	4.84	2.92	1.60	1.60	4.68
138	0, 0	5	-	40	0.33	27	13	.50	12.02	5.28	18.38	12.78	12.80	6.14	18.10	12.10	12.12	5.02
139	0, 0	5	+	40	0.50	10	30	.50	.70	5.10	1.58	.92	.92	4.98	1.62	.70	.70	5.26
140	0, 0	5	-	40	0.50	30	10	.50	16.86	6.42	23.10	17.12	17.12	6.32	22.54	16.72	16.72	6.10
141	0, 0	5	0	60	0	30	30	.50	4.92	4.38	8.54	5.36	5.36	4.90	8.38	5.06	5.06	4.58
142	0, 0	5	+	60	0.16	25	35	.50	2.92	4.66	5.50	3.36	3.36	4.74	5.46	2.90	2.90	4.76
143	0, 0	5	-	60	0.16	35	25	.50	7.84	5.08	12.52	8.22	8.22	4.78	12.04	7.94	7.94	5.10
144	0, 0	5	+	60	0.33	20	40	.50	1.76	4.62	3.20	1.64	1.64	4.88	3.04	1.62	1.62	4.76
145	0, 0	5	-	60	0.33	40	20	.50	12.36	5.08	17.10	12.36	12.36	4.88	17.90	12.44	12.44	5.24
146	0, 0	5	+	60	0.50	15	45	.50	.66	5.06	1.60	.78	.78	4.94	1.70	.70	.70	4.72
147	0, 0	5	-	60	0.50	45	15	.50	16.90	5.90	22.68	16.92	16.92	5.64	22.70	17.20	17.20	6.18
148	0, 0	5	0	80	0	40	40	.50	4.98	4.70	8.20	4.62	4.62	4.42	8.70	5.02	5.02	4.74
149	0, 0	5	+	80	0.16	34	46	.50	2.74	4.80	5.56	3.08	3.08	4.84	5.58	2.86	2.86	5.00
150	0, 0	5	-	80	0.16	46	34	.50	7.98	4.82	11.92	7.90	7.90	4.84	12.78	8.06	8.06	5.08
151	0, 0	5	+	80	0.33	27	53	.50	1.32	4.58	3.06	1.62	1.62	4.84	3.12	1.34	1.34	4.68
152	0, 0	5	-	80	0.33	53	27	.50	12.14	5.54	17.82	12.20	12.22	5.26	17.04	12.28	12.28	5.44
153	0, 0	5	+	80	0.50	20	60	.50	.54	4.60	1.62	.74	.74	4.82	1.18	.46	.46	4.64
154	0, 0	5	-	80	0.50	60	20	.50	17.50	6.20	23.54	18.18	18.18	5.74	23.54	17.72	17.72	6.26
155	0, 0	5	0	100	0	50	50	.50	5.28	5.02	8.34	5.10	5.10	4.90	8.34	5.12	5.14	5.08
156	0, 0	5	+	100	0.16	42	58	.50	3.00	4.90	5.68	2.96	3.00	4.96	5.48	3.08	3.08	5.04
157	0, 0	5	-	100	0.16	58	42	.50	8.54	5.42	12.10	7.92	7.92	5.04	12.98	8.12	8.12	4.94
158	0, 0	5	+	100	0.33	33	67	.50	.98	4.90	3.02	1.12	1.12	4.62	2.68	1.02	1.02	4.70

	D	VR	P	N	Δn	n1	n2	ε	F g	B-F g	F t	F-GG t	F-HF t	B-F t	F g x t	F-GG g x t	F-HF g x t	B-F g x t
159	0, 0	5	-	100	0.33	67	33	.50	13.36	6.06	17.98	12.60	12.62	5.48	18.08	13.28	13.30	5.86
160	0, 0	5	+	100	0.50	25	75	.50	.48	4.38	1.48	.52	.52	4.86	1.30	.44	.44	4.50
161	0, 0	5	-	100	0.50	75	25	.50	18.28	5.80	23.06	17.28	17.28	5.80	24.16	17.96	17.96	5.66
162	0, 0	5	0	150	0	75	75	.50	5.44	5.42	8.14	4.90	4.92	4.70	8.96	5.48	5.48	5.32
163	0, 0	5	+	150	0.16	63	87	.50	2.86	5.28	4.86	2.70	2.70	4.52	6.06	2.84	2.84	5.46
164	0, 0	5	-	150	0.16	87	63	.50	8.10	5.04	11.88	7.80	7.80	4.88	12.78	8.10	8.12	5.22
165	0, 0	5	+	150	0.33	50	100	.50	1.38	5.04	2.64	1.16	1.16	4.54	2.96	1.40	1.40	4.98
166	0, 0	5	-	150	0.33	100	50	.50	11.72	4.88	15.94	11.10	11.10	4.74	16.28	11.58	11.58	5.08
167	0, 0	5	+	150	0.50	37	113	.50	.48	5.02	1.36	.56	.56	4.76	1.40	.56	.56	4.96
168	0, 0	5	-	150	0.50	113	37	.50	16.92	4.90	22.12	16.20	16.20	4.52	22.58	17.08	17.08	5.02
169	0, 0	5	0	200	0	100	100	.50	5.64	5.28	8.02	4.96	4.96	4.84	8.90	5.50	5.50	5.34
170	0, 0	5	+	200	0.16	84	116	.50	3.16	5.38	5.28	2.84	2.84	4.96	5.90	3.22	3.22	5.42
171	0, 0	5	-	200	0.16	116	84	.50	8.08	5.74	11.88	7.84	7.84	5.14	11.80	8.08	8.08	5.58
172	0, 0	5	+	200	0.33	67	133	.50	1.74	5.08	2.94	1.30	1.30	4.74	3.32	1.62	1.62	5.14
173	0, 0	5	-	200	0.33	133	67	.50	12.26	5.64	16.96	11.92	11.92	5.10	16.76	12.06	12.06	5.50
174	0, 0	5	+	200	0.50	50	150	.50	.62	5.24	1.28	.46	.46	4.82	1.56	.52	.52	5.28
175	0, 0	5	-	200	0.50	150	50	.50	17.40	5.42	22.04	17.18	17.18	5.28	22.90	17.38	17.38	5.28
176	0, 0	1	0	20	0	10	10	.60	4.16	3.86	7.72	5.20	5.30	5.02	8.02	5.12	5.26	4.88
177	0, 0	1	0	20	0.16	8	12	.60	4.74	4.36	7.74	5.20	5.28	5.10	7.50	5.02	5.24	5.12
178	0, 0	1	0	20	0.33	7	13	.60	5.14	5.80	7.68	5.34	5.52	5.54	7.82	4.94	5.12	5.66
179	0, 0	1	0	20	0.50	5	15	.60	4.88	7.14	7.88	4.86	5.02	7.62	7.58	4.98	5.24	7.40
180	0, 0	1	0	40	0	20	20	.60	5.00	4.86	7.16	4.92	4.98	4.64	7.78	4.88	5.00	4.90
181	0, 0	1	0	40	0.16	17	23	.60	5.00	5.20	7.24	4.74	4.82	4.86	7.14	4.64	4.72	4.86
182	0, 0	1	0	40	0.33	13	27	.60	4.32	4.98	7.38	4.74	4.76	5.30	7.88	5.20	5.26	5.50
183	0, 0	1	0	40	0.50	10	30	.60	4.34	6.02	6.86	4.58	4.58	6.10	7.74	5.04	5.10	6.74
184	0, 0	1	0	60	0	30	30	.60	5.04	5.10	7.12	4.74	4.78	4.86	7.10	4.58	4.62	4.80
185	0, 0	1	0	60	0.16	25	35	.60	5.12	5.02	7.26	4.84	4.94	5.10	7.64	5.28	5.30	5.40
186	0, 0	1	0	60	0.33	20	40	.60	5.22	5.86	7.30	4.64	4.72	5.22	7.42	4.94	4.98	5.34
187	0, 0	1	0	60	0.50	15	45	.60	4.96	6.02	7.36	4.82	4.86	5.56	7.30	4.86	4.90	5.66
188	0, 0	1	0	80	0	40	40	.60	5.20	5.26	7.92	5.56	5.58	5.48	6.62	4.58	4.62	4.50
189	0, 0	1	0	80	0.16	34	46	.60	5.04	5.32	7.86	5.22	5.28	5.36	7.36	5.00	5.02	5.14
190	0, 0	1	0	80	0.33	27	53	.60	5.12	5.36	7.42	5.04	5.04	5.30	6.92	4.70	4.74	5.16

	D	VR	P	N	Δn	n1	n2	ε	F g	B-F g	F t	F-GG t	F-HF t	B-F t	F g x t	F-GG g x t	F-HF g x t	B-F g x t
191	0, 0	1	0	80	0.50	20	60	.60	5.32	6.12	7.44	4.94	4.96	5.72	7.20	4.98	5.06	5.50
192	0, 0	1	0	100	0	50	50	.60	5.24	5.28	7.66	4.84	4.92	5.16	7.24	4.86	4.86	5.00
193	0, 0	1	0	100	0.16	42	58	.60	5.18	5.12	7.56	5.06	5.06	5.32	6.76	4.30	4.30	4.52
194	0, 0	1	0	100	0.33	33	67	.60	4.88	5.46	7.48	5.00	5.02	5.46	7.02	4.80	4.80	5.16
195	0, 0	1	0	100	0.50	25	75	.60	5.22	5.50	7.30	4.78	4.80	5.34	7.28	4.66	4.68	5.20
196	0, 0	1	0	150	0	75	75	.60	5.52	5.52	8.38	5.60	5.62	5.54	7.74	5.14	5.14	5.34
197	0, 0	1	0	150	0.16	63	87	.60	5.18	5.28	8.16	5.58	5.58	5.92	6.98	4.68	4.78	4.88
198	0, 0	1	0	150	0.33	50	100	.60	5.36	5.56	8.10	5.42	5.46	5.72	7.22	5.06	5.06	5.16
199	0, 0	1	0	150	0.50	37	113	.60	5.30	6.00	7.60	5.18	5.18	5.48	7.14	4.66	4.66	5.08
200	0, 0	1	0	200	0	100	100	.60	4.92	5.06	7.70	5.26	5.28	5.36	7.32	5.26	5.28	5.30
201	0, 0	1	0	200	0.16	84	116	.60	5.24	5.06	7.78	5.44	5.44	5.14	7.56	5.38	5.38	5.44
202	0, 0	1	0	200	0.33	67	133	.60	5.16	5.26	7.92	5.36	5.36	5.42	7.12	5.10	5.10	4.98
203	0, 0	1	0	200	0.50	50	150	.60	5.14	5.38	7.98	5.20	5.24	5.72	7.16	4.76	4.78	4.98
204	0, 0	1.5	0	20	0	10	10	.60	4.36	3.90	7.76	5.06	5.24	5.00	7.96	5.26	5.38	4.98
205	0, 0	1.5	+	20	0.16	8	12	.60	3.94	4.50	6.76	4.54	4.66	4.98	6.48	4.08	4.32	4.70
206	0, 0	1.5	-	20	0.16	12	8	.60	5.76	4.82	8.82	6.00	6.24	5.60	9.52	6.54	6.78	5.58
207	0, 0	1.5	+	20	0.33	7	13	.60	4.08	5.32	6.12	4.02	4.28	5.44	6.18	3.64	3.80	5.36
208	0, 0	1.5	-	20	0.33	13	7	.60	6.38	5.52	9.56	6.66	6.80	5.96	10.38	7.12	7.34	6.26
209	0, 0	1.5	+	20	0.50	5	15	.60	3.12	6.16	5.14	3.14	3.28	6.94	5.06	2.96	3.20	6.92
210	0, 0	1.5	-	20	0.50	15	5	.60	7.70	8.16	10.78	7.82	8.02	8.16	11.08	7.90	8.16	7.82
211	0, 0	1.5	0	40	0	20	20	.60	5.04	4.94	7.40	4.86	4.96	4.78	7.66	5.20	5.24	5.00
212	0, 0	1.5	+	40	0.16	17	23	.60	4.52	5.02	6.38	4.18	4.28	4.58	6.44	3.98	4.06	4.72
213	0, 0	1.5	-	40	0.16	23	17	.60	5.58	5.22	8.72	5.66	5.70	5.10	8.26	5.82	5.86	5.10
214	0, 0	1.5	+	40	0.33	13	27	.60	3.06	4.80	5.12	3.34	3.38	5.18	5.92	3.62	3.68	5.20
215	0, 0	1.5	-	40	0.33	27	13	.60	6.88	5.76	9.66	6.84	6.88	5.78	9.12	6.46	6.56	5.26
216	0, 0	1.5	+	40	0.50	10	30	.60	2.58	5.44	4.40	2.74	2.80	5.54	5.08	3.00	3.06	6.24
217	0, 0	1.5	-	40	0.50	30	10	.60	7.42	6.66	10.56	7.20	7.28	6.44	10.58	7.20	7.32	6.46
218	0, 0	1.5	0	60	0	30	30	.60	5.08	4.72	7.56	4.94	4.98	4.94	6.98	4.72	4.76	4.56
219	0, 0	1.5	+	60	0.16	25	35	.60	4.32	4.98	6.30	4.08	4.08	4.72	6.58	4.46	4.52	5.14
220	0, 0	1.5	-	60	0.16	35	25	.60	5.40	4.58	8.80	6.00	6.02	5.22	8.32	5.58	5.64	4.68
221	0, 0	1.5	+	60	0.33	20	40	.60	3.76	5.42	5.66	3.22	3.28	4.90	5.70	3.68	3.72	5.20
222	0, 0	1.5	-	60	0.33	40	20	.60	6.32	5.02	9.76	6.90	6.96	5.62	9.34	6.50	6.54	5.00

	D	VR	P	N	Δn	n1	n2	ε	F g	B-F g	F t	F-GG t	F-HF t	B-F t	F g x t	F-GG g x t	F-HF g x t	B-F g x t
223	0, 0	1.5	+	60	0.50	15	45	.60	3.16	5.70	4.56	2.72	2.72	5.28	4.72	2.70	2.72	5.38
224	0, 0	1.5	-	60	0.50	45	15	.60	7.40	5.50	11.06	8.08	8.14	6.04	10.28	7.48	7.52	5.78
225	0, 0	1.5	0	80	0	40	40	.60	5.12	5.12	8.06	5.50	5.52	5.46	6.72	4.60	4.64	4.70
226	0, 0	1.5	+	80	0.16	34	46	.60	4.28	4.96	6.94	4.76	4.78	5.40	6.86	4.70	4.72	5.44
227	0, 0	1.5	-	80	0.16	46	34	.60	5.64	5.04	9.28	6.34	6.34	5.62	8.30	5.04	5.08	4.50
228	0, 0	1.5	+	80	0.33	27	53	.60	3.50	5.04	5.78	3.72	3.78	5.40	5.34	3.22	3.26	4.96
229	0, 0	1.5	-	80	0.33	53	27	.60	6.42	5.06	10.36	7.26	7.30	5.74	9.34	6.40	6.46	4.90
230	0, 0	1.5	+	80	0.50	20	60	.60	3.06	5.96	4.94	3.20	3.20	5.84	5.06	3.14	3.18	5.46
231	0, 0	1.5	-	80	0.50	60	20	.60	7.30	5.54	11.42	7.92	7.92	5.98	10.86	7.78	7.82	5.88
232	0, 0	1.5	0	100	0	50	50	.60	5.14	5.30	7.44	4.92	4.96	5.08	7.34	5.00	5.02	5.16
233	0, 0	1.5	+	100	0.16	42	58	.60	4.38	5.22	6.56	4.22	4.22	5.12	5.92	3.84	3.88	4.52
234	0, 0	1.5	-	100	0.16	58	42	.60	6.08	5.32	8.60	5.68	5.68	4.78	8.80	5.84	5.84	4.80
235	0, 0	1.5	+	100	0.33	33	67	.60	3.66	5.32	5.58	3.50	3.50	5.06	5.26	3.58	3.58	4.94
236	0, 0	1.5	-	100	0.33	67	33	.60	6.88	5.68	9.62	6.64	6.64	5.16	9.40	6.42	6.44	5.28
237	0, 0	1.5	+	100	0.50	25	75	.60	2.92	5.22	4.48	2.76	2.78	5.18	4.60	2.70	2.76	5.10
238	0, 0	1.5	-	100	0.50	75	25	.60	7.82	5.64	9.94	6.98	7.00	5.22	10.84	7.84	7.86	5.46
239	0, 0	1.5	0	150	0	75	75	.60	5.78	5.72	8.40	5.72	5.72	5.54	7.86	5.06	5.10	5.20
240	0, 0	1.5	+	150	0.16	63	87	.60	4.64	5.24	7.26	4.88	4.90	5.74	6.24	4.08	4.10	5.06
241	0, 0	1.5	-	150	0.16	87	63	.60	5.68	4.94	9.22	6.26	6.28	5.58	8.88	6.02	6.02	5.20
242	0, 0	1.5	+	150	0.33	50	100	.60	3.82	5.00	6.22	3.92	3.92	5.84	5.68	3.20	3.26	5.08
243	0, 0	1.5	-	150	0.33	100	50	.60	6.16	4.80	9.98	7.06	7.08	5.70	9.20	6.46	6.48	4.94
244	0, 0	1.5	+	150	0.50	37	113	.60	3.10	5.64	5.22	3.04	3.06	5.48	4.70	2.90	2.90	5.08
245	0, 0	1.5	-	150	0.50	113	37	.60	7.68	5.38	10.74	7.78	7.78	5.30	10.16	6.98	7.00	5.34
246	0, 0	1.5	0	200	0	100	100	.60	4.88	4.92	7.60	5.16	5.20	5.24	7.32	5.18	5.18	5.20
247	0, 0	1.5	+	200	0.16	84	116	.60	4.56	5.08	6.90	4.56	4.56	5.36	6.86	4.22	4.26	5.18
248	0, 0	1.5	-	200	0.16	116	84	.60	6.02	5.02	8.98	5.80	5.84	4.98	8.46	6.10	6.10	5.08
249	0, 0	1.5	+	200	0.33	67	133	.60	3.64	5.18	5.96	4.04	4.04	5.38	5.50	3.62	3.62	4.90
250	0, 0	1.5	-	200	0.33	133	67	.60	6.64	4.94	10.22	7.16	7.20	5.12	10.50	6.94	6.96	5.24
251	0, 0	1.5	+	200	0.50	50	150	.60	3.20	5.18	5.16	3.00	3.02	5.82	4.48	2.46	2.46	5.02
252	0, 0	1.5	-	200	0.50	150	50	.60	7.92	5.32	11.72	8.18	8.18	5.58	11.76	8.02	8.06	5.46
253	0, 0	2	0	20	0	10	10	.60	4.50	4.14	7.84	4.98	5.28	4.82	8.08	5.26	5.44	4.96
254	0, 0	2	+	20	0.16	8	12	.60	3.70	4.38	5.98	4.12	4.26	5.10	5.92	3.72	3.74	4.72

	D	VR	P	N	Δn	n1	n2	ε	F g	B-F g	F t	F-GG t	F-HF t	B-F t	F g x t	F-GG g x t	F-HF g x t	B-F g x t
255	0, 0	2	-	20	0.16	12	8	.60	6.42	5.06	9.90	6.86	7.06	5.66	10.66	7.36	7.54	5.68
256	0, 0	2	+	20	0.33	7	13	.60	3.48	4.98	5.22	3.46	3.62	5.24	5.08	3.08	3.34	5.32
257	0, 0	2	-	20	0.33	13	7	.60	7.54	5.90	11.10	8.16	8.28	5.86	12.00	8.56	8.76	6.22
258	0, 0	2	+	20	0.50	5	15	.60	2.32	5.82	3.84	2.26	2.36	6.34	3.70	2.06	2.24	6.16
259	0, 0	2	-	20	0.50	15	5	.60	9.78	8.48	13.96	9.88	10.28	8.46	13.44	10.16	10.36	7.98
260	0, 0	2	0	40	0	20	20	.60	5.08	4.80	7.60	4.96	5.04	4.64	7.56	5.26	5.32	5.10
261	0, 0	2	+	40	0.16	17	23	.60	4.40	4.94	5.98	3.86	3.94	4.64	5.88	3.54	3.60	4.44
262	0, 0	2	-	40	0.16	23	17	.60	6.04	5.36	9.24	6.52	6.70	5.38	8.74	6.26	6.32	4.98
263	0, 0	2	+	40	0.33	13	27	.60	2.48	4.56	4.06	2.66	2.72	4.98	4.68	2.82	2.84	5.06
264	0, 0	2	-	40	0.33	27	13	.60	8.18	5.88	11.52	8.08	8.16	5.86	11.00	7.74	7.88	5.58
265	0, 0	2	+	40	0.50	10	30	.60	1.58	5.12	3.14	1.86	1.88	5.44	3.38	2.10	2.12	5.86
266	0, 0	2	-	40	0.50	30	10	.60	9.46	6.58	13.24	9.72	9.76	6.54	13.44	9.50	9.66	6.70
267	0, 0	2	0	60	0	30	30	.60	4.90	4.74	7.62	4.96	4.98	4.94	7.00	4.66	4.66	4.32
268	0, 0	2	+	60	0.16	25	35	.60	3.86	4.78	5.64	3.86	3.88	4.70	6.08	3.80	3.86	5.02
269	0, 0	2	-	60	0.16	35	25	.60	5.88	4.48	9.68	6.74	6.78	5.06	9.34	6.44	6.44	4.90
270	0, 0	2	+	60	0.33	20	40	.60	2.76	5.22	4.54	2.50	2.50	4.86	4.78	3.04	3.12	5.16
271	0, 0	2	-	60	0.33	40	20	.60	7.66	5.06	11.70	8.24	8.28	5.48	11.14	7.94	7.98	4.86
272	0, 0	2	+	60	0.50	15	45	.60	2.04	5.64	3.20	1.74	1.88	4.96	3.24	1.92	1.92	5.22
273	0, 0	2	-	60	0.50	45	15	.60	9.36	5.46	13.50	10.18	10.22	5.90	12.84	9.30	9.38	6.06
274	0, 0	2	0	80	0	40	40	.60	5.00	4.96	8.20	5.62	5.62	5.46	6.88	4.72	4.74	4.82
275	0, 0	2	+	80	0.16	34	46	.60	3.94	4.80	6.56	4.24	4.30	5.48	6.34	4.52	4.54	5.66
276	0, 0	2	-	80	0.16	46	34	.60	6.12	4.84	10.00	6.94	6.98	5.58	9.34	5.78	5.80	4.70
277	0, 0	2	+	80	0.33	27	53	.60	2.90	4.74	4.88	2.92	2.92	5.46	4.36	2.66	2.66	4.82
278	0, 0	2	-	80	0.33	53	27	.60	7.38	4.90	11.86	8.62	8.66	5.48	11.24	7.72	7.74	4.92
279	0, 0	2	+	80	0.50	20	60	.60	2.16	5.84	3.52	2.04	2.10	5.80	3.62	2.10	2.12	5.30
280	0, 0	2	-	80	0.50	60	20	.60	9.28	5.42	13.92	10.48	10.52	6.12	13.56	10.06	10.12	5.76
281	0, 0	2	0	100	0	50	50	.60	5.28	5.16	7.42	4.88	4.90	4.84	7.40	5.06	5.10	4.96
282	0, 0	2	+	100	0.16	42	58	.60	4.02	5.22	6.14	3.84	3.88	5.24	5.50	3.42	3.46	4.68
283	0, 0	2	-	100	0.16	58	42	.60	6.64	5.24	9.32	6.28	6.28	5.02	9.48	6.34	6.40	4.66
284	0, 0	2	+	100	0.33	33	67	.60	2.78	5.24	4.46	2.78	2.78	5.30	4.54	2.92	2.94	4.80
285	0, 0	2	-	100	0.33	67	33	.60	8.18	5.28	11.16	7.88	7.88	4.86	11.38	7.76	7.80	4.86
286	0, 0	2	+	100	0.50	25	75	.60	2.06	5.12	3.08	1.84	1.84	5.32	3.28	1.76	1.76	5.04

	D	VR	P	N	Δn	n1	n2	ε	F g	B-F g	F t	F-GG t	F-HF t	B-F t	F g x t	F-GG g x t	F-HF g x t	B-F g x t
287	0, 0	2	-	100	0.50	75	25	.60	10.16	5.72	12.48	8.86	8.92	4.98	13.46	10.08	10.08	5.18
288	0, 0	2	0	150	0	75	75	.60	5.84	5.70	8.46	5.64	5.64	5.48	7.98	5.24	5.24	5.24
289	0, 0	2	+	150	0.16	63	87	.60	4.12	5.22	6.72	4.48	4.48	5.74	5.68	3.70	3.70	5.06
290	0, 0	2	-	150	0.16	87	63	.60	6.22	4.66	9.94	6.74	6.82	5.28	9.84	6.68	6.70	5.22
291	0, 0	2	+	150	0.33	50	100	.60	3.08	5.00	5.16	2.98	2.98	5.92	4.74	2.54	2.54	5.24
292	0, 0	2	-	150	0.33	100	50	.60	7.38	4.68	11.56	8.26	8.28	5.76	10.72	7.70	7.74	5.10
293	0, 0	2	+	150	0.50	37	113	.60	1.92	5.52	3.66	1.94	1.94	5.60	3.36	2.08	2.08	5.02
294	0, 0	2	-	150	0.50	113	37	.60	9.72	5.38	13.94	9.78	9.78	5.46	12.70	9.22	9.22	5.38
295	0, 0	2	0	200	0	100	100	.60	4.88	4.80	7.74	5.00	5.00	5.16	7.44	4.94	4.94	4.90
296	0, 0	2	+	200	0.16	84	116	.60	4.06	5.10	6.22	4.22	4.22	5.40	5.98	3.90	3.92	5.10
297	0, 0	2	-	200	0.16	116	84	.60	6.44	4.84	9.66	6.66	6.66	5.10	9.16	6.58	6.58	5.14
298	0, 0	2	+	200	0.33	67	133	.60	2.80	5.10	5.06	3.08	3.08	5.40	4.42	2.76	2.76	5.16
299	0, 0	2	-	200	0.33	133	67	.60	8.32	5.10	11.90	8.64	8.68	5.36	12.16	8.44	8.46	5.32
300	0, 0	2	+	200	0.50	50	150	.60	2.02	5.10	3.54	2.14	2.14	5.90	2.98	1.88	1.88	5.08
301	0, 0	2	-	200	0.50	150	50	.60	10.12	5.44	14.32	10.74	10.76	5.54	14.24	10.76	10.80	5.60
302	0, 0	5	0	20	0	10	10	.60	5.32	4.64	8.28	5.84	5.92	5.26	8.60	5.72	5.84	5.00
303	0, 0	5	+	20	0.16	8	12	.60	3.30	4.20	4.66	3.14	3.20	4.68	4.38	2.60	2.72	4.26
304	0, 0	5	-	20	0.16	12	8	.60	9.22	5.42	13.14	9.90	10.26	5.68	13.26	9.68	9.84	5.72
305	0, 0	5	+	20	0.33	7	13	.60	2.38	4.42	3.46	2.04	2.20	4.18	3.42	2.04	2.20	4.28
306	0, 0	5	-	20	0.33	13	7	.60	11.94	6.24	15.60	11.98	12.32	6.10	16.54	12.46	12.78	5.96
307	0, 0	5	+	20	0.50	5	15	.60	.92	5.40	1.48	.74	.84	5.54	1.32	.72	.74	5.24
308	0, 0	5	-	20	0.50	15	5	.60	18.12	8.62	23.56	18.62	18.88	8.16	23.66	18.24	18.46	7.80
309	0, 0	5	0	40	0	20	20	.60	4.98	4.72	7.98	5.16	5.22	4.96	7.86	5.38	5.52	5.18
310	0, 0	5	+	40	0.16	17	23	.60	3.34	4.98	4.86	3.04	3.12	4.80	4.86	3.00	3.08	4.68
311	0, 0	5	-	40	0.16	23	17	.60	8.02	5.26	11.30	8.08	8.12	5.68	10.68	7.44	7.56	5.12
312	0, 0	5	+	40	0.33	13	27	.60	1.26	4.38	2.36	1.24	1.28	4.74	2.36	1.34	1.34	4.64
313	0, 0	5	-	40	0.33	27	13	.60	12.54	5.80	16.78	13.06	13.24	5.80	16.28	12.56	12.56	5.58
314	0, 0	5	+	40	0.50	10	30	.60	.66	4.22	1.16	.48	.50	5.16	1.26	.58	.58	4.92
315	0, 0	5	-	40	0.50	30	10	.60	16.70	6.66	21.96	17.26	17.40	6.76	22.46	17.74	17.82	6.78
316	0, 0	5	0	60	0	30	30	.60	4.82	4.68	8.26	5.20	5.26	4.84	7.32	4.94	5.00	4.68
317	0, 0	5	+	60	0.16	25	35	.60	3.06	4.72	4.80	2.94	2.98	4.88	4.62	2.94	3.00	4.82
318	0, 0	5	-	60	0.16	35	25	.60	7.46	4.08	12.04	8.76	8.82	5.26	12.10	8.52	8.58	5.30

	D	VR	P	N	Δn	n1	n2	ε	F g	B-F g	F t	F-GG t	F-HF t	B-F t	F g x t	F-GG g x t	F-HF g x t	B-F g x t
319	0, 0	5	+	60	0.33	20	40	.60	1.58	5.10	2.56	1.48	1.48	4.84	2.68	1.62	1.62	5.60
320	0, 0	5	-	60	0.33	40	20	.60	12.00	4.90	16.66	12.82	12.86	5.78	16.02	12.20	12.30	4.98
321	0, 0	5	+	60	0.50	15	45	.60	.76	5.00	1.16	.64	.64	4.82	1.22	.64	.64	4.90
322	0, 0	5	-	60	0.50	45	15	.60	16.42	5.30	21.56	17.22	17.26	5.72	21.60	16.88	16.92	5.84
323	0, 0	5	0	80	0	40	40	.60	5.20	4.96	8.22	5.86	5.90	5.48	7.54	5.08	5.12	4.96
324	0, 0	5	+	80	0.16	34	46	.60	2.90	4.80	5.36	3.30	3.32	5.52	5.40	3.54	3.56	5.84
325	0, 0	5	-	80	0.16	46	34	.60	7.78	4.88	11.48	8.34	8.36	5.34	10.68	7.78	7.82	5.14
326	0, 0	5	+	80	0.33	27	53	.60	1.58	4.50	2.70	1.46	1.46	5.30	2.64	1.38	1.38	4.80
327	0, 0	5	-	80	0.33	53	27	.60	11.40	4.78	16.58	12.72	12.76	5.42	15.82	12.04	12.08	5.32
328	0, 0	5	+	80	0.50	20	60	.60	.56	5.30	1.24	.66	.68	5.66	1.08	.48	.50	5.30
329	0, 0	5	-	80	0.50	60	20	.60	16.62	5.20	23.44	18.18	18.18	5.60	21.76	17.40	17.42	5.62
330	0, 0	5	0	100	0	50	50	.60	5.10	5.16	7.40	5.02	5.02	4.84	7.80	5.16	5.16	4.66
331	0, 0	5	+	100	0.16	42	58	.60	2.80	4.54	4.58	2.90	2.94	5.04	4.62	2.72	2.72	4.90
332	0, 0	5	-	100	0.16	58	42	.60	8.06	5.32	11.00	7.84	7.88	4.62	11.72	7.88	7.88	4.58
333	0, 0	5	+	100	0.33	33	67	.60	1.30	4.74	2.22	1.18	1.18	4.98	2.66	1.42	1.44	5.32
334	0, 0	5	-	100	0.33	67	33	.60	12.80	5.08	16.18	12.32	12.34	4.76	15.52	12.20	12.24	4.76
335	0, 0	5	+	100	0.50	25	75	.60	.52	4.72	1.10	.42	.42	4.96	1.14	.48	.48	4.78
336	0, 0	5	-	100	0.50	75	25	.60	17.58	5.40	21.46	16.60	16.60	4.90	22.42	17.44	17.52	5.12
337	0, 0	5	0	150	0	75	75	.60	5.50	5.42	7.94	5.62	5.62	5.46	8.02	5.44	5.52	5.50
338	0, 0	5	+	150	0.16	63	87	.60	3.24	5.10	5.26	3.28	3.28	5.62	4.68	2.96	2.98	5.04
339	0, 0	5	-	150	0.16	87	63	.60	7.80	4.72	11.88	8.50	8.54	5.16	11.98	8.78	8.80	5.30
340	0, 0	5	+	150	0.33	50	100	.60	1.42	4.74	2.88	1.34	1.34	5.74	2.42	1.30	1.32	5.16
341	0, 0	5	-	150	0.33	100	50	.60	11.24	4.78	16.72	12.26	12.26	5.32	15.60	11.70	11.80	5.02
342	0, 0	5	+	150	0.50	37	113	.60	.48	5.12	1.10	.52	.52	5.76	1.18	.58	.60	4.96
343	0, 0	5	-	150	0.50	113	37	.60	17.40	5.42	22.02	17.46	17.48	5.34	21.64	16.66	16.68	5.08
344	0, 0	5	0	200	0	100	100	.60	4.96	4.86	7.94	5.44	5.44	5.24	7.26	5.02	5.02	4.96
345	0, 0	5	+	200	0.16	84	116	.60	2.68	5.12	4.80	3.26	3.26	5.22	4.82	3.06	3.06	5.14
346	0, 0	5	-	200	0.16	116	84	.60	7.94	4.84	11.54	8.36	8.36	5.04	10.86	7.98	8.02	4.92
347	0, 0	5	+	200	0.33	67	133	.60	1.64	4.88	2.90	1.54	1.54	5.16	2.42	1.40	1.40	4.98
348	0, 0	5	-	200	0.33	133	67	.60	11.88	4.92	17.04	13.12	13.14	5.46	16.64	12.84	12.84	5.40
349	0, 0	5	+	200	0.50	50	150	.60	.60	4.92	1.24	.64	.64	5.40	.94	.38	.38	4.94
350	0, 0	5	-	200	0.50	150	50	.60	17.24	5.50	22.88	18.48	18.50	5.54	22.34	17.86	17.86	5.72

	D	VR	P	N	Δn	n1	n2	ε	F g	B-F g	F t	F-GG t	F-HF t	B-F t	F g x t	F-GG g x t	F-HF g x t	B-F g x t
351	0, 0	1	0	20	0	10	10	.70	4.62	4.20	6.88	5.08	5.44	4.98	6.96	5.08	5.28	5.04
352	0, 0	1	0	20	0.16	8	12	.70	4.66	4.52	6.78	4.98	5.18	4.88	6.88	5.06	5.32	5.12
353	0, 0	1	0	20	0.33	7	13	.70	5.28	5.14	6.50	4.58	4.90	5.12	7.00	4.92	5.28	5.18
354	0, 0	1	0	20	0.50	5	15	.70	5.34	7.54	6.38	4.84	5.04	7.50	6.22	4.64	4.82	7.26
355	0, 0	1	0	40	0	20	20	.70	5.26	5.14	6.72	4.96	5.14	5.02	6.52	4.64	4.82	4.70
356	0, 0	1	0	40	0.16	17	23	.70	4.82	5.10	6.78	5.04	5.22	5.34	6.34	4.36	4.50	4.76
357	0, 0	1	0	40	0.33	13	27	.70	4.94	5.54	7.12	5.04	5.20	5.76	6.50	4.54	4.66	5.10
358	0, 0	1	0	40	0.50	10	30	.70	4.52	6.16	6.66	4.94	5.06	6.62	6.54	4.38	4.52	6.50
359	0, 0	1	0	60	0	30	30	.70	4.68	4.74	6.58	4.90	5.04	4.90	7.02	5.12	5.20	5.20
360	0, 0	1	0	60	0.16	25	35	.70	4.42	4.50	6.68	4.76	4.84	5.10	6.82	4.82	4.90	4.98
361	0, 0	1	0	60	0.33	20	40	.70	5.06	5.74	6.70	4.86	4.86	5.06	6.44	4.96	5.08	5.18
362	0, 0	1	0	60	0.50	15	45	.70	4.98	5.96	6.56	5.04	5.16	5.84	6.50	4.96	5.02	5.42
363	0, 0	1	0	80	0	40	40	.70	4.94	4.84	6.98	5.56	5.64	5.46	6.62	5.14	5.16	5.30
364	0, 0	1	0	80	0.16	34	46	.70	4.70	4.86	7.22	5.48	5.60	5.54	6.94	5.14	5.22	5.52
365	0, 0	1	0	80	0.33	27	53	.70	4.90	5.24	7.24	5.52	5.54	5.60	6.66	4.94	5.04	5.20
366	0, 0	1	0	80	0.50	20	60	.70	5.00	5.88	7.30	5.80	5.82	6.16	6.68	4.46	4.52	5.46
367	0, 0	1	0	100	0	50	50	.70	4.90	5.04	7.06	5.26	5.30	5.46	6.68	5.00	5.06	5.04
368	0, 0	1	0	100	0.16	42	58	.70	5.10	5.00	6.80	5.28	5.36	5.32	6.84	5.14	5.14	5.10
369	0, 0	1	0	100	0.33	33	67	.70	5.12	5.48	6.94	5.36	5.38	5.68	7.08	5.12	5.16	5.38
370	0, 0	1	0	100	0.50	25	75	.70	4.84	5.18	7.08	5.50	5.52	6.00	6.34	4.68	4.68	5.12
371	0, 0	1	0	150	0	75	75	.70	5.62	5.68	6.62	5.06	5.06	5.06	6.98	5.24	5.26	5.52
372	0, 0	1	0	150	0.16	63	87	.70	5.26	5.50	6.80	5.34	5.40	5.46	6.68	4.88	4.94	5.12
373	0, 0	1	0	150	0.33	50	100	.70	4.96	5.38	7.00	5.28	5.32	5.72	7.10	5.34	5.36	5.70
374	0, 0	1	0	150	0.50	37	113	.70	5.06	5.68	6.78	5.24	5.26	5.52	7.20	5.54	5.54	6.10
375	0, 0	1	0	200	0	100	100	.70	5.16	5.20	7.04	5.04	5.08	5.42	6.90	5.22	5.24	5.36
376	0, 0	1	0	200	0.16	84	116	.70	5.12	5.02	6.90	5.12	5.14	5.46	6.98	5.14	5.14	5.44
377	0, 0	1	0	200	0.33	67	133	.70	5.36	5.36	7.06	5.16	5.20	5.44	7.04	5.04	5.08	5.22
378	0, 0	1	0	200	0.50	50	150	.70	4.94	5.18	6.92	5.24	5.26	5.72	6.96	5.20	5.22	5.56
379	0, 0	1.5	0	20	0	10	10	.70	4.30	4.36	7.04	5.14	5.40	5.06	6.88	5.26	5.54	5.02
380	0, 0	1.5	+	20	0.16	8	12	.70	4.14	4.52	6.06	4.18	4.44	5.00	6.16	4.20	4.66	5.10
381	0, 0	1.5	-	20	0.16	12	8	.70	5.88	5.24	8.14	5.96	6.36	5.08	8.04	5.86	6.20	4.98
382	0, 0	1.5	+	20	0.33	7	13	.70	4.06	4.84	5.24	3.60	3.90	4.90	5.20	3.72	3.96	4.96

	D	VR	P	N	Δn	n1	n2	ε	F g	B-F g	F t	F-GG t	F-HF t	B-F t	F g x t	F-GG g x t	F-HF g x t	B-F g x t
383	0, 0	1.5	-	20	0.33	13	7	.70	6.44	5.70	9.04	6.64	7.06	5.72	8.58	6.38	6.82	5.48
384	0, 0	1.5	+	20	0.50	5	15	.70	3.14	6.90	4.24	3.06	3.30	6.90	3.90	2.76	2.90	6.72
385	0, 0	1.5	-	20	0.50	15	5	.70	7.94	7.62	10.32	7.92	8.22	7.74	10.80	8.36	8.72	8.30
386	0, 0	1.5	0	40	0	20	20	.70	4.90	4.72	6.54	4.94	5.10	5.00	6.32	4.72	4.84	4.58
387	0, 0	1.5	+	40	0.16	17	23	.70	4.16	5.16	5.90	4.36	4.44	5.20	5.30	3.88	3.98	4.96
388	0, 0	1.5	-	40	0.16	23	17	.70	5.38	4.94	7.64	5.66	5.82	5.28	7.50	5.42	5.48	5.00
389	0, 0	1.5	+	40	0.33	13	27	.70	3.14	5.42	4.86	3.40	3.44	5.66	4.56	3.24	3.28	4.96
390	0, 0	1.5	-	40	0.33	27	13	.70	6.28	5.52	9.04	7.08	7.22	6.16	8.76	6.58	6.68	5.50
391	0, 0	1.5	+	40	0.50	10	30	.70	2.70	5.68	4.10	2.84	2.92	6.18	3.78	2.74	2.82	6.16
392	0, 0	1.5	-	40	0.50	30	10	.70	7.34	6.50	10.54	8.18	8.28	6.36	10.24	7.90	8.06	6.70
393	0, 0	1.5	0	60	0	30	30	.70	4.40	4.44	6.82	5.20	5.32	5.16	6.82	4.96	5.12	4.90
394	0, 0	1.5	+	60	0.16	25	35	.70	4.06	4.74	5.82	4.00	4.06	5.04	5.68	4.26	4.30	4.96
395	0, 0	1.5	-	60	0.16	35	25	.70	5.48	4.56	8.12	6.14	6.24	5.30	7.56	5.88	6.00	5.00
396	0, 0	1.5	+	60	0.33	20	40	.70	3.90	5.58	4.92	3.44	3.52	5.14	4.82	3.42	3.50	4.94
397	0, 0	1.5	-	60	0.33	40	20	.70	6.26	5.10	9.18	7.02	7.06	5.40	9.08	6.82	6.94	5.42
398	0, 0	1.5	+	60	0.50	15	45	.70	2.96	5.56	4.12	2.92	3.00	5.56	4.04	2.60	2.68	5.44
399	0, 0	1.5	-	60	0.50	45	15	.70	7.68	6.08	10.40	8.14	8.26	5.66	10.20	7.64	7.78	5.68
400	0, 0	1.5	0	80	0	40	40	.70	4.74	4.76	7.18	5.34	5.38	5.38	6.42	4.98	5.02	5.16
401	0, 0	1.5	+	80	0.16	34	46	.70	4.30	4.74	6.28	4.96	5.02	5.70	5.86	4.50	4.56	5.32
402	0, 0	1.5	-	80	0.16	46	34	.70	5.72	4.98	8.10	6.32	6.46	5.68	7.80	6.02	6.10	5.32
403	0, 0	1.5	+	80	0.33	27	53	.70	3.20	4.86	5.32	4.08	4.16	5.72	4.92	3.34	3.46	5.24
404	0, 0	1.5	-	80	0.33	53	27	.70	6.44	4.88	9.56	7.30	7.34	5.70	8.92	6.54	6.60	5.62
405	0, 0	1.5	+	80	0.50	20	60	.70	3.06	5.82	4.80	3.56	3.58	6.10	3.76	2.70	2.76	5.34
406	0, 0	1.5	-	80	0.50	60	20	.70	7.58	5.66	10.92	8.74	8.82	6.38	10.40	8.28	8.34	6.02
407	0, 0	1.5	0	100	0	50	50	.70	4.86	4.94	6.96	5.30	5.34	5.44	6.64	5.06	5.06	5.04
408	0, 0	1.5	+	100	0.16	42	58	.70	4.38	5.06	6.22	4.70	4.74	5.56	5.78	4.26	4.32	5.24
409	0, 0	1.5	-	100	0.16	58	42	.70	5.90	5.32	8.00	6.16	6.24	5.26	7.52	5.62	5.66	5.10
410	0, 0	1.5	+	100	0.33	33	67	.70	3.58	5.30	5.24	3.92	3.94	5.66	5.00	3.60	3.62	5.38
411	0, 0	1.5	-	100	0.33	67	33	.70	6.90	5.30	8.66	6.86	6.90	5.34	9.26	6.74	6.86	4.80
412	0, 0	1.5	+	100	0.50	25	75	.70	2.80	5.14	4.56	3.32	3.32	5.94	3.74	2.60	2.64	5.20
413	0, 0	1.5	-	100	0.50	75	25	.70	8.00	5.88	9.88	7.88	7.92	5.80	10.44	7.88	8.10	5.76
414	0, 0	1.5	0	150	0	75	75	.70	5.66	5.68	6.52	4.78	4.82	5.10	6.80	5.20	5.24	5.24

	D	VR	P	N	Δn	n1	n2	ε	F g	B-F g	F t	F-GG t	F-HF t	B-F t	F g x t	F-GG g x t	F-HF g x t	B-F g x t
415	0, 0	1.5	+	150	0.16	63	87	.70	4.60	5.50	5.98	4.52	4.56	5.46	5.86	4.36	4.38	5.12
416	0, 0	1.5	-	150	0.16	87	63	.70	6.10	5.28	7.46	5.50	5.56	4.84	8.00	6.04	6.06	5.40
417	0, 0	1.5	+	150	0.33	50	100	.70	3.62	5.08	5.20	3.90	3.96	5.46	5.36	3.68	3.70	5.60
418	0, 0	1.5	-	150	0.33	100	50	.70	6.42	4.82	8.82	6.84	6.88	5.04	8.66	6.50	6.50	4.72
419	0, 0	1.5	+	150	0.50	37	113	.70	2.92	5.38	4.40	3.08	3.10	5.70	4.50	2.90	2.92	5.80
420	0, 0	1.5	-	150	0.50	113	37	.70	7.66	5.16	10.04	7.64	7.66	5.14	9.76	7.58	7.62	5.00
421	0, 0	1.5	0	200	0	100	100	.70	5.16	5.16	6.96	5.06	5.06	5.30	6.74	5.02	5.02	5.28
422	0, 0	1.5	+	200	0.16	84	116	.70	4.28	5.30	6.02	4.22	4.22	5.28	6.16	4.48	4.50	5.58
423	0, 0	1.5	-	200	0.16	116	84	.70	5.92	5.36	7.86	5.80	5.80	5.30	7.88	5.84	5.92	5.38
424	0, 0	1.5	+	200	0.33	67	133	.70	3.88	5.28	5.10	3.72	3.72	5.26	5.04	3.60	3.62	5.20
425	0, 0	1.5	-	200	0.33	133	67	.70	7.02	5.38	9.14	6.72	6.76	5.26	8.90	6.58	6.58	5.20
426	0, 0	1.5	+	200	0.50	50	150	.70	2.96	5.36	4.44	3.20	3.22	5.60	4.26	2.96	2.96	5.52
427	0, 0	1.5	-	200	0.50	150	50	.70	8.02	5.26	10.32	7.90	7.90	5.24	10.16	7.90	7.94	5.28
428	0, 0	2	0	20	0	10	10	.70	4.66	4.38	7.16	5.20	5.42	5.04	7.14	5.34	5.62	5.02
429	0, 0	2	+	20	0.16	8	12	.70	3.78	4.52	5.42	3.74	4.00	4.90	5.42	3.58	3.94	5.04
430	0, 0	2	-	20	0.16	12	8	.70	6.98	5.34	9.28	6.88	7.22	5.18	9.14	6.76	7.10	5.00
431	0, 0	2	+	20	0.33	7	13	.70	3.44	4.66	4.42	2.96	3.16	4.70	4.22	2.92	3.20	4.74
432	0, 0	2	-	20	0.33	13	7	.70	7.60	5.74	10.66	8.22	8.58	5.90	10.26	7.90	8.28	5.60
433	0, 0	2	+	20	0.50	5	15	.70	2.14	6.36	2.96	2.08	2.22	6.42	2.66	1.84	2.02	6.24
434	0, 0	2	-	20	0.50	15	5	.70	10.04	7.84	13.28	10.36	10.70	7.80	13.76	11.04	11.60	8.52
435	0, 0	2	0	40	0	20	20	.70	4.92	4.76	6.68	5.12	5.16	4.94	6.48	4.50	4.70	4.42
436	0, 0	2	+	40	0.16	17	23	.70	3.98	5.12	5.28	3.98	4.04	5.22	5.00	3.68	3.72	4.98
437	0, 0	2	-	40	0.16	23	17	.70	6.04	4.74	8.48	6.48	6.62	5.32	8.50	6.08	6.32	5.02
438	0, 0	2	+	40	0.33	13	27	.70	2.42	5.20	3.80	2.70	2.76	5.42	3.56	2.52	2.60	4.88
439	0, 0	2	-	40	0.33	27	13	.70	7.74	5.58	10.72	8.30	8.56	6.28	10.86	8.06	8.38	5.42
440	0, 0	2	+	40	0.50	10	30	.70	1.92	5.34	2.94	1.86	1.94	6.06	2.76	1.86	1.92	5.90
441	0, 0	2	-	40	0.50	30	10	.70	9.42	6.48	13.04	10.60	10.78	6.40	13.30	10.56	10.76	6.68
442	0, 0	2	0	60	0	30	30	.70	4.40	4.42	6.98	5.32	5.46	5.18	7.00	5.20	5.24	4.98
443	0, 0	2	+	60	0.16	25	35	.70	3.78	4.74	5.28	3.88	3.96	5.06	5.10	3.90	3.94	5.00
444	0, 0	2	-	60	0.16	35	25	.70	6.12	4.62	8.82	6.94	7.08	5.40	8.56	6.50	6.56	4.94
445	0, 0	2	+	60	0.33	20	40	.70	2.88	5.26	3.94	2.76	2.80	5.04	3.88	2.48	2.66	4.86
446	0, 0	2	-	60	0.33	40	20	.70	7.56	5.10	10.74	8.28	8.38	5.58	10.94	8.40	8.54	5.30

	D	VR	P	N	Δn	n1	n2	ε	F g	B-F g	F t	F-GG t	F-HF t	B-F t	F g x t	F-GG g x t	F-HF g x t	B-F g x t
447	0, 0	2	+	60	0.50	15	45	.70	2.06	5.40	2.78	1.94	1.96	5.54	2.62	1.82	1.84	5.20
448	0, 0	2	-	60	0.50	45	15	.70	9.64	6.24	12.88	10.62	10.70	5.76	13.24	10.28	10.38	5.90
449	0, 0	2	0	80	0	40	40	.70	4.74	4.50	7.16	5.22	5.30	5.48	6.52	5.08	5.10	4.96
450	0, 0	2	+	80	0.16	34	46	.70	3.84	4.60	5.80	4.30	4.36	5.50	5.36	3.98	4.08	5.24
451	0, 0	2	-	80	0.16	46	34	.70	6.10	4.82	8.82	6.82	6.90	5.70	8.30	6.54	6.62	5.42
452	0, 0	2	+	80	0.33	27	53	.70	2.64	4.54	4.24	3.20	3.24	5.62	3.82	2.70	2.74	5.12
453	0, 0	2	-	80	0.33	53	27	.70	7.50	4.88	11.32	8.66	8.76	5.62	10.40	8.18	8.28	5.48
454	0, 0	2	+	80	0.50	20	60	.70	2.12	5.46	3.54	2.24	2.26	6.10	2.50	1.66	1.72	5.10
455	0, 0	2	-	80	0.50	60	20	.70	9.72	5.58	13.58	11.24	11.32	6.42	13.16	10.60	10.76	6.30
456	0, 0	2	0	100	0	50	50	.70	4.88	4.90	7.04	5.38	5.42	5.26	6.56	4.88	4.90	4.94
457	0, 0	2	+	100	0.16	42	58	.70	3.86	4.94	5.46	4.04	4.12	5.26	5.30	3.74	3.80	5.00
458	0, 0	2	-	100	0.16	58	42	.70	6.28	5.22	8.62	6.56	6.70	5.28	8.28	6.16	6.22	4.94
459	0, 0	2	+	100	0.33	33	67	.70	2.74	5.00	4.38	3.12	3.24	5.64	3.92	2.68	2.70	5.20
460	0, 0	2	-	100	0.33	67	33	.70	8.12	5.26	10.80	8.26	8.38	5.44	11.24	8.48	8.56	4.86
461	0, 0	2	+	100	0.50	25	75	.70	1.96	4.86	3.20	1.92	2.00	5.82	2.40	1.58	1.58	5.10
462	0, 0	2	-	100	0.50	75	25	.70	10.38	5.90	12.34	9.98	10.06	5.54	13.40	10.66	10.72	5.62
463	0, 0	2	0	150	0	75	75	.70	5.90	5.76	6.42	4.78	4.78	4.84	6.86	5.00	5.04	5.18
464	0, 0	2	+	150	0.16	63	87	.70	4.26	5.48	5.30	3.68	3.70	5.12	5.44	3.80	3.82	5.12
465	0, 0	2	-	150	0.16	87	63	.70	6.52	5.48	8.02	6.16	6.20	4.84	8.62	6.62	6.66	5.28
466	0, 0	2	+	150	0.33	50	100	.70	2.92	4.98	4.22	3.02	3.02	5.36	4.14	2.84	2.90	5.48
467	0, 0	2	-	150	0.33	100	50	.70	7.60	4.74	10.38	8.06	8.06	5.12	10.46	8.00	8.04	4.64
468	0, 0	2	+	150	0.50	37	113	.70	1.94	5.30	3.00	2.32	2.32	5.64	2.84	2.00	2.02	5.54
469	0, 0	2	-	150	0.50	113	37	.70	9.74	5.18	12.58	10.04	10.10	4.92	12.44	9.90	9.94	4.96
470	0, 0	2	0	200	0	100	100	.70	5.04	4.84	6.92	5.14	5.16	5.36	6.84	4.90	4.90	5.04
471	0, 0	2	+	200	0.16	84	116	.70	3.86	5.22	5.44	3.84	3.88	5.32	5.44	4.12	4.16	5.34
472	0, 0	2	-	200	0.16	116	84	.70	6.44	5.00	8.60	6.34	6.36	5.18	8.50	6.68	6.72	4.90
473	0, 0	2	+	200	0.33	67	133	.70	3.12	5.28	4.08	2.82	2.82	5.30	4.00	2.82	2.84	5.20
474	0, 0	2	-	200	0.33	133	67	.70	8.44	5.40	10.52	8.20	8.22	5.30	10.34	8.28	8.32	5.46
475	0, 0	2	+	200	0.50	50	150	.70	1.98	5.32	3.14	2.12	2.12	5.44	2.88	1.86	1.90	5.38
476	0, 0	2	-	200	0.50	150	50	.70	10.30	5.50	13.00	10.42	10.48	5.24	12.66	10.28	10.30	5.34
477	0, 0	5	0	20	0	10	10	.70	5.26	4.12	7.70	5.52	5.88	4.78	7.98	6.00	6.28	5.38
478	0, 0	5	+	20	0.16	8	12	.70	3.22	4.74	3.78	2.72	2.90	4.76	3.80	2.72	2.86	4.72

	D	VR	P	N	Δn	n1	n2	ε	F g	B-F g	F t	F-GG t	F-HF t	B-F t	F g x t	F-GG g x t	F-HF g x t	B-F g x t
479	0, 0	5	-	20	0.16	12	8	.70	9.44	5.40	12.46	9.94	10.34	5.38	12.30	9.38	9.76	5.22
480	0, 0	5	+	20	0.33	7	13	.70	2.34	4.22	2.64	1.72	1.94	4.40	2.50	1.72	1.88	4.06
481	0, 0	5	-	20	0.33	13	7	.70	11.74	6.06	15.82	12.52	12.78	5.66	15.62	12.56	12.92	5.80
482	0, 0	5	+	20	0.50	5	15	.70	.86	5.02	.86	.60	.64	5.30	1.04	.70	.76	4.70
483	0, 0	5	-	20	0.50	15	5	.70	18.30	8.86	23.46	19.94	20.48	8.06	23.30	19.42	20.14	8.64
484	0, 0	5	0	40	0	20	20	.70	5.00	4.66	6.84	5.38	5.50	5.22	6.86	4.94	5.06	4.92
485	0, 0	5	+	40	0.16	17	23	.70	2.96	4.78	4.34	3.36	3.38	5.20	4.16	2.90	3.00	4.86
486	0, 0	5	-	40	0.16	23	17	.70	7.50	4.84	10.54	8.12	8.32	5.26	10.66	8.24	8.42	5.42
487	0, 0	5	+	40	0.33	13	27	.70	1.20	4.44	2.10	1.40	1.42	5.06	2.02	1.40	1.42	5.06
488	0, 0	5	-	40	0.33	27	13	.70	12.32	5.34	16.48	13.62	13.90	5.98	16.96	13.72	13.94	5.48
489	0, 0	5	+	40	0.50	10	30	.70	.62	4.60	1.10	.68	.68	5.40	.94	.58	.62	5.66
490	0, 0	5	-	40	0.50	30	10	.70	16.48	6.30	22.14	18.84	19.02	6.60	22.80	19.36	19.58	6.20
491	0, 0	5	0	60	0	30	30	.70	4.58	4.26	7.42	5.44	5.56	5.10	7.08	5.28	5.36	5.02
492	0, 0	5	+	60	0.16	25	35	.70	2.94	4.78	4.36	2.92	3.00	5.22	4.28	2.94	2.98	5.04
493	0, 0	5	-	60	0.16	35	25	.70	7.74	4.50	10.82	8.52	8.64	5.16	10.54	8.12	8.18	4.76
494	0, 0	5	+	60	0.33	20	40	.70	1.52	4.92	2.32	1.48	1.48	5.20	1.82	1.22	1.28	5.10
495	0, 0	5	-	60	0.33	40	20	.70	11.96	4.96	16.42	13.18	13.38	5.32	16.30	13.24	13.38	5.26
496	0, 0	5	+	60	0.50	15	45	.70	.64	4.70	.98	.56	.56	5.08	.76	.56	.56	4.92
497	0, 0	5	-	60	0.50	45	15	.70	16.46	6.12	22.22	18.80	19.00	5.84	22.46	19.24	19.34	5.88
498	0, 0	5	0	80	0	40	40	.70	4.90	4.54	7.38	5.56	5.64	5.30	6.68	5.18	5.18	4.98
499	0, 0	5	+	80	0.16	34	46	.70	2.90	4.60	4.32	3.26	3.34	5.40	4.16	3.02	3.14	4.98
500	0, 0	5	-	80	0.16	46	34	.70	7.48	4.84	10.82	8.46	8.54	5.48	10.06	7.66	7.70	5.46
501	0, 0	5	+	80	0.33	27	53	.70	1.40	4.28	2.48	1.70	1.74	5.48	1.84	1.04	1.08	5.00
502	0, 0	5	-	80	0.33	53	27	.70	11.54	5.00	16.50	13.20	13.30	5.44	15.66	12.58	12.64	5.34
503	0, 0	5	+	80	0.50	20	60	.70	.56	5.02	1.18	.72	.72	5.68	.64	.40	.40	4.52
504	0, 0	5	-	80	0.50	60	20	.70	16.22	5.58	22.72	19.48	19.62	6.34	22.50	19.02	19.08	6.10
505	0, 0	5	0	100	0	50	50	.70	5.10	5.12	7.10	5.06	5.08	5.24	6.42	4.94	4.96	4.76
506	0, 0	5	+	100	0.16	42	58	.70	2.76	4.42	4.22	2.88	2.90	5.16	4.00	2.90	2.92	4.80
507	0, 0	5	-	100	0.16	58	42	.70	7.84	5.16	11.00	8.84	8.88	5.42	10.66	8.08	8.14	5.00
508	0, 0	5	+	100	0.33	33	67	.70	1.10	4.72	2.20	1.40	1.46	5.34	2.06	1.36	1.38	5.00
509	0, 0	5	-	100	0.33	67	33	.70	12.24	5.26	16.18	12.82	12.98	5.28	16.38	13.34	13.42	5.10
510	0, 0	5	+	100	0.50	25	75	.70	.58	4.44	.84	.50	.52	5.44	.62	.28	.28	5.08

	D	VR	P	N	Δn	n1	n2	ε	F g	B-F g	F t	F-GG t	F-HF t	B-F t	F g x t	F-GG g x t	F-HF g x t	B-F g x t
511	0, 0	5	-	100	0.50	75	25	.70	17.04	5.68	21.92	18.32	18.46	5.50	21.94	18.82	18.92	5.92
512	0, 0	5	0	150	0	75	75	.70	5.90	5.80	6.54	4.76	4.78	4.84	6.84	5.06	5.08	5.10
513	0, 0	5	+	150	0.16	63	87	.70	3.34	5.32	3.76	2.68	2.68	4.92	4.20	2.86	2.88	5.26
514	0, 0	5	-	150	0.16	87	63	.70	8.10	5.06	10.28	8.04	8.08	5.06	10.82	8.38	8.48	5.22
515	0, 0	5	+	150	0.33	50	100	.70	1.54	5.10	2.10	1.44	1.46	5.10	2.20	1.30	1.30	5.40
516	0, 0	5	-	150	0.33	100	50	.70	11.20	4.72	15.48	12.32	12.34	4.76	15.18	12.04	12.06	4.52
517	0, 0	5	+	150	0.50	37	113	.70	.44	5.08	.88	.54	.54	5.64	.96	.70	.70	5.48
518	0, 0	5	-	150	0.50	113	37	.70	16.92	5.12	22.16	18.78	18.82	5.00	21.72	18.46	18.56	4.72
519	0, 0	5	0	200	0	100	100	.70	4.96	5.10	6.96	5.36	5.38	5.20	6.50	4.60	4.60	4.62
520	0, 0	5	+	200	0.16	84	116	.70	2.80	5.06	4.42	3.06	3.06	5.52	4.00	2.92	2.92	5.22
521	0, 0	5	-	200	0.16	116	84	.70	8.16	5.02	10.54	8.04	8.06	4.98	10.58	8.32	8.34	4.86
522	0, 0	5	+	200	0.33	67	133	.70	1.76	5.10	2.08	1.38	1.38	5.06	2.02	1.44	1.44	5.02
523	0, 0	5	-	200	0.33	133	67	.70	12.52	5.34	15.62	12.62	12.68	4.96	15.54	12.78	12.80	4.96
524	0, 0	5	+	200	0.50	50	150	.70	.58	4.94	.96	.46	.46	5.18	.68	.36	.36	5.30
525	0, 0	5	-	200	0.50	150	50	.70	17.66	5.38	22.26	18.78	18.80	5.10	22.36	18.70	18.74	5.10
526	0, 0	1	0	20	0	10	10	.80	4.40	4.00	5.84	4.40	4.68	4.38	6.44	5.18	5.56	4.96
527	0, 0	1	0	20	0.16	8	12	.80	4.68	4.34	5.40	4.30	4.62	4.60	5.74	4.78	5.16	4.78
528	0, 0	1	0	20	0.33	7	13	.80	4.94	5.42	5.50	4.30	4.66	5.20	6.06	4.94	5.26	5.56
529	0, 0	1	0	20	0.50	5	15	.80	4.64	6.80	5.72	4.48	4.92	6.78	6.06	4.68	5.04	7.12
530	0, 0	1	0	40	0	20	20	.80	5.14	5.04	5.54	4.50	4.74	4.50	6.34	5.08	5.32	5.20
531	0, 0	1	0	40	0.16	17	23	.80	5.16	5.46	5.38	4.06	4.36	4.64	5.70	4.50	4.66	4.92
532	0, 0	1	0	40	0.33	13	27	.80	4.50	5.00	5.40	4.36	4.52	5.02	6.04	4.82	4.96	5.88
533	0, 0	1	0	40	0.50	10	30	.80	4.40	5.76	5.36	4.10	4.32	6.04	6.32	5.18	5.34	6.72
534	0, 0	1	0	60	0	30	30	.80	5.42	5.34	5.90	4.90	5.04	5.02	5.90	4.78	4.94	5.04
535	0, 0	1	0	60	0.16	25	35	.80	4.98	5.18	5.98	4.84	4.98	4.96	6.16	4.92	5.06	5.04
536	0, 0	1	0	60	0.33	20	40	.80	5.16	5.52	5.82	4.74	4.82	4.92	6.68	5.30	5.40	5.84
537	0, 0	1	0	60	0.50	15	45	.80	5.12	6.06	5.80	4.50	4.64	5.10	6.02	5.06	5.16	5.68
538	0, 0	1	0	80	0	40	40	.80	5.16	5.10	6.34	5.12	5.16	5.32	5.56	4.54	4.58	4.58
539	0, 0	1	0	80	0.16	34	46	.80	5.04	5.26	6.36	5.16	5.22	5.48	5.84	4.72	4.78	5.28
540	0, 0	1	0	80	0.33	27	53	.80	5.22	5.50	6.02	4.98	5.06	5.22	5.50	4.42	4.50	4.84
541	0, 0	1	0	80	0.50	20	60	.80	5.26	6.02	6.26	5.14	5.24	5.74	6.18	5.22	5.28	5.74
542	0, 0	1	0	100	0	50	50	.80	5.64	5.54	5.78	4.84	4.90	4.88	6.10	4.72	4.80	4.96

	D	VR	P	N	Δn	n1	n2	ε	F g	B-F g	F t	F-GG t	F-HF t	B-F t	F g x t	F-GG g x t	F-HF g x t	B-F g x t
543	0, 0	1	0	100	0.16	42	58	.80	4.80	4.86	5.98	5.02	5.08	5.22	5.48	4.50	4.60	4.70
544	0, 0	1	0	100	0.33	33	67	.80	5.20	5.86	5.94	4.70	4.86	5.16	5.88	4.92	4.96	5.04
545	0, 0	1	0	100	0.50	25	75	.80	5.14	5.94	5.88	4.62	4.70	5.22	5.48	4.38	4.42	4.88
546	0, 0	1	0	150	0	75	75	.80	5.78	5.64	6.28	5.10	5.14	5.16	6.80	5.56	5.62	5.66
547	0, 0	1	0	150	0.16	63	87	.80	5.80	6.08	6.46	5.16	5.20	5.40	6.24	4.98	5.12	5.30
548	0, 0	1	0	150	0.33	50	100	.80	5.60	5.98	6.40	5.24	5.26	5.84	5.84	4.88	4.92	5.08
549	0, 0	1	0	150	0.50	37	113	.80	5.30	5.78	6.02	4.78	4.78	5.34	5.78	4.74	4.76	5.20
550	0, 0	1	0	200	0	100	100	.80	5.30	5.44	6.10	4.98	5.06	5.12	6.30	5.48	5.50	5.62
551	0, 0	1	0	200	0.16	84	116	.80	5.36	5.36	6.32	5.04	5.06	5.26	6.32	5.14	5.18	5.26
552	0, 0	1	0	200	0.33	67	133	.80	5.70	5.76	6.26	5.08	5.18	5.08	6.12	5.32	5.34	5.42
553	0, 0	1	0	200	0.50	50	150	.80	5.50	5.78	6.20	4.98	5.02	5.40	5.48	4.50	4.52	4.86
554	0, 0	1.5	0	20	0	10	10	.80	4.54	4.30	5.94	4.62	4.96	4.56	6.36	5.00	5.34	4.72
555	0, 0	1.5	+	20	0.16	8	12	.80	4.16	4.38	4.52	3.54	3.84	4.34	4.90	3.74	4.00	4.58
556	0, 0	1.5	-	20	0.16	12	8	.80	5.94	5.06	7.38	5.94	6.38	4.88	7.78	6.10	6.66	5.24
557	0, 0	1.5	+	20	0.33	7	13	.80	3.88	5.12	4.02	3.14	3.40	4.94	4.62	3.54	3.80	5.26
558	0, 0	1.5	-	20	0.33	13	7	.80	6.14	5.64	7.88	6.42	6.82	5.76	8.42	6.90	7.18	5.68
559	0, 0	1.5	+	20	0.50	5	15	.80	2.86	6.40	3.20	2.44	2.66	6.48	3.60	2.80	3.04	6.32
560	0, 0	1.5	-	20	0.50	15	5	.80	7.86	8.58	9.66	7.68	8.32	7.98	9.84	7.92	8.22	7.52
561	0, 0	1.5	0	40	0	20	20	.80	4.88	4.90	5.92	4.62	4.84	4.80	6.12	5.02	5.20	5.00
562	0, 0	1.5	+	40	0.16	17	23	.80	4.64	5.18	4.72	3.64	3.80	4.44	4.94	3.86	4.08	4.64
563	0, 0	1.5	-	40	0.16	23	17	.80	5.86	5.44	6.74	5.56	5.72	5.06	6.96	5.66	5.78	5.50
564	0, 0	1.5	+	40	0.33	13	27	.80	2.96	4.62	3.58	2.90	3.00	4.64	4.16	3.58	3.66	5.32
565	0, 0	1.5	-	40	0.33	27	13	.80	6.82	5.66	7.94	6.64	6.82	5.52	7.38	6.06	6.22	5.14
566	0, 0	1.5	+	40	0.50	10	30	.80	2.62	5.22	3.04	2.32	2.48	5.64	3.88	3.02	3.14	6.44
567	0, 0	1.5	-	40	0.50	30	10	.80	7.54	6.46	8.82	7.28	7.46	5.90	8.72	7.34	7.70	5.78
568	0, 0	1.5	0	60	0	30	30	.80	5.40	5.20	6.08	5.06	5.18	5.18	6.08	4.88	4.98	5.02
569	0, 0	1.5	+	60	0.16	25	35	.80	4.32	5.00	5.02	4.10	4.10	4.92	5.28	3.86	3.98	5.10
570	0, 0	1.5	-	60	0.16	35	25	.80	5.78	5.00	7.08	5.78	5.98	5.46	7.00	5.72	5.82	5.02
571	0, 0	1.5	+	60	0.33	20	40	.80	3.90	5.42	4.10	3.14	3.26	5.22	4.78	3.92	3.98	5.82
572	0, 0	1.5	-	60	0.33	40	20	.80	6.16	5.02	8.54	6.98	7.14	5.38	7.28	5.88	5.94	4.70
573	0, 0	1.5	+	60	0.50	15	45	.80	3.12	5.74	3.30	2.70	2.76	5.20	3.84	3.14	3.26	5.66
574	0, 0	1.5	-	60	0.50	45	15	.80	7.62	5.90	9.88	8.40	8.58	5.64	8.84	7.54	7.66	5.56

	D	VR	P	N	Δn	n1	n2	ε	F g	B-F g	F t	F-GG t	F-HF t	B-F t	F g x t	F-GG g x t	F-HF g x t	B-F g x t
575	0, 0	1.5	0	80	0	40	40	.80	5.08	5.04	6.28	5.04	5.06	5.22	5.62	4.70	4.74	4.72
576	0, 0	1.5	+	80	0.16	34	46	.80	4.34	4.94	5.46	4.42	4.46	5.50	5.26	4.18	4.26	5.24
577	0, 0	1.5	-	80	0.16	46	34	.80	5.38	4.74	7.34	5.88	5.94	5.40	6.82	5.44	5.54	4.66
578	0, 0	1.5	+	80	0.33	27	53	.80	3.84	5.26	4.50	3.42	3.44	5.34	3.86	3.14	3.18	4.72
579	0, 0	1.5	-	80	0.33	53	27	.80	6.24	4.92	8.50	7.00	7.06	5.54	8.18	6.46	6.58	5.04
580	0, 0	1.5	+	80	0.50	20	60	.80	3.26	5.82	3.52	2.60	2.70	5.58	3.76	2.94	3.00	5.70
581	0, 0	1.5	-	80	0.50	60	20	.80	7.50	5.72	10.04	8.44	8.64	5.98	9.50	7.96	8.04	5.42
582	0, 0	1.5	0	100	0	50	50	.80	5.28	5.36	6.10	4.66	4.70	4.84	6.28	5.18	5.24	5.12
583	0, 0	1.5	+	100	0.16	42	58	.80	4.16	4.68	5.06	4.12	4.18	4.90	4.64	3.78	3.88	4.60
584	0, 0	1.5	-	100	0.16	58	42	.80	5.94	5.32	7.00	5.54	5.64	4.84	7.20	6.14	6.26	5.44
585	0, 0	1.5	+	100	0.33	33	67	.80	3.70	5.36	3.92	3.28	3.30	5.14	4.30	3.52	3.58	5.02
586	0, 0	1.5	-	100	0.33	67	33	.80	7.00	5.84	8.52	6.80	7.00	5.16	8.60	7.36	7.40	5.58
587	0, 0	1.5	+	100	0.50	25	75	.80	3.18	5.54	3.26	2.50	2.54	5.02	3.38	2.60	2.68	4.96
588	0, 0	1.5	-	100	0.50	75	25	.80	7.96	5.98	9.16	7.44	7.54	5.44	9.52	7.94	8.00	5.48
589	0, 0	1.5	0	150	0	75	75	.80	5.72	5.62	6.20	5.24	5.24	5.26	6.84	5.42	5.46	5.76
590	0, 0	1.5	+	150	0.16	63	87	.80	4.92	5.94	5.32	4.28	4.32	5.38	5.22	4.32	4.36	5.28
591	0, 0	1.5	-	150	0.16	87	63	.80	5.72	5.10	7.60	6.16	6.22	5.64	7.46	6.28	6.30	5.74
592	0, 0	1.5	+	150	0.33	50	100	.80	4.04	5.64	4.54	3.54	3.56	5.78	4.12	3.14	3.14	5.22
593	0, 0	1.5	-	150	0.33	100	50	.80	6.18	4.50	8.50	7.28	7.28	5.80	8.50	7.12	7.16	5.24
594	0, 0	1.5	+	150	0.50	37	113	.80	3.34	5.46	3.58	2.92	2.94	5.60	3.34	2.74	2.78	5.06
595	0, 0	1.5	-	150	0.50	113	37	.80	7.36	5.26	10.16	8.48	8.56	5.54	9.48	8.24	8.24	5.82
596	0, 0	1.5	0	200	0	100	100	.80	5.38	5.44	5.90	4.86	4.86	5.06	6.30	5.54	5.54	5.50
597	0, 0	1.5	+	200	0.16	84	116	.80	4.48	5.36	5.18	4.06	4.12	5.14	5.52	4.28	4.30	5.46
598	0, 0	1.5	-	200	0.16	116	84	.80	6.10	5.20	7.16	5.68	5.72	4.90	7.60	6.30	6.34	5.26
599	0, 0	1.5	+	200	0.33	67	133	.80	3.90	5.68	4.26	3.44	3.46	5.20	4.60	3.52	3.52	5.36
600	0, 0	1.5	-	200	0.33	133	67	.80	6.80	5.00	8.38	6.92	6.98	5.26	9.10	7.54	7.60	5.46
601	0, 0	1.5	+	200	0.50	50	150	.80	3.30	5.62	3.52	2.70	2.74	5.36	3.40	2.60	2.64	5.02
602	0, 0	1.5	-	200	0.50	150	50	.80	7.90	5.48	9.78	8.36	8.40	5.24	9.52	8.30	8.30	5.58
603	0, 0	2	0	20	0	10	10	.80	4.60	4.22	6.06	4.78	5.12	4.42	6.38	5.10	5.40	4.74
604	0, 0	2	+	20	0.16	8	12	.80	3.62	4.48	3.94	3.18	3.38	4.24	4.14	3.24	3.58	4.54
605	0, 0	2	-	20	0.16	12	8	.80	6.84	5.28	8.46	6.90	7.28	5.24	8.66	7.08	7.50	5.12
606	0, 0	2	+	20	0.33	7	13	.80	3.20	4.92	3.22	2.50	2.68	4.76	3.68	2.86	3.18	5.18

	D	VR	P	N	Δn	n1	n2	ε	F g	B-F g	F t	F-GG t	F-HF t	B-F t	F g x t	F-GG g x t	F-HF g x t	B-F g x t
607	0, 0	2	-	20	0.33	13	7	.80	7.16	5.94	9.42	7.76	8.44	5.84	9.80	8.02	8.50	5.86
608	0, 0	2	+	20	0.50	5	15	.80	2.08	5.92	2.22	1.74	1.88	5.86	2.38	1.90	2.00	6.02
609	0, 0	2	-	20	0.50	15	5	.80	10.12	9.02	12.66	10.68	11.14	8.28	12.36	10.50	11.02	7.72
610	0, 0	2	0	40	0	20	20	.80	5.06	4.88	5.94	4.74	4.92	4.62	6.20	4.98	5.24	4.72
611	0, 0	2	+	40	0.16	17	23	.80	4.26	5.02	4.32	3.30	3.46	4.60	4.44	3.34	3.56	4.74
612	0, 0	2	-	40	0.16	23	17	.80	6.30	5.18	7.30	6.24	6.36	5.16	7.58	6.24	6.50	5.30
613	0, 0	2	+	40	0.33	13	27	.80	2.42	4.54	2.80	2.18	2.30	4.42	3.42	2.82	2.88	5.12
614	0, 0	2	-	40	0.33	27	13	.80	8.14	5.82	9.92	8.50	8.66	5.52	9.16	7.54	7.80	5.30
615	0, 0	2	+	40	0.50	10	30	.80	1.78	4.98	1.92	1.48	1.54	5.08	2.50	1.88	2.08	6.14
616	0, 0	2	-	40	0.50	30	10	.80	9.52	6.54	11.64	10.04	10.20	5.92	11.70	9.92	10.20	5.94
617	0, 0	2	0	60	0	30	30	.80	5.16	4.98	6.28	5.22	5.30	5.32	6.04	4.82	5.00	4.84
618	0, 0	2	+	60	0.16	25	35	.80	3.78	4.92	4.56	3.72	3.78	4.90	4.74	3.60	3.68	4.98
619	0, 0	2	-	60	0.16	35	25	.80	6.34	4.92	8.08	6.70	6.82	5.54	7.88	6.64	6.74	5.20
620	0, 0	2	+	60	0.33	20	40	.80	2.98	5.26	3.16	2.62	2.64	5.10	3.92	3.02	3.06	5.78
621	0, 0	2	-	60	0.33	40	20	.80	7.18	5.14	10.46	8.62	8.78	5.70	9.22	7.42	7.66	4.88
622	0, 0	2	+	60	0.50	15	45	.80	2.16	5.60	2.36	1.64	1.70	5.50	2.62	2.04	2.10	5.56
623	0, 0	2	-	60	0.50	45	15	.80	9.46	6.22	12.64	10.92	11.16	5.92	12.02	10.04	10.22	5.72
624	0, 0	2	0	80	0	40	40	.80	4.98	5.04	6.40	5.00	5.02	4.90	5.84	4.66	4.76	4.72
625	0, 0	2	+	80	0.16	34	46	.80	3.78	4.84	4.98	3.96	4.06	5.26	4.82	3.84	3.86	5.06
626	0, 0	2	-	80	0.16	46	34	.80	5.88	4.98	8.16	6.62	6.66	5.14	7.64	6.04	6.10	4.88
627	0, 0	2	+	80	0.33	27	53	.80	3.04	4.96	3.46	2.68	2.72	5.04	3.06	2.46	2.50	4.76
628	0, 0	2	-	80	0.33	53	27	.80	7.26	4.86	10.20	8.62	8.74	5.46	9.98	8.18	8.34	5.18
629	0, 0	2	+	80	0.50	20	60	.80	2.22	5.62	2.30	1.70	1.72	5.52	2.56	1.80	1.84	5.52
630	0, 0	2	-	80	0.50	60	20	.80	9.44	5.40	12.92	11.12	11.20	5.96	12.32	10.54	10.64	5.28
631	0, 0	2	0	100	0	50	50	.80	5.12	5.20	5.96	4.78	4.82	4.78	6.22	5.16	5.26	5.28
632	0, 0	2	+	100	0.16	42	58	.80	3.88	4.58	4.56	3.72	3.80	4.82	4.26	3.36	3.42	4.70
633	0, 0	2	-	100	0.16	58	42	.80	6.50	5.38	7.82	6.30	6.34	4.98	7.78	6.62	6.66	5.42
634	0, 0	2	+	100	0.33	33	67	.80	2.76	5.24	3.12	2.58	2.60	4.92	3.28	2.66	2.72	5.12
635	0, 0	2	-	100	0.33	67	33	.80	8.48	5.54	10.44	8.50	8.66	4.84	10.14	8.66	8.72	5.06
636	0, 0	2	+	100	0.50	25	75	.80	2.06	5.38	2.10	1.58	1.58	5.06	2.24	1.66	1.68	4.94
637	0, 0	2	-	100	0.50	75	25	.80	10.40	5.92	11.78	10.20	10.30	5.60	12.24	10.58	10.66	5.62
638	0, 0	2	0	150	0	75	75	.80	5.76	5.56	6.26	5.42	5.42	5.42	6.86	5.50	5.56	5.86

	D	VR	P	N	Δn	n1	n2	ε	F g	B-F g	F t	F-GG t	F-HF t	B-F t	F g x t	F-GG g x t	F-HF g x t	B-F g x t
639	0, 0	2	+	150	0.16	63	87	.80	4.42	5.70	4.68	3.86	3.88	5.36	4.76	3.70	3.76	5.44
640	0, 0	2	-	150	0.16	87	63	.80	6.14	5.06	8.32	6.96	6.98	5.62	8.16	7.00	7.06	5.62
641	0, 0	2	+	150	0.33	50	100	.80	3.12	5.52	3.38	2.68	2.68	5.84	3.08	2.52	2.52	5.00
642	0, 0	2	-	150	0.33	100	50	.80	7.84	4.54	10.16	8.52	8.60	5.64	10.14	8.58	8.66	5.26
643	0, 0	2	+	150	0.50	37	113	.80	2.08	5.38	2.44	1.88	1.88	5.58	2.28	1.66	1.66	4.94
644	0, 0	2	-	150	0.50	113	37	.80	9.60	5.38	12.66	11.40	11.46	5.52	12.10	10.24	10.38	5.66
645	0, 0	2	0	200	0	100	100	.80	5.22	5.28	6.00	4.86	4.88	4.88	6.30	5.50	5.54	5.42
646	0, 0	2	+	200	0.16	84	116	.80	3.96	5.32	4.64	3.50	3.52	5.04	4.74	3.80	3.82	5.46
647	0, 0	2	-	200	0.16	116	84	.80	6.68	5.46	7.84	6.54	6.60	5.12	8.46	7.18	7.20	5.62
648	0, 0	2	+	200	0.33	67	133	.80	2.94	5.26	3.32	2.64	2.68	5.32	3.64	2.98	3.04	5.56
649	0, 0	2	-	200	0.33	133	67	.80	7.92	4.86	10.52	8.82	8.94	5.30	10.98	9.50	9.58	5.88
650	0, 0	2	+	200	0.50	50	150	.80	2.26	5.58	2.26	1.86	1.86	5.46	2.20	1.62	1.62	4.96
651	0, 0	2	-	200	0.50	150	50	.80	10.20	5.38	12.64	10.96	11.00	5.36	12.68	10.72	10.80	5.66
652	0, 0	5	0	20	0	10	10	.80	5.36	4.30	6.80	5.38	5.84	4.58	6.52	5.38	5.68	4.80
653	0, 0	5	+	20	0.16	8	12	.80	3.10	4.36	3.12	2.44	2.62	4.24	3.24	2.46	2.68	4.18
654	0, 0	5	-	20	0.16	12	8	.80	9.18	5.26	12.08	10.00	10.60	5.62	11.82	9.70	10.28	5.36
655	0, 0	5	+	20	0.33	7	13	.80	2.30	4.58	1.84	1.38	1.56	4.56	2.12	1.54	1.68	4.40
656	0, 0	5	-	20	0.33	13	7	.80	11.82	6.14	15.16	12.44	13.04	5.66	15.10	12.80	13.22	5.78
657	0, 0	5	+	20	0.50	5	15	.80	.88	4.74	.78	.58	.58	4.46	.66	.50	.52	5.02
658	0, 0	5	-	20	0.50	15	5	.80	18.20	9.20	23.04	20.04	20.80	8.14	22.80	19.58	20.22	7.80
659	0, 0	5	0	40	0	20	20	.80	5.56	4.94	6.22	4.98	5.38	4.60	5.94	4.84	4.94	4.54
660	0, 0	5	+	40	0.16	17	23	.80	3.46	4.66	3.64	2.84	2.92	4.64	3.40	2.66	2.74	4.28
661	0, 0	5	-	40	0.16	23	17	.80	7.74	5.22	9.44	8.16	8.36	5.24	9.62	8.12	8.36	5.00
662	0, 0	5	+	40	0.33	13	27	.80	1.18	4.82	1.32	.94	1.00	4.14	1.82	1.42	1.48	4.84
663	0, 0	5	-	40	0.33	27	13	.80	12.56	5.56	15.46	13.40	13.64	5.22	15.58	13.14	13.54	4.96
664	0, 0	5	+	40	0.50	10	30	.80	.64	4.38	.54	.40	.48	4.66	.78	.52	.56	5.28
665	0, 0	5	-	40	0.50	30	10	.80	17.26	6.16	21.20	18.54	18.76	5.88	21.94	19.20	19.72	5.90
666	0, 0	5	0	60	0	30	30	.80	4.80	4.64	6.70	5.58	5.70	5.54	6.26	5.14	5.26	5.10
667	0, 0	5	+	60	0.16	25	35	.80	3.00	4.58	3.74	3.02	3.10	5.02	3.68	2.78	2.90	5.08
668	0, 0	5	-	60	0.16	35	25	.80	7.64	4.90	10.90	9.02	9.22	5.42	10.72	8.88	9.12	5.32
669	0, 0	5	+	60	0.33	20	40	.80	1.72	5.00	1.84	1.36	1.38	4.80	2.02	1.42	1.46	5.40
670	0, 0	5	-	60	0.33	40	20	.80	11.48	4.72	15.88	13.74	13.98	5.42	14.94	12.36	12.50	5.20

	D	VR	P	N	Δn	n1	n2	ε	F g	B-F g	F t	F-GG t	F-HF t	B-F t	F g x t	F-GG g x t	F-HF g x t	B-F g x t
671	0, 0	5	+	60	0.50	15	45	.80	.86	5.24	.62	.42	.44	5.32	.84	.64	.64	5.46
672	0, 0	5	-	60	0.50	45	15	.80	16.38	5.88	22.44	20.12	20.42	5.46	21.28	18.88	19.14	5.46
673	0, 0	5	0	80	0	40	40	.80	4.94	4.76	6.38	5.10	5.26	4.98	6.18	5.02	5.08	5.04
674	0, 0	5	+	80	0.16	34	46	.80	2.88	4.40	3.92	3.10	3.20	5.20	4.00	3.32	3.38	5.50
675	0, 0	5	-	80	0.16	46	34	.80	7.54	4.90	10.42	8.52	8.58	5.26	9.28	8.10	8.20	4.98
676	0, 0	5	+	80	0.33	27	53	.80	1.60	4.76	1.76	1.30	1.30	5.16	1.80	1.34	1.38	4.78
677	0, 0	5	-	80	0.33	53	27	.80	11.10	4.86	15.32	13.54	13.62	5.44	15.42	13.40	13.58	5.08
678	0, 0	5	+	80	0.50	20	60	.80	.74	5.28	.76	.58	.58	5.60	.52	.40	.42	5.40
679	0, 0	5	-	80	0.50	60	20	.80	16.62	5.26	22.54	20.26	20.42	5.76	22.46	19.62	19.90	5.36
680	0, 0	5	0	100	0	50	50	.80	5.04	5.04	6.16	4.72	4.80	4.74	6.14	5.12	5.20	4.96
681	0, 0	5	+	100	0.16	42	58	.80	2.86	4.90	3.10	2.48	2.54	4.84	3.48	2.82	2.88	5.06
682	0, 0	5	-	100	0.16	58	42	.80	8.12	5.14	9.98	8.36	8.50	4.62	10.22	8.70	8.78	5.06
683	0, 0	5	+	100	0.33	33	67	.80	1.30	4.74	1.54	1.14	1.14	4.84	1.80	1.38	1.42	5.30
684	0, 0	5	-	100	0.33	67	33	.80	13.08	5.34	15.96	13.60	13.70	4.80	15.44	13.26	13.38	5.02
685	0, 0	5	+	100	0.50	25	75	.80	.60	4.88	.60	.40	.40	5.12	.54	.38	.40	5.30
686	0, 0	5	-	100	0.50	75	25	.80	18.38	5.68	21.50	19.10	19.26	4.94	21.80	19.74	19.84	5.08
687	0, 0	5	0	150	0	75	75	.80	5.42	5.26	6.52	5.44	5.44	5.50	6.72	5.56	5.60	5.48
688	0, 0	5	+	150	0.16	63	87	.80	3.38	5.18	3.58	2.84	2.88	5.34	3.60	2.74	2.80	5.32
689	0, 0	5	-	150	0.16	87	63	.80	7.40	4.84	10.38	8.86	8.90	5.66	10.08	8.94	8.98	5.30
690	0, 0	5	+	150	0.33	50	100	.80	1.58	5.22	1.62	1.22	1.24	5.50	1.56	1.12	1.14	4.92
691	0, 0	5	-	150	0.33	100	50	.80	11.50	4.56	15.16	13.48	13.58	5.26	15.04	13.36	13.48	5.14
692	0, 0	5	+	150	0.50	37	113	.80	.56	4.96	.48	.34	.34	5.70	.64	.40	.42	4.98
693	0, 0	5	-	150	0.50	113	37	.80	17.12	5.10	21.38	18.92	18.98	5.38	21.74	19.04	19.20	5.26
694	0, 0	5	0	200	0	100	100	.80	5.00	4.82	6.28	5.12	5.18	5.00	6.22	5.28	5.28	5.32
695	0, 0	5	+	200	0.16	84	116	.80	2.70	4.82	3.46	2.76	2.76	4.96	3.38	2.74	2.78	5.04
696	0, 0	5	-	200	0.16	116	84	.80	7.82	5.32	10.20	8.42	8.44	5.08	10.44	9.22	9.24	5.78
697	0, 0	5	+	200	0.33	67	133	.80	1.66	5.10	1.68	1.16	1.16	4.94	1.62	1.26	1.26	5.54
698	0, 0	5	-	200	0.33	133	67	.80	11.84	4.96	16.10	13.90	14.06	5.50	16.16	14.40	14.48	5.74
699	0, 0	5	+	200	0.50	50	150	.80	.56	5.30	.64	.40	.40	5.18	.54	.32	.32	4.82
700	0, 0	5	-	200	0.50	150	50	.80	16.84	5.32	22.64	20.24	20.26	5.70	23.02	20.60	20.68	5.60
701	0, 0	1	0	20	0	10	10	.90	4.34	3.98	5.30	4.44	4.82	4.44	5.80	4.92	5.42	4.84
702	0, 0	1	0	20	0.16	8	12	.90	4.62	4.48	5.14	4.38	4.72	4.32	5.70	4.70	5.14	4.78

	D	VR	P	N	Δn	n1	n2	ε	F g	B-F g	F t	F-GG t	F-HF t	B-F t	F g x t	F-GG g x t	F-HF g x t	B-F g x t
703	0, 0	1	0	20	0.33	7	13	.90	5.22	5.18	5.28	4.52	4.86	4.56	5.46	4.72	5.14	5.02
704	0, 0	1	0	20	0.50	5	15	.90	5.10	7.44	5.32	4.48	4.86	7.06	5.70	4.78	5.04	7.00
705	0, 0	1	0	40	0	20	20	.90	4.96	4.84	5.34	4.82	4.94	4.94	5.34	4.52	4.76	4.88
706	0, 0	1	0	40	0.16	17	23	.90	4.90	4.98	5.56	4.94	5.10	4.84	4.92	4.34	4.54	4.52
707	0, 0	1	0	40	0.33	13	27	.90	4.64	4.94	5.28	4.44	4.66	5.18	5.26	4.68	4.88	5.16
708	0, 0	1	0	40	0.50	10	30	.90	4.48	5.92	5.06	4.36	4.56	5.76	5.48	4.68	4.86	6.46
709	0, 0	1	0	60	0	30	30	.90	4.76	4.72	5.42	4.72	4.90	4.98	5.22	4.64	4.80	4.72
710	0, 0	1	0	60	0.16	25	35	.90	4.80	4.82	5.16	4.66	4.84	4.84	5.34	4.82	4.94	4.82
711	0, 0	1	0	60	0.33	20	40	.90	5.14	5.44	5.02	4.56	4.68	4.62	5.02	4.38	4.60	4.72
712	0, 0	1	0	60	0.50	15	45	.90	4.98	5.90	4.96	4.46	4.52	5.08	5.40	4.86	4.94	5.84
713	0, 0	1	0	80	0	40	40	.90	4.86	4.66	6.06	5.36	5.44	5.34	5.34	4.64	4.76	4.76
714	0, 0	1	0	80	0.16	34	46	.90	4.66	5.00	5.90	5.22	5.28	5.66	5.44	4.86	4.96	5.24
715	0, 0	1	0	80	0.33	27	53	.90	5.02	5.50	5.82	5.30	5.34	5.64	5.16	4.64	4.72	5.04
716	0, 0	1	0	80	0.50	20	60	.90	5.48	5.58	5.54	4.92	5.00	5.64	5.30	4.60	4.78	5.36
717	0, 0	1	0	100	0	50	50	.90	5.24	5.40	5.28	4.62	4.72	4.98	5.14	4.52	4.60	4.56
718	0, 0	1	0	100	0.16	42	58	.90	5.04	5.02	5.52	5.04	5.06	5.24	5.38	4.72	4.82	5.08
719	0, 0	1	0	100	0.33	33	67	.90	5.06	5.38	5.74	4.96	5.04	5.50	5.34	4.80	4.86	5.34
720	0, 0	1	0	100	0.50	25	75	.90	4.96	5.20	5.46	4.86	4.92	5.62	4.92	4.24	4.36	4.96
721	0, 0	1	0	150	0	75	75	.90	5.84	5.68	5.88	5.32	5.32	5.48	6.22	5.48	5.52	5.68
722	0, 0	1	0	150	0.16	63	87	.90	5.92	5.76	5.96	5.34	5.46	5.68	5.50	5.06	5.12	5.32
723	0, 0	1	0	150	0.33	50	100	.90	5.08	5.54	5.92	5.32	5.40	5.64	5.20	4.72	4.80	5.10
724	0, 0	1	0	150	0.50	37	113	.90	5.16	5.64	5.88	5.44	5.52	5.78	5.42	4.84	4.88	5.36
725	0, 0	1	0	200	0	100	100	.90	5.24	5.24	5.64	5.20	5.26	5.30	5.62	5.14	5.18	5.20
726	0, 0	1	0	200	0.16	84	116	.90	5.08	5.18	5.86	5.18	5.22	5.20	5.72	5.20	5.20	5.32
727	0, 0	1	0	200	0.33	67	133	.90	5.34	5.42	6.02	5.42	5.48	5.64	5.56	5.04	5.08	5.02
728	0, 0	1	0	200	0.50	50	150	.90	5.00	5.54	5.80	5.12	5.12	5.68	5.34	4.64	4.64	5.18
729	0, 0	1.5	0	20	0	10	10	.90	4.28	4.00	5.52	4.66	4.98	4.48	5.74	4.82	5.26	4.56
730	0, 0	1.5	+	20	0.16	8	12	.90	3.84	4.50	4.24	3.56	3.74	4.22	4.44	3.72	4.02	4.74
731	0, 0	1.5	-	20	0.16	12	8	.90	5.78	5.18	6.90	5.90	6.36	4.78	6.44	5.48	5.88	4.40
732	0, 0	1.5	+	20	0.33	7	13	.90	4.04	5.36	3.68	3.10	3.30	4.54	4.26	3.52	3.94	5.20
733	0, 0	1.5	-	20	0.33	13	7	.90	6.54	5.86	7.12	6.28	6.68	5.34	7.72	6.42	6.86	5.34
734	0, 0	1.5	+	20	0.50	5	15	.90	3.12	6.60	2.78	2.20	2.46	6.22	3.26	2.70	2.94	6.40

	D	VR	P	N	Δn	n1	n2	ε	F g	B-F g	F t	F-GG t	F-HF t	B-F t	F g x t	F-GG g x t	F-HF g x t	B-F g x t
735	0, 0	1.5	-	20	0.50	15	5	.90	7.76	8.14	9.20	7.98	8.56	7.18	9.58	8.28	8.98	7.70
736	0, 0	1.5	0	40	0	20	20	.90	5.16	4.94	5.72	5.02	5.22	4.88	5.34	4.66	4.86	4.66
737	0, 0	1.5	+	40	0.16	17	23	.90	4.48	4.94	4.60	4.10	4.22	4.76	4.36	3.72	3.90	4.40
738	0, 0	1.5	-	40	0.16	23	17	.90	5.64	4.88	6.96	6.06	6.38	5.14	6.08	5.50	5.60	4.86
739	0, 0	1.5	+	40	0.33	13	27	.90	3.22	4.92	3.54	3.10	3.32	4.86	3.68	3.20	3.28	4.86
740	0, 0	1.5	-	40	0.33	27	13	.90	6.60	5.60	8.32	7.36	7.64	6.06	7.20	6.38	6.54	5.10
741	0, 0	1.5	+	40	0.50	10	30	.90	2.76	5.42	2.66	2.34	2.36	5.84	2.96	2.60	2.70	6.30
742	0, 0	1.5	-	40	0.50	30	10	.90	7.30	6.72	8.98	7.84	8.22	6.40	8.46	7.64	7.84	6.24
743	0, 0	1.5	0	60	0	30	30	.90	4.54	4.62	5.68	4.84	5.00	5.06	5.24	4.76	4.86	4.90
744	0, 0	1.5	+	60	0.16	25	35	.90	4.08	4.66	4.46	3.88	4.04	4.94	4.68	4.00	4.10	4.96
745	0, 0	1.5	-	60	0.16	35	25	.90	5.30	4.58	6.70	5.84	6.06	5.22	6.44	5.92	6.06	5.46
746	0, 0	1.5	+	60	0.33	20	40	.90	3.84	5.38	3.38	3.08	3.14	4.62	3.58	3.20	3.28	4.78
747	0, 0	1.5	-	60	0.33	40	20	.90	6.22	5.10	8.22	7.36	7.54	5.20	7.40	6.64	6.74	5.14
748	0, 0	1.5	+	60	0.50	15	45	.90	2.98	5.50	2.92	2.58	2.72	4.74	3.06	2.42	2.52	5.52
749	0, 0	1.5	-	60	0.50	45	15	.90	7.18	5.88	9.20	8.42	8.62	5.70	8.74	7.84	8.14	5.90
750	0, 0	1.5	0	80	0	40	40	.90	4.62	4.66	6.12	5.40	5.54	5.34	5.36	4.96	5.02	4.86
751	0, 0	1.5	+	80	0.16	34	46	.90	4.00	4.78	5.28	4.60	4.66	5.64	4.74	4.30	4.38	5.20
752	0, 0	1.5	-	80	0.16	46	34	.90	5.96	4.76	7.12	6.30	6.48	5.48	6.40	5.76	5.86	5.10
753	0, 0	1.5	+	80	0.33	27	53	.90	3.26	5.20	4.22	3.60	3.66	5.56	3.68	3.22	3.26	4.84
754	0, 0	1.5	-	80	0.33	53	27	.90	6.04	4.74	8.56	7.70	7.92	5.42	7.82	7.14	7.24	5.34
755	0, 0	1.5	+	80	0.50	20	60	.90	3.14	5.84	3.34	2.88	2.96	5.78	2.90	2.52	2.58	5.46
756	0, 0	1.5	-	80	0.50	60	20	.90	7.76	5.54	9.40	8.60	8.78	5.86	9.32	8.46	8.60	5.90
757	0, 0	1.5	0	100	0	50	50	.90	5.14	5.16	5.12	4.64	4.64	4.78	5.20	4.68	4.72	4.66
758	0, 0	1.5	+	100	0.16	42	58	.90	4.24	5.06	4.68	4.10	4.14	4.98	4.54	3.98	4.00	4.92
759	0, 0	1.5	-	100	0.16	58	42	.90	6.10	5.52	6.02	5.46	5.50	4.74	6.60	5.96	6.04	5.36
760	0, 0	1.5	+	100	0.33	33	67	.90	3.68	5.08	3.82	3.36	3.42	5.24	3.74	3.34	3.44	5.10
761	0, 0	1.5	-	100	0.33	67	33	.90	6.92	5.34	7.50	6.76	7.00	4.76	7.94	7.40	7.44	5.00
762	0, 0	1.5	+	100	0.50	25	75	.90	2.92	5.16	2.98	2.74	2.74	5.54	2.58	2.16	2.22	4.88
763	0, 0	1.5	-	100	0.50	75	25	.90	8.10	5.90	8.44	7.70	7.82	5.20	9.24	8.34	8.42	5.24
764	0, 0	1.5	0	150	0	75	75	.90	6.12	5.88	5.80	5.34	5.40	5.40	5.92	5.36	5.38	5.44
765	0, 0	1.5	+	150	0.16	63	87	.90	5.02	5.74	4.90	4.40	4.40	5.52	4.58	4.00	4.06	5.14
766	0, 0	1.5	-	150	0.16	87	63	.90	6.12	5.36	6.72	6.10	6.18	5.34	6.98	6.20	6.28	5.24

	D	VR	P	N	Δn	n1	n2	ε	F g	B-F g	F t	F-GG t	F-HF t	B-F t	F g x t	F-GG g x t	F-HF g x t	B-F g x t
767	0, 0	1.5	+	150	0.33	50	100	.90	3.76	5.24	4.18	3.74	3.80	5.78	3.80	3.34	3.42	5.00
768	0, 0	1.5	-	150	0.33	100	50	.90	6.32	4.98	7.86	7.12	7.22	5.06	7.54	6.74	6.88	4.96
769	0, 0	1.5	+	150	0.50	37	113	.90	3.02	5.68	3.26	2.82	2.86	5.84	3.06	2.86	2.88	5.46
770	0, 0	1.5	-	150	0.50	113	37	.90	7.54	5.46	9.08	8.10	8.16	5.02	9.08	8.38	8.44	5.24
771	0, 0	1.5	0	200	0	100	100	.90	5.14	4.98	5.82	5.32	5.40	5.32	5.58	5.04	5.14	5.18
772	0, 0	1.5	+	200	0.16	84	116	.90	4.34	5.42	4.94	4.54	4.54	5.34	4.86	4.40	4.42	5.34
773	0, 0	1.5	-	200	0.16	116	84	.90	5.96	5.14	6.84	6.22	6.26	5.70	6.72	6.06	6.14	5.36
774	0, 0	1.5	+	200	0.33	67	133	.90	3.94	5.42	4.22	3.70	3.78	5.50	3.76	3.34	3.36	5.24
775	0, 0	1.5	-	200	0.33	133	67	.90	7.08	5.54	7.92	7.10	7.14	5.36	7.86	7.00	7.06	5.38
776	0, 0	1.5	+	200	0.50	50	150	.90	3.08	5.40	3.32	2.92	2.96	5.72	2.76	2.34	2.42	5.18
777	0, 0	1.5	-	200	0.50	150	50	.90	7.90	5.44	9.58	8.78	8.86	5.44	9.52	8.70	8.78	5.30
778	0, 0	2	0	20	0	10	10	.90	4.46	3.92	5.66	4.62	5.08	4.54	5.68	4.76	5.20	4.50
779	0, 0	2	+	20	0.16	8	12	.90	3.64	4.40	3.62	3.02	3.30	4.30	3.80	3.04	3.36	4.62
780	0, 0	2	-	20	0.16	12	8	.90	6.58	5.18	7.90	6.92	7.42	4.78	7.40	6.50	6.80	4.58
781	0, 0	2	+	20	0.33	7	13	.90	3.30	5.22	2.82	2.50	2.66	4.50	3.34	2.80	3.02	5.16
782	0, 0	2	-	20	0.33	13	7	.90	7.42	6.04	8.72	7.44	7.96	5.60	9.14	7.72	8.34	5.36
783	0, 0	2	+	20	0.50	5	15	.90	2.22	6.26	1.72	1.54	1.62	5.72	2.08	1.60	1.82	5.92
784	0, 0	2	-	20	0.50	15	5	.90	10.00	8.20	12.00	10.78	11.20	7.42	12.38	11.14	11.54	7.76
785	0, 0	2	0	40	0	20	20	.90	5.04	4.82	5.80	5.08	5.26	5.02	5.32	4.58	4.80	4.58
786	0, 0	2	+	40	0.16	17	23	.90	4.14	4.84	4.02	3.58	3.68	4.68	4.02	3.40	3.58	4.28
787	0, 0	2	-	40	0.16	23	17	.90	6.04	5.02	7.74	6.98	7.22	5.12	7.04	6.20	6.38	5.12
788	0, 0	2	+	40	0.33	13	27	.90	2.48	4.72	2.68	2.26	2.34	4.86	2.74	2.26	2.46	4.96
789	0, 0	2	-	40	0.33	27	13	.90	7.90	5.70	10.46	9.38	9.72	6.16	8.80	8.12	8.36	5.20
790	0, 0	2	+	40	0.50	10	30	.90	1.68	5.26	1.64	1.46	1.56	5.66	1.84	1.52	1.62	5.90
791	0, 0	2	-	40	0.50	30	10	.90	9.36	6.72	11.86	10.72	11.08	6.50	11.88	10.88	11.08	6.12
792	0, 0	2	0	60	0	30	30	.90	4.64	4.40	5.72	4.88	4.98	5.14	5.44	4.88	5.08	4.90
793	0, 0	2	+	60	0.16	25	35	.90	3.74	4.48	3.96	3.52	3.60	4.78	4.20	3.72	3.84	4.78
794	0, 0	2	-	60	0.16	35	25	.90	5.86	4.50	7.44	6.58	6.80	5.28	7.48	6.72	6.82	5.62
795	0, 0	2	+	60	0.33	20	40	.90	3.04	5.32	2.60	2.34	2.42	4.56	2.82	2.52	2.62	4.92
796	0, 0	2	-	60	0.33	40	20	.90	7.62	5.12	9.92	9.12	9.28	5.50	9.22	8.46	8.70	5.16
797	0, 0	2	+	60	0.50	15	45	.90	2.10	5.38	2.02	1.66	1.74	4.54	1.74	1.50	1.54	5.28
798	0, 0	2	-	60	0.50	45	15	.90	9.50	5.90	12.14	11.18	11.40	5.70	11.86	10.94	11.08	5.98

	D	VR	P	N	Δn	n1	n2	ε	F g	B-F g	F t	F-GG t	F-HF t	B-F t	F g x t	F-GG g x t	F-HF g x t	B-F g x t
799	0, 0	2	0	80	0	40	40	.90	4.60	4.60	6.06	5.52	5.60	5.46	5.46	5.02	5.10	5.12
800	0, 0	2	+	80	0.16	34	46	.90	3.76	4.74	4.62	3.96	4.04	5.60	4.46	3.96	4.08	5.48
801	0, 0	2	-	80	0.16	46	34	.90	6.18	4.86	7.78	7.06	7.14	5.56	7.04	6.32	6.44	5.04
802	0, 0	2	+	80	0.33	27	53	.90	2.72	5.02	3.10	2.80	2.84	5.54	2.70	2.36	2.44	4.56
803	0, 0	2	-	80	0.33	53	27	.90	7.34	4.82	10.30	9.20	9.36	5.28	9.42	8.66	8.92	5.50
804	0, 0	2	+	80	0.50	20	60	.90	2.06	5.32	2.20	1.90	1.92	5.32	1.72	1.44	1.50	5.10
805	0, 0	2	-	80	0.50	60	20	.90	9.64	5.54	12.96	11.88	12.04	6.00	12.26	11.40	11.60	5.82
806	0, 0	2	0	100	0	50	50	.90	5.18	4.96	5.30	4.62	4.68	4.66	5.26	4.64	4.76	4.72
807	0, 0	2	+	100	0.16	42	58	.90	3.62	5.14	4.24	3.76	3.82	4.86	4.08	3.68	3.70	4.98
808	0, 0	2	-	100	0.16	58	42	.90	6.76	5.68	7.00	6.20	6.32	4.84	7.34	6.70	6.78	5.24
809	0, 0	2	+	100	0.33	33	67	.90	2.88	5.12	3.02	2.60	2.64	5.14	3.00	2.62	2.66	5.06
810	0, 0	2	-	100	0.33	67	33	.90	8.22	5.68	9.40	8.68	8.76	5.32	9.74	8.94	9.06	5.58
811	0, 0	2	+	100	0.50	25	75	.90	1.94	4.96	1.96	1.60	1.62	5.20	1.50	1.30	1.34	4.96
812	0, 0	2	-	100	0.50	75	25	.90	10.52	5.94	11.66	10.64	10.78	5.02	12.04	11.24	11.32	5.42
813	0, 0	2	0	150	0	75	75	.90	6.04	6.02	5.88	5.26	5.30	5.42	5.76	5.38	5.40	5.44
814	0, 0	2	+	150	0.16	63	87	.90	4.44	5.60	4.26	3.94	3.98	5.54	3.86	3.44	3.54	5.10
815	0, 0	2	-	150	0.16	87	63	.90	6.64	5.10	7.38	6.68	6.74	5.22	7.76	7.06	7.14	5.26
816	0, 0	2	+	150	0.33	50	100	.90	2.90	5.10	3.08	2.68	2.70	5.66	2.88	2.40	2.44	4.90
817	0, 0	2	-	150	0.33	100	50	.90	7.50	4.84	9.60	8.76	8.82	5.00	9.06	8.48	8.62	4.86
818	0, 0	2	+	150	0.50	37	113	.90	1.96	5.38	2.08	1.84	1.88	5.98	2.06	1.72	1.74	5.46
819	0, 0	2	-	150	0.50	113	37	.90	9.76	5.46	11.66	10.76	10.82	4.88	11.56	10.72	10.80	5.18
820	0, 0	2	0	200	0	100	100	.90	4.98	5.12	5.82	5.54	5.54	5.70	5.58	5.00	5.04	5.18
821	0, 0	2	+	200	0.16	84	116	.90	3.88	5.24	4.54	4.16	4.18	5.36	4.46	3.98	4.00	5.34
822	0, 0	2	-	200	0.16	116	84	.90	6.52	5.22	7.52	6.78	6.82	5.66	7.32	6.66	6.68	5.44
823	0, 0	2	+	200	0.33	67	133	.90	3.24	5.32	3.30	2.96	3.00	5.28	2.92	2.68	2.72	5.08
824	0, 0	2	-	200	0.33	133	67	.90	8.28	5.50	9.86	9.00	9.08	5.44	9.98	9.00	9.06	5.46
825	0, 0	2	+	200	0.50	50	150	.90	2.14	5.28	2.28	2.02	2.02	5.78	1.54	1.22	1.24	5.02
826	0, 0	2	-	200	0.50	150	50	.90	10.48	5.68	12.74	11.78	11.88	5.62	12.90	11.76	11.86	5.26
827	0, 0	5	0	20	0	10	10	.90	5.08	4.24	6.34	5.26	5.62	4.78	6.10	5.08	5.58	4.18
828	0, 0	5	+	20	0.16	8	12	.90	3.32	4.68	2.86	2.32	2.56	4.26	2.72	2.14	2.30	4.04
829	0, 0	5	-	20	0.16	12	8	.90	9.68	5.50	11.36	9.78	10.32	4.70	11.06	9.54	10.10	4.80
830	0, 0	5	+	20	0.33	7	13	.90	2.26	4.86	1.82	1.34	1.54	4.34	1.86	1.48	1.62	4.74

	D	VR	P	N	Δn	n1	n2	ε	F g	B-F g	F t	F-GG t	F-HF t	B-F t	F g x t	F-GG g x t	F-HF g x t	B-F g x t
831	0, 0	5	-	20	0.33	13	7	.90	11.98	6.18	14.30	12.38	13.08	5.80	14.22	12.66	13.22	5.14
832	0, 0	5	+	20	0.50	5	15	.90	.82	5.24	.54	.44	.48	4.44	.68	.44	.54	4.82
833	0, 0	5	-	20	0.50	15	5	.90	18.24	8.80	23.82	20.92	21.98	7.30	23.58	21.30	22.20	7.44
834	0, 0	5	0	40	0	20	20	.90	5.06	4.54	5.98	5.14	5.44	4.94	5.90	5.04	5.38	4.48
835	0, 0	5	+	40	0.16	17	23	.90	3.38	4.62	2.96	2.60	2.70	4.50	2.74	2.32	2.42	4.38
836	0, 0	5	-	40	0.16	23	17	.90	7.34	4.96	9.58	8.52	8.76	5.26	9.10	8.42	8.64	5.00
837	0, 0	5	+	40	0.33	13	27	.90	1.18	4.32	1.18	.98	1.04	4.60	1.16	.94	.96	4.32
838	0, 0	5	-	40	0.33	27	13	.90	12.46	5.46	16.08	14.94	15.28	5.70	15.40	14.20	14.56	5.40
839	0, 0	5	+	40	0.50	10	30	.90	.62	4.48	.36	.28	.28	5.04	.38	.30	.32	5.04
840	0, 0	5	-	40	0.50	30	10	.90	16.74	6.32	22.32	20.68	21.06	5.58	22.14	20.72	21.02	5.38
841	0, 0	5	0	60	0	30	30	.90	4.54	4.12	6.02	5.26	5.36	5.02	5.62	5.04	5.22	4.78
842	0, 0	5	+	60	0.16	25	35	.90	2.96	4.68	3.10	2.66	2.70	4.98	3.28	2.74	2.84	5.30
843	0, 0	5	-	60	0.16	35	25	.90	7.74	4.42	9.92	8.92	9.16	5.48	9.96	9.00	9.32	5.34
844	0, 0	5	+	60	0.33	20	40	.90	1.48	4.92	1.32	1.10	1.16	4.92	1.44	1.24	1.28	5.10
845	0, 0	5	-	60	0.33	40	20	.90	11.66	4.92	15.80	14.56	14.88	5.40	15.08	13.92	14.16	4.98
846	0, 0	5	+	60	0.50	15	45	.90	.72	5.02	.54	.42	.44	4.70	.56	.50	.54	5.18
847	0, 0	5	-	60	0.50	45	15	.90	16.68	5.70	22.98	21.52	21.78	5.14	23.04	21.00	21.48	5.58
848	0, 0	5	0	80	0	40	40	.90	5.02	4.64	5.86	5.38	5.44	5.08	5.50	5.00	5.12	5.20
849	0, 0	5	+	80	0.16	34	46	.90	2.98	4.60	3.66	3.14	3.18	5.60	3.56	3.24	3.34	5.50
850	0, 0	5	-	80	0.16	46	34	.90	7.54	4.70	9.42	8.68	8.82	5.44	9.26	8.24	8.28	4.92
851	0, 0	5	+	80	0.33	27	53	.90	1.40	4.48	1.72	1.30	1.32	5.58	1.32	1.08	1.12	4.58
852	0, 0	5	-	80	0.33	53	27	.90	11.22	4.98	15.68	14.68	14.86	5.36	15.32	14.04	14.30	5.34
853	0, 0	5	+	80	0.50	20	60	.90	.62	5.08	.56	.46	.46	5.40	.44	.40	.40	5.22
854	0, 0	5	-	80	0.50	60	20	.90	16.24	5.84	23.36	21.94	22.12	5.76	22.68	21.24	21.44	5.36
855	0, 0	5	0	100	0	50	50	.90	5.24	5.10	5.48	4.82	4.88	4.80	5.42	4.90	4.94	4.86
856	0, 0	5	+	100	0.16	42	58	.90	2.70	4.48	2.98	2.60	2.68	4.94	3.00	2.66	2.74	5.24
857	0, 0	5	-	100	0.16	58	42	.90	8.20	5.36	9.30	8.46	8.54	4.86	9.64	8.76	8.92	5.36
858	0, 0	5	+	100	0.33	33	67	.90	1.02	4.86	1.24	1.06	1.14	4.60	1.36	1.16	1.16	5.20
859	0, 0	5	-	100	0.33	67	33	.90	12.46	5.60	15.44	14.18	14.30	5.18	15.80	14.56	14.68	5.36
860	0, 0	5	+	100	0.50	25	75	.90	.56	4.46	.38	.30	.34	5.02	.42	.36	.38	5.04
861	0, 0	5	-	100	0.50	75	25	.90	17.54	5.90	21.44	20.16	20.38	5.06	22.40	20.74	20.92	5.36
862	0, 0	5	0	150	0	75	75	.90	5.80	5.62	5.82	5.26	5.28	5.32	5.86	5.12	5.22	5.38

	D	VR	P	N	Δn	n1	n2	ε	F g	B-F g	F t	F-GG t	F-HF t	B-F t	F g x t	F-GG g x t	F-HF g x t	B-F g x t
863	0, 0	5	+	150	0.16	63	87	.90	3.48	5.46	3.22	2.74	2.78	5.50	2.76	2.40	2.40	5.08
864	0, 0	5	-	150	0.16	87	63	.90	8.14	5.10	9.52	8.82	8.86	4.96	9.66	8.72	8.78	5.26
865	0, 0	5	+	150	0.33	50	100	.90	1.52	5.02	1.40	1.08	1.14	5.46	1.16	1.00	1.02	4.92
866	0, 0	5	-	150	0.33	100	50	.90	11.08	4.68	15.14	14.18	14.32	4.84	14.70	13.64	13.70	4.74
867	0, 0	5	+	150	0.50	37	113	.90	.48	5.14	.48	.38	.38	5.84	.46	.44	.44	5.10
868	0, 0	5	-	150	0.50	113	37	.90	16.66	5.28	22.74	21.42	21.50	4.72	21.40	20.30	20.42	5.12
869	0, 0	5	0	200	0	100	100	.90	5.04	5.00	6.02	5.36	5.38	5.54	5.54	4.90	4.94	5.10
870	0, 0	5	+	200	0.16	84	116	.90	2.84	5.06	3.18	2.88	2.88	5.34	3.24	2.78	2.80	5.34
871	0, 0	5	-	200	0.16	116	84	.90	8.26	5.08	9.36	8.62	8.66	5.56	9.36	8.64	8.66	5.68
872	0, 0	5	+	200	0.33	67	133	.90	1.80	4.98	1.44	1.16	1.22	5.48	1.24	1.06	1.08	5.12
873	0, 0	5	-	200	0.33	133	67	.90	12.36	5.30	15.40	14.36	14.44	5.72	15.74	14.74	14.92	5.54
874	0, 0	5	+	200	0.50	50	150	.90	.54	4.84	.44	.32	.32	5.48	.16	.14	.14	4.70
875	0, 0	5	-	200	0.50	150	50	.90	17.42	5.32	23.20	21.94	22.02	5.62	23.06	21.94	22.06	5.44
876	0, 0	1	0	20	0	10	10	1	4.02	3.94	4.74	4.20	4.62	4.18	5.20	4.50	4.94	4.52
877	0, 0	1	0	20	0.16	8	12	1	4.64	4.62	4.66	4.26	4.58	3.96	4.98	4.48	4.76	4.42
878	0, 0	1	0	20	0.33	7	13	1	5.00	5.08	4.64	4.18	4.50	4.18	4.90	4.52	4.68	5.02
879	0, 0	1	0	20	0.50	5	15	1	5.10	7.28	4.86	4.44	4.64	6.92	5.16	4.62	5.02	7.10
880	0, 0	1	0	40	0	20	20	1	5.08	4.98	4.78	4.62	4.72	4.42	4.86	4.68	4.76	4.66
881	0, 0	1	0	40	0.16	17	23	1	5.12	5.18	4.82	4.56	4.78	4.80	4.56	4.40	4.46	4.60
882	0, 0	1	0	40	0.33	13	27	1	4.38	4.96	4.70	4.54	4.64	4.94	5.26	4.98	5.12	5.54
883	0, 0	1	0	40	0.50	10	30	1	4.24	5.64	4.68	4.52	4.64	5.84	5.04	4.78	4.98	6.62
884	0, 0	1	0	60	0	30	30	1	5.12	5.10	5.20	5.08	5.16	5.06	5.14	4.88	5.04	4.98
885	0, 0	1	0	60	0.16	25	35	1	5.06	5.10	5.04	4.90	4.98	4.88	4.92	4.76	4.84	4.84
886	0, 0	1	0	60	0.33	20	40	1	5.26	5.58	4.78	4.62	4.70	5.18	5.32	5.10	5.22	5.28
887	0, 0	1	0	60	0.50	15	45	1	4.96	6.14	4.50	4.46	4.50	5.20	4.98	4.76	4.92	5.78
888	0, 0	1	0	80	0	40	40	1	4.90	4.94	5.44	5.26	5.40	5.22	4.30	4.26	4.28	4.40
889	0, 0	1	0	80	0.16	34	46	1	4.88	5.12	5.60	5.42	5.50	5.64	5.32	5.12	5.18	5.32
890	0, 0	1	0	80	0.33	27	53	1	5.22	5.22	5.20	5.08	5.14	5.56	4.52	4.34	4.48	4.72
891	0, 0	1	0	80	0.50	20	60	1	5.42	6.02	5.26	5.18	5.22	6.06	4.76	4.60	4.70	5.46
892	0, 0	1	0	100	0	50	50	1	5.26	5.44	5.12	5.10	5.12	5.22	4.88	4.84	4.86	5.02
893	0, 0	1	0	100	0.16	42	58	1	4.90	4.96	5.56	5.46	5.52	5.48	4.92	4.82	4.88	5.08
894	0, 0	1	0	100	0.33	33	67	1	5.08	5.40	5.10	4.98	5.04	5.26	5.08	5.02	5.04	5.36

	D	VR	P	N	Δn	n1	n2	ε	F g	B-F g	F t	F-GG t	F-HF t	B-F t	F g x t	F-GG g x t	F-HF g x t	B-F g x t
895	0, 0	1	0	100	0.50	25	75	1	5.16	5.52	4.80	4.70	4.80	5.20	4.60	4.52	4.54	4.80
896	0, 0	1	0	150	0	75	75	1	5.74	5.66	5.20	5.14	5.18	5.12	5.72	5.68	5.68	5.98
897	0, 0	1	0	150	0.16	63	87	1	5.64	5.66	5.36	5.20	5.26	5.54	5.18	5.14	5.18	5.12
898	0, 0	1	0	150	0.33	50	100	1	5.60	5.66	5.30	5.24	5.28	5.54	5.08	5.02	5.06	5.28
899	0, 0	1	0	150	0.50	37	113	1	5.20	5.76	5.34	5.22	5.26	5.62	4.96	4.86	4.94	5.28
900	0, 0	1	0	200	0	100	100	1	5.18	5.18	5.02	5.02	5.02	4.98	5.56	5.50	5.56	5.50
901	0, 0	1	0	200	0.16	84	116	1	5.28	5.28	5.26	5.22	5.26	5.16	5.58	5.50	5.54	5.54
902	0, 0	1	0	200	0.33	67	133	1	5.64	5.64	5.44	5.36	5.42	5.50	5.74	5.70	5.70	5.58
903	0, 0	1	0	200	0.50	50	150	1	5.12	5.56	5.06	5.00	5.04	5.34	5.10	5.06	5.08	5.14
904	0, 0	1.5	0	20	0	10	10	1	4.30	3.94	4.90	4.44	4.74	4.10	5.38	4.68	5.18	4.26
905	0, 0	1.5	+	20	0.16	8	12	1	3.98	4.56	3.66	3.18	3.44	3.98	4.26	3.82	4.10	4.76
906	0, 0	1.5	-	20	0.16	12	8	1	5.74	5.00	6.02	5.46	5.76	4.58	6.60	5.78	6.20	4.36
907	0, 0	1.5	+	20	0.33	7	13	1	3.92	5.26	3.16	2.84	3.10	4.54	3.40	3.08	3.22	5.08
908	0, 0	1.5	-	20	0.33	13	7	1	6.34	5.64	6.76	5.92	6.60	5.54	7.04	6.48	6.78	5.16
909	0, 0	1.5	+	20	0.50	5	15	1	3.08	6.30	2.44	2.24	2.36	6.06	2.66	2.32	2.54	6.32
910	0, 0	1.5	-	20	0.50	15	5	1	7.76	8.22	8.74	7.84	8.40	7.88	9.08	8.20	8.80	7.36
911	0, 0	1.5	0	40	0	20	20	1	5.00	4.98	4.80	4.64	4.74	4.44	4.92	4.62	4.76	4.60
912	0, 0	1.5	+	40	0.16	17	23	1	4.58	5.00	3.98	3.70	3.90	4.48	3.78	3.62	3.70	4.40
913	0, 0	1.5	-	40	0.16	23	17	1	5.84	5.24	5.92	5.62	5.78	4.96	5.76	5.50	5.68	5.04
914	0, 0	1.5	+	40	0.33	13	27	1	3.22	4.82	3.12	2.86	3.04	4.76	3.24	3.06	3.16	5.06
915	0, 0	1.5	-	40	0.33	27	13	1	6.86	5.52	7.52	7.30	7.42	5.20	6.60	6.26	6.52	4.66
916	0, 0	1.5	+	40	0.50	10	30	1	2.48	5.26	2.40	2.22	2.36	5.64	2.54	2.38	2.42	6.30
917	0, 0	1.5	-	40	0.50	30	10	1	7.34	6.66	8.44	8.04	8.28	6.30	8.00	7.54	7.82	5.88
918	0, 0	1.5	0	60	0	30	30	1	4.98	4.98	5.40	5.26	5.38	5.30	5.06	4.88	5.00	4.94
919	0, 0	1.5	+	60	0.16	25	35	1	4.22	4.94	4.30	4.22	4.26	5.20	4.04	3.84	4.00	4.84
920	0, 0	1.5	-	60	0.16	35	25	1	5.36	4.74	6.46	6.28	6.42	5.60	6.54	6.34	6.48	5.58
921	0, 0	1.5	+	60	0.33	20	40	1	3.88	5.38	3.42	3.30	3.38	5.02	3.50	3.36	3.44	5.22
922	0, 0	1.5	-	60	0.33	40	20	1	6.12	4.88	7.52	7.34	7.42	5.44	6.88	6.66	6.82	4.82
923	0, 0	1.5	+	60	0.50	15	45	1	3.18	5.74	2.58	2.48	2.54	5.20	2.72	2.60	2.70	5.66
924	0, 0	1.5	-	60	0.50	45	15	1	7.32	5.58	9.10	8.84	9.02	5.88	8.18	8.10	8.18	5.56
925	0, 0	1.5	0	80	0	40	40	1	4.80	4.68	5.46	5.38	5.42	5.24	4.56	4.44	4.48	4.34
926	0, 0	1.5	+	80	0.16	34	46	1	4.16	4.84	4.60	4.50	4.56	5.56	4.46	4.34	4.42	5.64

	D	VR	P	N	Δn	n1	n2	ε	F g	B-F g	F t	F-GG t	F-HF t	B-F t	F g x t	F-GG g x t	F-HF g x t	B-F g x t
927	0, 0	1.5	-	80	0.16	46	34	1	5.66	4.76	6.58	6.46	6.54	5.34	5.74	5.56	5.68	4.70
928	0, 0	1.5	+	80	0.33	27	53	1	3.48	5.24	3.52	3.48	3.52	5.52	3.06	2.92	3.02	4.64
929	0, 0	1.5	-	80	0.33	53	27	1	6.10	4.84	8.12	7.80	8.02	5.58	7.42	7.22	7.34	5.16
930	0, 0	1.5	+	80	0.50	20	60	1	3.10	5.74	2.64	2.54	2.58	5.78	2.38	2.20	2.30	5.46
931	0, 0	1.5	-	80	0.50	60	20	1	7.52	5.42	9.58	9.26	9.42	5.68	9.10	8.96	9.06	5.70
932	0, 0	1.5	0	100	0	50	50	1	5.26	5.30	5.06	5.00	5.04	5.10	4.94	4.86	4.92	5.00
933	0, 0	1.5	+	100	0.16	42	58	1	4.14	5.24	4.38	4.34	4.38	5.44	4.16	4.08	4.12	5.22
934	0, 0	1.5	-	100	0.16	58	42	1	6.32	5.60	6.20	6.08	6.14	5.06	6.18	6.06	6.12	5.06
935	0, 0	1.5	+	100	0.33	33	67	1	3.58	5.10	3.30	3.22	3.26	5.26	3.40	3.30	3.38	5.54
936	0, 0	1.5	-	100	0.33	67	33	1	6.84	5.40	7.44	7.30	7.40	4.94	7.24	7.08	7.18	4.76
937	0, 0	1.5	+	100	0.50	25	75	1	2.96	5.24	2.46	2.44	2.46	5.22	2.40	2.28	2.36	4.90
938	0, 0	1.5	-	100	0.50	75	25	1	8.00	5.80	8.28	8.18	8.24	5.18	8.74	8.62	8.68	5.24
939	0, 0	1.5	0	150	0	75	75	1	5.94	5.88	5.06	4.96	5.04	4.98	5.48	5.40	5.44	5.86
940	0, 0	1.5	+	150	0.16	63	87	1	5.08	5.58	4.10	4.02	4.08	5.36	4.12	4.06	4.12	4.94
941	0, 0	1.5	-	150	0.16	87	63	1	5.82	4.94	6.24	6.22	6.24	5.04	6.42	6.40	6.42	5.12
942	0, 0	1.5	+	150	0.33	50	100	1	3.90	5.42	3.28	3.24	3.26	5.48	3.46	3.44	3.46	5.20
943	0, 0	1.5	-	150	0.33	100	50	1	6.40	4.82	7.34	7.26	7.32	5.14	7.34	7.26	7.34	4.88
944	0, 0	1.5	+	150	0.50	37	113	1	3.02	5.72	2.74	2.66	2.72	5.74	2.62	2.60	2.60	5.28
945	0, 0	1.5	-	150	0.50	113	37	1	7.46	5.14	8.52	8.48	8.50	5.02	9.30	9.22	9.26	5.30
946	0, 0	1.5	0	200	0	100	100	1	5.12	5.30	4.96	4.94	4.94	5.04	5.70	5.66	5.70	5.46
947	0, 0	1.5	+	200	0.16	84	116	1	4.46	5.22	4.36	4.30	4.36	5.16	4.34	4.28	4.34	5.30
948	0, 0	1.5	-	200	0.16	116	84	1	6.32	5.30	6.06	6.00	6.04	4.66	6.64	6.58	6.64	5.10
949	0, 0	1.5	+	200	0.33	67	133	1	3.92	5.50	3.62	3.60	3.62	5.38	3.70	3.60	3.68	5.80
950	0, 0	1.5	-	200	0.33	133	67	1	6.60	5.04	7.44	7.30	7.36	4.90	7.52	7.46	7.52	5.30
951	0, 0	1.5	+	200	0.50	50	150	1	3.26	5.36	2.74	2.74	2.74	5.28	2.20	2.12	2.16	5.10
952	0, 0	1.5	-	200	0.50	150	50	1	7.96	5.42	9.04	9.02	9.04	5.46	9.14	9.08	9.10	5.46
953	0, 0	2	0	20	0	10	10	1	4.64	4.04	5.12	4.54	4.80	4.18	5.26	4.74	5.14	4.36
954	0, 0	2	+	20	0.16	8	12	1	3.64	4.44	3.16	2.84	3.06	4.08	3.54	3.10	3.36	4.58
955	0, 0	2	-	20	0.16	12	8	1	6.78	5.14	7.34	6.50	7.00	4.74	7.58	6.90	7.34	4.46
956	0, 0	2	+	20	0.33	7	13	1	3.48	5.02	2.40	2.08	2.24	4.30	2.66	2.42	2.66	4.82
957	0, 0	2	-	20	0.33	13	7	1	7.50	5.98	8.54	7.82	8.32	5.56	9.12	8.08	8.72	5.28
958	0, 0	2	+	20	0.50	5	15	1	2.16	5.84	1.62	1.42	1.54	5.24	1.78	1.54	1.68	5.80

	D	VR	P	N	Δn	n1	n2	ε	F g	B-F g	F t	F-GG t	F-HF t	B-F t	F g x t	F-GG g x t	F-HF g x t	B-F g x t
959	0, 0	2	-	20	0.50	15	5	1	9.66	8.68	12.06	11.18	11.80	7.94	12.54	11.30	12.10	7.56
960	0, 0	2	0	40	0	20	20	1	5.04	4.88	4.82	4.64	4.74	4.44	4.92	4.76	4.86	4.48
961	0, 0	2	+	40	0.16	17	23	1	4.16	4.88	3.58	3.24	3.46	4.40	3.36	3.18	3.26	4.38
962	0, 0	2	-	40	0.16	23	17	1	6.26	5.34	6.74	6.40	6.68	5.00	6.60	6.34	6.40	5.16
963	0, 0	2	+	40	0.33	13	27	1	2.52	4.60	2.22	1.96	2.08	4.74	2.48	2.38	2.42	4.70
964	0, 0	2	-	40	0.33	27	13	1	8.22	5.50	9.52	9.12	9.26	5.20	8.52	8.04	8.38	4.82
965	0, 0	2	+	40	0.50	10	30	1	1.68	5.14	1.48	1.40	1.44	5.40	1.56	1.42	1.50	6.04
966	0, 0	2	-	40	0.50	30	10	1	9.38	6.60	11.46	11.00	11.26	6.32	11.40	10.94	11.22	5.90
967	0, 0	2	0	60	0	30	30	1	4.90	4.80	5.56	5.30	5.46	5.26	5.10	5.00	5.10	4.96
968	0, 0	2	+	60	0.16	25	35	1	3.74	4.90	3.92	3.80	3.90	5.28	3.54	3.40	3.50	4.74
969	0, 0	2	-	60	0.16	35	25	1	5.92	4.54	7.32	7.08	7.20	5.58	7.42	7.24	7.38	5.48
970	0, 0	2	+	60	0.33	20	40	1	3.06	5.20	2.70	2.58	2.64	4.92	2.56	2.36	2.48	5.40
971	0, 0	2	-	60	0.33	40	20	1	7.40	4.86	9.76	9.50	9.62	5.26	8.68	8.44	8.60	4.84
972	0, 0	2	+	60	0.50	15	45	1	2.18	5.72	1.60	1.58	1.60	5.24	1.74	1.62	1.70	5.76
973	0, 0	2	-	60	0.50	45	15	1	9.50	5.64	12.02	11.68	11.78	5.62	11.16	10.84	11.06	5.58
974	0, 0	2	0	80	0	40	40	1	4.80	4.66	5.42	5.24	5.38	5.04	4.68	4.48	4.60	4.42
975	0, 0	2	+	80	0.16	34	46	1	3.82	4.88	3.90	3.72	3.84	5.50	3.98	3.86	3.92	5.38
976	0, 0	2	-	80	0.16	46	34	1	5.92	4.74	7.52	7.32	7.40	5.26	6.66	6.42	6.56	4.88
977	0, 0	2	+	80	0.33	27	53	1	2.80	5.02	2.78	2.68	2.76	5.38	2.24	2.14	2.22	4.74
978	0, 0	2	-	80	0.33	53	27	1	7.26	4.88	9.78	9.50	9.66	5.64	9.42	9.18	9.32	5.26
979	0, 0	2	+	80	0.50	20	60	1	2.06	5.64	1.72	1.68	1.70	5.82	1.24	1.22	1.22	5.54
980	0, 0	2	-	80	0.50	60	20	1	9.62	5.30	12.38	12.24	12.34	5.72	11.94	11.80	11.88	5.68
981	0, 0	2	0	100	0	50	50	1	5.26	5.18	5.12	5.00	5.08	4.96	5.02	4.92	4.98	5.02
982	0, 0	2	+	100	0.16	42	58	1	3.64	4.98	3.94	3.88	3.92	5.42	3.78	3.64	3.70	5.16
983	0, 0	2	-	100	0.16	58	42	1	6.96	5.50	6.96	6.74	6.90	5.16	7.10	6.88	6.92	5.16
984	0, 0	2	+	100	0.33	33	67	1	2.80	5.12	2.42	2.34	2.38	5.24	2.68	2.66	2.68	5.42
985	0, 0	2	-	100	0.33	67	33	1	8.48	5.42	9.26	8.94	9.16	4.98	9.28	9.20	9.28	4.60
986	0, 0	2	+	100	0.50	25	75	1	1.86	5.06	1.50	1.46	1.50	5.18	1.50	1.46	1.50	4.80
987	0, 0	2	-	100	0.50	75	25	1	10.20	6.14	11.50	11.24	11.44	5.40	11.46	11.28	11.40	5.50
988	0, 0	2	0	150	0	75	75	1	5.84	5.78	5.16	5.06	5.16	5.08	5.34	5.20	5.28	5.38
989	0, 0	2	+	150	0.16	63	87	1	4.50	5.58	3.58	3.52	3.58	5.14	3.64	3.60	3.64	5.18
990	0, 0	2	-	150	0.16	87	63	1	6.36	5.00	7.10	6.96	7.08	5.16	7.28	7.24	7.28	5.04

	D	VR	P	N	Δn	n1	n2	ε	F g	B-F g	F t	F-GG t	F-HF t	B-F t	F g x t	F-GG g x t	F-HF g x t	B-F g x t
991	0, 0	2	+	150	0.33	50	100	1	3.12	5.18	2.32	2.24	2.30	5.24	2.42	2.38	2.42	5.16
992	0, 0	2	-	150	0.33	100	50	1	7.64	4.70	9.12	9.00	9.06	5.18	9.56	9.48	9.54	4.92
993	0, 0	2	+	150	0.50	37	113	1	2.06	5.46	1.48	1.42	1.44	5.64	1.46	1.46	1.46	5.06
994	0, 0	2	-	150	0.50	113	37	1	9.68	5.16	12.28	12.14	12.20	5.12	12.04	11.96	12.00	5.40
995	0, 0	2	0	200	0	100	100	1	5.18	5.10	5.00	4.84	4.98	4.98	5.52	5.52	5.52	5.62
996	0, 0	2	+	200	0.16	84	116	1	3.86	5.14	3.74	3.70	3.74	5.02	3.74	3.70	3.74	5.24
997	0, 0	2	-	200	0.16	116	84	1	6.84	5.20	6.82	6.78	6.82	4.68	7.40	7.34	7.34	5.04
998	0, 0	2	+	200	0.33	67	133	1	2.98	5.52	2.62	2.58	2.60	5.08	2.52	2.48	2.52	5.84
999	0, 0	2	-	200	0.33	133	67	1	8.14	5.04	9.38	9.28	9.36	4.98	9.50	9.36	9.48	5.40
1000	0, 0	2	+	200	0.50	50	150	1	2.18	5.24	1.70	1.70	1.70	5.42	1.16	1.12	1.16	5.22
1001	0, 0	2	-	200	0.50	150	50	1	10.04	5.32	12.22	12.10	12.18	5.64	12.26	12.12	12.20	5.50
1002	0, 0	5	0	20	0	10	10	1	5.16	4.38	5.64	5.06	5.40	4.42	5.62	4.90	5.32	4.02
1003	0, 0	5	+	20	0.16	8	12	1	3.20	4.28	2.62	2.30	2.44	3.94	2.32	2.02	2.22	3.90
1004	0, 0	5	-	20	0.16	12	8	1	9.44	5.50	11.14	9.98	10.60	4.54	10.94	9.68	10.18	4.40
1005	0, 0	5	+	20	0.33	7	13	1	2.28	4.26	1.48	1.28	1.40	3.42	1.32	1.10	1.22	3.60
1006	0, 0	5	-	20	0.33	13	7	1	11.86	6.20	14.52	13.24	13.84	4.82	14.88	13.58	14.10	4.70
1007	0, 0	5	+	20	0.50	5	15	1	.84	5.00	.22	.18	.20	4.70	.36	.28	.34	5.04
1008	0, 0	5	-	20	0.50	15	5	1	18.18	8.82	23.52	21.84	22.68	6.90	24.58	22.66	23.58	6.88
1009	0, 0	5	0	40	0	20	20	1	5.12	4.54	5.24	4.94	5.12	4.56	4.82	4.58	4.74	4.42
1010	0, 0	5	+	40	0.16	17	23	1	3.22	4.88	2.70	2.40	2.56	4.68	2.52	2.28	2.46	4.68
1011	0, 0	5	-	40	0.16	23	17	1	7.76	5.24	9.06	8.52	8.82	4.94	8.72	8.10	8.42	4.84
1012	0, 0	5	+	40	0.33	13	27	1	1.22	4.26	1.02	.90	1.00	4.40	.88	.84	.86	4.70
1013	0, 0	5	-	40	0.33	27	13	1	12.46	5.36	15.80	14.80	15.40	5.20	15.20	14.48	14.74	4.78
1014	0, 0	5	+	40	0.50	10	30	1	.58	4.38	.34	.30	.34	4.96	.28	.18	.20	5.24
1015	0, 0	5	-	40	0.50	30	10	1	17.00	6.34	22.08	21.12	21.36	5.66	22.70	21.82	22.14	5.18
1016	0, 0	5	0	60	0	30	30	1	4.86	4.50	5.56	5.30	5.42	5.10	5.48	5.16	5.34	4.88
1017	0, 0	5	+	60	0.16	25	35	1	2.88	4.72	2.78	2.66	2.74	5.02	2.48	2.28	2.42	4.86
1018	0, 0	5	-	60	0.16	35	25	1	7.62	4.08	10.02	9.50	9.74	5.16	9.76	9.44	9.64	5.16
1019	0, 0	5	+	60	0.33	20	40	1	1.58	4.98	1.14	1.08	1.10	5.28	1.02	.90	.94	5.40
1020	0, 0	5	-	60	0.33	40	20	1	11.54	4.78	15.90	15.42	15.68	4.60	14.96	14.50	14.68	4.60
1021	0, 0	5	+	60	0.50	15	45	1	.80	4.82	.36	.36	.36	5.12	.30	.30	.30	5.12
1022	0, 0	5	-	60	0.50	45	15	1	16.30	5.38	23.46	22.64	22.88	5.14	22.72	22.00	22.20	5.08

	D	VR	P	N	Δn	n1	n2	ε	F g	B-F g	F t	F-GG t	F-HF t	B-F t	F g x t	F-GG g x t	F-HF g x t	B-F g x t
1023	0, 0	5	0	80	0	40	40	1	4.90	4.56	5.42	5.30	5.34	5.00	5.20	5.06	5.10	4.92
1024	0, 0	5	+	80	0.16	34	46	1	2.82	4.82	2.96	2.80	2.88	5.44	2.76	2.68	2.72	5.60
1025	0, 0	5	-	80	0.16	46	34	1	7.40	4.80	9.50	9.18	9.34	5.36	8.78	8.48	8.64	4.80
1026	0, 0	5	+	80	0.33	27	53	1	1.54	4.64	1.36	1.32	1.32	5.28	.90	.80	.86	4.72
1027	0, 0	5	-	80	0.33	53	27	1	11.24	4.72	15.74	15.38	15.60	5.22	15.06	14.80	14.90	4.96
1028	0, 0	5	+	80	0.50	20	60	1	.70	5.16	.36	.34	.36	5.76	.22	.20	.22	5.20
1029	0, 0	5	-	80	0.50	60	20	1	16.46	5.30	23.50	23.08	23.36	5.62	23.20	22.60	22.88	5.12
1030	0, 0	5	0	100	0	50	50	1	5.16	5.20	5.22	5.08	5.12	5.04	5.22	5.14	5.16	4.96
1031	0, 0	5	+	100	0.16	42	58	1	2.68	4.52	2.54	2.38	2.44	5.06	2.66	2.56	2.64	4.96
1032	0, 0	5	-	100	0.16	58	42	1	8.16	5.58	9.16	9.04	9.12	5.06	9.32	9.04	9.16	5.04
1033	0, 0	5	+	100	0.33	33	67	1	1.16	4.86	.88	.84	.84	5.36	1.06	1.06	1.06	5.22
1034	0, 0	5	-	100	0.33	67	33	1	12.78	5.36	15.40	15.12	15.26	4.84	15.38	15.16	15.28	4.54
1035	0, 0	5	+	100	0.50	25	75	1	.52	4.68	.36	.36	.36	5.32	.18	.18	.18	4.68
1036	0, 0	5	-	100	0.50	75	25	1	18.02	5.66	22.66	22.26	22.46	5.00	22.10	21.70	21.86	4.90
1037	0, 0	5	0	150	0	75	75	1	5.72	5.66	5.38	5.24	5.30	5.14	5.20	5.06	5.12	4.96
1038	0, 0	5	+	150	0.16	63	87	1	3.42	5.42	2.56	2.46	2.52	4.96	2.74	2.64	2.70	5.22
1039	0, 0	5	-	150	0.16	87	63	1	7.94	4.88	9.44	9.32	9.40	4.72	9.46	9.34	9.42	5.00
1040	0, 0	5	+	150	0.33	50	100	1	1.56	5.12	.96	.94	.94	5.34	.92	.86	.90	4.94
1041	0, 0	5	-	150	0.33	100	50	1	11.24	4.88	15.40	15.20	15.30	5.04	15.38	15.06	15.22	4.80
1042	0, 0	5	+	150	0.50	37	113	1	.46	5.08	.34	.34	.34	5.28	.32	.30	.32	4.70
1043	0, 0	5	-	150	0.50	113	37	1	16.94	5.22	22.64	22.36	22.44	4.94	22.26	21.96	22.08	5.18
1044	0, 0	5	0	200	0	100	100	1	4.84	4.76	5.10	5.08	5.08	5.12	5.30	5.22	5.26	5.26
1045	0, 0	5	+	200	0.16	84	116	1	2.76	4.86	2.74	2.66	2.68	5.22	2.74	2.70	2.72	5.22
1046	0, 0	5	-	200	0.16	116	84	1	8.10	4.92	9.16	9.00	9.04	4.96	9.48	9.40	9.42	4.88
1047	0, 0	5	+	200	0.33	67	133	1	1.80	4.88	1.00	1.00	1.00	5.04	1.04	1.04	1.04	5.30
1048	0, 0	5	-	200	0.33	133	67	1	11.66	5.04	15.52	15.40	15.48	4.98	15.44	15.26	15.30	5.40
1049	0, 0	5	+	200	0.50	50	150	1	.70	4.92	.30	.30	.30	5.34	.14	.14	.14	4.86
1050	0, 0	5	-	200	0.50	150	50	1	17.06	5.30	22.82	22.68	22.74	5.56	23.48	23.34	23.42	4.94
1051	0.4, 0.8	1	0	20	0	10	10	.50	4.50	4.04	8.32	4.86	4.86	4.56	8.08	4.56	4.56	4.32
1052	0.4, 0.8	1	0	20	0.16	8	12	.50	5.10	4.74	8.62	4.96	4.98	4.74	9.06	5.18	5.20	4.78
1053	0.4, 0.8	1	0	20	0.33	7	13	.50	5.02	5.56	8.56	5.16	5.16	5.22	8.62	5.06	5.06	5.62
1054	0.4, 0.8	1	0	20	0.50	5	15	.50	5.12	7.00	8.46	5.08	5.12	6.94	8.98	5.28	5.28	7.00

	D	VR	P	N	Δn	n1	n2	ε	F g	B-F g	F t	F-GG t	F-HF t	B-F t	F g x t	F-GG g x t	F-HF g x t	B-F g x t
1055	0.4, 0.8	1	0	40	0	20	20	.50	4.78	4.54	8.42	4.64	4.64	4.54	8.00	4.78	4.78	4.62
1056	0.4, 0.8	1	0	40	0.16	17	23	.50	4.60	4.70	8.30	4.94	4.94	4.98	8.02	4.40	4.40	4.32
1057	0.4, 0.8	1	0	40	0.33	13	27	.50	4.52	4.68	8.36	4.94	4.94	5.24	7.94	4.76	4.76	4.78
1058	0.4, 0.8	1	0	40	0.50	10	30	.50	4.54	5.84	8.06	4.72	4.74	5.88	8.50	4.74	4.74	5.90
1059	0.4, 0.8	1	0	60	0	30	30	.50	4.98	4.90	8.40	4.88	4.88	4.60	8.34	5.12	5.12	5.10
1060	0.4, 0.8	1	0	60	0.16	25	35	.50	4.78	4.52	8.22	4.78	4.78	4.82	7.90	4.82	4.82	4.72
1061	0.4, 0.8	1	0	60	0.33	20	40	.50	4.94	5.00	8.48	4.88	4.88	5.02	8.14	5.08	5.08	5.02
1062	0.4, 0.8	1	0	60	0.50	15	45	.50	4.92	5.62	8.32	4.66	4.66	5.66	8.30	4.88	4.88	5.36
1063	0.4, 0.8	1	0	80	0	40	40	.50	5.22	5.16	8.52	4.70	4.70	4.64	8.58	5.18	5.20	5.36
1064	0.4, 0.8	1	0	80	0.16	34	46	.50	4.84	4.86	8.50	4.70	4.70	4.92	8.26	4.98	5.00	5.02
1065	0.4, 0.8	1	0	80	0.33	27	53	.50	5.22	5.32	8.18	4.82	4.82	5.12	8.24	5.06	5.06	5.22
1066	0.4, 0.8	1	0	80	0.50	20	60	.50	4.82	5.34	8.68	5.26	5.26	5.90	8.14	4.82	4.82	5.46
1067	0.4, 0.8	1	0	100	0	50	50	.50	5.28	5.34	8.42	5.00	5.00	4.86	8.42	5.06	5.06	5.24
1068	0.4, 0.8	1	0	100	0.16	42	58	.50	5.26	5.26	8.32	4.90	4.90	4.94	8.42	5.12	5.12	5.20
1069	0.4, 0.8	1	0	100	0.33	33	67	.50	4.88	5.14	8.26	4.74	4.74	4.86	8.16	4.78	4.78	4.96
1070	0.4, 0.8	1	0	100	0.50	25	75	.50	4.84	5.30	7.88	4.96	4.96	5.26	8.08	4.62	4.62	5.30
1071	0.4, 0.8	1	0	150	0	75	75	.50	5.48	5.56	7.62	4.62	4.62	4.64	9.06	5.28	5.28	5.66
1072	0.4, 0.8	1	0	150	0.16	63	87	.50	5.38	5.42	7.94	4.92	4.92	4.98	8.48	5.38	5.38	5.38
1073	0.4, 0.8	1	0	150	0.33	50	100	.50	5.22	5.60	8.40	4.62	4.64	5.16	9.02	5.38	5.38	5.78
1074	0.4, 0.8	1	0	150	0.50	37	113	.50	5.10	5.58	8.10	4.64	4.64	5.34	8.36	5.06	5.06	5.54
1075	0.4, 0.8	1	0	200	0	100	100	.50	5.18	5.28	8.18	4.90	4.90	4.76	8.70	5.12	5.12	5.08
1076	0.4, 0.8	1	0	200	0.16	84	116	.50	5.28	5.48	8.34	4.88	4.88	4.82	8.70	5.32	5.32	5.56
1077	0.4, 0.8	1	0	200	0.33	67	133	.50	5.16	5.20	8.14	4.70	4.70	5.00	8.54	5.32	5.32	5.40
1078	0.4, 0.8	1	0	200	0.50	50	150	.50	5.18	5.68	8.38	4.70	4.70	5.20	8.76	5.36	5.36	5.66
1079	0.4, 0.8	1.5	0	20	0	10	10	.50	4.54	4.04	8.68	5.00	5.00	4.44	8.32	4.80	4.82	4.28
1080	0.4, 0.8	1.5	+	20	0.16	8	12	.50	4.52	4.76	7.22	4.08	4.08	4.30	7.78	4.56	4.58	4.74
1081	0.4, 0.8	1.5	-	20	0.16	12	8	.50	5.26	4.44	10.02	5.96	5.98	4.88	9.54	5.46	5.48	4.66
1082	0.4, 0.8	1.5	+	20	0.33	7	13	.50	3.78	5.06	7.06	3.82	3.82	5.10	7.00	4.16	4.16	5.26
1083	0.4, 0.8	1.5	-	20	0.33	13	7	.50	6.18	5.50	10.80	6.52	6.56	5.44	10.96	6.22	6.24	5.46
1084	0.4, 0.8	1.5	+	20	0.50	5	15	.50	3.34	6.44	6.14	3.44	3.46	6.36	6.22	3.26	3.26	6.44
1085	0.4, 0.8	1.5	-	20	0.50	15	5	.50	7.82	8.22	11.86	7.74	7.74	7.24	12.52	7.70	7.74	7.90
1086	0.4, 0.8	1.5	0	40	0	20	20	.50	4.80	4.42	8.44	4.66	4.68	4.68	8.12	4.92	4.92	4.62

	D	VR	P	N	Δn	n1	n2	ε	F g	B-F g	F t	F-GG t	F-HF t	B-F t	F g x t	F-GG g x t	F-HF g x t	B-F g x t
1087	0.4, 0.8	1.5	+	40	0.16	17	23	.50	4.14	4.62	7.58	4.10	4.10	4.86	6.98	3.94	3.94	4.46
1088	0.4, 0.8	1.5	-	40	0.16	23	17	.50	5.30	4.90	9.34	5.84	5.84	5.16	9.24	5.22	5.24	4.90
1089	0.4, 0.8	1.5	+	40	0.33	13	27	.50	3.22	4.36	6.46	3.62	3.62	4.86	6.08	3.30	3.30	4.60
1090	0.4, 0.8	1.5	-	40	0.33	27	13	.50	5.64	4.90	10.38	6.42	6.46	5.34	9.94	5.88	5.92	5.04
1091	0.4, 0.8	1.5	+	40	0.50	10	30	.50	2.74	5.38	5.44	2.94	2.94	5.42	5.58	2.74	2.76	5.72
1092	0.4, 0.8	1.5	-	40	0.50	30	10	.50	7.50	6.06	11.72	7.28	7.28	6.20	11.64	7.32	7.34	6.12
1093	0.4, 0.8	1.5	0	60	0	30	30	.50	4.98	5.02	8.24	5.14	5.14	4.90	8.48	5.00	5.00	4.88
1094	0.4, 0.8	1.5	+	60	0.16	25	35	.50	4.26	4.62	7.32	4.14	4.16	4.68	6.98	4.18	4.18	4.76
1095	0.4, 0.8	1.5	-	60	0.16	35	25	.50	5.74	5.00	9.48	6.12	6.12	5.34	9.58	5.56	5.56	5.00
1096	0.4, 0.8	1.5	+	60	0.33	20	40	.50	3.84	5.12	6.32	3.72	3.72	4.92	6.52	3.76	3.76	5.04
1097	0.4, 0.8	1.5	-	60	0.33	40	20	.50	7.06	5.42	10.72	6.50	6.50	4.90	11.14	6.86	6.86	5.48
1098	0.4, 0.8	1.5	+	60	0.50	15	45	.50	3.24	5.22	5.10	3.00	3.02	5.22	5.66	3.26	3.26	5.12
1099	0.4, 0.8	1.5	-	60	0.50	45	15	.50	8.24	6.22	12.14	7.90	7.90	5.92	12.40	8.24	8.24	6.04
1100	0.4, 0.8	1.5	0	80	0	40	40	.50	5.22	5.16	8.26	4.74	4.74	4.78	8.66	5.16	5.16	5.14
1101	0.4, 0.8	1.5	+	80	0.16	34	46	.50	4.14	4.96	7.48	4.14	4.14	4.82	7.48	4.04	4.04	5.12
1102	0.4, 0.8	1.5	-	80	0.16	46	34	.50	5.76	4.92	9.48	5.64	5.66	5.02	9.66	6.08	6.08	5.10
1103	0.4, 0.8	1.5	+	80	0.33	27	53	.50	3.76	5.16	6.20	3.80	3.80	5.10	6.42	3.70	3.70	5.30
1104	0.4, 0.8	1.5	-	80	0.33	53	27	.50	6.80	5.34	11.20	6.84	6.84	5.40	11.04	7.20	7.20	5.28
1105	0.4, 0.8	1.5	+	80	0.50	20	60	.50	2.88	5.38	5.90	3.04	3.04	5.50	5.16	2.98	2.98	5.22
1106	0.4, 0.8	1.5	-	80	0.50	60	20	.50	8.16	6.16	12.62	8.08	8.08	6.06	12.66	8.04	8.04	6.04
1107	0.4, 0.8	1.5	0	100	0	50	50	.50	5.18	5.04	8.44	5.14	5.14	5.06	8.38	5.10	5.10	5.12
1108	0.4, 0.8	1.5	+	100	0.16	42	58	.50	4.48	5.18	7.28	4.20	4.20	4.98	7.32	4.50	4.50	5.12
1109	0.4, 0.8	1.5	-	100	0.16	58	42	.50	6.36	5.76	9.18	5.86	5.86	5.20	10.02	6.00	6.00	5.50
1110	0.4, 0.8	1.5	+	100	0.33	33	67	.50	3.64	4.92	6.30	3.44	3.44	4.88	6.32	3.30	3.30	4.76
1111	0.4, 0.8	1.5	-	100	0.33	67	33	.50	7.92	6.26	11.02	6.82	6.82	5.50	11.78	8.02	8.04	6.10
1112	0.4, 0.8	1.5	+	100	0.50	25	75	.50	2.78	4.98	5.54	3.10	3.10	5.08	5.28	2.78	2.78	4.90
1113	0.4, 0.8	1.5	-	100	0.50	75	25	.50	8.06	5.72	12.42	8.06	8.06	5.92	12.62	8.06	8.08	5.62
1114	0.4, 0.8	1.5	0	150	0	75	75	.50	5.58	5.42	7.56	4.50	4.50	4.60	8.68	5.58	5.58	5.50
1115	0.4, 0.8	1.5	+	150	0.16	63	87	.50	4.56	5.50	6.92	3.96	3.96	4.56	7.72	4.60	4.60	5.30
1116	0.4, 0.8	1.5	-	150	0.16	87	63	.50	6.00	5.46	8.56	5.00	5.00	4.48	9.74	6.00	6.00	5.44
1117	0.4, 0.8	1.5	+	150	0.33	50	100	.50	3.82	5.32	6.08	3.34	3.34	5.00	6.72	4.04	4.04	5.50
1118	0.4, 0.8	1.5	-	150	0.33	100	50	.50	6.98	5.34	10.02	5.86	5.88	4.58	10.42	6.88	6.88	5.36

	D	VR	P	N	Δn	n1	n2	ε	F g	B-F g	F t	F-GG t	F-HF t	B-F t	F g x t	F-GG g x t	F-HF g x t	B-F g x t
1119	0.4, 0.8	1.5	+	150	0.50	37	113	.50	2.88	5.42	5.14	2.98	2.98	5.32	5.54	3.08	3.08	5.40
1120	0.4, 0.8	1.5	-	150	0.50	113	37	.50	7.40	4.88	11.16	6.76	6.76	4.88	11.36	7.60	7.60	5.02
1121	0.4, 0.8	1.5	0	200	0	100	100	.50	5.30	5.34	8.08	4.82	4.82	4.80	8.86	5.48	5.48	5.36
1122	0.4, 0.8	1.5	+	200	0.16	84	116	.50	4.62	5.20	7.24	4.14	4.14	4.92	7.64	4.72	4.72	5.22
1123	0.4, 0.8	1.5	-	200	0.16	116	84	.50	5.74	5.18	8.92	5.54	5.54	4.86	9.38	5.86	5.86	5.26
1124	0.4, 0.8	1.5	+	200	0.33	67	133	.50	3.80	5.36	6.54	3.42	3.42	4.88	6.66	3.92	3.92	5.38
1125	0.4, 0.8	1.5	-	200	0.33	133	67	.50	7.20	5.62	10.48	6.66	6.66	5.14	10.60	6.90	6.90	5.58
1126	0.4, 0.8	1.5	+	200	0.50	50	150	.50	3.00	5.66	5.44	2.72	2.72	5.28	5.84	3.14	3.14	5.60
1127	0.4, 0.8	1.5	-	200	0.50	150	50	.50	7.76	5.62	11.70	7.92	7.92	5.42	12.04	7.60	7.60	5.42
1128	0.4, 0.8	2	0	20	0	10	10	.50	4.82	4.22	8.76	5.12	5.12	4.64	8.58	5.02	5.04	4.52
1129	0.4, 0.8	2	+	20	0.16	8	12	.50	4.04	4.86	6.66	3.66	3.68	4.12	7.24	4.10	4.10	4.60
1130	0.4, 0.8	2	-	20	0.16	12	8	.50	6.22	4.90	11.14	6.60	6.60	4.78	10.50	6.30	6.34	4.84
1131	0.4, 0.8	2	+	20	0.33	7	13	.50	3.30	4.42	6.06	3.12	3.12	4.00	6.08	3.54	3.54	4.48
1132	0.4, 0.8	2	-	20	0.33	13	7	.50	7.48	5.64	12.38	7.58	7.58	5.58	12.64	7.46	7.46	5.48
1133	0.4, 0.8	2	+	20	0.50	5	15	.50	2.50	6.20	4.64	2.44	2.46	5.84	4.76	2.38	2.40	6.12
1134	0.4, 0.8	2	-	20	0.50	15	5	.50	10.06	8.40	14.76	9.66	9.66	7.62	15.48	9.80	9.82	8.20
1135	0.4, 0.8	2	0	40	0	20	20	.50	5.08	4.60	8.40	4.92	4.92	4.78	8.32	4.82	4.82	4.56
1136	0.4, 0.8	2	+	40	0.16	17	23	.50	3.76	4.72	6.96	3.74	3.76	4.88	6.86	3.68	3.68	4.68
1137	0.4, 0.8	2	-	40	0.16	23	17	.50	5.88	4.84	9.92	6.26	6.28	5.36	10.04	5.90	5.92	5.00
1138	0.4, 0.8	2	+	40	0.33	13	27	.50	2.54	4.52	5.24	2.86	2.86	4.72	4.98	2.60	2.60	4.40
1139	0.4, 0.8	2	-	40	0.33	27	13	.50	6.90	4.88	12.26	7.76	7.76	5.58	11.74	7.04	7.04	5.14
1140	0.4, 0.8	2	+	40	0.50	10	30	.50	2.02	5.26	4.16	2.02	2.02	5.48	4.00	1.94	1.94	5.46
1141	0.4, 0.8	2	-	40	0.50	30	10	.50	9.20	6.10	14.78	9.54	9.54	6.40	14.22	9.46	9.48	6.26
1142	0.4, 0.8	2	0	60	0	30	30	.50	4.94	4.94	8.20	5.40	5.40	5.06	8.48	4.84	4.86	5.00
1143	0.4, 0.8	2	+	60	0.16	25	35	.50	3.80	4.68	6.74	3.78	3.78	5.02	6.56	3.92	3.92	4.90
1144	0.4, 0.8	2	-	60	0.16	35	25	.50	6.32	5.18	10.20	6.76	6.76	5.42	10.06	6.12	6.14	5.28
1145	0.4, 0.8	2	+	60	0.33	20	40	.50	3.02	4.94	5.36	2.82	2.82	4.84	5.60	3.02	3.02	4.80
1146	0.4, 0.8	2	-	60	0.33	40	20	.50	8.16	5.96	12.58	7.76	7.76	4.98	12.82	8.12	8.12	5.84
1147	0.4, 0.8	2	+	60	0.50	15	45	.50	2.42	5.14	4.06	2.06	2.06	5.12	4.28	2.32	2.32	5.06
1148	0.4, 0.8	2	-	60	0.50	45	15	.50	10.36	6.26	15.04	10.28	10.28	6.22	14.96	10.32	10.32	6.26
1149	0.4, 0.8	2	0	80	0	40	40	.50	5.16	5.04	8.32	4.72	4.72	4.70	8.46	5.12	5.12	5.02
1150	0.4, 0.8	2	+	80	0.16	34	46	.50	3.92	4.64	6.92	3.78	3.78	4.84	6.96	3.76	3.76	4.62

	D	VR	P	N	Δn	n1	n2	ε	F g	B-F g	F t	F-GG t	F-HF t	B-F t	F g x t	F-GG g x t	F-HF g x t	B-F g x t
1151	0.4, 0.8	2	-	80	0.16	46	34	.50	6.30	5.00	10.44	5.94	5.94	4.94	10.46	6.50	6.50	5.14
1152	0.4, 0.8	2	+	80	0.33	27	53	.50	2.96	4.92	5.16	3.10	3.10	4.90	5.66	2.86	2.86	5.20
1153	0.4, 0.8	2	-	80	0.33	53	27	.50	8.14	5.34	12.70	8.10	8.10	5.30	12.42	8.34	8.34	5.44
1154	0.4, 0.8	2	+	80	0.50	20	60	.50	2.18	5.20	4.20	2.16	2.16	5.44	3.96	1.82	1.82	5.16
1155	0.4, 0.8	2	-	80	0.50	60	20	.50	10.32	6.20	15.64	10.34	10.34	5.98	15.00	10.42	10.42	6.18
1156	0.4, 0.8	2	0	100	0	50	50	.50	5.02	5.04	8.26	5.06	5.06	5.00	8.28	5.04	5.04	5.12
1157	0.4, 0.8	2	+	100	0.16	42	58	.50	3.98	5.02	6.76	3.88	3.88	5.04	6.78	3.96	3.96	4.94
1158	0.4, 0.8	2	-	100	0.16	58	42	.50	6.98	5.80	9.82	6.42	6.42	5.30	10.58	6.70	6.70	5.36
1159	0.4, 0.8	2	+	100	0.33	33	67	.50	2.92	4.78	5.28	2.64	2.64	4.86	5.22	2.58	2.58	4.70
1160	0.4, 0.8	2	-	100	0.33	67	33	.50	9.14	6.14	12.72	8.20	8.20	5.74	13.32	9.30	9.30	6.06
1161	0.4, 0.8	2	+	100	0.50	25	75	.50	1.82	4.90	4.08	2.16	2.16	5.02	3.84	1.76	1.76	4.96
1162	0.4, 0.8	2	-	100	0.50	75	25	.50	10.48	6.00	15.10	10.44	10.44	6.14	15.38	10.22	10.22	5.74
1163	0.4, 0.8	2	0	150	0	75	75	.50	5.52	5.40	7.56	4.72	4.72	4.78	8.80	5.58	5.58	5.50
1164	0.4, 0.8	2	+	150	0.16	63	87	.50	4.04	5.54	6.14	3.54	3.54	4.44	7.22	4.06	4.06	5.44
1165	0.4, 0.8	2	-	150	0.16	87	63	.50	6.70	5.26	9.62	5.64	5.64	4.36	10.44	6.58	6.58	5.28
1166	0.4, 0.8	2	+	150	0.33	50	100	.50	2.94	5.30	5.00	2.72	2.72	4.78	5.54	3.12	3.12	5.48
1167	0.4, 0.8	2	-	150	0.33	100	50	.50	8.02	5.18	11.40	7.30	7.30	4.82	11.76	7.94	7.94	5.22
1168	0.4, 0.8	2	+	150	0.50	37	113	.50	2.14	5.28	3.84	2.12	2.12	4.96	3.98	2.20	2.20	5.32
1169	0.4, 0.8	2	-	150	0.50	113	37	.50	9.42	5.12	13.82	9.10	9.10	5.00	14.20	9.34	9.34	5.00
1170	0.4, 0.8	2	0	200	0	100	100	.50	5.46	5.28	7.96	4.84	4.84	4.76	8.90	5.46	5.46	5.52
1171	0.4, 0.8	2	+	200	0.16	84	116	.50	4.16	5.52	6.68	3.50	3.50	4.64	7.10	4.22	4.22	5.22
1172	0.4, 0.8	2	-	200	0.16	116	84	.50	6.46	5.36	9.80	6.14	6.14	5.12	10.20	6.50	6.50	5.38
1173	0.4, 0.8	2	+	200	0.33	67	133	.50	2.96	5.18	5.22	2.70	2.70	5.14	5.52	2.98	2.98	5.34
1174	0.4, 0.8	2	-	200	0.33	133	67	.50	8.58	5.58	12.20	7.84	7.84	5.18	12.42	8.32	8.34	5.28
1175	0.4, 0.8	2	+	200	0.50	50	150	.50	2.32	5.54	3.78	1.76	1.76	5.26	4.24	2.28	2.28	5.50
1176	0.4, 0.8	2	-	200	0.50	150	50	.50	9.90	5.42	14.52	10.00	10.00	5.42	14.66	9.86	9.86	5.40
1177	0.4, 0.8	5	0	20	0	10	10	.50	5.60	4.56	9.72	6.04	6.04	4.54	9.28	5.50	5.50	4.56
1178	0.4, 0.8	5	+	20	0.16	8	12	.50	3.00	4.50	5.78	2.90	2.90	4.00	5.96	3.18	3.20	4.56
1179	0.4, 0.8	5	-	20	0.16	12	8	.50	9.00	5.42	14.54	9.76	9.76	5.24	13.80	8.92	8.92	5.34
1180	0.4, 0.8	5	+	20	0.33	7	13	.50	2.10	4.04	4.06	1.98	2.00	3.58	4.38	2.34	2.36	4.14
1181	0.4, 0.8	5	-	20	0.33	13	7	.50	11.66	6.10	17.44	12.04	12.04	5.78	17.42	11.54	11.64	5.72
1182	0.4, 0.8	5	+	20	0.50	5	15	.50	.98	4.92	1.94	.86	.86	4.84	2.28	1.14	1.14	5.00

	D	VR	P	N	Δn	n1	n2	ε	F g	B-F g	F t	F-GG t	F-HF t	B-F t	F g x t	F-GG g x t	F-HF g x t	B-F g x t
1183	0.4, 0.8	5	-	20	0.50	15	5	.50	17.76	8.10	23.92	17.16	17.18	8.02	23.90	17.62	17.70	8.06
1184	0.4, 0.8	5	0	40	0	20	20	.50	5.22	4.70	8.84	5.44	5.44	4.86	8.54	5.36	5.36	4.86
1185	0.4, 0.8	5	+	40	0.16	17	23	.50	3.16	4.62	6.12	3.40	3.42	5.18	5.80	2.96	2.96	4.74
1186	0.4, 0.8	5	-	40	0.16	23	17	.50	7.96	4.80	12.08	7.94	7.94	5.26	12.34	7.66	7.66	5.02
1187	0.4, 0.8	5	+	40	0.33	13	27	.50	1.44	4.24	3.02	1.56	1.56	4.26	3.22	1.56	1.56	4.58
1188	0.4, 0.8	5	-	40	0.33	27	13	.50	12.10	5.26	18.20	12.62	12.66	5.88	18.02	12.26	12.26	5.16
1189	0.4, 0.8	5	+	40	0.50	10	30	.50	.76	4.98	1.78	.78	.78	5.06	1.66	.76	.76	4.86
1190	0.4, 0.8	5	-	40	0.50	30	10	.50	16.78	6.38	22.74	17.42	17.42	6.54	22.56	16.82	16.84	6.52
1191	0.4, 0.8	5	0	60	0	30	30	.50	4.98	4.70	8.46	5.34	5.36	4.96	8.34	5.12	5.12	4.82
1192	0.4, 0.8	5	+	60	0.16	25	35	.50	2.90	4.54	5.50	3.30	3.30	4.90	5.36	2.88	2.90	4.70
1193	0.4, 0.8	5	-	60	0.16	35	25	.50	7.88	5.36	12.68	8.46	8.46	5.34	11.98	7.86	7.86	5.38
1194	0.4, 0.8	5	+	60	0.33	20	40	.50	1.74	4.46	3.28	1.62	1.62	4.64	2.96	1.72	1.72	4.70
1195	0.4, 0.8	5	-	60	0.33	40	20	.50	12.40	5.68	17.52	12.30	12.30	5.36	17.84	12.44	12.44	5.62
1196	0.4, 0.8	5	+	60	0.50	15	45	.50	.74	4.96	1.54	.78	.78	4.88	1.70	.70	.70	4.98
1197	0.4, 0.8	5	-	60	0.50	45	15	.50	16.94	6.10	22.94	17.12	17.14	6.04	22.50	17.42	17.42	6.00
1198	0.4, 0.8	5	0	80	0	40	40	.50	4.82	4.68	8.38	4.72	4.72	4.46	8.46	4.94	4.94	4.80
1199	0.4, 0.8	5	+	80	0.16	34	46	.50	2.88	4.58	5.52	3.02	3.02	4.72	5.60	2.94	2.94	4.84
1200	0.4, 0.8	5	-	80	0.16	46	34	.50	8.10	4.86	12.34	7.74	7.74	4.92	12.74	8.02	8.02	4.88
1201	0.4, 0.8	5	+	80	0.33	27	53	.50	1.40	4.44	3.12	1.60	1.60	4.82	2.98	1.30	1.30	4.64
1202	0.4, 0.8	5	-	80	0.33	53	27	.50	12.12	5.24	17.42	12.42	12.42	5.38	17.20	12.06	12.06	5.24
1203	0.4, 0.8	5	+	80	0.50	20	60	.50	.58	4.66	1.66	.70	.70	4.94	1.36	.54	.54	4.84
1204	0.4, 0.8	5	-	80	0.50	60	20	.50	17.56	6.12	23.62	18.28	18.28	6.16	23.54	17.80	17.82	5.96
1205	0.4, 0.8	5	0	100	0	50	50	.50	5.18	4.82	8.20	5.24	5.24	4.86	8.18	5.34	5.34	5.16
1206	0.4, 0.8	5	+	100	0.16	42	58	.50	3.04	4.72	5.68	3.10	3.10	5.14	5.42	2.96	2.96	4.94
1207	0.4, 0.8	5	-	100	0.16	58	42	.50	8.46	5.40	12.16	7.90	7.90	5.26	12.70	8.32	8.32	5.18
1208	0.4, 0.8	5	+	100	0.33	33	67	.50	1.06	4.96	3.08	1.22	1.22	5.02	2.74	1.04	1.04	4.78
1209	0.4, 0.8	5	-	100	0.33	67	33	.50	13.14	5.66	17.86	13.06	13.06	5.02	18.12	13.12	13.12	5.62
1210	0.4, 0.8	5	+	100	0.50	25	75	.50	.48	4.50	1.34	.58	.58	4.98	1.40	.46	.46	4.58
1211	0.4, 0.8	5	-	100	0.50	75	25	.50	18.32	5.70	22.90	17.42	17.44	5.80	24.06	17.96	17.96	5.54
1212	0.4, 0.8	5	0	150	0	75	75	.50	5.38	5.20	8.10	4.88	4.88	4.62	8.76	5.36	5.36	5.32
1213	0.4, 0.8	5	+	150	0.16	63	87	.50	2.90	5.42	4.88	2.72	2.72	4.58	5.88	2.92	2.92	5.20
1214	0.4, 0.8	5	-	150	0.16	87	63	.50	8.20	5.06	11.74	7.66	7.66	4.80	12.52	8.38	8.38	4.96

	D	VR	P	N	Δn	n1	n2	ε	F g	B-F g	F t	F-GG t	F-HF t	B-F t	F g x t	F-GG g x t	F-HF g x t	B-F g x t
1215	0.4, 0.8	5	+	150	0.33	50	100	.50	1.22	5.12	2.58	1.20	1.20	4.50	3.02	1.24	1.24	4.98
1216	0.4, 0.8	5	-	150	0.33	100	50	.50	11.54	4.96	16.16	11.40	11.40	4.54	16.48	11.34	11.34	5.16
1217	0.4, 0.8	5	+	150	0.50	37	113	.50	.54	4.94	1.42	.60	.60	4.76	1.60	.52	.52	5.10
1218	0.4, 0.8	5	-	150	0.50	113	37	.50	16.78	4.80	22.38	16.56	16.56	4.88	22.60	16.74	16.74	4.90
1219	0.4, 0.8	5	0	200	0	100	100	.50	5.56	5.44	8.12	4.90	4.90	5.00	9.08	5.68	5.68	5.60
1220	0.4, 0.8	5	+	200	0.16	84	116	.50	3.30	5.28	5.36	2.84	2.84	4.90	5.74	3.34	3.34	5.42
1221	0.4, 0.8	5	-	200	0.16	116	84	.50	8.06	5.36	11.92	7.88	7.88	5.18	11.88	8.12	8.12	5.40
1222	0.4, 0.8	5	+	200	0.33	67	133	.50	1.68	5.06	2.86	1.32	1.32	4.84	3.48	1.54	1.54	5.14
1223	0.4, 0.8	5	-	200	0.33	133	67	.50	12.28	5.64	16.92	11.96	11.96	5.08	16.42	12.00	12.00	5.62
1224	0.4, 0.8	5	+	200	0.50	50	150	.50	.62	5.26	1.28	.46	.46	4.78	1.56	.52	.52	5.26
1225	0.4, 0.8	5	-	200	0.50	150	50	.50	17.32	5.38	22.48	17.12	17.12	5.28	22.66	17.60	17.60	5.42
1226	0.4, 0.8	1	0	20	0	10	10	.60	4.10	3.90	7.42	5.32	5.50	5.22	8.00	4.94	5.08	4.62
1227	0.4, 0.8	1	0	20	0.16	8	12	.60	4.72	4.32	7.90	5.26	5.38	5.20	7.38	4.72	4.94	4.76
1228	0.4, 0.8	1	0	20	0.33	7	13	.60	5.14	5.66	7.78	5.22	5.38	5.46	7.62	5.02	5.18	5.66
1229	0.4, 0.8	1	0	20	0.50	5	15	.60	4.84	6.84	7.48	5.02	5.14	7.18	7.24	4.72	4.96	7.40
1230	0.4, 0.8	1	0	40	0	20	20	.60	4.80	4.70	7.30	4.96	5.04	4.68	7.74	5.04	5.06	4.94
1231	0.4, 0.8	1	0	40	0.16	17	23	.60	4.98	5.10	7.30	4.84	4.92	4.84	7.34	4.60	4.70	4.80
1232	0.4, 0.8	1	0	40	0.33	13	27	.60	4.22	4.98	7.52	4.76	4.82	5.16	7.80	5.20	5.30	5.46
1233	0.4, 0.8	1	0	40	0.50	10	30	.60	4.36	5.80	7.34	4.64	4.68	6.04	7.68	5.02	5.10	6.28
1234	0.4, 0.8	1	0	60	0	30	30	.60	5.12	5.04	7.08	5.06	5.12	4.92	7.24	4.68	4.72	4.62
1235	0.4, 0.8	1	0	60	0.16	25	35	.60	5.14	5.10	7.16	4.92	4.96	4.92	7.44	5.32	5.36	5.26
1236	0.4, 0.8	1	0	60	0.33	20	40	.60	5.32	5.82	7.08	4.74	4.78	4.98	7.42	5.16	5.22	5.40
1237	0.4, 0.8	1	0	60	0.50	15	45	.60	5.08	5.84	7.38	4.74	4.76	5.58	7.14	4.88	4.92	5.32
1238	0.4, 0.8	1	0	80	0	40	40	.60	5.14	4.96	7.94	5.46	5.50	5.36	6.78	4.44	4.48	4.48
1239	0.4, 0.8	1	0	80	0.16	34	46	.60	4.98	5.24	7.96	5.12	5.14	5.32	7.32	4.96	5.00	5.16
1240	0.4, 0.8	1	0	80	0.33	27	53	.60	4.92	5.26	7.46	5.00	5.06	5.46	6.70	4.68	4.70	5.02
1241	0.4, 0.8	1	0	80	0.50	20	60	.60	5.32	6.12	7.48	5.14	5.18	5.52	7.42	5.00	5.00	5.52
1242	0.4, 0.8	1	0	100	0	50	50	.60	5.14	5.20	7.82	5.12	5.14	5.28	7.30	4.76	4.78	4.88
1243	0.4, 0.8	1	0	100	0.16	42	58	.60	5.12	5.08	7.84	5.22	5.22	5.46	6.76	4.50	4.50	4.52
1244	0.4, 0.8	1	0	100	0.33	33	67	.60	5.02	5.60	7.64	4.88	4.88	5.42	6.82	4.82	4.86	4.92
1245	0.4, 0.8	1	0	100	0.50	25	75	.60	4.88	5.66	7.36	4.76	4.78	5.42	7.10	4.64	4.66	5.28
1246	0.4, 0.8	1	0	150	0	75	75	.60	5.64	5.70	8.32	5.76	5.76	5.68	7.92	5.32	5.32	5.20

	D	VR	P	N	Δn	n1	n2	ε	F g	B-F g	F t	F-GG t	F-HF t	B-F t	F g x t	F-GG g x t	F-HF g x t	B-F g x t
1247	0.4, 0.8	1	0	150	0.16	63	87	.60	5.20	5.30	8.20	5.74	5.76	5.88	7.06	4.62	4.62	4.92
1248	0.4, 0.8	1	0	150	0.33	50	100	.60	5.34	5.56	8.10	5.70	5.72	5.70	7.48	4.80	4.80	5.04
1249	0.4, 0.8	1	0	150	0.50	37	113	.60	5.48	5.96	7.66	5.12	5.12	5.64	7.02	4.52	4.54	4.96
1250	0.4, 0.8	1	0	200	0	100	100	.60	5.02	5.02	7.80	5.34	5.34	5.52	7.34	5.12	5.12	5.18
1251	0.4, 0.8	1	0	200	0.16	84	116	.60	5.36	5.54	7.68	5.42	5.42	5.46	7.56	5.22	5.22	5.52
1252	0.4, 0.8	1	0	200	0.33	67	133	.60	5.04	5.28	8.02	5.64	5.66	5.74	7.34	5.10	5.10	5.18
1253	0.4, 0.8	1	0	200	0.50	50	150	.60	4.88	5.34	7.88	5.52	5.52	5.82	7.18	4.52	4.52	4.98
1254	0.4, 0.8	1.5	+	20	0.16	8	12	.60	3.90	4.48	6.86	4.74	4.90	5.10	6.38	4.02	4.20	4.62
1255	0.4, 0.8	1.5	0	20	0	10	10	.60	4.26	3.92	7.54	5.22	5.40	4.90	7.90	4.96	5.10	4.86
1256	0.4, 0.8	1.5	-	20	0.16	12	8	.60	5.52	4.68	9.16	5.96	6.10	5.26	9.42	6.46	6.58	5.32
1257	0.4, 0.8	1.5	+	20	0.33	7	13	.60	4.00	5.24	6.16	4.18	4.32	5.38	5.96	3.74	3.84	5.24
1258	0.4, 0.8	1.5	-	20	0.33	13	7	.60	6.32	5.46	9.56	6.70	6.90	6.06	10.42	7.02	7.24	6.14
1259	0.4, 0.8	1.5	+	20	0.50	5	15	.60	3.12	6.14	5.18	3.22	3.34	6.48	4.82	2.90	3.08	6.62
1260	0.4, 0.8	1.5	-	20	0.50	15	5	.60	7.70	8.00	11.08	8.08	8.34	7.86	10.88	7.80	7.96	7.40
1261	0.4, 0.8	1.5	0	40	0	20	20	.60	4.92	4.68	7.66	4.94	5.08	4.80	7.60	5.14	5.18	4.98
1262	0.4, 0.8	1.5	+	40	0.16	17	23	.60	4.52	5.02	6.52	4.14	4.24	4.66	6.52	3.86	4.00	4.58
1263	0.4, 0.8	1.5	-	40	0.16	23	17	.60	5.42	5.02	8.74	5.74	5.86	5.36	8.06	5.58	5.64	5.26
1264	0.4, 0.8	1.5	+	40	0.33	13	27	.60	3.10	4.80	5.38	3.42	3.42	4.84	5.80	3.76	3.80	5.02
1265	0.4, 0.8	1.5	-	40	0.33	27	13	.60	7.00	5.50	9.72	6.90	7.02	5.88	9.10	6.32	6.38	5.16
1266	0.4, 0.8	1.5	+	40	0.50	10	30	.60	2.54	5.30	4.58	2.72	2.78	5.72	4.96	3.06	3.08	6.08
1267	0.4, 0.8	1.5	-	40	0.50	30	10	.60	7.30	6.56	10.48	7.48	7.66	6.46	10.34	7.30	7.38	6.20
1268	0.4, 0.8	1.5	0	60	0	30	30	.60	4.96	4.56	7.44	5.02	5.06	5.06	7.12	4.52	4.58	4.56
1269	0.4, 0.8	1.5	+	60	0.16	25	35	.60	4.48	4.74	6.40	4.02	4.08	4.96	6.72	4.58	4.58	5.20
1270	0.4, 0.8	1.5	-	60	0.16	35	25	.60	5.42	4.72	8.80	5.88	5.96	5.10	8.52	5.52	5.52	5.00
1271	0.4, 0.8	1.5	+	60	0.33	20	40	.60	3.58	5.44	5.46	3.44	3.44	4.90	5.74	3.90	3.94	5.26
1272	0.4, 0.8	1.5	-	60	0.33	40	20	.60	6.48	4.86	9.88	6.76	6.86	5.32	9.34	6.70	6.78	4.72
1273	0.4, 0.8	1.5	+	60	0.50	15	45	.60	3.26	5.84	4.76	2.78	2.82	5.26	4.62	2.84	2.84	5.12
1274	0.4, 0.8	1.5	-	60	0.50	45	15	.60	7.48	5.70	11.02	8.02	8.10	6.14	10.40	7.54	7.68	6.16
1275	0.4, 0.8	1.5	0	80	0	40	40	.60	4.92	4.86	8.42	5.40	5.46	5.34	6.80	4.62	4.66	4.66
1276	0.4, 0.8	1.5	+	80	0.16	34	46	.60	4.28	5.10	6.94	4.68	4.70	5.34	6.54	4.62	4.68	5.26
1277	0.4, 0.8	1.5	-	80	0.16	46	34	.60	5.58	4.96	9.22	6.48	6.54	5.54	8.40	5.20	5.24	4.56
1278	0.4, 0.8	1.5	+	80	0.33	27	53	.60	3.64	4.88	5.88	3.64	3.70	5.36	5.24	3.24	3.26	4.96

	D	VR	P	N	Δn	n1	n2	ε	F g	B-F g	F t	F-GG t	F-HF t	B-F t	F g x t	F-GG g x t	F-HF g x t	B-F g x t
1279	0.4, 0.8	1.5	-	80	0.33	53	27	.60	6.30	5.08	10.62	7.54	7.62	5.70	9.44	6.34	6.34	4.90
1280	0.4, 0.8	1.5	+	80	0.50	20	60	.60	3.24	6.04	4.96	3.16	3.20	5.72	4.94	3.18	3.22	5.46
1281	0.4, 0.8	1.5	-	80	0.50	60	20	.60	7.24	5.64	11.60	8.22	8.22	6.08	10.82	7.66	7.76	5.76
1282	0.4, 0.8	1.5	0	100	0	50	50	.60	5.10	5.12	7.66	5.04	5.06	5.16	7.42	4.96	4.96	4.74
1283	0.4, 0.8	1.5	+	100	0.16	42	58	.60	4.36	5.06	6.90	4.38	4.40	5.38	5.90	3.78	3.82	4.66
1284	0.4, 0.8	1.5	-	100	0.16	58	42	.60	5.80	5.16	8.82	5.82	5.84	5.22	8.74	5.46	5.50	4.78
1285	0.4, 0.8	1.5	+	100	0.33	33	67	.60	3.68	5.18	5.74	3.52	3.52	5.38	5.32	3.36	3.42	4.94
1286	0.4, 0.8	1.5	-	100	0.33	67	33	.60	6.76	5.60	9.72	6.76	6.84	5.16	9.28	6.62	6.68	5.32
1287	0.4, 0.8	1.5	+	100	0.50	25	75	.60	2.98	5.34	4.70	2.80	2.86	5.42	4.52	2.80	2.82	4.94
1288	0.4, 0.8	1.5	-	100	0.50	75	25	.60	7.98	5.98	9.80	7.02	7.04	5.22	10.66	7.82	7.84	5.46
1289	0.4, 0.8	1.5	0	150	0	75	75	.60	5.64	5.68	8.56	5.62	5.68	5.66	8.04	5.38	5.38	5.30
1290	0.4, 0.8	1.5	+	150	0.16	63	87	.60	4.60	5.36	7.30	5.06	5.10	6.10	6.28	4.08	4.10	4.98
1291	0.4, 0.8	1.5	-	150	0.16	87	63	.60	5.72	4.74	9.26	6.50	6.52	5.58	8.96	5.58	5.64	4.90
1292	0.4, 0.8	1.5	+	150	0.33	50	100	.60	3.86	5.08	6.38	4.02	4.04	5.80	5.58	3.42	3.44	5.00
1293	0.4, 0.8	1.5	-	150	0.33	100	50	.60	6.34	4.90	10.02	7.20	7.22	5.74	9.16	6.80	6.80	5.16
1294	0.4, 0.8	1.5	+	150	0.50	37	113	.60	2.82	5.86	4.98	3.18	3.20	5.76	4.42	2.88	2.88	4.92
1295	0.4, 0.8	1.5	-	150	0.50	113	37	.60	7.44	5.26	11.02	7.76	7.76	5.08	10.16	7.26	7.28	5.20
1296	0.4, 0.8	1.5	0	200	0	100	100	.60	4.88	4.92	8.04	5.32	5.40	5.22	7.26	5.06	5.06	4.94
1297	0.4, 0.8	1.5	+	200	0.16	84	116	.60	4.52	5.48	6.76	4.84	4.84	5.40	6.94	4.30	4.32	5.30
1298	0.4, 0.8	1.5	-	200	0.16	116	84	.60	6.10	4.94	9.00	6.08	6.08	5.42	8.56	6.04	6.06	5.36
1299	0.4, 0.8	1.5	+	200	0.33	67	133	.60	3.54	5.12	6.14	4.04	4.04	5.50	5.74	3.66	3.68	5.12
1300	0.4, 0.8	1.5	-	200	0.33	133	67	.60	6.54	4.94	10.34	7.28	7.30	5.28	10.38	7.04	7.04	5.56
1301	0.4, 0.8	1.5	+	200	0.50	50	150	.60	2.96	5.20	5.52	3.30	3.30	5.92	4.56	2.40	2.42	4.88
1302	0.4, 0.8	1.5	-	200	0.50	150	50	.60	7.94	5.32	11.66	8.40	8.40	5.66	11.74	7.84	7.84	5.34
1303	0.4, 0.8	2	0	20	0	10	10	.60	4.40	4.18	7.68	5.26	5.50	5.06	8.06	5.08	5.26	4.72
1304	0.4, 0.8	2	+	20	0.16	8	12	.60	3.64	4.16	6.10	4.22	4.38	5.00	5.80	3.60	3.76	4.48
1305	0.4, 0.8	2	-	20	0.16	12	8	.60	6.30	4.90	10.16	6.72	7.14	5.46	10.42	7.18	7.36	5.54
1306	0.4, 0.8	2	+	20	0.33	7	13	.60	3.46	4.80	5.42	3.42	3.66	5.32	5.00	3.16	3.26	5.16
1307	0.4, 0.8	2	-	20	0.33	13	7	.60	7.38	5.80	10.96	8.00	8.28	6.20	11.86	8.40	8.60	6.30
1308	0.4, 0.8	2	+	20	0.50	5	15	.60	2.26	5.64	3.82	2.38	2.48	6.04	3.38	2.10	2.22	5.98
1309	0.4, 0.8	2	-	20	0.50	15	5	.60	9.82	8.36	13.90	10.02	10.28	8.08	13.44	10.14	10.30	7.68
1310	0.4, 0.8	2	0	40	0	20	20	.60	5.06	4.44	7.42	5.04	5.14	4.70	7.42	5.16	5.22	5.12

	D	VR	P	N	Δn	n1	n2	ε	F g	B-F g	F t	F-GG t	F-HF t	B-F t	F g x t	F-GG g x t	F-HF g x t	B-F g x t
1311	0.4, 0.8	2	+	40	0.16	17	23	.60	4.34	5.02	6.02	3.92	3.96	4.64	6.04	3.42	3.54	4.44
1312	0.4, 0.8	2	-	40	0.16	23	17	.60	6.22	5.28	9.36	6.46	6.60	5.30	8.78	6.10	6.18	5.26
1313	0.4, 0.8	2	+	40	0.33	13	27	.60	2.56	4.54	4.34	2.64	2.70	4.80	4.58	2.86	2.92	4.84
1314	0.4, 0.8	2	-	40	0.33	27	13	.60	8.18	5.82	11.14	8.26	8.28	5.96	11.10	7.66	7.82	5.46
1315	0.4, 0.8	2	+	40	0.50	10	30	.60	1.74	4.92	3.18	1.76	1.82	5.30	3.46	2.10	2.12	5.70
1316	0.4, 0.8	2	-	40	0.50	30	10	.60	9.42	6.56	13.32	9.56	9.60	6.52	13.44	9.60	9.64	6.40
1317	0.4, 0.8	2	0	60	0	30	30	.60	4.88	4.54	7.64	4.80	4.90	4.96	6.98	4.68	4.68	4.56
1318	0.4, 0.8	2	+	60	0.16	25	35	.60	3.88	4.88	5.94	3.72	3.72	4.82	6.10	3.88	3.92	5.04
1319	0.4, 0.8	2	-	60	0.16	35	25	.60	5.94	4.52	9.46	6.56	6.64	5.20	9.40	6.42	6.54	5.24
1320	0.4, 0.8	2	+	60	0.33	20	40	.60	2.74	5.28	4.38	2.68	2.74	4.76	4.76	3.36	3.40	5.14
1321	0.4, 0.8	2	-	60	0.33	40	20	.60	7.80	5.02	11.50	8.18	8.24	5.38	11.08	8.00	8.04	5.00
1322	0.4, 0.8	2	+	60	0.50	15	45	.60	2.14	5.62	3.26	1.82	1.84	5.16	3.22	2.00	2.00	5.16
1323	0.4, 0.8	2	-	60	0.50	45	15	.60	9.38	5.62	13.68	10.22	10.28	6.36	12.84	9.52	9.62	6.10
1324	0.4, 0.8	2	0	80	0	40	40	.60	4.86	4.88	8.42	5.48	5.50	5.32	7.14	4.70	4.72	4.80
1325	0.4, 0.8	2	+	80	0.16	34	46	.60	3.88	4.78	6.58	4.36	4.38	5.44	6.22	4.20	4.22	5.46
1326	0.4, 0.8	2	-	80	0.16	46	34	.60	5.92	4.82	9.92	7.22	7.22	5.54	8.92	6.10	6.10	4.80
1327	0.4, 0.8	2	+	80	0.33	27	53	.60	2.82	4.92	4.98	2.98	3.00	5.24	4.28	2.80	2.80	4.94
1328	0.4, 0.8	2	-	80	0.33	53	27	.60	7.48	5.02	12.22	8.82	8.86	5.88	11.02	7.90	7.94	5.12
1329	0.4, 0.8	2	+	80	0.50	20	60	.60	2.08	5.66	3.44	2.18	2.22	5.68	3.64	2.34	2.40	5.50
1330	0.4, 0.8	2	-	80	0.50	60	20	.60	9.28	5.70	14.40	10.40	10.58	6.38	13.62	9.94	9.96	5.78
1331	0.4, 0.8	2	0	100	0	50	50	.60	5.26	5.12	7.76	4.84	4.90	4.90	7.64	4.94	5.00	4.82
1332	0.4, 0.8	2	+	100	0.16	42	58	.60	4.00	5.10	6.24	3.90	3.90	5.32	5.46	3.46	3.52	4.62
1333	0.4, 0.8	2	-	100	0.16	58	42	.60	6.52	5.20	9.52	6.52	6.54	5.20	9.54	6.20	6.24	4.74
1334	0.4, 0.8	2	+	100	0.33	33	67	.60	2.70	5.30	4.54	2.76	2.78	5.18	4.40	2.76	2.78	4.86
1335	0.4, 0.8	2	-	100	0.33	67	33	.60	8.20	5.10	11.38	7.90	7.94	5.06	11.08	7.76	7.78	4.74
1336	0.4, 0.8	2	+	100	0.50	25	75	.60	2.10	5.08	3.32	1.80	1.80	5.26	3.28	1.78	1.82	5.00
1337	0.4, 0.8	2	-	100	0.50	75	25	.60	10.12	5.82	12.50	9.08	9.10	5.12	13.40	10.00	10.04	5.32
1338	0.4, 0.8	2	0	150	0	75	75	.60	5.58	5.56	8.62	5.76	5.76	5.64	8.00	5.42	5.42	5.36
1339	0.4, 0.8	2	+	150	0.16	63	87	.60	4.06	5.30	6.76	4.40	4.44	5.84	5.78	3.80	3.80	4.96
1340	0.4, 0.8	2	-	150	0.16	87	63	.60	6.32	4.86	9.82	7.16	7.16	5.44	9.88	6.70	6.70	5.10
1341	0.4, 0.8	2	+	150	0.33	50	100	.60	3.12	5.02	5.38	3.12	3.12	5.88	4.62	2.62	2.62	5.26
1342	0.4, 0.8	2	-	150	0.33	100	50	.60	7.52	4.88	11.42	8.36	8.36	5.74	10.70	7.80	7.80	5.06

	D	VR	P	N	Δn	n1	n2	ε	F g	B-F g	F t	F-GG t	F-HF t	B-F t	F g x t	F-GG g x t	F-HF g x t	B-F g x t
1343	0.4, 0.8	2	+	150	0.50	37	113	.60	1.98	5.64	3.56	2.12	2.12	5.78	3.32	2.10	2.12	5.16
1344	0.4, 0.8	2	-	150	0.50	113	37	.60	9.68	5.54	14.04	10.06	10.10	5.16	12.74	9.14	9.20	5.20
1345	0.4, 0.8	2	0	200	0	100	100	.60	4.86	4.78	7.96	5.28	5.28	5.30	7.26	4.92	4.94	4.94
1346	0.4, 0.8	2	+	200	0.16	84	116	.60	4.16	5.24	6.34	4.16	4.20	5.48	6.30	3.84	3.84	5.20
1347	0.4, 0.8	2	-	200	0.16	116	84	.60	6.76	4.76	9.76	6.62	6.68	5.40	9.14	6.56	6.58	5.32
1348	0.4, 0.8	2	+	200	0.33	67	133	.60	2.94	5.00	4.94	3.38	3.38	5.38	4.66	2.84	2.86	5.12
1349	0.4, 0.8	2	-	200	0.33	133	67	.60	8.00	4.84	12.18	8.86	8.90	5.32	12.10	8.58	8.60	5.38
1350	0.4, 0.8	2	+	200	0.50	50	150	.60	1.98	5.08	3.72	2.24	2.24	6.10	2.94	1.80	1.80	4.92
1351	0.4, 0.8	2	-	200	0.50	150	50	.60	10.08	5.28	14.58	10.86	10.90	5.48	14.30	10.56	10.58	5.36
1352	0.4, 0.8	5	0	20	0	10	10	.60	5.14	4.38	8.30	5.80	6.12	5.36	8.60	5.64	5.82	4.82
1353	0.4, 0.8	5	+	20	0.16	8	12	.60	3.12	4.48	4.96	3.22	3.36	4.90	4.24	2.50	2.62	4.34
1354	0.4, 0.8	5	-	20	0.16	12	8	.60	9.30	5.38	13.60	10.00	10.28	5.56	13.10	9.58	9.92	5.32
1355	0.4, 0.8	5	+	20	0.33	7	13	.60	2.46	4.96	3.68	2.20	2.32	4.88	3.66	2.24	2.32	4.76
1356	0.4, 0.8	5	-	20	0.33	13	7	.60	11.80	6.30	15.44	11.66	11.88	6.06	16.56	12.38	12.60	6.02
1357	0.4, 0.8	5	+	20	0.50	5	15	.60	.90	4.90	1.52	.78	.84	5.20	1.40	.74	.80	4.84
1358	0.4, 0.8	5	-	20	0.50	15	5	.60	17.88	8.92	23.36	18.90	19.02	8.40	23.18	18.08	18.44	8.12
1359	0.4, 0.8	5	0	40	0	20	20	.60	5.04	4.42	7.94	5.38	5.46	4.82	7.92	5.42	5.52	4.84
1360	0.4, 0.8	5	+	40	0.16	17	23	.60	3.48	4.88	4.80	3.14	3.22	4.48	4.84	2.78	2.88	4.50
1361	0.4, 0.8	5	-	40	0.16	23	17	.60	7.94	5.08	11.12	8.08	8.18	5.38	10.70	7.74	7.82	4.80
1362	0.4, 0.8	5	+	40	0.33	13	27	.60	1.18	4.58	2.36	1.16	1.16	4.78	2.36	1.42	1.44	4.76
1363	0.4, 0.8	5	-	40	0.33	27	13	.60	12.60	5.88	16.64	12.78	12.90	5.86	16.32	12.66	12.80	5.74
1364	0.4, 0.8	5	+	40	0.50	10	30	.60	.70	4.44	1.06	.64	.64	5.30	1.32	.62	.64	5.24
1365	0.4, 0.8	5	-	40	0.50	30	10	.60	16.44	6.40	22.00	17.14	17.30	6.70	22.12	17.34	17.40	6.64
1366	0.4, 0.8	5	0	60	0	30	30	.60	4.72	4.46	8.00	5.10	5.18	5.08	7.44	5.10	5.14	4.96
1367	0.4, 0.8	5	+	60	0.16	25	35	.60	3.06	4.74	4.72	3.04	3.06	4.86	4.62	3.00	3.04	4.74
1368	0.4, 0.8	5	-	60	0.16	35	25	.60	7.54	4.34	12.14	8.50	8.56	5.30	12.04	8.68	8.76	5.48
1369	0.4, 0.8	5	+	60	0.33	20	40	.60	1.58	4.76	2.56	1.52	1.56	4.74	2.82	1.64	1.66	5.12
1370	0.4, 0.8	5	-	60	0.33	40	20	.60	12.00	5.04	16.84	13.00	13.08	5.74	16.10	12.54	12.62	5.58
1371	0.4, 0.8	5	+	60	0.50	15	45	.60	.72	4.92	1.28	.76	.76	4.88	1.30	.78	.82	4.88
1372	0.4, 0.8	5	-	60	0.50	45	15	.60	16.70	5.52	21.76	17.16	17.26	5.90	21.56	16.90	17.04	5.86
1373	0.4, 0.8	5	0	80	0	40	40	.60	5.00	4.92	8.40	5.96	5.96	5.58	7.64	5.12	5.14	5.02
1374	0.4, 0.8	5	+	80	0.16	34	46	.60	2.86	4.66	5.38	3.38	3.42	5.60	5.36	3.54	3.54	5.70

	D	VR	P	N	Δn	n1	n2	ε	F g	B-F g	F t	F-GG t	F-HF t	B-F t	F g x t	F-GG g x t	F-HF g x t	B-F g x t
1375	0.4, 0.8	5	-	80	0.16	46	34	.60	7.80	4.80	11.70	8.66	8.76	5.64	10.80	7.64	7.70	5.24
1376	0.4, 0.8	5	+	80	0.33	27	53	.60	1.66	4.54	2.76	1.60	1.64	5.28	2.60	1.56	1.56	4.78
1377	0.4, 0.8	5	-	80	0.33	53	27	.60	11.54	4.80	16.62	12.78	12.84	5.66	15.72	11.88	11.92	5.18
1378	0.4, 0.8	5	+	80	0.50	20	60	.60	.62	5.30	1.22	.68	.70	5.64	1.12	.50	.50	5.44
1379	0.4, 0.8	5	-	80	0.50	60	20	.60	16.52	5.50	22.92	18.64	18.68	6.06	22.00	17.12	17.20	5.80
1380	0.4, 0.8	5	0	100	0	50	50	.60	5.28	5.16	7.72	5.20	5.22	4.94	7.64	5.12	5.12	4.68
1381	0.4, 0.8	5	+	100	0.16	42	58	.60	2.88	4.74	4.72	2.84	2.90	5.34	4.70	2.80	2.80	5.20
1382	0.4, 0.8	5	-	100	0.16	58	42	.60	7.88	5.18	11.36	8.00	8.02	4.90	11.66	7.68	7.72	4.56
1383	0.4, 0.8	5	+	100	0.33	33	67	.60	1.16	4.82	2.34	1.30	1.32	5.10	2.60	1.34	1.34	5.30
1384	0.4, 0.8	5	-	100	0.33	67	33	.60	12.60	5.20	16.18	12.28	12.34	4.84	15.80	12.10	12.16	4.62
1385	0.4, 0.8	5	+	100	0.50	25	75	.60	.46	4.78	1.06	.44	.44	5.10	1.12	.50	.50	4.78
1386	0.4, 0.8	5	-	100	0.50	75	25	.60	17.82	5.50	21.30	16.92	16.96	5.24	22.36	17.52	17.58	5.40
1387	0.4, 0.8	5	0	150	0	75	75	.60	5.48	5.40	8.08	5.62	5.64	5.34	8.14	5.64	5.64	5.64
1388	0.4, 0.8	5	+	150	0.16	63	87	.60	3.38	5.24	5.48	3.34	3.36	5.74	4.84	3.12	3.14	5.24
1389	0.4, 0.8	5	-	150	0.16	87	63	.60	8.16	5.02	11.80	8.80	8.82	5.42	11.86	8.68	8.70	5.38
1390	0.4, 0.8	5	+	150	0.33	50	100	.60	1.48	4.80	2.90	1.36	1.36	5.94	2.56	1.38	1.40	4.92
1391	0.4, 0.8	5	-	150	0.33	100	50	.60	11.18	4.88	16.40	12.30	12.32	5.48	15.76	11.76	11.80	5.14
1392	0.4, 0.8	5	+	150	0.50	37	113	.60	.52	5.12	1.12	.62	.62	5.82	1.22	.62	.64	4.92
1393	0.4, 0.8	5	-	150	0.50	113	37	.60	17.30	5.48	22.04	17.38	17.42	5.38	21.54	16.90	16.90	5.02
1394	0.4, 0.8	5	0	200	0	100	100	.60	5.06	4.74	7.84	5.44	5.46	5.28	7.34	4.94	4.96	4.98
1395	0.4, 0.8	5	+	200	0.16	84	116	.60	2.86	4.94	4.96	3.06	3.06	5.42	4.88	3.04	3.04	5.34
1396	0.4, 0.8	5	-	200	0.16	116	84	.60	7.86	5.02	11.68	8.52	8.52	5.58	11.06	7.84	7.84	5.40
1397	0.4, 0.8	5	+	200	0.33	67	133	.60	1.58	4.90	2.82	1.64	1.64	5.32	2.56	1.44	1.44	4.90
1398	0.4, 0.8	5	-	200	0.33	133	67	.60	12.06	5.16	17.04	13.26	13.26	5.58	16.58	13.00	13.00	5.32
1399	0.4, 0.8	5	+	200	0.50	50	150	.60	.54	4.78	1.30	.66	.68	5.54	1.02	.36	.38	4.90
1400	0.4, 0.8	5	-	200	0.50	150	50	.60	17.04	5.46	22.52	18.20	18.22	5.48	22.64	18.14	18.14	5.68
1401	0.4, 0.8	1	0	20	0	10	10	.70	4.46	4.04	6.90	5.28	5.48	5.06	6.70	5.08	5.40	4.88
1402	0.4, 0.8	1	0	20	0.16	8	12	.70	4.76	4.48	6.78	5.04	5.38	4.98	6.72	4.80	5.04	4.90
1403	0.4, 0.8	1	0	20	0.33	7	13	.70	5.18	5.40	6.56	4.74	4.94	5.40	6.80	4.70	5.16	5.28
1404	0.4, 0.8	1	0	20	0.50	5	15	.70	5.18	7.30	6.58	4.72	4.98	7.04	6.50	4.62	4.92	6.70
1405	0.4, 0.8	1	0	40	0	20	20	.70	5.16	4.80	6.74	4.90	5.02	4.90	6.54	4.64	4.80	4.74
1406	0.4, 0.8	1	0	40	0.16	17	23	.70	4.82	4.80	6.90	4.94	5.08	5.10	6.30	4.60	4.72	4.54

	D	VR	P	N	Δn	n1	n2	ε	F g	B-F g	F t	F-GG t	F-HF t	B-F t	F g x t	F-GG g x t	F-HF g x t	B-F g x t
1407	0.4, 0.8	1	0	40	0.33	13	27	.70	4.70	5.28	6.92	5.02	5.22	5.62	6.24	4.62	4.80	4.80
1408	0.4, 0.8	1	0	40	0.50	10	30	.70	4.54	5.76	6.58	4.90	5.02	6.44	6.42	4.60	4.82	6.20
1409	0.4, 0.8	1	0	60	0	30	30	.70	4.46	4.54	6.72	4.90	4.92	4.98	7.12	5.22	5.32	5.18
1410	0.4, 0.8	1	0	60	0.16	25	35	.70	4.70	4.46	6.76	4.82	4.96	5.14	6.72	4.94	5.08	5.06
1411	0.4, 0.8	1	0	60	0.33	20	40	.70	5.30	5.46	6.70	4.76	4.86	5.18	6.42	4.86	4.88	4.98
1412	0.4, 0.8	1	0	60	0.50	15	45	.70	4.82	5.92	6.54	5.06	5.06	5.62	6.58	5.00	5.08	5.34
1413	0.4, 0.8	1	0	80	0	40	40	.70	4.66	4.52	7.24	5.42	5.44	5.56	6.72	5.16	5.18	5.12
1414	0.4, 0.8	1	0	80	0.16	34	46	.70	4.82	4.78	7.40	5.38	5.52	5.56	6.94	5.24	5.26	5.34
1415	0.4, 0.8	1	0	80	0.33	27	53	.70	5.02	5.20	7.38	5.54	5.56	5.78	6.80	4.92	5.06	5.30
1416	0.4, 0.8	1	0	80	0.50	20	60	.70	5.14	5.82	7.36	5.66	5.70	5.98	6.44	4.74	4.78	5.44
1417	0.4, 0.8	1	0	100	0	50	50	.70	4.86	5.12	7.28	5.42	5.46	5.66	6.82	4.86	4.90	5.16
1418	0.4, 0.8	1	0	100	0.16	42	58	.70	5.04	5.00	7.12	5.46	5.56	5.44	6.68	4.98	5.02	5.20
1419	0.4, 0.8	1	0	100	0.33	33	67	.70	5.04	5.36	7.18	5.40	5.44	5.52	7.02	5.10	5.14	5.32
1420	0.4, 0.8	1	0	100	0.50	25	75	.70	4.78	5.40	6.92	5.62	5.66	5.86	6.36	4.68	4.72	5.24
1421	0.4, 0.8	1	0	150	0	75	75	.70	5.58	5.70	6.82	5.28	5.30	5.42	6.84	5.50	5.52	5.64
1422	0.4, 0.8	1	0	150	0.16	63	87	.70	5.52	5.42	7.06	5.28	5.30	5.46	6.70	4.84	4.88	5.16
1423	0.4, 0.8	1	0	150	0.33	50	100	.70	4.92	5.30	7.02	5.38	5.44	5.72	7.12	5.32	5.38	5.74
1424	0.4, 0.8	1	0	150	0.50	37	113	.70	4.94	5.68	6.66	5.12	5.16	5.52	7.28	5.54	5.56	6.08
1425	0.4, 0.8	1	0	200	0	100	100	.70	5.16	5.16	6.96	5.12	5.12	5.24	6.84	5.12	5.16	5.40
1426	0.4, 0.8	1	0	200	0.16	84	116	.70	5.04	5.22	7.08	4.92	4.92	5.38	7.14	5.20	5.22	5.48
1427	0.4, 0.8	1	0	200	0.33	67	133	.70	5.24	5.48	6.84	5.28	5.30	5.40	6.90	5.10	5.16	5.30
1428	0.4, 0.8	1	0	200	0.50	50	150	.70	4.84	5.20	6.92	5.30	5.30	5.52	6.88	5.12	5.16	5.70
1429	0.4, 0.8	1.5	0	20	0	10	10	.70	4.42	4.06	6.92	5.00	5.38	4.94	7.12	5.12	5.40	4.90
1430	0.4, 0.8	1.5	+	20	0.16	8	12	.70	3.98	4.46	5.86	4.20	4.54	5.08	6.02	4.16	4.60	4.80
1431	0.4, 0.8	1.5	-	20	0.16	12	8	.70	5.92	5.04	8.20	5.76	6.18	4.94	8.20	5.82	6.22	5.00
1432	0.4, 0.8	1.5	+	20	0.33	7	13	.70	4.00	5.16	5.06	3.52	3.68	5.20	5.08	3.68	3.88	5.16
1433	0.4, 0.8	1.5	-	20	0.33	13	7	.70	6.16	5.38	8.86	6.54	6.80	5.60	8.38	6.42	6.64	5.38
1434	0.4, 0.8	1.5	+	20	0.50	5	15	.70	3.14	6.54	4.32	3.16	3.34	6.36	3.96	2.68	2.94	6.50
1435	0.4, 0.8	1.5	-	20	0.50	15	5	.70	7.62	7.78	10.08	7.74	8.10	7.74	10.64	8.16	8.62	8.00
1436	0.4, 0.8	1.5	0	40	0	20	20	.70	4.90	4.60	6.72	5.00	5.12	4.88	6.36	4.56	4.70	4.62
1437	0.4, 0.8	1.5	+	40	0.16	17	23	.70	4.24	4.70	5.88	4.24	4.32	4.84	5.42	3.90	4.00	4.58
1438	0.4, 0.8	1.5	-	40	0.16	23	17	.70	5.52	4.70	7.80	5.80	5.88	5.24	7.60	5.44	5.56	5.08

	D	VR	P	N	Δn	n1	n2	ε	F g	B-F g	F t	F-GG t	F-HF t	B-F t	F g x t	F-GG g x t	F-HF g x t	B-F g x t
1439	0.4, 0.8	1.5	+	40	0.33	13	27	.70	3.22	4.94	4.94	3.42	3.56	5.54	4.80	3.18	3.32	4.64
1440	0.4, 0.8	1.5	-	40	0.33	27	13	.70	6.34	5.34	8.80	6.92	7.06	5.72	8.72	6.68	6.84	5.20
1441	0.4, 0.8	1.5	+	40	0.50	10	30	.70	2.64	5.70	4.10	2.96	3.02	5.86	3.80	2.74	2.80	5.74
1442	0.4, 0.8	1.5	-	40	0.50	30	10	.70	7.12	6.52	10.18	8.02	8.14	6.78	10.10	7.80	8.00	6.78
1443	0.4, 0.8	1.5	0	60	0	30	30	.70	4.46	4.48	6.80	5.20	5.28	5.38	6.98	5.08	5.24	5.18
1444	0.4, 0.8	1.5	+	60	0.16	25	35	.70	4.10	4.58	5.58	4.18	4.26	4.98	5.66	4.38	4.44	5.18
1445	0.4, 0.8	1.5	-	60	0.16	35	25	.70	5.50	4.58	8.02	6.20	6.32	5.44	7.70	5.78	6.00	5.22
1446	0.4, 0.8	1.5	+	60	0.33	20	40	.70	3.84	5.30	4.78	3.48	3.56	4.98	4.90	3.34	3.42	4.94
1447	0.4, 0.8	1.5	-	60	0.33	40	20	.70	6.14	5.12	8.96	6.94	7.04	5.48	9.12	6.82	6.92	5.38
1448	0.4, 0.8	1.5	+	60	0.50	15	45	.70	2.98	5.62	4.16	3.06	3.12	5.44	4.14	2.92	2.96	5.34
1449	0.4, 0.8	1.5	-	60	0.50	45	15	.70	7.80	6.12	10.24	8.06	8.20	5.74	10.10	7.80	7.96	5.72
1450	0.4, 0.8	1.5	0	80	0	40	40	.70	4.70	4.48	7.20	5.50	5.54	5.44	6.72	4.90	4.98	5.10
1451	0.4, 0.8	1.5	+	80	0.16	34	46	.70	4.16	4.70	6.30	4.84	4.84	5.56	5.96	4.26	4.30	5.14
1452	0.4, 0.8	1.5	-	80	0.16	46	34	.70	5.58	4.78	8.22	6.26	6.42	5.76	8.18	5.98	6.04	5.18
1453	0.4, 0.8	1.5	+	80	0.33	27	53	.70	3.32	4.70	5.54	4.06	4.10	5.68	5.00	3.50	3.52	5.02
1454	0.4, 0.8	1.5	-	80	0.33	53	27	.70	6.40	4.94	9.48	7.32	7.42	5.98	8.88	6.72	6.78	5.22
1455	0.4, 0.8	1.5	+	80	0.50	20	60	.70	3.14	5.62	4.90	3.58	3.58	5.92	3.86	2.72	2.74	5.00
1456	0.4, 0.8	1.5	-	80	0.50	60	20	.70	7.40	5.76	10.96	8.90	8.94	6.24	10.28	7.88	7.94	6.06
1457	0.4, 0.8	1.5	0	100	0	50	50	.70	4.96	4.76	7.22	5.30	5.36	5.38	6.46	5.10	5.16	4.92
1458	0.4, 0.8	1.5	+	100	0.16	42	58	.70	4.42	5.14	6.44	4.66	4.66	5.34	5.70	4.10	4.16	5.06
1459	0.4, 0.8	1.5	-	100	0.16	58	42	.70	5.90	5.18	8.06	6.12	6.18	5.38	7.56	5.52	5.56	5.00
1460	0.4, 0.8	1.5	+	100	0.33	33	67	.70	3.66	5.24	5.38	4.02	4.02	5.70	5.02	3.52	3.52	5.34
1461	0.4, 0.8	1.5	-	100	0.33	67	33	.70	6.94	5.28	8.90	7.02	7.04	5.20	9.46	6.70	6.82	4.58
1462	0.4, 0.8	1.5	+	100	0.50	25	75	.70	2.88	5.10	4.74	3.42	3.48	5.90	3.86	2.44	2.46	5.10
1463	0.4, 0.8	1.5	-	100	0.50	75	25	.70	8.00	5.92	10.18	7.84	7.86	5.78	10.36	7.86	7.90	5.54
1464	0.4, 0.8	1.5	0	150	0	75	75	.70	5.76	5.82	6.48	5.10	5.12	5.28	7.02	5.16	5.22	5.34
1465	0.4, 0.8	1.5	+	150	0.16	63	87	.70	4.68	5.38	6.18	4.54	4.58	5.48	6.02	4.16	4.16	5.32
1466	0.4, 0.8	1.5	-	150	0.16	87	63	.70	6.16	5.22	7.46	5.52	5.62	5.00	7.96	6.24	6.28	5.48
1467	0.4, 0.8	1.5	+	150	0.33	50	100	.70	3.48	4.98	5.30	3.92	3.94	5.68	5.46	3.68	3.74	5.56
1468	0.4, 0.8	1.5	-	150	0.33	100	50	.70	6.38	4.96	9.06	6.94	6.98	5.16	8.88	6.48	6.52	4.78
1469	0.4, 0.8	1.5	+	150	0.50	37	113	.70	2.92	5.46	4.26	3.14	3.18	5.60	4.52	3.06	3.10	5.72
1470	0.4, 0.8	1.5	-	150	0.50	113	37	.70	7.52	5.18	9.92	7.84	7.88	5.22	9.72	7.38	7.46	5.08

	D	VR	P	N	Δn	n1	n2	ε	F g	B-F g	F t	F-GG t	F-HF t	B-F t	F g x t	F-GG g x t	F-HF g x t	B-F g x t
1471	0.4, 0.8	1.5	0	200	0	100	100	.70	5.02	5.20	6.88	5.26	5.28	5.34	6.76	5.06	5.06	5.40
1472	0.4, 0.8	1.5	+	200	0.16	84	116	.70	4.36	5.14	5.88	4.38	4.44	5.26	6.16	4.60	4.60	5.62
1473	0.4, 0.8	1.5	-	200	0.16	116	84	.70	5.94	4.98	7.64	5.76	5.82	5.10	7.80	5.84	5.86	4.96
1474	0.4, 0.8	1.5	+	200	0.33	67	133	.70	3.88	5.32	5.04	3.68	3.68	5.18	4.94	3.52	3.58	4.96
1475	0.4, 0.8	1.5	-	200	0.33	133	67	.70	7.12	5.50	8.90	6.90	6.92	5.22	8.92	6.78	6.82	5.36
1476	0.4, 0.8	1.5	+	200	0.50	50	150	.70	2.80	5.50	4.44	3.00	3.02	5.32	4.26	3.02	3.02	5.62
1477	0.4, 0.8	1.5	-	200	0.50	150	50	.70	7.76	5.38	10.16	7.78	7.84	4.98	10.00	7.86	7.92	5.22
1478	0.4, 0.8	2	0	20	0	10	10	.70	4.44	4.04	6.94	5.18	5.40	4.70	7.20	5.32	5.64	5.00
1479	0.4, 0.8	2	+	20	0.16	8	12	.70	3.84	4.62	5.38	3.60	3.88	4.96	5.24	3.72	4.00	4.98
1480	0.4, 0.8	2	-	20	0.16	12	8	.70	6.76	5.22	9.12	6.96	7.32	5.06	9.26	6.76	7.12	4.88
1481	0.4, 0.8	2	+	20	0.33	7	13	.70	3.48	4.98	4.32	2.82	3.10	4.92	4.22	2.80	3.10	4.90
1482	0.4, 0.8	2	-	20	0.33	13	7	.70	7.30	5.74	10.20	8.04	8.38	5.80	10.36	7.78	8.10	5.62
1483	0.4, 0.8	2	+	20	0.50	5	15	.70	2.38	6.20	3.02	1.92	2.14	6.08	2.60	1.88	2.02	6.06
1484	0.4, 0.8	2	-	20	0.50	15	5	.70	9.82	8.06	13.06	10.28	10.62	8.06	13.66	10.76	11.16	8.38
1485	0.4, 0.8	2	0	40	0	20	20	.70	4.88	4.54	6.76	5.16	5.22	4.96	6.54	4.48	4.60	4.48
1486	0.4, 0.8	2	+	40	0.16	17	23	.70	3.82	4.56	5.34	3.94	4.04	4.54	4.92	3.54	3.64	4.62
1487	0.4, 0.8	2	-	40	0.16	23	17	.70	5.98	4.72	8.40	6.40	6.58	5.30	8.26	6.32	6.46	5.14
1488	0.4, 0.8	2	+	40	0.33	13	27	.70	2.46	4.64	3.86	2.56	2.66	5.28	3.60	2.62	2.68	4.98
1489	0.4, 0.8	2	-	40	0.33	27	13	.70	7.50	5.30	10.50	8.14	8.22	5.62	10.70	8.18	8.36	5.32
1490	0.4, 0.8	2	+	40	0.50	10	30	.70	1.90	5.46	2.84	1.94	2.10	6.00	2.74	1.96	2.04	5.44
1491	0.4, 0.8	2	-	40	0.50	30	10	.70	9.40	6.64	12.94	10.32	10.48	6.78	13.08	10.30	10.66	7.08
1492	0.4, 0.8	2	0	60	0	30	30	.70	4.44	4.46	7.14	5.26	5.34	5.32	7.08	5.32	5.42	5.30
1493	0.4, 0.8	2	+	60	0.16	25	35	.70	3.68	4.62	5.16	3.92	3.96	5.14	5.28	4.06	4.12	5.22
1494	0.4, 0.8	2	-	60	0.16	35	25	.70	6.18	4.72	8.96	6.70	6.86	5.50	8.36	6.42	6.46	5.26
1495	0.4, 0.8	2	+	60	0.33	20	40	.70	2.84	5.18	4.00	2.72	2.76	4.90	3.88	2.66	2.70	4.76
1496	0.4, 0.8	2	-	60	0.33	40	20	.70	7.76	5.32	10.72	8.34	8.38	5.40	10.90	8.50	8.60	5.28
1497	0.4, 0.8	2	+	60	0.50	15	45	.70	2.00	5.46	2.96	2.04	2.06	5.42	2.74	1.66	1.68	5.04
1498	0.4, 0.8	2	-	60	0.50	45	15	.70	9.82	6.08	13.18	10.34	10.48	5.88	13.36	10.40	10.50	5.70
1499	0.4, 0.8	2	0	80	0	40	40	.70	4.64	4.50	7.18	5.54	5.58	5.38	6.64	4.94	4.98	4.90
1500	0.4, 0.8	2	+	80	0.16	34	46	.70	3.62	4.80	5.80	4.24	4.28	5.44	5.32	3.88	3.92	5.06
1501	0.4, 0.8	2	-	80	0.16	46	34	.70	6.00	4.78	8.88	6.78	6.84	5.44	8.46	6.50	6.60	5.36
1502	0.4, 0.8	2	+	80	0.33	27	53	.70	2.48	4.72	4.34	3.22	3.22	5.58	3.94	2.66	2.68	5.22

	D	VR	P	N	Δn	n1	n2	ε	F g	B-F g	F t	F-GG t	F-HF t	B-F t	F g x t	F-GG g x t	F-HF g x t	B-F g x t
1503	0.4, 0.8	2	-	80	0.33	53	27	.70	7.64	5.16	11.26	8.68	8.78	5.80	10.44	8.04	8.16	5.38
1504	0.4, 0.8	2	+	80	0.50	20	60	.70	2.20	5.46	3.56	2.24	2.28	5.80	2.52	1.70	1.70	5.22
1505	0.4, 0.8	2	-	80	0.50	60	20	.70	9.64	5.80	13.82	11.12	11.20	6.50	12.86	10.40	10.46	6.16
1506	0.4, 0.8	2	0	100	0	50	50	.70	4.90	4.80	7.12	5.24	5.28	5.46	6.42	4.96	5.02	4.92
1507	0.4, 0.8	2	+	100	0.16	42	58	.70	3.78	5.08	5.74	4.08	4.08	5.44	5.02	3.72	3.72	4.94
1508	0.4, 0.8	2	-	100	0.16	58	42	.70	6.22	5.08	8.76	6.58	6.60	5.36	8.36	6.20	6.28	5.00
1509	0.4, 0.8	2	+	100	0.33	33	67	.70	2.72	5.08	4.40	3.24	3.24	5.68	3.90	2.76	2.76	5.24
1510	0.4, 0.8	2	-	100	0.33	67	33	.70	8.02	5.22	10.68	8.32	8.38	5.22	11.18	8.56	8.66	4.90
1511	0.4, 0.8	2	+	100	0.50	25	75	.70	1.96	4.74	3.40	2.16	2.20	5.88	2.42	1.44	1.46	4.82
1512	0.4, 0.8	2	-	100	0.50	75	25	.70	10.26	5.96	12.74	10.22	10.26	5.70	13.46	10.48	10.58	5.66
1513	0.4, 0.8	2	0	150	0	75	75	.70	6.04	5.86	6.52	4.90	4.94	5.06	6.98	5.12	5.16	5.18
1514	0.4, 0.8	2	+	150	0.16	63	87	.70	4.30	5.42	5.42	4.06	4.06	5.42	5.40	3.74	3.76	5.42
1515	0.4, 0.8	2	-	150	0.16	87	63	.70	6.66	5.02	8.22	6.30	6.30	5.12	8.50	6.58	6.62	5.46
1516	0.4, 0.8	2	+	150	0.33	50	100	.70	2.92	4.98	4.32	3.02	3.04	5.50	4.22	2.74	2.78	5.52
1517	0.4, 0.8	2	-	150	0.33	100	50	.70	7.42	5.02	10.48	8.02	8.08	5.22	10.40	8.04	8.06	4.94
1518	0.4, 0.8	2	+	150	0.50	37	113	.70	2.00	5.26	3.12	2.30	2.32	5.52	2.98	2.08	2.08	5.68
1519	0.4, 0.8	2	-	150	0.50	113	37	.70	9.72	5.36	12.82	10.02	10.08	5.20	12.78	9.96	10.00	5.02
1520	0.4, 0.8	2	0	200	0	100	100	.70	5.12	4.96	6.74	5.30	5.32	5.36	6.68	4.88	4.88	4.88
1521	0.4, 0.8	2	+	200	0.16	84	116	.70	3.92	5.10	5.40	3.98	4.00	5.24	5.64	4.02	4.06	5.54
1522	0.4, 0.8	2	-	200	0.16	116	84	.70	6.38	5.00	8.20	6.30	6.30	5.00	8.62	6.66	6.68	4.92
1523	0.4, 0.8	2	+	200	0.33	67	133	.70	3.02	5.30	4.02	2.84	2.86	5.28	3.74	2.88	2.90	5.10
1524	0.4, 0.8	2	-	200	0.33	133	67	.70	8.22	5.22	10.68	8.30	8.34	4.98	10.36	8.38	8.40	4.96
1525	0.4, 0.8	2	+	200	0.50	50	150	.70	1.92	5.58	3.00	2.16	2.22	5.28	3.04	2.00	2.02	5.50
1526	0.4, 0.8	2	-	200	0.50	150	50	.70	10.26	5.46	13.00	10.38	10.46	5.12	12.88	10.00	10.08	5.34
1527	0.4, 0.8	5	0	20	0	10	10	.70	5.20	3.78	7.42	5.58	5.98	4.70	7.94	6.04	6.24	5.30
1528	0.4, 0.8	5	+	20	0.16	8	12	.70	3.26	4.98	3.74	2.56	2.70	4.60	3.88	2.58	2.82	4.68
1529	0.4, 0.8	5	-	20	0.16	12	8	.70	9.40	5.34	12.30	9.78	10.24	5.16	12.04	9.40	9.78	5.20
1530	0.4, 0.8	5	+	20	0.33	7	13	.70	2.32	4.70	2.54	1.76	1.84	4.78	2.46	1.78	1.84	4.56
1531	0.4, 0.8	5	-	20	0.33	13	7	.70	11.84	6.06	15.40	12.12	12.56	5.64	15.48	12.36	12.66	5.64
1532	0.4, 0.8	5	+	20	0.50	5	15	.70	.88	5.14	1.02	.50	.60	5.06	.98	.60	.72	4.68
1533	0.4, 0.8	5	-	20	0.50	15	5	.70	17.84	8.60	23.40	19.62	20.16	7.76	23.10	19.34	19.92	8.54
1534	0.4, 0.8	5	0	40	0	20	20	.70	4.74	4.48	6.78	5.28	5.42	4.96	6.76	4.64	4.76	4.60

	D	VR	P	N	Δn	n1	n2	ε	F g	B-F g	F t	F-GG t	F-HF t	B-F t	F g x t	F-GG g x t	F-HF g x t	B-F g x t
1535	0.4, 0.8	5	+	40	0.16	17	23	.70	3.08	4.26	4.42	3.22	3.32	4.96	4.08	2.84	2.94	4.40
1536	0.4, 0.8	5	-	40	0.16	23	17	.70	7.44	4.54	10.62	8.16	8.36	5.30	10.60	8.04	8.26	5.36
1537	0.4, 0.8	5	+	40	0.33	13	27	.70	1.22	4.26	2.10	1.42	1.46	4.84	2.00	1.30	1.36	4.92
1538	0.4, 0.8	5	-	40	0.33	27	13	.70	12.20	5.10	16.40	13.22	13.54	5.56	17.04	13.52	13.90	5.18
1539	0.4, 0.8	5	+	40	0.50	10	30	.70	.66	4.36	1.14	.68	.72	5.22	.90	.50	.52	5.26
1540	0.4, 0.8	5	-	40	0.50	30	10	.70	16.58	6.44	21.78	18.38	18.64	6.88	22.82	19.40	19.82	6.90
1541	0.4, 0.8	5	0	60	0	30	30	.70	4.40	4.22	7.46	5.30	5.36	5.24	7.18	5.30	5.36	5.02
1542	0.4, 0.8	5	+	60	0.16	25	35	.70	3.06	4.56	4.36	3.18	3.28	5.28	4.18	2.88	2.96	5.34
1543	0.4, 0.8	5	-	60	0.16	35	25	.70	7.84	4.76	10.68	8.50	8.62	5.28	10.68	8.08	8.20	5.18
1544	0.4, 0.8	5	+	60	0.33	20	40	.70	1.56	5.06	2.28	1.44	1.50	4.92	1.90	1.30	1.32	4.80
1545	0.4, 0.8	5	-	60	0.33	40	20	.70	11.96	5.34	16.32	13.12	13.24	5.72	16.32	13.18	13.28	5.14
1546	0.4, 0.8	5	+	60	0.50	15	45	.70	.64	4.90	.98	.58	.60	5.10	.82	.54	.54	4.82
1547	0.4, 0.8	5	-	60	0.50	45	15	.70	16.68	6.00	22.38	19.06	19.26	5.74	22.02	18.90	19.00	5.84
1548	0.4, 0.8	5	0	80	0	40	40	.70	4.78	4.50	7.18	5.24	5.24	5.32	6.78	4.98	5.10	4.72
1549	0.4, 0.8	5	+	80	0.16	34	46	.70	2.80	4.42	4.28	3.18	3.22	5.24	4.32	2.96	2.98	5.02
1550	0.4, 0.8	5	-	80	0.16	46	34	.70	7.34	4.64	10.60	8.36	8.42	5.42	10.02	7.68	7.84	5.04
1551	0.4, 0.8	5	+	80	0.33	27	53	.70	1.38	4.34	2.44	1.66	1.72	5.50	1.96	1.02	1.06	5.12
1552	0.4, 0.8	5	-	80	0.33	53	27	.70	11.64	5.06	16.54	13.10	13.32	5.50	15.60	12.52	12.62	5.34
1553	0.4, 0.8	5	+	80	0.50	20	60	.70	.64	5.48	1.06	.68	.70	6.02	.62	.34	.34	4.68
1554	0.4, 0.8	5	-	80	0.50	60	20	.70	16.18	5.42	22.98	19.50	19.66	6.06	22.20	19.04	19.18	5.98
1555	0.4, 0.8	5	0	100	0	50	50	.70	5.10	4.84	7.10	5.36	5.38	5.22	6.74	4.86	4.86	4.70
1556	0.4, 0.8	5	+	100	0.16	42	58	.70	2.76	4.70	4.38	2.98	3.06	5.40	3.84	2.72	2.76	4.78
1557	0.4, 0.8	5	-	100	0.16	58	42	.70	7.88	5.04	11.08	8.58	8.66	5.24	10.76	8.04	8.18	5.08
1558	0.4, 0.8	5	+	100	0.33	33	67	.70	1.12	4.52	2.22	1.50	1.54	5.60	2.06	1.20	1.20	4.82
1559	0.4, 0.8	5	-	100	0.33	67	33	.70	12.14	5.22	16.22	13.22	13.32	5.26	16.74	13.46	13.56	5.34
1560	0.4, 0.8	5	+	100	0.50	25	75	.70	.60	4.52	.84	.42	.44	5.46	.54	.26	.28	5.02
1561	0.4, 0.8	5	-	100	0.50	75	25	.70	17.06	5.84	22.04	18.60	18.72	5.64	21.80	18.74	18.76	5.94
1562	0.4, 0.8	5	0	150	0	75	75	.70	5.84	5.94	6.70	4.94	4.96	5.08	6.76	5.12	5.16	5.00
1563	0.4, 0.8	5	+	150	0.16	63	87	.70	3.46	5.38	3.96	2.82	2.84	4.92	4.14	2.86	2.92	5.30
1564	0.4, 0.8	5	-	150	0.16	87	63	.70	8.24	5.14	10.28	7.90	7.94	5.14	10.74	8.28	8.36	5.28
1565	0.4, 0.8	5	+	150	0.33	50	100	.70	1.42	5.12	2.00	1.40	1.40	5.38	2.10	1.32	1.34	5.40
1566	0.4, 0.8	5	-	150	0.33	100	50	.70	11.40	5.12	15.74	12.26	12.38	5.10	15.30	12.18	12.18	5.08

	D	VR	P	N	Δn	n1	n2	ε	F g	B-F g	F t	F-GG t	F-HF t	B-F t	F g x t	F-GG g x t	F-HF g x t	B-F g x t
1567	0.4, 0.8	5	+	150	0.50	37	113	.70	.46	5.14	.90	.56	.56	5.56	.88	.58	.60	5.52
1568	0.4, 0.8	5	-	150	0.50	113	37	.70	17.02	5.14	22.24	18.74	18.80	5.00	22.28	18.36	18.40	4.92
1569	0.4, 0.8	5	0	200	0	100	100	.70	5.18	5.26	6.82	5.36	5.40	5.34	6.46	4.56	4.58	4.76
1570	0.4, 0.8	5	+	200	0.16	84	116	.70	2.80	5.16	4.24	3.10	3.12	5.18	4.08	2.64	2.64	5.54
1571	0.4, 0.8	5	-	200	0.16	116	84	.70	8.22	4.88	10.46	7.76	7.78	4.98	10.36	8.26	8.26	4.92
1572	0.4, 0.8	5	+	200	0.33	67	133	.70	1.86	4.98	2.12	1.34	1.34	5.32	1.94	1.36	1.36	5.06
1573	0.4, 0.8	5	-	200	0.33	133	67	.70	12.42	5.00	15.50	12.68	12.72	4.86	15.42	12.52	12.54	4.88
1574	0.4, 0.8	5	+	200	0.50	50	150	.70	.56	4.98	.88	.48	.48	5.24	.64	.42	.42	5.68
1575	0.4, 0.8	5	-	200	0.50	150	50	.70	17.66	5.40	22.24	18.54	18.58	5.20	22.24	18.64	18.68	5.30
1576	0.4, 0.8	1	0	20	0	10	10	.80	4.34	3.86	5.66	4.64	5.00	4.44	6.28	4.92	5.30	4.58
1577	0.4, 0.8	1	0	20	0.16	8	12	.80	4.80	4.42	5.42	4.32	4.62	4.32	5.92	4.72	5.08	4.58
1578	0.4, 0.8	1	0	20	0.33	7	13	.80	4.88	5.22	5.58	4.38	4.78	5.10	5.92	4.68	5.04	5.50
1579	0.4, 0.8	1	0	20	0.50	5	15	.80	4.60	6.58	5.68	4.44	4.76	6.52	5.96	4.90	5.20	6.90
1580	0.4, 0.8	1	0	40	0	20	20	.80	5.00	4.94	5.58	4.40	4.62	4.54	6.26	5.00	5.18	4.80
1581	0.4, 0.8	1	0	40	0.16	17	23	.80	5.14	5.14	5.40	4.06	4.28	4.30	5.92	4.64	4.78	4.94
1582	0.4, 0.8	1	0	40	0.33	13	27	.80	4.48	4.88	5.52	4.64	4.76	4.74	6.02	4.96	5.10	5.42
1583	0.4, 0.8	1	0	40	0.50	10	30	.80	4.42	5.48	5.44	4.18	4.42	5.70	6.30	5.18	5.34	6.78
1584	0.4, 0.8	1	0	60	0	30	30	.80	5.38	5.36	5.86	4.84	4.98	4.98	5.78	4.52	4.66	4.56
1585	0.4, 0.8	1	0	60	0.16	25	35	.80	4.94	5.22	5.92	4.78	4.82	5.00	5.88	4.68	4.80	4.92
1586	0.4, 0.8	1	0	60	0.33	20	40	.80	5.12	5.56	5.78	4.56	4.70	4.94	6.48	5.56	5.70	5.82
1587	0.4, 0.8	1	0	60	0.50	15	45	.80	5.28	6.14	5.96	4.56	4.70	5.18	5.78	4.96	5.00	5.48
1588	0.4, 0.8	1	0	80	0	40	40	.80	5.12	4.96	6.46	5.14	5.32	5.34	5.46	4.48	4.56	4.62
1589	0.4, 0.8	1	0	80	0.16	34	46	.80	4.98	5.16	6.26	5.00	5.18	5.32	5.84	4.90	4.96	5.08
1590	0.4, 0.8	1	0	80	0.33	27	53	.80	5.38	5.48	5.96	4.84	4.92	4.98	5.26	4.28	4.30	4.66
1591	0.4, 0.8	1	0	80	0.50	20	60	.80	5.32	6.12	6.30	5.12	5.28	5.76	6.06	4.98	5.04	5.56
1592	0.4, 0.8	1	0	100	0	50	50	.80	5.38	5.34	5.80	4.54	4.58	4.78	5.78	4.76	4.90	4.96
1593	0.4, 0.8	1	0	100	0.16	42	58	.80	4.92	4.72	5.96	4.84	4.94	4.88	5.64	4.46	4.48	4.72
1594	0.4, 0.8	1	0	100	0.33	33	67	.80	5.40	5.60	5.84	4.48	4.54	4.88	5.86	4.78	4.82	5.02
1595	0.4, 0.8	1	0	100	0.50	25	75	.80	5.10	5.62	6.00	4.32	4.46	5.20	5.22	4.26	4.38	4.78
1596	0.4, 0.8	1	0	150	0	75	75	.80	5.50	5.56	6.32	5.10	5.12	5.40	6.92	5.64	5.66	5.92
1597	0.4, 0.8	1	0	150	0.16	63	87	.80	5.74	5.80	6.44	5.24	5.24	5.56	6.24	4.94	4.96	5.18
1598	0.4, 0.8	1	0	150	0.33	50	100	.80	5.36	5.72	6.44	5.08	5.14	5.54	5.90	4.74	4.78	5.00

	D	VR	P	N	Δn	n1	n2	ε	F g	B-F g	F t	F-GG t	F-HF t	B-F t	F g x t	F-GG g x t	F-HF g x t	B-F g x t
1599	0.4, 0.8	1	0	150	0.50	37	113	.80	5.24	5.74	5.78	4.88	4.90	5.50	5.78	4.56	4.60	5.34
1600	0.4, 0.8	1	0	200	0	100	100	.80	5.28	5.22	6.12	5.02	5.04	4.94	6.40	5.50	5.60	5.52
1601	0.4, 0.8	1	0	200	0.16	84	116	.80	5.34	5.46	6.32	5.12	5.16	5.32	6.30	5.10	5.10	5.32
1602	0.4, 0.8	1	0	200	0.33	67	133	.80	5.64	5.66	6.40	5.12	5.18	5.62	6.44	5.20	5.24	5.54
1603	0.4, 0.8	1	0	200	0.50	50	150	.80	5.30	5.68	6.26	5.06	5.10	5.36	5.60	4.82	4.86	4.94
1604	0.4, 0.8	1.5	0	20	0	10	10	.80	4.38	4.22	5.82	4.52	4.88	4.36	6.54	4.96	5.54	4.70
1605	0.4, 0.8	1.5	+	20	0.16	8	12	.80	4.20	4.38	4.54	3.56	3.86	4.20	4.86	3.70	4.08	4.52
1606	0.4, 0.8	1.5	-	20	0.16	12	8	.80	5.82	4.88	7.22	5.76	6.22	4.96	7.84	6.10	6.42	5.04
1607	0.4, 0.8	1.5	+	20	0.33	7	13	.80	3.86	5.16	4.08	3.04	3.38	4.70	4.52	3.48	3.96	5.18
1608	0.4, 0.8	1.5	-	20	0.33	13	7	.80	5.92	5.46	7.82	6.48	6.84	5.36	8.30	6.62	6.96	5.62
1609	0.4, 0.8	1.5	+	20	0.50	5	15	.80	3.00	6.12	3.36	2.56	2.80	6.30	3.80	2.78	3.16	6.14
1610	0.4, 0.8	1.5	-	20	0.50	15	5	.80	7.78	8.26	9.40	7.46	7.90	7.22	9.80	7.88	8.44	7.00
1611	0.4, 0.8	1.5	0	40	0	20	20	.80	4.76	4.76	5.94	4.64	4.86	4.56	6.08	4.60	4.80	4.58
1612	0.4, 0.8	1.5	+	40	0.16	17	23	.80	4.46	5.34	4.60	3.62	3.78	4.72	4.96	4.02	4.18	4.68
1613	0.4, 0.8	1.5	-	40	0.16	23	17	.80	5.80	5.20	6.92	5.48	5.70	5.24	6.94	5.70	5.82	5.28
1614	0.4, 0.8	1.5	+	40	0.33	13	27	.80	3.02	4.84	3.76	2.92	3.02	4.76	4.24	3.50	3.62	5.38
1615	0.4, 0.8	1.5	-	40	0.33	27	13	.80	6.68	5.64	8.00	6.60	6.76	5.32	7.10	5.58	5.76	4.76
1616	0.4, 0.8	1.5	+	40	0.50	10	30	.80	2.60	5.10	3.04	2.30	2.36	5.44	3.82	3.06	3.22	6.54
1617	0.4, 0.8	1.5	-	40	0.50	30	10	.80	7.34	6.30	8.56	7.22	7.44	5.74	8.52	7.32	7.40	5.64
1618	0.4, 0.8	1.5	0	60	0	30	30	.80	5.30	5.10	6.10	4.92	5.08	4.98	5.76	4.64	4.78	4.58
1619	0.4, 0.8	1.5	+	60	0.16	25	35	.80	4.28	4.74	5.18	4.10	4.16	4.78	4.98	3.76	3.84	4.92
1620	0.4, 0.8	1.5	-	60	0.16	35	25	.80	5.58	4.38	7.24	5.92	6.06	5.28	7.26	5.84	6.00	4.90
1621	0.4, 0.8	1.5	+	60	0.33	20	40	.80	3.86	5.34	4.18	3.36	3.46	5.02	4.88	4.06	4.16	6.00
1622	0.4, 0.8	1.5	-	60	0.33	40	20	.80	6.02	5.08	8.46	6.86	6.96	5.28	7.22	5.92	5.98	4.58
1623	0.4, 0.8	1.5	+	60	0.50	15	45	.80	3.12	5.88	3.50	2.60	2.66	5.12	3.84	3.14	3.18	5.46
1624	0.4, 0.8	1.5	-	60	0.50	45	15	.80	7.42	5.74	9.50	8.06	8.18	5.58	8.92	7.56	7.66	5.54
1625	0.4, 0.8	1.5	0	80	0	40	40	.80	5.06	5.00	6.34	5.36	5.42	5.18	5.64	4.64	4.66	4.70
1626	0.4, 0.8	1.5	+	80	0.16	34	46	.80	4.16	4.86	5.48	4.42	4.46	5.36	5.18	4.24	4.30	5.10
1627	0.4, 0.8	1.5	-	80	0.16	46	34	.80	5.42	4.66	7.32	6.02	6.06	5.34	7.04	5.50	5.56	4.82
1628	0.4, 0.8	1.5	+	80	0.33	27	53	.80	3.94	5.20	4.38	3.34	3.50	5.14	3.86	3.20	3.22	4.76
1629	0.4, 0.8	1.5	-	80	0.33	53	27	.80	6.42	4.90	8.54	7.16	7.34	5.48	8.32	6.62	6.74	5.10
1630	0.4, 0.8	1.5	+	80	0.50	20	60	.80	3.12	5.70	3.50	2.72	2.78	5.66	3.82	3.08	3.10	5.68

	D	VR	P	N	Δn	n1	n2	ε	F g	B-F g	F t	F-GG t	F-HF t	B-F t	F g x t	F-GG g x t	F-HF g x t	B-F g x t
1631	0.4, 0.8	1.5	-	80	0.50	60	20	.80	7.42	5.60	9.90	8.40	8.50	5.96	9.30	7.76	7.84	5.52
1632	0.4, 0.8	1.5	0	100	0	50	50	.80	5.26	5.32	6.16	4.64	4.68	4.82	6.10	5.02	5.06	5.16
1633	0.4, 0.8	1.5	+	100	0.16	42	58	.80	4.20	4.82	4.98	3.96	4.00	4.86	4.64	3.74	3.80	4.68
1634	0.4, 0.8	1.5	-	100	0.16	58	42	.80	6.10	5.46	7.04	5.64	5.72	5.02	7.14	6.04	6.16	5.40
1635	0.4, 0.8	1.5	+	100	0.33	33	67	.80	3.66	5.34	4.08	3.32	3.32	4.72	4.20	3.36	3.40	5.14
1636	0.4, 0.8	1.5	-	100	0.33	67	33	.80	7.08	5.60	8.46	6.78	6.88	4.90	8.56	7.38	7.44	5.12
1637	0.4, 0.8	1.5	+	100	0.50	25	75	.80	3.12	5.48	3.10	2.58	2.58	5.10	3.18	2.50	2.54	4.84
1638	0.4, 0.8	1.5	-	100	0.50	75	25	.80	8.08	5.88	9.00	7.30	7.54	5.14	9.40	8.00	8.08	5.30
1639	0.4, 0.8	1.5	0	150	0	75	75	.80	5.64	5.64	6.46	5.18	5.24	5.38	6.98	5.54	5.60	5.74
1640	0.4, 0.8	1.5	+	150	0.16	63	87	.80	4.76	5.94	5.34	4.42	4.42	5.36	5.28	4.30	4.38	5.16
1641	0.4, 0.8	1.5	-	150	0.16	87	63	.80	5.86	5.32	7.56	6.30	6.32	5.46	7.38	6.34	6.42	5.60
1642	0.4, 0.8	1.5	+	150	0.33	50	100	.80	4.12	5.24	4.38	3.42	3.46	5.64	4.26	3.36	3.38	5.16
1643	0.4, 0.8	1.5	-	150	0.33	100	50	.80	6.32	4.60	8.64	7.16	7.24	5.60	8.48	7.22	7.26	5.32
1644	0.4, 0.8	1.5	+	150	0.50	37	113	.80	3.26	5.66	3.66	2.86	2.90	5.38	3.24	2.58	2.58	4.82
1645	0.4, 0.8	1.5	-	150	0.50	113	37	.80	7.44	5.32	10.00	8.28	8.34	5.32	9.58	8.08	8.20	5.66
1646	0.4, 0.8	1.5	0	200	0	100	100	.80	5.26	5.22	6.18	4.86	4.92	4.92	6.32	5.58	5.62	5.48
1647	0.4, 0.8	1.5	+	200	0.16	84	116	.80	4.50	5.38	5.30	3.96	3.96	5.16	5.26	4.28	4.36	5.28
1648	0.4, 0.8	1.5	-	200	0.16	116	84	.80	6.04	5.24	7.30	5.92	5.92	5.00	7.54	6.22	6.30	5.28
1649	0.4, 0.8	1.5	+	200	0.33	67	133	.80	3.80	5.30	4.50	3.48	3.52	5.32	4.54	3.62	3.66	5.36
1650	0.4, 0.8	1.5	-	200	0.33	133	67	.80	6.80	5.08	8.66	7.18	7.26	5.54	8.98	7.40	7.40	5.44
1651	0.4, 0.8	1.5	+	200	0.50	50	150	.80	3.34	5.48	3.54	2.80	2.90	5.34	3.50	2.72	2.74	5.04
1652	0.4, 0.8	1.5	-	200	0.50	150	50	.80	7.86	5.52	10.00	8.32	8.38	5.48	9.34	8.12	8.14	5.52
1653	0.4, 0.8	2	0	20	0	10	10	.80	4.50	4.08	6.00	4.70	5.08	4.64	6.60	5.14	5.54	4.86
1654	0.4, 0.8	2	+	20	0.16	8	12	.80	3.60	4.34	4.04	3.24	3.44	4.20	4.22	3.36	3.56	4.28
1655	0.4, 0.8	2	-	20	0.16	12	8	.80	6.76	5.08	8.50	6.98	7.28	5.04	8.44	6.64	6.94	5.12
1656	0.4, 0.8	2	+	20	0.33	7	13	.80	3.20	4.30	3.24	2.44	2.80	4.20	3.60	2.94	3.14	4.36
1657	0.4, 0.8	2	-	20	0.33	13	7	.80	7.30	5.56	9.10	7.58	7.88	5.28	9.74	8.10	8.52	5.62
1658	0.4, 0.8	2	+	20	0.50	5	15	.80	2.16	5.58	2.36	1.76	2.00	5.94	2.48	1.96	2.08	5.78
1659	0.4, 0.8	2	-	20	0.50	15	5	.80	10.04	8.28	12.66	10.26	10.96	7.54	12.34	10.54	10.92	7.18
1660	0.4, 0.8	2	0	40	0	20	20	.80	4.94	4.72	5.98	4.86	4.94	4.72	6.04	4.82	5.00	4.72
1661	0.4, 0.8	2	+	40	0.16	17	23	.80	4.04	5.22	4.18	3.36	3.54	4.68	4.48	3.52	3.68	4.68
1662	0.4, 0.8	2	-	40	0.16	23	17	.80	6.44	5.06	7.42	6.10	6.28	5.22	7.80	6.32	6.50	5.10

	D	VR	P	N	Δn	n1	n2	ε	F g	B-F g	F t	F-GG t	F-HF t	B-F t	F g x t	F-GG g x t	F-HF g x t	B-F g x t
1663	0.4, 0.8	2	+	40	0.33	13	27	.80	2.26	4.72	2.80	2.20	2.28	4.58	3.36	2.72	2.76	5.44
1664	0.4, 0.8	2	-	40	0.33	27	13	.80	8.04	5.66	9.72	8.28	8.38	5.48	9.14	7.48	7.78	4.90
1665	0.4, 0.8	2	+	40	0.50	10	30	.80	1.74	4.78	2.08	1.48	1.58	5.26	2.60	2.08	2.12	6.26
1666	0.4, 0.8	2	-	40	0.50	30	10	.80	9.68	6.48	11.50	9.56	9.78	5.98	11.26	9.70	9.98	5.76
1667	0.4, 0.8	2	0	60	0	30	30	.80	5.20	5.12	6.42	5.12	5.34	5.14	5.94	4.92	5.00	4.70
1668	0.4, 0.8	2	+	60	0.16	25	35	.80	3.86	4.66	4.58	3.68	3.84	4.94	4.54	3.42	3.46	4.94
1669	0.4, 0.8	2	-	60	0.16	35	25	.80	6.22	4.32	8.22	6.68	6.78	5.24	7.90	6.60	6.78	4.94
1670	0.4, 0.8	2	+	60	0.33	20	40	.80	2.98	5.36	3.34	2.64	2.66	4.94	3.92	3.02	3.06	5.76
1671	0.4, 0.8	2	-	60	0.33	40	20	.80	7.24	5.08	10.32	8.70	8.94	5.54	9.08	7.62	7.74	4.66
1672	0.4, 0.8	2	+	60	0.50	15	45	.80	2.08	5.64	2.30	1.78	1.86	5.06	2.66	2.08	2.12	5.34
1673	0.4, 0.8	2	-	60	0.50	45	15	.80	9.66	5.58	12.56	10.82	11.00	5.50	11.84	10.00	10.16	5.46
1674	0.4, 0.8	2	0	80	0	40	40	.80	5.12	4.88	6.36	5.00	5.06	4.96	5.68	4.70	4.78	4.72
1675	0.4, 0.8	2	+	80	0.16	34	46	.80	3.62	4.78	5.06	4.02	4.04	5.32	4.74	3.94	4.00	5.04
1676	0.4, 0.8	2	-	80	0.16	46	34	.80	6.02	4.78	8.10	6.56	6.66	5.24	7.66	6.30	6.34	4.96
1677	0.4, 0.8	2	+	80	0.33	27	53	.80	3.02	5.12	3.60	2.86	2.94	5.22	3.08	2.50	2.52	4.80
1678	0.4, 0.8	2	-	80	0.33	53	27	.80	7.42	4.98	10.36	8.62	8.76	5.66	10.24	8.42	8.54	5.10
1679	0.4, 0.8	2	+	80	0.50	20	60	.80	2.38	5.42	2.40	1.60	1.72	5.46	2.50	1.80	1.82	5.54
1680	0.4, 0.8	2	-	80	0.50	60	20	.80	9.44	5.58	13.06	11.26	11.40	6.02	12.34	10.52	10.64	5.50
1681	0.4, 0.8	2	0	100	0	50	50	.80	5.06	4.96	6.12	4.74	4.80	4.86	6.14	5.12	5.14	5.24
1682	0.4, 0.8	2	+	100	0.16	42	58	.80	3.74	4.78	4.38	3.64	3.66	4.86	4.04	3.34	3.40	4.54
1683	0.4, 0.8	2	-	100	0.16	58	42	.80	6.54	5.30	7.90	6.22	6.32	4.74	7.72	6.44	6.52	5.48
1684	0.4, 0.8	2	+	100	0.33	33	67	.80	2.48	5.10	3.26	2.48	2.52	4.76	3.28	2.42	2.52	5.04
1685	0.4, 0.8	2	-	100	0.33	67	33	.80	8.28	6.02	10.42	8.52	8.56	5.40	9.96	8.74	8.82	5.76
1686	0.4, 0.8	2	+	100	0.50	25	75	.80	2.02	5.20	2.28	1.62	1.68	4.94	2.12	1.66	1.66	4.84
1687	0.4, 0.8	2	-	100	0.50	75	25	.80	10.48	6.12	11.94	10.16	10.30	5.54	12.40	10.64	10.74	5.48
1688	0.4, 0.8	2	0	150	0	75	75	.80	5.54	5.60	6.40	5.24	5.30	5.48	6.82	5.68	5.76	5.88
1689	0.4, 0.8	2	+	150	0.16	63	87	.80	4.36	5.60	4.86	3.76	3.80	5.38	4.92	3.74	3.78	5.46
1690	0.4, 0.8	2	-	150	0.16	87	63	.80	6.16	5.16	8.34	6.84	6.92	5.62	7.96	6.94	6.96	5.56
1691	0.4, 0.8	2	+	150	0.33	50	100	.80	3.20	5.28	3.20	2.58	2.60	5.52	3.28	2.62	2.66	5.08
1692	0.4, 0.8	2	-	150	0.33	100	50	.80	7.82	4.66	10.18	8.72	8.76	5.50	10.20	8.60	8.66	5.28
1693	0.4, 0.8	2	+	150	0.50	37	113	.80	2.22	5.48	2.50	1.88	1.90	5.62	2.24	1.58	1.60	4.80
1694	0.4, 0.8	2	-	150	0.50	113	37	.80	9.70	5.42	12.76	11.18	11.26	5.44	12.28	10.76	10.78	5.62

	D	VR	P	N	Δn	n1	n2	ε	F g	B-F g	F t	F-GG t	F-HF t	B-F t	F g x t	F-GG g x t	F-HF g x t	B-F g x t
1695	0.4, 0.8	2	0	200	0	100	100	.80	5.06	5.28	6.10	4.92	4.96	5.10	6.40	5.56	5.58	5.42
1696	0.4, 0.8	2	+	200	0.16	84	116	.80	4.06	5.16	4.52	3.58	3.58	5.22	4.74	3.96	3.96	5.32
1697	0.4, 0.8	2	-	200	0.16	116	84	.80	6.54	5.44	7.98	6.66	6.70	5.40	8.46	6.94	7.00	5.44
1698	0.4, 0.8	2	+	200	0.33	67	133	.80	2.86	5.38	3.36	2.54	2.56	5.54	3.56	2.90	2.92	5.40
1699	0.4, 0.8	2	-	200	0.33	133	67	.80	7.90	5.08	10.48	8.90	8.90	5.48	10.98	9.36	9.38	5.86
1700	0.4, 0.8	2	+	200	0.50	50	150	.80	2.34	5.34	2.30	1.84	1.84	5.44	2.32	1.50	1.54	5.02
1701	0.4, 0.8	2	-	200	0.50	150	50	.80	9.90	5.52	13.06	11.24	11.32	5.44	12.76	10.62	10.76	5.52
1702	0.4, 0.8	5	0	20	0	10	10	.80	5.24	4.38	6.64	5.30	5.66	4.52	6.68	5.28	5.50	4.70
1703	0.4, 0.8	5	+	20	0.16	8	12	.80	3.04	4.36	3.10	2.42	2.66	4.40	3.42	2.60	2.78	4.42
1704	0.4, 0.8	5	-	20	0.16	12	8	.80	9.54	5.28	11.98	9.84	10.42	5.06	11.68	9.50	10.00	5.16
1705	0.4, 0.8	5	+	20	0.33	7	13	.80	2.24	4.62	1.88	1.42	1.56	4.44	2.16	1.60	1.68	4.20
1706	0.4, 0.8	5	-	20	0.33	13	7	.80	11.48	5.84	15.22	12.32	13.00	5.26	15.32	12.64	13.10	5.62
1707	0.4, 0.8	5	+	20	0.50	5	15	.80	1.02	4.78	.84	.60	.64	4.82	.76	.46	.64	5.08
1708	0.4, 0.8	5	-	20	0.50	15	5	.80	18.02	8.74	22.78	19.78	20.38	7.26	22.86	19.66	20.22	7.36
1709	0.4, 0.8	5	0	40	0	20	20	.80	5.22	4.78	6.40	4.90	5.18	4.80	5.90	4.76	4.96	4.52
1710	0.4, 0.8	5	+	40	0.16	17	23	.80	3.42	4.94	3.68	2.76	2.82	4.92	3.32	2.66	2.70	4.62
1711	0.4, 0.8	5	-	40	0.16	23	17	.80	7.88	5.08	9.58	8.06	8.24	5.34	9.84	8.08	8.32	4.98
1712	0.4, 0.8	5	+	40	0.33	13	27	.80	1.30	4.82	1.36	1.00	1.04	4.58	1.84	1.46	1.50	4.76
1713	0.4, 0.8	5	-	40	0.33	27	13	.80	12.34	5.58	15.28	13.20	13.38	5.54	14.94	12.94	13.18	5.12
1714	0.4, 0.8	5	+	40	0.50	10	30	.80	.70	4.66	.38	.30	.32	4.76	.76	.56	.60	5.48
1715	0.4, 0.8	5	-	40	0.50	30	10	.80	16.92	6.30	21.02	18.78	19.02	6.24	21.62	19.12	19.52	5.82
1716	0.4, 0.8	5	0	60	0	30	30	.80	5.00	4.78	6.92	5.78	6.00	5.54	6.14	4.90	4.94	4.74
1717	0.4, 0.8	5	+	60	0.16	25	35	.80	2.78	4.60	3.70	2.94	3.00	5.02	3.50	2.72	2.82	5.02
1718	0.4, 0.8	5	-	60	0.16	35	25	.80	7.48	4.26	10.88	8.92	9.02	5.12	10.68	8.98	9.16	4.66
1719	0.4, 0.8	5	+	60	0.33	20	40	.80	1.70	5.14	1.76	1.34	1.34	5.12	2.02	1.36	1.40	5.50
1720	0.4, 0.8	5	-	60	0.33	40	20	.80	11.64	4.78	15.78	13.74	13.90	5.10	14.70	12.40	12.66	5.08
1721	0.4, 0.8	5	+	60	0.50	15	45	.80	.88	5.02	.60	.40	.44	5.00	.78	.66	.66	5.32
1722	0.4, 0.8	5	-	60	0.50	45	15	.80	16.38	5.60	22.66	19.94	20.28	5.26	21.48	19.00	19.22	5.18
1723	0.4, 0.8	5	0	80	0	40	40	.80	5.12	4.80	6.52	5.16	5.28	5.22	6.08	5.00	5.10	4.86
1724	0.4, 0.8	5	+	80	0.16	34	46	.80	2.78	4.46	3.92	3.10	3.16	5.68	3.90	3.22	3.30	5.64
1725	0.4, 0.8	5	-	80	0.16	46	34	.80	7.74	4.76	10.14	8.64	8.76	5.22	9.44	8.02	8.08	5.18
1726	0.4, 0.8	5	+	80	0.33	27	53	.80	1.56	4.64	1.64	1.20	1.24	5.44	1.82	1.36	1.44	4.78

	D	VR	P	N	Δn	n1	n2	ε	F g	B-F g	F t	F-GG t	F-HF t	B-F t	F g x t	F-GG g x t	F-HF g x t	B-F g x t
1727	0.4, 0.8	5	-	80	0.33	53	27	.80	11.28	4.96	15.28	13.44	13.64	5.58	15.38	13.38	13.48	5.10
1728	0.4, 0.8	5	+	80	0.50	20	60	.80	.82	5.14	.70	.54	.56	5.54	.52	.34	.36	5.54
1729	0.4, 0.8	5	-	80	0.50	60	20	.80	16.16	5.20	22.76	20.26	20.48	5.78	22.30	19.90	20.12	5.26
1730	0.4, 0.8	5	0	100	0	50	50	.80	5.00	4.96	6.14	4.86	4.98	4.76	6.16	5.20	5.32	5.20
1731	0.4, 0.8	5	+	100	0.16	42	58	.80	2.96	4.70	3.20	2.44	2.48	4.64	3.44	2.82	2.86	4.76
1732	0.4, 0.8	5	-	100	0.16	58	42	.80	7.98	5.16	9.90	8.12	8.32	4.72	10.24	8.82	8.92	5.24
1733	0.4, 0.8	5	+	100	0.33	33	67	.80	1.12	4.50	1.60	1.04	1.06	4.84	1.58	1.22	1.22	4.86
1734	0.4, 0.8	5	-	100	0.33	67	33	.80	12.60	6.00	15.78	13.78	13.86	5.38	15.40	13.08	13.30	5.54
1735	0.4, 0.8	5	+	100	0.50	25	75	.80	.60	4.66	.58	.40	.40	4.72	.54	.34	.34	4.90
1736	0.4, 0.8	5	-	100	0.50	75	25	.80	18.12	6.04	21.76	19.20	19.36	5.52	21.86	19.62	19.78	5.06
1737	0.4, 0.8	5	0	150	0	75	75	.80	5.38	5.48	6.58	5.34	5.36	5.56	6.60	5.46	5.48	5.72
1738	0.4, 0.8	5	+	150	0.16	63	87	.80	3.36	5.18	3.68	2.72	2.78	5.30	3.60	2.78	2.86	5.44
1739	0.4, 0.8	5	-	150	0.16	87	63	.80	7.62	5.08	10.44	8.90	9.00	5.50	10.04	8.60	8.64	5.50
1740	0.4, 0.8	5	+	150	0.33	50	100	.80	1.60	5.08	1.54	1.06	1.06	5.38	1.64	1.20	1.20	4.78
1741	0.4, 0.8	5	-	150	0.33	100	50	.80	11.32	4.82	15.48	13.28	13.40	5.16	14.98	13.34	13.40	5.22
1742	0.4, 0.8	5	+	150	0.50	37	113	.80	.54	5.02	.66	.36	.36	5.54	.54	.38	.38	4.74
1743	0.4, 0.8	5	-	150	0.50	113	37	.80	16.90	5.62	21.54	19.54	19.62	5.34	21.70	19.14	19.26	5.26
1744	0.4, 0.8	5	0	200	0	100	100	.80	4.98	4.86	6.30	5.10	5.14	5.08	6.08	5.38	5.40	5.32
1745	0.4, 0.8	5	+	200	0.16	84	116	.80	2.98	4.78	3.34	2.84	2.84	5.02	3.54	2.74	2.76	5.20
1746	0.4, 0.8	5	-	200	0.16	116	84	.80	7.96	5.36	10.10	8.68	8.70	5.22	10.56	9.08	9.10	5.78
1747	0.4, 0.8	5	+	200	0.33	67	133	.80	1.66	5.08	1.72	1.32	1.32	5.06	1.64	1.20	1.20	5.50
1748	0.4, 0.8	5	-	200	0.33	133	67	.80	11.82	5.24	16.02	13.82	13.90	5.32	16.44	14.28	14.32	5.54
1749	0.4, 0.8	5	+	200	0.50	50	150	.80	.58	5.14	.72	.38	.38	5.20	.48	.32	.34	4.98
1750	0.4, 0.8	5	-	200	0.50	150	50	.80	17.00	5.38	22.40	20.10	20.14	5.76	23.24	20.70	20.80	5.44
1751	0.4, 0.8	1	0	20	0	10	10	.90	4.28	4.04	5.40	4.42	4.84	4.32	5.84	4.72	5.12	4.44
1752	0.4, 0.8	1	0	20	0.16	8	12	.90	4.64	4.18	5.32	4.28	4.72	4.30	5.44	4.52	4.96	4.72
1753	0.4, 0.8	1	0	20	0.33	7	13	.90	5.18	5.10	5.38	4.46	4.80	4.40	5.56	4.72	5.12	4.76
1754	0.4, 0.8	1	0	20	0.50	5	15	.90	5.02	7.30	5.48	4.54	5.12	6.78	5.48	4.70	5.06	6.92
1755	0.4, 0.8	1	0	40	0	20	20	.90	4.94	4.88	5.66	4.96	5.12	4.88	5.26	4.48	4.56	4.66
1756	0.4, 0.8	1	0	40	0.16	17	23	.90	4.80	4.96	5.52	4.90	5.10	4.92	4.90	4.24	4.46	4.30
1757	0.4, 0.8	1	0	40	0.33	13	27	.90	4.64	5.00	5.26	4.50	4.70	5.00	5.28	4.56	4.66	5.06
1758	0.4, 0.8	1	0	40	0.50	10	30	.90	4.32	5.78	5.00	4.34	4.74	5.86	5.54	4.82	5.04	6.30

	D	VR	P	N	Δn	n1	n2	ε	F g	B-F g	F t	F-GG t	F-HF t	B-F t	F g x t	F-GG g x t	F-HF g x t	B-F g x t
1759	0.4, 0.8	1	0	60	0	30	30	.90	4.72	4.68	5.38	4.62	4.72	4.68	5.38	4.78	4.90	4.90
1760	0.4, 0.8	1	0	60	0.16	25	35	.90	4.78	4.64	5.22	4.62	4.76	4.72	5.42	4.76	4.84	4.92
1761	0.4, 0.8	1	0	60	0.33	20	40	.90	5.30	5.52	5.06	4.42	4.60	4.74	4.88	4.30	4.44	4.60
1762	0.4, 0.8	1	0	60	0.50	15	45	.90	4.78	5.80	5.24	4.68	4.78	5.06	5.40	4.94	5.04	5.46
1763	0.4, 0.8	1	0	80	0	40	40	.90	4.76	4.70	6.30	5.46	5.62	5.52	5.52	4.96	5.00	4.78
1764	0.4, 0.8	1	0	80	0.16	34	46	.90	4.70	4.86	5.92	5.42	5.50	5.44	5.46	4.84	4.96	5.00
1765	0.4, 0.8	1	0	80	0.33	27	53	.90	4.90	5.18	6.00	5.46	5.50	5.64	5.16	4.50	4.64	4.94
1766	0.4, 0.8	1	0	80	0.50	20	60	.90	5.58	5.94	5.74	5.04	5.14	5.76	5.14	4.58	4.68	5.18
1767	0.4, 0.8	1	0	100	0	50	50	.90	5.34	5.46	5.48	4.78	4.88	5.00	5.10	4.54	4.66	4.64
1768	0.4, 0.8	1	0	100	0.16	42	58	.90	4.80	4.86	5.58	5.02	5.10	5.22	5.32	4.66	4.72	4.86
1769	0.4, 0.8	1	0	100	0.33	33	67	.90	5.00	5.28	5.76	4.96	5.14	5.50	5.42	4.94	5.06	5.16
1770	0.4, 0.8	1	0	100	0.50	25	75	.90	4.92	5.30	5.46	4.78	4.90	5.60	5.20	4.34	4.44	4.84
1771	0.4, 0.8	1	0	150	0	75	75	.90	6.02	5.92	5.90	5.32	5.38	5.40	6.02	5.38	5.44	5.56
1772	0.4, 0.8	1	0	150	0.16	63	87	.90	5.86	5.70	5.86	5.40	5.48	5.48	5.56	4.82	4.88	5.28
1773	0.4, 0.8	1	0	150	0.33	50	100	.90	5.26	5.52	6.00	5.36	5.48	5.60	5.28	4.54	4.60	4.94
1774	0.4, 0.8	1	0	150	0.50	37	113	.90	5.08	5.72	5.80	5.26	5.34	6.04	5.28	4.82	4.86	5.34
1775	0.4, 0.8	1	0	200	0	100	100	.90	5.26	5.22	5.86	5.32	5.34	5.30	5.62	5.20	5.24	5.32
1776	0.4, 0.8	1	0	200	0.16	84	116	.90	5.18	5.26	6.00	5.38	5.42	5.46	5.72	5.18	5.22	5.34
1777	0.4, 0.8	1	0	200	0.33	67	133	.90	5.36	5.58	5.94	5.44	5.46	5.66	5.68	5.20	5.24	5.24
1778	0.4, 0.8	1	0	200	0.50	50	150	.90	4.98	5.30	5.74	5.16	5.18	5.38	5.00	4.60	4.62	5.06
1779	0.4, 0.8	1.5	0	20	0	10	10	.90	4.14	3.84	5.54	4.56	4.98	4.62	5.64	4.64	5.14	4.40
1780	0.4, 0.8	1.5	+	20	0.16	8	12	.90	3.80	4.40	4.20	3.44	3.70	4.12	4.34	3.46	3.74	4.50
1781	0.4, 0.8	1.5	-	20	0.16	12	8	.90	5.64	5.10	6.50	5.50	5.82	4.62	6.40	5.42	5.68	4.30
1782	0.4, 0.8	1.5	+	20	0.33	7	13	.90	4.12	5.22	3.76	3.04	3.38	4.54	4.20	3.26	3.76	5.14
1783	0.4, 0.8	1.5	-	20	0.33	13	7	.90	6.22	5.72	7.14	6.04	6.58	5.12	7.48	6.28	6.86	5.10
1784	0.4, 0.8	1.5	+	20	0.50	5	15	.90	3.20	6.50	2.82	2.36	2.46	5.72	3.06	2.46	2.70	6.02
1785	0.4, 0.8	1.5	-	20	0.50	15	5	.90	7.68	7.96	9.00	7.86	8.28	6.86	9.58	8.28	8.88	7.22
1786	0.4, 0.8	1.5	0	40	0	20	20	.90	5.06	4.80	5.88	5.24	5.42	4.74	5.26	4.58	4.80	4.62
1787	0.4, 0.8	1.5	+	40	0.16	17	23	.90	4.32	4.76	4.66	4.08	4.22	4.88	4.20	3.54	3.70	4.34
1788	0.4, 0.8	1.5	-	40	0.16	23	17	.90	5.44	4.98	7.10	6.18	6.50	5.20	6.10	5.42	5.58	4.72
1789	0.4, 0.8	1.5	+	40	0.33	13	27	.90	3.16	4.86	3.48	2.98	3.18	4.94	3.58	3.06	3.22	4.78
1790	0.4, 0.8	1.5	-	40	0.33	27	13	.90	6.62	5.18	8.56	7.44	7.72	5.52	6.90	6.06	6.28	5.02

	D	VR	P	N	Δn	n1	n2	ε	F g	B-F g	F t	F-GG t	F-HF t	B-F t	F g x t	F-GG g x t	F-HF g x t	B-F g x t
1791	0.4, 0.8	1.5	+	40	0.50	10	30	.90	2.70	5.32	2.66	2.36	2.48	5.54	3.04	2.68	2.78	5.84
1792	0.4, 0.8	1.5	-	40	0.50	30	10	.90	7.22	6.42	9.06	8.06	8.28	6.22	8.38	7.50	7.74	5.98
1793	0.4, 0.8	1.5	0	60	0	30	30	.90	4.50	4.38	5.34	4.68	4.78	4.78	5.42	4.78	5.02	5.02
1794	0.4, 0.8	1.5	+	60	0.16	25	35	.90	3.96	4.58	4.30	3.84	3.98	4.72	4.60	4.04	4.20	4.80
1795	0.4, 0.8	1.5	-	60	0.16	35	25	.90	5.50	4.62	6.62	5.86	6.06	5.22	6.50	5.86	6.08	5.32
1796	0.4, 0.8	1.5	+	60	0.33	20	40	.90	3.66	5.36	3.48	3.12	3.18	4.64	3.74	3.36	3.46	4.80
1797	0.4, 0.8	1.5	-	60	0.33	40	20	.90	6.24	4.92	8.08	7.10	7.28	5.14	7.46	6.52	6.62	5.08
1798	0.4, 0.8	1.5	+	60	0.50	15	45	.90	3.20	5.42	2.84	2.46	2.62	4.86	3.00	2.52	2.66	5.34
1799	0.4, 0.8	1.5	-	60	0.50	45	15	.90	7.22	5.90	9.50	8.58	8.82	5.60	8.64	7.88	8.00	5.80
1800	0.4, 0.8	1.5	0	80	0	40	40	.90	4.48	4.58	6.20	5.42	5.58	5.50	5.52	4.90	4.96	4.84
1801	0.4, 0.8	1.5	+	80	0.16	34	46	.90	4.00	4.60	5.20	4.62	4.72	5.56	4.90	4.40	4.44	5.24
1802	0.4, 0.8	1.5	-	80	0.16	46	34	.90	5.60	4.68	7.24	6.56	6.68	5.82	6.38	5.76	5.86	4.82
1803	0.4, 0.8	1.5	+	80	0.33	27	53	.90	3.36	5.30	4.30	3.72	3.76	5.74	3.68	3.20	3.30	4.90
1804	0.4, 0.8	1.5	-	80	0.33	53	27	.90	6.10	4.76	8.56	7.58	7.70	5.52	7.52	6.94	7.08	5.26
1805	0.4, 0.8	1.5	+	80	0.50	20	60	.90	3.16	5.82	3.30	3.00	3.06	5.74	2.90	2.58	2.62	5.24
1806	0.4, 0.8	1.5	-	80	0.50	60	20	.90	7.56	5.64	9.64	8.74	8.88	5.86	9.04	8.14	8.34	5.62
1807	0.4, 0.8	1.5	0	100	0	50	50	.90	5.06	5.02	5.30	4.74	4.82	4.86	5.20	4.56	4.68	4.70
1808	0.4, 0.8	1.5	+	100	0.16	42	58	.90	4.18	5.02	4.50	4.16	4.16	5.00	4.42	3.82	3.92	4.92
1809	0.4, 0.8	1.5	-	100	0.16	58	42	.90	6.16	5.56	6.34	5.56	5.72	4.78	6.68	5.96	6.08	5.42
1810	0.4, 0.8	1.5	+	100	0.33	33	67	.90	3.72	5.24	4.08	3.52	3.54	5.24	3.82	3.32	3.36	5.20
1811	0.4, 0.8	1.5	-	100	0.33	67	33	.90	7.04	5.42	7.52	6.78	6.92	4.84	7.96	7.32	7.46	4.78
1812	0.4, 0.8	1.5	+	100	0.50	25	75	.90	2.90	5.12	3.16	2.72	2.82	5.42	2.46	2.04	2.06	4.76
1813	0.4, 0.8	1.5	-	100	0.50	75	25	.90	8.18	5.72	8.94	8.18	8.30	5.06	9.28	8.64	8.70	5.32
1814	0.4, 0.8	1.5	0	150	0	75	75	.90	5.94	5.92	5.92	5.30	5.38	5.58	6.00	5.34	5.40	5.60
1815	0.4, 0.8	1.5	+	150	0.16	63	87	.90	5.20	5.72	4.92	4.34	4.36	5.60	4.68	4.04	4.08	5.16
1816	0.4, 0.8	1.5	-	150	0.16	87	63	.90	6.12	5.10	6.76	6.22	6.32	5.26	6.82	6.16	6.22	5.20
1817	0.4, 0.8	1.5	+	150	0.33	50	100	.90	3.74	5.16	4.16	3.62	3.68	5.74	3.64	3.26	3.28	5.04
1818	0.4, 0.8	1.5	-	150	0.33	100	50	.90	6.34	4.88	7.92	7.36	7.38	5.22	7.46	6.78	6.86	4.78
1819	0.4, 0.8	1.5	+	150	0.50	37	113	.90	2.92	5.66	3.34	3.02	3.02	5.84	2.94	2.72	2.74	5.30
1820	0.4, 0.8	1.5	-	150	0.50	113	37	.90	7.36	5.32	9.06	8.30	8.34	5.22	8.60	7.90	7.92	5.16
1821	0.4, 0.8	1.5	0	200	0	100	100	.90	5.32	5.10	5.72	5.20	5.28	5.44	5.48	5.16	5.16	5.24
1822	0.4, 0.8	1.5	+	200	0.16	84	116	.90	4.26	5.40	4.94	4.44	4.48	5.48	4.86	4.40	4.42	5.12

	D	VR	P	N	Δn	n1	n2	ε	F g	B-F g	F t	F-GG t	F-HF t	B-F t	F g x t	F-GG g x t	F-HF g x t	B-F g x t
1823	0.4, 0.8	1.5	-	200	0.16	116	84	.90	5.88	5.26	6.94	6.32	6.36	5.60	6.64	5.86	5.98	5.26
1824	0.4, 0.8	1.5	+	200	0.33	67	133	.90	3.78	5.30	4.18	3.86	3.86	5.76	3.88	3.42	3.44	5.08
1825	0.4, 0.8	1.5	-	200	0.33	133	67	.90	6.94	5.52	8.22	7.28	7.36	5.30	7.94	7.08	7.16	5.16
1826	0.4, 0.8	1.5	+	200	0.50	50	150	.90	2.94	5.34	3.44	3.00	3.04	5.88	2.68	2.20	2.22	4.84
1827	0.4, 0.8	1.5	-	200	0.50	150	50	.90	7.84	5.40	9.56	8.62	8.66	5.34	9.46	8.72	8.74	5.06
1828	0.4, 0.8	2	0	20	0	10	10	.90	4.28	3.88	5.60	4.74	5.10	4.60	5.66	4.52	4.98	4.32
1829	0.4, 0.8	2	+	20	0.16	8	12	.90	3.52	4.20	3.52	2.90	3.06	4.16	3.78	2.94	3.18	4.46
1830	0.4, 0.8	2	-	20	0.16	12	8	.90	6.38	5.20	7.78	6.54	6.96	4.60	7.38	6.32	6.66	4.50
1831	0.4, 0.8	2	+	20	0.33	7	13	.90	3.36	4.98	2.86	2.36	2.58	4.36	3.32	2.66	2.92	4.98
1832	0.4, 0.8	2	-	20	0.33	13	7	.90	7.36	5.92	8.64	7.30	7.74	5.28	8.80	7.72	8.06	5.14
1833	0.4, 0.8	2	+	20	0.50	5	15	.90	2.24	6.30	1.90	1.50	1.72	5.44	2.04	1.66	1.80	5.66
1834	0.4, 0.8	2	-	20	0.50	15	5	.90	9.74	8.32	11.72	10.46	11.04	7.18	12.22	10.78	11.28	7.12
1835	0.4, 0.8	2	0	40	0	20	20	.90	5.02	4.64	6.00	5.12	5.48	5.08	5.48	4.66	4.94	4.58
1836	0.4, 0.8	2	+	40	0.16	17	23	.90	3.92	4.58	4.14	3.58	3.86	4.88	3.70	3.26	3.34	4.24
1837	0.4, 0.8	2	-	40	0.16	23	17	.90	5.96	4.60	7.62	6.86	7.12	5.38	6.90	6.02	6.20	4.96
1838	0.4, 0.8	2	+	40	0.33	13	27	.90	2.58	4.56	2.56	2.18	2.26	4.98	2.78	2.34	2.50	4.76
1839	0.4, 0.8	2	-	40	0.33	27	13	.90	7.94	5.44	10.44	9.42	9.62	5.54	9.16	8.08	8.32	4.92
1840	0.4, 0.8	2	+	40	0.50	10	30	.90	1.86	4.94	1.68	1.54	1.58	5.18	1.94	1.62	1.70	5.58
1841	0.4, 0.8	2	-	40	0.50	30	10	.90	9.34	6.48	11.80	10.70	10.90	6.28	11.82	10.48	10.76	6.10
1842	0.4, 0.8	2	0	60	0	30	30	.90	4.54	4.46	5.42	4.82	5.00	4.86	5.40	4.96	5.12	4.92
1843	0.4, 0.8	2	+	60	0.16	25	35	.90	3.70	4.70	3.82	3.36	3.52	4.60	4.14	3.60	3.78	4.82
1844	0.4, 0.8	2	-	60	0.16	35	25	.90	5.98	4.62	7.42	6.66	6.78	5.20	7.12	6.50	6.56	5.34
1845	0.4, 0.8	2	+	60	0.33	20	40	.90	2.98	5.10	2.78	2.36	2.42	4.56	2.92	2.52	2.64	4.84
1846	0.4, 0.8	2	-	60	0.33	40	20	.90	7.70	5.04	9.96	9.08	9.28	5.40	9.30	8.40	8.66	5.04
1847	0.4, 0.8	2	+	60	0.50	15	45	.90	2.10	5.40	1.92	1.66	1.76	4.76	1.90	1.64	1.70	5.14
1848	0.4, 0.8	2	-	60	0.50	45	15	.90	9.46	6.00	12.34	11.28	11.50	5.56	12.04	10.94	11.08	5.84
1849	0.4, 0.8	2	0	80	0	40	40	.90	4.62	4.48	6.10	5.50	5.62	5.46	5.40	4.96	5.06	4.90
1850	0.4, 0.8	2	+	80	0.16	34	46	.90	3.58	4.60	4.64	4.14	4.20	5.56	4.50	3.98	4.10	5.22
1851	0.4, 0.8	2	-	80	0.16	46	34	.90	6.02	4.94	7.96	7.22	7.38	5.78	7.24	6.32	6.46	5.04
1852	0.4, 0.8	2	+	80	0.33	27	53	.90	2.58	4.68	3.22	2.66	2.72	5.80	2.72	2.30	2.36	4.62
1853	0.4, 0.8	2	-	80	0.33	53	27	.90	7.36	4.88	10.20	9.46	9.62	5.70	9.32	8.56	8.74	5.20
1854	0.4, 0.8	2	+	80	0.50	20	60	.90	2.22	5.38	2.16	1.78	1.84	5.46	1.80	1.58	1.60	5.06

	D	VR	P	N	Δn	n1	n2	ε	F g	B-F g	F t	F-GG t	F-HF t	B-F t	F g x t	F-GG g x t	F-HF g x t	B-F g x t
1855	0.4, 0.8	2	-	80	0.50	60	20	.90	9.64	6.14	13.64	12.24	12.42	5.86	12.34	11.46	11.56	5.62
1856	0.4, 0.8	2	0	100	0	50	50	.90	5.24	5.18	5.32	4.72	4.78	4.98	5.04	4.44	4.62	4.68
1857	0.4, 0.8	2	+	100	0.16	42	58	.90	3.64	4.76	4.26	3.70	3.78	5.08	3.90	3.36	3.46	5.00
1858	0.4, 0.8	2	-	100	0.16	58	42	.90	6.84	5.44	7.12	6.52	6.72	4.98	7.30	6.56	6.66	5.08
1859	0.4, 0.8	2	+	100	0.33	33	67	.90	2.86	5.12	3.08	2.54	2.60	5.04	2.90	2.62	2.62	5.04
1860	0.4, 0.8	2	-	100	0.33	67	33	.90	8.32	5.30	9.46	8.50	8.66	4.88	9.56	9.02	9.06	4.78
1861	0.4, 0.8	2	+	100	0.50	25	75	.90	1.92	5.04	2.02	1.68	1.72	5.48	1.46	1.34	1.36	4.68
1862	0.4, 0.8	2	-	100	0.50	75	25	.90	10.44	5.82	11.86	10.92	11.04	4.90	12.52	11.44	11.66	5.48
1863	0.4, 0.8	2	0	150	0	75	75	.90	6.02	5.96	5.94	5.32	5.34	5.40	5.76	5.18	5.22	5.28
1864	0.4, 0.8	2	+	150	0.16	63	87	.90	4.54	5.80	4.50	3.92	3.98	5.54	4.04	3.60	3.62	5.04
1865	0.4, 0.8	2	-	150	0.16	87	63	.90	6.58	4.96	7.46	6.82	6.88	5.22	7.82	7.00	7.08	5.14
1866	0.4, 0.8	2	+	150	0.33	50	100	.90	2.80	4.94	3.08	2.50	2.52	5.68	2.86	2.48	2.52	5.10
1867	0.4, 0.8	2	-	150	0.33	100	50	.90	7.30	4.88	9.80	8.98	9.04	5.12	9.40	8.54	8.58	4.86
1868	0.4, 0.8	2	+	150	0.50	37	113	.90	1.98	5.58	2.16	1.82	1.88	5.90	2.08	1.68	1.70	5.30
1869	0.4, 0.8	2	-	150	0.50	113	37	.90	9.60	5.38	11.74	11.00	11.04	5.00	11.64	10.76	10.82	5.26
1870	0.4, 0.8	2	0	200	0	100	100	.90	5.30	5.20	5.76	5.38	5.40	5.38	5.62	5.24	5.26	5.22
1871	0.4, 0.8	2	+	200	0.16	84	116	.90	3.84	5.28	4.38	3.94	3.96	5.44	4.04	3.66	3.66	5.12
1872	0.4, 0.8	2	-	200	0.16	116	84	.90	6.40	5.28	7.54	6.86	6.90	5.60	7.22	6.68	6.70	5.42
1873	0.4, 0.8	2	+	200	0.33	67	133	.90	3.10	5.40	3.24	2.96	2.96	5.38	2.88	2.52	2.52	4.98
1874	0.4, 0.8	2	-	200	0.33	133	67	.90	8.26	5.48	10.00	8.98	9.02	5.40	9.86	9.14	9.24	5.54
1875	0.4, 0.8	2	+	200	0.50	50	150	.90	2.12	5.18	2.16	1.92	1.94	5.70	1.62	1.50	1.52	4.82
1876	0.4, 0.8	2	-	200	0.50	150	50	.90	10.36	5.16	12.58	11.78	11.82	5.38	12.80	11.86	11.90	5.16
1877	0.4, 0.8	5	0	20	0	10	10	.90	5.16	4.22	6.30	5.10	5.62	4.48	6.26	4.94	5.56	4.20
1878	0.4, 0.8	5	+	20	0.16	8	12	.90	3.22	4.46	2.76	2.10	2.38	4.10	2.56	2.04	2.22	4.16
1879	0.4, 0.8	5	-	20	0.16	12	8	.90	9.38	5.44	11.04	9.64	10.10	4.68	11.04	9.34	9.98	4.44
1880	0.4, 0.8	5	+	20	0.33	7	13	.90	2.34	4.68	1.60	1.34	1.44	4.22	1.70	1.38	1.44	4.64
1881	0.4, 0.8	5	-	20	0.33	13	7	.90	11.64	6.18	14.34	12.50	13.02	5.56	14.44	12.60	13.18	5.00
1882	0.4, 0.8	5	+	20	0.50	5	15	.90	.92	4.92	.52	.36	.46	4.52	.56	.40	.50	4.64
1883	0.4, 0.8	5	-	20	0.50	15	5	.90	18.02	8.66	23.32	20.58	21.52	7.08	23.52	21.18	21.90	7.26
1884	0.4, 0.8	5	0	40	0	20	20	.90	4.92	4.60	5.98	5.36	5.58	4.82	5.60	4.98	5.16	4.48
1885	0.4, 0.8	5	+	40	0.16	17	23	.90	3.26	4.62	2.90	2.56	2.68	4.44	2.66	2.28	2.34	4.26
1886	0.4, 0.8	5	-	40	0.16	23	17	.90	7.44	4.82	9.60	8.70	8.84	5.22	9.08	8.24	8.42	5.22

	D	VR	P	N	Δn	n1	n2	ε	F g	B-F g	F t	F-GG t	F-HF t	B-F t	F g x t	F-GG g x t	F-HF g x t	B-F g x t
1887	0.4, 0.8	5	+	40	0.33	13	27	.90	1.24	4.36	1.10	.84	.96	4.62	1.04	.86	.98	4.24
1888	0.4, 0.8	5	-	40	0.33	27	13	.90	12.04	5.38	16.00	14.56	14.84	5.54	15.12	13.50	13.94	5.02
1889	0.4, 0.8	5	+	40	0.50	10	30	.90	.64	4.32	.38	.34	.34	4.80	.40	.36	.36	5.12
1890	0.4, 0.8	5	-	40	0.50	30	10	.90	16.52	6.22	22.32	20.58	20.98	5.54	22.54	20.90	21.34	5.56
1891	0.4, 0.8	5	0	60	0	30	30	.90	4.48	4.16	5.90	5.18	5.26	4.84	5.72	5.02	5.14	4.88
1892	0.4, 0.8	5	+	60	0.16	25	35	.90	2.96	4.62	2.94	2.68	2.72	4.82	3.18	2.74	2.82	4.96
1893	0.4, 0.8	5	-	60	0.16	35	25	.90	7.82	4.22	10.14	8.84	9.14	4.98	10.36	9.16	9.42	4.90
1894	0.4, 0.8	5	+	60	0.33	20	40	.90	1.60	4.80	1.32	1.14	1.14	4.54	1.50	1.22	1.22	4.84
1895	0.4, 0.8	5	-	60	0.33	40	20	.90	11.90	5.00	15.76	14.36	14.66	5.20	15.42	14.06	14.36	5.04
1896	0.4, 0.8	5	+	60	0.50	15	45	.90	.74	4.86	.56	.46	.48	4.60	.50	.42	.42	4.90
1897	0.4, 0.8	5	-	60	0.50	45	15	.90	16.56	6.06	22.90	21.34	21.62	5.28	22.74	21.32	21.64	5.44
1898	0.4, 0.8	5	0	80	0	40	40	.90	4.88	4.64	5.86	5.20	5.28	5.18	5.52	4.96	5.14	4.88
1899	0.4, 0.8	5	+	80	0.16	34	46	.90	2.82	4.46	3.66	3.14	3.26	5.36	3.34	2.98	3.02	5.18
1900	0.4, 0.8	5	-	80	0.16	46	34	.90	7.52	5.02	9.78	8.78	9.00	5.62	9.16	8.30	8.44	5.06
1901	0.4, 0.8	5	+	80	0.33	27	53	.90	1.38	4.52	1.64	1.30	1.38	5.72	1.18	1.06	1.08	4.48
1902	0.4, 0.8	5	-	80	0.33	53	27	.90	11.36	4.88	16.00	14.52	14.68	5.58	15.18	13.88	14.10	5.30
1903	0.4, 0.8	5	+	80	0.50	20	60	.90	.66	5.14	.62	.54	.54	5.64	.48	.36	.40	4.86
1904	0.4, 0.8	5	-	80	0.50	60	20	.90	16.14	6.02	23.60	22.10	22.32	5.78	22.94	21.70	21.94	5.34
1905	0.4, 0.8	5	0	100	0	50	50	.90	5.06	4.94	5.54	5.06	5.14	4.92	5.46	4.88	5.00	4.88
1906	0.4, 0.8	5	+	100	0.16	42	58	.90	2.78	4.42	3.04	2.70	2.76	4.78	2.84	2.52	2.58	4.94
1907	0.4, 0.8	5	-	100	0.16	58	42	.90	8.22	5.38	9.46	8.54	8.62	5.10	9.54	8.72	8.82	5.08
1908	0.4, 0.8	5	+	100	0.33	33	67	.90	1.04	4.88	1.30	1.10	1.14	4.92	1.22	1.08	1.12	5.16
1909	0.4, 0.8	5	-	100	0.33	67	33	.90	12.32	5.14	15.38	14.20	14.34	4.66	15.72	14.56	14.62	4.54
1910	0.4, 0.8	5	+	100	0.50	25	75	.90	.58	4.52	.38	.36	.36	5.14	.46	.32	.36	4.66
1911	0.4, 0.8	5	-	100	0.50	75	25	.90	17.64	5.72	21.74	20.38	20.60	4.90	22.32	20.92	21.06	5.12
1912	0.4, 0.8	5	0	150	0	75	75	.90	5.70	5.62	5.92	5.42	5.48	5.28	5.80	5.24	5.40	5.22
1913	0.4, 0.8	5	+	150	0.16	63	87	.90	3.46	5.42	3.00	2.52	2.54	5.54	2.80	2.44	2.46	5.16
1914	0.4, 0.8	5	-	150	0.16	87	63	.90	8.32	5.20	9.72	8.84	8.88	5.10	9.66	8.88	8.94	5.04
1915	0.4, 0.8	5	+	150	0.33	50	100	.90	1.42	5.06	1.40	1.14	1.16	5.64	1.22	1.04	1.06	5.20
1916	0.4, 0.8	5	-	150	0.33	100	50	.90	11.18	4.94	15.06	14.14	14.18	4.90	14.70	13.80	13.88	4.84
1917	0.4, 0.8	5	+	150	0.50	37	113	.90	.50	5.10	.52	.38	.38	5.86	.52	.46	.46	5.16
1918	0.4, 0.8	5	-	150	0.50	113	37	.90	17.06	5.54	22.80	21.22	21.36	4.82	21.24	19.88	20.00	5.18

	D	VR	P	N	Δn	n1	n2	ε	F g	B-F g	F t	F-GG t	F-HF t	B-F t	F g x t	F-GG g x t	F-HF g x t	B-F g x t
1919	0.4, 0.8	5	0	200	0	100	100	.90	5.10	5.02	5.84	5.38	5.44	5.26	5.56	5.22	5.22	5.10
1920	0.4, 0.8	5	+	200	0.16	84	116	.90	2.88	5.12	3.24	2.82	2.90	5.64	3.14	2.82	2.82	5.16
1921	0.4, 0.8	5	-	200	0.16	116	84	.90	7.90	5.12	9.64	8.74	8.80	5.74	9.16	8.56	8.58	5.56
1922	0.4, 0.8	5	+	200	0.33	67	133	.90	1.90	5.14	1.46	1.16	1.18	5.22	1.26	1.08	1.08	4.98
1923	0.4, 0.8	5	-	200	0.33	133	67	.90	12.04	5.26	15.40	14.46	14.48	5.70	15.62	14.70	14.82	5.64
1924	0.4, 0.8	5	+	200	0.50	50	150	.90	.54	4.90	.42	.36	.36	5.50	.14	.14	.14	4.64
1925	0.4, 0.8	5	-	200	0.50	150	50	.90	17.30	5.18	23.14	21.76	21.84	5.34	23.18	21.92	22.04	5.44
1926	0.4, 0.8	1	0	20	0	10	10	1	4.04	3.90	4.68	4.06	4.48	4.12	5.20	4.50	4.98	4.68
1927	0.4, 0.8	1	0	20	0.16	8	12	1	4.80	4.38	4.50	4.06	4.40	4.14	5.28	4.60	5.08	4.36
1928	0.4, 0.8	1	0	20	0.33	7	13	1	4.84	5.00	4.68	4.08	4.46	4.30	4.90	4.34	4.66	4.62
1929	0.4, 0.8	1	0	20	0.50	5	15	1	5.10	7.00	4.94	4.46	4.70	6.62	5.10	4.48	4.88	6.62
1930	0.4, 0.8	1	0	40	0	20	20	1	4.90	4.96	4.74	4.38	4.54	4.40	4.72	4.48	4.60	4.30
1931	0.4, 0.8	1	0	40	0.16	17	23	1	5.08	5.02	5.00	4.64	4.82	4.94	4.50	4.16	4.44	4.48
1932	0.4, 0.8	1	0	40	0.33	13	27	1	4.28	4.98	4.80	4.52	4.68	4.88	5.04	4.74	4.94	5.10
1933	0.4, 0.8	1	0	40	0.50	10	30	1	4.30	5.76	4.70	4.44	4.62	5.76	5.04	4.82	4.94	6.22
1934	0.4, 0.8	1	0	60	0	30	30	1	5.12	5.06	5.18	4.92	5.10	4.98	5.22	5.02	5.14	4.78
1935	0.4, 0.8	1	0	60	0.16	25	35	1	5.06	4.92	4.94	4.88	4.90	4.86	4.92	4.64	4.78	4.70
1936	0.4, 0.8	1	0	60	0.33	20	40	1	5.24	5.52	4.98	4.84	4.92	5.04	4.98	4.80	4.94	5.32
1937	0.4, 0.8	1	0	60	0.50	15	45	1	4.90	6.04	4.70	4.60	4.62	5.28	5.06	4.90	4.98	5.80
1938	0.4, 0.8	1	0	80	0	40	40	1	4.68	4.90	5.80	5.60	5.76	5.52	4.30	4.20	4.28	4.12
1939	0.4, 0.8	1	0	80	0.16	34	46	1	4.74	4.76	5.62	5.46	5.58	5.42	5.24	5.08	5.12	5.28
1940	0.4, 0.8	1	0	80	0.33	27	53	1	4.96	5.26	5.38	5.10	5.24	5.34	4.66	4.54	4.60	4.78
1941	0.4, 0.8	1	0	80	0.50	20	60	1	5.48	5.88	5.40	5.24	5.28	5.34	4.92	4.80	4.88	5.24
1942	0.4, 0.8	1	0	100	0	50	50	1	5.34	5.46	5.34	5.20	5.28	5.22	4.98	4.90	4.96	4.94
1943	0.4, 0.8	1	0	100	0.16	42	58	1	4.78	5.00	5.46	5.28	5.38	5.48	4.88	4.80	4.86	4.80
1944	0.4, 0.8	1	0	100	0.33	33	67	1	5.08	5.18	5.12	4.98	5.12	5.32	5.16	5.06	5.12	5.36
1945	0.4, 0.8	1	0	100	0.50	25	75	1	5.18	5.50	5.04	4.92	4.94	5.10	4.32	4.22	4.30	4.82
1946	0.4, 0.8	1	0	150	0	75	75	1	5.64	5.62	5.08	4.98	5.02	4.98	5.68	5.64	5.68	5.80
1947	0.4, 0.8	1	0	150	0.16	63	87	1	5.60	5.80	4.96	4.88	4.94	5.18	5.16	5.16	5.16	5.16
1948	0.4, 0.8	1	0	150	0.33	50	100	1	5.50	5.50	5.20	5.08	5.18	5.38	5.20	5.14	5.20	5.40
1949	0.4, 0.8	1	0	150	0.50	37	113	1	5.10	5.80	5.36	5.22	5.32	5.54	4.88	4.86	4.88	5.18
1950	0.4, 0.8	1	0	200	0	100	100	1	5.04	5.32	5.02	4.98	5.02	5.08	5.60	5.58	5.60	5.54

	D	VR	P	N	Δn	n1	n2	ε	F g	B-F g	F t	F-GG t	F-HF t	B-F t	F g x t	F-GG g x t	F-HF g x t	B-F g x t
1951	0.4, 0.8	1	0	200	0.16	84	116	1	5.30	5.34	5.22	5.16	5.22	5.30	5.48	5.44	5.44	5.54
1952	0.4, 0.8	1	0	200	0.33	67	133	1	5.60	5.62	5.42	5.32	5.42	5.52	5.72	5.68	5.72	5.60
1953	0.4, 0.8	1	0	200	0.50	50	150	1	4.96	5.38	5.18	5.10	5.10	5.12	5.02	5.02	5.02	5.02
1954	0.4, 0.8	1.5	0	20	0	10	10	1	4.28	3.94	4.78	4.26	4.58	3.96	5.22	4.42	4.88	3.98
1955	0.4, 0.8	1.5	+	20	0.16	8	12	1	4.12	4.46	3.62	3.04	3.38	3.94	4.02	3.64	3.80	4.56
1956	0.4, 0.8	1.5	-	20	0.16	12	8	1	5.74	4.96	6.10	5.30	5.80	4.66	6.46	5.82	6.18	4.34
1957	0.4, 0.8	1.5	+	20	0.33	7	13	1	3.86	5.12	3.18	2.80	3.06	4.46	3.44	3.04	3.26	4.68
1958	0.4, 0.8	1.5	-	20	0.33	13	7	1	6.36	5.50	6.80	6.06	6.46	5.10	7.02	6.06	6.64	4.88
1959	0.4, 0.8	1.5	+	20	0.50	5	15	1	3.16	6.14	2.60	2.34	2.52	5.88	2.64	2.44	2.54	5.84
1960	0.4, 0.8	1.5	-	20	0.50	15	5	1	7.76	8.06	8.52	7.78	8.24	7.60	9.26	8.28	8.86	7.06
1961	0.4, 0.8	1.5	0	40	0	20	20	1	4.92	4.96	4.76	4.52	4.62	4.48	4.58	4.34	4.48	4.38
1962	0.4, 0.8	1.5	+	40	0.16	17	23	1	4.40	4.90	4.08	3.80	4.02	4.62	3.70	3.52	3.62	4.36
1963	0.4, 0.8	1.5	-	40	0.16	23	17	1	5.68	5.18	5.82	5.52	5.72	4.82	5.66	5.42	5.58	4.90
1964	0.4, 0.8	1.5	+	40	0.33	13	27	1	3.06	4.80	3.12	2.88	3.04	4.82	3.30	3.14	3.24	4.86
1965	0.4, 0.8	1.5	-	40	0.33	27	13	1	6.68	5.38	7.66	7.22	7.42	5.00	6.48	6.26	6.40	4.70
1966	0.4, 0.8	1.5	+	40	0.50	10	30	1	2.48	4.92	2.42	2.32	2.34	5.58	2.58	2.40	2.48	6.22
1967	0.4, 0.8	1.5	-	40	0.50	30	10	1	7.20	6.24	8.34	7.98	8.24	5.96	7.98	7.50	7.80	5.24
1968	0.4, 0.8	1.5	0	60	0	30	30	1	5.16	4.94	5.32	5.12	5.30	5.12	5.14	4.96	5.12	4.74
1969	0.4, 0.8	1.5	+	60	0.16	25	35	1	4.08	4.80	4.22	4.10	4.18	4.92	4.02	3.68	3.94	4.74
1970	0.4, 0.8	1.5	-	60	0.16	35	25	1	5.50	4.38	6.52	6.26	6.40	5.16	6.36	6.16	6.24	5.06
1971	0.4, 0.8	1.5	+	60	0.33	20	40	1	3.66	5.04	3.42	3.28	3.38	4.94	3.42	3.26	3.36	5.10
1972	0.4, 0.8	1.5	-	60	0.33	40	20	1	6.14	4.82	7.56	7.16	7.36	4.96	6.72	6.50	6.68	4.46
1973	0.4, 0.8	1.5	+	60	0.50	15	45	1	3.20	5.72	2.68	2.64	2.66	5.08	2.66	2.54	2.56	5.66
1974	0.4, 0.8	1.5	-	60	0.50	45	15	1	7.30	5.42	9.22	9.00	9.14	5.38	8.42	8.14	8.30	5.26
1975	0.4, 0.8	1.5	0	80	0	40	40	1	4.80	4.90	5.66	5.50	5.60	5.56	4.64	4.56	4.58	4.54
1976	0.4, 0.8	1.5	+	80	0.16	34	46	1	4.00	4.62	4.82	4.70	4.80	5.74	4.24	4.12	4.24	5.22
1977	0.4, 0.8	1.5	-	80	0.16	46	34	1	5.48	4.84	6.64	6.36	6.52	5.52	5.82	5.60	5.78	4.46
1978	0.4, 0.8	1.5	+	80	0.33	27	53	1	3.52	5.00	3.74	3.56	3.64	5.54	3.04	2.98	3.02	4.78
1979	0.4, 0.8	1.5	-	80	0.33	53	27	1	6.10	4.74	7.92	7.72	7.84	5.62	7.32	7.10	7.22	4.90
1980	0.4, 0.8	1.5	+	80	0.50	20	60	1	3.30	5.60	2.78	2.66	2.72	5.56	2.54	2.42	2.48	5.08
1981	0.4, 0.8	1.5	-	80	0.50	60	20	1	7.58	5.66	9.52	9.26	9.44	5.90	9.04	8.70	8.92	5.72
1982	0.4, 0.8	1.5	0	100	0	50	50	1	5.34	5.44	5.28	5.20	5.28	5.16	4.88	4.80	4.86	4.76

	D	VR	P	N	Δn	n1	n2	ε	F g	B-F g	F t	F-GG t	F-HF t	B-F t	F g x t	F-GG g x t	F-HF g x t	B-F g x t
1983	0.4, 0.8	1.5	+	100	0.16	42	58	1	4.12	4.88	4.26	4.16	4.24	5.36	4.14	4.08	4.12	4.86
1984	0.4, 0.8	1.5	-	100	0.16	58	42	1	6.10	5.48	6.22	6.08	6.14	5.02	6.10	6.02	6.08	5.02
1985	0.4, 0.8	1.5	+	100	0.33	33	67	1	3.68	5.08	3.50	3.38	3.42	5.32	3.36	3.28	3.32	5.54
1986	0.4, 0.8	1.5	-	100	0.33	67	33	1	7.06	5.46	7.46	7.34	7.44	4.82	7.00	6.84	6.90	4.60
1987	0.4, 0.8	1.5	+	100	0.50	25	75	1	3.12	5.22	2.50	2.44	2.48	5.28	2.40	2.28	2.34	4.82
1988	0.4, 0.8	1.5	-	100	0.50	75	25	1	7.98	5.62	8.28	8.14	8.22	5.02	8.66	8.46	8.52	5.24
1989	0.4, 0.8	1.5	0	150	0	75	75	1	5.82	5.74	5.06	4.96	5.04	5.06	5.64	5.56	5.62	5.74
1990	0.4, 0.8	1.5	+	150	0.16	63	87	1	5.04	5.72	4.10	4.06	4.06	5.16	4.38	4.32	4.32	5.20
1991	0.4, 0.8	1.5	-	150	0.16	87	63	1	5.66	4.96	6.34	6.20	6.24	4.82	6.58	6.48	6.52	5.38
1992	0.4, 0.8	1.5	+	150	0.33	50	100	1	3.86	5.30	3.32	3.24	3.26	5.24	3.46	3.38	3.44	5.42
1993	0.4, 0.8	1.5	-	150	0.33	100	50	1	6.42	4.82	7.44	7.30	7.36	5.20	7.42	7.34	7.40	5.16
1994	0.4, 0.8	1.5	+	150	0.50	37	113	1	3.00	5.62	2.74	2.68	2.70	5.58	2.50	2.48	2.50	4.82
1995	0.4, 0.8	1.5	-	150	0.50	113	37	1	7.48	5.22	8.68	8.58	8.66	4.94	9.00	8.88	8.92	5.24
1996	0.4, 0.8	1.5	0	200	0	100	100	1	5.12	5.18	4.94	4.94	4.94	5.00	5.70	5.66	5.68	5.70
1997	0.4, 0.8	1.5	+	200	0.16	84	116	1	4.42	5.30	4.26	4.22	4.24	5.08	4.50	4.40	4.48	5.44
1998	0.4, 0.8	1.5	-	200	0.16	116	84	1	6.38	5.54	6.20	6.12	6.18	5.10	6.46	6.36	6.44	5.50
1999	0.4, 0.8	1.5	+	200	0.33	67	133	1	4.00	5.50	3.48	3.46	3.48	5.42	3.76	3.70	3.76	5.54
2000	0.4, 0.8	1.5	-	200	0.33	133	67	1	6.62	5.18	7.42	7.38	7.40	4.84	7.72	7.54	7.60	5.54
2001	0.4, 0.8	1.5	+	200	0.50	50	150	1	3.22	5.46	2.68	2.64	2.68	5.18	2.38	2.38	2.38	5.14
2002	0.4, 0.8	1.5	-	200	0.50	150	50	1	7.60	5.46	8.92	8.78	8.88	5.28	8.94	8.82	8.92	5.06
2003	0.4, 0.8	2	0	20	0	10	10	1	4.48	3.96	5.02	4.38	4.82	4.00	5.18	4.62	4.96	4.06
2004	0.4, 0.8	2	+	20	0.16	8	12	1	3.72	4.36	3.20	2.66	2.94	3.96	3.34	2.98	3.24	4.46
2005	0.4, 0.8	2	-	20	0.16	12	8	1	6.64	4.92	6.86	6.36	6.66	4.54	7.58	6.80	7.24	4.40
2006	0.4, 0.8	2	+	20	0.33	7	13	1	3.38	4.78	2.44	2.20	2.34	4.30	2.58	2.26	2.48	4.82
2007	0.4, 0.8	2	-	20	0.33	13	7	1	7.54	5.76	8.60	7.78	8.24	5.30	8.76	7.90	8.26	5.02
2008	0.4, 0.8	2	+	20	0.50	5	15	1	2.14	5.78	1.66	1.44	1.58	5.18	1.86	1.48	1.64	5.36
2009	0.4, 0.8	2	-	20	0.50	15	5	1	9.74	8.74	11.72	10.88	11.34	7.78	12.54	11.48	12.20	7.10
2010	0.4, 0.8	2	0	40	0	20	20	1	4.92	4.96	4.74	4.44	4.66	4.48	4.76	4.52	4.60	4.42
2011	0.4, 0.8	2	+	40	0.16	17	23	1	4.16	4.90	3.60	3.46	3.56	4.36	3.38	3.18	3.32	4.12
2012	0.4, 0.8	2	-	40	0.16	23	17	1	6.28	5.16	6.58	6.32	6.50	4.82	6.58	6.22	6.40	5.14
2013	0.4, 0.8	2	+	40	0.33	13	27	1	2.44	4.64	2.18	2.06	2.12	4.80	2.50	2.28	2.46	4.76
2014	0.4, 0.8	2	-	40	0.33	27	13	1	8.02	5.52	9.66	9.40	9.52	5.10	8.42	7.88	8.18	4.64

	D	VR	P	N	Δn	n1	n2	ε	F g	B-F g	F t	F-GG t	F-HF t	B-F t	F g x t	F-GG g x t	F-HF g x t	B-F g x t
2015	0.4, 0.8	2	+	40	0.50	10	30	1	1.86	4.64	1.52	1.42	1.52	5.42	1.64	1.46	1.58	5.92
2016	0.4, 0.8	2	-	40	0.50	30	10	1	9.42	6.40	11.46	10.94	11.28	5.96	10.98	10.40	10.68	5.38
2017	0.4, 0.8	2	0	60	0	30	30	1	5.00	4.92	5.42	5.24	5.38	5.02	5.28	4.98	5.18	4.82
2018	0.4, 0.8	2	+	60	0.16	25	35	1	3.72	4.78	3.88	3.70	3.80	4.98	3.32	3.02	3.20	4.82
2019	0.4, 0.8	2	-	60	0.16	35	25	1	5.98	4.08	7.42	7.22	7.36	5.04	7.32	7.04	7.22	4.94
2020	0.4, 0.8	2	+	60	0.33	20	40	1	3.04	5.04	2.72	2.66	2.70	5.04	2.56	2.36	2.44	5.20
2021	0.4, 0.8	2	-	60	0.33	40	20	1	7.30	4.80	9.40	9.14	9.30	4.94	8.82	8.48	8.72	4.56
2022	0.4, 0.8	2	+	60	0.50	15	45	1	2.20	5.28	1.72	1.62	1.62	5.10	1.74	1.68	1.68	5.54
2023	0.4, 0.8	2	-	60	0.50	45	15	1	9.38	5.56	12.02	11.66	11.84	5.46	11.34	11.06	11.28	5.20
2024	0.4, 0.8	2	0	80	0	40	40	1	4.86	4.86	5.64	5.26	5.44	5.20	4.82	4.60	4.68	4.50
2025	0.4, 0.8	2	+	80	0.16	34	46	1	3.68	4.72	4.02	3.90	3.96	5.56	3.88	3.82	3.86	5.18
2026	0.4, 0.8	2	-	80	0.16	46	34	1	5.82	4.78	7.42	7.04	7.28	5.32	6.48	6.28	6.38	4.60
2027	0.4, 0.8	2	+	80	0.33	27	53	1	2.84	4.70	2.80	2.60	2.64	5.62	2.16	2.08	2.14	4.70
2028	0.4, 0.8	2	-	80	0.33	53	27	1	7.26	4.88	9.98	9.80	9.86	5.58	9.24	9.02	9.10	4.94
2029	0.4, 0.8	2	+	80	0.50	20	60	1	2.08	5.50	1.76	1.70	1.74	5.82	1.32	1.26	1.28	5.30
2030	0.4, 0.8	2	-	80	0.50	60	20	1	9.46	5.40	12.76	12.54	12.70	5.76	12.04	11.80	11.94	5.60
2031	0.4, 0.8	2	0	100	0	50	50	1	5.28	5.28	5.28	5.14	5.20	4.92	5.08	4.90	5.00	5.12
2032	0.4, 0.8	2	+	100	0.16	42	58	1	3.64	4.88	3.78	3.66	3.70	5.48	3.58	3.46	3.52	4.98
2033	0.4, 0.8	2	-	100	0.16	58	42	1	6.74	5.50	6.70	6.60	6.62	4.86	7.08	6.90	7.02	5.10
2034	0.4, 0.8	2	+	100	0.33	33	67	1	2.84	5.12	2.28	2.24	2.26	5.36	2.58	2.46	2.52	5.30
2035	0.4, 0.8	2	-	100	0.33	67	33	1	8.44	5.64	9.44	9.28	9.38	5.04	9.24	8.98	9.12	4.68
2036	0.4, 0.8	2	+	100	0.50	25	75	1	1.94	5.08	1.50	1.44	1.46	5.16	1.40	1.38	1.40	4.62
2037	0.4, 0.8	2	-	100	0.50	75	25	1	10.12	5.80	11.52	11.34	11.48	5.38	11.40	11.26	11.34	5.18
2038	0.4, 0.8	2	0	150	0	75	75	1	5.76	5.70	5.24	5.12	5.16	4.96	5.54	5.44	5.54	5.46
2039	0.4, 0.8	2	+	150	0.16	63	87	1	4.50	5.58	3.36	3.28	3.30	5.08	3.86	3.78	3.84	5.12
2040	0.4, 0.8	2	-	150	0.16	87	63	1	6.22	5.16	7.04	6.98	7.04	5.02	7.50	7.38	7.44	5.02
2041	0.4, 0.8	2	+	150	0.33	50	100	1	3.06	5.22	2.42	2.34	2.34	5.18	2.56	2.50	2.54	5.30
2042	0.4, 0.8	2	-	150	0.33	100	50	1	7.56	4.88	9.34	9.14	9.26	5.08	9.40	9.34	9.38	5.00
2043	0.4, 0.8	2	+	150	0.50	37	113	1	2.08	5.30	1.64	1.60	1.62	5.62	1.48	1.44	1.46	5.02
2044	0.4, 0.8	2	-	150	0.50	113	37	1	9.72	5.30	12.36	12.20	12.30	4.96	11.86	11.68	11.76	5.32
2045	0.4, 0.8	2	0	200	0	100	100	1	5.22	5.12	4.98	4.92	4.92	4.94	5.72	5.72	5.72	5.80
2046	0.4, 0.8	2	+	200	0.16	84	116	1	3.76	5.26	3.78	3.76	3.76	5.06	3.88	3.82	3.88	5.38

	D	VR	P	N	Δn	n1	n2	ε	F g	B-F g	F t	F-GG t	F-HF t	B-F t	F g x t	F-GG g x t	F-HF g x t	B-F g x t
2047	0.4, 0.8	2	-	200	0.16	116	84	1	6.98	5.36	7.10	6.96	7.04	5.14	7.26	7.18	7.22	5.38
2048	0.4, 0.8	2	+	200	0.33	67	133	1	3.02	5.44	2.52	2.52	2.52	5.34	2.36	2.34	2.36	5.56
2049	0.4, 0.8	2	-	200	0.33	133	67	1	8.00	5.20	9.30	9.22	9.26	4.78	9.56	9.44	9.50	5.44
2050	0.4, 0.8	2	+	200	0.50	50	150	1	2.22	5.30	1.80	1.78	1.78	5.36	1.26	1.22	1.26	5.46
2051	0.4, 0.8	2	-	200	0.50	150	50	1	10.12	5.44	12.18	12.06	12.16	5.36	12.22	12.10	12.22	5.12
2052	0.4, 0.8	5	0	20	0	10	10	1	5.20	4.24	5.52	4.76	5.16	4.04	5.58	4.84	5.24	3.96
2053	0.4, 0.8	5	+	20	0.16	8	12	1	3.02	4.36	2.46	2.06	2.36	4.02	2.38	1.96	2.16	3.86
2054	0.4, 0.8	5	-	20	0.16	12	8	1	9.36	5.52	10.98	9.90	10.36	4.30	10.94	9.68	10.08	3.98
2055	0.4, 0.8	5	+	20	0.33	7	13	1	2.30	4.18	1.44	1.18	1.32	3.20	1.24	1.04	1.12	3.58
2056	0.4, 0.8	5	-	20	0.33	13	7	1	11.90	6.18	14.28	12.72	13.50	4.52	14.92	13.10	14.08	4.54
2057	0.4, 0.8	5	+	20	0.50	5	15	1	.96	5.20	.32	.24	.26	4.78	.32	.24	.30	4.82
2058	0.4, 0.8	5	-	20	0.50	15	5	1	17.84	8.60	23.02	21.00	21.98	6.66	24.32	21.96	23.00	6.60
2059	0.4, 0.8	5	0	40	0	20	20	1	5.20	4.62	5.14	4.92	5.00	4.58	4.78	4.44	4.64	4.18
2060	0.4, 0.8	5	+	40	0.16	17	23	1	3.38	4.96	2.86	2.62	2.74	4.60	2.68	2.46	2.58	4.78
2061	0.4, 0.8	5	-	40	0.16	23	17	1	7.76	5.20	9.16	8.66	8.84	4.78	8.60	8.00	8.24	4.54
2062	0.4, 0.8	5	+	40	0.33	13	27	1	1.16	4.64	1.08	.98	1.04	4.70	.86	.80	.82	4.96
2063	0.4, 0.8	5	-	40	0.33	27	13	1	12.30	5.58	15.70	14.90	15.28	5.52	15.10	14.30	14.70	4.92
2064	0.4, 0.8	5	+	40	0.50	10	30	1	.64	4.22	.36	.34	.34	4.82	.30	.18	.28	5.10
2065	0.4, 0.8	5	-	40	0.50	30	10	1	16.90	6.24	22.14	21.12	21.44	5.64	22.42	21.38	21.70	5.20
2066	0.4, 0.8	5	0	60	0	30	30	1	4.82	4.58	5.52	5.24	5.32	4.96	5.50	5.22	5.36	4.90
2067	0.4, 0.8	5	+	60	0.16	25	35	1	2.86	4.60	2.74	2.58	2.70	4.98	2.40	2.24	2.34	4.66
2068	0.4, 0.8	5	-	60	0.16	35	25	1	7.64	3.98	9.98	9.70	9.86	5.00	9.82	9.38	9.60	5.10
2069	0.4, 0.8	5	+	60	0.33	20	40	1	1.52	4.74	1.24	1.20	1.22	5.16	1.04	1.02	1.02	5.02
2070	0.4, 0.8	5	-	60	0.33	40	20	1	11.72	4.66	16.18	15.52	15.74	4.52	14.94	14.32	14.54	4.40
2071	0.4, 0.8	5	+	60	0.50	15	45	1	.86	5.02	.40	.38	.40	5.00	.30	.28	.30	5.08
2072	0.4, 0.8	5	-	60	0.50	45	15	1	16.40	5.58	23.48	22.80	23.18	4.90	22.72	22.12	22.26	4.92
2073	0.4, 0.8	5	0	80	0	40	40	1	4.84	4.70	5.48	5.20	5.32	5.22	5.26	5.02	5.08	4.82
2074	0.4, 0.8	5	+	80	0.16	34	46	1	2.78	4.58	2.96	2.88	2.90	5.62	2.82	2.68	2.80	5.72
2075	0.4, 0.8	5	-	80	0.16	46	34	1	7.52	4.66	9.32	8.96	9.16	5.20	8.88	8.42	8.60	4.74
2076	0.4, 0.8	5	+	80	0.33	27	53	1	1.60	4.42	1.32	1.20	1.28	5.26	.92	.86	.88	4.88
2077	0.4, 0.8	5	-	80	0.33	53	27	1	11.26	4.70	15.78	15.40	15.64	5.38	15.50	14.80	15.04	4.98
2078	0.4, 0.8	5	+	80	0.50	20	60	1	.70	5.18	.40	.38	.38	5.90	.24	.22	.22	5.22

	D	VR	P	N	Δn	n1	n2	ε	F g	B-F g	F t	F-GG t	F-HF t	B-F t	F g x t	F-GG g x t	F-HF g x t	B-F g x t
2079	0.4, 0.8	5	-	80	0.50	60	20	1	16.18	5.40	23.70	23.18	23.44	5.58	23.08	22.50	22.80	5.28
2080	0.4, 0.8	5	0	100	0	50	50	1	5.20	5.02	5.18	5.04	5.08	4.94	5.06	4.92	5.02	4.88
2081	0.4, 0.8	5	+	100	0.16	42	58	1	2.68	4.50	2.48	2.36	2.38	5.06	2.60	2.48	2.54	5.02
2082	0.4, 0.8	5	-	100	0.16	58	42	1	8.10	5.34	9.12	8.80	8.94	5.00	9.12	8.86	8.94	5.32
2083	0.4, 0.8	5	+	100	0.33	33	67	1	1.12	4.84	.82	.82	.82	5.36	.98	.98	.98	5.10
2084	0.4, 0.8	5	-	100	0.33	67	33	1	12.52	5.38	15.70	15.28	15.50	4.72	15.58	15.18	15.30	4.52
2085	0.4, 0.8	5	+	100	0.50	25	75	1	.56	4.88	.40	.38	.38	5.22	.16	.16	.16	4.74
2086	0.4, 0.8	5	-	100	0.50	75	25	1	18.12	6.04	22.36	22.04	22.24	5.06	22.24	21.72	21.90	5.24
2087	0.4, 0.8	5	0	150	0	75	75	1	5.58	5.70	5.00	4.90	4.96	5.14	5.16	5.04	5.14	5.34
2088	0.4, 0.8	5	+	150	0.16	63	87	1	3.50	5.46	2.52	2.36	2.46	4.96	2.90	2.80	2.82	5.10
2089	0.4, 0.8	5	-	150	0.16	87	63	1	7.94	5.02	9.34	9.16	9.20	4.94	9.60	9.42	9.56	4.74
2090	0.4, 0.8	5	+	150	0.33	50	100	1	1.50	4.96	1.02	1.00	1.02	4.94	.82	.78	.80	4.94
2091	0.4, 0.8	5	-	150	0.33	100	50	1	11.28	4.90	15.46	15.18	15.28	5.00	15.46	15.24	15.36	5.04
2092	0.4, 0.8	5	+	150	0.50	37	113	1	.40	4.96	.36	.36	.36	5.24	.32	.28	.32	4.74
2093	0.4, 0.8	5	-	150	0.50	113	37	1	16.84	5.28	23.02	22.76	22.82	4.84	22.48	22.08	22.24	5.48
2094	0.4, 0.8	5	0	200	0	100	100	1	4.86	4.78	5.40	5.32	5.40	5.12	5.46	5.40	5.44	5.22
2095	0.4, 0.8	5	+	200	0.16	84	116	1	2.86	5.04	2.64	2.56	2.58	5.26	2.68	2.62	2.64	5.34
2096	0.4, 0.8	5	-	200	0.16	116	84	1	8.06	5.28	9.24	9.20	9.22	5.24	9.72	9.54	9.60	5.28
2097	0.4, 0.8	5	+	200	0.33	67	133	1	1.68	4.78	1.08	1.06	1.06	5.18	.96	.94	.94	5.44
2098	0.4, 0.8	5	-	200	0.33	133	67	1	11.84	5.06	15.48	15.34	15.36	4.86	15.70	15.58	15.60	5.56
2099	0.4, 0.8	5	+	200	0.50	50	150	1	.64	4.82	.30	.28	.28	5.20	.16	.16	.16	5.04
2100	0.4, 0.8	5	-	200	0.50	150	50	1	17.24	5.32	22.84	22.66	22.76	5.60	23.26	22.96	23.10	4.88
2101	1, 1.5	1	0	20	0	10	10	.50	4.28	3.84	9.38	5.68	5.68	4.90	7.98	4.44	4.44	4.14
2102	1, 1.5	1	0	20	0.16	8	12	.50	5.12	4.50	9.02	5.72	5.72	4.84	8.98	5.16	5.16	4.54
2103	1, 1.5	1	0	20	0.33	7	13	.50	4.80	4.86	9.26	5.62	5.66	5.20	8.52	4.84	4.84	4.84
2104	1, 1.5	1	0	20	0.50	5	15	.50	4.86	7.26	9.54	5.40	5.40	7.60	8.54	4.90	4.90	7.48
2105	1, 1.5	1	0	40	0	20	20	.50	4.70	4.42	9.02	5.54	5.54	4.92	8.16	4.94	4.94	4.64
2106	1, 1.5	1	0	40	0.16	17	23	.50	4.66	4.70	8.96	5.42	5.42	5.40	8.26	4.52	4.54	4.44
2107	1, 1.5	1	0	40	0.33	13	27	.50	4.52	4.72	8.90	5.52	5.52	5.66	8.18	4.58	4.58	4.76
2108	1, 1.5	1	0	40	0.50	10	30	.50	4.54	5.92	8.82	5.20	5.20	6.40	8.74	4.72	4.72	6.00
2109	1, 1.5	1	0	60	0	30	30	.50	4.80	4.72	8.68	5.40	5.40	5.20	8.26	4.80	4.80	4.84
2110	1, 1.5	1	0	60	0.16	25	35	.50	4.64	4.66	8.62	5.34	5.34	5.20	7.96	4.86	4.86	4.72

	D	VR	P	N	Δn	n1	n2	ε	F g	B-F g	F t	F-GG t	F-HF t	B-F t	F g x t	F-GG g x t	F-HF g x t	B-F g x t
2111	1, 1.5	1	0	60	0.33	20	40	.50	4.80	5.30	9.02	5.48	5.48	5.76	8.36	5.08	5.08	5.30
2112	1, 1.5	1	0	60	0.50	15	45	.50	4.86	5.74	9.14	5.14	5.14	6.34	8.30	4.82	4.84	5.78
2113	1, 1.5	1	0	80	0	40	40	.50	5.32	5.18	8.64	5.08	5.10	4.92	8.50	5.28	5.28	5.12
2114	1, 1.5	1	0	80	0.16	34	46	.50	5.00	4.98	8.58	5.14	5.14	5.36	8.24	5.00	5.00	5.04
2115	1, 1.5	1	0	80	0.33	27	53	.50	5.10	5.62	8.60	5.14	5.14	5.52	8.14	4.94	4.94	5.44
2116	1, 1.5	1	0	80	0.50	20	60	.50	4.68	5.68	9.16	5.32	5.32	6.42	7.98	4.56	4.56	5.68
2117	1, 1.5	1	0	100	0	50	50	.50	5.14	5.24	8.60	5.12	5.12	4.88	8.54	5.20	5.20	5.26
2118	1, 1.5	1	0	100	0.16	42	58	.50	4.98	5.12	8.68	4.94	4.94	4.76	8.20	5.00	5.02	5.16
2119	1, 1.5	1	0	100	0.33	33	67	.50	4.98	4.94	8.50	5.00	5.00	5.26	8.00	4.84	4.84	5.04
2120	1, 1.5	1	0	100	0.50	25	75	.50	4.60	5.44	8.38	5.26	5.26	5.54	7.84	4.54	4.54	5.56
2121	1, 1.5	1	0	150	0	75	75	.50	5.62	5.56	7.72	4.56	4.56	4.46	8.88	5.40	5.40	5.48
2122	1, 1.5	1	0	150	0.16	63	87	.50	5.24	5.46	7.96	4.90	4.90	4.74	8.72	5.20	5.20	5.36
2123	1, 1.5	1	0	150	0.33	50	100	.50	5.20	5.46	8.30	4.94	4.94	5.10	8.54	5.32	5.32	5.78
2124	1, 1.5	1	0	150	0.50	37	113	.50	4.92	5.58	8.36	4.80	4.80	5.28	8.24	5.04	5.04	5.80
2125	1, 1.5	1	0	200	0	100	100	.50	5.32	5.22	8.16	5.02	5.02	4.90	8.46	5.02	5.02	5.00
2126	1, 1.5	1	0	200	0.16	84	116	.50	5.14	5.26	8.28	5.20	5.20	4.88	8.68	5.08	5.08	5.32
2127	1, 1.5	1	0	200	0.33	67	133	.50	5.30	5.76	8.44	4.84	4.84	4.90	8.74	5.46	5.46	5.64
2128	1, 1.5	1	0	200	0.50	50	150	.50	5.38	5.70	8.70	4.90	4.90	5.66	8.64	5.38	5.38	5.66
2129	1, 1.5	1.5	0	20	0	10	10	.50	4.58	4.00	9.60	5.76	5.76	5.08	8.38	4.62	4.62	4.18
2130	1, 1.5	1.5	+	20	0.16	8	12	.50	4.28	4.54	8.18	4.82	4.84	4.84	7.82	4.56	4.56	4.48
2131	1, 1.5	1.5	-	20	0.16	12	8	.50	5.38	4.62	10.78	6.86	6.88	5.28	9.62	5.24	5.26	4.54
2132	1, 1.5	1.5	+	20	0.33	7	13	.50	3.72	4.52	7.58	4.46	4.46	5.24	6.70	3.76	3.78	4.80
2133	1, 1.5	1.5	-	20	0.33	13	7	.50	6.06	5.68	11.38	7.10	7.12	6.10	10.68	6.10	6.14	5.94
2134	1, 1.5	1.5	+	20	0.50	5	15	.50	3.48	6.30	6.46	3.82	3.82	6.62	5.90	3.34	3.34	6.16
2135	1, 1.5	1.5	-	20	0.50	15	5	.50	7.60	8.42	12.66	8.18	8.22	8.06	12.26	7.60	7.60	8.62
2136	1, 1.5	1.5	0	40	0	20	20	.50	5.06	4.68	8.94	5.46	5.46	5.00	8.42	5.02	5.02	4.52
2137	1, 1.5	1.5	+	40	0.16	17	23	.50	4.28	4.76	8.16	4.82	4.82	5.28	7.48	4.08	4.10	4.54
2138	1, 1.5	1.5	-	40	0.16	23	17	.50	5.44	4.82	9.80	6.16	6.18	5.28	9.36	5.72	5.72	5.22
2139	1, 1.5	1.5	+	40	0.33	13	27	.50	3.34	4.68	6.92	4.08	4.08	5.40	6.24	3.36	3.36	4.58
2140	1, 1.5	1.5	-	40	0.33	27	13	.50	5.64	5.36	10.44	6.88	6.88	5.98	9.90	5.70	5.72	5.40
2141	1, 1.5	1.5	+	40	0.50	10	30	.50	2.68	5.64	6.14	3.16	3.16	6.12	5.50	2.92	2.92	5.76
2142	1, 1.5	1.5	-	40	0.50	30	10	.50	7.10	6.28	12.22	7.70	7.70	6.76	11.60	7.44	7.44	6.38

	D	VR	P	N	Δn	n1	n2	ε	F g	B-F g	F t	F-GG t	F-HF t	B-F t	F g x t	F-GG g x t	F-HF g x t	B-F g x t
2143	1, 1.5	1.5	0	60	0	30	30	.50	4.86	4.86	8.64	5.48	5.48	5.16	8.32	5.00	5.02	4.84
2144	1, 1.5	1.5	+	60	0.16	25	35	.50	4.10	4.66	7.90	4.56	4.58	5.06	7.26	4.30	4.30	4.86
2145	1, 1.5	1.5	-	60	0.16	35	25	.50	5.82	5.30	9.92	6.36	6.36	5.10	9.50	5.90	5.90	5.22
2146	1, 1.5	1.5	+	60	0.33	20	40	.50	3.84	5.16	6.82	4.08	4.08	5.52	6.76	3.78	3.78	5.16
2147	1, 1.5	1.5	-	60	0.33	40	20	.50	6.92	5.96	10.94	6.88	6.88	5.40	11.22	6.98	6.98	5.96
2148	1, 1.5	1.5	+	60	0.50	15	45	.50	3.42	5.44	6.00	3.20	3.20	5.96	5.62	3.24	3.24	5.44
2149	1, 1.5	1.5	-	60	0.50	45	15	.50	7.98	6.44	12.48	7.80	7.80	6.26	12.16	8.12	8.12	6.50
2150	1, 1.5	1.5	0	80	0	40	40	.50	4.98	4.86	8.50	5.04	5.04	4.84	8.56	4.96	4.96	5.02
2151	1, 1.5	1.5	+	80	0.16	34	46	.50	4.38	4.78	7.86	4.50	4.50	4.94	7.44	4.00	4.00	4.64
2152	1, 1.5	1.5	-	80	0.16	46	34	.50	5.58	4.86	9.76	5.80	5.80	4.88	9.44	5.68	5.68	5.06
2153	1, 1.5	1.5	+	80	0.33	27	53	.50	3.80	5.26	6.82	3.82	3.84	5.26	6.48	3.78	3.78	5.12
2154	1, 1.5	1.5	-	80	0.33	53	27	.50	7.00	5.26	11.40	7.16	7.16	5.36	10.90	6.70	6.70	5.46
2155	1, 1.5	1.5	+	80	0.50	20	60	.50	2.80	5.48	5.86	3.32	3.34	5.94	5.50	2.76	2.76	5.34
2156	1, 1.5	1.5	-	80	0.50	60	20	.50	8.30	6.54	12.54	8.38	8.38	6.40	12.50	8.08	8.08	6.58
2157	1, 1.5	1.5	0	100	0	50	50	.50	5.06	4.98	8.74	4.94	4.94	4.74	8.44	5.06	5.06	5.10
2158	1, 1.5	1.5	+	100	0.16	42	58	.50	4.34	4.90	7.60	4.40	4.40	4.84	7.44	4.28	4.28	5.14
2159	1, 1.5	1.5	-	100	0.16	58	42	.50	6.22	5.48	9.46	5.90	5.90	5.22	9.90	6.04	6.04	5.38
2160	1, 1.5	1.5	+	100	0.33	33	67	.50	3.68	4.84	6.50	3.64	3.64	5.18	6.12	3.34	3.34	5.02
2161	1, 1.5	1.5	-	100	0.33	67	33	.50	7.62	5.76	10.94	6.92	6.92	5.40	11.92	7.56	7.58	5.70
2162	1, 1.5	1.5	+	100	0.50	25	75	.50	2.82	5.22	5.92	3.12	3.12	5.40	5.22	2.82	2.82	5.24
2163	1, 1.5	1.5	-	100	0.50	75	25	.50	8.16	6.10	12.66	8.32	8.32	6.16	12.66	8.18	8.18	5.96
2164	1, 1.5	1.5	0	150	0	75	75	.50	5.42	5.32	7.66	4.52	4.52	4.42	8.78	5.36	5.36	5.48
2165	1, 1.5	1.5	+	150	0.16	63	87	.50	4.44	5.52	6.88	4.10	4.10	4.68	7.80	4.34	4.34	5.56
2166	1, 1.5	1.5	-	150	0.16	87	63	.50	6.30	5.46	8.40	5.22	5.22	4.44	9.90	6.24	6.24	5.40
2167	1, 1.5	1.5	+	150	0.33	50	100	.50	3.66	5.28	6.28	3.48	3.48	5.02	6.82	3.90	3.90	5.50
2168	1, 1.5	1.5	-	150	0.33	100	50	.50	6.90	5.28	10.00	5.98	5.98	4.72	10.64	6.88	6.88	5.24
2169	1, 1.5	1.5	+	150	0.50	37	113	.50	2.88	5.30	5.32	2.98	2.98	5.14	5.46	3.26	3.26	5.46
2170	1, 1.5	1.5	-	150	0.50	113	37	.50	7.38	5.08	10.98	7.14	7.14	5.08	11.22	7.54	7.54	5.02
2171	1, 1.5	1.5	0	200	0	100	100	.50	5.42	5.52	8.30	4.96	4.96	4.80	8.84	5.20	5.20	5.36
2172	1, 1.5	1.5	+	200	0.16	84	116	.50	4.32	5.50	7.28	4.30	4.30	4.90	7.62	4.42	4.42	5.30
2173	1, 1.5	1.5	-	200	0.16	116	84	.50	5.90	5.20	9.14	5.68	5.68	4.94	9.28	5.74	5.74	5.06
2174	1, 1.5	1.5	+	200	0.33	67	133	.50	3.56	5.34	6.62	3.62	3.62	4.94	6.70	3.90	3.90	5.40

	D	VR	P	N	Δn	n1	n2	ε	F g	B-F g	F t	F-GG t	F-HF t	B-F t	F g x t	F-GG g x t	F-HF g x t	B-F g x t
2175	1, 1.5	1.5	-	200	0.33	133	67	.50	6.92	5.38	10.32	6.84	6.84	5.24	10.66	6.78	6.78	5.34
2176	1, 1.5	1.5	+	200	0.50	50	150	.50	3.02	5.50	5.62	2.80	2.80	5.44	5.94	3.06	3.06	5.60
2177	1, 1.5	1.5	-	200	0.50	150	50	.50	7.68	5.78	11.92	7.94	7.94	5.66	12.12	7.84	7.84	5.74
2178	1, 1.5	2	0	20	0	10	10	.50	5.18	4.52	9.80	5.76	5.76	5.04	8.86	5.12	5.12	4.50
2179	1, 1.5	2	+	20	0.16	8	12	.50	3.84	4.44	7.70	4.42	4.44	4.66	7.36	4.18	4.18	4.48
2180	1, 1.5	2	-	20	0.16	12	8	.50	6.16	5.14	11.68	7.56	7.60	5.74	10.88	6.10	6.10	5.00
2181	1, 1.5	2	+	20	0.33	7	13	.50	3.34	4.32	6.72	3.80	3.82	4.98	6.00	3.42	3.42	4.66
2182	1, 1.5	2	-	20	0.33	13	7	.50	7.50	6.08	13.06	8.40	8.40	6.48	12.52	7.66	7.66	6.24
2183	1, 1.5	2	+	20	0.50	5	15	.50	2.76	5.62	5.10	2.78	2.82	6.20	4.72	2.72	2.74	5.54
2184	1, 1.5	2	-	20	0.50	15	5	.50	9.82	8.90	15.68	10.20	10.20	8.74	15.28	9.82	9.82	9.02
2185	1, 1.5	2	0	40	0	20	20	.50	5.08	4.76	9.12	5.56	5.56	5.24	8.48	5.02	5.04	4.88
2186	1, 1.5	2	+	40	0.16	17	23	.50	4.08	4.78	7.78	4.50	4.50	5.30	7.22	3.86	3.86	4.66
2187	1, 1.5	2	-	40	0.16	23	17	.50	6.18	5.02	10.42	6.54	6.54	5.58	10.28	6.34	6.36	5.44
2188	1, 1.5	2	+	40	0.33	13	27	.50	2.72	4.48	5.84	3.26	3.26	5.14	5.14	2.86	2.86	4.34
2189	1, 1.5	2	-	40	0.33	27	13	.50	7.04	5.72	12.66	8.10	8.10	5.92	11.96	6.96	6.96	5.66
2190	1, 1.5	2	+	40	0.50	10	30	.50	2.00	5.24	4.72	2.14	2.16	6.04	4.20	2.06	2.08	5.40
2191	1, 1.5	2	-	40	0.50	30	10	.50	9.22	6.66	15.04	10.16	10.16	6.88	14.16	9.52	9.52	6.80
2192	1, 1.5	2	0	60	0	30	30	.50	5.20	4.96	8.86	5.46	5.46	5.12	8.28	5.00	5.00	4.86
2193	1, 1.5	2	+	60	0.16	25	35	.50	3.80	4.56	7.38	4.26	4.26	5.04	6.82	3.92	3.92	4.86
2194	1, 1.5	2	-	60	0.16	35	25	.50	6.58	5.52	10.66	7.00	7.00	5.38	10.18	6.44	6.44	5.56
2195	1, 1.5	2	+	60	0.33	20	40	.50	3.16	5.00	5.80	3.30	3.30	5.32	5.52	3.16	3.16	5.06
2196	1, 1.5	2	-	60	0.33	40	20	.50	8.16	5.96	12.82	8.32	8.32	5.42	12.74	8.56	8.56	6.04
2197	1, 1.5	2	+	60	0.50	15	45	.50	2.48	5.24	4.34	2.22	2.22	5.54	4.28	2.38	2.38	5.12
2198	1, 1.5	2	-	60	0.50	45	15	.50	10.30	6.66	15.42	10.24	10.24	6.40	15.10	10.28	10.28	6.58
2199	1, 1.5	2	0	80	0	40	40	.50	4.98	4.88	8.80	5.06	5.06	4.74	8.38	5.08	5.08	4.96
2200	1, 1.5	2	+	80	0.16	34	46	.50	3.96	4.80	7.18	4.16	4.16	5.12	6.88	3.70	3.70	4.78
2201	1, 1.5	2	-	80	0.16	46	34	.50	6.32	5.02	10.72	6.32	6.32	5.06	10.52	6.24	6.24	5.02
2202	1, 1.5	2	+	80	0.33	27	53	.50	3.00	5.04	5.60	3.30	3.30	5.08	5.62	2.92	2.92	5.22
2203	1, 1.5	2	-	80	0.33	53	27	.50	8.08	5.28	12.90	8.50	8.50	5.50	12.68	8.12	8.12	5.54
2204	1, 1.5	2	+	80	0.50	20	60	.50	2.02	5.16	4.28	2.32	2.32	5.70	3.88	2.04	2.04	4.88
2205	1, 1.5	2	-	80	0.50	60	20	.50	10.56	6.74	15.42	10.50	10.50	6.64	15.16	10.56	10.56	6.80
2206	1, 1.5	2	0	100	0	50	50	.50	4.96	4.90	8.62	5.10	5.10	4.74	8.24	5.12	5.12	4.86

	D	VR	P	N	Δn	n1	n2	ε	F g	B-F g	F t	F-GG t	F-HF t	B-F t	F g x t	F-GG g x t	F-HF g x t	B-F g x t
2207	1, 1.5	2	+	100	0.16	42	58	.50	4.02	4.88	7.06	3.98	3.98	4.94	6.58	3.84	3.84	4.82
2208	1, 1.5	2	-	100	0.16	58	42	.50	6.92	5.50	9.96	6.50	6.50	5.24	10.66	6.78	6.78	5.44
2209	1, 1.5	2	+	100	0.33	33	67	.50	2.86	4.74	5.34	2.84	2.84	5.02	5.16	2.76	2.76	4.84
2210	1, 1.5	2	-	100	0.33	67	33	.50	9.10	5.94	12.80	8.36	8.36	5.42	13.40	9.20	9.20	6.00
2211	1, 1.5	2	+	100	0.50	25	75	.50	1.84	4.96	4.24	2.06	2.08	5.32	3.76	1.76	1.78	4.96
2212	1, 1.5	2	-	100	0.50	75	25	.50	10.20	6.20	14.94	10.42	10.42	6.36	15.46	10.20	10.22	6.22
2213	1, 1.5	2	0	150	0	75	75	.50	5.20	5.16	7.60	4.56	4.56	4.42	8.68	5.46	5.46	5.48
2214	1, 1.5	2	+	150	0.16	63	87	.50	3.98	5.52	6.12	3.62	3.62	4.56	7.34	4.00	4.00	5.38
2215	1, 1.5	2	-	150	0.16	87	63	.50	6.74	5.20	9.28	5.72	5.72	4.52	10.68	6.64	6.64	5.26
2216	1, 1.5	2	+	150	0.33	50	100	.50	2.80	4.92	5.10	2.78	2.78	4.76	5.58	3.00	3.00	5.34
2217	1, 1.5	2	-	150	0.33	100	50	.50	8.18	5.26	11.36	7.18	7.18	4.96	11.98	7.94	7.96	5.38
2218	1, 1.5	2	+	150	0.50	37	113	.50	2.00	5.42	4.00	2.12	2.12	5.12	3.96	2.08	2.08	5.64
2219	1, 1.5	2	-	150	0.50	113	37	.50	9.22	4.96	13.98	9.24	9.24	5.00	13.94	9.30	9.30	4.94
2220	1, 1.5	2	0	200	0	100	100	.50	5.58	5.46	8.24	4.88	4.88	4.94	8.92	5.36	5.36	5.48
2221	1, 1.5	2	+	200	0.16	84	116	.50	4.00	5.16	6.74	3.80	3.80	5.14	7.06	4.18	4.18	5.18
2222	1, 1.5	2	-	200	0.16	116	84	.50	6.58	5.12	10.02	6.32	6.32	4.92	9.94	6.32	6.32	5.16
2223	1, 1.5	2	+	200	0.33	67	133	.50	2.96	5.16	5.42	2.76	2.76	4.92	5.66	3.12	3.12	5.26
2224	1, 1.5	2	-	200	0.33	133	67	.50	8.54	5.58	11.98	8.16	8.16	5.12	12.40	8.12	8.12	5.50
2225	1, 1.5	2	+	200	0.50	50	150	.50	2.06	5.50	4.08	1.82	1.82	5.44	4.16	2.22	2.22	5.42
2226	1, 1.5	2	-	200	0.50	150	50	.50	10.04	5.78	14.52	10.22	10.22	5.68	15.14	10.12	10.12	5.72
2227	1, 1.5	5	0	20	0	10	10	.50	6.22	5.10	10.80	6.62	6.66	5.12	9.80	6.14	6.14	5.42
2228	1, 1.5	5	+	20	0.16	8	12	.50	3.68	5.02	6.56	3.74	3.76	4.96	6.62	3.88	3.88	5.02
2229	1, 1.5	5	-	20	0.16	12	8	.50	9.94	6.16	15.56	10.60	10.60	6.50	14.56	9.88	9.92	6.24
2230	1, 1.5	5	+	20	0.33	7	13	.50	2.86	4.52	4.86	2.70	2.70	4.66	4.78	2.64	2.64	4.66
2231	1, 1.5	5	-	20	0.33	13	7	.50	12.12	7.36	18.18	12.72	12.72	7.16	18.06	12.20	12.24	7.04
2232	1, 1.5	5	+	20	0.50	5	15	.50	1.64	4.56	2.46	1.38	1.40	5.14	2.90	1.50	1.50	4.78
2233	1, 1.5	5	-	20	0.50	15	5	.50	18.50	9.82	24.72	17.92	17.92	9.34	24.64	17.98	18.00	9.68
2234	1, 1.5	5	0	40	0	20	20	.50	5.68	4.98	9.16	5.70	5.70	5.18	9.32	5.60	5.60	5.10
2235	1, 1.5	5	+	40	0.16	17	23	.50	3.72	4.90	6.82	3.82	3.82	4.84	6.58	3.42	3.44	4.74
2236	1, 1.5	5	-	40	0.16	23	17	.50	8.02	5.58	12.38	8.36	8.36	5.62	12.74	8.26	8.26	5.70
2237	1, 1.5	5	+	40	0.33	13	27	.50	1.78	4.32	3.64	1.82	1.82	4.92	3.42	1.76	1.76	4.44
2238	1, 1.5	5	-	40	0.33	27	13	.50	12.18	6.08	18.34	13.14	13.14	6.08	18.14	12.48	12.48	6.26

	D	VR	P	N	Δn	n1	n2	ε	F g	B-F g	F t	F-GG t	F-HF t	B-F t	F g x t	F-GG g x t	F-HF g x t	B-F g x t
2239	1, 1.5	5	+	40	0.50	10	30	.50	1.00	4.56	1.90	1.00	1.00	5.04	1.94	.96	.96	4.44
2240	1, 1.5	5	-	40	0.50	30	10	.50	17.16	7.72	23.50	17.70	17.70	7.72	23.20	16.76	16.78	7.54
2241	1, 1.5	5	0	60	0	30	30	.50	5.20	4.76	9.26	5.72	5.72	5.14	8.76	5.50	5.50	4.92
2242	1, 1.5	5	+	60	0.16	25	35	.50	3.08	4.64	6.04	3.56	3.56	5.10	5.44	3.18	3.18	4.64
2243	1, 1.5	5	-	60	0.16	35	25	.50	8.26	5.92	12.84	8.80	8.80	5.78	12.14	8.20	8.20	5.90
2244	1, 1.5	5	+	60	0.33	20	40	.50	1.78	4.62	3.60	1.76	1.76	5.04	3.40	1.72	1.72	4.62
2245	1, 1.5	5	-	60	0.33	40	20	.50	12.60	6.14	17.62	12.74	12.74	5.86	17.58	12.58	12.58	6.20
2246	1, 1.5	5	+	60	0.50	15	45	.50	.96	4.94	1.92	.68	.68	5.20	1.92	.84	.84	4.74
2247	1, 1.5	5	-	60	0.50	45	15	.50	17.18	6.96	23.28	17.62	17.64	6.60	22.86	17.76	17.78	6.70
2248	1, 1.5	5	0	80	0	40	40	.50	4.96	4.70	8.56	5.30	5.30	4.80	8.70	5.02	5.02	4.64
2249	1, 1.5	5	+	80	0.16	34	46	.50	3.38	4.44	5.78	3.24	3.24	4.62	5.58	3.18	3.18	4.46
2250	1, 1.5	5	-	80	0.16	46	34	.50	7.94	4.98	12.90	8.14	8.14	5.16	12.88	8.24	8.24	5.16
2251	1, 1.5	5	+	80	0.33	27	53	.50	1.50	4.56	3.38	1.74	1.74	4.72	3.16	1.40	1.40	4.66
2252	1, 1.5	5	-	80	0.33	53	27	.50	11.98	5.36	17.48	12.56	12.56	5.50	17.46	12.22	12.22	5.42
2253	1, 1.5	5	+	80	0.50	20	60	.50	.74	4.66	1.76	.80	.80	5.00	1.48	.70	.70	4.76
2254	1, 1.5	5	-	80	0.50	60	20	.50	17.42	6.94	23.42	18.04	18.06	6.42	23.58	18.00	18.00	6.72
2255	1, 1.5	5	0	100	0	50	50	.50	5.02	4.68	8.82	5.14	5.14	4.76	8.44	4.92	4.92	4.68
2256	1, 1.5	5	+	100	0.16	42	58	.50	2.88	4.94	6.06	3.08	3.08	4.96	5.50	2.92	2.92	4.76
2257	1, 1.5	5	-	100	0.16	58	42	.50	8.52	5.30	12.52	7.96	7.96	5.34	13.26	8.00	8.00	5.28
2258	1, 1.5	5	+	100	0.33	33	67	.50	1.30	4.64	3.28	1.56	1.56	4.74	2.92	1.32	1.32	4.60
2259	1, 1.5	5	-	100	0.33	67	33	.50	13.06	5.92	17.48	12.78	12.80	5.34	17.80	13.08	13.08	5.82
2260	1, 1.5	5	+	100	0.50	25	75	.50	.56	4.52	1.52	.78	.78	4.96	1.44	.52	.52	4.46
2261	1, 1.5	5	-	100	0.50	75	25	.50	17.84	6.40	23.00	17.36	17.38	5.86	24.12	18.06	18.06	6.06
2262	1, 1.5	5	0	150	0	75	75	.50	5.22	4.94	7.90	4.72	4.72	4.56	8.98	5.24	5.24	5.14
2263	1, 1.5	5	+	150	0.16	63	87	.50	3.04	5.28	4.78	2.62	2.62	4.06	5.98	2.96	2.96	5.28
2264	1, 1.5	5	-	150	0.16	87	63	.50	8.28	5.22	11.48	7.76	7.78	4.62	12.24	8.48	8.48	5.08
2265	1, 1.5	5	+	150	0.33	50	100	.50	1.26	4.98	2.70	1.36	1.36	4.36	3.34	1.38	1.38	5.10
2266	1, 1.5	5	-	150	0.33	100	50	.50	11.46	5.18	16.32	10.92	10.92	4.80	16.28	11.58	11.58	5.28
2267	1, 1.5	5	+	150	0.50	37	113	.50	.62	5.12	1.44	.62	.62	4.68	1.66	.58	.58	5.10
2268	1, 1.5	5	-	150	0.50	113	37	.50	16.76	5.08	22.16	16.20	16.20	4.90	22.62	16.98	16.98	5.02
2269	1, 1.5	5	0	200	0	100	100	.50	5.34	5.28	8.56	5.04	5.04	4.90	9.10	5.60	5.60	5.40
2270	1, 1.5	5	+	200	0.16	84	116	.50	3.28	5.24	5.54	2.92	2.92	5.00	5.38	3.28	3.28	5.10

	D	VR	P	N	Δn	n1	n2	ε	F g	B-F g	F t	F-GG t	F-HF t	B-F t	F g x t	F-GG g x t	F-HF g x t	B-F g x t
2271	1, 1.5	5	-	200	0.16	116	84	.50	7.94	5.34	12.20	8.00	8.00	5.02	11.98	8.00	8.00	5.04
2272	1, 1.5	5	+	200	0.33	67	133	.50	1.54	5.12	3.10	1.36	1.36	4.80	3.36	1.64	1.64	5.30
2273	1, 1.5	5	-	200	0.33	133	67	.50	12.18	6.06	16.90	12.00	12.00	5.68	16.92	12.06	12.06	5.88
2274	1, 1.5	5	+	200	0.50	50	150	.50	.68	5.04	1.32	.46	.46	4.98	1.80	.62	.62	5.18
2275	1, 1.5	5	-	200	0.50	150	50	.50	17.34	5.92	22.32	17.18	17.18	5.58	22.84	17.64	17.64	5.86
2276	1, 1.5	1	0	20	0	10	10	.60	4.06	3.74	8.14	5.76	5.92	5.20	7.64	4.74	4.84	4.20
2277	1, 1.5	1	0	20	0.16	8	12	.60	4.62	4.40	8.02	5.60	5.74	5.56	7.18	4.68	4.82	4.34
2278	1, 1.5	1	0	20	0.33	7	13	.60	5.08	5.44	8.24	5.44	5.68	5.78	7.66	4.80	5.04	5.28
2279	1, 1.5	1	0	20	0.50	5	15	.60	4.74	7.00	8.14	5.44	5.70	7.84	7.26	4.24	4.72	7.20
2280	1, 1.5	1	0	40	0	20	20	.60	5.04	4.94	7.60	5.22	5.34	5.06	7.56	5.18	5.24	4.96
2281	1, 1.5	1	0	40	0.16	17	23	.60	5.04	5.06	7.78	5.20	5.24	5.04	7.32	4.52	4.64	4.70
2282	1, 1.5	1	0	40	0.33	13	27	.60	4.40	5.08	7.76	5.28	5.38	5.52	7.66	5.12	5.20	5.56
2283	1, 1.5	1	0	40	0.50	10	30	.60	4.32	5.74	7.46	4.94	4.94	5.88	7.62	5.14	5.16	6.34
2284	1, 1.5	1	0	60	0	30	30	.60	5.00	4.86	7.60	5.22	5.26	4.88	7.12	4.86	4.92	4.88
2285	1, 1.5	1	0	60	0.16	25	35	.60	5.16	5.10	7.50	4.96	5.00	4.92	7.64	5.26	5.30	5.08
2286	1, 1.5	1	0	60	0.33	20	40	.60	5.24	5.72	7.66	4.90	4.94	5.16	7.54	5.20	5.24	5.44
2287	1, 1.5	1	0	60	0.50	15	45	.60	4.90	6.04	7.52	4.94	5.02	5.88	7.02	4.86	4.90	5.70
2288	1, 1.5	1	0	80	0	40	40	.60	4.70	4.64	8.42	5.64	5.66	5.46	6.86	4.52	4.54	4.44
2289	1, 1.5	1	0	80	0.16	34	46	.60	4.80	5.10	7.92	5.48	5.52	5.50	7.10	4.80	4.88	5.22
2290	1, 1.5	1	0	80	0.33	27	53	.60	4.98	5.26	7.70	5.10	5.12	5.44	6.70	4.50	4.54	5.00
2291	1, 1.5	1	0	80	0.50	20	60	.60	5.42	6.10	7.92	5.22	5.30	5.84	7.54	5.02	5.08	5.98
2292	1, 1.5	1	0	100	0	50	50	.60	5.20	4.98	8.10	5.44	5.46	5.38	7.18	4.62	4.64	4.52
2293	1, 1.5	1	0	100	0.16	42	58	.60	4.88	4.92	8.00	5.44	5.48	5.38	7.08	4.52	4.54	4.56
2294	1, 1.5	1	0	100	0.33	33	67	.60	4.96	5.34	7.70	5.14	5.14	5.42	6.92	4.66	4.66	4.80
2295	1, 1.5	1	0	100	0.50	25	75	.60	4.64	5.62	7.62	5.02	5.06	5.46	7.04	4.70	4.70	5.46
2296	1, 1.5	1	0	150	0	75	75	.60	5.50	5.68	8.42	5.68	5.68	5.84	7.86	5.44	5.46	5.34
2297	1, 1.5	1	0	150	0.16	63	87	.60	5.34	5.32	8.58	5.44	5.44	5.90	7.22	4.84	4.86	4.98
2298	1, 1.5	1	0	150	0.33	50	100	.60	5.10	5.18	8.20	5.62	5.64	5.62	7.22	4.78	4.80	5.14
2299	1, 1.5	1	0	150	0.50	37	113	.60	5.20	5.80	7.84	5.22	5.26	6.04	7.02	4.58	4.58	5.10
2300	1, 1.5	1	0	200	0	100	100	.60	5.06	5.16	8.00	5.80	5.80	5.66	7.68	5.02	5.02	5.12
2301	1, 1.5	1	0	200	0.16	84	116	.60	5.34	5.38	8.28	5.72	5.76	5.60	8.02	5.30	5.30	5.60
2302	1, 1.5	1	0	200	0.33	67	133	.60	5.00	4.98	8.42	6.04	6.04	5.98	7.60	5.20	5.24	5.30

	D	VR	P	N	Δn	n1	n2	ε	F g	B-F g	F t	F-GG t	F-HF t	B-F t	F g x t	F-GG g x t	F-HF g x t	B-F g x t
2303	1, 1.5	1	0	200	0.50	50	150	.60	4.90	5.12	8.20	5.76	5.80	6.10	7.14	4.56	4.56	5.02
2304	1, 1.5	1.5	0	20	0	10	10	.60	4.08	3.90	8.04	5.88	6.04	5.22	8.16	4.84	5.06	4.40
2305	1, 1.5	1.5	+	20	0.16	8	12	.60	3.90	4.10	7.08	5.14	5.38	5.40	6.08	3.94	4.08	4.32
2306	1, 1.5	1.5	-	20	0.16	12	8	.60	5.56	4.76	9.50	6.58	6.86	5.68	9.10	6.38	6.52	5.36
2307	1, 1.5	1.5	+	20	0.33	7	13	.60	3.98	4.96	6.52	4.40	4.66	5.60	5.88	3.74	3.84	5.04
2308	1, 1.5	1.5	-	20	0.33	13	7	.60	6.10	5.66	10.06	7.02	7.34	6.46	10.28	6.70	6.94	6.06
2309	1, 1.5	1.5	+	20	0.50	5	15	.60	3.22	5.98	5.50	3.74	3.90	6.88	4.42	2.88	3.02	6.36
2310	1, 1.5	1.5	-	20	0.50	15	5	.60	7.60	8.60	11.32	7.98	8.34	8.60	10.60	7.52	7.64	7.44
2311	1, 1.5	1.5	0	40	0	20	20	.60	4.96	4.64	7.82	5.26	5.34	4.98	7.58	5.10	5.16	4.78
2312	1, 1.5	1.5	+	40	0.16	17	23	.60	4.50	4.86	6.72	4.72	4.74	5.08	6.44	4.12	4.18	4.56
2313	1, 1.5	1.5	-	40	0.16	23	17	.60	5.90	5.22	8.84	6.16	6.24	5.36	8.24	5.50	5.60	5.26
2314	1, 1.5	1.5	+	40	0.33	13	27	.60	3.12	4.72	5.82	3.70	3.74	5.28	5.74	3.80	3.92	5.14
2315	1, 1.5	1.5	-	40	0.33	27	13	.60	6.96	5.86	9.68	7.08	7.12	5.88	9.30	6.24	6.30	5.42
2316	1, 1.5	1.5	+	40	0.50	10	30	.60	2.50	5.06	4.98	3.06	3.12	5.64	5.06	2.76	2.84	5.92
2317	1, 1.5	1.5	-	40	0.50	30	10	.60	7.46	7.06	10.68	7.60	7.64	7.04	10.44	7.44	7.52	6.68
2318	1, 1.5	1.5	0	60	0	30	30	.60	5.10	4.86	7.62	5.32	5.36	5.22	7.14	4.62	4.70	4.80
2319	1, 1.5	1.5	+	60	0.16	25	35	.60	4.46	4.80	6.74	4.54	4.56	5.10	6.78	4.40	4.50	5.00
2320	1, 1.5	1.5	-	60	0.16	35	25	.60	5.68	4.68	8.84	5.78	5.86	5.16	8.26	5.54	5.56	4.90
2321	1, 1.5	1.5	+	60	0.33	20	40	.60	3.80	5.40	5.76	3.56	3.56	4.98	5.78	3.98	4.04	5.18
2322	1, 1.5	1.5	-	60	0.33	40	20	.60	6.40	5.22	10.22	6.92	7.00	5.58	9.48	6.58	6.60	5.08
2323	1, 1.5	1.5	+	60	0.50	15	45	.60	3.16	5.64	4.58	3.16	3.16	5.54	4.86	2.96	3.02	5.42
2324	1, 1.5	1.5	-	60	0.50	45	15	.60	7.56	5.88	11.02	8.24	8.30	6.66	10.74	7.66	7.70	6.44
2325	1, 1.5	1.5	0	80	0	40	40	.60	4.76	4.78	8.70	5.70	5.70	5.70	7.00	4.78	4.80	4.66
2326	1, 1.5	1.5	+	80	0.16	34	46	.60	4.20	4.76	7.28	4.88	4.90	5.62	6.48	4.36	4.38	5.18
2327	1, 1.5	1.5	-	80	0.16	46	34	.60	5.46	4.72	9.94	6.88	6.90	5.80	7.78	5.36	5.40	4.76
2328	1, 1.5	1.5	+	80	0.33	27	53	.60	3.56	4.94	6.08	3.98	4.02	5.40	5.14	3.38	3.40	4.86
2329	1, 1.5	1.5	-	80	0.33	53	27	.60	6.32	4.94	10.84	8.10	8.10	6.18	9.40	6.42	6.46	4.88
2330	1, 1.5	1.5	+	80	0.50	20	60	.60	3.40	5.76	4.98	3.28	3.28	5.64	5.14	3.16	3.20	5.54
2331	1, 1.5	1.5	-	80	0.50	60	20	.60	7.32	5.58	11.88	8.64	8.70	6.64	10.64	7.50	7.52	5.68
2332	1, 1.5	1.5	0	100	0	50	50	.60	5.14	4.98	8.02	5.56	5.58	5.44	7.42	4.50	4.54	4.62
2333	1, 1.5	1.5	+	100	0.16	42	58	.60	4.36	4.92	7.26	4.52	4.60	5.48	6.10	4.00	4.02	4.56
2334	1, 1.5	1.5	-	100	0.16	58	42	.60	5.84	5.02	9.10	6.32	6.34	5.72	8.12	5.50	5.52	4.84

	D	VR	P	N	Δn	n1	n2	ε	F g	B-F g	F t	F-GG t	F-HF t	B-F t	F g x t	F-GG g x t	F-HF g x t	B-F g x t
2335	1, 1.5	1.5	+	100	0.33	33	67	.60	3.34	5.32	5.90	3.78	3.82	5.22	5.24	3.44	3.46	4.80
2336	1, 1.5	1.5	-	100	0.33	67	33	.60	6.96	5.40	9.66	6.82	6.84	5.30	9.18	6.68	6.70	5.02
2337	1, 1.5	1.5	+	100	0.50	25	75	.60	2.76	5.32	4.82	2.96	2.96	5.44	4.52	2.94	2.96	5.24
2338	1, 1.5	1.5	-	100	0.50	75	25	.60	8.14	5.84	10.10	7.22	7.24	5.34	10.72	7.72	7.72	5.54
2339	1, 1.5	1.5	0	150	0	75	75	.60	5.66	5.64	8.60	5.58	5.62	5.64	7.90	5.50	5.52	5.38
2340	1, 1.5	1.5	+	150	0.16	63	87	.60	4.54	5.26	7.26	4.94	4.96	5.66	6.48	4.36	4.36	5.16
2341	1, 1.5	1.5	-	150	0.16	87	63	.60	5.52	5.04	9.38	6.46	6.46	5.34	9.10	6.28	6.30	5.44
2342	1, 1.5	1.5	+	150	0.33	50	100	.60	3.74	5.10	6.16	4.14	4.18	5.60	5.50	3.30	3.30	5.24
2343	1, 1.5	1.5	-	150	0.33	100	50	.60	6.40	5.06	10.26	7.20	7.20	5.70	9.54	6.84	6.88	5.18
2344	1, 1.5	1.5	+	150	0.50	37	113	.60	3.08	5.70	5.10	3.36	3.36	6.00	4.34	2.82	2.82	4.94
2345	1, 1.5	1.5	-	150	0.50	113	37	.60	7.44	5.54	11.46	8.00	8.00	5.48	10.02	7.28	7.32	5.20
2346	1, 1.5	1.5	0	200	0	100	100	.60	5.16	4.96	7.96	5.70	5.70	5.62	7.50	5.04	5.06	5.00
2347	1, 1.5	1.5	+	200	0.16	84	116	.60	4.54	5.32	7.14	4.88	4.88	5.80	7.06	4.48	4.48	5.40
2348	1, 1.5	1.5	-	200	0.16	116	84	.60	5.90	4.88	8.86	6.40	6.40	5.58	8.54	5.92	5.92	5.02
2349	1, 1.5	1.5	+	200	0.33	67	133	.60	3.52	4.88	6.42	4.18	4.20	5.96	5.94	3.72	3.72	5.32
2350	1, 1.5	1.5	-	200	0.33	133	67	.60	6.66	5.22	10.52	7.38	7.42	5.52	10.56	7.24	7.30	5.44
2351	1, 1.5	1.5	+	200	0.50	50	150	.60	2.96	5.08	5.58	3.68	3.68	6.30	4.54	2.60	2.60	4.82
2352	1, 1.5	1.5	-	200	0.50	150	50	.60	7.86	5.42	11.98	8.66	8.66	5.86	11.50	8.04	8.08	5.50
2353	1, 1.5	2	0	20	0	10	10	.60	4.34	4.04	8.26	5.92	6.06	5.38	8.08	5.16	5.42	4.72
2354	1, 1.5	2	+	20	0.16	8	12	.60	3.50	4.10	6.60	4.74	4.90	5.52	5.62	3.56	3.78	4.40
2355	1, 1.5	2	-	20	0.16	12	8	.60	6.48	4.92	10.64	7.66	7.86	5.88	10.22	7.10	7.36	5.46
2356	1, 1.5	2	+	20	0.33	7	13	.60	3.34	4.70	5.72	3.98	4.16	5.48	5.12	3.24	3.34	4.90
2357	1, 1.5	2	-	20	0.33	13	7	.60	7.38	6.42	11.54	8.60	8.94	6.80	11.70	8.12	8.34	6.34
2358	1, 1.5	2	+	20	0.50	5	15	.60	2.36	5.48	4.24	2.90	3.08	6.50	3.34	2.30	2.36	5.68
2359	1, 1.5	2	-	20	0.50	15	5	.60	10.10	9.34	14.04	10.34	10.50	9.12	13.54	9.90	10.20	7.92
2360	1, 1.5	2	0	40	0	20	20	.60	5.20	4.54	7.80	5.46	5.52	5.06	7.52	5.16	5.24	5.06
2361	1, 1.5	2	+	40	0.16	17	23	.60	4.22	4.82	6.42	4.24	4.30	4.92	6.12	3.64	3.76	4.46
2362	1, 1.5	2	-	40	0.16	23	17	.60	6.32	5.42	9.56	6.68	6.72	5.44	8.78	5.98	6.04	5.16
2363	1, 1.5	2	+	40	0.33	13	27	.60	2.62	4.74	4.76	2.82	2.84	5.20	4.64	2.70	2.76	4.92
2364	1, 1.5	2	-	40	0.33	27	13	.60	8.20	5.90	11.52	8.46	8.60	6.06	10.88	8.00	8.08	5.80
2365	1, 1.5	2	+	40	0.50	10	30	.60	1.80	4.64	3.34	2.22	2.22	5.68	3.20	2.08	2.10	5.50
2366	1, 1.5	2	-	40	0.50	30	10	.60	9.70	7.12	13.24	9.88	10.00	7.16	13.54	9.60	9.66	7.04

	D	VR	P	N	Δn	n1	n2	ε	F g	B-F g	F t	F-GG t	F-HF t	B-F t	F g x t	F-GG g x t	F-HF g x t	B-F g x t
2367	1, 1.5	2	0	60	0	30	30	.60	4.72	4.62	7.62	5.46	5.54	5.20	7.28	4.72	4.76	4.84
2368	1, 1.5	2	+	60	0.16	25	35	.60	4.18	4.94	6.26	4.04	4.06	5.10	6.20	4.02	4.06	4.84
2369	1, 1.5	2	-	60	0.16	35	25	.60	6.10	4.76	9.74	6.50	6.54	5.26	9.34	6.28	6.36	5.30
2370	1, 1.5	2	+	60	0.33	20	40	.60	2.98	5.14	4.66	2.88	2.94	4.84	5.00	3.54	3.56	5.12
2371	1, 1.5	2	-	60	0.33	40	20	.60	7.88	5.44	11.80	8.52	8.56	5.80	11.22	8.14	8.18	5.50
2372	1, 1.5	2	+	60	0.50	15	45	.60	2.28	5.46	3.54	1.96	1.96	5.32	3.60	1.96	1.98	5.28
2373	1, 1.5	2	-	60	0.50	45	15	.60	9.60	6.06	13.68	10.36	10.46	6.60	13.12	9.78	9.80	6.54
2374	1, 1.5	2	0	80	0	40	40	.60	4.74	4.74	8.64	6.04	6.04	5.80	7.18	4.86	4.90	4.84
2375	1, 1.5	2	+	80	0.16	34	46	.60	3.88	4.70	6.98	4.48	4.56	5.52	6.06	4.02	4.04	5.02
2376	1, 1.5	2	-	80	0.16	46	34	.60	6.08	5.02	10.56	7.60	7.68	6.00	8.78	6.04	6.08	5.30
2377	1, 1.5	2	+	80	0.33	27	53	.60	3.06	4.90	5.08	3.24	3.24	5.46	4.38	2.72	2.72	4.76
2378	1, 1.5	2	-	80	0.33	53	27	.60	7.70	5.10	12.46	9.22	9.26	6.28	10.84	7.78	7.80	4.82
2379	1, 1.5	2	+	80	0.50	20	60	.60	2.26	5.60	3.60	2.30	2.32	5.54	3.80	2.32	2.34	5.40
2380	1, 1.5	2	-	80	0.50	60	20	.60	9.40	5.76	14.92	11.10	11.10	6.56	13.40	9.84	9.92	5.64
2381	1, 1.5	2	0	100	0	50	50	.60	5.10	4.92	8.08	5.66	5.68	5.46	7.50	4.76	4.78	4.66
2382	1, 1.5	2	+	100	0.16	42	58	.60	4.06	5.10	6.58	4.26	4.28	5.68	5.68	3.72	3.72	4.72
2383	1, 1.5	2	-	100	0.16	58	42	.60	6.22	5.20	9.58	7.12	7.12	5.64	8.88	6.02	6.02	4.88
2384	1, 1.5	2	+	100	0.33	33	67	.60	2.60	5.24	4.76	2.98	2.98	5.32	4.34	2.60	2.72	4.96
2385	1, 1.5	2	-	100	0.33	67	33	.60	8.30	5.38	11.72	7.90	7.96	5.22	11.12	7.84	7.84	5.06
2386	1, 1.5	2	+	100	0.50	25	75	.60	1.94	4.96	3.38	2.16	2.20	5.14	3.52	1.96	1.98	5.16
2387	1, 1.5	2	-	100	0.50	75	25	.60	10.14	6.30	13.02	9.28	9.28	5.66	13.44	9.74	9.82	5.74
2388	1, 1.5	2	0	150	0	75	75	.60	5.44	5.28	8.54	5.52	5.54	5.58	7.94	5.62	5.68	5.76
2389	1, 1.5	2	+	150	0.16	63	87	.60	4.24	5.38	7.00	4.42	4.42	5.66	6.10	3.92	3.94	5.14
2390	1, 1.5	2	-	150	0.16	87	63	.60	6.34	4.92	10.12	7.06	7.08	5.46	9.98	7.02	7.02	5.86
2391	1, 1.5	2	+	150	0.33	50	100	.60	2.94	5.08	5.10	3.24	3.26	5.64	4.44	2.70	2.70	5.24
2392	1, 1.5	2	-	150	0.33	100	50	.60	7.86	5.04	11.88	8.44	8.46	5.64	11.04	8.04	8.08	5.32
2393	1, 1.5	2	+	150	0.50	37	113	.60	1.96	5.44	3.96	2.26	2.26	5.86	3.00	2.02	2.02	5.00
2394	1, 1.5	2	-	150	0.50	113	37	.60	9.78	5.62	14.12	10.58	10.58	5.54	12.68	9.36	9.38	5.22
2395	1, 1.5	2	0	200	0	100	100	.60	5.16	5.00	8.08	5.58	5.58	5.38	7.56	5.18	5.18	5.18
2396	1, 1.5	2	+	200	0.16	84	116	.60	4.06	5.32	6.80	4.36	4.36	5.68	6.62	3.92	3.92	5.32
2397	1, 1.5	2	-	200	0.16	116	84	.60	6.34	5.10	9.74	6.96	6.98	5.60	9.24	6.46	6.48	5.52
2398	1, 1.5	2	+	200	0.33	67	133	.60	2.96	4.84	5.34	3.28	3.28	5.62	4.72	2.96	2.98	5.10

	D	VR	P	N	Δn	n1	n2	ε	F g	B-F g	F t	F-GG t	F-HF t	B-F t	F g x t	F-GG g x t	F-HF g x t	B-F g x t
2399	1, 1.5	2	-	200	0.33	133	67	.60	7.90	5.34	12.20	8.98	9.04	5.76	12.18	8.56	8.58	5.54
2400	1, 1.5	2	+	200	0.50	50	150	.60	2.00	5.10	3.94	2.42	2.42	6.02	3.08	1.76	1.76	4.76
2401	1, 1.5	2	-	200	0.50	150	50	.60	10.16	5.38	14.52	10.98	10.98	5.98	14.06	10.62	10.66	5.60
2402	1, 1.5	5	0	20	0	10	10	.60	5.30	4.48	9.22	6.58	6.68	5.78	9.18	6.24	6.46	5.48
2403	1, 1.5	5	+	20	0.16	8	12	.60	3.40	4.54	5.46	3.92	4.10	5.34	4.66	2.96	3.14	4.46
2404	1, 1.5	5	-	20	0.16	12	8	.60	9.38	5.70	13.98	10.42	10.62	6.42	13.56	10.22	10.42	6.06
2405	1, 1.5	5	+	20	0.33	7	13	.60	2.76	4.92	4.28	2.88	2.92	5.56	3.58	2.46	2.56	4.68
2406	1, 1.5	5	-	20	0.33	13	7	.60	12.14	7.04	16.14	11.98	12.44	6.90	16.70	12.58	12.90	6.72
2407	1, 1.5	5	+	20	0.50	5	15	.60	1.28	4.78	1.88	1.06	1.14	5.72	1.58	1.06	1.12	4.60
2408	1, 1.5	5	-	20	0.50	15	5	.60	18.52	9.82	23.52	19.06	19.36	9.52	23.10	18.54	18.90	8.84
2409	1, 1.5	5	0	40	0	20	20	.60	5.66	4.74	8.36	5.78	5.88	5.20	7.94	5.52	5.58	5.04
2410	1, 1.5	5	+	40	0.16	17	23	.60	3.64	4.80	5.12	3.36	3.42	4.54	4.94	3.18	3.20	4.58
2411	1, 1.5	5	-	40	0.16	23	17	.60	7.76	5.34	11.68	8.52	8.62	5.52	11.30	8.06	8.14	5.18
2412	1, 1.5	5	+	40	0.33	13	27	.60	1.54	4.32	2.66	1.44	1.46	4.88	2.48	1.60	1.62	4.58
2413	1, 1.5	5	-	40	0.33	27	13	.60	12.76	5.92	17.06	13.12	13.30	6.10	16.66	12.72	12.86	5.78
2414	1, 1.5	5	+	40	0.50	10	30	.60	.86	4.04	1.24	.72	.74	5.22	1.36	.86	.88	4.88
2415	1, 1.5	5	-	40	0.50	30	10	.60	16.68	6.74	22.42	17.58	17.74	6.96	22.20	17.42	17.54	7.00
2416	1, 1.5	5	0	60	0	30	30	.60	4.76	4.42	8.06	5.46	5.50	5.08	7.56	5.16	5.20	4.86
2417	1, 1.5	5	+	60	0.16	25	35	.60	3.18	4.86	5.08	3.26	3.26	4.98	5.28	3.20	3.22	5.24
2418	1, 1.5	5	-	60	0.16	35	25	.60	7.78	4.96	12.38	8.60	8.66	5.62	12.24	8.72	8.74	5.68
2419	1, 1.5	5	+	60	0.33	20	40	.60	1.76	4.94	2.80	1.74	1.74	4.98	3.14	1.80	1.88	5.18
2420	1, 1.5	5	-	60	0.33	40	20	.60	12.02	5.28	16.50	13.04	13.08	5.94	16.82	13.08	13.16	5.60
2421	1, 1.5	5	+	60	0.50	15	45	.60	.78	4.94	1.32	.82	.82	5.06	1.34	.78	.80	4.84
2422	1, 1.5	5	-	60	0.50	45	15	.60	16.70	6.10	21.70	17.26	17.32	6.34	21.92	17.26	17.32	6.60
2423	1, 1.5	5	0	80	0	40	40	.60	5.20	4.88	8.84	6.12	6.16	5.68	7.80	5.54	5.58	5.20
2424	1, 1.5	5	+	80	0.16	34	46	.60	3.08	4.48	5.92	3.82	3.86	5.84	5.22	3.26	3.26	5.04
2425	1, 1.5	5	-	80	0.16	46	34	.60	7.70	5.32	12.50	9.04	9.08	6.08	11.08	8.14	8.18	5.46
2426	1, 1.5	5	+	80	0.33	27	53	.60	1.56	4.68	3.02	1.88	1.90	5.48	2.76	1.68	1.74	4.68
2427	1, 1.5	5	-	80	0.33	53	27	.60	11.66	4.92	16.78	12.98	13.06	6.14	15.88	12.04	12.12	5.38
2428	1, 1.5	5	+	80	0.50	20	60	.60	.76	5.32	1.52	.84	.86	5.62	1.28	.64	.64	5.38
2429	1, 1.5	5	-	80	0.50	60	20	.60	16.34	5.66	23.44	18.98	19.12	6.32	21.96	17.72	17.76	5.84
2430	1, 1.5	5	0	100	0	50	50	.60	5.14	5.08	8.40	5.54	5.60	5.38	7.70	5.22	5.24	5.06

	D	VR	P	N	Δn	n1	n2	ε	F g	B-F g	F t	F-GG t	F-HF t	B-F t	F g x t	F-GG g x t	F-HF g x t	B-F g x t
2431	1, 1.5	5	+	100	0.16	42	58	.60	3.00	4.78	5.00	3.16	3.16	5.46	4.94	3.06	3.10	5.14
2432	1, 1.5	5	-	100	0.16	58	42	.60	8.06	5.02	11.60	8.48	8.54	5.22	11.36	8.00	8.02	4.56
2433	1, 1.5	5	+	100	0.33	33	67	.60	1.22	4.88	2.60	1.56	1.56	5.28	2.52	1.42	1.42	5.24
2434	1, 1.5	5	-	100	0.33	67	33	.60	12.10	5.50	16.28	12.50	12.52	5.18	16.22	12.34	12.36	5.04
2435	1, 1.5	5	+	100	0.50	25	75	.60	.62	4.84	1.20	.66	.66	5.36	1.18	.52	.52	4.92
2436	1, 1.5	5	-	100	0.50	75	25	.60	17.64	5.86	20.90	16.58	16.66	5.26	22.54	17.50	17.58	5.46
2437	1, 1.5	5	0	150	0	75	75	.60	5.88	5.52	8.08	5.64	5.66	5.54	8.36	5.84	5.84	5.82
2438	1, 1.5	5	+	150	0.16	63	87	.60	3.46	5.24	5.44	3.30	3.32	5.50	5.14	3.40	3.40	5.26
2439	1, 1.5	5	-	150	0.16	87	63	.60	8.20	4.94	11.98	8.58	8.60	5.78	11.78	8.94	8.96	5.86
2440	1, 1.5	5	+	150	0.33	50	100	.60	1.52	4.78	3.06	1.54	1.54	5.72	2.68	1.44	1.44	5.28
2441	1, 1.5	5	-	150	0.33	100	50	.60	11.66	4.92	16.68	12.48	12.52	5.50	15.84	11.92	11.92	5.48
2442	1, 1.5	5	+	150	0.50	37	113	.60	.54	5.04	1.14	.70	.70	5.62	1.20	.74	.74	4.98
2443	1, 1.5	5	-	150	0.50	113	37	.60	17.40	5.70	22.04	17.64	17.68	5.30	21.86	17.22	17.26	5.40
2444	1, 1.5	5	0	200	0	100	100	.60	5.02	5.04	7.84	5.64	5.64	5.24	7.66	5.32	5.32	5.18
2445	1, 1.5	5	+	200	0.16	84	116	.60	2.94	4.88	5.24	3.16	3.18	5.60	4.96	3.18	3.20	5.38
2446	1, 1.5	5	-	200	0.16	116	84	.60	7.76	5.00	11.84	8.56	8.58	5.40	11.10	7.90	7.90	5.34
2447	1, 1.5	5	+	200	0.33	67	133	.60	1.64	4.84	2.90	1.74	1.74	5.56	2.66	1.72	1.72	5.00
2448	1, 1.5	5	-	200	0.33	133	67	.60	12.02	5.08	16.88	13.56	13.60	5.70	16.86	13.18	13.24	5.58
2449	1, 1.5	5	+	200	0.50	50	150	.60	.48	4.74	1.54	.76	.76	5.70	1.04	.48	.48	4.78
2450	1, 1.5	5	-	200	0.50	150	50	.60	17.22	5.18	22.70	18.30	18.32	5.84	22.44	18.30	18.32	5.92
2451	1, 1.5	1	0	20	0	10	10	.70	4.12	3.74	6.74	4.90	5.32	4.72	6.74	4.92	5.32	4.80
2452	1, 1.5	1	0	20	0.16	8	12	.70	4.66	4.42	6.48	4.56	4.90	4.64	6.30	4.42	4.78	4.40
2453	1, 1.5	1	0	20	0.33	7	13	.70	5.16	4.82	6.64	4.70	5.00	4.76	6.52	4.66	5.02	4.52
2454	1, 1.5	1	0	20	0.50	5	15	.70	4.84	7.40	6.78	4.92	5.30	6.98	6.72	4.68	4.96	6.40
2455	1, 1.5	1	0	40	0	20	20	.70	5.06	4.82	6.76	4.98	5.10	4.76	6.40	4.58	4.78	4.48
2456	1, 1.5	1	0	40	0.16	17	23	.70	4.82	4.68	6.56	5.06	5.16	4.94	6.24	4.60	4.68	4.64
2457	1, 1.5	1	0	40	0.33	13	27	.70	4.52	4.86	7.08	4.98	5.22	5.40	6.36	4.76	4.84	4.72
2458	1, 1.5	1	0	40	0.50	10	30	.70	4.30	5.88	6.66	4.96	5.04	6.40	6.46	4.64	4.78	6.02
2459	1, 1.5	1	0	60	0	30	30	.70	4.58	4.44	7.06	5.12	5.16	5.18	7.36	5.14	5.26	5.36
2460	1, 1.5	1	0	60	0.16	25	35	.70	4.78	4.84	6.88	5.08	5.22	5.22	6.88	5.04	5.16	5.08
2461	1, 1.5	1	0	60	0.33	20	40	.70	5.34	5.30	6.88	5.12	5.24	5.38	6.36	4.80	4.84	4.90
2462	1, 1.5	1	0	60	0.50	15	45	.70	4.96	5.92	6.80	5.06	5.20	5.54	6.48	4.78	4.84	5.32

	D	VR	P	N	Δn	n1	n2	ε	F g	B-F g	F t	F-GG t	F-HF t	B-F t	F g x t	F-GG g x t	F-HF g x t	B-F g x t
2463	1, 1.5	1	0	80	0	40	40	.70	4.42	4.34	7.22	5.60	5.66	5.58	6.36	5.06	5.10	5.08
2464	1, 1.5	1	0	80	0.16	34	46	.70	4.78	4.96	7.50	5.46	5.54	5.68	6.78	5.10	5.10	5.52
2465	1, 1.5	1	0	80	0.33	27	53	.70	4.96	5.38	7.24	5.54	5.56	5.72	6.78	5.00	5.02	5.00
2466	1, 1.5	1	0	80	0.50	20	60	.70	5.12	6.08	7.30	5.50	5.54	6.16	6.60	4.92	4.98	5.36
2467	1, 1.5	1	0	100	0	50	50	.70	4.84	4.98	7.38	5.70	5.72	5.98	6.56	4.80	4.80	5.00
2468	1, 1.5	1	0	100	0.16	42	58	.70	4.90	4.88	7.42	5.72	5.76	5.96	6.34	4.76	4.82	5.22
2469	1, 1.5	1	0	100	0.33	33	67	.70	4.96	5.52	7.20	5.40	5.40	5.56	6.78	4.96	5.02	5.34
2470	1, 1.5	1	0	100	0.50	25	75	.70	4.76	5.42	6.96	5.36	5.36	6.08	6.52	4.58	4.62	5.16
2471	1, 1.5	1	0	150	0	75	75	.70	5.66	5.78	6.94	5.10	5.10	5.26	7.36	5.64	5.68	5.68
2472	1, 1.5	1	0	150	0.16	63	87	.70	5.56	5.34	7.18	5.12	5.16	5.60	6.64	4.72	4.74	4.82
2473	1, 1.5	1	0	150	0.33	50	100	.70	4.82	5.20	6.98	5.24	5.26	5.54	6.94	5.18	5.26	5.50
2474	1, 1.5	1	0	150	0.50	37	113	.70	4.92	5.68	6.64	5.04	5.08	5.44	7.22	5.06	5.10	5.68
2475	1, 1.5	1	0	200	0	100	100	.70	5.18	5.08	6.74	4.92	4.92	4.92	6.70	4.86	4.90	5.06
2476	1, 1.5	1	0	200	0.16	84	116	.70	5.10	5.10	6.94	5.10	5.12	5.48	7.26	5.14	5.14	5.30
2477	1, 1.5	1	0	200	0.33	67	133	.70	5.08	5.34	6.74	5.20	5.24	5.32	7.04	5.30	5.32	5.34
2478	1, 1.5	1	0	200	0.50	50	150	.70	4.58	5.08	6.76	4.94	4.98	5.42	6.90	5.18	5.20	5.50
2479	1, 1.5	1.5	0	20	0	10	10	.70	4.36	3.84	6.68	5.10	5.28	4.70	6.92	5.00	5.32	4.64
2480	1, 1.5	1.5	+	20	0.16	8	12	.70	4.04	4.26	5.60	3.98	4.24	4.68	5.58	3.86	4.16	4.46
2481	1, 1.5	1.5	-	20	0.16	12	8	.70	6.06	5.26	7.88	5.86	6.16	4.62	8.06	5.94	6.16	4.68
2482	1, 1.5	1.5	+	20	0.33	7	13	.70	4.02	5.18	5.10	3.46	3.74	4.86	4.96	3.38	3.68	4.68
2483	1, 1.5	1.5	-	20	0.33	13	7	.70	6.12	5.40	8.52	6.40	6.62	5.34	8.14	6.28	6.70	5.22
2484	1, 1.5	1.5	+	20	0.50	5	15	.70	3.36	6.52	4.26	3.18	3.30	6.00	4.02	2.82	3.04	6.02
2485	1, 1.5	1.5	-	20	0.50	15	5	.70	7.22	8.18	10.20	7.60	8.00	7.46	10.40	8.04	8.56	7.72
2486	1, 1.5	1.5	0	40	0	20	20	.70	5.02	4.80	7.00	5.00	5.12	4.90	6.20	4.56	4.70	4.48
2487	1, 1.5	1.5	+	40	0.16	17	23	.70	4.32	4.50	6.00	4.32	4.42	4.88	5.54	3.90	4.06	4.44
2488	1, 1.5	1.5	-	40	0.16	23	17	.70	5.60	5.00	8.00	5.86	5.98	5.26	7.68	5.68	5.82	5.14
2489	1, 1.5	1.5	+	40	0.33	13	27	.70	3.20	4.66	5.08	3.42	3.58	5.08	4.76	3.32	3.48	4.74
2490	1, 1.5	1.5	-	40	0.33	27	13	.70	6.12	5.40	9.08	7.06	7.14	5.86	8.76	6.46	6.52	5.60
2491	1, 1.5	1.5	+	40	0.50	10	30	.70	2.66	5.44	4.32	3.04	3.06	6.14	3.80	2.70	2.86	5.84
2492	1, 1.5	1.5	-	40	0.50	30	10	.70	7.18	6.72	9.70	7.74	7.96	6.52	10.18	7.72	7.90	6.56
2493	1, 1.5	1.5	0	60	0	30	30	.70	4.38	4.40	7.02	5.32	5.36	5.40	7.38	5.32	5.40	5.56
2494	1, 1.5	1.5	+	60	0.16	25	35	.70	4.02	4.58	5.98	4.06	4.08	5.00	5.88	4.44	4.46	5.14

	D	VR	P	N	Δn	n1	n2	ε	F g	B-F g	F t	F-GG t	F-HF t	B-F t	F g x t	F-GG g x t	F-HF g x t	B-F g x t
2495	1, 1.5	1.5	-	60	0.16	35	25	.70	5.66	4.58	8.04	5.98	6.06	5.22	7.90	5.70	5.82	4.82
2496	1, 1.5	1.5	+	60	0.33	20	40	.70	3.86	5.10	5.02	3.86	3.96	5.24	4.80	3.56	3.62	4.92
2497	1, 1.5	1.5	-	60	0.33	40	20	.70	6.44	5.30	8.74	6.90	6.96	5.70	9.24	6.78	6.94	5.34
2498	1, 1.5	1.5	+	60	0.50	15	45	.70	3.06	5.56	4.24	3.18	3.18	5.50	4.00	2.82	2.88	4.96
2499	1, 1.5	1.5	-	60	0.50	45	15	.70	7.36	6.12	10.48	8.10	8.20	5.80	10.28	8.04	8.16	5.82
2500	1, 1.5	1.5	0	80	0	40	40	.70	4.24	4.24	7.22	5.38	5.44	5.40	6.32	4.76	4.80	4.80
2501	1, 1.5	1.5	+	80	0.16	34	46	.70	4.08	4.68	6.54	4.74	4.82	5.84	5.82	4.20	4.30	5.42
2502	1, 1.5	1.5	-	80	0.16	46	34	.70	5.60	4.78	8.16	6.04	6.10	5.42	7.64	5.56	5.62	5.00
2503	1, 1.5	1.5	+	80	0.33	27	53	.70	3.36	5.06	5.48	4.00	4.02	5.70	4.90	3.26	3.28	5.08
2504	1, 1.5	1.5	-	80	0.33	53	27	.70	6.44	5.08	9.28	7.32	7.44	5.70	8.82	6.64	6.70	5.22
2505	1, 1.5	1.5	+	80	0.50	20	60	.70	3.08	5.96	4.74	3.44	3.48	6.02	4.08	2.66	2.70	5.28
2506	1, 1.5	1.5	-	80	0.50	60	20	.70	7.46	5.68	10.80	8.64	8.72	6.32	10.26	8.00	8.04	5.92
2507	1, 1.5	1.5	0	100	0	50	50	.70	4.80	4.88	7.06	5.62	5.64	5.54	6.56	4.90	4.92	5.00
2508	1, 1.5	1.5	+	100	0.16	42	58	.70	4.02	5.04	6.72	5.02	5.06	5.96	5.42	4.16	4.20	4.92
2509	1, 1.5	1.5	-	100	0.16	58	42	.70	5.80	5.16	7.98	5.96	5.98	5.36	7.34	5.56	5.64	4.90
2510	1, 1.5	1.5	+	100	0.33	33	67	.70	3.54	5.20	5.40	4.24	4.26	5.88	4.82	3.42	3.42	5.20
2511	1, 1.5	1.5	-	100	0.33	67	33	.70	6.94	5.24	8.94	6.82	6.86	5.14	9.38	6.86	6.92	4.86
2512	1, 1.5	1.5	+	100	0.50	25	75	.70	2.90	5.30	4.74	3.44	3.52	5.88	3.70	2.28	2.30	4.76
2513	1, 1.5	1.5	-	100	0.50	75	25	.70	7.76	5.88	10.40	8.12	8.16	5.78	10.18	7.86	7.92	5.50
2514	1, 1.5	1.5	0	150	0	75	75	.70	5.68	5.64	6.58	5.08	5.12	5.08	7.30	5.36	5.42	5.64
2515	1, 1.5	1.5	+	150	0.16	63	87	.70	4.84	5.48	5.84	4.48	4.50	5.46	5.54	3.94	3.98	4.84
2516	1, 1.5	1.5	-	150	0.16	87	63	.70	6.28	5.42	7.58	5.72	5.76	5.26	7.94	6.10	6.20	5.38
2517	1, 1.5	1.5	+	150	0.33	50	100	.70	3.56	4.98	5.24	3.90	3.90	5.46	5.18	3.80	3.82	5.46
2518	1, 1.5	1.5	-	150	0.33	100	50	.70	6.70	5.04	8.86	6.72	6.80	5.04	8.70	6.90	6.94	4.74
2519	1, 1.5	1.5	+	150	0.50	37	113	.70	2.90	5.54	4.40	3.14	3.18	5.56	4.48	3.18	3.18	5.58
2520	1, 1.5	1.5	-	150	0.50	113	37	.70	7.62	5.32	9.96	7.64	7.64	5.10	9.76	7.16	7.16	5.10
2521	1, 1.5	1.5	0	200	0	100	100	.70	5.20	4.96	6.80	4.86	4.86	5.08	6.34	5.12	5.12	5.14
2522	1, 1.5	1.5	+	200	0.16	84	116	.70	4.50	5.40	5.94	4.10	4.14	5.36	6.44	4.34	4.40	5.72
2523	1, 1.5	1.5	-	200	0.16	116	84	.70	5.96	5.04	7.80	5.74	5.74	4.90	7.92	5.92	5.92	4.98
2524	1, 1.5	1.5	+	200	0.33	67	133	.70	3.78	5.26	5.04	3.68	3.68	5.12	5.14	3.70	3.72	5.08
2525	1, 1.5	1.5	-	200	0.33	133	67	.70	6.74	5.52	8.58	6.78	6.80	5.16	8.90	6.96	6.96	5.82
2526	1, 1.5	1.5	+	200	0.50	50	150	.70	2.94	5.26	4.10	2.86	2.92	5.36	4.14	3.06	3.08	5.60

	D	VR	P	N	Δn	n1	n2	ε	F g	B-F g	F t	F-GG t	F-HF t	B-F t	F g x t	F-GG g x t	F-HF g x t	B-F g x t
2527	1, 1.5	1.5	-	200	0.50	150	50	.70	7.76	5.58	10.08	7.66	7.68	5.30	10.46	8.20	8.22	5.24
2528	1, 1.5	2	0	20	0	10	10	.70	4.50	3.94	6.68	5.20	5.46	4.58	7.08	5.14	5.50	4.66
2529	1, 1.5	2	+	20	0.16	8	12	.70	3.80	4.34	5.22	3.58	3.88	4.78	4.98	3.56	3.70	4.58
2530	1, 1.5	2	-	20	0.16	12	8	.70	6.78	5.26	9.06	6.84	7.24	4.94	8.96	6.62	6.96	4.74
2531	1, 1.5	2	+	20	0.33	7	13	.70	3.30	4.90	4.10	2.92	3.06	4.58	4.10	3.00	3.20	4.56
2532	1, 1.5	2	-	20	0.33	13	7	.70	7.46	5.86	10.16	7.60	7.98	5.48	9.68	7.42	7.84	5.44
2533	1, 1.5	2	+	20	0.50	5	15	.70	2.46	6.26	3.22	2.04	2.26	5.96	2.80	2.04	2.18	5.74
2534	1, 1.5	2	-	20	0.50	15	5	.70	9.60	8.68	13.14	9.92	10.50	7.46	13.32	10.50	10.76	7.86
2535	1, 1.5	2	0	40	0	20	20	.70	4.96	4.88	7.04	5.04	5.10	4.86	6.20	4.56	4.62	4.50
2536	1, 1.5	2	+	40	0.16	17	23	.70	3.82	4.74	5.40	3.86	3.96	5.08	5.18	3.60	3.78	4.86
2537	1, 1.5	2	-	40	0.16	23	17	.70	6.10	5.06	8.62	6.32	6.52	5.18	8.32	6.12	6.30	5.00
2538	1, 1.5	2	+	40	0.33	13	27	.70	2.50	4.84	3.84	2.70	2.76	5.26	3.80	2.54	2.68	4.76
2539	1, 1.5	2	-	40	0.33	27	13	.70	7.42	5.62	10.64	8.18	8.38	5.94	10.52	7.76	8.00	5.56
2540	1, 1.5	2	+	40	0.50	10	30	.70	1.96	5.06	2.82	2.08	2.12	5.94	2.74	1.76	1.88	5.48
2541	1, 1.5	2	-	40	0.50	30	10	.70	9.34	6.84	12.34	9.76	10.00	6.34	12.80	10.54	10.70	6.70
2542	1, 1.5	2	0	60	0	30	30	.70	4.36	4.44	7.06	5.12	5.26	5.38	7.34	5.38	5.46	5.36
2543	1, 1.5	2	+	60	0.16	25	35	.70	3.50	4.42	5.40	3.84	3.92	5.12	5.46	3.96	4.02	5.16
2544	1, 1.5	2	-	60	0.16	35	25	.70	6.32	4.78	8.78	6.72	6.76	5.16	8.58	6.42	6.54	4.72
2545	1, 1.5	2	+	60	0.33	20	40	.70	3.04	5.08	4.28	2.94	3.00	5.16	3.90	2.88	2.98	4.82
2546	1, 1.5	2	-	60	0.33	40	20	.70	7.86	5.42	10.72	8.20	8.30	5.66	10.88	8.52	8.60	5.22
2547	1, 1.5	2	+	60	0.50	15	45	.70	2.22	5.36	3.08	2.14	2.16	5.40	2.68	1.72	1.76	4.98
2548	1, 1.5	2	-	60	0.50	45	15	.70	9.50	6.22	12.86	10.52	10.66	5.94	13.54	10.78	10.88	5.92
2549	1, 1.5	2	0	80	0	40	40	.70	4.40	4.24	7.24	5.28	5.32	5.36	6.34	4.66	4.68	4.82
2550	1, 1.5	2	+	80	0.16	34	46	.70	3.50	4.54	6.02	4.30	4.36	5.64	5.34	3.70	3.78	5.22
2551	1, 1.5	2	-	80	0.16	46	34	.70	6.12	4.92	8.90	6.74	6.76	5.48	8.10	6.30	6.38	5.12
2552	1, 1.5	2	+	80	0.33	27	53	.70	2.62	5.02	4.38	3.16	3.20	5.56	3.68	2.48	2.56	4.94
2553	1, 1.5	2	-	80	0.33	53	27	.70	7.84	5.18	11.18	8.82	8.88	5.66	10.32	8.28	8.30	5.24
2554	1, 1.5	2	+	80	0.50	20	60	.70	2.20	5.72	3.46	2.28	2.32	6.08	2.54	1.58	1.60	5.20
2555	1, 1.5	2	-	80	0.50	60	20	.70	9.52	5.84	13.86	11.08	11.26	6.38	13.04	10.24	10.36	6.00
2556	1, 1.5	2	0	100	0	50	50	.70	5.10	4.98	6.98	5.58	5.60	5.54	6.56	4.96	5.04	4.80
2557	1, 1.5	2	+	100	0.16	42	58	.70	3.64	4.78	5.98	4.44	4.46	5.60	4.80	3.46	3.52	4.58
2558	1, 1.5	2	-	100	0.16	58	42	.70	6.22	5.54	8.82	6.68	6.76	5.24	8.40	6.14	6.14	4.86

	D	VR	P	N	Δn	n1	n2	ε	F g	B-F g	F t	F-GG t	F-HF t	B-F t	F g x t	F-GG g x t	F-HF g x t	B-F g x t
2559	1, 1.5	2	+	100	0.33	33	67	.70	2.66	5.18	4.54	3.42	3.44	5.84	3.72	2.70	2.72	4.76
2560	1, 1.5	2	-	100	0.33	67	33	.70	8.12	5.74	10.80	8.44	8.54	5.42	11.36	8.46	8.54	5.32
2561	1, 1.5	2	+	100	0.50	25	75	.70	1.94	4.84	3.38	2.06	2.14	5.96	2.16	1.42	1.42	4.52
2562	1, 1.5	2	-	100	0.50	75	25	.70	10.32	6.08	13.38	10.52	10.58	5.74	12.88	10.54	10.60	5.58
2563	1, 1.5	2	0	150	0	75	75	.70	5.68	5.58	6.58	4.96	4.98	5.00	7.24	5.20	5.24	5.38
2564	1, 1.5	2	+	150	0.16	63	87	.70	4.28	5.56	5.42	4.02	4.06	5.36	5.08	3.48	3.48	4.88
2565	1, 1.5	2	-	150	0.16	87	63	.70	6.86	5.54	8.24	6.26	6.30	5.14	8.78	6.74	6.78	5.28
2566	1, 1.5	2	+	150	0.33	50	100	.70	2.92	4.78	4.20	2.96	2.98	5.52	4.26	2.92	2.94	5.46
2567	1, 1.5	2	-	150	0.33	100	50	.70	7.76	5.14	10.36	8.18	8.26	5.06	10.36	8.28	8.30	4.70
2568	1, 1.5	2	+	150	0.50	37	113	.70	1.94	5.42	3.04	2.14	2.14	5.48	3.08	2.02	2.02	5.62
2569	1, 1.5	2	-	150	0.50	113	37	.70	9.80	5.56	12.70	10.08	10.12	5.08	12.54	9.92	9.96	5.10
2570	1, 1.5	2	0	200	0	100	100	.70	5.20	5.24	6.80	5.18	5.20	5.08	6.30	5.04	5.08	5.10
2571	1, 1.5	2	+	200	0.16	84	116	.70	4.00	5.22	5.28	3.82	3.84	5.14	5.64	3.94	3.96	5.42
2572	1, 1.5	2	-	200	0.16	116	84	.70	6.56	5.20	8.48	6.54	6.56	5.30	8.64	6.38	6.40	5.30
2573	1, 1.5	2	+	200	0.33	67	133	.70	3.08	5.04	4.02	2.98	2.98	5.24	4.00	2.74	2.74	5.24
2574	1, 1.5	2	-	200	0.33	133	67	.70	8.04	5.30	10.46	8.02	8.02	4.80	10.68	8.34	8.34	5.46
2575	1, 1.5	2	+	200	0.50	50	150	.70	2.10	5.54	2.80	2.06	2.06	5.24	3.12	2.22	2.22	5.50
2576	1, 1.5	2	-	200	0.50	150	50	.70	10.36	5.58	12.62	10.32	10.38	5.46	12.98	10.50	10.54	5.26
2577	1, 1.5	5	0	20	0	10	10	.70	5.58	4.58	7.30	5.30	5.64	4.50	7.64	5.54	5.82	4.72
2578	1, 1.5	5	+	20	0.16	8	12	.70	3.60	4.96	3.72	2.54	2.92	4.64	3.50	2.46	2.70	4.18
2579	1, 1.5	5	-	20	0.16	12	8	.70	9.76	6.08	11.84	9.30	9.70	4.90	11.96	9.22	9.48	4.96
2580	1, 1.5	5	+	20	0.33	7	13	.70	2.70	4.66	2.66	1.62	1.80	4.56	2.42	1.68	1.74	4.24
2581	1, 1.5	5	-	20	0.33	13	7	.70	12.24	6.72	15.10	11.84	12.30	5.50	15.02	11.90	12.44	5.46
2582	1, 1.5	5	+	20	0.50	5	15	.70	1.20	4.88	.90	.60	.64	4.72	.92	.62	.68	4.70
2583	1, 1.5	5	-	20	0.50	15	5	.70	18.64	9.46	22.90	18.90	19.58	7.46	23.00	18.70	19.54	7.74
2584	1, 1.5	5	0	40	0	20	20	.70	5.46	4.94	6.86	5.10	5.24	4.94	6.32	4.84	4.98	4.64
2585	1, 1.5	5	+	40	0.16	17	23	.70	3.22	4.44	4.20	3.18	3.26	5.18	4.14	2.92	3.00	5.02
2586	1, 1.5	5	-	40	0.16	23	17	.70	7.86	5.02	10.58	7.96	8.18	5.16	10.98	8.26	8.50	5.34
2587	1, 1.5	5	+	40	0.33	13	27	.70	1.36	4.32	1.96	1.34	1.42	4.86	2.06	1.32	1.36	4.68
2588	1, 1.5	5	-	40	0.33	27	13	.70	12.06	5.82	16.22	13.00	13.28	5.58	16.54	13.18	13.36	4.98
2589	1, 1.5	5	+	40	0.50	10	30	.70	.86	4.48	1.10	.60	.64	5.06	.88	.54	.58	5.04
2590	1, 1.5	5	-	40	0.50	30	10	.70	16.62	6.88	22.10	18.44	18.80	6.40	22.16	18.78	19.02	6.42

	D	VR	P	N	Δn	n1	n2	ε	F g	B-F g	F t	F-GG t	F-HF t	B-F t	F g x t	F-GG g x t	F-HF g x t	B-F g x t
2591	1, 1.5	5	0	60	0	30	30	.70	4.42	4.18	7.08	5.36	5.36	4.98	7.22	5.26	5.40	5.04
2592	1, 1.5	5	+	60	0.16	25	35	.70	3.14	4.42	4.14	2.86	2.96	5.22	4.16	2.88	2.92	5.04
2593	1, 1.5	5	-	60	0.16	35	25	.70	8.26	4.82	10.64	8.20	8.30	5.02	10.56	8.38	8.46	4.70
2594	1, 1.5	5	+	60	0.33	20	40	.70	1.66	4.82	2.14	1.54	1.60	5.30	1.88	1.24	1.28	4.72
2595	1, 1.5	5	-	60	0.33	40	20	.70	11.90	5.72	15.98	13.04	13.20	5.82	16.08	13.32	13.48	5.20
2596	1, 1.5	5	+	60	0.50	15	45	.70	.74	4.74	1.00	.62	.64	5.24	.84	.46	.48	4.76
2597	1, 1.5	5	-	60	0.50	45	15	.70	17.34	6.30	22.38	18.74	18.92	5.72	22.26	18.82	19.02	5.86
2598	1, 1.5	5	0	80	0	40	40	.70	4.84	4.72	7.36	5.46	5.54	5.20	6.50	4.88	4.92	4.58
2599	1, 1.5	5	+	80	0.16	34	46	.70	2.80	4.40	4.42	3.20	3.28	5.26	4.18	2.74	2.80	4.92
2600	1, 1.5	5	-	80	0.16	46	34	.70	7.76	5.06	10.68	8.56	8.62	5.34	10.20	7.68	7.84	4.98
2601	1, 1.5	5	+	80	0.33	27	53	.70	1.40	4.60	2.46	1.56	1.56	5.74	1.62	1.04	1.08	4.78
2602	1, 1.5	5	-	80	0.33	53	27	.70	12.18	5.36	16.42	13.20	13.32	5.34	15.56	12.52	12.64	5.26
2603	1, 1.5	5	+	80	0.50	20	60	.70	.86	5.44	1.12	.68	.70	6.00	.52	.24	.24	4.96
2604	1, 1.5	5	-	80	0.50	60	20	.70	16.06	5.64	23.46	19.70	19.82	6.18	22.38	18.92	18.94	5.78
2605	1, 1.5	5	0	100	0	50	50	.70	4.96	4.84	6.72	5.02	5.06	5.02	6.62	4.70	4.74	4.82
2606	1, 1.5	5	+	100	0.16	42	58	.70	3.02	4.76	4.40	3.00	3.04	5.36	3.84	2.60	2.62	4.66
2607	1, 1.5	5	-	100	0.16	58	42	.70	8.04	5.44	11.00	8.72	8.82	5.36	10.28	7.92	7.98	5.04
2608	1, 1.5	5	+	100	0.33	33	67	.70	1.28	4.62	2.14	1.34	1.34	5.58	1.90	1.10	1.10	4.90
2609	1, 1.5	5	-	100	0.33	67	33	.70	12.04	5.68	16.20	13.26	13.30	5.26	16.34	13.34	13.40	5.52
2610	1, 1.5	5	+	100	0.50	25	75	.70	.72	4.36	.68	.38	.40	5.98	.56	.30	.30	4.30
2611	1, 1.5	5	-	100	0.50	75	25	.70	16.90	6.12	21.36	18.30	18.40	5.84	21.82	18.18	18.34	5.58
2612	1, 1.5	5	0	150	0	75	75	.70	5.66	5.86	6.72	5.12	5.12	4.90	6.94	5.14	5.22	5.28
2613	1, 1.5	5	+	150	0.16	63	87	.70	3.56	5.36	3.98	2.86	2.86	4.86	3.84	2.76	2.76	5.04
2614	1, 1.5	5	-	150	0.16	87	63	.70	8.50	5.46	10.12	8.08	8.12	5.08	10.72	8.26	8.30	5.38
2615	1, 1.5	5	+	150	0.33	50	100	.70	1.42	5.04	2.06	1.30	1.30	5.24	2.04	1.32	1.32	5.36
2616	1, 1.5	5	-	150	0.33	100	50	.70	11.48	5.22	15.44	12.28	12.34	5.12	15.14	12.04	12.08	4.90
2617	1, 1.5	5	+	150	0.50	37	113	.70	.62	4.98	.90	.50	.50	5.50	.86	.54	.54	5.44
2618	1, 1.5	5	-	150	0.50	113	37	.70	17.00	5.62	21.90	18.64	18.66	5.00	21.70	18.06	18.12	4.68
2619	1, 1.5	5	0	200	0	100	100	.70	5.34	5.38	7.02	5.10	5.14	5.26	6.34	4.74	4.80	4.92
2620	1, 1.5	5	+	200	0.16	84	116	.70	3.08	5.16	3.92	2.96	2.96	5.14	4.02	3.04	3.04	5.12
2621	1, 1.5	5	-	200	0.16	116	84	.70	7.98	5.12	10.00	7.98	7.98	5.24	10.52	8.46	8.48	5.40
2622	1, 1.5	5	+	200	0.33	67	133	.70	1.80	5.00	2.10	1.40	1.40	5.26	2.00	1.32	1.32	5.18

	D	VR	P	N	Δn	n1	n2	ε	F g	B-F g	F t	F-GG t	F-HF t	B-F t	F g x t	F-GG g x t	F-HF g x t	B-F g x t
2623	1, 1.5	5	-	200	0.33	133	67	.70	12.36	5.42	15.46	12.26	12.28	4.82	15.34	12.58	12.60	5.50
2624	1, 1.5	5	+	200	0.50	50	150	.70	.58	5.12	.90	.58	.58	5.02	.86	.42	.44	5.62
2625	1, 1.5	5	-	200	0.50	150	50	.70	17.42	5.54	21.84	17.96	17.98	5.60	22.08	18.80	18.84	5.20
2626	1, 1.5	1	0	20	0	10	10	.80	4.20	3.80	5.88	4.58	4.92	4.42	6.36	4.92	5.26	4.66
2627	1, 1.5	1	0	20	0.16	8	12	.80	4.84	4.38	5.78	4.32	4.66	4.30	5.68	4.18	4.54	4.14
2628	1, 1.5	1	0	20	0.33	7	13	.80	5.04	4.70	5.78	4.60	4.86	4.42	6.00	4.82	5.08	4.54
2629	1, 1.5	1	0	20	0.50	5	15	.80	4.56	6.90	5.82	4.70	4.92	6.46	6.00	4.94	5.22	6.54
2630	1, 1.5	1	0	40	0	20	20	.80	4.98	4.80	5.76	4.56	4.66	4.52	6.06	4.80	5.02	4.78
2631	1, 1.5	1	0	40	0.16	17	23	.80	5.12	5.26	5.52	4.60	4.70	4.86	5.86	4.66	4.78	4.90
2632	1, 1.5	1	0	40	0.33	13	27	.80	4.50	5.14	5.84	4.62	4.90	4.66	6.52	5.20	5.44	5.42
2633	1, 1.5	1	0	40	0.50	10	30	.80	4.26	5.64	5.80	4.46	4.64	5.42	6.40	5.08	5.30	7.02
2634	1, 1.5	1	0	60	0	30	30	.80	5.18	5.14	5.92	4.76	4.96	4.84	5.66	4.64	4.80	4.68
2635	1, 1.5	1	0	60	0.16	25	35	.80	5.04	4.86	6.00	4.92	5.06	4.92	5.72	4.80	4.88	4.68
2636	1, 1.5	1	0	60	0.33	20	40	.80	5.06	5.44	6.02	4.92	5.06	5.06	6.58	5.48	5.58	5.82
2637	1, 1.5	1	0	60	0.50	15	45	.80	5.12	6.16	5.86	4.56	4.68	5.18	5.90	4.86	4.94	5.62
2638	1, 1.5	1	0	80	0	40	40	.80	5.12	4.90	6.58	5.14	5.22	5.18	5.58	4.48	4.56	4.54
2639	1, 1.5	1	0	80	0.16	34	46	.80	4.90	5.16	6.24	5.08	5.16	5.18	6.02	4.96	4.98	5.12
2640	1, 1.5	1	0	80	0.33	27	53	.80	5.28	5.44	5.80	4.74	4.84	5.16	5.50	4.30	4.34	4.74
2641	1, 1.5	1	0	80	0.50	20	60	.80	5.06	6.04	6.24	5.04	5.12	5.56	5.96	4.78	4.86	5.70
2642	1, 1.5	1	0	100	0	50	50	.80	5.48	5.40	5.78	4.62	4.68	4.62	5.84	4.84	4.86	4.74
2643	1, 1.5	1	0	100	0.16	42	58	.80	4.98	5.16	5.90	4.78	4.82	4.82	5.60	4.56	4.56	4.70
2644	1, 1.5	1	0	100	0.33	33	67	.80	5.26	5.42	5.50	4.50	4.62	4.72	5.70	4.64	4.70	4.82
2645	1, 1.5	1	0	100	0.50	25	75	.80	4.86	5.64	5.66	4.62	4.66	5.02	5.38	4.38	4.48	4.56
2646	1, 1.5	1	0	150	0	75	75	.80	5.38	5.44	6.48	5.40	5.46	5.32	6.62	5.52	5.58	5.86
2647	1, 1.5	1	0	150	0.16	63	87	.80	5.58	5.66	6.40	5.14	5.16	5.60	6.10	4.96	4.96	5.24
2648	1, 1.5	1	0	150	0.33	50	100	.80	5.20	5.56	6.36	5.14	5.18	5.38	5.78	4.70	4.74	5.02
2649	1, 1.5	1	0	150	0.50	37	113	.80	5.26	5.74	5.86	4.68	4.74	5.24	5.80	4.52	4.52	5.02
2650	1, 1.5	1	0	200	0	100	100	.80	5.02	5.18	6.48	5.08	5.16	5.40	6.42	5.34	5.38	5.66
2651	1, 1.5	1	0	200	0.16	84	116	.80	5.22	5.26	6.40	5.08	5.08	5.58	6.40	5.26	5.30	5.50
2652	1, 1.5	1	0	200	0.33	67	133	.80	5.22	5.42	6.38	5.16	5.20	5.30	6.26	5.42	5.48	5.36
2653	1, 1.5	1	0	200	0.50	50	150	.80	5.36	5.64	5.98	4.96	5.04	5.26	5.84	4.66	4.66	4.90
2654	1, 1.5	1.5	0	20	0	10	10	.80	4.52	3.96	6.06	4.72	5.10	4.48	6.24	4.72	5.22	4.54

	D	VR	P	N	Δn	n1	n2	ε	F g	B-F g	F t	F-GG t	F-HF t	B-F t	F g x t	F-GG g x t	F-HF g x t	B-F g x t
2655	1, 1.5	1.5	+	20	0.16	8	12	.80	4.28	4.28	4.84	3.68	4.12	4.18	4.52	3.58	3.96	4.02
2656	1, 1.5	1.5	-	20	0.16	12	8	.80	5.74	4.80	7.30	6.04	6.44	4.80	7.42	5.92	6.40	4.78
2657	1, 1.5	1.5	+	20	0.33	7	13	.80	3.88	4.52	4.20	3.14	3.48	4.04	4.38	3.56	3.78	4.18
2658	1, 1.5	1.5	-	20	0.33	13	7	.80	5.98	5.52	7.60	6.30	6.60	4.96	8.28	6.62	7.10	5.32
2659	1, 1.5	1.5	+	20	0.50	5	15	.80	2.94	5.92	3.82	2.84	3.12	5.90	3.96	3.04	3.28	5.88
2660	1, 1.5	1.5	-	20	0.50	15	5	.80	7.98	8.52	9.58	7.68	8.14	6.78	9.36	7.84	8.10	6.70
2661	1, 1.5	1.5	0	40	0	20	20	.80	4.94	4.80	5.80	4.72	4.96	4.46	6.12	4.68	4.94	4.72
2662	1, 1.5	1.5	+	40	0.16	17	23	.80	4.40	5.06	5.00	3.90	4.12	4.68	4.98	4.10	4.26	4.80
2663	1, 1.5	1.5	-	40	0.16	23	17	.80	5.78	5.26	6.66	5.30	5.50	4.80	6.62	5.48	5.58	4.86
2664	1, 1.5	1.5	+	40	0.33	13	27	.80	3.04	4.80	3.98	3.02	3.08	4.64	4.56	3.52	3.74	5.36
2665	1, 1.5	1.5	-	40	0.33	27	13	.80	6.76	5.64	8.10	6.58	6.76	5.36	7.22	6.02	6.16	4.70
2666	1, 1.5	1.5	+	40	0.50	10	30	.80	2.56	5.32	3.22	2.46	2.48	5.40	3.94	3.06	3.14	6.66
2667	1, 1.5	1.5	-	40	0.50	30	10	.80	7.52	6.56	8.94	7.46	7.74	5.74	8.66	7.50	7.68	5.56
2668	1, 1.5	1.5	0	60	0	30	30	.80	5.10	5.02	6.18	4.86	4.96	4.88	5.90	4.64	4.72	4.66
2669	1, 1.5	1.5	+	60	0.16	25	35	.80	4.44	4.64	5.22	4.22	4.36	5.08	4.86	3.88	3.96	4.66
2670	1, 1.5	1.5	-	60	0.16	35	25	.80	5.48	4.58	6.98	6.00	6.14	5.10	6.90	5.60	5.78	4.62
2671	1, 1.5	1.5	+	60	0.33	20	40	.80	3.76	5.44	4.54	3.60	3.62	5.22	4.86	3.86	3.96	5.50
2672	1, 1.5	1.5	-	60	0.33	40	20	.80	6.04	4.92	8.10	6.82	6.94	5.10	7.18	5.84	5.92	4.64
2673	1, 1.5	1.5	+	60	0.50	15	45	.80	3.16	5.66	3.44	2.50	2.60	4.90	3.64	2.90	3.00	5.34
2674	1, 1.5	1.5	-	60	0.50	45	15	.80	7.60	5.78	9.32	7.90	8.02	5.40	8.80	7.18	7.24	5.36
2675	1, 1.5	1.5	0	80	0	40	40	.80	4.98	4.86	6.54	5.32	5.36	5.50	5.56	4.56	4.66	4.74
2676	1, 1.5	1.5	+	80	0.16	34	46	.80	3.92	4.72	5.50	4.34	4.40	5.44	5.12	4.34	4.48	5.48
2677	1, 1.5	1.5	-	80	0.16	46	34	.80	5.46	4.82	7.60	6.16	6.28	5.34	6.90	5.50	5.60	4.70
2678	1, 1.5	1.5	+	80	0.33	27	53	.80	3.94	5.14	4.32	3.56	3.60	5.02	3.90	3.12	3.22	4.84
2679	1, 1.5	1.5	-	80	0.33	53	27	.80	6.42	5.34	8.68	7.34	7.46	5.54	8.38	6.76	6.86	4.84
2680	1, 1.5	1.5	+	80	0.50	20	60	.80	3.14	5.76	3.66	2.90	3.02	5.58	3.68	2.84	2.90	5.88
2681	1, 1.5	1.5	-	80	0.50	60	20	.80	7.46	5.70	10.18	8.54	8.58	5.88	9.12	7.82	7.88	5.54
2682	1, 1.5	1.5	0	100	0	50	50	.80	5.12	5.22	5.86	4.58	4.62	4.68	6.02	4.82	4.94	5.06
2683	1, 1.5	1.5	+	100	0.16	42	58	.80	4.42	4.88	4.94	3.86	3.98	4.70	4.72	3.78	3.84	4.76
2684	1, 1.5	1.5	-	100	0.16	58	42	.80	6.12	5.50	6.90	5.54	5.60	4.98	6.84	5.58	5.64	5.16
2685	1, 1.5	1.5	+	100	0.33	33	67	.80	3.72	5.26	4.28	3.38	3.50	4.66	4.24	3.28	3.34	4.90
2686	1, 1.5	1.5	-	100	0.33	67	33	.80	7.22	5.92	8.38	6.92	7.00	4.92	8.36	7.08	7.20	4.78

	D	VR	P	N	Δn	n1	n2	ε	F g	B-F g	F t	F-GG t	F-HF t	B-F t	F g x t	F-GG g x t	F-HF g x t	B-F g x t
2687	1, 1.5	1.5	+	100	0.50	25	75	.80	3.10	5.36	3.46	2.62	2.72	4.98	3.16	2.36	2.46	4.82
2688	1, 1.5	1.5	-	100	0.50	75	25	.80	8.26	5.92	8.92	7.34	7.40	5.04	9.48	8.04	8.12	5.24
2689	1, 1.5	1.5	0	150	0	75	75	.80	5.28	5.40	6.64	5.34	5.46	5.36	6.84	5.46	5.58	5.76
2690	1, 1.5	1.5	+	150	0.16	63	87	.80	4.84	5.62	5.42	4.42	4.44	5.38	5.32	4.30	4.38	5.24
2691	1, 1.5	1.5	-	150	0.16	87	63	.80	6.06	5.02	7.68	6.22	6.34	5.44	7.10	6.02	6.06	5.48
2692	1, 1.5	1.5	+	150	0.33	50	100	.80	3.82	5.22	4.36	3.40	3.42	5.22	4.18	3.52	3.58	5.04
2693	1, 1.5	1.5	-	150	0.33	100	50	.80	6.34	5.08	8.80	7.48	7.52	5.80	8.34	6.94	6.98	5.20
2694	1, 1.5	1.5	+	150	0.50	37	113	.80	3.14	5.54	3.62	2.96	2.98	5.20	3.14	2.24	2.30	4.90
2695	1, 1.5	1.5	-	150	0.50	113	37	.80	7.62	5.48	9.88	8.34	8.40	5.30	9.86	8.24	8.24	5.20
2696	1, 1.5	1.5	0	200	0	100	100	.80	5.18	5.40	6.44	5.22	5.28	5.32	6.50	5.46	5.46	5.74
2697	1, 1.5	1.5	+	200	0.16	84	116	.80	4.38	5.46	5.24	4.36	4.36	5.24	5.60	4.50	4.52	5.38
2698	1, 1.5	1.5	-	200	0.16	116	84	.80	5.84	5.26	7.40	6.18	6.24	5.50	7.32	6.10	6.14	5.46
2699	1, 1.5	1.5	+	200	0.33	67	133	.80	3.58	5.20	4.28	3.66	3.68	5.38	4.60	3.76	3.76	5.46
2700	1, 1.5	1.5	-	200	0.33	133	67	.80	6.54	5.16	8.54	7.28	7.36	5.76	8.60	7.02	7.02	5.38
2701	1, 1.5	1.5	+	200	0.50	50	150	.80	3.16	5.60	3.78	2.82	2.84	5.34	3.44	2.76	2.78	5.00
2702	1, 1.5	1.5	-	200	0.50	150	50	.80	7.80	5.58	10.22	8.52	8.56	5.66	9.62	8.24	8.26	5.18
2703	1, 1.5	2	0	20	0	10	10	.80	4.72	4.10	6.16	4.82	5.08	4.66	6.22	5.02	5.30	4.62
2704	1, 1.5	2	+	20	0.16	8	12	.80	3.88	4.30	4.46	3.38	3.64	4.08	4.20	3.16	3.44	3.94
2705	1, 1.5	2	-	20	0.16	12	8	.80	6.52	5.06	8.22	6.68	7.02	4.76	8.22	6.46	6.90	5.02
2706	1, 1.5	2	+	20	0.33	7	13	.80	3.34	4.38	3.36	2.58	2.68	3.96	3.72	2.88	3.10	3.96
2707	1, 1.5	2	-	20	0.33	13	7	.80	7.20	5.66	9.10	7.28	7.64	5.10	9.66	7.98	8.46	5.06
2708	1, 1.5	2	+	20	0.50	5	15	.80	2.42	5.30	2.46	1.78	2.02	5.48	2.72	2.02	2.20	5.76
2709	1, 1.5	2	-	20	0.50	15	5	.80	10.16	9.08	12.08	10.18	10.62	6.90	12.18	10.34	10.72	6.74
2710	1, 1.5	2	0	40	0	20	20	.80	5.10	4.80	5.84	4.58	4.84	4.46	5.88	4.84	5.00	4.66
2711	1, 1.5	2	+	40	0.16	17	23	.80	4.08	5.00	4.26	3.22	3.40	4.72	4.52	3.60	3.74	4.82
2712	1, 1.5	2	-	40	0.16	23	17	.80	6.36	5.42	7.50	5.90	6.14	4.92	7.50	6.10	6.20	4.84
2713	1, 1.5	2	+	40	0.33	13	27	.80	2.24	4.48	3.06	2.20	2.42	4.58	3.72	2.76	2.82	5.24
2714	1, 1.5	2	-	40	0.33	27	13	.80	8.16	5.82	9.82	8.06	8.44	5.52	8.78	7.28	7.50	4.70
2715	1, 1.5	2	+	40	0.50	10	30	.80	1.78	4.82	2.06	1.50	1.56	5.36	2.68	2.14	2.24	6.16
2716	1, 1.5	2	-	40	0.50	30	10	.80	9.78	6.98	11.70	9.80	10.10	6.12	11.34	9.66	9.96	5.78
2717	1, 1.5	2	0	60	0	30	30	.80	5.22	5.04	6.22	5.04	5.24	4.98	5.72	4.72	4.82	4.72
2718	1, 1.5	2	+	60	0.16	25	35	.80	3.94	4.60	4.58	3.68	3.80	4.82	4.48	3.38	3.48	4.60

	D	VR	P	N	Δn	n1	n2	ε	F g	B-F g	F t	F-GG t	F-HF t	B-F t	F g x t	F-GG g x t	F-HF g x t	B-F g x t
2719	1, 1.5	2	-	60	0.16	35	25	.80	6.14	4.66	7.82	6.64	6.72	5.52	7.96	6.38	6.56	5.22
2720	1, 1.5	2	+	60	0.33	20	40	.80	2.96	5.28	3.62	2.62	2.68	4.88	3.68	2.80	2.86	5.38
2721	1, 1.5	2	-	60	0.33	40	20	.80	7.42	5.26	9.92	8.22	8.40	5.28	9.10	7.42	7.60	4.80
2722	1, 1.5	2	+	60	0.50	15	45	.80	2.18	5.54	2.18	1.74	1.84	5.18	2.60	2.00	2.02	5.26
2723	1, 1.5	2	-	60	0.50	45	15	.80	9.76	6.18	12.44	10.34	10.50	5.84	11.78	9.66	9.84	5.54
2724	1, 1.5	2	0	80	0	40	40	.80	4.98	4.86	6.62	5.20	5.44	5.44	5.62	4.42	4.52	4.70
2725	1, 1.5	2	+	80	0.16	34	46	.80	3.46	4.52	5.18	4.00	4.00	5.88	4.88	4.02	4.06	5.44
2726	1, 1.5	2	-	80	0.16	46	34	.80	5.94	4.94	8.20	6.86	6.96	5.48	7.58	6.18	6.20	4.66
2727	1, 1.5	2	+	80	0.33	27	53	.80	2.86	4.88	3.52	2.72	2.76	4.96	3.06	2.50	2.54	4.78
2728	1, 1.5	2	-	80	0.33	53	27	.80	7.84	5.32	10.34	8.78	8.84	5.54	10.44	8.26	8.36	5.24
2729	1, 1.5	2	+	80	0.50	20	60	.80	2.26	5.64	2.38	1.78	1.84	5.66	2.38	1.92	1.96	6.00
2730	1, 1.5	2	-	80	0.50	60	20	.80	9.56	5.84	12.80	11.18	11.30	5.86	12.18	10.20	10.42	5.42
2731	1, 1.5	2	0	100	0	50	50	.80	4.94	5.08	6.00	4.78	4.84	4.90	6.04	4.94	5.00	5.10
2732	1, 1.5	2	+	100	0.16	42	58	.80	4.04	4.86	4.32	3.44	3.50	4.66	4.20	3.50	3.56	4.48
2733	1, 1.5	2	-	100	0.16	58	42	.80	6.64	5.32	7.52	6.18	6.24	4.82	7.44	6.08	6.10	5.06
2734	1, 1.5	2	+	100	0.33	33	67	.80	2.72	4.96	3.38	2.40	2.44	4.78	3.22	2.28	2.38	5.12
2735	1, 1.5	2	-	100	0.33	67	33	.80	8.64	5.96	10.40	8.70	8.78	4.80	10.52	8.46	8.52	4.68
2736	1, 1.5	2	+	100	0.50	25	75	.80	2.06	5.24	2.36	1.80	1.82	4.84	1.92	1.50	1.52	4.82
2737	1, 1.5	2	-	100	0.50	75	25	.80	10.60	6.16	11.94	9.96	10.14	5.12	12.30	10.62	10.72	5.20
2738	1, 1.5	2	0	150	0	75	75	.80	5.30	5.22	6.44	5.32	5.36	5.48	6.76	5.62	5.62	5.66
2739	1, 1.5	2	+	150	0.16	63	87	.80	4.28	5.48	4.98	4.12	4.16	5.60	4.94	3.90	3.96	5.44
2740	1, 1.5	2	-	150	0.16	87	63	.80	6.50	5.16	8.28	6.80	6.84	5.54	7.86	6.68	6.74	5.32
2741	1, 1.5	2	+	150	0.33	50	100	.80	3.08	5.22	3.42	2.60	2.68	5.62	3.42	2.64	2.66	5.18
2742	1, 1.5	2	-	150	0.33	100	50	.80	7.54	4.90	10.26	8.80	8.88	5.40	9.88	8.46	8.54	5.16
2743	1, 1.5	2	+	150	0.50	37	113	.80	2.06	5.44	2.56	2.00	2.00	5.34	1.98	1.66	1.66	4.84
2744	1, 1.5	2	-	150	0.50	113	37	.80	9.94	5.92	12.52	11.02	11.04	5.38	12.56	10.72	10.82	5.28
2745	1, 1.5	2	0	200	0	100	100	.80	5.20	5.22	6.22	5.18	5.22	5.32	6.56	5.44	5.50	5.56
2746	1, 1.5	2	+	200	0.16	84	116	.80	3.90	5.32	4.74	3.86	3.88	5.34	5.00	4.08	4.14	5.56
2747	1, 1.5	2	-	200	0.16	116	84	.80	6.40	5.08	8.18	6.88	6.88	5.38	8.08	6.78	6.82	5.58
2748	1, 1.5	2	+	200	0.33	67	133	.80	2.80	5.10	3.48	2.84	2.84	5.36	3.44	2.82	2.84	5.24
2749	1, 1.5	2	-	200	0.33	133	67	.80	7.78	5.20	10.26	8.80	8.80	5.62	10.54	8.88	8.92	5.44
2750	1, 1.5	2	+	200	0.50	50	150	.80	2.18	5.54	2.28	2.00	2.00	5.26	2.32	1.80	1.80	5.08

	D	VR	P	N	Δn	n1	n2	ε	F g	B-F g	F t	F-GG t	F-HF t	B-F t	F g x t	F-GG g x t	F-HF g x t	B-F g x t
2751	1, 1.5	2	-	200	0.50	150	50	.80	10.00	5.38	12.90	11.22	11.26	5.76	12.80	10.84	10.88	5.46
2752	1, 1.5	5	0	20	0	10	10	.80	5.50	4.68	6.60	5.28	5.56	4.68	6.88	5.18	5.56	4.36
2753	1, 1.5	5	+	20	0.16	8	12	.80	3.30	4.80	3.22	2.38	2.60	4.34	3.20	2.38	2.60	4.10
2754	1, 1.5	5	-	20	0.16	12	8	.80	9.62	5.80	11.70	9.54	9.94	4.76	11.40	9.10	9.74	4.72
2755	1, 1.5	5	+	20	0.33	7	13	.80	2.58	4.84	2.00	1.62	1.74	4.28	2.14	1.70	1.84	4.10
2756	1, 1.5	5	-	20	0.33	13	7	.80	12.06	6.56	14.92	11.76	12.56	5.20	15.02	12.80	13.18	4.84
2757	1, 1.5	5	+	20	0.50	5	15	.80	1.18	4.48	.84	.56	.68	4.52	.72	.50	.58	4.50
2758	1, 1.5	5	-	20	0.50	15	5	.80	18.26	9.96	22.70	19.56	20.16	7.08	22.40	18.86	19.64	6.94
2759	1, 1.5	5	0	40	0	20	20	.80	5.38	4.74	5.94	4.78	4.90	4.32	6.00	4.56	4.72	4.34
2760	1, 1.5	5	+	40	0.16	17	23	.80	3.74	5.00	3.34	2.58	2.66	4.18	3.44	2.58	2.68	4.26
2761	1, 1.5	5	-	40	0.16	23	17	.80	7.94	5.26	9.64	8.18	8.42	5.18	9.56	7.94	8.20	4.66
2762	1, 1.5	5	+	40	0.33	13	27	.80	1.60	4.30	1.28	.92	1.00	4.26	1.90	1.36	1.40	4.66
2763	1, 1.5	5	-	40	0.33	27	13	.80	12.62	5.78	15.24	13.18	13.44	5.76	14.92	12.82	12.98	4.94
2764	1, 1.5	5	+	40	0.50	10	30	.80	.94	4.58	.48	.26	.30	4.80	.72	.54	.54	5.56
2765	1, 1.5	5	-	40	0.50	30	10	.80	16.92	7.12	21.74	19.40	19.82	6.04	21.38	18.82	19.08	5.48
2766	1, 1.5	5	0	60	0	30	30	.80	5.22	4.88	6.78	5.54	5.64	5.22	5.72	4.96	5.02	4.72
2767	1, 1.5	5	+	60	0.16	25	35	.80	3.00	4.58	3.40	2.68	2.72	4.64	3.26	2.60	2.64	4.84
2768	1, 1.5	5	-	60	0.16	35	25	.80	7.82	4.92	10.46	8.46	8.60	5.34	10.58	8.66	8.96	5.10
2769	1, 1.5	5	+	60	0.33	20	40	.80	1.56	4.96	1.80	1.36	1.40	4.68	1.74	1.32	1.36	4.92
2770	1, 1.5	5	-	60	0.33	40	20	.80	11.94	5.22	15.72	13.42	13.92	5.02	15.00	12.54	12.70	4.92
2771	1, 1.5	5	+	60	0.50	15	45	.80	.84	5.20	.66	.40	.42	5.04	.86	.70	.70	5.18
2772	1, 1.5	5	-	60	0.50	45	15	.80	16.62	6.12	22.58	19.96	20.18	5.50	21.82	18.92	19.36	5.32
2773	1, 1.5	5	0	80	0	40	40	.80	5.06	4.82	6.70	5.36	5.42	5.40	6.02	4.78	4.88	4.86
2774	1, 1.5	5	+	80	0.16	34	46	.80	2.86	4.18	3.88	3.20	3.22	5.86	3.90	3.12	3.22	5.38
2775	1, 1.5	5	-	80	0.16	46	34	.80	7.78	4.86	10.30	8.82	8.88	5.34	9.44	8.08	8.24	4.94
2776	1, 1.5	5	+	80	0.33	27	53	.80	1.54	4.52	1.78	1.28	1.30	5.30	1.68	1.28	1.32	4.68
2777	1, 1.5	5	-	80	0.33	53	27	.80	11.40	5.12	14.86	13.18	13.32	5.64	15.80	13.84	14.04	5.06
2778	1, 1.5	5	+	80	0.50	20	60	.80	.86	5.02	.74	.50	.50	5.48	.56	.34	.38	5.52
2779	1, 1.5	5	-	80	0.50	60	20	.80	16.38	5.80	22.80	20.08	20.26	5.82	22.08	19.72	19.94	5.28
2780	1, 1.5	5	0	100	0	50	50	.80	5.16	5.04	6.18	4.70	4.88	4.86	6.24	4.98	5.06	4.98
2781	1, 1.5	5	+	100	0.16	42	58	.80	3.00	4.64	3.38	2.64	2.68	4.82	3.50	2.92	2.94	4.68
2782	1, 1.5	5	-	100	0.16	58	42	.80	8.28	5.24	9.86	8.18	8.24	4.82	9.82	8.28	8.34	4.84

	D	VR	P	N	Δn	n1	n2	ε	F g	B-F g	F t	F-GG t	F-HF t	B-F t	F g x t	F-GG g x t	F-HF g x t	B-F g x t
2783	1, 1.5	5	+	100	0.33	33	67	.80	1.30	4.64	1.56	1.20	1.22	4.64	1.50	1.16	1.16	5.00
2784	1, 1.5	5	-	100	0.33	67	33	.80	12.62	5.60	15.60	13.64	13.76	4.90	15.62	13.68	13.84	4.62
2785	1, 1.5	5	+	100	0.50	25	75	.80	.60	4.56	.66	.46	.50	4.94	.44	.38	.38	4.94
2786	1, 1.5	5	-	100	0.50	75	25	.80	17.88	5.72	21.72	19.32	19.36	4.94	21.32	19.10	19.14	5.02
2787	1, 1.5	5	0	150	0	75	75	.80	5.18	5.00	6.22	5.32	5.36	5.22	7.02	5.54	5.62	5.56
2788	1, 1.5	5	+	150	0.16	63	87	.80	3.36	5.24	3.84	2.80	2.80	5.84	3.56	2.78	2.78	5.56
2789	1, 1.5	5	-	150	0.16	87	63	.80	8.06	5.18	10.28	8.86	8.90	5.24	9.70	8.34	8.40	5.38
2790	1, 1.5	5	+	150	0.33	50	100	.80	1.58	5.00	1.56	1.14	1.16	5.66	1.66	1.14	1.16	5.02
2791	1, 1.5	5	-	150	0.33	100	50	.80	11.50	4.88	15.28	13.28	13.34	5.48	15.52	13.12	13.18	5.18
2792	1, 1.5	5	+	150	0.50	37	113	.80	.52	4.84	.66	.52	.54	5.72	.56	.36	.36	4.84
2793	1, 1.5	5	-	150	0.50	113	37	.80	16.98	5.88	22.22	19.84	19.90	5.40	21.42	19.30	19.30	5.36
2794	1, 1.5	5	0	200	0	100	100	.80	5.10	5.10	6.52	5.28	5.32	5.08	6.38	5.30	5.34	5.34
2795	1, 1.5	5	+	200	0.16	84	116	.80	2.96	5.02	3.48	2.78	2.78	5.22	3.74	2.72	2.72	5.50
2796	1, 1.5	5	-	200	0.16	116	84	.80	8.20	5.08	10.26	8.78	8.80	5.44	10.54	9.12	9.12	5.76
2797	1, 1.5	5	+	200	0.33	67	133	.80	1.64	4.64	1.74	1.42	1.42	5.24	1.78	1.22	1.26	5.16
2798	1, 1.5	5	-	200	0.33	133	67	.80	11.84	5.28	15.58	13.78	13.88	5.50	16.44	14.24	14.28	5.60
2799	1, 1.5	5	+	200	0.50	50	150	.80	.64	4.90	.62	.48	.48	5.28	.48	.26	.26	5.14
2800	1, 1.5	5	-	200	0.50	150	50	.80	16.82	5.22	22.40	20.10	20.22	5.90	23.42	20.84	20.92	5.06
2801	1, 1.5	1	0	20	0	10	10	.90	4.02	3.64	5.78	4.46	4.98	4.30	5.48	4.66	5.04	4.36
2802	1, 1.5	1	0	20	0.16	8	12	.90	4.76	4.22	5.52	4.64	5.02	4.46	5.06	3.94	4.40	4.24
2803	1, 1.5	1	0	20	0.33	7	13	.90	4.90	5.32	5.48	4.52	5.02	4.90	5.22	4.26	4.54	4.72
2804	1, 1.5	1	0	20	0.50	5	15	.90	4.76	7.26	5.46	4.86	5.04	6.04	5.14	4.46	4.72	6.34
2805	1, 1.5	1	0	40	0	20	20	.90	5.00	4.78	5.66	4.92	5.12	4.74	5.26	4.66	4.84	4.58
2806	1, 1.5	1	0	40	0.16	17	23	.90	4.86	4.70	5.42	4.76	4.88	4.94	5.10	4.32	4.60	4.42
2807	1, 1.5	1	0	40	0.33	13	27	.90	4.64	4.94	5.32	4.56	4.66	4.88	5.04	4.42	4.50	4.94
2808	1, 1.5	1	0	40	0.50	10	30	.90	4.34	6.06	4.98	4.38	4.48	5.88	5.74	4.96	5.20	5.88
2809	1, 1.5	1	0	60	0	30	30	.90	4.62	4.56	5.42	4.60	4.78	4.76	5.48	4.64	4.88	4.84
2810	1, 1.5	1	0	60	0.16	25	35	.90	4.68	4.56	5.42	4.68	4.90	4.70	5.66	4.82	4.88	5.00
2811	1, 1.5	1	0	60	0.33	20	40	.90	5.18	5.40	5.46	4.88	4.98	5.00	5.18	4.58	4.72	4.82
2812	1, 1.5	1	0	60	0.50	15	45	.90	4.88	5.68	5.58	5.12	5.20	5.26	5.42	4.74	4.88	5.30
2813	1, 1.5	1	0	80	0	40	40	.90	4.24	4.36	6.42	5.66	5.78	5.56	5.14	4.66	4.72	4.60
2814	1, 1.5	1	0	80	0.16	34	46	.90	4.50	4.78	6.20	5.62	5.74	5.76	5.42	4.88	4.98	5.12

	D	VR	P	N	Δn	n1	n2	ε	F g	B-F g	F t	F-GG t	F-HF t	B-F t	F g x t	F-GG g x t	F-HF g x t	B-F g x t
2815	1, 1.5	1	0	80	0.33	27	53	.90	5.14	5.36	6.04	5.50	5.60	5.92	5.16	4.50	4.68	4.90
2816	1, 1.5	1	0	80	0.50	20	60	.90	5.34	6.26	5.76	5.14	5.22	5.86	5.30	4.72	4.84	5.22
2817	1, 1.5	1	0	100	0	50	50	.90	4.98	5.10	5.58	4.78	4.92	5.02	4.96	4.48	4.52	4.58
2818	1, 1.5	1	0	100	0.16	42	58	.90	4.84	5.02	5.82	5.24	5.28	5.44	5.12	4.66	4.76	4.78
2819	1, 1.5	1	0	100	0.33	33	67	.90	4.96	5.28	5.64	4.92	5.06	5.50	5.56	4.84	4.94	5.38
2820	1, 1.5	1	0	100	0.50	25	75	.90	4.72	5.50	5.68	5.08	5.12	5.62	5.16	4.26	4.38	4.80
2821	1, 1.5	1	0	150	0	75	75	.90	5.74	5.64	5.94	5.22	5.26	5.32	5.90	5.36	5.40	5.86
2822	1, 1.5	1	0	150	0.16	63	87	.90	5.64	5.80	5.78	5.24	5.26	5.60	5.66	4.90	4.98	5.38
2823	1, 1.5	1	0	150	0.33	50	100	.90	5.02	5.28	5.96	5.28	5.34	5.62	5.12	4.64	4.66	5.00
2824	1, 1.5	1	0	150	0.50	37	113	.90	5.20	5.70	5.90	5.24	5.40	5.92	5.24	4.82	4.84	5.36
2825	1, 1.5	1	0	200	0	100	100	.90	5.04	5.28	5.78	5.30	5.34	5.40	5.58	5.14	5.20	5.24
2826	1, 1.5	1	0	200	0.16	84	116	.90	5.18	5.46	5.94	5.34	5.40	5.46	5.86	5.38	5.40	5.50
2827	1, 1.5	1	0	200	0.33	67	133	.90	5.18	5.42	6.10	5.56	5.60	5.66	5.64	5.10	5.12	5.42
2828	1, 1.5	1	0	200	0.50	50	150	.90	4.82	5.28	5.82	5.34	5.36	5.60	5.14	4.40	4.46	5.02
2829	1, 1.5	1.5	0	20	0	10	10	.90	4.08	3.80	5.66	4.62	4.98	4.34	5.44	4.48	4.86	4.28
2830	1, 1.5	1.5	+	20	0.16	8	12	.90	3.96	4.16	4.62	3.80	4.16	4.26	4.22	3.30	3.62	4.00
2831	1, 1.5	1.5	-	20	0.16	12	8	.90	5.66	5.04	6.60	5.56	5.92	4.36	6.56	5.42	5.78	4.20
2832	1, 1.5	1.5	+	20	0.33	7	13	.90	4.00	4.54	4.22	3.22	3.70	4.12	3.86	3.10	3.36	4.04
2833	1, 1.5	1.5	-	20	0.33	13	7	.90	6.14	5.66	7.18	5.98	6.46	5.00	7.16	6.32	6.64	4.56
2834	1, 1.5	1.5	+	20	0.50	5	15	.90	3.16	6.44	3.32	2.68	2.92	5.70	3.06	2.54	2.68	5.70
2835	1, 1.5	1.5	-	20	0.50	15	5	.90	7.66	8.30	8.76	7.56	7.92	7.00	9.20	7.96	8.50	6.92
2836	1, 1.5	1.5	0	40	0	20	20	.90	5.18	4.76	6.00	5.10	5.28	4.84	5.38	4.70	5.04	4.52
2837	1, 1.5	1.5	+	40	0.16	17	23	.90	4.24	4.66	4.62	4.00	4.18	4.84	4.18	3.64	3.78	4.30
2838	1, 1.5	1.5	-	40	0.16	23	17	.90	5.42	5.04	6.94	6.20	6.44	5.02	6.30	5.46	5.76	5.04
2839	1, 1.5	1.5	+	40	0.33	13	27	.90	3.20	4.78	3.46	2.84	3.00	4.88	3.46	3.00	3.08	4.80
2840	1, 1.5	1.5	-	40	0.33	27	13	.90	6.42	5.36	8.28	7.28	7.58	5.36	7.08	6.14	6.48	4.82
2841	1, 1.5	1.5	+	40	0.50	10	30	.90	2.50	5.34	2.86	2.56	2.68	5.40	3.22	2.80	2.88	5.56
2842	1, 1.5	1.5	-	40	0.50	30	10	.90	7.10	6.64	8.76	7.82	8.06	5.94	8.54	7.58	7.94	5.70
2843	1, 1.5	1.5	0	60	0	30	30	.90	4.38	4.40	5.54	4.76	4.88	4.66	5.34	4.66	4.80	4.80
2844	1, 1.5	1.5	+	60	0.16	25	35	.90	3.96	4.56	4.42	3.84	3.94	4.66	4.64	4.02	4.14	4.84
2845	1, 1.5	1.5	-	60	0.16	35	25	.90	5.44	4.56	6.46	5.68	5.90	4.98	6.62	6.00	6.14	5.34
2846	1, 1.5	1.5	+	60	0.33	20	40	.90	3.70	5.20	3.76	3.38	3.44	4.92	3.68	3.16	3.30	4.94

	D	VR	P	N	Δn	n1	n2	ε	F g	B-F g	F t	F-GG t	F-HF t	B-F t	F g x t	F-GG g x t	F-HF g x t	B-F g x t
2847	1, 1.5	1.5	-	60	0.33	40	20	.90	6.42	5.32	7.84	6.86	7.04	5.22	7.24	6.28	6.42	5.06
2848	1, 1.5	1.5	+	60	0.50	15	45	.90	3.24	5.36	3.30	2.58	2.64	5.08	3.04	2.52	2.64	5.10
2849	1, 1.5	1.5	-	60	0.50	45	15	.90	7.48	6.00	9.48	8.64	8.82	5.80	8.96	8.00	8.20	5.70
2850	1, 1.5	1.5	0	80	0	40	40	.90	4.34	4.28	6.50	5.60	5.74	5.46	5.34	4.82	4.90	4.70
2851	1, 1.5	1.5	+	80	0.16	34	46	.90	3.90	4.68	5.48	4.94	5.02	5.90	4.88	4.26	4.42	5.42
2852	1, 1.5	1.5	-	80	0.16	46	34	.90	5.60	4.72	7.48	6.60	6.74	5.74	6.28	5.74	5.84	4.84
2853	1, 1.5	1.5	+	80	0.33	27	53	.90	3.46	5.26	4.26	3.76	3.88	5.82	3.48	3.06	3.12	4.80
2854	1, 1.5	1.5	-	80	0.33	53	27	.90	6.34	4.82	8.56	7.66	7.88	5.74	7.64	6.88	7.04	4.74
2855	1, 1.5	1.5	+	80	0.50	20	60	.90	3.04	5.96	3.52	2.98	3.16	5.62	2.90	2.52	2.54	5.22
2856	1, 1.5	1.5	-	80	0.50	60	20	.90	7.54	5.72	10.34	9.22	9.46	6.02	8.78	8.06	8.24	5.32
2857	1, 1.5	1.5	0	100	0	50	50	.90	5.04	4.94	5.58	4.76	4.86	4.72	4.84	4.26	4.30	4.64
2858	1, 1.5	1.5	+	100	0.16	42	58	.90	4.14	4.98	4.60	4.12	4.16	5.10	4.16	3.66	3.66	4.78
2859	1, 1.5	1.5	-	100	0.16	58	42	.90	6.08	5.48	6.56	5.90	5.96	5.04	6.62	6.04	6.14	4.88
2860	1, 1.5	1.5	+	100	0.33	33	67	.90	3.42	5.12	4.06	3.66	3.74	5.32	3.86	3.46	3.48	5.30
2861	1, 1.5	1.5	-	100	0.33	67	33	.90	7.22	5.74	7.72	6.94	7.02	5.32	7.68	7.08	7.12	5.28
2862	1, 1.5	1.5	+	100	0.50	25	75	.90	3.00	5.20	3.16	2.68	2.76	5.44	2.52	2.28	2.32	4.76
2863	1, 1.5	1.5	-	100	0.50	75	25	.90	8.08	6.14	8.74	7.82	7.94	5.04	9.44	8.58	8.68	5.56
2864	1, 1.5	1.5	0	150	0	75	75	.90	5.84	5.96	6.10	5.36	5.42	5.72	5.92	5.28	5.36	5.56
2865	1, 1.5	1.5	+	150	0.16	63	87	.90	4.86	5.76	5.00	4.28	4.32	5.38	4.54	4.04	4.12	5.06
2866	1, 1.5	1.5	-	150	0.16	87	63	.90	6.22	5.42	6.94	6.42	6.44	5.32	6.70	5.96	6.08	5.10
2867	1, 1.5	1.5	+	150	0.33	50	100	.90	3.58	4.94	4.24	3.64	3.76	5.80	3.58	3.26	3.28	4.84
2868	1, 1.5	1.5	-	150	0.33	100	50	.90	6.42	5.06	8.16	7.42	7.52	5.70	7.60	6.82	6.84	5.10
2869	1, 1.5	1.5	+	150	0.50	37	113	.90	3.02	5.36	3.36	3.08	3.10	5.86	3.02	2.72	2.72	5.06
2870	1, 1.5	1.5	-	150	0.50	113	37	.90	7.74	5.52	9.26	8.20	8.30	5.42	8.54	7.80	7.94	5.20
2871	1, 1.5	1.5	0	200	0	100	100	.90	5.18	5.34	5.64	5.12	5.14	5.18	5.72	5.32	5.38	5.40
2872	1, 1.5	1.5	+	200	0.16	84	116	.90	4.36	5.36	4.96	4.56	4.58	5.68	4.72	4.22	4.26	5.08
2873	1, 1.5	1.5	-	200	0.16	116	84	.90	5.74	5.18	6.64	6.10	6.10	5.58	6.78	5.98	6.00	5.28
2874	1, 1.5	1.5	+	200	0.33	67	133	.90	3.68	5.14	4.14	3.60	3.62	5.48	4.00	3.62	3.62	5.08
2875	1, 1.5	1.5	-	200	0.33	133	67	.90	6.74	5.38	8.24	7.32	7.40	5.18	8.14	7.42	7.46	5.42
2876	1, 1.5	1.5	+	200	0.50	50	150	.90	2.98	5.20	3.34	3.00	3.02	5.58	2.70	2.32	2.36	4.90
2877	1, 1.5	1.5	-	200	0.50	150	50	.90	7.74	5.54	9.66	8.58	8.60	5.62	9.48	8.62	8.68	5.50
2878	1, 1.5	2	0	20	0	10	10	.90	4.10	4.00	5.72	4.66	5.06	4.20	5.48	4.42	4.82	3.88

	D	VR	P	N	Δn	n1	n2	ε	F g	B-F g	F t	F-GG t	F-HF t	B-F t	F g x t	F-GG g x t	F-HF g x t	B-F g x t
2879	1, 1.5	2	+	20	0.16	8	12	.90	3.62	3.94	3.98	3.22	3.52	4.18	3.64	2.92	3.10	3.96
2880	1, 1.5	2	-	20	0.16	12	8	.90	6.60	5.14	7.78	6.46	6.80	4.64	7.62	6.38	6.84	4.48
2881	1, 1.5	2	+	20	0.33	7	13	.90	3.66	4.50	3.16	2.40	2.68	3.80	3.10	2.44	2.72	3.94
2882	1, 1.5	2	-	20	0.33	13	7	.90	7.28	6.04	8.68	7.50	7.84	5.12	8.74	7.46	7.98	4.70
2883	1, 1.5	2	+	20	0.50	5	15	.90	2.48	5.82	2.06	1.50	1.66	5.50	2.08	1.78	1.90	5.28
2884	1, 1.5	2	-	20	0.50	15	5	.90	9.64	8.82	11.58	10.10	10.50	7.10	11.88	10.42	10.92	7.18
2885	1, 1.5	2	0	40	0	20	20	.90	5.22	4.94	5.94	5.16	5.42	4.80	5.42	4.92	5.08	4.54
2886	1, 1.5	2	+	40	0.16	17	23	.90	3.90	4.40	4.20	3.56	3.76	4.76	3.80	3.22	3.42	4.30
2887	1, 1.5	2	-	40	0.16	23	17	.90	6.00	4.92	7.74	6.86	7.22	5.00	6.90	6.12	6.28	4.86
2888	1, 1.5	2	+	40	0.33	13	27	.90	2.50	4.74	2.44	2.10	2.26	4.68	2.66	2.38	2.44	4.58
2889	1, 1.5	2	-	40	0.33	27	13	.90	7.76	5.52	10.02	9.08	9.36	5.20	9.18	7.92	8.16	5.20
2890	1, 1.5	2	+	40	0.50	10	30	.90	1.92	4.94	1.88	1.60	1.70	5.20	2.06	1.54	1.66	5.22
2891	1, 1.5	2	-	40	0.50	30	10	.90	9.60	6.80	11.72	10.62	10.92	5.90	11.78	10.62	10.82	6.12
2892	1, 1.5	2	0	60	0	30	30	.90	4.34	4.34	5.42	4.66	4.80	4.72	5.28	4.76	4.80	4.76
2893	1, 1.5	2	+	60	0.16	25	35	.90	3.66	4.60	3.76	3.34	3.40	4.76	4.18	3.60	3.72	4.90
2894	1, 1.5	2	-	60	0.16	35	25	.90	6.06	4.70	7.28	6.64	6.76	4.98	7.16	6.54	6.62	5.32
2895	1, 1.5	2	+	60	0.33	20	40	.90	2.66	5.12	2.96	2.56	2.68	4.86	2.74	2.40	2.50	4.68
2896	1, 1.5	2	-	60	0.33	40	20	.90	7.94	5.44	9.78	8.80	9.12	5.36	9.18	8.20	8.38	5.10
2897	1, 1.5	2	+	60	0.50	15	45	.90	2.36	5.34	1.88	1.70	1.70	5.04	1.94	1.70	1.78	4.94
2898	1, 1.5	2	-	60	0.50	45	15	.90	9.58	6.24	12.72	11.68	11.88	5.78	12.16	11.04	11.22	5.68
2899	1, 1.5	2	0	80	0	40	40	.90	4.56	4.36	6.40	5.44	5.54	5.56	5.32	4.82	4.86	4.84
2900	1, 1.5	2	+	80	0.16	34	46	.90	3.60	4.52	4.82	4.34	4.34	5.96	4.42	3.86	3.92	5.50
2901	1, 1.5	2	-	80	0.16	46	34	.90	6.24	4.98	8.14	7.22	7.34	5.68	6.80	6.22	6.32	5.02
2902	1, 1.5	2	+	80	0.33	27	53	.90	2.82	5.28	3.42	2.80	2.92	5.90	2.66	2.38	2.40	4.68
2903	1, 1.5	2	-	80	0.33	53	27	.90	7.62	5.00	10.64	9.56	9.72	5.58	9.34	8.42	8.64	4.96
2904	1, 1.5	2	+	80	0.50	20	60	.90	2.40	5.88	2.36	2.10	2.14	5.56	1.94	1.64	1.66	5.22
2905	1, 1.5	2	-	80	0.50	60	20	.90	9.46	5.78	13.24	12.16	12.36	5.82	12.38	10.96	11.28	5.40
2906	1, 1.5	2	0	100	0	50	50	.90	5.12	4.96	5.68	4.82	4.90	4.88	4.86	4.42	4.44	4.50
2907	1, 1.5	2	+	100	0.16	42	58	.90	3.72	5.04	4.00	3.62	3.68	5.10	3.64	3.28	3.32	4.62
2908	1, 1.5	2	-	100	0.16	58	42	.90	6.66	5.60	7.36	6.68	6.78	5.02	7.34	6.50	6.60	4.96
2909	1, 1.5	2	+	100	0.33	33	67	.90	2.64	5.14	3.20	2.62	2.70	5.24	2.86	2.56	2.60	5.20
2910	1, 1.5	2	-	100	0.33	67	33	.90	8.48	5.42	9.54	8.70	8.78	4.94	9.88	8.94	9.04	4.58

	D	VR	P	N	Δn	n1	n2	ε	F g	B-F g	F t	F-GG t	F-HF t	B-F t	F g x t	F-GG g x t	F-HF g x t	B-F g x t
2911	1, 1.5	2	+	100	0.50	25	75	.90	1.94	5.02	2.00	1.76	1.82	5.58	1.64	1.44	1.44	4.58
2912	1, 1.5	2	-	100	0.50	75	25	.90	10.46	6.26	11.98	11.00	11.20	5.02	12.44	11.40	11.54	5.40
2913	1, 1.5	2	0	150	0	75	75	.90	5.70	5.76	6.04	5.46	5.56	5.92	5.92	5.36	5.44	5.42
2914	1, 1.5	2	+	150	0.16	63	87	.90	4.52	5.62	4.52	3.86	3.92	5.54	3.98	3.44	3.44	5.06
2915	1, 1.5	2	-	150	0.16	87	63	.90	6.52	5.22	7.52	6.84	6.92	5.16	7.48	6.50	6.62	4.96
2916	1, 1.5	2	+	150	0.33	50	100	.90	3.00	4.92	3.04	2.54	2.60	5.60	2.88	2.62	2.62	4.94
2917	1, 1.5	2	-	150	0.33	100	50	.90	7.66	5.22	9.80	8.88	8.98	5.60	9.34	8.50	8.52	5.16
2918	1, 1.5	2	+	150	0.50	37	113	.90	1.88	5.30	2.12	1.76	1.80	6.06	2.02	1.70	1.76	4.96
2919	1, 1.5	2	-	150	0.50	113	37	.90	9.44	5.62	12.00	11.16	11.24	5.32	11.42	10.50	10.58	5.20
2920	1, 1.5	2	0	200	0	100	100	.90	5.36	5.06	5.58	5.12	5.14	5.08	5.80	5.34	5.38	5.32
2921	1, 1.5	2	+	200	0.16	84	116	.90	3.76	5.16	4.46	3.88	3.92	5.40	4.18	3.72	3.76	5.12
2922	1, 1.5	2	-	200	0.16	116	84	.90	6.44	4.80	7.48	6.90	6.96	5.18	7.42	6.70	6.80	4.86
2923	1, 1.5	2	+	200	0.33	67	133	.90	3.02	5.06	3.08	2.78	2.80	5.52	2.84	2.44	2.48	5.12
2924	1, 1.5	2	-	200	0.33	133	67	.90	8.04	5.26	10.08	9.38	9.42	5.30	10.24	9.12	9.16	5.44
2925	1, 1.5	2	+	200	0.50	50	150	.90	2.18	5.14	2.18	1.80	1.82	5.70	1.78	1.56	1.60	4.96
2926	1, 1.5	2	-	200	0.50	150	50	.90	10.44	5.74	12.88	11.80	11.94	5.58	12.48	11.52	11.58	5.50
2927	1, 1.5	5	0	20	0	10	10	.90	5.46	4.64	6.20	5.16	5.56	4.46	6.00	4.90	5.30	4.30
2928	1, 1.5	5	+	20	0.16	8	12	.90	3.52	4.56	2.80	2.22	2.40	4.16	2.64	2.28	2.34	4.02
2929	1, 1.5	5	-	20	0.16	12	8	.90	9.40	5.82	11.26	9.62	10.16	4.72	10.84	9.44	9.80	4.32
2930	1, 1.5	5	+	20	0.33	7	13	.90	2.54	4.86	1.62	1.30	1.40	4.36	1.76	1.26	1.50	4.08
2931	1, 1.5	5	-	20	0.33	13	7	.90	12.08	6.58	14.64	12.50	13.12	5.58	14.72	12.18	13.06	4.98
2932	1, 1.5	5	+	20	0.50	5	15	.90	1.26	4.64	.54	.40	.44	4.66	.62	.44	.52	4.12
2933	1, 1.5	5	-	20	0.50	15	5	.90	18.22	9.86	23.24	20.40	21.30	7.28	22.98	20.38	21.26	6.92
2934	1, 1.5	5	0	40	0	20	20	.90	5.50	4.78	6.10	5.06	5.42	4.60	5.56	4.96	5.16	4.52
2935	1, 1.5	5	+	40	0.16	17	23	.90	3.34	4.42	3.06	2.40	2.56	4.44	2.92	2.40	2.50	4.12
2936	1, 1.5	5	-	40	0.16	23	17	.90	8.06	5.00	9.84	8.60	8.80	4.86	9.26	8.30	8.48	4.80
2937	1, 1.5	5	+	40	0.33	13	27	.90	1.42	4.22	.96	.82	.84	4.70	1.16	1.00	1.00	4.26
2938	1, 1.5	5	-	40	0.33	27	13	.90	12.24	5.78	15.98	14.74	15.12	5.30	15.08	13.50	13.86	5.18
2939	1, 1.5	5	+	40	0.50	10	30	.90	.86	4.30	.50	.42	.42	4.92	.52	.40	.42	4.64
2940	1, 1.5	5	-	40	0.50	30	10	.90	16.82	6.94	22.28	20.46	20.78	5.94	22.26	20.22	20.72	6.18
2941	1, 1.5	5	0	60	0	30	30	.90	4.70	4.16	6.00	5.12	5.28	4.84	5.68	4.96	5.10	4.76
2942	1, 1.5	5	+	60	0.16	25	35	.90	3.04	4.48	2.92	2.48	2.56	4.58	3.06	2.56	2.62	4.84

	D	VR	P	N	Δn	n1	n2	ε	F g	B-F g	F t	F-GG t	F-HF t	B-F t	F g x t	F-GG g x t	F-HF g x t	B-F g x t
2943	1, 1.5	5	-	60	0.16	35	25	.90	7.92	4.66	9.90	8.88	9.14	5.10	9.94	8.74	8.94	5.42
2944	1, 1.5	5	+	60	0.33	20	40	.90	1.60	4.94	1.40	1.22	1.24	4.70	1.44	1.12	1.26	4.62
2945	1, 1.5	5	-	60	0.33	40	20	.90	11.74	5.46	15.52	14.12	14.44	5.34	15.34	14.12	14.50	5.12
2946	1, 1.5	5	+	60	0.50	15	45	.90	.82	5.02	.56	.46	.50	4.82	.54	.36	.42	4.68
2947	1, 1.5	5	-	60	0.50	45	15	.90	16.86	6.48	22.78	21.16	21.42	5.50	22.66	21.08	21.36	5.68
2948	1, 1.5	5	0	80	0	40	40	.90	4.92	4.62	6.08	5.30	5.44	5.06	5.52	4.94	5.06	4.72
2949	1, 1.5	5	+	80	0.16	34	46	.90	2.84	4.30	3.82	3.16	3.28	5.38	3.24	2.98	3.04	4.90
2950	1, 1.5	5	-	80	0.16	46	34	.90	7.98	5.14	10.16	9.26	9.44	5.36	8.96	8.00	8.14	5.04
2951	1, 1.5	5	+	80	0.33	27	53	.90	1.60	4.88	1.68	1.72	1.78	5.96	1.62	1.50	1.50	4.80
2952	1, 1.5	5	-	80	0.33	53	27	.90	11.64	5.18	15.98	14.62	14.78	5.44	15.16	13.76	13.90	4.86
2953	1, 1.5	5	+	80	0.50	20	60	.90	.88	5.22	.66	.50	.54	5.82	.32	.30	.30	5.28
2954	1, 1.5	5	-	80	0.50	60	20	.90	16.36	5.82	23.64	22.16	22.48	5.52	22.66	21.32	21.50	5.12
2955	1, 1.5	5	0	100	0	50	50	.90	5.08	4.72	5.76	5.26	5.34	5.10	5.40	4.88	4.94	4.80
2956	1, 1.5	5	+	100	0.16	42	58	.90	2.76	4.78	3.26	2.94	2.98	4.82	2.76	2.38	2.40	4.62
2957	1, 1.5	5	-	100	0.16	58	42	.90	8.16	5.12	9.32	8.56	8.62	5.28	9.72	8.52	8.72	4.86
2958	1, 1.5	5	+	100	0.33	33	67	.90	1.28	4.54	1.38	1.14	1.18	4.96	1.24	1.04	1.04	5.06
2959	1, 1.5	5	-	100	0.33	67	33	.90	12.14	5.34	15.32	14.26	14.34	4.92	16.02	14.64	14.78	4.20
2960	1, 1.5	5	+	100	0.50	25	75	.90	.58	4.60	.40	.34	.34	5.26	.32	.32	.32	4.60
2961	1, 1.5	5	-	100	0.50	75	25	.90	17.42	6.10	21.64	20.46	20.60	4.92	22.38	20.60	20.72	5.32
2962	1, 1.5	5	0	150	0	75	75	.90	5.72	5.62	5.84	5.12	5.14	5.26	5.90	5.28	5.32	5.50
2963	1, 1.5	5	+	150	0.16	63	87	.90	3.36	5.34	2.86	2.54	2.60	5.46	2.92	2.40	2.46	4.94
2964	1, 1.5	5	-	150	0.16	87	63	.90	8.30	5.18	9.42	8.50	8.64	5.08	9.86	8.80	8.92	5.14
2965	1, 1.5	5	+	150	0.33	50	100	.90	1.58	5.14	1.38	1.14	1.16	5.24	1.30	1.06	1.06	5.44
2966	1, 1.5	5	-	150	0.33	100	50	.90	11.48	5.32	15.30	14.14	14.22	5.32	15.18	14.06	14.20	5.28
2967	1, 1.5	5	+	150	0.50	37	113	.90	.56	4.98	.54	.46	.48	5.56	.56	.46	.48	4.78
2968	1, 1.5	5	-	150	0.50	113	37	.90	17.14	5.54	22.66	21.14	21.26	5.10	21.34	20.02	20.14	5.00
2969	1, 1.5	5	0	200	0	100	100	.90	5.36	5.24	5.52	5.02	5.02	5.08	5.84	5.40	5.44	5.34
2970	1, 1.5	5	+	200	0.16	84	116	.90	2.96	5.24	3.26	2.92	2.96	5.14	3.14	2.70	2.72	5.38
2971	1, 1.5	5	-	200	0.16	116	84	.90	7.84	4.94	9.72	8.74	8.78	4.96	9.56	8.78	8.86	4.74
2972	1, 1.5	5	+	200	0.33	67	133	.90	1.88	4.74	1.48	1.26	1.26	5.52	1.36	1.18	1.20	5.24
2973	1, 1.5	5	-	200	0.33	133	67	.90	12.12	5.28	15.92	14.80	14.82	5.50	15.92	14.62	14.76	5.76
2974	1, 1.5	5	+	200	0.50	50	150	.90	.54	4.86	.46	.36	.40	5.44	.30	.18	.18	4.82

	D	VR	P	N	Δn	n1	n2	ε	F g	B-F g	F t	F-GG t	F-HF t	B-F t	F g x t	F-GG g x t	F-HF g x t	B-F g x t
2975	1, 1.5	5	-	200	0.50	150	50	.90	17.24	5.76	22.78	21.74	21.78	5.50	22.98	21.54	21.60	5.58
2976	1, 1.5	1	0	20	0	10	10	1	4.06	3.78	4.80	4.08	4.52	4.04	5.00	4.34	4.70	4.24
2977	1, 1.5	1	0	20	0.16	8	12	1	4.72	4.34	4.50	4.08	4.32	3.74	4.80	4.24	4.54	4.04
2978	1, 1.5	1	0	20	0.33	7	13	1	4.88	5.34	4.84	4.28	4.64	4.40	4.92	4.38	4.76	4.70
2979	1, 1.5	1	0	20	0.50	5	15	1	4.90	7.08	5.20	4.62	4.94	5.86	5.20	4.42	4.88	5.58
2980	1, 1.5	1	0	40	0	20	20	1	5.06	4.86	4.74	4.24	4.50	4.48	4.66	4.54	4.58	4.28
2981	1, 1.5	1	0	40	0.16	17	23	1	5.04	5.02	4.68	4.40	4.54	4.56	4.72	4.42	4.54	4.42
2982	1, 1.5	1	0	40	0.33	13	27	1	4.38	5.14	4.82	4.46	4.62	4.86	4.88	4.68	4.76	5.04
2983	1, 1.5	1	0	40	0.50	10	30	1	4.24	5.96	4.64	4.34	4.50	5.86	5.12	4.78	4.98	5.60
2984	1, 1.5	1	0	60	0	30	30	1	4.82	4.74	4.98	4.76	4.86	4.72	4.96	4.78	4.90	5.00
2985	1, 1.5	1	0	60	0.16	25	35	1	4.84	5.00	5.24	4.94	5.10	4.86	4.88	4.60	4.80	4.82
2986	1, 1.5	1	0	60	0.33	20	40	1	5.32	5.66	5.22	4.74	5.00	5.02	5.28	4.90	5.10	4.88
2987	1, 1.5	1	0	60	0.50	15	45	1	4.90	6.04	4.84	4.74	4.76	5.20	5.00	4.80	4.96	5.36
2988	1, 1.5	1	0	80	0	40	40	1	4.68	4.66	5.74	5.50	5.62	5.40	4.58	4.44	4.46	4.34
2989	1, 1.5	1	0	80	0.16	34	46	1	4.90	4.84	5.58	5.36	5.44	5.40	5.22	5.06	5.14	5.04
2990	1, 1.5	1	0	80	0.33	27	53	1	4.98	5.16	5.28	5.20	5.26	5.32	4.78	4.62	4.74	4.98
2991	1, 1.5	1	0	80	0.50	20	60	1	5.28	6.26	5.32	5.08	5.24	5.78	4.68	4.58	4.62	5.08
2992	1, 1.5	1	0	100	0	50	50	1	5.30	5.34	5.24	5.12	5.20	5.26	4.94	4.76	4.88	5.00
2993	1, 1.5	1	0	100	0.16	42	58	1	5.04	5.14	5.40	5.16	5.30	5.28	4.78	4.66	4.76	5.02
2994	1, 1.5	1	0	100	0.33	33	67	1	5.06	5.50	5.12	4.98	5.00	5.38	5.18	5.00	5.06	5.48
2995	1, 1.5	1	0	100	0.50	25	75	1	4.84	5.36	4.88	4.78	4.82	5.40	4.50	4.28	4.40	4.70
2996	1, 1.5	1	0	150	0	75	75	1	5.68	5.64	5.16	5.00	5.08	5.04	5.54	5.48	5.48	5.64
2997	1, 1.5	1	0	150	0.16	63	87	1	5.58	5.78	5.36	5.28	5.30	5.28	5.26	5.22	5.24	5.44
2998	1, 1.5	1	0	150	0.33	50	100	1	5.20	5.40	5.14	5.02	5.10	5.14	5.18	5.10	5.12	5.26
2999	1, 1.5	1	0	150	0.50	37	113	1	5.26	5.78	5.10	5.04	5.06	5.30	4.94	4.84	4.88	5.22
3000	1, 1.5	1	0	200	0	100	100	1	5.16	5.18	5.04	4.96	5.04	5.10	5.62	5.62	5.62	5.58
3001	1, 1.5	1	0	200	0.16	84	116	1	5.20	5.30	5.06	5.04	5.06	5.06	5.58	5.58	5.58	5.52
3002	1, 1.5	1	0	200	0.33	67	133	1	5.30	5.44	5.34	5.28	5.32	5.54	5.54	5.38	5.42	5.54
3003	1, 1.5	1	0	200	0.50	50	150	1	5.02	5.34	4.98	4.86	4.94	5.42	5.02	5.00	5.02	5.28
3004	1, 1.5	1.5	0	20	0	10	10	1	4.06	3.72	4.74	3.98	4.50	3.88	5.02	4.36	4.72	4.08
3005	1, 1.5	1.5	+	20	0.16	8	12	1	3.92	4.16	3.52	3.04	3.34	3.76	4.00	3.36	3.68	4.14
3006	1, 1.5	1.5	-	20	0.16	12	8	1	5.54	4.76	5.98	5.26	5.54	4.24	6.22	5.50	5.92	4.08

	D	VR	P	N	Δn	n1	n2	ε	F g	B-F g	F t	F-GG t	F-HF t	B-F t	F g x t	F-GG g x t	F-HF g x t	B-F g x t
3007	1, 1.5	1.5	+	20	0.33	7	13	1	3.98	4.96	3.18	2.72	2.94	4.16	3.42	2.90	3.26	4.36
3008	1, 1.5	1.5	-	20	0.33	13	7	1	6.30	5.68	6.52	5.90	6.32	4.62	6.76	6.02	6.46	4.42
3009	1, 1.5	1.5	+	20	0.50	5	15	1	3.20	5.98	2.82	2.38	2.56	5.34	2.60	2.20	2.42	5.08
3010	1, 1.5	1.5	-	20	0.50	15	5	1	7.76	8.44	8.16	7.24	7.84	6.72	9.22	8.10	8.66	6.60
3011	1, 1.5	1.5	0	40	0	20	20	1	5.00	5.04	4.60	4.24	4.46	4.26	4.62	4.40	4.54	4.28
3012	1, 1.5	1.5	+	40	0.16	17	23	1	4.36	4.64	4.10	3.78	3.90	4.52	3.76	3.62	3.68	4.26
3013	1, 1.5	1.5	-	40	0.16	23	17	1	5.60	5.24	5.70	5.30	5.50	4.78	5.68	5.16	5.50	4.56
3014	1, 1.5	1.5	+	40	0.33	13	27	1	3.04	4.42	3.12	2.82	3.00	4.76	3.28	3.10	3.22	4.86
3015	1, 1.5	1.5	-	40	0.33	27	13	1	6.76	5.56	7.34	6.76	7.10	4.90	6.52	6.00	6.26	4.16
3016	1, 1.5	1.5	+	40	0.50	10	30	1	2.48	5.22	2.50	2.34	2.40	5.50	2.84	2.54	2.72	5.78
3017	1, 1.5	1.5	-	40	0.50	30	10	1	7.34	6.68	8.30	7.84	8.06	5.48	8.20	7.54	7.82	5.02
3018	1, 1.5	1.5	0	60	0	30	30	1	4.90	4.80	4.96	4.80	4.84	4.80	5.26	4.92	5.14	4.76
3019	1, 1.5	1.5	+	60	0.16	25	35	1	4.06	4.68	4.12	3.92	4.00	4.80	3.78	3.60	3.64	4.54
3020	1, 1.5	1.5	-	60	0.16	35	25	1	5.30	4.38	6.32	6.04	6.18	5.00	6.30	6.08	6.20	4.72
3021	1, 1.5	1.5	+	60	0.33	20	40	1	3.74	5.34	3.52	3.36	3.46	4.86	3.44	3.18	3.30	5.16
3022	1, 1.5	1.5	-	60	0.33	40	20	1	6.20	4.98	7.30	6.96	7.10	4.72	6.54	6.30	6.42	4.24
3023	1, 1.5	1.5	+	60	0.50	15	45	1	3.10	5.50	2.82	2.72	2.78	4.90	2.86	2.68	2.80	5.46
3024	1, 1.5	1.5	-	60	0.50	45	15	1	7.50	5.62	8.82	8.44	8.64	5.34	8.50	8.20	8.34	5.04
3025	1, 1.5	1.5	0	80	0	40	40	1	4.80	4.72	6.00	5.70	5.82	5.62	4.54	4.36	4.46	4.44
3026	1, 1.5	1.5	+	80	0.16	34	46	1	4.00	4.68	4.84	4.56	4.64	5.60	4.46	4.28	4.36	5.28
3027	1, 1.5	1.5	-	80	0.16	46	34	1	5.50	4.92	6.72	6.50	6.60	5.50	5.70	5.48	5.58	4.68
3028	1, 1.5	1.5	+	80	0.33	27	53	1	3.48	5.04	3.80	3.52	3.68	5.54	3.04	2.94	3.02	4.88
3029	1, 1.5	1.5	-	80	0.33	53	27	1	6.04	4.94	7.80	7.58	7.66	5.44	7.36	7.12	7.26	4.76
3030	1, 1.5	1.5	+	80	0.50	20	60	1	3.24	6.06	2.80	2.66	2.74	5.52	2.36	2.28	2.34	5.16
3031	1, 1.5	1.5	-	80	0.50	60	20	1	7.50	5.64	9.28	8.98	9.14	5.66	8.76	8.48	8.66	5.20
3032	1, 1.5	1.5	0	100	0	50	50	1	5.24	5.28	5.26	5.06	5.16	5.30	4.96	4.82	4.94	4.80
3033	1, 1.5	1.5	+	100	0.16	42	58	1	4.30	5.02	4.50	4.26	4.40	5.04	3.76	3.70	3.72	4.88
3034	1, 1.5	1.5	-	100	0.16	58	42	1	6.16	5.42	5.80	5.60	5.76	4.68	5.88	5.66	5.78	4.84
3035	1, 1.5	1.5	+	100	0.33	33	67	1	3.44	5.06	3.50	3.44	3.46	5.24	3.36	3.22	3.26	5.36
3036	1, 1.5	1.5	-	100	0.33	67	33	1	7.26	5.66	7.28	6.96	7.12	5.16	7.40	7.30	7.32	5.02
3037	1, 1.5	1.5	+	100	0.50	25	75	1	2.88	5.16	2.62	2.46	2.56	5.12	2.48	2.40	2.48	4.66
3038	1, 1.5	1.5	-	100	0.50	75	25	1	8.06	6.06	8.12	7.86	7.96	5.04	8.56	8.28	8.44	5.24

	D	VR	P	N	Δn	n1	n2	ε	F g	B-F g	F t	F-GG t	F-HF t	B-F t	F g x t	F-GG g x t	F-HF g x t	B-F g x t
3039	1, 1.5	1.5	0	150	0	75	75	1	5.54	5.48	5.20	5.14	5.16	4.84	5.58	5.44	5.46	5.44
3040	1, 1.5	1.5	+	150	0.16	63	87	1	4.84	5.76	4.28	4.08	4.20	5.06	4.52	4.36	4.48	5.32
3041	1, 1.5	1.5	-	150	0.16	87	63	1	5.96	5.36	6.26	6.12	6.16	5.18	6.14	6.04	6.08	4.98
3042	1, 1.5	1.5	+	150	0.33	50	100	1	3.72	5.16	3.26	3.24	3.26	5.24	3.46	3.42	3.42	5.34
3043	1, 1.5	1.5	-	150	0.33	100	50	1	6.60	4.98	7.50	7.34	7.42	4.66	7.54	7.40	7.46	5.04
3044	1, 1.5	1.5	+	150	0.50	37	113	1	3.06	5.56	2.70	2.68	2.68	5.32	2.44	2.44	2.44	5.08
3045	1, 1.5	1.5	-	150	0.50	113	37	1	7.40	5.56	9.10	8.92	8.96	5.18	9.06	8.94	9.02	5.60
3046	1, 1.5	1.5	0	200	0	100	100	1	5.02	5.06	5.04	5.00	5.02	5.00	5.60	5.60	5.60	5.64
3047	1, 1.5	1.5	+	200	0.16	84	116	1	4.42	5.30	4.44	4.34	4.38	5.20	4.56	4.54	4.56	5.38
3048	1, 1.5	1.5	-	200	0.16	116	84	1	6.02	5.02	6.38	6.28	6.38	4.70	6.40	6.34	6.38	5.08
3049	1, 1.5	1.5	+	200	0.33	67	133	1	3.80	5.38	3.62	3.58	3.58	5.16	3.58	3.46	3.54	5.42
3050	1, 1.5	1.5	-	200	0.33	133	67	1	6.56	5.24	7.36	7.22	7.24	4.80	7.40	7.36	7.40	5.62
3051	1, 1.5	1.5	+	200	0.50	50	150	1	3.04	5.26	2.90	2.86	2.90	5.32	2.72	2.70	2.70	5.50
3052	1, 1.5	1.5	-	200	0.50	150	50	1	7.76	5.60	9.08	8.94	9.00	5.60	8.74	8.68	8.72	5.02
3053	1, 1.5	2	0	20	0	10	10	1	4.28	3.72	4.74	4.12	4.48	4.00	5.12	4.36	4.78	3.98
3054	1, 1.5	2	+	20	0.16	8	12	1	3.80	4.28	3.00	2.58	2.76	3.64	3.34	2.88	3.12	4.08
3055	1, 1.5	2	-	20	0.16	12	8	1	6.42	5.20	7.08	6.26	6.64	4.32	7.32	6.34	6.82	4.38
3056	1, 1.5	2	+	20	0.33	7	13	1	3.46	4.82	2.36	2.06	2.24	4.04	2.64	2.18	2.40	4.28
3057	1, 1.5	2	-	20	0.33	13	7	1	7.54	6.02	8.30	7.36	7.98	4.70	8.24	7.34	7.78	4.54
3058	1, 1.5	2	+	20	0.50	5	15	1	2.52	5.62	1.86	1.66	1.78	5.04	1.60	1.34	1.42	4.88
3059	1, 1.5	2	-	20	0.50	15	5	1	9.68	9.02	11.48	10.34	10.90	6.86	12.28	10.92	11.76	6.62
3060	1, 1.5	2	0	40	0	20	20	1	5.28	4.72	4.74	4.44	4.52	4.28	4.76	4.38	4.56	4.20
3061	1, 1.5	2	+	40	0.16	17	23	1	3.94	4.66	3.76	3.44	3.56	4.42	3.36	3.20	3.32	4.02
3062	1, 1.5	2	-	40	0.16	23	17	1	6.36	5.30	6.58	6.04	6.34	4.68	6.24	5.82	6.06	4.66
3063	1, 1.5	2	+	40	0.33	13	27	1	2.40	4.36	2.16	2.02	2.16	4.58	2.46	2.24	2.38	4.90
3064	1, 1.5	2	-	40	0.33	27	13	1	8.22	5.76	9.74	9.08	9.42	4.94	8.46	7.78	8.10	4.40
3065	1, 1.5	2	+	40	0.50	10	30	1	1.86	4.84	1.50	1.46	1.46	5.36	1.60	1.40	1.52	5.50
3066	1, 1.5	2	-	40	0.50	30	10	1	9.50	6.82	11.28	10.70	10.98	5.48	11.38	10.56	11.02	5.16
3067	1, 1.5	2	0	60	0	30	30	1	4.96	4.66	5.24	5.04	5.10	4.98	5.32	5.12	5.26	4.84
3068	1, 1.5	2	+	60	0.16	25	35	1	3.68	4.54	3.80	3.64	3.66	4.86	3.06	2.92	3.02	4.40
3069	1, 1.5	2	-	60	0.16	35	25	1	5.78	4.32	7.40	7.00	7.26	4.96	7.22	6.92	7.08	4.74
3070	1, 1.5	2	+	60	0.33	20	40	1	2.84	5.24	2.66	2.48	2.58	4.98	2.48	2.32	2.38	5.14

	D	VR	P	N	Δn	n1	n2	ε	F g	B-F g	F t	F-GG t	F-HF t	B-F t	F g x t	F-GG g x t	F-HF g x t	B-F g x t
3071	1, 1.5	2	-	60	0.33	40	20	1	7.40	5.16	9.32	9.02	9.20	4.70	8.46	8.08	8.32	4.14
3072	1, 1.5	2	+	60	0.50	15	45	1	2.26	5.24	1.70	1.54	1.60	4.90	1.82	1.72	1.74	5.44
3073	1, 1.5	2	-	60	0.50	45	15	1	9.36	5.90	11.96	11.52	11.78	4.96	11.36	11.04	11.24	5.16
3074	1, 1.5	2	0	80	0	40	40	1	4.74	4.62	5.78	5.62	5.72	5.54	4.66	4.50	4.58	4.48
3075	1, 1.5	2	+	80	0.16	34	46	1	3.54	4.50	4.44	4.26	4.36	5.76	4.00	3.80	3.88	5.40
3076	1, 1.5	2	-	80	0.16	46	34	1	6.00	4.88	7.36	7.10	7.24	5.44	6.44	6.20	6.28	4.70
3077	1, 1.5	2	+	80	0.33	27	53	1	2.80	4.92	2.78	2.58	2.66	5.48	2.10	1.98	2.06	4.74
3078	1, 1.5	2	-	80	0.33	53	27	1	7.20	5.10	9.66	9.24	9.40	5.46	9.14	8.88	8.98	4.82
3079	1, 1.5	2	+	80	0.50	20	60	1	2.26	5.72	1.90	1.86	1.86	5.60	1.32	1.30	1.30	5.10
3080	1, 1.5	2	-	80	0.50	60	20	1	9.64	5.60	12.80	12.42	12.70	5.60	12.32	11.98	12.16	5.14
3081	1, 1.5	2	0	100	0	50	50	1	5.18	5.10	5.22	5.02	5.12	4.92	4.80	4.70	4.78	4.90
3082	1, 1.5	2	+	100	0.16	42	58	1	3.92	4.90	4.04	3.78	3.98	5.24	3.36	3.24	3.30	4.58
3083	1, 1.5	2	-	100	0.16	58	42	1	6.62	5.46	6.72	6.52	6.62	4.78	6.90	6.60	6.70	4.80
3084	1, 1.5	2	+	100	0.33	33	67	1	2.64	5.08	2.42	2.24	2.32	5.24	2.36	2.32	2.34	5.04
3085	1, 1.5	2	-	100	0.33	67	33	1	8.52	5.78	9.18	8.90	9.02	5.08	9.30	9.12	9.26	5.10
3086	1, 1.5	2	+	100	0.50	25	75	1	2.08	4.84	1.60	1.50	1.56	5.14	1.46	1.44	1.46	4.58
3087	1, 1.5	2	-	100	0.50	75	25	1	10.36	6.20	11.48	11.18	11.32	4.90	11.72	11.46	11.54	5.18
3088	1, 1.5	2	0	150	0	75	75	1	5.56	5.40	5.18	5.06	5.12	5.04	5.46	5.32	5.38	5.40
3089	1, 1.5	2	+	150	0.16	63	87	1	4.40	5.88	3.46	3.36	3.38	5.04	3.88	3.80	3.82	5.20
3090	1, 1.5	2	-	150	0.16	87	63	1	6.52	5.22	6.86	6.78	6.84	5.20	6.74	6.68	6.72	4.90
3091	1, 1.5	2	+	150	0.33	50	100	1	2.92	5.06	2.38	2.34	2.36	5.14	2.52	2.46	2.48	5.20
3092	1, 1.5	2	-	150	0.33	100	50	1	7.80	4.86	9.48	9.28	9.40	4.76	9.18	9.04	9.12	4.94
3093	1, 1.5	2	+	150	0.50	37	113	1	2.08	5.52	1.68	1.68	1.68	5.60	1.50	1.48	1.48	5.04
3094	1, 1.5	2	-	150	0.50	113	37	1	9.70	5.50	12.42	12.24	12.32	5.04	11.86	11.70	11.80	5.58
3095	1, 1.5	2	0	200	0	100	100	1	5.14	5.22	5.08	5.04	5.06	5.20	5.68	5.62	5.64	5.66
3096	1, 1.5	2	+	200	0.16	84	116	1	3.76	5.20	3.72	3.64	3.70	5.08	4.14	4.12	4.12	5.36
3097	1, 1.5	2	-	200	0.16	116	84	1	6.68	5.14	7.02	6.90	7.00	5.20	7.04	6.96	6.98	5.22
3098	1, 1.5	2	+	200	0.33	67	133	1	3.00	5.12	2.66	2.60	2.64	5.14	2.66	2.60	2.62	5.50
3099	1, 1.5	2	-	200	0.33	133	67	1	7.84	5.10	9.14	9.04	9.08	4.76	9.64	9.52	9.58	5.82
3100	1, 1.5	2	+	200	0.50	50	150	1	2.14	5.18	1.74	1.72	1.74	5.22	1.38	1.34	1.38	5.44
3101	1, 1.5	2	-	200	0.50	150	50	1	10.16	5.62	12.20	12.02	12.12	5.50	12.30	12.14	12.18	4.88
3102	1, 1.5	5	0	20	0	10	10	1	5.46	4.82	5.30	4.62	4.90	3.88	5.40	4.60	5.00	3.62

	D	VR	P	N	Δn	n1	n2	ε	F g	B-F g	F t	F-GG t	F-HF t	B-F t	F g x t	F-GG g x t	F-HF g x t	B-F g x t
3103	1, 1.5	5	+	20	0.16	8	12	1	3.36	4.44	2.12	1.82	1.98	3.80	2.26	1.82	2.04	3.76
3104	1, 1.5	5	-	20	0.16	12	8	1	9.82	5.76	10.40	8.94	9.46	4.08	10.36	8.74	9.38	3.94
3105	1, 1.5	5	+	20	0.33	7	13	1	2.54	4.40	1.40	1.12	1.30	3.04	1.32	1.10	1.18	3.22
3106	1, 1.5	5	-	20	0.33	13	7	1	12.26	6.84	13.94	12.26	12.82	4.18	14.16	12.76	13.40	4.22
3107	1, 1.5	5	+	20	0.50	5	15	1	1.28	4.68	.38	.26	.32	4.10	.22	.18	.18	3.96
3108	1, 1.5	5	-	20	0.50	15	5	1	18.18	10.24	22.72	20.60	21.50	6.46	23.20	20.82	21.90	6.36
3109	1, 1.5	5	0	40	0	20	20	1	5.58	4.82	4.96	4.40	4.62	4.04	4.88	4.52	4.72	4.14
3110	1, 1.5	5	+	40	0.16	17	23	1	3.44	4.56	2.74	2.56	2.62	4.06	2.70	2.48	2.58	4.04
3111	1, 1.5	5	-	40	0.16	23	17	1	8.06	5.18	9.00	8.44	8.64	4.46	8.82	8.08	8.34	4.66
3112	1, 1.5	5	+	40	0.33	13	27	1	1.46	4.20	.94	.80	.88	4.16	.94	.84	.90	4.64
3113	1, 1.5	5	-	40	0.33	27	13	1	12.20	5.80	15.60	14.54	14.94	4.74	14.78	13.78	14.26	4.40
3114	1, 1.5	5	+	40	0.50	10	30	1	.82	4.12	.36	.32	.34	4.68	.32	.26	.28	4.68
3115	1, 1.5	5	-	40	0.50	30	10	1	16.80	6.66	21.66	20.46	20.90	5.44	22.00	20.98	21.30	4.98
3116	1, 1.5	5	0	60	0	30	30	1	5.24	4.66	5.52	5.24	5.42	4.90	5.30	4.94	5.06	4.72
3117	1, 1.5	5	+	60	0.16	25	35	1	2.98	4.44	2.58	2.44	2.50	4.90	2.30	2.12	2.22	4.56
3118	1, 1.5	5	-	60	0.16	35	25	1	7.82	4.40	9.88	9.34	9.52	4.74	9.40	9.10	9.24	4.62
3119	1, 1.5	5	+	60	0.33	20	40	1	1.58	4.74	1.28	1.18	1.22	4.60	.96	.84	.92	4.88
3120	1, 1.5	5	-	60	0.33	40	20	1	11.86	5.00	15.62	14.84	15.22	4.22	14.92	14.32	14.46	4.00
3121	1, 1.5	5	+	60	0.50	15	45	1	.86	4.96	.32	.28	.28	4.84	.26	.24	.24	5.24
3122	1, 1.5	5	-	60	0.50	45	15	1	16.42	5.96	23.58	22.56	22.86	4.60	22.82	22.12	22.32	4.82
3123	1, 1.5	5	0	80	0	40	40	1	5.06	4.70	5.78	5.46	5.54	5.22	5.14	4.88	5.00	4.58
3124	1, 1.5	5	+	80	0.16	34	46	1	2.82	4.40	3.18	2.96	3.06	5.74	2.74	2.56	2.68	5.52
3125	1, 1.5	5	-	80	0.16	46	34	1	7.82	4.94	9.46	9.04	9.18	5.08	8.40	8.06	8.16	4.80
3126	1, 1.5	5	+	80	0.33	27	53	1	1.68	4.60	1.18	1.08	1.12	5.54	.96	.88	.90	4.52
3127	1, 1.5	5	-	80	0.33	53	27	1	11.16	5.32	15.70	15.00	15.24	5.20	15.20	14.70	14.98	4.66
3128	1, 1.5	5	+	80	0.50	20	60	1	.76	4.90	.38	.36	.36	5.82	.26	.22	.24	4.94
3129	1, 1.5	5	-	80	0.50	60	20	1	16.08	5.80	23.86	23.24	23.50	5.38	23.32	22.76	22.92	5.26
3130	1, 1.5	5	0	100	0	50	50	1	5.22	4.96	5.18	5.00	5.10	4.76	5.16	4.96	5.02	4.86
3131	1, 1.5	5	+	100	0.16	42	58	1	2.86	4.68	2.42	2.30	2.34	4.86	2.54	2.40	2.50	4.34
3132	1, 1.5	5	-	100	0.16	58	42	1	8.30	5.40	8.80	8.48	8.60	5.12	9.18	8.98	9.06	4.74
3133	1, 1.5	5	+	100	0.33	33	67	1	1.26	4.52	.84	.80	.82	5.06	.96	.94	.96	4.88
3134	1, 1.5	5	-	100	0.33	67	33	1	12.60	5.84	15.84	15.30	15.44	5.48	16.02	15.42	15.66	5.30

	D	VR	P	N	Δn	n1	n2	ε	F g	B-F g	F t	F-GG t	F-HF t	B-F t	F g x t	F-GG g x t	F-HF g x t	B-F g x t
3135	1, 1.5	5	+	100	0.50	25	75	1	.58	4.82	.32	.30	.30	5.20	.24	.24	.24	4.54
3136	1, 1.5	5	-	100	0.50	75	25	1	17.90	6.24	22.78	22.00	22.28	4.80	22.78	22.12	22.30	5.10
3137	1, 1.5	5	0	150	0	75	75	1	5.56	5.36	5.06	4.96	5.02	5.08	5.00	4.88	4.94	5.04
3138	1, 1.5	5	+	150	0.16	63	87	1	3.40	5.36	2.60	2.50	2.50	4.86	2.72	2.70	2.72	5.00
3139	1, 1.5	5	-	150	0.16	87	63	1	7.96	5.24	9.20	9.04	9.10	4.94	8.92	8.64	8.80	5.00
3140	1, 1.5	5	+	150	0.33	50	100	1	1.58	4.96	1.00	.96	.98	5.00	.96	.94	.94	5.16
3141	1, 1.5	5	-	150	0.33	100	50	1	11.92	4.86	15.36	15.02	15.14	4.92	15.56	15.30	15.40	4.78
3142	1, 1.5	5	+	150	0.50	37	113	1	.52	5.08	.34	.34	.34	5.48	.30	.28	.28	4.72
3143	1, 1.5	5	-	150	0.50	113	37	1	17.08	5.92	23.22	22.86	22.96	4.78	22.80	22.38	22.46	5.50
3144	1, 1.5	5	0	200	0	100	100	1	5.02	5.06	5.32	5.24	5.28	5.26	5.58	5.40	5.48	5.42
3145	1, 1.5	5	+	200	0.16	84	116	1	2.88	4.98	2.42	2.38	2.40	5.30	2.82	2.74	2.78	5.42
3146	1, 1.5	5	-	200	0.16	116	84	1	8.16	5.22	9.12	8.98	9.10	5.14	9.34	9.14	9.20	5.32
3147	1, 1.5	5	+	200	0.33	67	133	1	1.66	4.90	1.04	1.04	1.04	5.18	.98	.96	.98	5.46
3148	1, 1.5	5	-	200	0.33	133	67	1	11.82	5.24	15.34	15.02	15.14	5.04	15.38	15.12	15.24	5.70
3149	1, 1.5	5	+	200	0.50	50	150	1	.60	4.62	.32	.30	.32	5.04	.18	.18	.18	5.12
3150	1, 1.5	5	-	200	0.50	150	50	1	17.26	5.50	22.70	22.44	22.56	5.52	23.28	23.02	23.16	4.86
3151	1.41, 3	1	0	20	0	10	10	.50	4.04	3.34	10.12	6.62	6.62	5.54	8.10	4.32	4.34	3.54
3152	1.41, 3	1	0	20	0.16	8	12	.50	4.94	4.36	10.26	6.22	6.22	5.46	8.64	4.98	4.98	4.24
3153	1.41, 3	1	0	20	0.33	7	13	.50	4.64	4.70	10.22	6.40	6.40	6.06	8.30	4.60	4.64	4.62
3154	1.41, 3	1	0	20	0.50	5	15	.50	4.68	6.74	9.78	5.92	5.94	8.02	8.42	4.70	4.70	6.66
3155	1.41, 3	1	0	40	0	20	20	.50	4.58	4.16	9.46	6.10	6.12	5.38	8.12	4.64	4.64	4.16
3156	1.41, 3	1	0	40	0.16	17	23	.50	4.70	4.16	9.62	5.92	5.92	5.48	8.20	4.46	4.46	4.00
3157	1.41, 3	1	0	40	0.33	13	27	.50	4.62	5.02	9.58	5.88	5.88	6.10	8.32	4.46	4.46	4.78
3158	1.41, 3	1	0	40	0.50	10	30	.50	4.54	5.72	9.34	5.60	5.60	6.64	8.38	4.48	4.50	6.16
3159	1.41, 3	1	0	60	0	30	30	.50	4.72	4.50	9.34	6.08	6.08	5.36	8.20	4.92	4.92	4.62
3160	1.41, 3	1	0	60	0.16	25	35	.50	4.72	4.82	9.20	6.14	6.14	5.40	7.76	4.92	4.92	4.70
3161	1.41, 3	1	0	60	0.33	20	40	.50	4.82	5.24	9.54	5.86	5.86	5.96	8.14	4.88	4.88	5.14
3162	1.41, 3	1	0	60	0.50	15	45	.50	4.80	5.92	9.54	5.58	5.58	6.76	8.26	4.80	4.80	6.24
3163	1.41, 3	1	0	80	0	40	40	.50	5.16	5.18	8.76	5.64	5.64	5.22	8.60	5.16	5.16	5.02
3164	1.41, 3	1	0	80	0.16	34	46	.50	4.86	4.58	9.04	5.52	5.52	5.16	7.96	5.10	5.10	4.70
3165	1.41, 3	1	0	80	0.33	27	53	.50	4.94	5.42	9.18	5.44	5.44	5.60	7.94	4.94	4.94	5.06
3166	1.41, 3	1	0	80	0.50	20	60	.50	4.70	5.66	9.48	5.68	5.68	6.62	7.94	4.72	4.72	5.86

	D	VR	P	N	Δn	n1	n2	ε	F g	B-F g	F t	F-GG t	F-HF t	B-F t	F g x t	F-GG g x t	F-HF g x t	B-F g x t
3167	1.41, 3	1	0	100	0	50	50	.50	5.30	5.22	9.02	5.26	5.26	5.06	8.60	5.14	5.14	5.18
3168	1.41, 3	1	0	100	0.16	42	58	.50	4.70	4.80	8.94	5.12	5.12	5.14	8.26	4.90	4.90	4.86
3169	1.41, 3	1	0	100	0.33	33	67	.50	5.08	4.94	9.04	5.22	5.22	5.30	8.04	4.90	4.90	5.10
3170	1.41, 3	1	0	100	0.50	25	75	.50	4.48	5.52	9.04	5.54	5.54	6.00	7.76	4.50	4.50	5.52
3171	1.41, 3	1	0	150	0	75	75	.50	5.40	5.36	7.84	4.74	4.74	4.68	8.58	5.38	5.38	5.36
3172	1.41, 3	1	0	150	0.16	63	87	.50	5.14	5.48	8.14	5.08	5.08	5.00	8.84	5.16	5.16	5.38
3173	1.41, 3	1	0	150	0.33	50	100	.50	5.20	5.52	8.46	5.14	5.14	5.30	8.50	5.24	5.26	5.46
3174	1.41, 3	1	0	150	0.50	37	113	.50	4.80	5.60	8.86	5.08	5.10	5.60	8.08	4.96	4.96	5.52
3175	1.41, 3	1	0	200	0	100	100	.50	5.24	5.30	8.34	5.10	5.10	4.90	8.06	5.04	5.04	5.08
3176	1.41, 3	1	0	200	0.16	84	116	.50	5.10	5.18	8.54	5.18	5.18	5.06	8.62	5.10	5.10	5.38
3177	1.41, 3	1	0	200	0.33	67	133	.50	5.30	5.36	8.68	5.18	5.18	5.02	9.06	5.30	5.30	5.50
3178	1.41, 3	1	0	200	0.50	50	150	.50	5.26	5.82	8.96	5.22	5.22	5.68	8.62	5.10	5.10	5.82
3179	1.41, 3	1.5	0	20	0	10	10	.50	4.62	3.86	10.20	6.46	6.48	5.48	8.58	4.58	4.60	3.82
3180	1.41, 3	1.5	+	20	0.16	8	12	.50	4.30	4.08	9.14	5.68	5.68	5.34	7.64	4.32	4.32	4.02
3181	1.41, 3	1.5	-	20	0.16	12	8	.50	5.28	4.26	11.72	7.64	7.64	5.70	9.38	5.28	5.30	4.48
3182	1.41, 3	1.5	+	20	0.33	7	13	.50	3.64	3.62	8.58	5.30	5.30	5.20	6.54	3.68	3.68	3.68
3183	1.41, 3	1.5	-	20	0.33	13	7	.50	5.74	5.28	12.34	7.96	7.96	6.48	10.76	5.82	5.88	5.56
3184	1.41, 3	1.5	+	20	0.50	5	15	.50	3.46	5.72	6.96	4.24	4.26	7.14	5.92	3.46	3.48	5.88
3185	1.41, 3	1.5	-	20	0.50	15	5	.50	7.34	8.30	13.32	8.50	8.50	8.82	12.10	7.36	7.38	8.58
3186	1.41, 3	1.5	0	40	0	20	20	.50	4.78	4.28	9.40	6.10	6.12	5.22	8.56	4.76	4.76	4.32
3187	1.41, 3	1.5	+	40	0.16	17	23	.50	4.40	4.50	8.60	5.38	5.38	5.52	7.74	4.18	4.18	4.22
3188	1.41, 3	1.5	-	40	0.16	23	17	.50	5.48	4.98	10.14	6.54	6.54	5.36	9.26	5.92	5.92	5.20
3189	1.41, 3	1.5	+	40	0.33	13	27	.50	3.38	4.42	7.50	4.66	4.68	5.48	6.34	3.32	3.32	4.46
3190	1.41, 3	1.5	-	40	0.33	27	13	.50	5.80	5.36	11.14	7.34	7.34	5.90	9.90	5.86	5.86	5.50
3191	1.41, 3	1.5	+	40	0.50	10	30	.50	3.00	5.20	6.40	3.60	3.60	6.20	5.64	3.08	3.08	5.00
3192	1.41, 3	1.5	-	40	0.50	30	10	.50	7.12	6.68	12.88	8.18	8.18	7.12	11.48	7.28	7.28	6.88
3193	1.41, 3	1.5	0	60	0	30	30	.50	4.90	4.58	9.34	5.86	5.86	5.32	8.22	5.02	5.02	4.66
3194	1.41, 3	1.5	+	60	0.16	25	35	.50	4.20	4.46	8.44	5.28	5.28	5.32	7.26	4.34	4.34	4.68
3195	1.41, 3	1.5	-	60	0.16	35	25	.50	6.00	5.28	10.38	6.82	6.84	5.68	9.82	5.92	5.92	5.36
3196	1.41, 3	1.5	+	60	0.33	20	40	.50	3.70	4.88	7.38	4.64	4.64	5.68	6.62	3.80	3.80	5.04
3197	1.41, 3	1.5	-	60	0.33	40	20	.50	7.10	6.04	11.32	7.26	7.26	5.62	10.94	7.04	7.04	5.98
3198	1.41, 3	1.5	+	60	0.50	15	45	.50	3.12	5.54	6.38	3.54	3.54	6.20	5.66	3.14	3.14	5.58

	D	VR	P	N	Δn	n1	n2	ε	F g	B-F g	F t	F-GG t	F-HF t	B-F t	F g x t	F-GG g x t	F-HF g x t	B-F g x t
3199	1.41, 3	1.5	-	60	0.50	45	15	.50	8.00	6.54	12.56	8.36	8.38	6.66	12.08	8.04	8.04	6.74
3200	1.41, 3	1.5	0	80	0	40	40	.50	5.18	4.90	8.68	5.68	5.68	5.20	8.52	4.96	4.96	4.78
3201	1.41, 3	1.5	+	80	0.16	34	46	.50	4.50	4.84	7.82	4.88	4.88	5.48	7.44	4.28	4.28	4.62
3202	1.41, 3	1.5	-	80	0.16	46	34	.50	5.60	4.94	10.06	6.34	6.34	5.24	9.76	5.88	5.92	5.26
3203	1.41, 3	1.5	+	80	0.33	27	53	.50	3.86	5.04	7.06	4.08	4.08	5.34	6.36	3.68	3.68	4.96
3204	1.41, 3	1.5	-	80	0.33	53	27	.50	6.84	5.44	11.56	7.28	7.28	5.48	10.72	6.88	6.88	5.72
3205	1.41, 3	1.5	+	80	0.50	20	60	.50	2.96	5.04	6.38	3.60	3.60	6.32	5.38	2.74	2.76	5.12
3206	1.41, 3	1.5	-	80	0.50	60	20	.50	7.98	6.88	12.72	8.40	8.40	6.74	12.62	8.14	8.14	6.74
3207	1.41, 3	1.5	0	100	0	50	50	.50	5.12	4.90	8.94	5.40	5.40	5.10	8.44	5.08	5.08	4.90
3208	1.41, 3	1.5	+	100	0.16	42	58	.50	4.14	4.72	7.94	4.72	4.72	5.14	7.34	4.04	4.04	4.74
3209	1.41, 3	1.5	-	100	0.16	58	42	.50	6.04	5.30	9.58	6.20	6.20	5.40	9.78	6.02	6.02	5.12
3210	1.41, 3	1.5	+	100	0.33	33	67	.50	3.66	4.74	6.86	3.80	3.80	5.22	6.10	3.46	3.46	4.74
3211	1.41, 3	1.5	-	100	0.33	67	33	.50	7.38	6.16	11.02	6.86	6.86	5.74	11.68	7.58	7.58	6.16
3212	1.41, 3	1.5	+	100	0.50	25	75	.50	2.86	5.12	6.22	3.42	3.42	5.82	5.04	2.78	2.78	5.04
3213	1.41, 3	1.5	-	100	0.50	75	25	.50	7.90	6.42	12.68	8.48	8.50	6.30	12.74	7.98	7.98	6.44
3214	1.41, 3	1.5	0	150	0	75	75	.50	5.22	5.08	7.90	4.62	4.62	4.52	8.82	5.36	5.36	5.30
3215	1.41, 3	1.5	+	150	0.16	63	87	.50	4.56	5.28	7.06	4.10	4.10	4.64	7.70	4.44	4.44	5.26
3216	1.41, 3	1.5	-	150	0.16	87	63	.50	6.26	5.26	8.58	5.30	5.30	4.48	9.92	6.12	6.12	5.44
3217	1.41, 3	1.5	+	150	0.33	50	100	.50	3.78	5.32	6.56	3.68	3.68	5.12	6.90	3.76	3.76	5.32
3218	1.41, 3	1.5	-	150	0.33	100	50	.50	6.88	5.26	9.96	6.28	6.28	4.94	10.82	7.04	7.04	5.36
3219	1.41, 3	1.5	+	150	0.50	37	113	.50	2.86	5.68	5.74	3.10	3.10	5.34	5.54	3.26	3.26	5.54
3220	1.41, 3	1.5	-	150	0.50	113	37	.50	7.18	5.22	11.30	7.32	7.32	5.02	11.40	7.30	7.30	5.24
3221	1.41, 3	1.5	0	200	0	100	100	.50	5.42	5.56	8.42	5.24	5.24	5.02	8.46	5.34	5.34	5.32
3222	1.41, 3	1.5	+	200	0.16	84	116	.50	4.46	5.00	7.42	4.50	4.50	5.00	7.72	4.56	4.56	5.02
3223	1.41, 3	1.5	-	200	0.16	116	84	.50	5.78	5.04	9.36	5.72	5.72	4.84	9.30	5.64	5.64	5.06
3224	1.41, 3	1.5	+	200	0.33	67	133	.50	3.72	5.36	6.62	3.64	3.64	4.80	6.74	3.92	3.92	5.24
3225	1.41, 3	1.5	-	200	0.33	133	67	.50	6.90	5.76	10.58	6.96	6.96	5.68	10.74	6.66	6.68	5.48
3226	1.41, 3	1.5	+	200	0.50	50	150	.50	2.88	5.50	6.02	2.90	2.90	5.78	5.90	2.92	2.92	5.40
3227	1.41, 3	1.5	-	200	0.50	150	50	.50	7.66	6.04	11.90	8.00	8.00	5.44	12.44	7.92	7.92	5.98
3228	1.41, 3	2	0	20	0	10	10	.50	5.06	4.28	10.44	6.56	6.56	5.50	8.78	5.12	5.12	4.38
3229	1.41, 3	2	+	20	0.16	8	12	.50	4.16	4.16	8.66	5.20	5.20	5.22	7.56	4.14	4.14	4.24
3230	1.41, 3	2	-	20	0.16	12	8	.50	6.25	5.01	12.88	8.49	8.57	6.07	10.66	6.17	6.19	5.11

	D	VR	P	N	Δn	n1	n2	ε	F g	B-F g	F t	F-GG t	F-HF t	B-F t	F g x t	F-GG g x t	F-HF g x t	B-F g x t
3231	1.41, 3	2	+	20	0.33	7	13	.50	3.40	3.30	7.64	4.62	4.68	5.04	5.92	3.48	3.50	3.40
3232	1.41, 3	2	-	20	0.33	13	7	.50	7.34	5.94	13.82	9.14	9.14	6.88	12.50	7.62	7.64	6.02
3233	1.41, 3	2	+	20	0.50	5	15	.50	2.92	5.24	6.00	3.40	3.40	6.50	4.92	3.00	3.00	5.18
3234	1.41, 3	2	-	20	0.50	15	5	.50	9.64	9.04	15.94	10.82	10.84	9.10	15.10	9.62	9.62	9.32
3235	1.41, 3	2	0	40	0	20	20	.50	5.08	4.74	9.48	6.04	6.04	5.26	8.82	5.18	5.18	4.78
3236	1.41, 3	2	+	40	0.16	17	23	.50	4.26	4.76	8.36	5.00	5.02	5.42	7.34	4.06	4.06	4.66
3237	1.41, 3	2	-	40	0.16	23	17	.50	6.30	5.42	10.84	7.04	7.06	5.82	10.18	6.60	6.62	5.42
3238	1.41, 3	2	+	40	0.33	13	27	.50	2.80	4.28	6.38	3.66	3.66	5.28	5.40	2.96	2.98	4.10
3239	1.41, 3	2	-	40	0.33	27	13	.50	7.28	5.80	13.06	8.78	8.78	6.18	12.20	7.08	7.10	5.74
3240	1.41, 3	2	+	40	0.50	10	30	.50	2.22	4.88	4.92	2.66	2.66	5.88	4.44	2.26	2.26	4.76
3241	1.41, 3	2	-	40	0.50	30	10	.50	9.24	7.20	15.30	10.50	10.50	7.34	14.22	9.38	9.40	7.36
3242	1.41, 3	2	0	60	0	30	30	.50	5.12	4.82	9.20	5.96	5.96	5.38	8.38	4.88	4.90	4.68
3243	1.41, 3	2	+	60	0.16	25	35	.50	3.86	4.46	7.88	4.64	4.64	5.16	6.90	3.82	3.82	4.70
3244	1.41, 3	2	-	60	0.16	35	25	.50	6.62	5.68	11.14	7.30	7.30	5.78	10.38	6.68	6.68	5.52
3245	1.41, 3	2	+	60	0.33	20	40	.50	3.22	4.80	6.34	3.62	3.64	5.58	5.60	3.24	3.24	5.00
3246	1.41, 3	2	-	60	0.33	40	20	.50	8.26	6.22	13.20	8.46	8.46	5.82	12.76	8.52	8.52	6.14
3247	1.41, 3	2	+	60	0.50	15	45	.50	2.26	5.16	4.90	2.58	2.58	6.08	4.24	2.40	2.40	5.04
3248	1.41, 3	2	-	60	0.50	45	15	.50	10.48	6.98	15.48	10.46	10.46	6.92	14.84	10.06	10.06	7.04
3249	1.41, 3	2	0	80	0	40	40	.50	5.02	4.68	8.80	5.56	5.58	5.00	8.64	4.88	4.88	4.74
3250	1.41, 3	2	+	80	0.16	34	46	.50	4.22	4.80	7.48	4.44	4.44	5.38	7.00	3.92	3.94	4.74
3251	1.41, 3	2	-	80	0.16	46	34	.50	6.30	5.06	10.72	6.92	6.92	5.40	10.54	6.32	6.32	5.28
3252	1.41, 3	2	+	80	0.33	27	53	.50	3.18	5.00	6.02	3.42	3.42	5.18	5.40	2.90	2.90	4.82
3253	1.41, 3	2	-	80	0.33	53	27	.50	8.00	5.44	13.28	8.74	8.74	5.66	12.36	8.02	8.02	5.70
3254	1.41, 3	2	+	80	0.50	20	60	.50	2.08	5.12	4.66	2.48	2.48	5.90	3.98	2.06	2.06	4.92
3255	1.41, 3	2	-	80	0.50	60	20	.50	10.58	7.06	15.38	10.56	10.56	6.94	15.68	10.34	10.34	7.16
3256	1.41, 3	2	0	100	0	50	50	.50	5.06	4.96	8.86	5.48	5.48	4.76	8.44	4.94	4.94	4.98
3257	1.41, 3	2	+	100	0.16	42	58	.50	3.82	4.64	7.54	4.18	4.18	4.96	6.60	3.88	3.88	4.70
3258	1.41, 3	2	-	100	0.16	58	42	.50	6.74	5.42	10.18	6.72	6.72	5.50	10.50	6.68	6.68	5.34
3259	1.41, 3	2	+	100	0.33	33	67	.50	2.96	4.70	5.66	3.20	3.20	5.02	5.08	2.78	2.78	4.56
3260	1.41, 3	2	-	100	0.33	67	33	.50	8.98	6.04	12.78	8.50	8.50	5.34	13.46	8.94	8.94	6.08
3261	1.41, 3	2	+	100	0.50	25	75	.50	2.02	4.78	4.56	2.32	2.32	5.74	3.80	2.00	2.00	4.96
3262	1.41, 3	2	-	100	0.50	75	25	.50	10.30	6.54	15.10	10.66	10.66	6.38	15.30	10.26	10.28	6.68

	D	VR	P	N	Δn	n1	n2	ε	F g	B-F g	F t	F-GG t	F-HF t	B-F t	F g x t	F-GG g x t	F-HF g x t	B-F g x t
3263	1.41, 3	2	0	150	0	75	75	.50	5.26	5.18	7.70	4.66	4.66	4.54	8.98	5.38	5.38	5.24
3264	1.41, 3	2	+	150	0.16	63	87	.50	4.06	5.24	6.26	3.64	3.64	4.50	7.22	4.20	4.20	5.20
3265	1.41, 3	2	-	150	0.16	87	63	.50	6.66	5.12	9.38	5.98	5.98	4.68	10.82	6.72	6.72	5.18
3266	1.41, 3	2	+	150	0.33	50	100	.50	2.96	5.18	5.14	2.80	2.80	4.98	5.74	2.88	2.88	5.32
3267	1.41, 3	2	-	150	0.33	100	50	.50	8.00	5.24	11.56	7.44	7.44	4.82	12.14	8.18	8.18	5.26
3268	1.41, 3	2	+	150	0.50	37	113	.50	2.12	5.38	4.26	2.36	2.36	5.20	4.04	2.24	2.24	5.42
3269	1.41, 3	2	-	150	0.50	113	37	.50	9.36	5.20	14.02	9.50	9.50	5.30	13.70	9.34	9.34	5.16
3270	1.41, 3	2	0	200	0	100	100	.50	5.42	5.58	8.48	5.14	5.14	5.00	8.64	5.46	5.46	5.54
3271	1.41, 3	2	+	200	0.16	84	116	.50	4.18	5.06	6.90	3.98	3.98	4.94	7.04	3.96	3.96	5.14
3272	1.41, 3	2	-	200	0.16	116	84	.50	6.40	5.04	10.28	6.52	6.52	4.94	10.22	6.20	6.20	5.20
3273	1.41, 3	2	+	200	0.33	67	133	.50	2.94	5.08	5.60	2.78	2.78	4.74	5.52	2.98	2.98	5.16
3274	1.41, 3	2	-	200	0.33	133	67	.50	8.36	5.78	12.18	8.02	8.02	5.42	12.46	8.06	8.06	5.66
3275	1.41, 3	2	+	200	0.50	50	150	.50	1.94	5.16	4.26	1.96	1.96	5.34	4.14	2.04	2.04	5.10
3276	1.41, 3	2	-	200	0.50	150	50	.50	10.22	5.96	14.78	10.44	10.44	5.86	14.96	10.52	10.52	6.12
3277	1.41, 3	5	0	20	0	10	10	.50	6.38	5.36	11.34	7.52	7.54	5.84	10.40	6.56	6.56	5.66
3278	1.41, 3	5	+	20	0.16	8	12	.50	4.34	5.14	7.44	4.44	4.44	5.48	7.06	4.28	4.28	5.08
3279	1.41, 3	5	-	20	0.16	12	8	.50	10.46	6.76	16.24	11.16	11.16	7.22	15.22	10.38	10.38	6.80
3280	1.41, 3	5	+	20	0.33	7	13	.50	3.22	4.52	5.80	3.30	3.34	5.24	5.00	3.36	3.36	4.70
3281	1.41, 3	5	-	20	0.33	13	7	.50	12.70	8.06	18.62	13.76	13.80	8.28	18.64	12.50	12.58	7.98
3282	1.41, 3	5	+	20	0.50	5	15	.50	1.90	4.42	3.16	1.74	1.74	5.60	3.26	2.06	2.06	4.56
3283	1.41, 3	5	-	20	0.50	15	5	.50	18.74	11.26	25.24	18.56	18.60	10.62	24.96	18.42	18.44	11.16
3284	1.41, 3	5	0	40	0	20	20	.50	5.98	5.30	9.90	6.22	6.24	5.06	9.52	5.94	5.94	5.26
3285	1.41, 3	5	+	40	0.16	17	23	.50	4.04	5.00	7.12	4.20	4.20	4.98	7.12	3.96	3.98	4.80
3286	1.41, 3	5	-	40	0.16	23	17	.50	8.52	6.04	13.06	8.92	8.92	5.84	13.10	8.62	8.64	6.20
3287	1.41, 3	5	+	40	0.33	13	27	.50	2.14	4.36	4.12	2.20	2.20	5.00	3.74	2.04	2.04	4.28
3288	1.41, 3	5	-	40	0.33	27	13	.50	12.62	6.92	18.96	13.44	13.50	6.50	18.12	12.90	12.92	7.22
3289	1.41, 3	5	+	40	0.50	10	30	.50	1.22	4.34	2.28	1.24	1.24	5.40	2.24	1.16	1.16	4.10
3290	1.41, 3	5	-	40	0.50	30	10	.50	17.44	8.40	23.84	18.04	18.08	8.54	22.84	17.16	17.16	8.70
3291	1.41, 3	5	0	60	0	30	30	.50	5.56	4.94	9.80	6.26	6.26	5.30	8.76	5.34	5.34	4.80
3292	1.41, 3	5	+	60	0.16	25	35	.50	3.28	4.76	6.50	3.86	3.86	5.16	5.74	3.18	3.18	4.84
3293	1.41, 3	5	-	60	0.16	35	25	.50	8.60	6.24	13.48	9.24	9.24	5.98	12.84	8.70	8.70	6.26
3294	1.41, 3	5	+	60	0.33	20	40	.50	2.14	4.60	4.08	2.24	2.24	5.16	3.90	2.06	2.06	4.70

	D	VR	P	N	Δn	n1	n2	ε	F g	B-F g	F t	F-GG t	F-HF t	B-F t	F g x t	F-GG g x t	F-HF g x t	B-F g x t
3295	1.41, 3	5	-	60	0.33	40	20	.50	12.52	6.62	17.78	12.84	12.84	6.38	17.94	12.90	12.92	6.68
3296	1.41, 3	5	+	60	0.50	15	45	.50	1.26	4.70	2.22	.86	.86	5.30	2.08	1.16	1.16	4.70
3297	1.41, 3	5	-	60	0.50	45	15	.50	17.40	7.62	23.46	18.06	18.06	7.38	23.50	17.76	17.78	7.66
3298	1.41, 3	5	0	80	0	40	40	.50	5.38	5.02	9.12	5.68	5.68	4.98	8.96	5.50	5.50	4.94
3299	1.41, 3	5	+	80	0.16	34	46	.50	3.30	4.60	6.28	3.58	3.58	5.08	5.86	3.48	3.48	4.64
3300	1.41, 3	5	-	80	0.16	46	34	.50	8.34	5.14	13.40	8.66	8.66	5.44	13.06	8.42	8.42	5.20
3301	1.41, 3	5	+	80	0.33	27	53	.50	1.66	4.40	3.62	1.90	1.90	4.90	3.38	1.66	1.68	4.48
3302	1.41, 3	5	-	80	0.33	53	27	.50	12.38	5.70	17.72	12.82	12.82	5.88	17.56	12.58	12.58	5.64
3303	1.41, 3	5	+	80	0.50	20	60	.50	.90	4.64	1.86	.94	.94	5.34	1.68	.86	.86	4.34
3304	1.41, 3	5	-	80	0.50	60	20	.50	17.74	7.30	23.38	18.00	18.00	7.16	23.80	17.88	17.88	7.10
3305	1.41, 3	5	0	100	0	50	50	.50	5.22	4.78	8.80	5.64	5.64	4.98	8.56	5.16	5.16	4.70
3306	1.41, 3	5	+	100	0.16	42	58	.50	3.02	4.80	6.30	3.46	3.46	5.04	5.58	3.02	3.02	4.72
3307	1.41, 3	5	-	100	0.16	58	42	.50	8.86	5.76	12.92	8.22	8.22	5.68	13.24	8.34	8.36	5.54
3308	1.41, 3	5	+	100	0.33	33	67	.50	1.52	4.58	3.44	1.62	1.62	4.88	3.10	1.64	1.64	4.54
3309	1.41, 3	5	-	100	0.33	67	33	.50	13.06	6.28	17.76	12.96	12.96	5.48	17.94	13.06	13.06	6.08
3310	1.41, 3	5	+	100	0.50	25	75	.50	.64	4.38	1.68	.90	.90	4.86	1.54	.62	.62	4.16
3311	1.41, 3	5	-	100	0.50	75	25	.50	17.84	7.06	22.82	17.46	17.46	6.44	23.84	17.82	17.82	6.82
3312	1.41, 3	5	0	150	0	75	75	.50	5.48	4.98	8.04	4.94	4.94	4.80	9.08	5.52	5.52	5.28
3313	1.41, 3	5	+	150	0.16	63	87	.50	3.18	4.94	5.14	2.78	2.78	4.08	6.14	3.12	3.12	5.02
3314	1.41, 3	5	-	150	0.16	87	63	.50	8.46	5.20	11.60	8.06	8.06	4.78	12.82	8.56	8.56	5.12
3315	1.41, 3	5	+	150	0.33	50	100	.50	1.44	4.72	2.98	1.48	1.48	4.40	3.30	1.40	1.40	5.06
3316	1.41, 3	5	-	150	0.33	100	50	.50	11.84	5.02	16.28	11.36	11.36	4.82	16.66	11.72	11.72	5.12
3317	1.41, 3	5	+	150	0.50	37	113	.50	.74	4.90	1.44	.72	.72	4.76	1.78	.74	.74	4.98
3318	1.41, 3	5	-	150	0.50	113	37	.50	16.88	5.66	22.32	16.54	16.54	5.36	22.74	16.86	16.86	5.40
3319	1.41, 3	5	0	200	0	100	100	.50	5.50	5.16	8.60	5.28	5.28	5.16	9.32	5.34	5.34	5.14
3320	1.41, 3	5	+	200	0.16	84	116	.50	3.36	5.04	5.84	3.08	3.08	4.90	5.78	3.36	3.36	5.00
3321	1.41, 3	5	-	200	0.16	116	84	.50	8.16	5.32	12.48	8.12	8.12	5.18	11.96	8.02	8.02	5.34
3322	1.41, 3	5	+	200	0.33	67	133	.50	1.80	5.10	3.18	1.50	1.50	4.86	3.28	1.70	1.70	4.96
3323	1.41, 3	5	-	200	0.33	133	67	.50	12.42	5.78	16.84	11.96	11.96	5.40	16.92	12.22	12.22	5.76
3324	1.41, 3	5	+	200	0.50	50	150	.50	.80	4.82	1.50	.58	.58	4.96	1.72	.92	.92	4.82
3325	1.41, 3	5	-	200	0.50	150	50	.50	17.68	6.20	22.44	17.24	17.26	6.06	22.76	17.60	17.60	6.16
3326	1.41, 3	1	0	20	0	10	10	.60	4.02	3.52	8.20	6.18	6.34	5.84	7.36	4.56	4.62	4.04

	D	VR	P	N	Δn	n1	n2	ε	F g	B-F g	F t	F-GG t	F-HF t	B-F t	F g x t	F-GG g x t	F-HF g x t	B-F g x t
3327	1.41, 3	1	0	20	0.16	8	12	.60	4.62	4.14	8.44	6.08	6.24	5.80	6.98	4.48	4.60	4.00
3328	1.41, 3	1	0	20	0.33	7	13	.60	4.88	4.64	8.40	6.16	6.34	5.90	7.40	4.80	4.94	4.52
3329	1.41, 3	1	0	20	0.50	5	15	.60	4.60	7.08	8.42	5.84	6.00	8.28	7.08	4.34	4.62	7.12
3330	1.41, 3	1	0	40	0	20	20	.60	5.12	4.86	8.10	5.58	5.72	5.22	7.60	5.06	5.06	4.80
3331	1.41, 3	1	0	40	0.16	17	23	.60	5.10	4.90	7.98	5.52	5.70	5.38	7.28	4.60	4.64	4.56
3332	1.41, 3	1	0	40	0.33	13	27	.60	4.54	5.02	8.26	5.38	5.52	5.72	7.90	4.98	5.08	5.42
3333	1.41, 3	1	0	40	0.50	10	30	.60	4.16	5.58	7.60	5.26	5.34	6.20	7.50	4.80	4.86	6.30
3334	1.41, 3	1	0	60	0	30	30	.60	4.80	4.92	7.76	5.30	5.32	4.90	7.36	4.90	4.90	4.82
3335	1.41, 3	1	0	60	0.16	25	35	.60	5.20	4.90	7.68	5.28	5.28	4.92	7.56	4.86	4.98	4.84
3336	1.41, 3	1	0	60	0.33	20	40	.60	5.16	5.58	7.84	5.26	5.32	5.50	7.34	5.04	5.08	5.36
3337	1.41, 3	1	0	60	0.50	15	45	.60	5.08	5.88	8.00	5.14	5.16	5.92	7.12	5.04	5.10	5.78
3338	1.41, 3	1	0	80	0	40	40	.60	4.60	4.64	8.72	5.86	5.96	5.72	6.70	4.44	4.46	4.60
3339	1.41, 3	1	0	80	0.16	34	46	.60	4.68	4.74	8.32	5.74	5.76	5.64	7.06	4.74	4.78	4.82
3340	1.41, 3	1	0	80	0.33	27	53	.60	5.18	5.24	7.94	5.26	5.28	5.70	6.90	4.34	4.38	4.92
3341	1.41, 3	1	0	80	0.50	20	60	.60	5.08	6.14	7.68	5.48	5.50	5.92	7.52	5.32	5.34	5.88
3342	1.41, 3	1	0	100	0	50	50	.60	5.10	5.04	8.18	5.80	5.84	5.58	7.24	4.54	4.56	4.34
3343	1.41, 3	1	0	100	0.16	42	58	.60	5.02	5.02	8.14	5.56	5.60	5.50	7.00	4.70	4.70	4.66
3344	1.41, 3	1	0	100	0.33	33	67	.60	4.74	5.22	7.74	5.32	5.34	5.56	6.94	4.42	4.44	4.82
3345	1.41, 3	1	0	100	0.50	25	75	.60	4.56	5.60	7.68	5.30	5.32	5.80	7.06	4.52	4.54	5.66
3346	1.41, 3	1	0	150	0	75	75	.60	5.54	5.60	8.42	5.74	5.74	5.74	7.72	5.54	5.58	5.58
3347	1.41, 3	1	0	150	0.16	63	87	.60	5.06	5.50	8.24	5.70	5.72	5.56	7.24	4.76	4.76	5.06
3348	1.41, 3	1	0	150	0.33	50	100	.60	5.02	5.28	8.30	5.66	5.68	5.70	7.20	4.70	4.70	5.22
3349	1.41, 3	1	0	150	0.50	37	113	.60	5.20	5.54	7.80	5.24	5.26	6.10	6.92	4.68	4.72	5.26
3350	1.41, 3	1	0	200	0	100	100	.60	5.20	5.22	8.12	5.76	5.78	5.82	7.74	5.10	5.14	5.16
3351	1.41, 3	1	0	200	0.16	84	116	.60	5.38	5.42	8.48	5.98	6.00	5.90	8.08	5.24	5.26	5.50
3352	1.41, 3	1	0	200	0.33	67	133	.60	5.02	4.96	8.46	5.90	5.94	5.82	7.70	5.42	5.42	5.28
3353	1.41, 3	1	0	200	0.50	50	150	.60	4.68	5.30	8.14	5.72	5.72	6.10	7.10	4.52	4.54	5.12
3354	1.41, 3	1.5	0	20	0	10	10	.60	4.14	3.70	8.40	6.14	6.30	5.68	7.52	4.84	5.06	4.16
3355	1.41, 3	1.5	+	20	0.16	8	12	.60	3.86	3.88	7.42	5.72	5.92	5.80	6.02	3.74	3.92	4.00
3356	1.41, 3	1.5	-	20	0.16	12	8	.60	5.54	4.70	9.90	7.06	7.26	6.18	9.12	6.10	6.38	4.94
3357	1.41, 3	1.5	+	20	0.33	7	13	.60	4.04	4.64	7.08	5.02	5.22	6.14	5.82	3.80	3.88	4.78
3358	1.41, 3	1.5	-	20	0.33	13	7	.60	5.94	5.52	10.40	7.60	7.74	6.84	9.84	6.62	6.76	5.60

	D	VR	P	N	Δn	n1	n2	ε	F g	B-F g	F t	F-GG t	F-HF t	B-F t	F g x t	F-GG g x t	F-HF g x t	B-F g x t
3359	1.41, 3	1.5	+	20	0.50	5	15	.60	3.14	5.70	5.94	4.20	4.30	7.26	4.58	2.90	2.98	5.88
3360	1.41, 3	1.5	-	20	0.50	15	5	.60	7.44	8.60	11.64	8.26	8.46	9.00	10.38	7.44	7.68	7.20
3361	1.41, 3	1.5	0	40	0	20	20	.60	5.04	4.88	8.04	5.58	5.62	5.26	7.66	5.06	5.08	4.76
3362	1.41, 3	1.5	+	40	0.16	17	23	.60	4.50	4.98	7.12	4.90	4.90	5.22	6.52	3.92	4.04	4.50
3363	1.41, 3	1.5	-	40	0.16	23	17	.60	5.94	5.08	8.78	6.30	6.40	5.46	8.26	5.50	5.60	5.10
3364	1.41, 3	1.5	+	40	0.33	13	27	.60	3.22	4.94	6.14	4.16	4.28	5.62	5.72	3.48	3.54	5.54
3365	1.41, 3	1.5	-	40	0.33	27	13	.60	6.58	5.82	10.18	7.44	7.50	6.22	9.34	6.26	6.30	5.58
3366	1.41, 3	1.5	+	40	0.50	10	30	.60	2.46	5.36	5.16	3.26	3.30	6.20	4.58	2.84	2.86	5.78
3367	1.41, 3	1.5	-	40	0.50	30	10	.60	7.50	6.92	10.74	8.08	8.16	7.08	10.22	7.42	7.48	6.82
3368	1.41, 3	1.5	0	60	0	30	30	.60	4.90	4.76	7.84	5.40	5.40	5.10	7.16	4.90	4.92	4.84
3369	1.41, 3	1.5	+	60	0.16	25	35	.60	4.38	4.74	6.68	4.56	4.60	5.14	6.74	4.24	4.34	4.66
3370	1.41, 3	1.5	-	60	0.16	35	25	.60	5.78	4.94	8.94	6.08	6.14	5.46	8.26	5.64	5.72	5.02
3371	1.41, 3	1.5	+	60	0.33	20	40	.60	3.58	5.26	5.94	3.78	3.84	5.04	5.68	3.86	3.88	5.20
3372	1.41, 3	1.5	-	60	0.33	40	20	.60	6.46	5.38	10.04	7.24	7.30	5.48	9.44	6.62	6.66	5.54
3373	1.41, 3	1.5	+	60	0.50	15	45	.60	3.22	5.42	4.94	3.24	3.24	5.60	5.04	2.98	3.04	5.46
3374	1.41, 3	1.5	-	60	0.50	45	15	.60	7.44	5.70	11.22	8.30	8.32	6.44	10.64	7.84	7.86	6.18
3375	1.41, 3	1.5	0	80	0	40	40	.60	4.70	4.56	8.78	5.92	6.06	5.74	6.92	4.90	4.94	4.94
3376	1.41, 3	1.5	+	80	0.16	34	46	.60	4.16	4.52	7.48	5.08	5.10	5.90	6.20	4.34	4.34	4.98
3377	1.41, 3	1.5	-	80	0.16	46	34	.60	5.54	4.78	10.08	7.24	7.28	6.16	7.76	5.34	5.38	5.00
3378	1.41, 3	1.5	+	80	0.33	27	53	.60	3.62	4.88	6.14	4.20	4.28	5.58	5.08	3.32	3.34	4.82
3379	1.41, 3	1.5	-	80	0.33	53	27	.60	6.26	4.86	11.20	8.00	8.10	6.26	9.10	6.18	6.20	4.86
3380	1.41, 3	1.5	+	80	0.50	20	60	.60	3.34	5.76	5.18	3.54	3.58	5.92	5.30	3.18	3.22	5.76
3381	1.41, 3	1.5	-	80	0.50	60	20	.60	7.20	5.78	12.24	9.00	9.04	6.86	10.50	7.24	7.26	5.66
3382	1.41, 3	1.5	0	100	0	50	50	.60	5.08	4.96	8.46	5.88	5.90	5.66	7.46	4.56	4.60	4.52
3383	1.41, 3	1.5	+	100	0.16	42	58	.60	4.38	4.90	7.26	5.00	5.02	5.44	6.30	4.16	4.18	4.90
3384	1.41, 3	1.5	-	100	0.16	58	42	.60	5.64	5.10	9.40	6.60	6.64	5.78	8.30	5.46	5.52	4.90
3385	1.41, 3	1.5	+	100	0.33	33	67	.60	3.38	5.10	5.86	4.04	4.06	5.44	5.24	3.44	3.46	4.92
3386	1.41, 3	1.5	-	100	0.33	67	33	.60	6.98	5.44	10.26	6.88	6.88	5.24	9.44	6.64	6.66	5.22
3387	1.41, 3	1.5	+	100	0.50	25	75	.60	2.66	5.38	5.08	3.40	3.44	5.50	4.52	2.82	2.84	5.32
3388	1.41, 3	1.5	-	100	0.50	75	25	.60	8.10	6.00	10.44	7.56	7.58	5.66	10.50	7.72	7.72	5.64
3389	1.41, 3	1.5	0	150	0	75	75	.60	5.42	5.42	8.50	5.72	5.72	5.60	7.88	5.70	5.72	5.66
3390	1.41, 3	1.5	+	150	0.16	63	87	.60	4.62	5.40	7.40	4.94	5.04	5.56	6.64	4.26	4.30	5.20

	D	VR	P	N	Δn	n1	n2	ε	F g	B-F g	F t	F-GG t	F-HF t	B-F t	F g x t	F-GG g x t	F-HF g x t	B-F g x t
3391	1.41, 3	1.5	-	150	0.16	87	63	.60	5.94	5.02	9.58	6.48	6.50	5.58	9.18	6.34	6.36	5.46
3392	1.41, 3	1.5	+	150	0.33	50	100	.60	3.70	5.22	6.34	4.18	4.20	5.58	5.48	3.46	3.46	5.28
3393	1.41, 3	1.5	-	150	0.33	100	50	.60	6.58	5.26	10.40	7.42	7.44	5.88	9.58	6.82	6.84	5.52
3394	1.41, 3	1.5	+	150	0.50	37	113	.60	3.08	5.42	5.24	3.56	3.58	5.90	4.30	2.74	2.78	5.08
3395	1.41, 3	1.5	-	150	0.50	113	37	.60	7.52	5.52	11.20	8.32	8.34	5.70	10.22	7.28	7.32	5.28
3396	1.41, 3	1.5	0	200	0	100	100	.60	5.16	5.18	8.02	5.84	5.84	5.66	7.56	5.28	5.28	5.02
3397	1.41, 3	1.5	+	200	0.16	84	116	.60	4.68	5.38	7.28	5.12	5.12	5.84	7.02	4.48	4.48	5.52
3398	1.41, 3	1.5	-	200	0.16	116	84	.60	5.80	5.14	8.92	6.24	6.24	5.40	8.46	5.72	5.72	5.22
3399	1.41, 3	1.5	+	200	0.33	67	133	.60	3.56	4.84	6.84	4.20	4.22	5.94	5.86	3.92	3.94	5.30
3400	1.41, 3	1.5	-	200	0.33	133	67	.60	6.86	5.32	10.42	7.56	7.58	5.68	10.58	7.22	7.26	5.68
3401	1.41, 3	1.5	+	200	0.50	50	150	.60	2.82	5.12	5.70	3.56	3.58	6.06	4.50	2.64	2.66	4.84
3402	1.41, 3	1.5	-	200	0.50	150	50	.60	7.76	5.46	11.86	8.80	8.86	6.18	11.22	8.08	8.10	5.82
3403	1.41, 3	2	0	20	0	10	10	.60	4.34	3.70	8.80	6.22	6.46	5.58	8.00	5.04	5.22	4.68
3404	1.41, 3	2	+	20	0.16	8	12	.60	3.64	3.94	6.90	5.34	5.60	5.84	5.56	3.56	3.74	4.24
3405	1.41, 3	2	-	20	0.16	12	8	.60	6.38	4.92	10.92	8.12	8.30	6.60	10.02	7.02	7.12	5.36
3406	1.41, 3	2	+	20	0.33	7	13	.60	3.34	4.56	6.26	4.56	4.78	5.96	4.94	3.10	3.26	4.36
3407	1.41, 3	2	-	20	0.33	13	7	.60	7.16	6.14	11.76	8.88	9.16	7.20	11.48	7.92	8.12	5.92
3408	1.41, 3	2	+	20	0.50	5	15	.60	2.56	5.14	4.64	3.22	3.34	6.76	3.40	2.28	2.30	5.34
3409	1.41, 3	2	-	20	0.50	15	5	.60	9.76	9.50	14.54	10.68	11.02	9.58	13.48	9.52	9.70	8.12
3410	1.41, 3	2	0	40	0	20	20	.60	5.16	4.80	7.98	5.74	5.78	5.64	7.60	5.22	5.28	4.90
3411	1.41, 3	2	+	40	0.16	17	23	.60	4.06	4.92	6.60	4.68	4.78	5.32	6.14	3.74	3.82	4.60
3412	1.41, 3	2	-	40	0.16	23	17	.60	6.36	5.24	9.34	6.90	7.02	5.60	8.78	6.06	6.08	5.32
3413	1.41, 3	2	+	40	0.33	13	27	.60	2.58	4.60	5.10	3.32	3.40	5.56	4.56	2.78	2.82	5.14
3414	1.41, 3	2	-	40	0.33	27	13	.60	8.14	6.20	11.70	8.64	8.72	6.38	10.98	7.78	7.90	5.90
3415	1.41, 3	2	+	40	0.50	10	30	.60	1.98	4.90	3.68	2.40	2.46	5.98	3.22	2.12	2.14	5.38
3416	1.41, 3	2	-	40	0.50	30	10	.60	9.98	7.28	13.38	10.02	10.14	7.34	13.16	9.64	9.72	7.46
3417	1.41, 3	2	0	60	0	30	30	.60	4.72	4.60	7.64	5.54	5.56	5.06	7.14	4.94	4.96	4.84
3418	1.41, 3	2	+	60	0.16	25	35	.60	4.08	4.76	6.06	4.38	4.40	5.02	6.36	3.98	4.06	4.84
3419	1.41, 3	2	-	60	0.16	35	25	.60	6.28	5.10	9.62	6.86	6.90	5.58	9.30	6.20	6.28	5.26
3420	1.41, 3	2	+	60	0.33	20	40	.60	2.98	5.12	4.88	3.14	3.20	5.04	5.02	3.44	3.48	5.12
3421	1.41, 3	2	-	60	0.33	40	20	.60	7.90	5.72	11.86	8.62	8.72	5.44	11.32	8.18	8.18	5.60
3422	1.41, 3	2	+	60	0.50	15	45	.60	2.20	5.22	3.72	2.18	2.22	5.52	3.70	1.96	2.02	5.26

	D	VR	P	N	Δn	n1	n2	ε	F g	B-F g	F t	F-GG t	F-HF t	B-F t	F g x t	F-GG g x t	F-HF g x t	B-F g x t
3423	1.41, 3	2	-	60	0.50	45	15	.60	9.66	6.06	13.62	10.56	10.64	6.82	13.16	9.64	9.70	6.26
3424	1.41, 3	2	0	80	0	40	40	.60	4.72	4.64	8.92	6.26	6.34	5.88	7.40	4.98	5.02	5.14
3425	1.41, 3	2	+	80	0.16	34	46	.60	3.80	4.40	7.12	4.78	4.84	5.76	5.82	3.94	3.96	4.92
3426	1.41, 3	2	-	80	0.16	46	34	.60	5.98	4.82	10.70	7.86	7.94	6.26	8.62	6.06	6.10	5.30
3427	1.41, 3	2	+	80	0.33	27	53	.60	3.02	4.78	5.26	3.48	3.48	5.54	4.20	2.70	2.72	4.64
3428	1.41, 3	2	-	80	0.33	53	27	.60	7.58	5.06	12.92	9.54	9.58	6.30	10.98	7.70	7.74	5.00
3429	1.41, 3	2	+	80	0.50	20	60	.60	2.40	5.86	3.90	2.48	2.48	5.96	3.56	2.32	2.34	5.56
3430	1.41, 3	2	-	80	0.50	60	20	.60	9.60	6.00	14.82	11.34	11.36	6.86	13.10	9.70	9.74	5.82
3431	1.41, 3	2	0	100	0	50	50	.60	5.04	5.06	8.36	5.80	5.84	5.70	7.72	4.84	4.84	4.64
3432	1.41, 3	2	+	100	0.16	42	58	.60	4.14	5.06	6.88	4.46	4.56	5.70	5.88	3.74	3.76	4.98
3433	1.41, 3	2	-	100	0.16	58	42	.60	6.16	5.12	10.00	7.30	7.34	6.00	8.86	6.26	6.28	4.90
3434	1.41, 3	2	+	100	0.33	33	67	.60	2.54	5.12	4.98	3.32	3.32	5.44	4.34	2.82	2.82	4.98
3435	1.41, 3	2	-	100	0.33	67	33	.60	8.24	5.54	11.96	8.22	8.34	5.40	11.24	7.94	7.94	5.28
3436	1.41, 3	2	+	100	0.50	25	75	.60	1.96	5.14	3.64	2.44	2.46	5.44	3.34	2.00	2.00	5.14
3437	1.41, 3	2	-	100	0.50	75	25	.60	10.40	6.36	13.16	9.56	9.56	5.66	13.38	10.00	10.10	5.84
3438	1.41, 3	2	0	150	0	75	75	.60	5.48	5.38	8.44	5.78	5.80	5.60	8.14	5.66	5.68	5.78
3439	1.41, 3	2	+	150	0.16	63	87	.60	4.26	5.34	6.90	4.46	4.46	5.62	5.92	4.18	4.18	5.22
3440	1.41, 3	2	-	150	0.16	87	63	.60	6.52	5.18	10.40	7.20	7.20	5.56	10.10	7.20	7.26	5.52
3441	1.41, 3	2	+	150	0.33	50	100	.60	2.88	5.06	5.20	3.34	3.36	5.50	4.72	2.74	2.74	5.26
3442	1.41, 3	2	-	150	0.33	100	50	.60	8.04	5.28	12.04	8.90	8.90	5.78	11.36	7.82	7.84	5.46
3443	1.41, 3	2	+	150	0.50	37	113	.60	1.96	5.26	3.98	2.40	2.40	5.64	3.14	1.92	1.92	5.00
3444	1.41, 3	2	-	150	0.50	113	37	.60	9.96	5.72	14.04	10.44	10.46	5.76	12.88	9.38	9.42	5.52
3445	1.41, 3	2	0	200	0	100	100	.60	5.16	5.16	7.96	5.76	5.76	5.56	7.78	5.34	5.34	5.44
3446	1.41, 3	2	+	200	0.16	84	116	.60	4.00	5.34	6.76	4.60	4.60	5.78	6.50	3.88	3.88	5.46
3447	1.41, 3	2	-	200	0.16	116	84	.60	6.18	4.90	9.82	6.94	6.94	5.14	9.20	6.30	6.34	4.92
3448	1.41, 3	2	+	200	0.33	67	133	.60	3.02	4.98	5.44	3.30	3.30	5.84	4.72	3.08	3.08	5.06
3449	1.41, 3	2	-	200	0.33	133	67	.60	7.90	5.28	12.28	9.10	9.12	5.66	11.76	8.96	8.98	5.78
3450	1.41, 3	2	+	200	0.50	50	150	.60	1.90	4.98	4.06	2.52	2.52	6.10	3.24	1.78	1.78	4.80
3451	1.41, 3	2	-	200	0.50	150	50	.60	9.94	5.44	14.56	10.92	10.96	6.44	14.10	10.50	10.54	6.06
3452	1.41, 3	5	0	20	0	10	10	.60	5.58	4.80	9.38	7.12	7.32	6.14	9.02	6.62	6.80	5.62
3453	1.41, 3	5	+	20	0.16	8	12	.60	3.80	4.58	5.92	4.16	4.40	5.86	4.88	3.30	3.54	4.70
3454	1.41, 3	5	-	20	0.16	12	8	.60	9.30	5.96	14.10	10.94	11.18	6.90	14.08	10.48	10.72	6.40

	D	VR	P	N	Δn	n1	n2	ε	F g	B-F g	F t	F-GG t	F-HF t	B-F t	F g x t	F-GG g x t	F-HF g x t	B-F g x t
3455	1.41, 3	5	+	20	0.33	7	13	.60	2.72	4.70	4.52	3.34	3.48	5.90	3.64	2.68	2.74	4.60
3456	1.41, 3	5	-	20	0.33	13	7	.60	12.18	7.40	16.32	12.74	12.94	7.56	16.60	12.78	13.06	7.20
3457	1.41, 3	5	+	20	0.50	5	15	.60	1.48	4.60	2.28	1.46	1.50	6.00	1.64	1.08	1.14	4.10
3458	1.41, 3	5	-	20	0.50	15	5	.60	18.62	10.80	24.02	19.28	19.46	10.08	23.64	18.60	19.06	9.70
3459	1.41, 3	5	0	40	0	20	20	.60	5.80	4.90	8.54	6.14	6.28	5.36	8.16	5.66	5.76	5.32
3460	1.41, 3	5	+	40	0.16	17	23	.60	3.80	4.90	5.46	3.82	3.92	4.90	5.16	3.40	3.52	4.60
3461	1.41, 3	5	-	40	0.16	23	17	.60	7.92	5.60	11.66	8.66	8.72	5.94	11.70	8.68	8.78	5.36
3462	1.41, 3	5	+	40	0.33	13	27	.60	1.68	4.28	2.74	1.56	1.68	5.00	2.48	1.76	1.80	4.36
3463	1.41, 3	5	-	40	0.33	27	13	.60	12.88	6.22	17.02	13.46	13.58	6.52	16.80	12.62	12.80	6.20
3464	1.41, 3	5	+	40	0.50	10	30	.60	.94	3.96	1.40	.80	.82	5.36	1.52	1.00	1.00	4.54
3465	1.41, 3	5	-	40	0.50	30	10	.60	16.84	7.38	22.44	18.00	18.22	7.70	22.18	17.90	18.12	7.88
3466	1.41, 3	5	0	60	0	30	30	.60	4.98	4.56	8.36	5.78	5.86	5.32	7.78	5.36	5.36	5.16
3467	1.41, 3	5	+	60	0.16	25	35	.60	3.20	4.84	5.08	3.56	3.62	4.98	5.42	3.16	3.22	5.16
3468	1.41, 3	5	-	60	0.16	35	25	.60	7.96	5.08	12.20	8.84	8.90	5.74	12.24	8.78	8.80	5.64
3469	1.41, 3	5	+	60	0.33	20	40	.60	1.88	5.00	3.16	1.80	1.82	4.68	3.14	1.96	2.00	4.90
3470	1.41, 3	5	-	60	0.33	40	20	.60	12.06	5.80	16.66	12.88	12.94	6.10	17.02	13.18	13.30	6.04
3471	1.41, 3	5	+	60	0.50	15	45	.60	.96	4.84	1.46	.90	.90	4.94	1.48	.98	.98	4.72
3472	1.41, 3	5	-	60	0.50	45	15	.60	17.44	6.42	22.16	17.36	17.46	6.82	22.10	17.52	17.54	6.72
3473	1.41, 3	5	0	80	0	40	40	.60	5.30	4.78	9.22	6.64	6.64	6.14	8.00	5.70	5.76	5.36
3474	1.41, 3	5	+	80	0.16	34	46	.60	3.16	4.42	6.26	4.12	4.14	6.12	5.28	3.38	3.40	5.06
3475	1.41, 3	5	-	80	0.16	46	34	.60	7.88	5.08	12.66	9.60	9.62	6.52	11.14	8.28	8.32	5.66
3476	1.41, 3	5	+	80	0.33	27	53	.60	1.78	4.66	3.28	1.98	1.98	5.60	2.90	1.90	1.90	4.78
3477	1.41, 3	5	-	80	0.33	53	27	.60	11.62	5.24	16.98	13.24	13.28	6.32	15.92	12.10	12.10	5.38
3478	1.41, 3	5	+	80	0.50	20	60	.60	.90	4.96	1.64	1.06	1.06	5.82	1.38	.88	.90	5.18
3479	1.41, 3	5	-	80	0.50	60	20	.60	16.38	6.08	23.62	19.12	19.18	6.74	22.16	17.70	17.76	6.06
3480	1.41, 3	5	0	100	0	50	50	.60	5.10	4.84	8.66	5.98	6.06	5.62	7.76	5.22	5.22	4.90
3481	1.41, 3	5	+	100	0.16	42	58	.60	3.30	4.86	5.42	3.36	3.42	5.64	5.06	3.18	3.18	4.98
3482	1.41, 3	5	-	100	0.16	58	42	.60	8.14	5.10	11.82	8.86	8.90	5.58	11.38	7.78	7.78	5.12
3483	1.41, 3	5	+	100	0.33	33	67	.60	1.28	4.64	2.84	1.72	1.74	5.46	2.66	1.40	1.40	5.04
3484	1.41, 3	5	-	100	0.33	67	33	.60	12.28	5.62	16.66	12.72	12.74	5.20	16.04	12.46	12.52	5.26
3485	1.41, 3	5	+	100	0.50	25	75	.60	.66	4.78	1.30	.78	.78	5.40	1.28	.58	.58	4.78
3486	1.41, 3	5	-	100	0.50	75	25	.60	17.42	6.16	20.82	16.70	16.72	5.70	22.10	17.48	17.50	5.98

	D	VR	P	N	Δn	n1	n2	ε	F g	B-F g	F t	F-GG t	F-HF t	B-F t	F g x t	F-GG g x t	F-HF g x t	B-F g x t
3487	1.41, 3	5	0	150	0	75	75	.60	5.86	5.52	8.18	5.88	5.90	5.56	8.38	5.94	5.94	5.90
3488	1.41, 3	5	+	150	0.16	63	87	.60	3.50	5.34	5.46	3.56	3.58	5.46	5.32	3.48	3.48	5.58
3489	1.41, 3	5	-	150	0.16	87	63	.60	8.28	5.12	11.84	8.72	8.72	5.64	11.90	8.70	8.76	5.70
3490	1.41, 3	5	+	150	0.33	50	100	.60	1.66	4.80	3.24	1.62	1.62	5.60	2.74	1.44	1.46	5.34
3491	1.41, 3	5	-	150	0.33	100	50	.60	11.98	5.32	16.48	12.80	12.84	5.90	15.76	12.30	12.38	5.60
3492	1.41, 3	5	+	150	0.50	37	113	.60	.66	4.94	1.38	.90	.92	5.44	1.22	.78	.78	4.86
3493	1.41, 3	5	-	150	0.50	113	37	.60	17.58	5.86	22.18	17.80	17.82	5.78	22.12	16.98	17.02	5.68
3494	1.41, 3	5	0	200	0	100	100	.60	5.26	5.06	7.92	5.54	5.58	5.40	8.00	5.34	5.36	5.34
3495	1.41, 3	5	+	200	0.16	84	116	.60	2.86	5.12	5.24	3.34	3.34	5.76	4.96	3.34	3.34	5.00
3496	1.41, 3	5	-	200	0.16	116	84	.60	8.02	4.80	12.10	8.48	8.48	5.04	11.40	8.28	8.30	5.08
3497	1.41, 3	5	+	200	0.33	67	133	.60	1.50	4.62	3.14	1.84	1.84	5.60	2.82	1.80	1.80	4.96
3498	1.41, 3	5	-	200	0.33	133	67	.60	12.02	5.06	17.12	13.46	13.48	6.18	16.78	13.10	13.14	5.70
3499	1.41, 3	5	+	200	0.50	50	150	.60	.56	4.78	1.58	.92	.92	5.94	1.04	.50	.50	4.76
3500	1.41, 3	5	-	200	0.50	150	50	.60	17.20	5.58	22.30	18.28	18.34	6.26	22.64	18.06	18.06	5.92
3501	1.41, 3	1	0	20	0	10	10	.70	4.28	3.68	6.50	4.80	5.16	4.28	6.68	4.66	5.04	4.16
3502	1.41, 3	1	0	20	0.16	8	12	.70	4.58	4.06	6.56	4.38	4.70	4.20	6.14	4.36	4.62	4.04
3503	1.41, 3	1	0	20	0.33	7	13	.70	4.90	4.72	6.42	4.58	4.94	4.36	6.30	4.26	4.66	4.08
3504	1.41, 3	1	0	20	0.50	5	15	.70	4.68	7.24	6.60	5.02	5.22	6.42	6.82	4.80	5.04	5.74
3505	1.41, 3	1	0	40	0	20	20	.70	5.06	5.14	6.68	4.72	4.80	4.68	6.26	4.68	4.80	4.42
3506	1.41, 3	1	0	40	0.16	17	23	.70	4.74	4.86	6.46	4.90	5.10	4.90	6.30	4.58	4.76	4.70
3507	1.41, 3	1	0	40	0.33	13	27	.70	4.50	4.84	6.84	4.94	5.14	5.34	6.32	4.68	4.72	4.70
3508	1.41, 3	1	0	40	0.50	10	30	.70	4.20	6.04	6.92	5.06	5.22	6.18	6.58	4.80	4.98	5.84
3509	1.41, 3	1	0	60	0	30	30	.70	4.78	4.62	7.12	5.24	5.28	5.28	7.40	5.32	5.44	5.42
3510	1.41, 3	1	0	60	0.16	25	35	.70	4.78	4.84	6.96	5.10	5.22	5.26	6.96	4.84	4.92	4.84
3511	1.41, 3	1	0	60	0.33	20	40	.70	5.22	5.34	6.98	5.08	5.26	5.36	6.32	4.88	4.98	4.92
3512	1.41, 3	1	0	60	0.50	15	45	.70	4.88	5.86	6.74	5.02	5.06	5.52	6.46	4.82	4.86	4.86
3513	1.41, 3	1	0	80	0	40	40	.70	4.40	4.24	7.20	5.36	5.48	5.50	6.36	4.72	4.74	4.76
3514	1.41, 3	1	0	80	0.16	34	46	.70	4.64	4.76	7.34	5.48	5.56	5.54	6.52	4.96	5.04	5.16
3515	1.41, 3	1	0	80	0.33	27	53	.70	5.00	5.34	7.00	5.44	5.50	5.46	6.56	4.86	4.96	5.18
3516	1.41, 3	1	0	80	0.50	20	60	.70	4.94	6.20	7.18	5.38	5.50	6.18	6.68	5.04	5.08	5.04
3517	1.41, 3	1	0	100	0	50	50	.70	4.74	4.74	7.34	5.66	5.72	5.78	6.50	4.62	4.68	4.88
3518	1.41, 3	1	0	100	0.16	42	58	.70	4.70	4.90	7.42	5.90	5.90	6.18	6.20	4.70	4.74	5.02

	D	VR	P	N	Δn	n1	n2	ε	F g	B-F g	F t	F-GG t	F-HF t	B-F t	F g x t	F-GG g x t	F-HF g x t	B-F g x t
3519	1.41, 3	1	0	100	0.33	33	67	.70	4.82	5.44	7.28	5.64	5.68	5.76	6.42	4.88	4.92	5.04
3520	1.41, 3	1	0	100	0.50	25	75	.70	4.70	5.80	6.88	5.46	5.52	5.86	6.38	4.52	4.54	4.92
3521	1.41, 3	1	0	150	0	75	75	.70	5.52	5.56	7.08	5.00	5.04	5.32	7.20	5.50	5.50	5.56
3522	1.41, 3	1	0	150	0.16	63	87	.70	5.32	5.46	7.08	5.18	5.20	5.34	6.76	4.58	4.62	4.86
3523	1.41, 3	1	0	150	0.33	50	100	.70	4.80	5.16	6.98	5.18	5.24	5.38	6.86	5.04	5.06	5.34
3524	1.41, 3	1	0	150	0.50	37	113	.70	5.00	5.66	6.76	5.06	5.10	5.50	6.84	5.20	5.22	5.68
3525	1.41, 3	1	0	200	0	100	100	.70	5.04	5.34	6.54	4.78	4.80	4.98	6.58	5.04	5.08	5.04
3526	1.41, 3	1	0	200	0.16	84	116	.70	5.00	5.16	6.78	5.24	5.26	5.10	7.04	5.08	5.10	5.28
3527	1.41, 3	1	0	200	0.33	67	133	.70	5.02	5.36	6.78	5.24	5.26	5.34	7.12	5.18	5.22	5.38
3528	1.41, 3	1	0	200	0.50	50	150	.70	4.62	5.32	6.62	4.98	5.00	5.46	6.82	5.10	5.10	5.48
3529	1.41, 3	1.5	0	20	0	10	10	.70	4.32	3.70	6.58	4.86	5.18	4.34	6.80	4.80	5.04	4.10
3530	1.41, 3	1.5	+	20	0.16	8	12	.70	4.30	4.06	5.50	3.92	4.18	4.24	5.36	3.72	3.92	4.10
3531	1.41, 3	1.5	-	20	0.16	12	8	.70	5.66	4.72	7.78	5.66	5.92	4.42	7.76	5.74	5.92	4.36
3532	1.41, 3	1.5	+	20	0.33	7	13	.70	4.04	4.80	5.02	3.32	3.58	4.50	4.80	3.32	3.50	4.22
3533	1.41, 3	1.5	-	20	0.33	13	7	.70	5.88	5.38	8.42	6.10	6.52	4.82	8.14	6.26	6.48	4.76
3534	1.41, 3	1.5	+	20	0.50	5	15	.70	3.20	6.36	4.38	3.14	3.44	5.54	4.24	2.84	3.08	5.32
3535	1.41, 3	1.5	-	20	0.50	15	5	.70	7.12	8.08	10.28	7.52	7.94	6.74	10.20	7.90	8.26	7.20
3536	1.41, 3	1.5	0	40	0	20	20	.70	5.10	4.58	6.88	4.70	4.74	4.78	6.22	4.44	4.60	4.38
3537	1.41, 3	1.5	+	40	0.16	17	23	.70	3.92	4.18	5.76	4.24	4.36	4.70	5.58	3.92	4.00	4.32
3538	1.41, 3	1.5	-	40	0.16	23	17	.70	5.62	5.08	7.92	5.76	5.98	5.06	7.68	5.62	5.66	4.88
3539	1.41, 3	1.5	+	40	0.33	13	27	.70	3.10	4.26	5.04	3.44	3.52	5.00	4.66	3.42	3.52	4.72
3540	1.41, 3	1.5	-	40	0.33	27	13	.70	6.36	5.18	9.04	6.98	7.08	5.30	8.64	6.48	6.62	5.18
3541	1.41, 3	1.5	+	40	0.50	10	30	.70	2.66	5.38	4.32	3.02	3.08	5.66	4.10	2.76	2.88	5.42
3542	1.41, 3	1.5	-	40	0.50	30	10	.70	7.42	6.98	9.56	7.42	7.48	6.04	9.86	7.52	7.74	5.90
3543	1.41, 3	1.5	0	60	0	30	30	.70	4.30	4.48	6.96	5.22	5.24	5.42	7.24	5.38	5.40	5.44
3544	1.41, 3	1.5	+	60	0.16	25	35	.70	3.86	4.60	6.00	4.36	4.40	5.16	5.94	4.44	4.52	5.12
3545	1.41, 3	1.5	-	60	0.16	35	25	.70	5.44	4.74	7.94	5.88	6.02	5.20	7.86	5.84	5.94	5.12
3546	1.41, 3	1.5	+	60	0.33	20	40	.70	4.06	5.14	5.24	3.80	3.88	5.24	4.84	3.78	3.78	4.80
3547	1.41, 3	1.5	-	60	0.33	40	20	.70	6.42	5.40	8.80	6.64	6.74	5.48	9.22	6.82	6.96	5.38
3548	1.41, 3	1.5	+	60	0.50	15	45	.70	3.16	5.56	4.52	3.34	3.34	5.68	3.88	2.70	2.78	4.80
3549	1.41, 3	1.5	-	60	0.50	45	15	.70	7.64	6.12	10.18	8.20	8.28	5.66	10.42	7.92	7.96	5.58
3550	1.41, 3	1.5	0	80	0	40	40	.70	4.38	4.30	7.22	5.40	5.50	5.52	6.38	4.62	4.66	4.74

	D	VR	P	N	Δn	n1	n2	ε	F g	B-F g	F t	F-GG t	F-HF t	B-F t	F g x t	F-GG g x t	F-HF g x t	B-F g x t
3551	1.41, 3	1.5	+	80	0.16	34	46	.70	3.86	4.44	6.46	4.60	4.68	5.52	5.66	4.10	4.14	4.90
3552	1.41, 3	1.5	-	80	0.16	46	34	.70	5.58	4.90	8.26	6.20	6.26	5.38	7.48	5.50	5.56	4.98
3553	1.41, 3	1.5	+	80	0.33	27	53	.70	3.54	5.12	5.30	3.98	3.98	5.60	4.78	3.30	3.34	4.96
3554	1.41, 3	1.5	-	80	0.33	53	27	.70	6.36	5.16	9.44	7.00	7.08	5.70	8.54	6.76	6.80	5.14
3555	1.41, 3	1.5	+	80	0.50	20	60	.70	2.96	5.86	4.76	3.54	3.56	6.24	3.94	2.70	2.76	5.14
3556	1.41, 3	1.5	-	80	0.50	60	20	.70	7.42	5.80	10.86	8.44	8.48	6.20	10.26	8.20	8.30	5.68
3557	1.41, 3	1.5	0	100	0	50	50	.70	4.86	4.96	7.30	5.56	5.58	5.56	6.50	4.90	4.90	5.00
3558	1.41, 3	1.5	+	100	0.16	42	58	.70	3.94	4.96	6.76	5.00	5.08	6.06	5.24	4.08	4.16	4.68
3559	1.41, 3	1.5	-	100	0.16	58	42	.70	5.84	5.22	8.04	6.14	6.16	5.32	7.66	5.68	5.74	5.14
3560	1.41, 3	1.5	+	100	0.33	33	67	.70	3.58	4.98	5.42	4.14	4.20	5.90	4.80	3.40	3.42	5.12
3561	1.41, 3	1.5	-	100	0.33	67	33	.70	6.88	5.10	9.00	6.90	6.96	5.10	9.18	6.68	6.80	4.68
3562	1.41, 3	1.5	+	100	0.50	25	75	.70	2.68	5.34	4.80	3.40	3.42	5.96	3.64	2.48	2.48	4.72
3563	1.41, 3	1.5	-	100	0.50	75	25	.70	7.74	6.24	10.50	8.32	8.36	5.42	9.94	7.84	7.88	5.50
3564	1.41, 3	1.5	0	150	0	75	75	.70	5.54	5.58	6.76	5.02	5.04	5.24	7.22	5.26	5.26	5.64
3565	1.41, 3	1.5	+	150	0.16	63	87	.70	4.70	5.62	6.02	4.22	4.24	5.32	5.54	3.96	3.96	4.82
3566	1.41, 3	1.5	-	150	0.16	87	63	.70	6.28	5.38	7.54	5.64	5.72	5.14	8.06	6.08	6.12	5.20
3567	1.41, 3	1.5	+	150	0.33	50	100	.70	3.62	4.94	5.10	3.84	3.90	5.36	5.40	3.80	3.90	5.28
3568	1.41, 3	1.5	-	150	0.33	100	50	.70	6.78	5.30	9.02	6.86	6.92	5.30	8.84	7.00	7.00	5.28
3569	1.41, 3	1.5	+	150	0.50	37	113	.70	2.84	5.10	4.36	3.10	3.10	5.48	4.46	3.22	3.26	5.52
3570	1.41, 3	1.5	-	150	0.50	113	37	.70	7.72	5.72	10.08	7.70	7.72	5.24	9.74	7.28	7.34	4.92
3571	1.41, 3	1.5	0	200	0	100	100	.70	5.20	5.16	6.92	5.00	5.02	4.98	6.50	4.96	4.96	4.96
3572	1.41, 3	1.5	+	200	0.16	84	116	.70	4.56	5.14	5.68	4.10	4.10	5.06	6.34	4.34	4.38	5.36
3573	1.41, 3	1.5	-	200	0.16	116	84	.70	5.86	5.14	7.96	5.86	5.90	5.30	7.78	5.86	5.86	5.18
3574	1.41, 3	1.5	+	200	0.33	67	133	.70	3.58	5.26	5.12	3.86	3.88	5.36	5.16	3.66	3.66	5.36
3575	1.41, 3	1.5	-	200	0.33	133	67	.70	6.62	5.44	8.66	6.42	6.46	4.58	8.98	6.76	6.78	5.52
3576	1.41, 3	1.5	+	200	0.50	50	150	.70	2.90	5.34	4.06	2.88	2.88	5.34	4.28	3.06	3.08	5.60
3577	1.41, 3	1.5	-	200	0.50	150	50	.70	7.68	5.84	10.12	7.84	7.86	5.32	10.18	7.98	7.98	5.24
3578	1.41, 3	2	0	20	0	10	10	.70	4.54	3.96	6.66	4.92	5.22	4.30	6.78	5.04	5.26	4.06
3579	1.41, 3	2	+	20	0.16	8	12	.70	4.10	3.98	4.92	3.42	3.60	4.26	4.92	3.38	3.56	4.10
3580	1.41, 3	2	-	20	0.16	12	8	.70	6.82	5.38	8.72	6.56	6.84	4.42	8.78	6.22	6.62	4.44
3581	1.41, 3	2	+	20	0.33	7	13	.70	3.52	4.62	4.26	2.68	2.94	4.36	4.14	2.80	3.00	4.16
3582	1.41, 3	2	-	20	0.33	13	7	.70	7.40	6.14	9.82	7.32	7.74	4.98	9.46	7.16	7.46	5.02

	D	VR	P	N	Δn	n1	n2	ε	F g	B-F g	F t	F-GG t	F-HF t	B-F t	F g x t	F-GG g x t	F-HF g x t	B-F g x t
3583	1.41, 3	2	+	20	0.50	5	15	.70	2.52	5.74	3.28	2.16	2.32	5.34	3.00	1.94	2.14	5.04
3584	1.41, 3	2	-	20	0.50	15	5	.70	9.40	9.00	13.12	9.90	10.36	7.08	12.78	9.98	10.58	7.38
3585	1.41, 3	2	0	40	0	20	20	.70	5.00	4.58	6.84	4.84	4.90	4.52	6.04	4.48	4.60	4.36
3586	1.41, 3	2	+	40	0.16	17	23	.70	3.70	4.10	5.22	3.82	3.86	4.66	5.12	3.54	3.68	4.26
3587	1.41, 3	2	-	40	0.16	23	17	.70	6.06	4.98	8.68	6.34	6.58	4.98	8.34	6.08	6.22	5.14
3588	1.41, 3	2	+	40	0.33	13	27	.70	2.48	4.40	3.94	2.60	2.72	4.78	3.88	2.58	2.68	4.56
3589	1.41, 3	2	-	40	0.33	27	13	.70	7.36	5.66	10.58	8.38	8.58	5.36	10.38	7.82	8.18	5.22
3590	1.41, 3	2	+	40	0.50	10	30	.70	2.00	4.88	3.04	2.12	2.22	5.38	2.76	1.88	1.94	5.20
3591	1.41, 3	2	-	40	0.50	30	10	.70	9.12	7.16	12.10	9.42	9.52	6.22	12.78	9.98	10.10	6.00
3592	1.41, 3	2	0	60	0	30	30	.70	4.42	4.46	6.86	5.12	5.20	5.28	7.18	5.36	5.42	5.38
3593	1.41, 3	2	+	60	0.16	25	35	.70	3.48	4.32	5.62	3.92	3.96	5.12	5.50	4.00	4.10	5.20
3594	1.41, 3	2	-	60	0.16	35	25	.70	6.10	4.94	8.60	6.66	6.70	5.16	8.54	6.62	6.66	4.96
3595	1.41, 3	2	+	60	0.33	20	40	.70	3.08	4.86	4.24	3.04	3.12	5.10	4.14	3.04	3.10	4.80
3596	1.41, 3	2	-	60	0.33	40	20	.70	7.94	5.58	10.36	8.16	8.28	5.52	10.76	8.46	8.56	5.38
3597	1.41, 3	2	+	60	0.50	15	45	.70	2.36	5.34	3.26	2.12	2.16	5.46	2.74	1.78	1.80	4.86
3598	1.41, 3	2	-	60	0.50	45	15	.70	9.58	6.50	12.76	10.24	10.44	5.60	13.26	10.56	10.64	5.78
3599	1.41, 3	2	0	80	0	40	40	.70	4.58	4.36	7.18	5.36	5.38	5.22	6.28	4.68	4.76	4.70
3600	1.41, 3	2	+	80	0.16	34	46	.70	3.44	4.26	5.84	4.20	4.26	5.42	5.18	3.52	3.56	4.80
3601	1.41, 3	2	-	80	0.16	46	34	.70	6.04	5.22	8.86	6.74	6.80	5.38	8.04	6.02	6.10	4.90
3602	1.41, 3	2	+	80	0.33	27	53	.70	2.76	5.02	4.38	3.14	3.16	5.66	3.88	2.48	2.50	4.96
3603	1.41, 3	2	-	80	0.33	53	27	.70	7.86	5.44	11.14	8.60	8.64	5.56	10.18	8.06	8.14	5.04
3604	1.41, 3	2	+	80	0.50	20	60	.70	2.22	5.60	3.32	2.16	2.16	6.16	2.60	1.50	1.56	5.14
3605	1.41, 3	2	-	80	0.50	60	20	.70	9.58	5.98	13.78	10.80	10.96	6.42	13.20	10.46	10.52	5.76
3606	1.41, 3	2	0	100	0	50	50	.70	4.90	4.92	7.22	5.58	5.64	5.60	6.30	5.08	5.12	4.86
3607	1.41, 3	2	+	100	0.16	42	58	.70	3.62	4.82	6.04	4.48	4.50	6.08	4.76	3.36	3.40	4.58
3608	1.41, 3	2	-	100	0.16	58	42	.70	6.44	5.32	8.54	6.56	6.62	5.36	8.22	6.42	6.50	5.20
3609	1.41, 3	2	+	100	0.33	33	67	.70	2.46	4.88	4.54	3.24	3.30	5.82	3.62	2.56	2.56	5.02
3610	1.41, 3	2	-	100	0.33	67	33	.70	8.16	5.44	10.74	8.38	8.42	5.48	11.00	8.36	8.46	5.16
3611	1.41, 3	2	+	100	0.50	25	75	.70	2.02	5.12	3.20	2.04	2.12	6.02	2.26	1.46	1.50	4.68
3612	1.41, 3	2	-	100	0.50	75	25	.70	10.18	6.38	13.24	10.78	10.84	5.62	12.78	10.24	10.30	5.52
3613	1.41, 3	2	0	150	0	75	75	.70	5.62	5.64	6.60	4.82	4.84	4.98	7.02	5.22	5.26	5.68
3614	1.41, 3	2	+	150	0.16	63	87	.70	4.34	5.50	5.30	3.88	3.90	5.24	5.12	3.66	3.70	4.96

	D	VR	P	N	Δn	n1	n2	ε	F g	B-F g	F t	F-GG t	F-HF t	B-F t	F g x t	F-GG g x t	F-HF g x t	B-F g x t
3615	1.41, 3	2	-	150	0.16	87	63	.70	6.80	5.50	8.36	6.30	6.32	4.96	8.66	6.54	6.56	5.12
3616	1.41, 3	2	+	150	0.33	50	100	.70	2.92	4.90	4.08	2.96	2.96	5.32	4.32	3.00	3.00	5.26
3617	1.41, 3	2	-	150	0.33	100	50	.70	7.90	5.36	10.32	8.34	8.34	5.12	10.36	8.22	8.28	5.36
3618	1.41, 3	2	+	150	0.50	37	113	.70	1.98	5.18	3.06	2.12	2.14	5.54	3.12	2.04	2.04	5.44
3619	1.41, 3	2	-	150	0.50	113	37	.70	10.24	5.86	12.90	10.46	10.48	5.26	12.38	9.80	9.86	5.06
3620	1.41, 3	2	0	200	0	100	100	.70	5.14	5.26	6.96	5.18	5.22	5.08	6.42	4.98	4.98	5.02
3621	1.41, 3	2	+	200	0.16	84	116	.70	4.00	5.22	5.20	3.96	3.96	4.96	5.70	3.96	3.96	5.38
3622	1.41, 3	2	-	200	0.16	116	84	.70	6.40	5.28	8.54	6.36	6.36	5.34	8.64	6.46	6.48	5.12
3623	1.41, 3	2	+	200	0.33	67	133	.70	2.90	5.06	4.12	3.04	3.06	5.12	3.96	2.80	2.82	5.24
3624	1.41, 3	2	-	200	0.33	133	67	.70	7.94	5.34	10.52	7.86	7.90	4.56	10.54	8.36	8.42	5.36
3625	1.41, 3	2	+	200	0.50	50	150	.70	2.14	5.42	2.66	2.12	2.14	5.20	3.16	2.18	2.20	5.50
3626	1.41, 3	2	-	200	0.50	150	50	.70	10.18	5.90	12.68	10.32	10.38	5.34	13.40	10.48	10.54	5.16
3627	1.41, 3	5	0	20	0	10	10	.70	6.10	5.28	6.98	5.20	5.40	4.08	7.38	5.30	5.62	4.28
3628	1.41, 3	5	+	20	0.16	8	12	.70	3.88	4.62	3.64	2.60	2.84	4.06	3.52	2.24	2.46	3.88
3629	1.41, 3	5	-	20	0.16	12	8	.70	9.96	6.44	11.74	9.08	9.30	4.82	11.74	8.92	9.24	4.38
3630	1.41, 3	5	+	20	0.33	7	13	.70	3.00	4.02	2.48	1.68	1.90	3.56	2.46	1.68	1.76	3.34
3631	1.41, 3	5	-	20	0.33	13	7	.70	12.40	7.12	14.58	11.38	11.80	4.80	14.48	11.18	11.72	4.76
3632	1.41, 3	5	+	20	0.50	5	15	.70	1.46	4.86	.86	.56	.62	4.62	.94	.60	.62	4.64
3633	1.41, 3	5	-	20	0.50	15	5	.70	18.82	10.16	22.28	18.50	18.94	6.60	22.66	18.46	18.94	7.06
3634	1.41, 3	5	0	40	0	20	20	.70	5.54	5.10	6.94	5.04	5.10	4.74	6.24	4.78	4.96	4.52
3635	1.41, 3	5	+	40	0.16	17	23	.70	3.48	4.36	4.14	2.96	3.06	4.62	4.16	2.82	2.86	4.26
3636	1.41, 3	5	-	40	0.16	23	17	.70	8.34	5.52	10.28	8.00	8.10	4.96	10.74	8.28	8.54	5.14
3637	1.41, 3	5	+	40	0.33	13	27	.70	1.62	4.04	1.84	1.34	1.34	4.52	2.00	1.28	1.34	4.46
3638	1.41, 3	5	-	40	0.33	27	13	.70	12.36	5.94	16.20	12.92	13.06	5.18	16.10	13.24	13.38	5.16
3639	1.41, 3	5	+	40	0.50	10	30	.70	.92	4.26	1.04	.54	.58	4.82	.92	.52	.56	4.74
3640	1.41, 3	5	-	40	0.50	30	10	.70	17.04	7.08	21.92	18.16	18.32	5.70	21.78	18.28	18.42	5.50
3641	1.41, 3	5	0	60	0	30	30	.70	4.66	4.58	7.10	5.50	5.58	5.04	7.16	5.36	5.44	4.94
3642	1.41, 3	5	+	60	0.16	25	35	.70	3.12	4.38	4.00	2.86	2.92	4.84	4.22	2.66	2.74	5.06
3643	1.41, 3	5	-	60	0.16	35	25	.70	8.24	5.30	10.62	8.32	8.46	4.86	10.80	8.32	8.44	4.72
3644	1.41, 3	5	+	60	0.33	20	40	.70	1.86	4.72	2.16	1.54	1.58	5.16	2.16	1.30	1.38	4.54
3645	1.41, 3	5	-	60	0.33	40	20	.70	12.02	5.58	15.56	13.04	13.16	5.38	15.80	13.02	13.26	5.28
3646	1.41, 3	5	+	60	0.50	15	45	.70	.86	4.78	.98	.56	.56	5.28	.78	.42	.46	4.60

	D	VR	P	N	Δn	n1	n2	ε	F g	B-F g	F t	F-GG t	F-HF t	B-F t	F g x t	F-GG g x t	F-HF g x t	B-F g x t
3647	1.41, 3	5	-	60	0.50	45	15	.70	17.70	6.64	21.98	18.70	18.90	5.36	22.20	18.46	18.70	5.80
3648	1.41, 3	5	0	80	0	40	40	.70	5.10	4.50	7.28	5.28	5.38	5.32	6.40	4.72	4.76	4.56
3649	1.41, 3	5	+	80	0.16	34	46	.70	2.76	4.34	4.64	3.24	3.24	5.54	3.98	2.60	2.66	4.94
3650	1.41, 3	5	-	80	0.16	46	34	.70	8.18	5.30	10.72	8.10	8.14	5.46	9.82	7.54	7.66	4.74
3651	1.41, 3	5	+	80	0.33	27	53	.70	1.64	4.54	2.28	1.64	1.66	5.54	1.76	1.10	1.12	4.62
3652	1.41, 3	5	-	80	0.33	53	27	.70	12.24	5.52	16.60	13.18	13.26	5.26	15.34	12.60	12.64	5.10
3653	1.41, 3	5	+	80	0.50	20	60	.70	.96	5.14	1.14	.66	.68	5.76	.52	.22	.22	5.06
3654	1.41, 3	5	-	80	0.50	60	20	.70	16.28	6.16	23.76	19.78	19.82	6.10	22.44	18.98	19.04	5.68
3655	1.41, 3	5	0	100	0	50	50	.70	5.10	4.88	6.58	5.14	5.14	4.96	6.56	4.60	4.70	4.60
3656	1.41, 3	5	+	100	0.16	42	58	.70	3.16	4.84	4.34	2.98	3.02	5.54	3.42	2.40	2.42	4.66
3657	1.41, 3	5	-	100	0.16	58	42	.70	8.28	5.42	10.70	8.64	8.66	5.24	10.32	7.82	7.86	5.16
3658	1.41, 3	5	+	100	0.33	33	67	.70	1.48	4.22	2.12	1.30	1.32	5.78	1.84	1.02	1.02	4.68
3659	1.41, 3	5	-	100	0.33	67	33	.70	12.08	5.88	16.16	13.08	13.18	5.24	16.28	13.14	13.24	5.18
3660	1.41, 3	5	+	100	0.50	25	75	.70	.84	4.38	.78	.40	.40	6.00	.60	.30	.30	4.38
3661	1.41, 3	5	-	100	0.50	75	25	.70	17.16	6.50	21.52	18.10	18.20	5.92	21.66	17.96	18.04	5.52
3662	1.41, 3	5	0	150	0	75	75	.70	5.78	5.78	6.76	5.02	5.08	5.10	6.90	4.92	4.98	5.18
3663	1.41, 3	5	+	150	0.16	63	87	.70	3.58	5.20	4.02	2.88	2.88	5.06	4.02	2.72	2.76	5.14
3664	1.41, 3	5	-	150	0.16	87	63	.70	8.52	5.46	10.18	8.08	8.10	4.98	10.76	8.20	8.28	5.32
3665	1.41, 3	5	+	150	0.33	50	100	.70	1.58	4.84	2.10	1.28	1.28	5.14	2.16	1.44	1.46	5.26
3666	1.41, 3	5	-	150	0.33	100	50	.70	11.92	5.64	15.48	12.80	12.92	5.14	14.96	11.96	11.98	5.08
3667	1.41, 3	5	+	150	0.50	37	113	.70	.66	4.94	.94	.48	.48	5.34	.96	.54	.54	5.52
3668	1.41, 3	5	-	150	0.50	113	37	.70	17.02	6.18	21.90	18.62	18.66	4.94	21.58	18.00	18.10	5.04
3669	1.41, 3	5	0	200	0	100	100	.70	5.58	5.60	6.84	5.02	5.04	5.08	6.46	4.78	4.82	4.88
3670	1.41, 3	5	+	200	0.16	84	116	.70	3.22	5.18	4.00	2.76	2.76	5.22	4.06	3.06	3.06	5.10
3671	1.41, 3	5	-	200	0.16	116	84	.70	7.98	5.20	9.94	8.00	8.04	5.12	10.64	8.34	8.38	5.34
3672	1.41, 3	5	+	200	0.33	67	133	.70	1.82	4.92	1.96	1.42	1.44	5.22	2.04	1.40	1.40	5.30
3673	1.41, 3	5	-	200	0.33	133	67	.70	12.36	5.30	15.38	12.26	12.28	4.82	15.46	12.62	12.66	5.26
3674	1.41, 3	5	+	200	0.50	50	150	.70	.60	5.02	.92	.48	.50	4.98	.92	.46	.46	5.70
3675	1.41, 3	5	-	200	0.50	150	50	.70	17.52	5.72	21.62	18.34	18.36	5.30	21.96	18.64	18.74	5.22
3676	1.41, 3	1	0	20	0	10	10	.80	4.06	3.48	5.90	4.48	4.90	4.02	6.20	4.62	5.08	4.12
3677	1.41, 3	1	0	20	0.16	8	12	.80	4.62	4.16	5.74	4.32	4.62	3.88	5.38	3.98	4.36	3.80
3678	1.41, 3	1	0	20	0.33	7	13	.80	4.88	5.02	5.62	4.36	4.74	4.62	5.80	4.62	4.84	4.48

	D	VR	P	N	Δn	n1	n2	ε	F g	B-F g	F t	F-GG t	F-HF t	B-F t	F g x t	F-GG g x t	F-HF g x t	B-F g x t
3679	1.41, 3	1	0	20	0.50	5	15	.80	4.52	6.46	6.08	4.92	5.22	5.62	6.00	4.78	5.14	5.56
3680	1.41, 3	1	0	40	0	20	20	.80	5.06	4.56	5.62	4.56	4.60	4.38	6.12	4.70	4.92	4.66
3681	1.41, 3	1	0	40	0.16	17	23	.80	5.06	5.00	5.74	4.70	4.80	4.48	5.80	4.74	4.80	4.42
3682	1.41, 3	1	0	40	0.33	13	27	.80	4.56	5.16	6.08	4.58	4.86	4.72	6.48	5.14	5.28	5.34
3683	1.41, 3	1	0	40	0.50	10	30	.80	4.22	5.84	5.68	4.64	4.74	5.02	6.26	5.14	5.30	6.62
3684	1.41, 3	1	0	60	0	30	30	.80	5.20	4.94	5.92	4.78	4.82	4.66	5.86	4.62	4.78	4.62
3685	1.41, 3	1	0	60	0.16	25	35	.80	5.10	4.94	6.02	4.90	5.06	4.92	5.82	4.66	4.86	4.44
3686	1.41, 3	1	0	60	0.33	20	40	.80	4.68	5.16	6.10	4.92	5.00	4.90	6.24	5.26	5.34	5.24
3687	1.41, 3	1	0	60	0.50	15	45	.80	5.12	6.08	5.72	4.58	4.64	4.88	5.88	4.76	4.88	5.52
3688	1.41, 3	1	0	80	0	40	40	.80	4.98	4.88	6.56	5.34	5.46	5.24	5.64	4.50	4.54	4.60
3689	1.41, 3	1	0	80	0.16	34	46	.80	4.68	5.00	6.28	4.98	5.04	4.98	5.76	4.92	4.94	4.92
3690	1.41, 3	1	0	80	0.33	27	53	.80	5.34	5.42	6.10	4.68	4.80	5.50	5.58	4.20	4.30	4.54
3691	1.41, 3	1	0	80	0.50	20	60	.80	5.12	5.88	6.16	5.14	5.20	5.42	5.90	4.84	4.88	5.22
3692	1.41, 3	1	0	100	0	50	50	.80	5.48	5.40	5.72	4.68	4.72	4.82	5.94	5.00	5.02	4.82
3693	1.41, 3	1	0	100	0.16	42	58	.80	5.02	5.04	5.90	4.82	4.88	4.76	5.50	4.46	4.52	4.74
3694	1.41, 3	1	0	100	0.33	33	67	.80	4.90	5.50	5.56	4.58	4.62	4.64	5.78	4.66	4.78	4.68
3695	1.41, 3	1	0	100	0.50	25	75	.80	4.88	5.62	5.62	4.60	4.68	5.14	5.30	4.24	4.28	4.76
3696	1.41, 3	1	0	150	0	75	75	.80	5.56	5.48	6.64	5.38	5.42	5.62	6.92	5.50	5.54	5.66
3697	1.41, 3	1	0	150	0.16	63	87	.80	5.52	5.76	6.50	5.02	5.12	5.56	5.96	4.94	5.00	5.08
3698	1.41, 3	1	0	150	0.33	50	100	.80	4.90	5.42	6.22	5.02	5.06	5.14	5.92	4.72	4.76	4.90
3699	1.41, 3	1	0	150	0.50	37	113	.80	5.26	5.78	5.82	4.62	4.70	5.28	5.62	4.36	4.42	5.00
3700	1.41, 3	1	0	200	0	100	100	.80	5.02	4.94	6.32	5.08	5.10	5.34	6.32	5.24	5.24	5.28
3701	1.41, 3	1	0	200	0.16	84	116	.80	5.34	5.46	6.30	5.34	5.38	5.64	6.38	5.44	5.46	5.54
3702	1.41, 3	1	0	200	0.33	67	133	.80	5.12	5.08	6.34	5.06	5.10	5.36	6.26	5.20	5.24	5.20
3703	1.41, 3	1	0	200	0.50	50	150	.80	5.42	5.88	6.10	5.04	5.06	5.32	5.68	4.70	4.74	5.04
3704	1.41, 3	1.5	0	20	0	10	10	.80	4.52	3.92	6.26	4.80	5.10	4.14	6.30	4.54	4.98	4.12
3705	1.41, 3	1.5	+	20	0.16	8	12	.80	4.28	4.02	4.78	3.60	3.86	3.96	4.40	3.42	3.74	3.72
3706	1.41, 3	1.5	-	20	0.16	12	8	.80	5.48	4.50	7.18	5.92	6.24	4.28	7.34	5.86	6.26	4.72
3707	1.41, 3	1.5	+	20	0.33	7	13	.80	3.92	4.78	4.22	3.18	3.42	4.30	4.42	3.64	3.74	4.36
3708	1.41, 3	1.5	-	20	0.33	13	7	.80	5.80	5.20	7.54	6.06	6.36	4.36	7.96	6.46	6.78	4.94
3709	1.41, 3	1.5	+	20	0.50	5	15	.80	3.12	5.36	4.06	2.88	3.02	5.04	3.82	2.98	3.26	5.08
3710	1.41, 3	1.5	-	20	0.50	15	5	.80	7.82	8.94	9.20	7.32	7.66	6.18	9.62	7.66	8.14	6.04

	D	VR	P	N	Δn	n1	n2	ε	F g	B-F g	F t	F-GG t	F-HF t	B-F t	F g x t	F-GG g x t	F-HF g x t	B-F g x t
3711	1.41, 3	1.5	0	40	0	20	20	.80	5.08	4.60	5.80	4.44	4.52	4.30	5.80	4.48	4.68	4.30
3712	1.41, 3	1.5	+	40	0.16	17	23	.80	4.50	4.82	4.80	3.94	4.06	4.36	5.10	4.16	4.30	4.42
3713	1.41, 3	1.5	-	40	0.16	23	17	.80	5.56	5.28	6.52	5.26	5.40	4.70	6.68	5.22	5.48	4.48
3714	1.41, 3	1.5	+	40	0.33	13	27	.80	3.06	4.72	4.22	2.94	3.10	4.50	4.72	3.48	3.64	5.22
3715	1.41, 3	1.5	-	40	0.33	27	13	.80	6.92	5.68	8.02	6.32	6.54	5.16	7.10	5.86	6.18	4.62
3716	1.41, 3	1.5	+	40	0.50	10	30	.80	2.58	5.38	3.36	2.56	2.68	5.08	3.80	2.96	3.08	6.20
3717	1.41, 3	1.5	-	40	0.50	30	10	.80	7.32	7.16	9.10	7.36	7.52	5.44	8.50	7.18	7.38	5.32
3718	1.41, 3	1.5	0	60	0	30	30	.80	5.24	5.10	6.00	4.84	4.94	5.02	5.92	4.68	4.80	4.80
3719	1.41, 3	1.5	+	60	0.16	25	35	.80	4.20	4.84	5.02	4.18	4.28	4.52	4.96	4.02	4.12	4.74
3720	1.41, 3	1.5	-	60	0.16	35	25	.80	5.76	4.70	6.80	5.68	5.82	5.12	6.98	5.66	5.88	5.12
3721	1.41, 3	1.5	+	60	0.33	20	40	.80	3.58	5.04	4.54	3.36	3.48	4.96	4.72	3.66	3.78	5.12
3722	1.41, 3	1.5	-	60	0.33	40	20	.80	6.06	5.34	8.06	6.80	6.94	5.10	7.24	5.88	5.96	4.56
3723	1.41, 3	1.5	+	60	0.50	15	45	.80	3.08	5.72	3.42	2.78	2.86	4.92	3.66	2.94	3.00	5.36
3724	1.41, 3	1.5	-	60	0.50	45	15	.80	7.44	6.10	9.26	7.72	7.86	5.70	8.62	7.08	7.24	5.44
3725	1.41, 3	1.5	0	80	0	40	40	.80	4.88	4.82	6.64	5.42	5.50	5.26	5.88	4.46	4.52	4.50
3726	1.41, 3	1.5	+	80	0.16	34	46	.80	3.94	4.58	5.66	4.30	4.42	5.20	5.06	4.36	4.40	4.88
3727	1.41, 3	1.5	-	80	0.16	46	34	.80	5.48	4.70	7.60	6.30	6.44	5.32	6.64	5.40	5.44	4.80
3728	1.41, 3	1.5	+	80	0.33	27	53	.80	4.02	5.08	4.34	3.42	3.50	5.20	3.92	3.00	3.12	4.62
3729	1.41, 3	1.5	-	80	0.33	53	27	.80	6.46	5.12	8.68	7.24	7.34	5.32	8.50	6.82	6.92	4.82
3730	1.41, 3	1.5	+	80	0.50	20	60	.80	3.18	5.76	3.68	2.90	3.00	5.38	3.50	2.72	2.78	5.26
3731	1.41, 3	1.5	-	80	0.50	60	20	.80	7.50	5.78	9.92	8.18	8.30	5.94	9.20	7.82	7.98	5.36
3732	1.41, 3	1.5	0	100	0	50	50	.80	5.24	5.08	5.86	4.80	4.82	4.92	5.92	4.74	4.78	4.70
3733	1.41, 3	1.5	+	100	0.16	42	58	.80	4.44	4.92	4.96	3.84	3.96	4.80	4.82	3.82	3.86	4.60
3734	1.41, 3	1.5	-	100	0.16	58	42	.80	6.04	5.34	7.04	5.74	5.82	5.00	6.68	5.50	5.54	5.00
3735	1.41, 3	1.5	+	100	0.33	33	67	.80	3.60	5.32	4.34	3.44	3.50	4.66	4.00	3.08	3.12	4.98
3736	1.41, 3	1.5	-	100	0.33	67	33	.80	7.34	6.02	8.52	6.80	6.90	4.78	8.50	6.66	6.78	4.74
3737	1.41, 3	1.5	+	100	0.50	25	75	.80	3.04	5.38	3.60	2.64	2.74	4.98	3.14	2.40	2.44	4.68
3738	1.41, 3	1.5	-	100	0.50	75	25	.80	8.14	6.20	8.86	7.56	7.64	4.90	9.60	8.00	8.22	5.20
3739	1.41, 3	1.5	0	150	0	75	75	.80	5.28	5.14	6.62	5.42	5.44	5.54	6.96	5.62	5.72	5.96
3740	1.41, 3	1.5	+	150	0.16	63	87	.80	4.92	5.46	5.62	4.40	4.44	5.50	5.28	4.32	4.36	5.26
3741	1.41, 3	1.5	-	150	0.16	87	63	.80	5.68	5.06	7.54	6.28	6.32	5.76	7.06	5.96	5.98	5.10
3742	1.41, 3	1.5	+	150	0.33	50	100	.80	3.78	5.16	4.62	3.32	3.32	5.56	4.14	3.32	3.34	5.08

	D	VR	P	N	Δn	n1	n2	ε	F g	B-F g	F t	F-GG t	F-HF t	B-F t	F g x t	F-GG g x t	F-HF g x t	B-F g x t
3743	1.41, 3	1.5	-	150	0.33	100	50	.80	6.58	5.14	8.96	7.54	7.64	5.28	8.00	6.64	6.74	5.10
3744	1.41, 3	1.5	+	150	0.50	37	113	.80	3.14	5.54	3.54	2.94	2.94	5.20	3.04	2.44	2.46	4.88
3745	1.41, 3	1.5	-	150	0.50	113	37	.80	7.74	5.90	9.80	8.36	8.38	5.30	9.82	8.04	8.10	5.02
3746	1.41, 3	1.5	0	200	0	100	100	.80	5.12	5.10	6.32	5.16	5.26	5.48	6.34	5.38	5.40	5.26
3747	1.41, 3	1.5	+	200	0.16	84	116	.80	4.38	5.32	5.50	4.30	4.34	5.42	5.74	4.72	4.78	5.52
3748	1.41, 3	1.5	-	200	0.16	116	84	.80	5.78	5.10	7.38	6.16	6.18	5.36	7.26	6.10	6.14	5.36
3749	1.41, 3	1.5	+	200	0.33	67	133	.80	3.40	5.14	4.56	3.56	3.56	5.42	4.34	3.50	3.52	5.22
3750	1.41, 3	1.5	-	200	0.33	133	67	.80	6.44	5.26	8.70	7.24	7.28	5.60	8.38	7.00	7.04	5.48
3751	1.41, 3	1.5	+	200	0.50	50	150	.80	3.16	5.64	3.58	2.84	2.88	5.58	3.44	2.80	2.80	5.10
3752	1.41, 3	1.5	-	200	0.50	150	50	.80	7.86	5.72	10.04	8.72	8.78	5.72	9.82	8.22	8.28	5.14
3753	1.41, 3	2	0	20	0	10	10	.80	4.50	3.98	6.32	4.84	5.22	4.26	6.14	4.70	5.06	4.16
3754	1.41, 3	2	+	20	0.16	8	12	.80	4.02	4.20	4.50	3.26	3.46	4.22	4.14	3.08	3.38	3.66
3755	1.41, 3	2	-	20	0.16	12	8	.80	6.52	5.16	7.92	6.48	6.80	4.40	8.12	6.30	6.74	4.60
3756	1.41, 3	2	+	20	0.33	7	13	.80	3.38	4.64	3.54	2.62	2.86	4.20	3.84	2.96	3.12	4.10
3757	1.41, 3	2	-	20	0.33	13	7	.80	7.24	5.70	9.20	7.16	7.56	4.62	9.66	7.54	8.06	4.98
3758	1.41, 3	2	+	20	0.50	5	15	.80	2.48	4.86	2.64	1.88	2.12	4.92	2.72	1.92	2.10	4.92
3759	1.41, 3	2	-	20	0.50	15	5	.80	10.04	9.64	12.04	9.80	10.44	6.34	12.18	10.12	10.66	6.24
3760	1.41, 3	2	0	40	0	20	20	.80	5.02	4.56	5.72	4.38	4.64	4.20	5.70	4.56	4.66	4.40
3761	1.41, 3	2	+	40	0.16	17	23	.80	4.12	4.72	4.32	3.40	3.56	4.28	4.66	3.60	3.74	4.46
3762	1.41, 3	2	-	40	0.16	23	17	.80	6.30	5.38	7.28	5.76	5.98	4.94	7.34	5.82	6.10	4.46
3763	1.41, 3	2	+	40	0.33	13	27	.80	2.48	4.54	3.02	2.12	2.24	4.44	3.50	2.64	2.78	5.08
3764	1.41, 3	2	-	40	0.33	27	13	.80	8.24	5.82	9.70	7.98	8.14	5.24	8.54	7.18	7.26	4.52
3765	1.41, 3	2	+	40	0.50	10	30	.80	1.92	4.72	2.26	1.58	1.62	4.98	2.68	2.04	2.18	5.80
3766	1.41, 3	2	-	40	0.50	30	10	.80	9.70	7.40	12.00	10.10	10.26	5.42	11.38	9.34	9.64	5.34
3767	1.41, 3	2	0	60	0	30	30	.80	5.24	5.04	6.06	4.88	5.00	4.76	5.96	4.64	4.72	4.62
3768	1.41, 3	2	+	60	0.16	25	35	.80	3.88	4.50	4.52	3.50	3.62	4.70	4.40	3.30	3.40	4.56
3769	1.41, 3	2	-	60	0.16	35	25	.80	6.30	4.80	7.86	6.34	6.56	5.26	7.78	6.48	6.64	5.12
3770	1.41, 3	2	+	60	0.33	20	40	.80	2.80	5.10	3.52	2.72	2.84	4.96	3.58	2.70	2.78	5.16
3771	1.41, 3	2	-	60	0.33	40	20	.80	7.66	5.36	9.62	8.14	8.20	5.08	9.14	7.44	7.68	4.66
3772	1.41, 3	2	+	60	0.50	15	45	.80	2.26	5.40	2.42	1.76	1.84	5.08	2.66	2.10	2.10	5.16
3773	1.41, 3	2	-	60	0.50	45	15	.80	9.58	6.34	12.04	10.10	10.30	5.60	11.80	9.60	9.80	5.50
3774	1.41, 3	2	0	80	0	40	40	.80	4.90	4.76	6.80	5.28	5.34	5.32	5.68	4.66	4.72	4.54

	D	VR	P	N	Δn	n1	n2	ε	F g	B-F g	F t	F-GG t	F-HF t	B-F t	F g x t	F-GG g x t	F-HF g x t	B-F g x t
3775	1.41, 3	2	+	80	0.16	34	46	.80	3.52	4.46	5.16	4.04	4.18	5.54	4.80	3.96	4.04	5.08
3776	1.41, 3	2	-	80	0.16	46	34	.80	5.98	4.96	8.16	6.96	7.08	5.42	7.60	5.98	6.12	4.82
3777	1.41, 3	2	+	80	0.33	27	53	.80	2.92	4.68	3.50	2.62	2.68	5.28	3.02	2.48	2.52	4.68
3778	1.41, 3	2	-	80	0.33	53	27	.80	8.06	5.28	10.58	8.72	8.80	5.50	10.44	8.50	8.60	5.04
3779	1.41, 3	2	+	80	0.50	20	60	.80	2.14	5.68	2.50	1.90	1.92	5.08	2.30	1.88	1.94	5.30
3780	1.41, 3	2	-	80	0.50	60	20	.80	9.48	6.00	13.02	10.94	11.16	5.96	12.40	10.32	10.52	5.36
3781	1.41, 3	2	0	100	0	50	50	.80	5.04	4.96	6.00	4.92	4.94	5.00	5.88	4.84	4.90	4.70
3782	1.41, 3	2	+	100	0.16	42	58	.80	4.10	4.98	4.50	3.50	3.56	4.80	4.38	3.42	3.52	4.72
3783	1.41, 3	2	-	100	0.16	58	42	.80	6.50	5.40	7.66	6.28	6.42	5.20	7.48	6.02	6.10	4.90
3784	1.41, 3	2	+	100	0.33	33	67	.80	2.80	4.94	3.34	2.42	2.50	4.74	3.18	2.34	2.42	5.04
3785	1.41, 3	2	-	100	0.33	67	33	.80	8.52	6.10	10.44	8.66	8.82	4.90	10.32	8.38	8.46	4.62
3786	1.41, 3	2	+	100	0.50	25	75	.80	2.14	5.10	2.34	1.80	1.84	5.06	1.92	1.40	1.42	4.72
3787	1.41, 3	2	-	100	0.50	75	25	.80	10.44	6.12	11.82	9.74	9.92	5.00	12.76	10.56	10.66	5.16
3788	1.41, 3	2	0	150	0	75	75	.80	5.32	5.06	6.62	5.28	5.32	5.38	7.00	5.56	5.62	5.84
3789	1.41, 3	2	+	150	0.16	63	87	.80	4.38	5.52	5.16	3.98	3.98	5.60	4.86	3.88	3.94	5.28
3790	1.41, 3	2	-	150	0.16	87	63	.80	6.38	5.10	8.32	6.94	6.96	5.68	7.88	6.60	6.64	5.12
3791	1.41, 3	2	+	150	0.33	50	100	.80	3.02	5.08	3.36	2.70	2.70	5.48	3.32	2.66	2.66	5.14
3792	1.41, 3	2	-	150	0.33	100	50	.80	7.64	5.06	10.30	8.98	9.04	5.30	9.64	8.06	8.14	5.20
3793	1.41, 3	2	+	150	0.50	37	113	.80	1.98	5.36	2.56	1.96	2.00	5.20	1.98	1.56	1.62	4.80
3794	1.41, 3	2	-	150	0.50	113	37	.80	9.96	6.00	12.06	10.70	10.78	5.20	12.64	10.68	10.78	5.16
3795	1.41, 3	2	0	200	0	100	100	.80	5.38	5.38	6.30	5.08	5.14	5.28	6.34	5.28	5.36	5.34
3796	1.41, 3	2	+	200	0.16	84	116	.80	4.06	5.20	4.72	3.92	3.94	5.28	5.14	4.28	4.34	5.52
3797	1.41, 3	2	-	200	0.16	116	84	.80	6.42	5.02	8.26	6.86	6.92	5.44	8.22	6.84	6.86	5.60
3798	1.41, 3	2	+	200	0.33	67	133	.80	2.84	4.88	3.52	2.78	2.82	5.30	3.42	2.70	2.74	5.28
3799	1.41, 3	2	-	200	0.33	133	67	.80	7.86	5.28	10.18	8.76	8.78	5.68	10.02	8.56	8.62	5.58
3800	1.41, 3	2	+	200	0.50	50	150	.80	2.14	5.48	2.42	1.98	2.00	5.32	2.30	1.68	1.70	5.10
3801	1.41, 3	2	-	200	0.50	150	50	.80	9.78	5.64	12.82	11.34	11.40	5.90	12.76	10.80	10.84	5.20
3802	1.41, 3	5	0	20	0	10	10	.80	5.66	4.74	6.50	5.10	5.32	4.38	6.64	4.84	5.26	4.14
3803	1.41, 3	5	+	20	0.16	8	12	.80	3.70	4.80	3.28	2.34	2.48	4.22	3.20	2.30	2.52	3.80
3804	1.41, 3	5	-	20	0.16	12	8	.80	10.24	6.48	11.38	9.14	9.48	4.16	11.24	8.70	9.14	4.46
3805	1.41, 3	5	+	20	0.33	7	13	.80	2.74	4.94	2.02	1.48	1.60	4.18	2.04	1.58	1.74	3.72
3806	1.41, 3	5	-	20	0.33	13	7	.80	12.22	6.80	14.42	11.30	11.82	4.62	14.82	12.04	12.70	4.42

	D	VR	P	N	Δn	n1	n2	ε	F g	B-F g	F t	F-GG t	F-HF t	B-F t	F g x t	F-GG g x t	F-HF g x t	B-F g x t
3807	1.41, 3	5	+	20	0.50	5	15	.80	1.28	4.30	.78	.50	.58	4.30	.72	.44	.52	4.14
3808	1.41, 3	5	-	20	0.50	15	5	.80	18.66	10.72	22.06	18.76	19.46	6.36	21.70	18.62	19.18	6.38
3809	1.41, 3	5	0	40	0	20	20	.80	5.64	4.86	5.96	4.80	4.94	4.30	5.88	4.44	4.68	4.08
3810	1.41, 3	5	+	40	0.16	17	23	.80	3.84	4.72	3.26	2.58	2.70	3.96	3.56	2.68	2.72	4.12
3811	1.41, 3	5	-	40	0.16	23	17	.80	8.08	5.70	9.76	7.94	8.10	4.82	9.50	7.70	7.82	4.28
3812	1.41, 3	5	+	40	0.33	13	27	.80	1.70	4.04	1.32	.88	.90	4.10	1.66	1.30	1.40	4.72
3813	1.41, 3	5	-	40	0.33	27	13	.80	13.00	6.22	15.54	13.46	13.60	5.04	14.66	12.68	12.82	4.74
3814	1.41, 3	5	+	40	0.50	10	30	.80	1.04	4.28	.44	.32	.34	4.80	.80	.54	.56	5.00
3815	1.41, 3	5	-	40	0.50	30	10	.80	16.90	7.52	21.80	19.36	19.60	5.48	21.32	18.58	18.90	5.22
3816	1.41, 3	5	0	60	0	30	30	.80	5.42	4.94	6.46	5.34	5.44	5.14	5.70	4.62	4.70	4.46
3817	1.41, 3	5	+	60	0.16	25	35	.80	3.02	4.74	3.14	2.60	2.66	4.68	3.24	2.48	2.54	4.60
3818	1.41, 3	5	-	60	0.16	35	25	.80	7.90	5.04	10.32	8.38	8.52	5.06	10.38	8.70	8.80	5.04
3819	1.41, 3	5	+	60	0.33	20	40	.80	1.80	4.74	1.82	1.40	1.44	4.60	1.78	1.14	1.18	4.66
3820	1.41, 3	5	-	60	0.33	40	20	.80	12.06	5.76	15.46	13.12	13.34	4.94	15.02	12.72	12.92	4.72
3821	1.41, 3	5	+	60	0.50	15	45	.80	.92	4.92	.70	.36	.40	4.70	.86	.68	.74	4.90
3822	1.41, 3	5	-	60	0.50	45	15	.80	16.88	6.50	22.10	19.44	19.64	5.54	21.56	18.96	19.18	5.16
3823	1.41, 3	5	0	80	0	40	40	.80	5.02	4.92	6.62	5.28	5.32	5.12	5.72	4.60	4.68	4.42
3824	1.41, 3	5	+	80	0.16	34	46	.80	2.88	4.46	4.06	3.08	3.08	5.48	3.76	3.12	3.12	4.74
3825	1.41, 3	5	-	80	0.16	46	34	.80	8.06	4.96	10.16	8.74	8.80	5.04	9.38	7.82	8.00	5.16
3826	1.41, 3	5	+	80	0.33	27	53	.80	1.70	4.60	1.84	1.32	1.40	5.40	1.66	1.26	1.30	4.54
3827	1.41, 3	5	-	80	0.33	53	27	.80	11.66	5.70	14.98	12.96	13.12	5.48	15.68	13.52	13.58	4.98
3828	1.41, 3	5	+	80	0.50	20	60	.80	.84	4.98	.72	.50	.50	4.96	.56	.42	.42	5.04
3829	1.41, 3	5	-	80	0.50	60	20	.80	17.08	6.06	22.86	20.26	20.38	5.36	22.08	19.18	19.50	5.20
3830	1.41, 3	5	0	100	0	50	50	.80	5.14	5.02	6.40	4.82	4.92	4.90	6.16	5.02	5.14	4.84
3831	1.41, 3	5	+	100	0.16	42	58	.80	3.10	4.68	3.54	2.72	2.76	4.86	3.38	2.72	2.74	4.66
3832	1.41, 3	5	-	100	0.16	58	42	.80	8.32	5.24	10.20	8.46	8.64	4.74	10.02	8.16	8.24	4.96
3833	1.41, 3	5	+	100	0.33	33	67	.80	1.40	4.58	1.60	1.12	1.12	4.80	1.54	1.20	1.24	4.82
3834	1.41, 3	5	-	100	0.33	67	33	.80	12.70	5.80	15.62	13.58	13.68	4.72	15.84	13.58	13.68	4.32
3835	1.41, 3	5	+	100	0.50	25	75	.80	.74	4.40	.66	.46	.46	4.94	.48	.40	.40	4.68
3836	1.41, 3	5	-	100	0.50	75	25	.80	18.12	6.00	21.54	19.24	19.38	4.84	21.08	18.78	18.92	4.72
3837	1.41, 3	5	0	150	0	75	75	.80	5.16	4.96	6.42	5.36	5.36	5.38	6.86	5.68	5.68	5.68
3838	1.41, 3	5	+	150	0.16	63	87	.80	3.44	5.30	3.78	2.90	2.94	5.78	3.62	2.84	2.88	5.28

	D	VR	P	N	Δn	n1	n2	ε	F g	B-F g	F t	F-GG t	F-HF t	B-F t	F g x t	F-GG g x t	F-HF g x t	B-F g x t
3839	1.41, 3	5	-	150	0.16	87	63	.80	8.06	5.02	10.24	8.90	8.94	5.40	9.72	8.24	8.28	5.22
3840	1.41, 3	5	+	150	0.33	50	100	.80	1.70	4.88	1.58	1.18	1.22	5.74	1.52	1.10	1.12	5.14
3841	1.41, 3	5	-	150	0.33	100	50	.80	11.60	5.00	15.42	13.28	13.30	5.28	15.38	13.28	13.34	5.28
3842	1.41, 3	5	+	150	0.50	37	113	.80	.54	4.80	.66	.50	.50	5.68	.54	.36	.36	4.76
3843	1.41, 3	5	-	150	0.50	113	37	.80	17.18	6.24	22.62	19.94	20.06	5.30	21.52	19.38	19.48	5.32
3844	1.41, 3	5	0	200	0	100	100	.80	5.06	5.12	6.52	5.28	5.36	5.22	6.36	5.18	5.22	5.16
3845	1.41, 3	5	+	200	0.16	84	116	.80	2.94	5.02	3.60	2.88	2.92	5.10	3.68	2.80	2.80	5.34
3846	1.41, 3	5	-	200	0.16	116	84	.80	8.46	5.30	10.42	8.82	8.88	5.46	10.56	9.30	9.32	5.54
3847	1.41, 3	5	+	200	0.33	67	133	.80	1.58	4.88	1.66	1.34	1.36	5.32	1.76	1.32	1.34	5.26
3848	1.41, 3	5	-	200	0.33	133	67	.80	12.02	5.34	15.78	13.82	14.00	5.56	16.02	13.84	13.94	5.52
3849	1.41, 3	5	+	200	0.50	50	150	.80	.56	4.86	.68	.46	.48	5.28	.50	.26	.26	5.08
3850	1.41, 3	5	-	200	0.50	150	50	.80	16.94	5.40	22.16	20.28	20.38	5.90	23.12	20.72	20.80	5.18
3851	1.41, 3	1	0	20	0	10	10	.90	4.02	3.58	5.68	4.68	5.04	4.36	5.20	4.46	4.74	4.12
3852	1.41, 3	1	0	20	0.16	8	12	.90	4.62	4.04	5.82	4.64	4.94	4.04	4.84	3.74	4.14	3.80
3853	1.41, 3	1	0	20	0.33	7	13	.90	4.90	4.72	5.62	4.84	5.04	4.42	5.00	4.20	4.44	3.94
3854	1.41, 3	1	0	20	0.50	5	15	.90	4.78	7.28	5.64	4.70	5.08	5.90	5.02	4.18	4.50	6.08
3855	1.41, 3	1	0	40	0	20	20	.90	4.96	4.60	5.58	4.78	5.02	4.70	5.34	4.40	4.66	4.26
3856	1.41, 3	1	0	40	0.16	17	23	.90	4.84	4.52	5.20	4.36	4.56	4.28	4.98	4.44	4.58	4.36
3857	1.41, 3	1	0	40	0.33	13	27	.90	4.50	4.64	5.40	4.54	4.74	4.76	5.08	4.38	4.56	4.76
3858	1.41, 3	1	0	40	0.50	10	30	.90	4.36	5.94	5.18	4.60	4.72	5.50	5.50	4.82	5.00	5.36
3859	1.41, 3	1	0	60	0	30	30	.90	4.62	4.36	5.50	4.78	4.90	4.56	5.44	4.60	4.76	4.78
3860	1.41, 3	1	0	60	0.16	25	35	.90	4.68	4.84	5.56	4.88	4.98	4.76	5.46	4.78	4.90	4.78
3861	1.41, 3	1	0	60	0.33	20	40	.90	5.18	5.40	5.50	4.94	5.02	5.00	5.06	4.58	4.74	4.66
3862	1.41, 3	1	0	60	0.50	15	45	.90	4.88	5.86	5.80	5.10	5.26	5.22	5.44	4.72	4.84	5.34
3863	1.41, 3	1	0	80	0	40	40	.90	4.30	4.20	6.46	5.68	5.78	5.48	5.16	4.56	4.74	4.72
3864	1.41, 3	1	0	80	0.16	34	46	.90	4.52	4.64	6.24	5.68	5.78	5.46	5.34	4.84	4.92	4.86
3865	1.41, 3	1	0	80	0.33	27	53	.90	5.06	5.28	5.96	5.44	5.48	5.84	5.00	4.30	4.48	4.76
3866	1.41, 3	1	0	80	0.50	20	60	.90	5.18	6.18	5.96	5.44	5.56	5.60	5.24	4.62	4.74	5.12
3867	1.41, 3	1	0	100	0	50	50	.90	4.94	4.90	5.72	5.18	5.32	5.16	4.88	4.24	4.30	4.34
3868	1.41, 3	1	0	100	0.16	42	58	.90	4.70	4.96	6.00	5.28	5.36	5.42	5.26	4.68	4.72	4.96
3869	1.41, 3	1	0	100	0.33	33	67	.90	4.82	5.26	5.70	5.22	5.24	5.52	5.62	5.00	5.12	5.22
3870	1.41, 3	1	0	100	0.50	25	75	.90	4.66	5.50	5.92	4.96	5.08	5.36	5.02	4.44	4.52	4.88

	D	VR	P	N	Δn	n1	n2	ε	F g	B-F g	F t	F-GG t	F-HF t	B-F t	F g x t	F-GG g x t	F-HF g x t	B-F g x t
3871	1.41, 3	1	0	150	0	75	75	.90	5.74	5.72	5.80	5.26	5.28	5.32	6.04	5.26	5.26	5.52
3872	1.41, 3	1	0	150	0.16	63	87	.90	5.58	5.82	5.56	4.94	5.02	5.20	5.56	5.10	5.12	5.34
3873	1.41, 3	1	0	150	0.33	50	100	.90	4.88	5.12	5.66	5.12	5.18	5.52	4.98	4.52	4.56	5.00
3874	1.41, 3	1	0	150	0.50	37	113	.90	5.30	5.52	5.92	5.26	5.32	5.76	5.28	4.86	4.96	5.24
3875	1.41, 3	1	0	200	0	100	100	.90	5.06	5.04	5.98	5.24	5.30	5.34	5.66	5.22	5.24	5.28
3876	1.41, 3	1	0	200	0.16	84	116	.90	5.20	5.44	5.82	5.38	5.42	5.56	5.86	5.24	5.30	5.42
3877	1.41, 3	1	0	200	0.33	67	133	.90	4.88	5.28	6.16	5.50	5.54	5.60	6.00	5.24	5.26	5.30
3878	1.41, 3	1	0	200	0.50	50	150	.90	4.74	5.28	5.98	5.38	5.38	5.86	4.98	4.58	4.62	4.90
3879	1.41, 3	1.5	0	20	0	10	10	.90	4.04	3.52	5.72	4.70	5.06	4.34	5.32	4.36	4.66	4.08
3880	1.41, 3	1.5	+	20	0.16	8	12	.90	3.78	3.82	4.90	3.88	4.24	4.12	4.02	3.32	3.48	3.58
3881	1.41, 3	1.5	-	20	0.16	12	8	.90	5.60	4.78	6.66	5.50	5.84	4.22	6.44	5.28	5.60	3.92
3882	1.41, 3	1.5	+	20	0.33	7	13	.90	3.98	4.66	4.46	3.48	3.84	4.54	3.86	3.04	3.34	3.96
3883	1.41, 3	1.5	-	20	0.33	13	7	.90	6.00	5.48	7.30	6.12	6.54	4.96	7.24	5.96	6.32	4.58
3884	1.41, 3	1.5	+	20	0.50	5	15	.90	3.22	6.16	3.60	2.70	2.98	5.28	3.10	2.62	2.76	5.30
3885	1.41, 3	1.5	-	20	0.50	15	5	.90	7.44	8.40	8.50	7.22	7.76	6.58	8.94	7.50	7.94	6.52
3886	1.41, 3	1.5	0	40	0	20	20	.90	5.02	4.60	5.78	5.04	5.20	4.74	5.36	4.58	4.84	4.30
3887	1.41, 3	1.5	+	40	0.16	17	23	.90	4.24	4.42	4.52	3.86	4.08	4.38	4.16	3.44	3.56	4.18
3888	1.41, 3	1.5	-	40	0.16	23	17	.90	5.70	4.88	6.94	5.92	6.14	4.92	6.24	5.38	5.52	4.84
3889	1.41, 3	1.5	+	40	0.33	13	27	.90	3.26	4.36	3.54	3.12	3.26	4.70	3.58	3.04	3.22	4.42
3890	1.41, 3	1.5	-	40	0.33	27	13	.90	6.20	5.38	8.38	7.20	7.50	5.02	7.16	6.22	6.42	4.74
3891	1.41, 3	1.5	+	40	0.50	10	30	.90	2.72	5.26	3.10	2.66	2.76	5.38	3.14	2.74	2.82	5.20
3892	1.41, 3	1.5	-	40	0.50	30	10	.90	7.36	6.86	8.98	7.92	8.24	6.18	8.42	7.48	7.76	6.08
3893	1.41, 3	1.5	0	60	0	30	30	.90	4.38	4.48	5.50	4.88	4.96	4.90	5.52	4.80	4.98	4.88
3894	1.41, 3	1.5	+	60	0.16	25	35	.90	4.10	4.42	4.48	3.86	4.00	4.76	4.64	4.08	4.20	4.74
3895	1.41, 3	1.5	-	60	0.16	35	25	.90	5.48	4.88	6.54	5.64	5.88	4.88	6.50	5.82	5.98	5.30
3896	1.41, 3	1.5	+	60	0.33	20	40	.90	3.60	5.12	3.92	3.38	3.46	4.84	3.56	3.08	3.22	4.76
3897	1.41, 3	1.5	-	60	0.33	40	20	.90	6.42	5.46	7.82	6.76	6.90	5.04	7.22	6.26	6.36	5.18
3898	1.41, 3	1.5	+	60	0.50	15	45	.90	3.28	5.38	3.34	3.02	3.06	5.06	3.18	2.78	2.84	5.00
3899	1.41, 3	1.5	-	60	0.50	45	15	.90	7.66	6.18	9.36	8.18	8.56	5.52	8.98	8.00	8.22	5.48
3900	1.41, 3	1.5	0	80	0	40	40	.90	4.50	4.26	6.56	5.72	5.84	5.56	5.28	4.52	4.58	4.50
3901	1.41, 3	1.5	+	80	0.16	34	46	.90	3.86	4.58	5.54	5.04	5.08	5.82	4.90	4.36	4.42	5.34
3902	1.41, 3	1.5	-	80	0.16	46	34	.90	5.68	5.20	7.50	6.62	6.74	5.66	6.22	5.60	5.70	4.86

	D	VR	P	N	Δn	n1	n2	ε	F g	B-F g	F t	F-GG t	F-HF t	B-F t	F g x t	F-GG g x t	F-HF g x t	B-F g x t
3903	1.41, 3	1.5	+	80	0.33	27	53	.90	3.56	5.06	4.34	3.90	3.90	5.98	3.36	3.00	3.04	4.78
3904	1.41, 3	1.5	-	80	0.33	53	27	.90	6.44	5.04	8.76	7.86	8.00	5.94	7.48	6.66	6.86	4.56
3905	1.41, 3	1.5	+	80	0.50	20	60	.90	3.18	6.02	3.74	3.10	3.14	5.76	2.90	2.52	2.60	5.10
3906	1.41, 3	1.5	-	80	0.50	60	20	.90	7.50	5.76	10.24	9.24	9.34	5.74	8.90	7.82	8.02	5.00
3907	1.41, 3	1.5	0	100	0	50	50	.90	4.98	4.96	5.62	4.98	5.04	4.98	5.02	4.44	4.54	4.72
3908	1.41, 3	1.5	+	100	0.16	42	58	.90	4.16	5.06	5.06	4.26	4.32	5.28	4.18	3.52	3.60	4.78
3909	1.41, 3	1.5	-	100	0.16	58	42	.90	6.06	5.24	6.38	5.80	5.90	5.04	6.42	5.68	5.78	4.78
3910	1.41, 3	1.5	+	100	0.33	33	67	.90	3.34	4.74	4.10	3.58	3.66	5.28	3.92	3.40	3.42	5.22
3911	1.41, 3	1.5	-	100	0.33	67	33	.90	7.28	5.36	7.94	6.90	7.00	5.02	7.76	6.92	7.08	4.58
3912	1.41, 3	1.5	+	100	0.50	25	75	.90	2.90	5.24	3.20	2.66	2.68	5.46	2.78	2.32	2.38	4.70
3913	1.41, 3	1.5	-	100	0.50	75	25	.90	7.94	6.30	8.54	7.84	7.90	5.04	9.38	8.52	8.62	5.16
3914	1.41, 3	1.5	0	150	0	75	75	.90	5.70	5.58	5.88	5.38	5.40	5.32	5.92	5.26	5.34	5.64
3915	1.41, 3	1.5	+	150	0.16	63	87	.90	4.82	5.86	4.88	4.44	4.46	5.50	4.60	4.00	4.04	5.34
3916	1.41, 3	1.5	-	150	0.16	87	63	.90	6.32	5.58	7.12	6.32	6.46	5.56	6.66	5.80	5.92	4.88
3917	1.41, 3	1.5	+	150	0.33	50	100	.90	3.58	5.02	4.04	3.64	3.68	5.34	3.54	3.28	3.30	4.84
3918	1.41, 3	1.5	-	150	0.33	100	50	.90	6.54	5.34	8.08	7.38	7.50	5.64	7.58	6.88	7.04	5.22
3919	1.41, 3	1.5	+	150	0.50	37	113	.90	2.86	5.56	3.32	2.94	2.98	5.74	3.14	2.64	2.72	5.20
3920	1.41, 3	1.5	-	150	0.50	113	37	.90	7.66	5.76	9.14	8.50	8.56	5.32	8.60	8.02	8.10	5.24
3921	1.41, 3	1.5	0	200	0	100	100	.90	5.22	5.32	5.68	5.00	5.00	5.18	5.84	5.36	5.38	5.48
3922	1.41, 3	1.5	+	200	0.16	84	116	.90	4.38	5.34	5.00	4.62	4.64	5.34	4.70	4.20	4.26	5.10
3923	1.41, 3	1.5	-	200	0.16	116	84	.90	5.88	5.18	6.60	6.04	6.10	5.18	6.66	5.88	5.92	5.14
3924	1.41, 3	1.5	+	200	0.33	67	133	.90	3.72	5.18	4.18	3.64	3.68	5.72	4.04	3.46	3.58	5.32
3925	1.41, 3	1.5	-	200	0.33	133	67	.90	6.58	5.44	8.02	7.30	7.32	5.08	8.26	7.42	7.44	5.40
3926	1.41, 3	1.5	+	200	0.50	50	150	.90	2.94	5.28	3.44	2.98	2.98	5.62	2.76	2.48	2.50	4.94
3927	1.41, 3	1.5	-	200	0.50	150	50	.90	7.68	5.62	9.72	8.90	8.94	5.50	9.66	8.70	8.72	5.58
3928	1.41, 3	2	0	20	0	10	10	.90	4.16	3.70	5.74	4.78	5.08	4.04	5.34	4.30	4.72	3.74
3929	1.41, 3	2	+	20	0.16	8	12	.90	3.68	3.74	4.28	3.42	3.68	4.36	3.60	2.86	3.08	3.52
3930	1.41, 3	2	-	20	0.16	12	8	.90	6.62	5.20	7.74	6.50	6.90	4.56	7.68	6.10	6.66	4.12
3931	1.41, 3	2	+	20	0.33	7	13	.90	3.56	4.60	3.58	2.48	2.90	4.46	3.16	2.54	2.80	3.86
3932	1.41, 3	2	-	20	0.33	13	7	.90	7.28	6.16	8.92	7.56	7.90	5.22	8.54	7.22	7.70	4.68
3933	1.41, 3	2	+	20	0.50	5	15	.90	2.50	5.44	2.34	1.74	2.02	5.12	2.22	1.76	1.94	4.98
3934	1.41, 3	2	-	20	0.50	15	5	.90	10.00	9.10	11.50	9.92	10.44	6.78	11.66	10.22	10.70	6.54

	D	VR	P	N	Δn	n1	n2	ε	F g	B-F g	F t	F-GG t	F-HF t	B-F t	F g x t	F-GG g x t	F-HF g x t	B-F g x t
3935	1.41, 3	2	0	40	0	20	20	.90	5.10	4.68	5.80	5.10	5.32	4.62	5.28	4.70	4.88	4.38
3936	1.41, 3	2	+	40	0.16	17	23	.90	3.86	4.26	4.16	3.42	3.66	4.36	3.88	3.22	3.38	4.04
3937	1.41, 3	2	-	40	0.16	23	17	.90	6.16	4.98	7.82	6.70	6.94	4.86	6.98	5.94	6.26	4.66
3938	1.41, 3	2	+	40	0.33	13	27	.90	2.48	4.44	2.68	2.08	2.22	4.34	2.64	2.12	2.26	4.46
3939	1.41, 3	2	-	40	0.33	27	13	.90	7.70	5.54	10.02	8.94	9.30	4.98	8.96	7.82	8.02	4.94
3940	1.41, 3	2	+	40	0.50	10	30	.90	2.00	4.84	1.98	1.60	1.74	5.06	2.26	1.78	1.86	5.04
3941	1.41, 3	2	-	40	0.50	30	10	.90	9.56	7.16	11.74	10.54	10.78	6.22	11.44	10.20	10.52	6.20
3942	1.41, 3	2	0	60	0	30	30	.90	4.52	4.38	5.62	4.84	4.92	4.68	5.60	4.66	4.82	4.76
3943	1.41, 3	2	+	60	0.16	25	35	.90	3.64	4.44	4.04	3.38	3.50	4.70	4.16	3.60	3.68	4.70
3944	1.41, 3	2	-	60	0.16	35	25	.90	6.24	4.70	7.48	6.70	6.88	5.00	7.22	6.32	6.52	5.32
3945	1.41, 3	2	+	60	0.33	20	40	.90	2.86	4.92	3.08	2.72	2.86	4.76	2.86	2.46	2.50	4.56
3946	1.41, 3	2	-	60	0.33	40	20	.90	7.90	5.62	9.66	8.42	8.76	5.16	9.08	8.12	8.32	5.18
3947	1.41, 3	2	+	60	0.50	15	45	.90	2.40	5.14	2.22	1.82	1.84	5.02	1.90	1.74	1.76	4.78
3948	1.41, 3	2	-	60	0.50	45	15	.90	9.76	6.32	12.38	11.20	11.50	5.78	12.04	10.94	11.22	5.48
3949	1.41, 3	2	0	80	0	40	40	.90	4.60	4.76	6.50	5.58	5.80	5.52	5.42	4.74	4.86	4.56
3950	1.41, 3	2	+	80	0.16	34	46	.90	3.56	4.50	5.02	4.30	4.42	5.96	4.38	3.84	3.96	5.28
3951	1.41, 3	2	-	80	0.16	46	34	.90	6.10	5.24	8.18	7.38	7.50	5.68	6.90	6.12	6.24	4.96
3952	1.41, 3	2	+	80	0.33	27	53	.90	2.90	4.92	3.38	2.96	2.98	5.80	2.70	2.32	2.36	4.70
3953	1.41, 3	2	-	80	0.33	53	27	.90	7.60	5.28	10.58	9.56	9.70	5.80	9.06	8.38	8.50	4.68
3954	1.41, 3	2	+	80	0.50	20	60	.90	2.24	5.86	2.38	2.02	2.10	5.70	2.00	1.80	1.84	5.02
3955	1.41, 3	2	-	80	0.50	60	20	.90	9.48	5.88	13.28	12.24	12.48	5.74	12.04	10.78	10.92	5.00
3956	1.41, 3	2	0	100	0	50	50	.90	4.94	4.90	5.70	5.06	5.16	5.00	5.00	4.42	4.54	4.64
3957	1.41, 3	2	+	100	0.16	42	58	.90	3.90	5.10	4.32	3.76	3.80	5.12	3.66	3.20	3.26	4.60
3958	1.41, 3	2	-	100	0.16	58	42	.90	6.56	5.50	7.46	6.52	6.58	5.08	7.22	6.26	6.36	5.02
3959	1.41, 3	2	+	100	0.33	33	67	.90	2.60	4.80	3.18	2.68	2.76	5.14	2.84	2.38	2.46	4.96
3960	1.41, 3	2	-	100	0.33	67	33	.90	8.42	5.82	9.62	8.70	8.82	5.32	9.66	8.78	8.86	5.08
3961	1.41, 3	2	+	100	0.50	25	75	.90	1.98	4.98	2.22	1.70	1.78	5.52	1.74	1.42	1.46	4.70
3962	1.41, 3	2	-	100	0.50	75	25	.90	10.28	6.74	11.98	10.82	11.06	5.10	12.10	11.10	11.22	5.54
3963	1.41, 3	2	0	150	0	75	75	.90	5.64	5.68	5.98	5.28	5.44	5.68	5.90	5.34	5.40	5.58
3964	1.41, 3	2	+	150	0.16	63	87	.90	4.40	5.68	4.36	3.80	3.94	5.46	3.96	3.58	3.58	5.28
3965	1.41, 3	2	-	150	0.16	87	63	.90	6.66	5.38	7.76	7.00	7.04	5.32	7.46	6.62	6.76	4.76
3966	1.41, 3	2	+	150	0.33	50	100	.90	2.90	4.88	3.02	2.54	2.58	5.34	2.88	2.52	2.58	4.84

	D	VR	P	N	Δn	n1	n2	ε	F g	B-F g	F t	F-GG t	F-HF t	B-F t	F g x t	F-GG g x t	F-HF g x t	B-F g x t
3967	1.41, 3	2	-	150	0.33	100	50	.90	7.94	5.34	9.74	8.92	9.02	5.66	9.28	8.46	8.50	5.24
3968	1.41, 3	2	+	150	0.50	37	113	.90	1.96	5.46	2.18	1.72	1.76	5.68	1.96	1.72	1.74	5.04
3969	1.41, 3	2	-	150	0.50	113	37	.90	9.66	5.72	11.86	10.94	11.02	5.26	11.30	10.38	10.44	5.20
3970	1.41, 3	2	0	200	0	100	100	.90	5.30	5.34	5.62	5.06	5.10	5.22	5.84	5.32	5.32	5.32
3971	1.41, 3	2	+	200	0.16	84	116	.90	3.92	5.40	4.42	3.88	3.94	5.34	4.16	3.74	3.76	5.02
3972	1.41, 3	2	-	200	0.16	116	84	.90	6.42	5.18	7.40	6.72	6.82	5.16	7.44	6.66	6.70	5.34
3973	1.41, 3	2	+	200	0.33	67	133	.90	3.02	5.12	3.10	2.80	2.80	5.48	2.90	2.62	2.66	5.36
3974	1.41, 3	2	-	200	0.33	133	67	.90	7.96	5.24	10.18	9.28	9.32	5.18	10.06	9.06	9.16	5.56
3975	1.41, 3	2	+	200	0.50	50	150	.90	2.12	5.12	2.22	1.78	1.78	5.80	1.94	1.78	1.80	4.96
3976	1.41, 3	2	-	200	0.50	150	50	.90	10.16	6.00	12.64	11.52	11.60	5.46	12.26	11.54	11.62	5.66
3977	1.41, 3	5	0	20	0	10	10	.90	5.72	4.92	6.40	5.04	5.52	4.34	5.92	4.78	5.18	3.80
3978	1.41, 3	5	+	20	0.16	8	12	.90	3.68	4.62	2.98	2.20	2.48	4.00	2.66	2.16	2.32	3.72
3979	1.41, 3	5	-	20	0.16	12	8	.90	9.80	6.24	11.14	9.32	10.02	4.44	10.94	9.02	9.54	4.08
3980	1.41, 3	5	+	20	0.33	7	13	.90	2.86	4.76	1.74	1.38	1.50	4.38	1.74	1.28	1.34	3.88
3981	1.41, 3	5	-	20	0.33	13	7	.90	12.50	7.28	14.54	12.40	13.20	5.14	14.26	11.88	12.54	4.94
3982	1.41, 3	5	+	20	0.50	5	15	.90	1.58	4.60	.54	.42	.44	4.48	.62	.46	.52	3.94
3983	1.41, 3	5	-	20	0.50	15	5	.90	18.58	10.26	22.84	19.94	20.84	6.98	22.78	19.96	20.80	6.76
3984	1.41, 3	5	0	40	0	20	20	.90	5.62	4.92	6.06	4.94	5.22	4.28	5.66	4.92	5.06	4.32
3985	1.41, 3	5	+	40	0.16	17	23	.90	3.62	4.72	2.98	2.32	2.50	4.74	3.04	2.34	2.60	4.10
3986	1.41, 3	5	-	40	0.16	23	17	.90	8.20	5.32	9.76	8.68	8.84	4.90	9.42	8.08	8.32	4.60
3987	1.41, 3	5	+	40	0.33	13	27	.90	1.66	4.20	1.00	.86	.90	4.78	1.12	1.00	1.02	4.42
3988	1.41, 3	5	-	40	0.33	27	13	.90	12.48	6.02	16.20	14.48	14.92	5.42	15.24	13.34	13.74	5.32
3989	1.41, 3	5	+	40	0.50	10	30	.90	.94	4.44	.50	.44	.44	5.02	.60	.54	.56	4.68
3990	1.41, 3	5	-	40	0.50	30	10	.90	17.16	7.30	22.42	20.32	20.76	5.86	22.04	20.12	20.58	6.20
3991	1.41, 3	5	0	60	0	30	30	.90	4.88	4.38	5.74	4.88	5.04	4.60	5.64	4.84	4.98	4.86
3992	1.41, 3	5	+	60	0.16	25	35	.90	3.10	4.66	3.00	2.52	2.54	4.52	2.86	2.36	2.42	4.46
3993	1.41, 3	5	-	60	0.16	35	25	.90	8.26	5.02	10.04	8.88	9.12	5.12	9.84	8.68	8.86	5.52
3994	1.41, 3	5	+	60	0.33	20	40	.90	1.76	4.76	1.52	1.26	1.32	4.56	1.42	1.16	1.24	4.66
3995	1.41, 3	5	-	60	0.33	40	20	.90	11.80	5.48	15.82	14.28	14.58	4.70	15.36	14.04	14.28	4.78
3996	1.41, 3	5	+	60	0.50	15	45	.90	.88	4.66	.46	.46	.46	4.66	.48	.40	.42	4.30
3997	1.41, 3	5	-	60	0.50	45	15	.90	17.04	6.54	22.64	20.86	21.30	5.24	22.70	20.76	21.04	5.46
3998	1.41, 3	5	0	80	0	40	40	.90	5.10	4.48	6.30	5.54	5.62	5.42	5.64	4.94	5.06	4.78

	D	VR	P	N	Δn	n1	n2	ε	F g	B-F g	F t	F-GG t	F-HF t	B-F t	F g x t	F-GG g x t	F-HF g x t	B-F g x t
3999	1.41, 3	5	+	80	0.16	34	46	.90	2.84	4.18	3.78	3.18	3.22	5.70	3.28	2.94	3.00	5.28
4000	1.41, 3	5	-	80	0.16	46	34	.90	8.20	5.20	10.48	9.42	9.64	5.56	9.16	8.08	8.28	4.88
4001	1.41, 3	5	+	80	0.33	27	53	.90	1.88	4.68	1.76	1.44	1.50	5.60	1.30	1.16	1.20	4.62
4002	1.41, 3	5	-	80	0.33	53	27	.90	11.72	5.24	15.94	14.76	14.90	5.44	15.12	13.64	13.84	4.72
4003	1.41, 3	5	+	80	0.50	20	60	.90	.88	5.16	.64	.40	.44	5.78	.30	.26	.28	4.96
4004	1.41, 3	5	-	80	0.50	60	20	.90	16.48	6.22	24.20	22.18	22.34	5.42	22.98	21.08	21.34	5.06
4005	1.41, 3	5	0	100	0	50	50	.90	5.20	4.92	6.18	5.28	5.42	5.16	5.34	4.62	4.70	4.68
4006	1.41, 3	5	+	100	0.16	42	58	.90	2.92	4.92	3.26	2.84	2.88	4.92	2.72	2.34	2.44	4.32
4007	1.41, 3	5	-	100	0.16	58	42	.90	8.32	5.50	9.60	8.56	8.72	5.28	9.58	8.64	8.74	4.58
4008	1.41, 3	5	+	100	0.33	33	67	.90	1.50	4.50	1.58	1.14	1.22	4.96	1.22	.98	1.10	4.68
4009	1.41, 3	5	-	100	0.33	67	33	.90	12.34	5.72	15.68	14.10	14.22	5.26	16.00	14.78	14.92	4.70
4010	1.41, 3	5	+	100	0.50	25	75	.90	.68	4.44	.42	.36	.36	5.28	.40	.34	.36	4.38
4011	1.41, 3	5	-	100	0.50	75	25	.90	17.36	6.80	21.86	20.26	20.44	5.10	22.52	20.76	20.98	5.50
4012	1.41, 3	5	0	150	0	75	75	.90	5.64	5.62	5.90	5.20	5.24	5.30	5.84	5.16	5.24	5.48
4013	1.41, 3	5	+	150	0.16	63	87	.90	3.50	5.46	2.88	2.52	2.54	5.54	2.86	2.56	2.56	4.96
4014	1.41, 3	5	-	150	0.16	87	63	.90	8.52	5.36	9.66	8.70	8.78	5.16	9.60	8.70	8.74	4.96
4015	1.41, 3	5	+	150	0.33	50	100	.90	1.54	4.90	1.42	1.20	1.24	5.20	1.30	1.04	1.04	5.32
4016	1.41, 3	5	-	150	0.33	100	50	.90	11.72	5.42	15.38	14.16	14.28	5.28	14.86	13.58	13.72	5.42
4017	1.41, 3	5	+	150	0.50	37	113	.90	.66	4.94	.58	.52	.52	5.48	.46	.42	.44	4.80
4018	1.41, 3	5	-	150	0.50	113	37	.90	17.30	5.76	22.66	20.80	21.04	5.06	21.76	20.32	20.42	5.44
4019	1.41, 3	5	0	200	0	100	100	.90	5.24	5.22	5.70	5.18	5.20	5.12	5.80	5.32	5.34	5.12
4020	1.41, 3	5	+	200	0.16	84	116	.90	3.24	5.06	3.22	2.98	3.00	4.88	3.12	2.74	2.74	5.18
4021	1.41, 3	5	-	200	0.16	116	84	.90	8.04	5.16	9.64	8.62	8.72	5.30	9.52	8.66	8.70	5.24
4022	1.41, 3	5	+	200	0.33	67	133	.90	1.82	4.96	1.52	1.22	1.24	5.48	1.40	1.18	1.20	5.20
4023	1.41, 3	5	-	200	0.33	133	67	.90	12.06	5.40	16.12	14.86	14.94	5.46	15.82	14.72	14.80	5.82
4024	1.41, 3	5	+	200	0.50	50	150	.90	.62	4.90	.52	.44	.44	5.48	.34	.26	.28	4.88
4025	1.41, 3	5	-	200	0.50	150	50	.90	17.12	5.90	23.10	21.72	21.84	5.50	23.06	21.64	21.78	5.54
4026	1.41, 3	1	0	20	0	10	10	1	3.96	3.50	4.44	3.82	4.00	3.54	4.88	4.08	4.50	3.66
4027	1.41, 3	1	0	20	0.16	8	12	1	4.52	4.06	4.62	3.88	4.28	3.74	4.62	3.98	4.32	3.64
4028	1.41, 3	1	0	20	0.33	7	13	1	4.98	5.00	4.68	3.98	4.44	4.14	5.04	4.18	4.66	4.26
4029	1.41, 3	1	0	20	0.50	5	15	1	4.74	6.90	5.40	4.64	4.96	5.24	5.20	4.44	4.90	4.96
4030	1.41, 3	1	0	40	0	20	20	1	5.08	4.52	4.72	4.20	4.32	4.32	4.54	4.16	4.36	3.76

	D	VR	P	N	Δn	n1	n2	ε	F g	B-F g	F t	F-GG t	F-HF t	B-F t	F g x t	F-GG g x t	F-HF g x t	B-F g x t
4031	1.41, 3	1	0	40	0.16	17	23	1	4.96	4.84	4.72	4.26	4.42	4.28	4.54	4.32	4.44	4.40
4032	1.41, 3	1	0	40	0.33	13	27	1	4.52	5.04	5.00	4.56	4.78	4.70	4.94	4.66	4.82	4.70
4033	1.41, 3	1	0	40	0.50	10	30	1	4.28	5.78	4.98	4.38	4.60	5.50	5.14	4.72	4.92	5.28
4034	1.41, 3	1	0	60	0	30	30	1	4.80	4.58	5.00	4.64	4.76	4.74	5.16	4.86	5.04	4.60
4035	1.41, 3	1	0	60	0.16	25	35	1	4.74	4.68	5.40	5.22	5.28	4.96	4.68	4.42	4.56	4.34
4036	1.41, 3	1	0	60	0.33	20	40	1	5.06	5.62	5.26	5.06	5.16	5.04	5.20	5.02	5.12	4.84
4037	1.41, 3	1	0	60	0.50	15	45	1	4.90	5.94	4.88	4.68	4.74	5.10	5.00	4.78	4.88	5.06
4038	1.41, 3	1	0	80	0	40	40	1	4.54	4.50	5.94	5.62	5.82	5.52	4.70	4.56	4.64	4.56
4039	1.41, 3	1	0	80	0.16	34	46	1	4.82	4.90	5.42	5.20	5.24	5.14	5.12	4.94	5.06	5.12
4040	1.41, 3	1	0	80	0.33	27	53	1	5.06	5.00	5.12	4.82	4.94	5.06	4.70	4.48	4.66	4.46
4041	1.41, 3	1	0	80	0.50	20	60	1	5.12	6.20	5.38	5.12	5.26	5.42	4.78	4.64	4.70	4.62
4042	1.41, 3	1	0	100	0	50	50	1	5.20	5.24	5.12	5.00	5.04	5.26	4.94	4.84	4.90	4.80
4043	1.41, 3	1	0	100	0.16	42	58	1	4.92	5.02	5.36	5.16	5.22	5.40	4.70	4.56	4.62	4.64
4044	1.41, 3	1	0	100	0.33	33	67	1	4.76	5.30	5.08	4.84	5.00	5.06	5.24	5.02	5.16	5.38
4045	1.41, 3	1	0	100	0.50	25	75	1	4.64	5.54	5.02	4.90	4.96	5.00	4.38	4.18	4.28	4.64
4046	1.41, 3	1	0	150	0	75	75	1	5.64	5.70	5.10	4.94	5.00	4.96	5.60	5.44	5.48	5.64
4047	1.41, 3	1	0	150	0.16	63	87	1	5.52	5.70	5.22	5.14	5.20	5.18	5.24	5.14	5.22	5.44
4048	1.41, 3	1	0	150	0.33	50	100	1	5.02	5.30	4.90	4.80	4.86	5.20	5.12	4.96	5.02	5.12
4049	1.41, 3	1	0	150	0.50	37	113	1	5.10	5.72	5.18	5.06	5.08	5.32	4.88	4.76	4.78	5.06
4050	1.41, 3	1	0	200	0	100	100	1	5.18	5.36	5.30	5.22	5.28	5.06	5.42	5.36	5.40	5.54
4051	1.41, 3	1	0	200	0.16	84	116	1	5.32	5.50	5.14	5.08	5.10	5.16	5.70	5.60	5.66	5.54
4052	1.41, 3	1	0	200	0.33	67	133	1	5.04	5.44	5.42	5.28	5.34	5.68	5.66	5.58	5.60	5.66
4053	1.41, 3	1	0	200	0.50	50	150	1	5.04	5.32	4.98	4.96	4.96	5.44	5.02	4.94	4.96	5.28
4054	1.41, 3	1.5	0	20	0	10	10	1	3.96	3.60	4.62	3.78	4.08	3.60	4.74	4.14	4.46	3.56
4055	1.41, 3	1.5	+	20	0.16	8	12	1	3.98	3.82	3.60	3.02	3.28	3.52	3.70	3.12	3.50	3.52
4056	1.41, 3	1.5	-	20	0.16	12	8	1	5.30	4.66	5.98	4.90	5.52	3.48	5.80	5.00	5.40	3.58
4057	1.41, 3	1.5	+	20	0.33	7	13	1	3.90	4.68	3.32	2.64	2.98	3.78	3.42	2.90	3.18	4.02
4058	1.41, 3	1.5	-	20	0.33	13	7	1	6.02	5.72	6.46	5.56	6.08	4.16	6.62	5.66	6.08	3.94
4059	1.41, 3	1.5	+	20	0.50	5	15	1	3.36	5.80	2.90	2.62	2.74	4.72	2.74	2.22	2.48	4.60
4060	1.41, 3	1.5	-	20	0.50	15	5	1	7.52	8.50	8.16	7.20	7.48	5.84	8.84	8.02	8.42	5.76
4061	1.41, 3	1.5	0	40	0	20	20	1	4.96	4.50	4.54	4.16	4.22	4.06	4.56	4.06	4.30	3.82
4062	1.41, 3	1.5	+	40	0.16	17	23	1	4.24	4.56	4.12	3.72	3.84	4.40	3.72	3.48	3.62	3.98

	D	VR	P	N	Δn	n1	n2	ε	F g	B-F g	F t	F-GG t	F-HF t	B-F t	F g x t	F-GG g x t	F-HF g x t	B-F g x t
4063	1.41, 3	1.5	-	40	0.16	23	17	1	5.66	5.20	5.64	5.02	5.20	4.46	5.72	5.24	5.54	4.50
4064	1.41, 3	1.5	+	40	0.33	13	27	1	3.14	4.28	3.12	2.74	2.82	4.50	3.20	2.88	3.06	4.60
4065	1.41, 3	1.5	-	40	0.33	27	13	1	6.60	5.58	7.36	6.80	7.00	4.48	6.32	5.94	6.18	4.16
4066	1.41, 3	1.5	+	40	0.50	10	30	1	2.66	5.30	2.52	2.28	2.42	5.06	2.78	2.60	2.70	5.24
4067	1.41, 3	1.5	-	40	0.50	30	10	1	7.28	6.86	8.30	7.82	8.08	5.28	8.00	7.46	7.66	5.32
4068	1.41, 3	1.5	0	60	0	30	30	1	4.78	4.72	5.04	4.78	4.88	4.68	5.24	4.98	5.12	4.96
4069	1.41, 3	1.5	+	60	0.16	25	35	1	4.10	4.50	4.28	4.06	4.16	4.72	3.68	3.44	3.52	4.38
4070	1.41, 3	1.5	-	60	0.16	35	25	1	5.46	4.80	6.32	5.80	6.06	5.00	5.82	5.56	5.66	5.00
4071	1.41, 3	1.5	+	60	0.33	20	40	1	3.56	5.36	3.50	3.26	3.38	4.84	3.46	3.28	3.40	4.74
4072	1.41, 3	1.5	-	60	0.33	40	20	1	6.14	5.18	7.32	6.84	6.98	4.80	6.72	6.46	6.60	4.40
4073	1.41, 3	1.5	+	60	0.50	15	45	1	3.28	5.50	2.94	2.78	2.84	4.92	2.80	2.62	2.74	5.26
4074	1.41, 3	1.5	-	60	0.50	45	15	1	7.32	5.88	8.70	8.14	8.40	4.74	8.52	8.14	8.40	5.10
4075	1.41, 3	1.5	0	80	0	40	40	1	4.62	4.56	5.78	5.56	5.58	5.44	4.82	4.52	4.68	4.52
4076	1.41, 3	1.5	+	80	0.16	34	46	1	3.96	4.40	4.56	4.36	4.40	5.36	4.48	4.34	4.38	5.26
4077	1.41, 3	1.5	-	80	0.16	46	34	1	5.40	4.82	6.58	6.22	6.48	5.34	5.58	5.28	5.42	4.50
4078	1.41, 3	1.5	+	80	0.33	27	53	1	3.60	4.86	3.78	3.64	3.72	5.34	3.04	2.88	2.94	4.82
4079	1.41, 3	1.5	-	80	0.33	53	27	1	6.26	5.28	7.88	7.54	7.68	5.08	7.14	6.94	7.00	4.72
4080	1.41, 3	1.5	+	80	0.50	20	60	1	3.14	5.84	2.90	2.68	2.74	5.56	2.40	2.18	2.26	4.98
4081	1.41, 3	1.5	-	80	0.50	60	20	1	7.58	5.76	9.16	8.84	8.96	5.46	8.60	8.34	8.44	4.70
4082	1.41, 3	1.5	0	100	0	50	50	1	5.18	5.06	5.10	4.82	4.98	4.88	4.96	4.84	4.88	4.86
4083	1.41, 3	1.5	+	100	0.16	42	58	1	4.24	4.92	4.46	4.30	4.34	5.18	3.82	3.64	3.72	4.84
4084	1.41, 3	1.5	-	100	0.16	58	42	1	6.04	5.40	6.20	5.74	5.98	4.98	6.00	5.74	5.84	4.98
4085	1.41, 3	1.5	+	100	0.33	33	67	1	3.58	5.00	3.40	3.22	3.30	5.10	3.44	3.30	3.38	5.04
4086	1.41, 3	1.5	-	100	0.33	67	33	1	7.24	5.88	7.14	6.96	7.04	4.90	7.50	7.28	7.44	5.20
4087	1.41, 3	1.5	+	100	0.50	25	75	1	2.90	5.10	2.64	2.48	2.58	5.06	2.46	2.40	2.40	4.54
4088	1.41, 3	1.5	-	100	0.50	75	25	1	8.06	6.38	8.48	8.22	8.38	4.78	8.54	8.26	8.38	5.32
4089	1.41, 3	1.5	0	150	0	75	75	1	5.52	5.58	4.98	4.78	4.86	4.98	5.54	5.44	5.48	5.48
4090	1.41, 3	1.5	+	150	0.16	63	87	1	4.80	5.58	4.20	4.08	4.14	5.08	4.48	4.40	4.48	5.60
4091	1.41, 3	1.5	-	150	0.16	87	63	1	6.12	5.34	6.10	6.00	6.06	5.02	6.12	5.92	5.96	4.98
4092	1.41, 3	1.5	+	150	0.33	50	100	1	3.80	5.08	3.24	3.22	3.22	5.08	3.38	3.28	3.32	5.38
4093	1.41, 3	1.5	-	150	0.33	100	50	1	6.48	4.94	7.44	7.28	7.32	4.92	7.20	7.12	7.20	5.20
4094	1.41, 3	1.5	+	150	0.50	37	113	1	3.02	5.64	2.68	2.64	2.66	5.42	2.34	2.34	2.34	4.96

	D	VR	P	N	Δn	n1	n2	ε	F g	B-F g	F t	F-GG t	F-HF t	B-F t	F g x t	F-GG g x t	F-HF g x t	B-F g x t
4095	1.41, 3	1.5	-	150	0.50	113	37	1	7.42	5.84	9.22	9.00	9.12	5.00	9.12	8.86	8.94	5.76
4096	1.41, 3	1.5	0	200	0	100	100	1	5.16	5.18	5.22	5.02	5.10	5.02	5.46	5.36	5.38	5.46
4097	1.41, 3	1.5	+	200	0.16	84	116	1	4.30	5.38	4.50	4.36	4.42	5.08	4.66	4.62	4.64	5.48
4098	1.41, 3	1.5	-	200	0.16	116	84	1	5.90	5.04	6.36	6.22	6.28	5.16	6.46	6.36	6.40	5.38
4099	1.41, 3	1.5	+	200	0.33	67	133	1	3.70	5.10	3.74	3.58	3.62	5.38	3.66	3.56	3.60	5.38
4100	1.41, 3	1.5	-	200	0.33	133	67	1	6.52	5.38	7.12	6.96	7.02	4.96	7.58	7.44	7.52	5.60
4101	1.41, 3	1.5	+	200	0.50	50	150	1	3.02	5.34	2.86	2.76	2.82	5.46	2.80	2.70	2.74	5.34
4102	1.41, 3	1.5	-	200	0.50	150	50	1	7.76	5.66	8.84	8.68	8.76	5.34	8.82	8.68	8.76	4.96
4103	1.41, 3	2	0	20	0	10	10	1	4.32	3.74	4.52	3.70	4.20	3.42	4.94	4.08	4.52	3.52
4104	1.41, 3	2	+	20	0.16	8	12	1	3.76	4.06	3.06	2.64	2.78	3.36	3.16	2.74	2.92	3.38
4105	1.41, 3	2	-	20	0.16	12	8	1	6.46	5.32	7.08	5.96	6.32	3.76	6.86	5.88	6.32	3.78
4106	1.41, 3	2	+	20	0.33	7	13	1	3.40	4.70	2.50	2.06	2.26	3.72	2.46	2.02	2.26	3.84
4107	1.41, 3	2	-	20	0.33	13	7	1	7.46	6.18	8.14	7.08	7.48	4.20	8.14	6.90	7.50	4.06
4108	1.41, 3	2	+	20	0.50	5	15	1	2.66	5.44	1.94	1.60	1.82	4.42	1.66	1.38	1.46	4.44
4109	1.41, 3	2	-	20	0.50	15	5	1	9.92	9.32	11.32	9.88	10.46	6.20	11.82	10.36	10.96	5.98
4110	1.41, 3	2	0	40	0	20	20	1	5.12	4.66	4.72	4.14	4.28	4.02	4.60	4.16	4.34	3.86
4111	1.41, 3	2	+	40	0.16	17	23	1	4.22	4.58	3.64	3.32	3.34	4.24	3.40	3.10	3.26	3.92
4112	1.41, 3	2	-	40	0.16	23	17	1	6.20	5.30	6.54	5.76	6.14	4.24	6.30	5.84	6.08	4.58
4113	1.41, 3	2	+	40	0.33	13	27	1	2.62	4.22	2.46	2.16	2.28	4.46	2.38	2.02	2.18	4.56
4114	1.41, 3	2	-	40	0.33	27	13	1	8.12	5.94	9.62	8.88	9.22	4.50	8.20	7.66	7.92	4.14
4115	1.41, 3	2	+	40	0.50	10	30	1	1.92	4.78	1.66	1.46	1.56	4.98	1.82	1.58	1.70	5.12
4116	1.41, 3	2	-	40	0.50	30	10	1	9.42	7.08	11.08	10.40	10.70	5.34	11.12	10.18	10.70	5.34
4117	1.41, 3	2	0	60	0	30	30	1	5.02	4.62	5.28	4.94	5.10	4.96	5.20	4.90	5.04	4.70
4118	1.41, 3	2	+	60	0.16	25	35	1	3.80	4.46	3.72	3.46	3.52	4.58	3.20	2.98	3.14	4.42
4119	1.41, 3	2	-	60	0.16	35	25	1	6.10	4.84	7.06	6.62	6.80	5.02	6.92	6.62	6.76	5.08
4120	1.41, 3	2	+	60	0.33	20	40	1	2.76	5.16	2.66	2.56	2.60	4.82	2.46	2.36	2.40	4.58
4121	1.41, 3	2	-	60	0.33	40	20	1	7.48	5.26	8.98	8.72	8.80	4.72	8.44	8.12	8.24	4.16
4122	1.41, 3	2	+	60	0.50	15	45	1	2.28	5.26	1.72	1.58	1.70	4.94	1.84	1.76	1.78	5.18
4123	1.41, 3	2	-	60	0.50	45	15	1	9.66	6.30	11.74	11.16	11.44	5.04	11.38	10.92	11.06	5.18
4124	1.41, 3	2	0	80	0	40	40	1	4.86	4.44	5.62	5.34	5.46	5.16	4.78	4.60	4.68	4.54
4125	1.41, 3	2	+	80	0.16	34	46	1	3.64	4.34	4.22	4.02	4.08	5.58	3.78	3.66	3.72	5.16
4126	1.41, 3	2	-	80	0.16	46	34	1	6.04	4.94	7.24	6.86	7.04	5.24	6.26	5.98	6.10	4.70

	D	VR	P	N	Δn	n1	n2	ε	F g	B-F g	F t	F-GG t	F-HF t	B-F t	F g x t	F-GG g x t	F-HF g x t	B-F g x t
4127	1.41, 3	2	+	80	0.33	27	53	1	3.02	4.78	2.82	2.60	2.72	5.22	2.04	1.96	1.98	4.72
4128	1.41, 3	2	-	80	0.33	53	27	1	7.28	5.40	9.62	9.24	9.42	5.18	9.26	8.86	8.98	4.80
4129	1.41, 3	2	+	80	0.50	20	60	1	2.32	5.60	1.84	1.78	1.80	5.50	1.32	1.24	1.30	5.02
4130	1.41, 3	2	-	80	0.50	60	20	1	9.64	5.92	12.76	12.24	12.44	5.44	12.12	11.80	11.94	4.90
4131	1.41, 3	2	0	100	0	50	50	1	5.00	4.98	4.90	4.72	4.80	4.70	4.96	4.76	4.84	4.94
4132	1.41, 3	2	+	100	0.16	42	58	1	4.06	4.94	3.94	3.70	3.80	5.06	3.30	3.20	3.26	4.60
4133	1.41, 3	2	-	100	0.16	58	42	1	6.60	5.44	7.02	6.78	6.86	4.86	6.82	6.60	6.66	4.88
4134	1.41, 3	2	+	100	0.33	33	67	1	2.60	4.86	2.52	2.46	2.50	5.30	2.34	2.18	2.26	5.22
4135	1.41, 3	2	-	100	0.33	67	33	1	8.40	5.98	9.30	8.96	9.10	4.86	9.18	8.94	9.08	5.06
4136	1.41, 3	2	+	100	0.50	25	75	1	2.10	4.80	1.64	1.56	1.58	5.00	1.54	1.44	1.52	4.30
4137	1.41, 3	2	-	100	0.50	75	25	1	10.36	6.50	11.44	11.12	11.26	4.86	11.94	11.72	11.78	5.06
4138	1.41, 3	2	0	150	0	75	75	1	5.54	5.40	5.04	4.94	5.02	5.16	5.38	5.20	5.26	5.30
4139	1.41, 3	2	+	150	0.16	63	87	1	4.34	5.46	3.68	3.48	3.56	5.08	3.88	3.68	3.84	5.46
4140	1.41, 3	2	-	150	0.16	87	63	1	6.56	5.36	6.94	6.70	6.84	4.80	6.70	6.58	6.58	4.90
4141	1.41, 3	2	+	150	0.33	50	100	1	3.02	5.02	2.24	2.14	2.20	5.14	2.44	2.42	2.44	5.32
4142	1.41, 3	2	-	150	0.33	100	50	1	7.92	5.14	9.18	9.04	9.08	4.90	9.10	8.84	8.94	5.24
4143	1.41, 3	2	+	150	0.50	37	113	1	2.02	5.58	1.68	1.62	1.66	5.36	1.54	1.54	1.54	4.80
4144	1.41, 3	2	-	150	0.50	113	37	1	9.72	6.00	12.22	12.04	12.10	5.12	11.90	11.68	11.80	5.42
4145	1.41, 3	2	0	200	0	100	100	1	5.10	5.10	5.32	5.16	5.22	5.02	5.60	5.48	5.48	5.64
4146	1.41, 3	2	+	200	0.16	84	116	1	3.78	5.32	3.82	3.72	3.80	5.26	4.12	4.04	4.04	5.38
4147	1.41, 3	2	-	200	0.16	116	84	1	6.64	5.14	6.98	6.82	6.92	5.30	7.02	6.92	6.98	5.30
4148	1.41, 3	2	+	200	0.33	67	133	1	3.08	5.00	2.60	2.48	2.50	5.28	2.66	2.62	2.64	5.20
4149	1.41, 3	2	-	200	0.33	133	67	1	7.72	5.20	9.30	9.16	9.20	4.80	9.46	9.36	9.40	5.72
4150	1.41, 3	2	+	200	0.50	50	150	1	2.16	5.10	1.78	1.68	1.72	5.38	1.46	1.42	1.44	5.32
4151	1.41, 3	2	-	200	0.50	150	50	1	10.14	5.70	12.08	11.90	12.00	5.28	12.20	11.94	12.12	4.94
4152	1.41, 3	5	0	20	0	10	10	1	5.92	5.12	5.20	4.36	4.70	3.36	5.04	4.22	4.62	3.32
4153	1.41, 3	5	+	20	0.16	8	12	1	3.82	4.68	2.00	1.68	1.82	3.26	2.06	1.60	1.82	3.38
4154	1.41, 3	5	-	20	0.16	12	8	1	9.90	6.40	9.96	8.36	8.90	3.54	10.06	8.30	8.90	3.60
4155	1.41, 3	5	+	20	0.33	7	13	1	3.00	4.76	1.32	1.12	1.20	3.28	1.30	.94	1.06	3.36
4156	1.41, 3	5	-	20	0.33	13	7	1	12.48	7.30	13.58	11.58	12.34	3.92	13.62	12.06	12.44	3.64
4157	1.41, 3	5	+	20	0.50	5	15	1	1.50	4.60	.38	.26	.32	3.56	.26	.18	.22	3.78
4158	1.41, 3	5	-	20	0.50	15	5	1	18.54	10.92	22.26	19.72	20.48	5.70	22.42	20.18	20.84	5.68

	D	VR	P	N	Δn	n1	n2	ε	F g	B-F g	F t	F-GG t	F-HF t	B-F t	F g x t	F-GG g x t	F-HF g x t	B-F g x t
4159	1.41, 3	5	0	40	0	20	20	1	5.46	4.82	4.72	4.32	4.42	3.74	4.90	4.36	4.56	3.72
4160	1.41, 3	5	+	40	0.16	17	23	1	3.54	4.72	2.74	2.44	2.60	3.84	2.66	2.38	2.48	4.00
4161	1.41, 3	5	-	40	0.16	23	17	1	8.06	5.48	8.40	7.56	7.90	4.20	8.60	7.74	8.10	4.40
4162	1.41, 3	5	+	40	0.33	13	27	1	1.72	4.20	.94	.82	.88	4.06	1.06	.88	.98	4.32
4163	1.41, 3	5	-	40	0.33	27	13	1	12.32	5.88	15.42	14.08	14.62	4.30	14.62	13.52	13.80	3.98
4164	1.41, 3	5	+	40	0.50	10	30	1	.88	3.94	.32	.28	.30	4.60	.38	.24	.30	4.54
4165	1.41, 3	5	-	40	0.50	30	10	1	16.92	7.26	21.60	20.24	20.60	4.80	21.82	20.34	20.78	4.60
4166	1.41, 3	5	0	60	0	30	30	1	5.10	4.62	5.48	5.20	5.32	4.80	5.22	4.78	4.98	4.56
4167	1.41, 3	5	+	60	0.16	25	35	1	3.00	4.64	2.56	2.44	2.46	4.78	2.24	2.06	2.20	4.42
4168	1.41, 3	5	-	60	0.16	35	25	1	7.98	4.54	9.92	9.08	9.32	4.46	9.50	9.08	9.24	4.48
4169	1.41, 3	5	+	60	0.33	20	40	1	1.68	4.66	1.22	1.08	1.14	4.68	.90	.82	.88	4.68
4170	1.41, 3	5	-	60	0.33	40	20	1	11.72	5.36	14.94	13.94	14.36	4.12	15.00	14.16	14.48	3.68
4171	1.41, 3	5	+	60	0.50	15	45	1	1.04	4.86	.34	.30	.32	5.00	.20	.20	.20	4.94
4172	1.41, 3	5	-	60	0.50	45	15	1	16.78	6.80	23.08	22.20	22.50	4.80	22.62	21.66	21.96	4.84
4173	1.41, 3	5	0	80	0	40	40	1	5.10	4.80	5.54	5.14	5.28	4.96	5.08	4.84	4.96	4.36
4174	1.41, 3	5	+	80	0.16	34	46	1	2.98	4.14	3.06	2.92	3.00	5.72	2.74	2.56	2.64	5.42
4175	1.41, 3	5	-	80	0.16	46	34	1	7.94	5.16	9.22	8.72	8.88	5.00	8.28	7.92	8.06	4.62
4176	1.41, 3	5	+	80	0.33	27	53	1	1.82	4.76	1.24	1.12	1.18	5.72	.88	.84	.84	4.62
4177	1.41, 3	5	-	80	0.33	53	27	1	11.24	5.68	15.66	14.84	15.10	4.88	15.04	14.36	14.60	4.50
4178	1.41, 3	5	+	80	0.50	20	60	1	.88	4.88	.34	.34	.34	5.56	.18	.16	.18	4.72
4179	1.41, 3	5	-	80	0.50	60	20	1	16.40	6.24	23.58	22.80	22.98	4.90	23.38	22.42	22.80	5.06
4180	1.41, 3	5	0	100	0	50	50	1	4.98	4.84	5.06	4.86	4.92	4.62	5.00	4.72	4.76	4.84
4181	1.41, 3	5	+	100	0.16	42	58	1	3.00	4.90	2.40	2.30	2.32	4.68	2.38	2.28	2.30	4.44
4182	1.41, 3	5	-	100	0.16	58	42	1	8.44	5.34	9.20	8.86	8.98	5.08	9.36	8.82	8.94	4.78
4183	1.41, 3	5	+	100	0.33	33	67	1	1.50	4.68	.88	.88	.88	4.94	.96	.92	.94	4.84
4184	1.41, 3	5	-	100	0.33	67	33	1	12.78	6.08	15.82	15.34	15.54	5.02	15.54	15.00	15.20	4.98
4185	1.41, 3	5	+	100	0.50	25	75	1	.72	4.50	.36	.32	.34	4.88	.26	.18	.22	4.36
4186	1.41, 3	5	-	100	0.50	75	25	1	17.82	6.56	22.86	22.12	22.40	4.44	22.02	21.46	21.68	4.76
4187	1.41, 3	5	0	150	0	75	75	1	5.62	5.50	5.14	4.96	5.04	5.02	5.00	4.86	4.92	5.04
4188	1.41, 3	5	+	150	0.16	63	87	1	3.54	5.34	2.56	2.42	2.46	5.04	2.74	2.64	2.68	5.12
4189	1.41, 3	5	-	150	0.16	87	63	1	7.92	5.28	8.92	8.74	8.78	4.90	8.62	8.44	8.48	4.86
4190	1.41, 3	5	+	150	0.33	50	100	1	1.72	4.96	1.00	.96	.98	4.90	1.02	.98	1.00	5.20

	D	VR	P	N	Δn	n1	n2	ε	F g	B-F g	F t	F-GG t	F-HF t	B-F t	F g x t	F-GG g x t	F-HF g x t	B-F g x t
4191	1.41, 3	5	-	150	0.33	100	50	1	11.82	5.00	15.18	14.80	14.90	5.02	15.50	15.10	15.24	4.74
4192	1.41, 3	5	+	150	0.50	37	113	1	.54	4.76	.36	.34	.34	5.36	.28	.28	.28	4.84
4193	1.41, 3	5	-	150	0.50	113	37	1	17.38	5.86	23.00	22.54	22.70	4.96	22.68	22.32	22.38	5.32
4194	1.41, 3	5	0	200	0	100	100	1	5.14	4.94	5.38	5.16	5.20	5.06	5.26	5.20	5.24	5.38
4195	1.41, 3	5	+	200	0.16	84	116	1	2.92	5.16	2.56	2.48	2.48	5.10	2.66	2.58	2.62	5.32
4196	1.41, 3	5	-	200	0.16	116	84	1	8.24	5.06	9.40	9.24	9.32	5.02	9.42	9.28	9.30	5.14
4197	1.41, 3	5	+	200	0.33	67	133	1	1.86	4.76	.98	.94	.94	4.98	.92	.92	.92	5.36
4198	1.41, 3	5	-	200	0.33	133	67	1	12.10	5.34	15.22	14.86	15.02	4.68	15.62	15.30	15.36	5.78
4199	1.41, 3	5	+	200	0.50	50	150	1	.58	4.80	.28	.28	.28	5.26	.18	.18	.18	5.18
4200	1.41, 3	5	-	200	0.50	150	50	1	17.36	5.40	22.72	22.42	22.52	5.28	23.32	23.00	23.10	4.94
4201	2.31, 8	1	0	20	0	10	10	.50	3.70	2.60	12.42	8.90	8.90	6.66	7.90	3.94	3.98	2.62
4202	2.31, 8	1	0	20	0.16	8	12	.50	4.32	3.10	12.38	8.66	8.66	6.72	7.88	4.44	4.48	3.22
4203	2.31, 8	1	0	20	0.33	7	13	.50	4.10	3.12	12.48	8.96	8.98	6.86	7.58	4.18	4.18	3.02
4204	2.31, 8	1	0	20	0.50	5	15	.50	4.62	5.32	12.24	8.20	8.22	8.82	7.64	4.88	4.88	5.36
4205	2.31, 8	1	0	40	0	20	20	.50	4.24	3.20	11.18	7.70	7.74	5.86	8.12	4.34	4.34	3.16
4206	2.31, 8	1	0	40	0.16	17	23	.50	4.26	3.22	11.30	7.68	7.68	6.08	8.10	4.28	4.28	3.30
4207	2.31, 8	1	0	40	0.33	13	27	.50	4.46	4.34	11.28	7.22	7.22	6.44	8.22	4.36	4.36	4.06
4208	2.31, 8	1	0	40	0.50	10	30	.50	4.24	5.32	10.88	7.12	7.12	7.94	7.70	4.40	4.40	5.56
4209	2.31, 8	1	0	60	0	30	30	.50	4.48	3.78	10.72	7.04	7.04	5.72	7.94	4.52	4.52	3.96
4210	2.31, 8	1	0	60	0.16	25	35	.50	4.84	4.24	10.64	7.30	7.30	6.14	8.34	4.82	4.82	4.20
4211	2.31, 8	1	0	60	0.33	20	40	.50	4.62	5.12	10.76	7.52	7.52	6.76	8.32	4.52	4.52	5.24
4212	2.31, 8	1	0	60	0.50	15	45	.50	4.72	5.80	10.86	6.96	6.96	7.22	8.46	4.54	4.54	6.40
4213	2.31, 8	1	0	80	0	40	40	.50	5.02	4.52	9.92	6.52	6.52	5.48	8.86	5.24	5.24	4.64
4214	2.31, 8	1	0	80	0.16	34	46	.50	4.74	4.10	10.02	6.54	6.54	5.66	8.32	4.66	4.66	4.38
4215	2.31, 8	1	0	80	0.33	27	53	.50	4.86	5.12	10.36	6.64	6.66	6.30	8.00	4.96	4.96	4.94
4216	2.31, 8	1	0	80	0.50	20	60	.50	4.52	5.84	10.20	6.62	6.62	7.06	7.98	4.38	4.38	5.90
4217	2.31, 8	1	0	100	0	50	50	.50	5.20	4.54	9.40	6.28	6.28	5.46	8.34	4.76	4.76	4.16
4218	2.31, 8	1	0	100	0.16	42	58	.50	4.72	4.46	9.82	6.20	6.20	5.66	8.48	4.74	4.74	4.58
4219	2.31, 8	1	0	100	0.33	33	67	.50	4.78	4.54	10.08	6.28	6.28	5.90	7.74	4.88	4.88	4.66
4220	2.31, 8	1	0	100	0.50	25	75	.50	4.52	5.76	10.00	6.32	6.32	7.10	7.60	4.34	4.34	5.42
4221	2.31, 8	1	0	150	0	75	75	.50	5.14	5.02	8.70	5.46	5.46	4.80	8.66	5.04	5.04	4.90
4222	2.31, 8	1	0	150	0.16	63	87	.50	5.56	5.38	8.86	5.46	5.46	5.04	8.98	5.50	5.50	5.36

	D	VR	P	N	Δn	n1	n2	ε	F g	B-F g	F t	F-GG t	F-HF t	B-F t	F g x t	F-GG g x t	F-HF g x t	B-F g x t
4223	2.31, 8	1	0	150	0.33	50	100	.50	5.00	5.14	9.06	5.74	5.74	5.60	8.56	4.98	4.98	5.48
4224	2.31, 8	1	0	150	0.50	37	113	.50	4.60	5.50	9.36	5.76	5.76	6.14	8.44	4.96	4.96	5.80
4225	2.31, 8	1	0	200	0	100	100	.50	5.04	4.86	8.68	5.28	5.28	4.76	8.18	4.96	4.96	4.84
4226	2.31, 8	1	0	200	0.16	84	116	.50	5.10	5.26	9.18	5.26	5.26	4.96	8.34	4.92	4.92	5.06
4227	2.31, 8	1	0	200	0.33	67	133	.50	5.46	5.60	8.86	5.48	5.48	5.20	9.28	5.42	5.42	5.90
4228	2.31, 8	1	0	200	0.50	50	150	.50	4.84	5.98	9.44	5.70	5.70	6.06	8.22	4.94	4.94	5.82
4229	2.31, 8	1.5	0	20	0	10	10	.50	4.40	2.88	12.60	9.04	9.08	6.86	8.22	4.44	4.44	3.16
4230	2.31, 8	1.5	+	20	0.16	8	12	.50	4.06	2.86	11.48	7.96	7.96	6.38	7.54	4.02	4.02	2.76
4231	2.31, 8	1.5	-	20	0.16	12	8	.50	5.10	3.62	13.54	9.80	9.86	7.36	9.42	5.10	5.18	3.90
4232	2.31, 8	1.5	+	20	0.33	7	13	.50	3.64	2.64	10.98	7.70	7.70	6.52	6.38	3.68	3.68	2.46
4233	2.31, 8	1.5	-	20	0.33	13	7	.50	5.28	4.50	14.44	10.24	10.26	7.96	9.94	5.40	5.40	4.36
4234	2.31, 8	1.5	+	20	0.50	5	15	.50	3.88	4.20	9.74	6.58	6.58	8.04	6.46	4.04	4.04	4.06
4235	2.31, 8	1.5	-	20	0.50	15	5	.50	6.44	7.56	15.28	10.50	10.54	9.92	11.26	6.64	6.66	7.76
4236	2.31, 8	1.5	0	40	0	20	20	.50	4.46	3.30	11.36	7.48	7.50	5.84	8.66	4.42	4.44	3.52
4237	2.31, 8	1.5	+	40	0.16	17	23	.50	4.20	3.14	10.58	7.08	7.08	6.06	7.44	4.04	4.04	3.24
4238	2.31, 8	1.5	-	40	0.16	23	17	.50	5.64	4.58	11.86	8.08	8.10	6.20	10.06	5.58	5.58	4.60
4239	2.31, 8	1.5	+	40	0.33	13	27	.50	3.60	3.66	9.32	6.06	6.08	6.22	6.62	3.74	3.74	3.34
4240	2.31, 8	1.5	-	40	0.33	27	13	.50	5.68	5.68	13.26	8.70	8.72	6.96	10.20	5.70	5.70	5.94
4241	2.31, 8	1.5	+	40	0.50	10	30	.50	3.10	4.20	8.04	4.94	4.94	7.20	5.72	3.16	3.16	4.08
4242	2.31, 8	1.5	-	40	0.50	30	10	.50	6.78	7.18	14.32	9.72	9.74	8.82	11.14	6.54	6.56	7.64
4243	2.31, 8	1.5	0	60	0	30	30	.50	4.56	3.98	10.54	6.96	6.96	5.62	8.20	4.62	4.62	3.90
4244	2.31, 8	1.5	+	60	0.16	25	35	.50	4.00	3.90	9.58	6.48	6.48	5.60	7.62	4.22	4.22	4.16
4245	2.31, 8	1.5	-	60	0.16	35	25	.50	5.94	5.02	11.38	8.06	8.06	6.08	9.82	6.04	6.04	5.08
4246	2.31, 8	1.5	+	60	0.33	20	40	.50	3.56	4.50	9.04	5.66	5.66	6.44	6.54	3.74	3.74	4.52
4247	2.31, 8	1.5	-	60	0.33	40	20	.50	6.74	6.14	12.34	8.54	8.56	6.48	11.14	6.78	6.78	6.00
4248	2.31, 8	1.5	+	60	0.50	15	45	.50	3.14	5.00	7.98	4.58	4.58	7.06	5.50	3.16	3.16	5.02
4249	2.31, 8	1.5	-	60	0.50	45	15	.50	7.60	7.50	13.68	9.32	9.32	7.62	11.92	7.50	7.50	7.54
4250	2.31, 8	1.5	0	80	0	40	40	.50	5.16	4.60	9.84	6.26	6.26	5.14	8.88	5.34	5.34	4.78
4251	2.31, 8	1.5	+	80	0.16	34	46	.50	4.46	4.42	8.90	5.82	5.82	5.48	7.48	4.46	4.46	4.36
4252	2.31, 8	1.5	-	80	0.16	46	34	.50	5.56	4.94	10.86	7.14	7.14	5.60	9.82	5.50	5.50	4.90
4253	2.31, 8	1.5	+	80	0.33	27	53	.50	3.66	4.54	8.72	5.02	5.02	5.94	6.56	3.66	3.66	4.32
4254	2.31, 8	1.5	-	80	0.33	53	27	.50	6.56	5.30	11.92	8.26	8.26	6.22	10.80	6.68	6.68	5.48

	D	VR	P	N	Δn	n1	n2	ε	F g	B-F g	F t	F-GG t	F-HF t	B-F t	F g x t	F-GG g x t	F-HF g x t	B-F g x t
4255	2.31, 8	1.5	+	80	0.50	20	60	.50	2.94	5.06	7.24	4.62	4.62	6.76	5.46	3.06	3.06	4.98
4256	2.31, 8	1.5	-	80	0.50	60	20	.50	7.52	7.40	13.12	8.90	8.90	7.56	12.40	7.82	7.82	7.24
4257	2.31, 8	1.5	0	100	0	50	50	.50	5.18	4.68	9.60	6.48	6.48	5.58	8.46	4.68	4.68	4.58
4258	2.31, 8	1.5	+	100	0.16	42	58	.50	4.12	4.28	8.78	5.64	5.64	5.54	7.30	4.26	4.28	4.16
4259	2.31, 8	1.5	-	100	0.16	58	42	.50	5.54	5.14	10.34	7.24	7.24	5.98	9.82	5.62	5.64	5.02
4260	2.31, 8	1.5	+	100	0.33	33	67	.50	3.64	4.24	7.76	4.86	4.86	5.82	6.12	3.72	3.72	4.52
4261	2.31, 8	1.5	-	100	0.33	67	33	.50	7.28	6.06	11.68	7.76	7.76	5.82	11.38	7.12	7.12	5.94
4262	2.31, 8	1.5	+	100	0.50	25	75	.50	2.94	4.88	7.14	4.28	4.28	6.48	5.10	2.84	2.84	4.70
4263	2.31, 8	1.5	-	100	0.50	75	25	.50	7.84	7.22	12.66	9.24	9.24	7.50	11.74	7.40	7.40	7.08
4264	2.31, 8	1.5	0	150	0	75	75	.50	5.32	4.98	8.50	5.44	5.44	5.04	8.72	5.26	5.26	4.98
4265	2.31, 8	1.5	+	150	0.16	63	87	.50	4.54	5.10	7.68	4.76	4.76	5.04	7.98	4.80	4.80	5.18
4266	2.31, 8	1.5	-	150	0.16	87	63	.50	6.14	5.28	9.52	5.84	5.84	4.72	9.92	6.06	6.06	5.30
4267	2.31, 8	1.5	+	150	0.33	50	100	.50	3.76	4.78	7.08	4.16	4.16	5.22	6.52	3.82	3.82	4.86
4268	2.31, 8	1.5	-	150	0.33	100	50	.50	6.88	5.44	10.58	6.64	6.64	5.18	10.58	6.70	6.70	5.74
4269	2.31, 8	1.5	+	150	0.50	37	113	.50	3.12	5.32	6.38	3.66	3.66	5.84	5.70	3.02	3.02	5.58
4270	2.31, 8	1.5	-	150	0.50	113	37	.50	7.52	6.24	11.98	7.72	7.72	5.80	11.48	7.38	7.38	5.98
4271	2.31, 8	1.5	0	200	0	100	100	.50	5.28	5.26	8.54	5.42	5.42	4.82	8.46	5.16	5.16	4.92
4272	2.31, 8	1.5	+	200	0.16	84	116	.50	4.38	4.88	7.94	4.48	4.48	4.74	7.56	4.32	4.32	5.04
4273	2.31, 8	1.5	-	200	0.16	116	84	.50	5.66	5.08	9.74	6.10	6.10	4.78	9.36	5.66	5.66	5.16
4274	2.31, 8	1.5	+	200	0.33	67	133	.50	4.02	5.58	6.90	3.96	3.96	5.14	7.02	3.88	3.88	5.56
4275	2.31, 8	1.5	-	200	0.33	133	67	.50	6.60	5.76	11.12	7.00	7.02	5.42	10.64	6.46	6.46	5.48
4276	2.31, 8	1.5	+	200	0.50	50	150	.50	3.20	5.18	6.22	3.52	3.54	5.72	5.54	3.12	3.12	5.42
4277	2.31, 8	1.5	-	200	0.50	150	50	.50	7.60	6.26	12.18	7.86	7.86	6.20	12.14	7.46	7.46	6.18
4278	2.31, 8	2	0	20	0	10	10	.50	5.04	3.64	12.64	9.32	9.34	6.86	9.10	5.14	5.14	3.70
4279	2.31, 8	2	+	20	0.16	8	12	.50	4.36	3.00	10.84	7.70	7.70	6.46	7.74	4.28	4.32	2.88
4280	2.31, 8	2	-	20	0.16	12	8	.50	6.42	4.82	14.50	10.72	10.76	7.70	11.00	6.44	6.48	5.04
4281	2.31, 8	2	+	20	0.33	7	13	.50	3.74	3.06	10.10	7.22	7.22	7.00	6.04	3.76	3.76	2.86
4282	2.31, 8	2	-	20	0.33	13	7	.50	7.02	5.90	15.96	11.28	11.28	8.62	12.42	7.12	7.16	5.90
4283	2.31, 8	2	+	20	0.50	5	15	.50	3.52	3.46	8.22	5.74	5.76	7.46	5.72	3.80	3.80	3.46
4284	2.31, 8	2	-	20	0.50	15	5	.50	8.74	9.24	18.04	12.92	12.92	10.82	14.44	8.96	9.00	9.26
4285	2.31, 8	2	0	40	0	20	20	.50	5.14	4.18	11.34	7.58	7.58	5.72	9.14	5.22	5.22	3.96
4286	2.31, 8	2	+	40	0.16	17	23	.50	4.14	3.48	10.08	6.46	6.48	5.80	7.60	4.06	4.06	3.54

	D	VR	P	N	Δn	n1	n2	ε	F g	B-F g	F t	F-GG t	F-HF t	B-F t	F g x t	F-GG g x t	F-HF g x t	B-F g x t
4287	2.31, 8	2	-	40	0.16	23	17	.50	6.56	5.20	12.76	8.62	8.62	6.48	10.84	6.72	6.72	5.42
4288	2.31, 8	2	+	40	0.33	13	27	.50	3.40	3.28	8.18	5.10	5.10	5.96	6.08	3.56	3.56	3.22
4289	2.31, 8	2	-	40	0.33	27	13	.50	7.36	6.50	14.92	10.22	10.22	7.40	12.28	7.60	7.62	6.96
4290	2.31, 8	2	+	40	0.50	10	30	.50	2.66	4.02	6.42	3.84	3.84	7.06	4.62	2.74	2.74	3.52
4291	2.31, 8	2	-	40	0.50	30	10	.50	9.04	8.46	16.74	12.10	12.10	9.20	14.20	9.00	9.02	8.50
4292	2.31, 8	2	0	60	0	30	30	.50	4.98	4.30	10.46	6.96	6.96	5.58	8.46	4.80	4.80	4.32
4293	2.31, 8	2	+	60	0.16	25	35	.50	3.90	3.92	9.02	5.84	5.84	5.80	6.96	3.84	3.84	4.20
4294	2.31, 8	2	-	60	0.16	35	25	.50	6.90	5.80	12.28	8.40	8.40	6.32	10.90	6.70	6.70	5.64
4295	2.31, 8	2	+	60	0.33	20	40	.50	3.30	4.26	7.80	4.84	4.84	5.82	5.50	3.54	3.54	4.28
4296	2.31, 8	2	-	60	0.33	40	20	.50	8.52	6.90	13.76	9.96	9.96	6.68	13.00	8.44	8.44	6.88
4297	2.31, 8	2	+	60	0.50	15	45	.50	2.64	4.42	6.24	3.58	3.58	6.64	4.54	2.68	2.68	4.48
4298	2.31, 8	2	-	60	0.50	45	15	.50	9.98	8.40	16.12	11.50	11.50	7.96	14.98	9.96	10.00	8.34
4299	2.31, 8	2	0	80	0	40	40	.50	5.32	4.84	9.92	6.52	6.52	5.28	8.74	5.56	5.56	4.96
4300	2.31, 8	2	+	80	0.16	34	46	.50	4.32	4.68	8.52	5.48	5.48	5.58	7.04	4.32	4.34	4.68
4301	2.31, 8	2	-	80	0.16	46	34	.50	6.62	5.68	11.64	7.62	7.62	5.64	10.66	6.22	6.22	5.30
4302	2.31, 8	2	+	80	0.33	27	53	.50	3.08	4.54	7.14	4.32	4.32	5.64	5.80	3.14	3.16	4.36
4303	2.31, 8	2	-	80	0.33	53	27	.50	8.00	5.76	13.64	9.52	9.52	6.54	12.52	8.12	8.12	5.82
4304	2.31, 8	2	+	80	0.50	20	60	.50	2.40	4.56	5.86	3.30	3.30	6.48	4.30	2.34	2.34	4.52
4305	2.31, 8	2	-	80	0.50	60	20	.50	10.16	7.92	15.58	10.92	10.92	7.84	15.30	10.24	10.24	7.86
4306	2.31, 8	2	0	100	0	50	50	.50	5.24	4.80	9.66	6.48	6.48	5.64	8.38	4.86	4.86	4.62
4307	2.31, 8	2	+	100	0.16	42	58	.50	3.74	4.28	8.34	5.22	5.22	5.48	6.72	3.96	3.96	4.22
4308	2.31, 8	2	-	100	0.16	58	42	.50	6.30	5.40	10.86	7.84	7.84	6.10	10.76	6.40	6.40	5.24
4309	2.31, 8	2	+	100	0.33	33	67	.50	3.08	4.34	6.72	4.14	4.14	5.50	5.16	3.18	3.18	4.32
4310	2.31, 8	2	-	100	0.33	67	33	.50	8.86	6.56	13.50	9.14	9.14	6.20	13.04	8.78	8.78	6.36
4311	2.31, 8	2	+	100	0.50	25	75	.50	2.18	4.58	5.58	3.12	3.12	5.98	3.96	2.06	2.06	4.48
4312	2.31, 8	2	-	100	0.50	75	25	.50	10.10	7.44	15.20	11.24	11.24	7.50	14.84	9.72	9.72	7.60
4313	2.31, 8	2	0	150	0	75	75	.50	5.58	5.12	8.76	5.38	5.38	4.70	9.02	5.56	5.56	5.18
4314	2.31, 8	2	+	150	0.16	63	87	.50	4.24	4.98	7.08	4.30	4.30	4.84	7.46	4.24	4.24	4.98
4315	2.31, 8	2	-	150	0.16	87	63	.50	6.70	5.40	10.26	6.36	6.36	4.86	10.82	6.72	6.72	5.26
4316	2.31, 8	2	+	150	0.33	50	100	.50	3.18	4.70	5.88	3.38	3.38	4.98	5.62	3.00	3.00	4.68
4317	2.31, 8	2	-	150	0.33	100	50	.50	8.22	5.60	11.90	7.90	7.90	5.24	12.30	8.38	8.38	5.76
4318	2.31, 8	2	+	150	0.50	37	113	.50	2.14	4.96	4.82	2.52	2.54	5.82	4.22	2.30	2.30	5.12

	D	VR	P	N	Δn	n1	n2	ε	F g	B-F g	F t	F-GG t	F-HF t	B-F t	F g x t	F-GG g x t	F-HF g x t	B-F g x t
4319	2.31, 8	2	-	150	0.50	113	37	.50	9.92	6.46	14.52	10.14	10.14	5.90	14.30	9.56	9.56	6.48
4320	2.31, 8	2	0	200	0	100	100	.50	5.40	5.18	8.54	5.28	5.28	4.72	8.82	5.32	5.32	5.18
4321	2.31, 8	2	+	200	0.16	84	116	.50	4.18	4.84	7.16	4.32	4.32	4.94	6.74	4.00	4.00	4.88
4322	2.31, 8	2	-	200	0.16	116	84	.50	6.32	5.18	10.38	6.68	6.68	5.04	10.12	6.36	6.36	5.26
4323	2.31, 8	2	+	200	0.33	67	133	.50	3.36	5.16	5.58	3.32	3.32	4.92	5.92	3.24	3.24	5.28
4324	2.31, 8	2	-	200	0.33	133	67	.50	8.18	5.90	12.76	8.54	8.54	5.58	12.62	7.94	7.94	5.70
4325	2.31, 8	2	+	200	0.50	50	150	.50	2.30	4.94	4.64	2.38	2.38	5.62	4.30	2.30	2.30	5.08
4326	2.31, 8	2	-	200	0.50	150	50	.50	10.30	6.56	14.60	10.28	10.28	6.22	15.12	10.08	10.08	6.46
4327	2.31, 8	5	0	20	0	10	10	.50	7.32	6.36	13.92	10.40	10.42	7.24	11.80	7.86	7.90	6.70
4328	2.31, 8	5	+	20	0.16	8	12	.50	5.40	4.98	10.72	7.56	7.58	6.52	8.26	5.54	5.58	5.16
4329	2.31, 8	5	-	20	0.16	12	8	.50	11.80	8.86	18.42	13.74	13.74	8.74	17.08	12.26	12.26	9.32
4330	2.31, 8	5	+	20	0.33	7	13	.50	4.46	4.30	8.70	6.02	6.04	6.82	6.96	4.60	4.64	4.78
4331	2.31, 8	5	-	20	0.33	13	7	.50	14.38	10.64	21.50	16.12	16.12	10.26	19.90	14.20	14.22	10.84
4332	2.31, 8	5	+	20	0.50	5	15	.50	3.06	3.74	5.58	3.60	3.60	6.80	5.06	3.10	3.10	3.88
4333	2.31, 8	5	-	20	0.50	15	5	.50	18.98	13.78	27.34	21.46	21.48	13.22	26.54	19.52	19.56	14.50
4334	2.31, 8	5	0	40	0	20	20	.50	6.68	5.90	12.34	8.38	8.42	6.38	10.36	6.84	6.84	6.08
4335	2.31, 8	5	+	40	0.16	17	23	.50	5.08	5.16	9.12	6.02	6.02	6.00	7.72	4.96	4.96	5.46
4336	2.31, 8	5	-	40	0.16	23	17	.50	9.38	7.64	15.80	11.26	11.26	7.72	14.14	9.66	9.66	7.58
4337	2.31, 8	5	+	40	0.33	13	27	.50	2.78	4.28	5.82	3.70	3.70	5.68	4.60	2.86	2.86	4.32
4338	2.31, 8	5	-	40	0.33	27	13	.50	13.88	9.06	20.34	15.24	15.24	8.48	19.52	13.90	13.90	9.36
4339	2.31, 8	5	+	40	0.50	10	30	.50	1.90	4.06	3.70	2.24	2.26	6.12	3.36	1.86	1.88	3.80
4340	2.31, 8	5	-	40	0.50	30	10	.50	18.28	10.96	24.96	19.20	19.22	10.38	24.36	18.40	18.40	11.12
4341	2.31, 8	5	0	60	0	30	30	.50	6.32	5.60	11.20	7.62	7.62	6.32	9.52	6.30	6.30	5.62
4342	2.31, 8	5	+	60	0.16	25	35	.50	4.02	4.84	7.58	5.30	5.30	5.92	6.74	4.04	4.04	5.00
4343	2.31, 8	5	-	60	0.16	35	25	.50	9.24	6.60	14.96	10.86	10.86	7.10	13.24	9.26	9.26	6.52
4344	2.31, 8	5	+	60	0.33	20	40	.50	2.58	4.36	5.40	3.36	3.36	5.66	4.18	2.52	2.52	4.30
4345	2.31, 8	5	-	60	0.33	40	20	.50	12.82	7.66	19.66	14.88	14.88	7.70	18.18	13.26	13.26	7.90
4346	2.31, 8	5	+	60	0.50	15	45	.50	1.80	4.26	3.32	1.78	1.78	5.62	2.94	1.98	1.98	4.36
4347	2.31, 8	5	-	60	0.50	45	15	.50	18.66	9.66	24.46	19.34	19.34	9.34	24.14	18.34	18.34	10.08
4348	2.31, 8	5	0	80	0	40	40	.50	6.46	5.58	10.44	7.14	7.14	6.22	9.68	6.46	6.46	5.62
4349	2.31, 8	5	+	80	0.16	34	46	.50	4.38	5.46	7.92	5.04	5.04	5.82	7.06	4.34	4.34	5.28
4350	2.31, 8	5	-	80	0.16	46	34	.50	8.62	6.02	13.92	9.60	9.60	6.34	12.88	9.04	9.04	6.18

	D	VR	P	N	Δn	n1	n2	ε	F g	B-F g	F t	F-GG t	F-HF t	B-F t	F g x t	F-GG g x t	F-HF g x t	B-F g x t
4351	2.31, 8	5	+	80	0.33	27	53	.50	2.34	4.90	4.94	2.76	2.76	5.28	4.44	2.66	2.66	5.14
4352	2.31, 8	5	-	80	0.33	53	27	.50	12.36	7.32	18.82	13.52	13.54	7.00	17.04	12.18	12.18	7.20
4353	2.31, 8	5	+	80	0.50	20	60	.50	1.30	4.58	2.72	1.50	1.50	5.28	2.50	1.42	1.42	4.66
4354	2.31, 8	5	-	80	0.50	60	20	.50	17.18	8.60	23.94	18.78	18.78	8.20	23.28	17.30	17.30	8.74
4355	2.31, 8	5	0	100	0	50	50	.50	6.12	5.58	10.20	7.12	7.12	5.90	9.64	6.04	6.04	5.68
4356	2.31, 8	5	+	100	0.16	42	58	.50	3.90	4.94	6.92	4.42	4.42	5.20	6.52	3.74	3.74	4.80
4357	2.31, 8	5	-	100	0.16	58	42	.50	9.12	6.08	13.88	9.80	9.80	6.26	13.60	8.76	8.76	6.02
4358	2.31, 8	5	+	100	0.33	33	67	.50	2.34	4.70	4.30	2.56	2.56	5.14	4.12	2.40	2.40	4.64
4359	2.31, 8	5	-	100	0.33	67	33	.50	12.72	6.70	18.14	13.86	13.88	6.74	17.54	12.74	12.74	6.76
4360	2.31, 8	5	+	100	0.50	25	75	.50	1.20	4.52	2.54	1.28	1.28	5.52	2.12	1.18	1.18	4.26
4361	2.31, 8	5	-	100	0.50	75	25	.50	17.54	7.42	23.50	18.74	18.76	7.88	22.98	17.22	17.22	7.60
4362	2.31, 8	5	0	150	0	75	75	.50	5.74	5.22	9.26	6.04	6.04	5.48	9.92	5.82	5.82	5.36
4363	2.31, 8	5	+	150	0.16	63	87	.50	4.04	5.50	6.24	3.86	3.86	4.90	6.78	4.08	4.08	5.64
4364	2.31, 8	5	-	150	0.16	87	63	.50	9.32	6.38	13.56	9.20	9.20	6.02	13.72	9.24	9.24	6.24
4365	2.31, 8	5	+	150	0.33	50	100	.50	2.02	4.82	3.66	2.20	2.20	4.64	4.10	2.08	2.08	4.88
4366	2.31, 8	5	-	150	0.33	100	50	.50	12.84	6.64	18.12	13.44	13.44	6.70	17.96	12.86	12.86	6.68
4367	2.31, 8	5	+	150	0.50	37	113	.50	1.10	4.52	1.84	.98	.98	4.68	2.16	1.16	1.16	4.72
4368	2.31, 8	5	-	150	0.50	113	37	.50	18.00	6.92	23.50	18.64	18.64	6.90	24.18	18.50	18.50	7.12
4369	2.31, 8	5	0	200	0	100	100	.50	5.50	5.12	9.50	5.92	5.92	5.18	9.06	5.44	5.44	5.06
4370	2.31, 8	5	+	200	0.16	84	116	.50	3.62	5.02	6.00	3.64	3.64	4.88	6.02	3.60	3.60	4.92
4371	2.31, 8	5	-	200	0.16	116	84	.50	8.54	5.74	13.34	8.94	8.94	5.58	12.60	8.66	8.66	5.74
4372	2.31, 8	5	+	200	0.33	67	133	.50	1.96	4.60	3.44	1.96	1.96	4.64	3.66	2.04	2.04	4.84
4373	2.31, 8	5	-	200	0.33	133	67	.50	13.38	6.44	17.72	12.68	12.68	5.76	18.22	13.24	13.24	6.68
4374	2.31, 8	5	+	200	0.50	50	150	.50	.80	4.48	1.86	.92	.92	4.68	1.60	.86	.86	4.44
4375	2.31, 8	5	-	200	0.50	150	50	.50	17.40	6.80	23.30	17.72	17.72	6.96	23.30	17.56	17.56	7.06
4376	2.31, 8	1	0	20	0	10	10	.60	3.88	2.92	9.64	7.46	7.66	6.90	6.96	4.28	4.48	3.08
4377	2.31, 8	1	0	20	0.16	8	12	.60	4.24	3.58	9.98	7.52	7.72	6.94	6.22	4.04	4.14	3.04
4378	2.31, 8	1	0	20	0.33	7	13	.60	4.62	4.06	9.78	7.66	7.86	7.34	6.88	4.34	4.48	3.82
4379	2.31, 8	1	0	20	0.50	5	15	.60	4.22	6.10	9.88	7.24	7.44	8.56	6.78	4.00	4.18	5.26
4380	2.31, 8	1	0	40	0	20	20	.60	5.04	4.28	8.80	6.36	6.50	5.88	7.26	4.80	4.96	3.94
4381	2.31, 8	1	0	40	0.16	17	23	.60	4.68	4.22	9.10	6.42	6.58	6.12	6.90	4.50	4.62	4.18
4382	2.31, 8	1	0	40	0.33	13	27	.60	4.26	4.72	8.72	6.20	6.34	6.14	7.72	4.70	4.82	4.90

	D	VR	P	N	Δn	n1	n2	ε	F g	B-F g	F t	F-GG t	F-HF t	B-F t	F g x t	F-GG g x t	F-HF g x t	B-F g x t
4383	2.31, 8	1	0	40	0.50	10	30	.60	4.30	5.70	8.56	5.86	5.92	7.24	7.36	4.64	4.72	5.74
4384	2.31, 8	1	0	60	0	30	30	.60	5.02	4.62	8.16	5.92	5.92	5.42	7.14	4.92	4.94	4.28
4385	2.31, 8	1	0	60	0.16	25	35	.60	4.92	4.74	8.60	5.86	6.02	5.68	7.36	4.72	4.84	4.46
4386	2.31, 8	1	0	60	0.33	20	40	.60	4.88	5.24	8.40	6.00	6.06	6.00	7.56	4.80	4.82	5.24
4387	2.31, 8	1	0	60	0.50	15	45	.60	4.76	6.30	8.14	5.90	5.92	6.64	7.10	4.68	4.72	5.58
4388	2.31, 8	1	0	80	0	40	40	.60	4.30	4.06	9.18	6.66	6.70	6.14	6.72	4.74	4.74	4.26
4389	2.31, 8	1	0	80	0.16	34	46	.60	4.64	4.70	8.88	6.12	6.14	6.08	6.98	4.94	4.98	4.54
4390	2.31, 8	1	0	80	0.33	27	53	.60	4.74	4.94	8.64	6.10	6.16	6.20	6.86	4.64	4.68	4.74
4391	2.31, 8	1	0	80	0.50	20	60	.60	5.08	6.46	8.36	6.06	6.12	6.58	7.30	5.02	5.08	6.18
4392	2.31, 8	1	0	100	0	50	50	.60	5.04	5.02	8.62	6.24	6.28	5.78	6.84	4.22	4.24	4.02
4393	2.31, 8	1	0	100	0.16	42	58	.60	4.70	4.80	8.44	6.00	6.04	5.76	7.00	4.74	4.74	4.84
4394	2.31, 8	1	0	100	0.33	33	67	.60	4.58	5.18	7.98	5.78	5.90	5.86	7.00	4.36	4.40	4.94
4395	2.31, 8	1	0	100	0.50	25	75	.60	4.44	5.80	8.04	5.78	5.80	6.28	7.04	4.34	4.34	5.64
4396	2.31, 8	1	0	150	0	75	75	.60	5.54	5.44	8.62	6.00	6.00	5.78	7.68	5.56	5.58	5.64
4397	2.31, 8	1	0	150	0.16	63	87	.60	5.14	5.24	8.64	6.08	6.10	5.94	7.26	4.66	4.70	4.74
4398	2.31, 8	1	0	150	0.33	50	100	.60	4.82	5.30	8.46	6.04	6.04	5.96	7.00	4.54	4.56	5.10
4399	2.31, 8	1	0	150	0.50	37	113	.60	4.96	5.84	8.20	5.56	5.60	6.24	7.16	4.68	4.68	5.26
4400	2.31, 8	1	0	200	0	100	100	.60	5.10	5.04	8.52	5.96	5.96	5.74	7.46	5.10	5.12	5.12
4401	2.31, 8	1	0	200	0.16	84	116	.60	5.18	5.18	8.80	6.16	6.16	6.04	8.04	5.46	5.46	5.54
4402	2.31, 8	1	0	200	0.33	67	133	.60	4.78	4.94	8.76	5.94	5.98	5.78	7.46	5.34	5.34	5.00
4403	2.31, 8	1	0	200	0.50	50	150	.60	4.50	5.48	8.28	6.06	6.08	6.26	6.88	4.38	4.38	5.28
4404	2.31, 8	1.5	0	20	0	10	10	.60	4.16	3.12	9.92	7.52	7.76	6.76	7.28	4.66	4.88	3.50
4405	2.31, 8	1.5	+	20	0.16	8	12	.60	3.92	3.32	8.92	6.74	7.06	6.72	5.78	3.74	3.90	2.84
4406	2.31, 8	1.5	-	20	0.16	12	8	.60	5.32	4.02	11.04	8.58	8.78	7.34	8.16	5.42	5.76	3.86
4407	2.31, 8	1.5	+	20	0.33	7	13	.60	3.64	3.34	8.32	6.46	6.66	6.80	5.60	3.50	3.68	3.12
4408	2.31, 8	1.5	-	20	0.33	13	7	.60	5.52	4.92	11.64	8.88	9.16	7.62	9.10	5.92	6.16	4.30
4409	2.31, 8	1.5	+	20	0.50	5	15	.60	3.36	4.98	7.32	5.52	5.72	8.20	4.48	2.90	3.00	4.16
4410	2.31, 8	1.5	-	20	0.50	15	5	.60	6.86	8.16	12.60	9.58	9.76	9.34	10.24	7.04	7.42	6.16
4411	2.31, 8	1.5	0	40	0	20	20	.60	5.00	4.36	8.90	6.46	6.54	5.94	7.64	4.72	4.78	4.08
4412	2.31, 8	1.5	+	40	0.16	17	23	.60	4.28	4.22	8.24	5.78	5.88	6.12	6.46	4.06	4.14	3.96
4413	2.31, 8	1.5	-	40	0.16	23	17	.60	5.56	4.80	9.54	7.10	7.18	5.98	7.94	5.42	5.52	4.30
4414	2.31, 8	1.5	+	40	0.33	13	27	.60	3.38	4.10	6.72	4.82	4.90	6.10	5.74	3.44	3.58	4.04

	D	VR	P	N	Δn	n1	n2	ε	F g	B-F g	F t	F-GG t	F-HF t	B-F t	F g x t	F-GG g x t	F-HF g x t	B-F g x t
4415	2.31, 8	1.5	-	40	0.33	27	13	.60	6.38	5.50	11.06	7.88	8.02	6.72	8.90	6.38	6.44	4.96
4416	2.31, 8	1.5	+	40	0.50	10	30	.60	2.86	4.88	5.80	4.20	4.20	6.42	4.94	3.14	3.20	4.90
4417	2.31, 8	1.5	-	40	0.50	30	10	.60	6.96	7.14	11.92	8.58	8.70	7.88	9.92	7.12	7.20	6.72
4418	2.31, 8	1.5	0	60	0	30	30	.60	4.74	4.40	8.24	5.92	5.96	5.48	7.50	4.92	4.98	4.38
4419	2.31, 8	1.5	+	60	0.16	25	35	.60	4.20	4.34	7.36	5.24	5.26	5.40	6.60	4.10	4.14	4.40
4420	2.31, 8	1.5	-	60	0.16	35	25	.60	5.66	4.44	9.36	6.96	7.04	6.08	8.40	5.52	5.60	4.46
4421	2.31, 8	1.5	+	60	0.33	20	40	.60	3.64	4.90	6.54	4.74	4.78	5.78	5.42	3.68	3.70	5.00
4422	2.31, 8	1.5	-	60	0.33	40	20	.60	6.34	5.54	10.52	7.68	7.70	6.18	9.52	6.16	6.22	5.22
4423	2.31, 8	1.5	+	60	0.50	15	45	.60	3.24	5.22	5.84	3.82	3.86	5.96	4.74	3.04	3.08	5.10
4424	2.31, 8	1.5	-	60	0.50	45	15	.60	7.46	6.58	11.70	8.70	8.76	7.06	10.62	7.20	7.28	6.16
4425	2.31, 8	1.5	0	80	0	40	40	.60	4.58	4.52	9.50	6.90	6.92	6.32	7.30	4.86	4.92	4.48
4426	2.31, 8	1.5	+	80	0.16	34	46	.60	3.90	4.26	8.20	5.74	5.78	6.10	6.28	4.30	4.30	4.62
4427	2.31, 8	1.5	-	80	0.16	46	34	.60	5.40	4.68	10.64	7.94	7.94	6.82	7.78	5.12	5.16	4.88
4428	2.31, 8	1.5	+	80	0.33	27	53	.60	3.58	4.52	7.04	4.96	5.00	5.96	5.34	3.36	3.36	4.40
4429	2.31, 8	1.5	-	80	0.33	53	27	.60	6.26	4.98	11.82	8.56	8.64	6.70	9.06	6.08	6.14	4.94
4430	2.31, 8	1.5	+	80	0.50	20	60	.60	3.20	5.74	5.70	4.06	4.06	6.28	4.70	3.06	3.08	5.40
4431	2.31, 8	1.5	-	80	0.50	60	20	.60	7.10	6.58	12.82	9.66	9.66	7.72	10.10	7.24	7.28	5.82
4432	2.31, 8	1.5	0	100	0	50	50	.60	5.02	4.68	8.68	6.18	6.20	6.08	7.10	4.36	4.38	4.18
4433	2.31, 8	1.5	+	100	0.16	42	58	.60	4.08	4.52	7.76	5.38	5.40	5.72	6.14	4.24	4.24	4.52
4434	2.31, 8	1.5	-	100	0.16	58	42	.60	5.60	4.82	9.62	6.96	7.00	6.08	8.06	5.72	5.76	4.94
4435	2.31, 8	1.5	+	100	0.33	33	67	.60	3.44	4.76	6.64	4.42	4.46	5.68	5.22	3.28	3.28	4.66
4436	2.31, 8	1.5	-	100	0.33	67	33	.60	7.24	5.88	10.74	7.50	7.56	5.98	9.48	6.82	6.84	5.46
4437	2.31, 8	1.5	+	100	0.50	25	75	.60	2.66	5.18	5.58	3.78	3.80	6.10	4.28	2.64	2.64	5.06
4438	2.31, 8	1.5	-	100	0.50	75	25	.60	8.04	6.30	11.26	8.08	8.12	5.94	10.88	7.50	7.56	6.02
4439	2.31, 8	1.5	0	150	0	75	75	.60	5.60	5.40	8.64	5.92	5.92	5.64	7.82	5.66	5.70	5.72
4440	2.31, 8	1.5	+	150	0.16	63	87	.60	4.84	5.18	7.56	5.34	5.38	5.70	6.56	4.28	4.30	4.94
4441	2.31, 8	1.5	-	150	0.16	87	63	.60	5.96	5.32	9.48	6.96	6.98	5.92	9.28	6.24	6.26	5.30
4442	2.31, 8	1.5	+	150	0.33	50	100	.60	3.66	5.06	6.60	4.48	4.50	6.06	5.52	3.42	3.42	4.94
4443	2.31, 8	1.5	-	150	0.33	100	50	.60	6.84	5.56	10.76	7.54	7.60	5.96	9.62	6.74	6.74	5.44
4444	2.31, 8	1.5	+	150	0.50	37	113	.60	2.96	5.44	5.34	3.64	3.64	5.92	4.68	2.70	2.70	4.92
4445	2.31, 8	1.5	-	150	0.50	113	37	.60	7.50	5.94	10.88	8.50	8.50	6.18	10.54	7.26	7.26	5.72
4446	2.31, 8	1.5	0	200	0	100	100	.60	5.16	5.06	8.46	5.76	5.76	5.60	7.58	5.22	5.22	5.10

	D	VR	P	N	Δn	n1	n2	ε	F g	B-F g	F t	F-GG t	F-HF t	B-F t	F g x t	F-GG g x t	F-HF g x t	B-F g x t
4447	2.31, 8	1.5	+	200	0.16	84	116	.60	4.28	5.22	7.86	5.42	5.42	5.94	7.02	4.90	4.92	5.46
4448	2.31, 8	1.5	-	200	0.16	116	84	.60	5.90	5.42	9.12	6.40	6.40	5.52	8.48	5.70	5.74	5.08
4449	2.31, 8	1.5	+	200	0.33	67	133	.60	3.68	4.98	6.92	4.54	4.54	5.84	5.74	3.88	3.88	4.92
4450	2.31, 8	1.5	-	200	0.33	133	67	.60	6.70	5.28	10.90	7.52	7.54	6.14	10.12	7.02	7.06	6.00
4451	2.31, 8	1.5	+	200	0.50	50	150	.60	2.80	5.06	5.98	3.94	3.94	6.18	4.32	2.68	2.70	5.02
4452	2.31, 8	1.5	-	200	0.50	150	50	.60	7.54	5.76	11.78	8.62	8.62	6.84	11.00	8.08	8.08	6.38
4453	2.31, 8	2	0	20	0	10	10	.60	4.58	3.44	10.26	7.74	7.90	6.98	7.60	5.04	5.26	3.94
4454	2.31, 8	2	+	20	0.16	8	12	.60	3.92	3.50	8.40	6.16	6.46	6.60	5.64	3.70	3.88	3.26
4455	2.31, 8	2	-	20	0.16	12	8	.60	6.32	4.68	12.10	9.34	9.64	7.78	9.58	6.40	6.68	4.38
4456	2.31, 8	2	+	20	0.33	7	13	.60	3.44	3.62	7.56	5.72	5.82	7.20	4.86	3.04	3.20	3.50
4457	2.31, 8	2	-	20	0.33	13	7	.60	7.08	6.00	13.12	10.06	10.38	7.84	10.78	7.10	7.38	5.06
4458	2.31, 8	2	+	20	0.50	5	15	.60	2.80	4.20	5.82	4.30	4.56	8.00	3.68	2.46	2.52	3.62
4459	2.31, 8	2	-	20	0.50	15	5	.60	9.24	9.50	15.18	11.56	11.86	9.84	12.72	9.26	9.44	7.06
4460	2.31, 8	2	0	40	0	20	20	.60	5.22	4.56	8.92	6.60	6.76	5.96	7.90	4.94	5.08	4.24
4461	2.31, 8	2	+	40	0.16	17	23	.60	4.04	4.58	7.72	5.32	5.42	5.96	6.14	3.78	3.88	4.16
4462	2.31, 8	2	-	40	0.16	23	17	.60	6.28	5.14	10.40	7.78	7.88	6.16	9.06	6.16	6.28	4.76
4463	2.31, 8	2	+	40	0.33	13	27	.60	2.88	3.86	5.86	3.90	4.00	5.88	4.82	2.90	3.00	3.88
4464	2.31, 8	2	-	40	0.33	27	13	.60	7.82	6.22	12.50	9.54	9.64	6.94	10.80	7.78	7.92	5.70
4465	2.31, 8	2	+	40	0.50	10	30	.60	2.36	4.64	4.40	3.04	3.14	6.68	3.78	2.40	2.44	4.62
4466	2.31, 8	2	-	40	0.50	30	10	.60	9.60	8.32	14.48	11.16	11.22	8.60	13.14	9.44	9.54	7.82
4467	2.31, 8	2	0	60	0	30	30	.60	5.12	4.56	8.32	5.94	6.02	5.26	7.54	4.98	5.04	4.46
4468	2.31, 8	2	+	60	0.16	25	35	.60	4.00	4.34	6.76	4.88	4.96	5.34	5.94	3.92	3.94	4.30
4469	2.31, 8	2	-	60	0.16	35	25	.60	6.24	4.78	10.30	7.72	7.78	6.06	9.34	6.30	6.36	4.80
4470	2.31, 8	2	+	60	0.33	20	40	.60	3.32	4.72	5.44	3.98	4.06	5.40	4.62	3.22	3.24	4.82
4471	2.31, 8	2	-	60	0.33	40	20	.60	7.92	5.96	12.26	8.86	8.88	6.48	11.60	8.14	8.20	5.72
4472	2.31, 8	2	+	60	0.50	15	45	.60	2.40	4.80	4.38	2.72	2.80	5.76	3.66	2.26	2.30	4.54
4473	2.31, 8	2	-	60	0.50	45	15	.60	9.86	7.24	13.96	10.84	10.88	7.12	13.24	9.62	9.78	6.56
4474	2.31, 8	2	0	80	0	40	40	.60	4.82	4.60	9.48	7.02	7.04	6.30	7.62	5.12	5.16	5.00
4475	2.31, 8	2	+	80	0.16	34	46	.60	3.68	4.22	7.90	5.56	5.62	6.20	5.98	4.00	4.04	4.82
4476	2.31, 8	2	-	80	0.16	46	34	.60	6.14	4.70	11.12	8.70	8.72	6.96	8.66	5.90	5.94	5.22
4477	2.31, 8	2	+	80	0.33	27	53	.60	3.08	4.58	5.94	4.12	4.18	5.92	4.46	2.86	2.88	4.78
4478	2.31, 8	2	-	80	0.33	53	27	.60	7.46	5.30	13.34	10.00	10.04	6.82	10.72	7.52	7.58	5.08

	D	VR	P	N	Δn	n1	n2	ε	F g	B-F g	F t	F-GG t	F-HF t	B-F t	F g x t	F-GG g x t	F-HF g x t	B-F g x t
4479	2.31, 8	2	+	80	0.50	20	60	.60	2.44	5.52	4.48	3.12	3.14	6.24	3.66	2.34	2.34	5.20
4480	2.31, 8	2	-	80	0.50	60	20	.60	9.58	6.86	15.62	11.76	11.86	7.80	12.58	9.42	9.42	6.18
4481	2.31, 8	2	0	100	0	50	50	.60	4.96	4.72	8.84	6.38	6.40	6.04	7.44	4.52	4.58	4.40
4482	2.31, 8	2	+	100	0.16	42	58	.60	3.84	4.56	7.24	5.14	5.18	5.78	5.60	4.10	4.14	4.56
4483	2.31, 8	2	-	100	0.16	58	42	.60	6.28	5.12	10.68	7.60	7.66	6.10	8.80	6.38	6.40	4.94
4484	2.31, 8	2	+	100	0.33	33	67	.60	2.76	4.74	5.54	3.64	3.68	5.60	4.30	2.80	2.80	4.62
4485	2.31, 8	2	-	100	0.33	67	33	.60	8.58	6.12	12.26	9.02	9.12	5.92	11.10	8.00	8.00	5.52
4486	2.31, 8	2	+	100	0.50	25	75	.60	1.94	4.80	4.26	2.86	2.88	5.96	3.18	1.92	1.94	4.70
4487	2.31, 8	2	-	100	0.50	75	25	.60	10.24	6.60	13.76	10.12	10.20	6.22	13.54	9.94	9.94	6.60
4488	2.31, 8	2	0	150	0	75	75	.60	5.88	5.64	8.54	6.08	6.14	5.86	7.88	5.64	5.64	5.64
4489	2.31, 8	2	+	150	0.16	63	87	.60	4.52	5.14	7.00	4.94	4.94	5.84	5.98	4.08	4.08	5.00
4490	2.31, 8	2	-	150	0.16	87	63	.60	6.60	5.34	10.24	7.64	7.64	5.98	10.06	6.96	6.96	5.40
4491	2.31, 8	2	+	150	0.33	50	100	.60	3.00	4.98	5.44	3.62	3.62	5.76	4.52	2.66	2.66	4.82
4492	2.31, 8	2	-	150	0.33	100	50	.60	8.10	5.34	12.50	9.14	9.18	5.86	11.50	8.16	8.16	5.32
4493	2.31, 8	2	+	150	0.50	37	113	.60	2.24	4.98	4.08	2.74	2.76	5.82	3.22	1.92	1.92	4.72
4494	2.31, 8	2	-	150	0.50	113	37	.60	9.94	6.00	14.24	10.26	10.30	6.28	13.32	9.66	9.70	5.74
4495	2.31, 8	2	0	200	0	100	100	.60	5.10	5.20	8.42	5.66	5.68	5.64	7.80	5.26	5.26	5.30
4496	2.31, 8	2	+	200	0.16	84	116	.60	3.84	5.12	7.16	4.78	4.82	5.88	6.40	4.50	4.50	5.48
4497	2.31, 8	2	-	200	0.16	116	84	.60	6.40	5.28	9.94	6.86	6.94	5.74	9.34	6.56	6.58	5.28
4498	2.31, 8	2	+	200	0.33	67	133	.60	2.76	4.96	5.64	3.70	3.74	5.82	4.72	3.14	3.14	5.08
4499	2.31, 8	2	-	200	0.33	133	67	.60	8.08	5.48	12.74	9.00	9.00	6.10	11.86	8.58	8.58	6.00
4500	2.31, 8	2	+	200	0.50	50	150	.60	1.94	4.72	4.50	2.86	2.86	6.10	3.04	1.98	1.98	4.98
4501	2.31, 8	2	-	200	0.50	150	50	.60	9.86	5.90	14.44	11.04	11.08	6.52	13.90	10.26	10.26	6.52
4502	2.31, 8	5	0	20	0	10	10	.60	6.70	5.78	10.72	8.48	8.72	7.26	9.44	7.14	7.28	5.80
4503	2.31, 8	5	+	20	0.16	8	12	.60	4.64	4.82	7.18	5.58	5.82	6.90	5.66	4.24	4.48	4.74
4504	2.31, 8	5	-	20	0.16	12	8	.60	10.66	7.62	15.02	12.08	12.34	8.48	14.20	10.72	10.92	7.04
4505	2.31, 8	5	+	20	0.33	7	13	.60	3.56	4.76	5.62	4.32	4.54	6.80	4.20	3.18	3.28	4.34
4506	2.31, 8	5	-	20	0.33	13	7	.60	12.90	8.72	17.84	14.24	14.62	9.26	17.56	12.94	13.32	7.92
4507	2.31, 8	5	+	20	0.50	5	15	.60	2.14	3.94	3.30	2.42	2.54	7.02	2.52	1.54	1.74	3.66
4508	2.31, 8	5	-	20	0.50	15	5	.60	19.70	13.02	24.20	19.68	20.00	11.10	23.92	18.54	18.94	10.68
4509	2.31, 8	5	0	40	0	20	20	.60	6.18	5.50	9.08	7.04	7.08	6.12	8.72	6.38	6.46	5.92
4510	2.31, 8	5	+	40	0.16	17	23	.60	4.22	5.00	6.42	4.76	4.86	6.02	5.62	3.82	3.92	5.04

	D	VR	P	N	Δn	n1	n2	ε	F g	B-F g	F t	F-GG t	F-HF t	B-F t	F g x t	F-GG g x t	F-HF g x t	B-F g x t
4511	2.31, 8	5	-	40	0.16	23	17	.60	8.74	6.38	12.72	9.84	9.98	6.80	12.28	9.12	9.20	6.28
4512	2.31, 8	5	+	40	0.33	13	27	.60	2.34	4.14	3.50	2.42	2.48	5.74	3.24	2.10	2.10	4.32
4513	2.31, 8	5	-	40	0.33	27	13	.60	13.56	7.28	18.12	14.44	14.60	7.36	17.04	12.92	12.94	7.18
4514	2.31, 8	5	+	40	0.50	10	30	.60	1.44	3.64	2.08	1.40	1.46	5.86	1.96	1.40	1.42	4.28
4515	2.31, 8	5	-	40	0.50	30	10	.60	17.54	9.22	22.98	18.86	19.02	8.84	22.70	17.72	17.94	8.80
4516	2.31, 8	5	0	60	0	30	30	.60	5.68	4.82	9.12	6.80	6.84	5.72	8.04	5.80	5.82	5.12
4517	2.31, 8	5	+	60	0.16	25	35	.60	3.70	4.50	5.76	4.06	4.16	5.48	5.20	3.54	3.56	4.90
4518	2.31, 8	5	-	60	0.16	35	25	.60	8.82	5.24	12.50	9.88	9.92	6.34	12.28	9.10	9.16	6.02
4519	2.31, 8	5	+	60	0.33	20	40	.60	2.40	4.40	3.56	2.44	2.50	5.38	3.20	2.12	2.12	4.44
4520	2.31, 8	5	-	60	0.33	40	20	.60	12.18	6.70	17.22	13.42	13.48	6.90	17.14	13.46	13.48	6.78
4521	2.31, 8	5	+	60	0.50	15	45	.60	1.44	4.32	1.98	1.38	1.40	5.10	1.72	1.10	1.14	4.14
4522	2.31, 8	5	-	60	0.50	45	15	.60	17.84	8.10	22.94	18.62	18.70	8.04	22.70	18.28	18.38	7.50
4523	2.31, 8	5	0	80	0	40	40	.60	5.54	5.06	9.88	7.18	7.26	6.52	8.34	5.96	6.06	5.76
4524	2.31, 8	5	+	80	0.16	34	46	.60	3.74	4.80	7.08	4.74	4.80	6.78	5.44	3.76	3.76	5.28
4525	2.31, 8	5	-	80	0.16	46	34	.60	8.32	5.74	13.18	10.28	10.30	7.02	11.42	8.50	8.54	6.06
4526	2.31, 8	5	+	80	0.33	27	53	.60	2.14	4.58	4.06	2.56	2.60	6.18	3.18	2.18	2.18	4.82
4527	2.31, 8	5	-	80	0.33	53	27	.60	12.12	5.94	17.84	14.50	14.58	7.02	16.32	12.62	12.74	6.04
4528	2.31, 8	5	+	80	0.50	20	60	.60	1.46	4.84	1.86	1.24	1.28	6.26	1.80	1.18	1.20	4.72
4529	2.31, 8	5	-	80	0.50	60	20	.60	17.36	7.36	24.42	19.44	19.52	8.16	22.52	17.82	17.88	7.06
4530	2.31, 8	5	0	100	0	50	50	.60	5.32	5.00	9.04	6.74	6.74	6.14	8.02	5.68	5.70	5.16
4531	2.31, 8	5	+	100	0.16	42	58	.60	3.22	4.48	6.04	4.20	4.20	5.90	4.88	3.54	3.62	4.84
4532	2.31, 8	5	-	100	0.16	58	42	.60	8.30	5.50	12.36	9.24	9.32	6.14	11.42	8.22	8.26	5.40
4533	2.31, 8	5	+	100	0.33	33	67	.60	1.64	4.38	3.42	2.32	2.34	5.64	2.72	1.64	1.64	4.88
4534	2.31, 8	5	-	100	0.33	67	33	.60	12.72	6.28	17.50	13.24	13.30	6.12	16.08	12.66	12.74	6.14
4535	2.31, 8	5	+	100	0.50	25	75	.60	.76	4.34	1.88	1.10	1.12	5.70	1.42	.82	.82	4.48
4536	2.31, 8	5	-	100	0.50	75	25	.60	17.74	7.16	22.10	17.34	17.40	6.66	21.96	17.58	17.62	6.88
4537	2.31, 8	5	0	150	0	75	75	.60	5.88	5.78	8.66	6.02	6.02	5.72	8.26	6.00	6.00	5.92
4538	2.31, 8	5	+	150	0.16	63	87	.60	3.70	5.20	5.84	3.86	3.92	5.72	5.42	3.66	3.66	5.38
4539	2.31, 8	5	-	150	0.16	87	63	.60	8.78	5.66	12.50	9.60	9.66	5.80	11.78	8.98	8.98	5.80
4540	2.31, 8	5	+	150	0.33	50	100	.60	1.90	4.66	3.34	2.16	2.16	5.86	2.80	1.72	1.74	4.96
4541	2.31, 8	5	-	150	0.33	100	50	.60	12.36	5.66	16.92	13.18	13.24	5.96	16.64	12.90	12.90	5.86
4542	2.31, 8	5	+	150	0.50	37	113	.60	.82	4.50	1.76	1.10	1.10	5.92	1.38	.80	.82	4.72

	D	VR	P	N	Δn	n1	n2	ε	F g	B-F g	F t	F-GG t	F-HF t	B-F t	F g x t	F-GG g x t	F-HF g x t	B-F g x t
4543	2.31, 8	5	-	150	0.50	113	37	.60	17.68	6.28	23.00	18.30	18.32	6.72	22.36	17.84	17.88	6.56
4544	2.31, 8	5	0	200	0	100	100	.60	5.22	5.08	8.42	5.82	5.82	5.64	7.98	5.66	5.66	5.42
4545	2.31, 8	5	+	200	0.16	84	116	.60	3.14	5.22	5.44	3.68	3.70	5.62	5.44	3.84	3.86	5.70
4546	2.31, 8	5	-	200	0.16	116	84	.60	8.16	5.42	12.26	8.98	9.00	5.70	11.70	8.72	8.74	5.34
4547	2.31, 8	5	+	200	0.33	67	133	.60	1.60	4.82	3.34	2.12	2.12	5.66	2.98	1.90	1.90	5.08
4548	2.31, 8	5	-	200	0.33	133	67	.60	12.28	5.62	17.20	13.22	13.28	6.60	16.98	12.94	12.98	6.30
4549	2.31, 8	5	+	200	0.50	50	150	.60	.72	4.60	1.96	1.20	1.20	5.94	1.06	.68	.68	4.68
4550	2.31, 8	5	-	200	0.50	150	50	.60	17.26	6.14	22.68	18.54	18.54	6.60	22.74	18.10	18.12	6.60
4551	2.31, 8	1	0	20	0	10	10	.70	4.08	3.00	6.50	4.24	4.56	3.34	6.26	4.08	4.24	3.30
4552	2.31, 8	1	0	20	0.16	8	12	.70	4.12	3.54	6.52	4.20	4.64	3.50	5.98	3.78	4.26	3.16
4553	2.31, 8	1	0	20	0.33	7	13	.70	4.78	4.04	6.46	4.54	4.94	3.60	5.84	3.74	4.04	2.94
4554	2.31, 8	1	0	20	0.50	5	15	.70	4.48	6.82	6.62	4.82	5.10	4.86	6.88	4.62	5.00	4.56
4555	2.31, 8	1	0	40	0	20	20	.70	4.56	4.08	6.88	4.76	4.88	4.40	6.18	4.18	4.26	3.70
4556	2.31, 8	1	0	40	0.16	17	23	.70	4.32	4.00	6.90	5.02	5.18	4.60	6.34	4.18	4.34	3.92
4557	2.31, 8	1	0	40	0.33	13	27	.70	4.20	4.38	7.10	5.24	5.38	4.62	6.16	4.46	4.64	4.02
4558	2.31, 8	1	0	40	0.50	10	30	.70	4.08	5.92	7.28	5.22	5.32	5.08	6.26	4.72	4.88	4.84
4559	2.31, 8	1	0	60	0	30	30	.70	4.28	4.00	6.80	5.08	5.18	5.04	7.04	5.16	5.26	4.78
4560	2.31, 8	1	0	60	0.16	25	35	.70	4.86	4.46	6.74	4.90	4.96	4.94	6.58	4.76	4.78	4.46
4561	2.31, 8	1	0	60	0.33	20	40	.70	5.00	5.22	7.00	5.04	5.14	4.90	6.92	5.14	5.14	4.94
4562	2.31, 8	1	0	60	0.50	15	45	.70	4.88	5.92	6.82	5.08	5.14	5.40	6.76	4.62	4.70	4.48
4563	2.31, 8	1	0	80	0	40	40	.70	4.54	4.46	6.84	5.26	5.30	5.18	6.54	4.58	4.70	4.52
4564	2.31, 8	1	0	80	0.16	34	46	.70	4.58	4.54	7.00	5.22	5.24	5.40	6.50	4.50	4.58	4.82
4565	2.31, 8	1	0	80	0.33	27	53	.70	4.64	4.96	7.20	5.50	5.54	5.78	6.62	4.60	4.70	4.92
4566	2.31, 8	1	0	80	0.50	20	60	.70	4.82	6.08	7.42	5.50	5.52	5.90	6.82	5.10	5.18	4.84
4567	2.31, 8	1	0	100	0	50	50	.70	4.52	4.64	7.06	5.50	5.54	5.26	6.26	4.52	4.54	4.48
4568	2.31, 8	1	0	100	0.16	42	58	.70	4.72	4.64	7.02	5.48	5.52	5.48	6.44	4.64	4.68	4.58
4569	2.31, 8	1	0	100	0.33	33	67	.70	4.56	5.06	7.24	5.38	5.40	5.72	6.56	4.68	4.72	4.70
4570	2.31, 8	1	0	100	0.50	25	75	.70	4.46	5.96	7.30	5.52	5.58	5.60	6.12	4.56	4.64	4.58
4571	2.31, 8	1	0	150	0	75	75	.70	5.40	5.24	6.84	5.04	5.06	5.10	6.88	5.04	5.08	5.10
4572	2.31, 8	1	0	150	0.16	63	87	.70	5.18	5.14	6.82	5.16	5.20	5.34	6.94	5.02	5.04	5.28
4573	2.31, 8	1	0	150	0.33	50	100	.70	4.82	4.98	6.96	5.06	5.08	5.12	6.80	4.96	4.98	5.26
4574	2.31, 8	1	0	150	0.50	37	113	.70	4.68	5.74	6.88	5.12	5.16	5.24	6.72	5.00	5.08	5.28

	D	VR	P	N	Δn	n1	n2	ε	F g	B-F g	F t	F-GG t	F-HF t	B-F t	F g x t	F-GG g x t	F-HF g x t	B-F g x t
4575	2.31, 8	1	0	200	0	100	100	.70	5.22	5.08	6.74	4.84	4.84	4.96	6.76	4.60	4.66	4.94
4576	2.31, 8	1	0	200	0.16	84	116	.70	4.88	5.20	6.94	5.20	5.20	5.08	6.76	5.36	5.36	5.16
4577	2.31, 8	1	0	200	0.33	67	133	.70	5.14	5.32	6.88	5.24	5.26	5.22	6.84	5.22	5.28	5.18
4578	2.31, 8	1	0	200	0.50	50	150	.70	4.34	5.50	7.14	4.94	5.00	5.08	7.18	5.18	5.22	5.48
4579	2.31, 8	1.5	0	20	0	10	10	.70	4.46	3.36	6.36	4.24	4.62	3.34	6.16	3.98	4.24	3.08
4580	2.31, 8	1.5	+	20	0.16	8	12	.70	4.06	3.14	5.48	3.66	3.84	3.42	5.08	3.28	3.56	3.10
4581	2.31, 8	1.5	-	20	0.16	12	8	.70	5.32	4.28	7.44	5.20	5.50	3.16	6.74	4.76	5.00	3.30
4582	2.31, 8	1.5	+	20	0.33	7	13	.70	4.10	3.90	4.90	3.32	3.60	3.94	4.36	2.80	2.98	3.02
4583	2.31, 8	1.5	-	20	0.33	13	7	.70	5.28	4.94	8.28	5.72	6.02	3.70	7.76	5.38	5.86	3.64
4584	2.31, 8	1.5	+	20	0.50	5	15	.70	3.42	5.12	4.38	3.12	3.28	4.38	4.30	2.92	3.14	4.16
4585	2.31, 8	1.5	-	20	0.50	15	5	.70	6.64	7.90	10.10	7.30	7.62	4.90	9.64	7.28	7.48	4.70
4586	2.31, 8	1.5	0	40	0	20	20	.70	4.68	3.84	6.72	4.90	4.98	4.40	6.10	4.18	4.30	3.68
4587	2.31, 8	1.5	+	40	0.16	17	23	.70	3.68	3.40	5.92	4.40	4.52	4.32	5.36	3.62	3.88	3.84
4588	2.31, 8	1.5	-	40	0.16	23	17	.70	5.28	4.44	7.62	5.46	5.68	4.64	7.04	5.16	5.28	4.40
4589	2.31, 8	1.5	+	40	0.33	13	27	.70	3.28	3.82	5.24	3.58	3.62	4.54	4.54	3.26	3.32	3.80
4590	2.31, 8	1.5	-	40	0.33	27	13	.70	5.96	5.40	8.74	6.56	6.68	4.54	8.48	6.04	6.22	4.44
4591	2.31, 8	1.5	+	40	0.50	10	30	.70	2.84	4.92	4.68	3.24	3.42	4.80	4.08	2.78	2.88	4.54
4592	2.31, 8	1.5	-	40	0.50	30	10	.70	6.82	7.02	9.78	7.04	7.26	4.88	9.52	7.32	7.44	4.56
4593	2.31, 8	1.5	0	60	0	30	30	.70	4.36	3.94	6.56	4.96	5.04	4.70	7.18	5.28	5.36	4.72
4594	2.31, 8	1.5	+	60	0.16	25	35	.70	4.10	4.26	5.88	4.32	4.40	4.98	5.98	4.20	4.26	4.76
4595	2.31, 8	1.5	-	60	0.16	35	25	.70	5.42	4.76	7.86	5.70	5.82	5.18	7.80	5.84	5.92	4.88
4596	2.31, 8	1.5	+	60	0.33	20	40	.70	3.80	4.78	5.18	3.72	3.76	4.84	5.16	3.74	3.76	4.60
4597	2.31, 8	1.5	-	60	0.33	40	20	.70	6.46	5.44	8.48	6.60	6.68	5.08	9.12	7.00	7.02	5.04
4598	2.31, 8	1.5	+	60	0.50	15	45	.70	3.26	5.32	4.64	3.36	3.46	5.12	4.06	2.78	2.78	4.50
4599	2.31, 8	1.5	-	60	0.50	45	15	.70	7.62	6.86	10.12	7.58	7.74	5.58	10.26	8.06	8.12	5.72
4600	2.31, 8	1.5	0	80	0	40	40	.70	4.66	4.40	6.94	5.20	5.30	5.04	6.32	4.60	4.62	4.42
4601	2.31, 8	1.5	+	80	0.16	34	46	.70	4.00	4.20	6.32	4.70	4.76	5.30	5.58	4.00	4.06	4.66
4602	2.31, 8	1.5	-	80	0.16	46	34	.70	5.74	5.04	8.04	5.76	5.88	5.04	7.42	5.44	5.46	4.44
4603	2.31, 8	1.5	+	80	0.33	27	53	.70	3.76	4.60	5.22	3.98	4.04	5.40	4.66	3.24	3.28	4.80
4604	2.31, 8	1.5	-	80	0.33	53	27	.70	6.26	5.22	9.14	6.78	6.88	4.84	8.70	6.60	6.62	5.14
4605	2.31, 8	1.5	+	80	0.50	20	60	.70	3.06	5.62	4.72	3.52	3.54	5.72	4.46	2.98	3.00	4.46
4606	2.31, 8	1.5	-	80	0.50	60	20	.70	7.14	6.44	10.66	8.34	8.38	5.80	10.32	8.14	8.18	5.70

	D	VR	P	N	Δn	n1	n2	ε	F g	B-F g	F t	F-GG t	F-HF t	B-F t	F g x t	F-GG g x t	F-HF g x t	B-F g x t
4607	2.31, 8	1.5	0	100	0	50	50	.70	4.88	4.84	7.02	5.28	5.30	5.32	6.32	4.54	4.54	4.60
4608	2.31, 8	1.5	+	100	0.16	42	58	.70	3.96	4.34	6.44	4.68	4.74	5.10	5.40	3.96	4.06	4.54
4609	2.31, 8	1.5	-	100	0.16	58	42	.70	5.98	5.36	7.88	5.88	5.98	5.24	7.70	5.56	5.68	4.78
4610	2.31, 8	1.5	+	100	0.33	33	67	.70	3.14	4.48	5.30	3.94	3.98	5.48	4.58	3.06	3.10	4.72
4611	2.31, 8	1.5	-	100	0.33	67	33	.70	6.94	5.42	8.96	6.78	6.88	5.12	8.72	6.32	6.34	4.72
4612	2.31, 8	1.5	+	100	0.50	25	75	.70	2.92	5.22	4.62	3.50	3.56	5.54	3.82	2.70	2.72	4.42
4613	2.31, 8	1.5	-	100	0.50	75	25	.70	8.10	6.70	10.30	8.06	8.18	5.06	9.54	7.04	7.06	4.72
4614	2.31, 8	1.5	0	150	0	75	75	.70	5.24	5.30	6.84	4.80	4.86	5.02	6.88	4.98	4.98	5.00
4615	2.31, 8	1.5	+	150	0.16	63	87	.70	4.58	5.20	5.88	4.28	4.30	5.12	5.86	4.38	4.38	5.46
4616	2.31, 8	1.5	-	150	0.16	87	63	.70	5.90	5.36	7.38	5.84	5.84	5.06	8.04	6.22	6.24	5.56
4617	2.31, 8	1.5	+	150	0.33	50	100	.70	3.56	4.96	4.98	3.58	3.60	5.34	5.00	3.56	3.58	4.98
4618	2.31, 8	1.5	-	150	0.33	100	50	.70	6.50	5.38	8.94	6.44	6.52	5.08	8.44	6.52	6.52	5.08
4619	2.31, 8	1.5	+	150	0.50	37	113	.70	2.66	5.40	4.38	3.22	3.24	5.22	4.10	3.00	3.00	5.16
4620	2.31, 8	1.5	-	150	0.50	113	37	.70	8.08	6.18	9.96	7.78	7.80	5.42	9.90	7.62	7.66	5.06
4621	2.31, 8	1.5	0	200	0	100	100	.70	5.30	5.24	6.54	5.00	5.04	4.90	6.72	4.70	4.72	4.84
4622	2.31, 8	1.5	+	200	0.16	84	116	.70	4.28	5.02	5.94	4.26	4.26	5.02	5.96	4.32	4.34	5.26
4623	2.31, 8	1.5	-	200	0.16	116	84	.70	5.66	5.24	7.62	5.44	5.48	4.96	7.86	5.94	5.96	5.50
4624	2.31, 8	1.5	+	200	0.33	67	133	.70	3.48	5.06	5.14	3.82	3.84	5.48	5.20	3.56	3.56	5.38
4625	2.31, 8	1.5	-	200	0.33	133	67	.70	6.32	5.36	8.50	6.54	6.56	4.70	8.78	6.60	6.66	5.18
4626	2.31, 8	1.5	+	200	0.50	50	150	.70	2.82	5.22	4.06	3.16	3.16	5.20	4.10	3.18	3.18	5.50
4627	2.31, 8	1.5	-	200	0.50	150	50	.70	7.32	6.18	9.84	7.26	7.30	5.10	10.26	7.84	7.86	5.16
4628	2.31, 8	2	0	20	0	10	10	.70	4.86	3.86	6.36	4.32	4.64	3.24	6.22	3.90	4.28	2.86
4629	2.31, 8	2	+	20	0.16	8	12	.70	4.18	3.40	4.60	3.14	3.38	3.38	4.68	2.94	3.20	2.98
4630	2.31, 8	2	-	20	0.16	12	8	.70	6.44	5.10	8.48	5.88	6.10	3.32	7.64	5.38	5.76	3.28
4631	2.31, 8	2	+	20	0.33	7	13	.70	3.82	3.94	4.06	2.56	2.86	3.78	3.62	2.40	2.54	3.08
4632	2.31, 8	2	-	20	0.33	13	7	.70	7.08	6.34	9.70	6.90	7.08	3.70	9.02	6.66	6.96	3.56
4633	2.31, 8	2	+	20	0.50	5	15	.70	2.88	4.40	3.24	2.26	2.48	4.16	3.16	1.96	2.12	3.94
4634	2.31, 8	2	-	20	0.50	15	5	.70	9.00	9.58	12.80	9.58	10.18	4.88	11.86	9.00	9.46	4.96
4635	2.31, 8	2	0	40	0	20	20	.70	4.84	4.30	6.84	4.80	4.92	4.28	6.12	4.30	4.40	3.64
4636	2.31, 8	2	+	40	0.16	17	23	.70	3.80	3.58	5.52	3.82	3.88	4.28	4.84	3.20	3.46	3.68
4637	2.31, 8	2	-	40	0.16	23	17	.70	6.04	5.04	8.22	5.94	6.04	4.54	7.96	5.86	6.00	4.52
4638	2.31, 8	2	+	40	0.33	13	27	.70	2.84	3.72	4.14	2.62	2.72	4.48	3.60	2.40	2.52	3.82

	D	VR	P	N	Δn	n1	n2	ε	F g	B-F g	F t	F-GG t	F-HF t	B-F t	F g x t	F-GG g x t	F-HF g x t	B-F g x t
4639	2.31, 8	2	-	40	0.33	27	13	.70	7.64	5.84	10.54	8.06	8.28	4.54	10.22	7.86	8.04	4.54
4640	2.31, 8	2	+	40	0.50	10	30	.70	2.42	4.32	3.38	2.16	2.24	4.62	2.84	1.92	2.00	4.36
4641	2.31, 8	2	-	40	0.50	30	10	.70	9.04	7.88	12.16	9.50	9.76	5.14	12.10	9.38	9.58	5.04
4642	2.31, 8	2	0	60	0	30	30	.70	4.32	4.24	6.54	4.86	4.92	4.80	6.88	5.28	5.44	4.94
4643	2.31, 8	2	+	60	0.16	25	35	.70	3.86	4.30	5.52	4.00	4.04	4.78	5.38	3.94	3.98	4.78
4644	2.31, 8	2	-	60	0.16	35	25	.70	6.32	5.04	8.64	6.42	6.52	5.02	8.72	6.40	6.48	4.84
4645	2.31, 8	2	+	60	0.33	20	40	.70	3.34	4.66	4.24	3.12	3.14	4.72	4.22	3.02	3.08	4.68
4646	2.31, 8	2	-	60	0.33	40	20	.70	7.76	5.72	10.10	7.78	7.88	5.10	10.86	8.28	8.38	4.86
4647	2.31, 8	2	+	60	0.50	15	45	.70	2.66	4.96	3.40	2.24	2.30	5.24	2.84	2.00	2.02	4.52
4648	2.31, 8	2	-	60	0.50	45	15	.70	10.32	7.28	12.54	10.16	10.28	5.40	13.12	10.38	10.60	5.64
4649	2.31, 8	2	0	80	0	40	40	.70	4.80	4.54	6.98	5.16	5.16	5.02	6.54	4.54	4.58	4.44
4650	2.31, 8	2	+	80	0.16	34	46	.70	3.56	4.26	5.78	4.12	4.16	5.36	5.12	3.48	3.66	4.68
4651	2.31, 8	2	-	80	0.16	46	34	.70	6.48	5.52	8.56	6.24	6.30	4.80	7.94	5.90	5.94	4.74
4652	2.31, 8	2	+	80	0.33	27	53	.70	3.06	4.30	4.42	3.14	3.18	5.36	3.76	2.46	2.50	4.66
4653	2.31, 8	2	-	80	0.33	53	27	.70	7.66	5.44	11.00	8.32	8.38	4.88	10.16	7.86	7.92	5.12
4654	2.31, 8	2	+	80	0.50	20	60	.70	2.52	5.16	3.32	2.34	2.34	5.46	2.96	2.00	2.04	4.58
4655	2.31, 8	2	-	80	0.50	60	20	.70	9.42	6.68	13.58	10.84	10.90	5.84	13.08	10.28	10.46	5.80
4656	2.31, 8	2	0	100	0	50	50	.70	4.94	4.82	6.96	5.24	5.26	5.12	6.24	4.54	4.56	4.58
4657	2.31, 8	2	+	100	0.16	42	58	.70	3.74	4.18	5.92	4.14	4.16	5.28	4.98	3.46	3.56	4.48
4658	2.31, 8	2	-	100	0.16	58	42	.70	6.62	5.54	8.42	6.42	6.42	5.18	8.42	6.36	6.40	4.98
4659	2.31, 8	2	+	100	0.33	33	67	.70	2.72	4.34	4.32	3.08	3.12	5.44	3.52	2.26	2.30	4.60
4660	2.31, 8	2	-	100	0.33	67	33	.70	8.36	5.72	10.70	8.32	8.36	5.06	10.54	8.08	8.16	4.66
4661	2.31, 8	2	+	100	0.50	25	75	.70	2.08	4.82	3.34	2.06	2.08	5.38	2.52	1.56	1.56	4.02
4662	2.31, 8	2	-	100	0.50	75	25	.70	10.08	6.90	13.18	10.38	10.38	5.12	12.42	9.70	9.82	4.72
4663	2.31, 8	2	0	150	0	75	75	.70	5.40	5.36	6.88	4.88	4.92	5.04	6.96	4.80	4.84	5.00
4664	2.31, 8	2	+	150	0.16	63	87	.70	4.40	5.38	5.38	3.84	3.86	5.12	5.54	4.08	4.08	5.32
4665	2.31, 8	2	-	150	0.16	87	63	.70	6.40	5.30	7.88	6.16	6.22	5.26	8.72	6.60	6.64	5.38
4666	2.31, 8	2	+	150	0.33	50	100	.70	2.82	4.92	4.04	2.88	2.90	5.30	4.04	2.96	2.96	5.14
4667	2.31, 8	2	-	150	0.33	100	50	.70	8.02	5.54	10.80	8.04	8.04	5.08	10.30	7.52	7.58	4.82
4668	2.31, 8	2	+	150	0.50	37	113	.70	2.10	5.08	3.08	2.08	2.10	5.12	2.98	1.94	1.94	5.20
4669	2.31, 8	2	-	150	0.50	113	37	.70	10.06	6.46	12.46	9.92	9.94	5.38	12.66	9.98	10.02	5.10
4670	2.31, 8	2	0	200	0	100	100	.70	5.30	5.26	6.70	4.78	4.80	4.98	6.50	4.68	4.72	4.78

	D	VR	P	N	Δn	n1	n2	ε	F g	B-F g	F t	F-GG t	F-HF t	B-F t	F g x t	F-GG g x t	F-HF g x t	B-F g x t
4671	2.31, 8	2	+	200	0.16	84	116	.70	3.92	4.92	5.14	3.76	3.78	4.84	5.46	3.70	3.74	5.24
4672	2.31, 8	2	-	200	0.16	116	84	.70	6.26	5.14	8.26	6.20	6.26	4.92	8.54	6.62	6.62	5.48
4673	2.31, 8	2	+	200	0.33	67	133	.70	2.90	4.86	4.16	2.78	2.82	5.46	4.04	2.76	2.78	5.40
4674	2.31, 8	2	-	200	0.33	133	67	.70	7.96	5.52	10.10	7.70	7.72	4.80	10.22	8.02	8.02	5.06
4675	2.31, 8	2	+	200	0.50	50	150	.70	1.90	5.06	2.96	1.94	2.00	5.20	3.16	2.16	2.16	5.32
4676	2.31, 8	2	-	200	0.50	150	50	.70	10.12	6.42	12.68	10.10	10.14	5.18	12.76	10.12	10.20	5.10
4677	2.31, 8	5	0	20	0	10	10	.70	7.20	6.12	6.38	4.22	4.48	3.00	6.64	4.72	5.00	3.04
4678	2.31, 8	5	+	20	0.16	8	12	.70	4.86	5.32	3.36	2.16	2.38	3.06	3.28	2.02	2.18	2.82
4679	2.31, 8	5	-	20	0.16	12	8	.70	11.02	8.34	11.26	8.14	8.54	3.04	11.04	7.76	8.18	3.12
4680	2.31, 8	5	+	20	0.33	7	13	.70	4.06	4.96	2.26	1.44	1.56	3.46	2.26	1.50	1.64	2.88
4681	2.31, 8	5	-	20	0.33	13	7	.70	13.64	9.60	14.18	10.52	10.94	3.84	13.94	10.38	10.82	3.64
4682	2.31, 8	5	+	20	0.50	5	15	.70	2.26	4.00	1.10	.68	.76	3.76	1.10	.64	.76	3.44
4683	2.31, 8	5	-	20	0.50	15	5	.70	19.20	13.26	21.58	17.52	17.92	5.24	21.30	17.06	17.48	4.98
4684	2.31, 8	5	0	40	0	20	20	.70	6.26	5.60	6.66	4.80	5.00	4.00	6.14	4.50	4.62	3.76
4685	2.31, 8	5	+	40	0.16	17	23	.70	4.22	4.62	4.18	2.66	2.78	4.04	3.68	2.68	2.78	3.54
4686	2.31, 8	5	-	40	0.16	23	17	.70	8.78	6.26	10.14	7.76	7.88	4.38	10.02	7.76	7.92	4.66
4687	2.31, 8	5	+	40	0.33	13	27	.70	2.22	4.04	1.70	1.08	1.08	4.18	1.92	1.06	1.14	3.78
4688	2.31, 8	5	-	40	0.33	27	13	.70	12.50	7.22	15.92	12.78	12.94	4.34	15.78	12.52	12.70	4.48
4689	2.31, 8	5	+	40	0.50	10	30	.70	1.58	4.02	.82	.54	.58	4.40	.98	.62	.64	4.06
4690	2.31, 8	5	-	40	0.50	30	10	.70	17.24	9.38	21.56	17.86	18.04	5.02	21.34	17.32	17.58	4.98
4691	2.31, 8	5	0	60	0	30	30	.70	5.42	4.80	6.46	5.10	5.18	4.72	7.10	5.52	5.58	4.76
4692	2.31, 8	5	+	60	0.16	25	35	.70	3.86	4.98	4.16	2.90	2.92	4.42	4.20	2.80	2.88	4.66
4693	2.31, 8	5	-	60	0.16	35	25	.70	8.90	6.24	10.68	8.06	8.08	4.66	10.60	8.36	8.40	4.68
4694	2.31, 8	5	+	60	0.33	20	40	.70	2.40	4.46	2.24	1.48	1.50	4.58	2.16	1.22	1.26	4.44
4695	2.31, 8	5	-	60	0.33	40	20	.70	12.68	6.62	15.38	12.22	12.32	5.04	15.58	12.58	12.70	5.16
4696	2.31, 8	5	+	60	0.50	15	45	.70	1.30	4.80	.98	.66	.68	4.90	.86	.46	.46	4.08
4697	2.31, 8	5	-	60	0.50	45	15	.70	18.34	8.52	21.42	17.80	18.02	5.16	21.82	18.40	18.78	5.44
4698	2.31, 8	5	0	80	0	40	40	.70	5.56	5.02	6.80	5.10	5.12	4.94	6.32	4.64	4.64	4.24
4699	2.31, 8	5	+	80	0.16	34	46	.70	3.46	4.56	4.46	3.12	3.16	5.32	3.82	2.66	2.70	4.52
4700	2.31, 8	5	-	80	0.16	46	34	.70	8.44	5.50	10.86	8.00	8.06	4.56	10.16	7.48	7.58	4.34
4701	2.31, 8	5	+	80	0.33	27	53	.70	2.32	4.76	2.26	1.56	1.60	5.16	1.94	1.22	1.22	4.02
4702	2.31, 8	5	-	80	0.33	53	27	.70	11.98	6.12	16.30	12.74	12.86	4.56	15.00	12.12	12.24	4.84

	D	VR	P	N	Δn	n1	n2	ε	F g	B-F g	F t	F-GG t	F-HF t	B-F t	F g x t	F-GG g x t	F-HF g x t	B-F g x t
4703	2.31, 8	5	+	80	0.50	20	60	.70	1.26	4.74	1.14	.72	.76	5.26	.68	.28	.30	4.60
4704	2.31, 8	5	-	80	0.50	60	20	.70	17.04	7.36	23.32	19.36	19.44	5.70	22.46	18.82	18.84	5.64
4705	2.31, 8	5	0	100	0	50	50	.70	5.52	5.18	6.48	4.98	4.98	4.54	6.40	4.36	4.38	4.12
4706	2.31, 8	5	+	100	0.16	42	58	.70	3.46	5.08	4.14	2.96	2.98	4.98	3.48	2.28	2.28	4.32
4707	2.31, 8	5	-	100	0.16	58	42	.70	8.58	5.42	10.38	7.86	7.92	4.74	10.04	7.90	8.00	4.86
4708	2.31, 8	5	+	100	0.33	33	67	.70	1.74	3.94	2.20	1.32	1.36	5.40	1.64	1.16	1.16	4.54
4709	2.31, 8	5	-	100	0.33	67	33	.70	12.64	6.22	16.04	12.88	12.96	4.78	15.78	12.66	12.72	4.94
4710	2.31, 8	5	+	100	0.50	25	75	.70	.96	4.10	.90	.44	.44	5.40	.62	.38	.40	4.06
4711	2.31, 8	5	-	100	0.50	75	25	.70	17.42	7.22	21.44	18.44	18.54	5.04	21.62	18.00	18.06	4.88
4712	2.31, 8	5	0	150	0	75	75	.70	5.92	5.72	6.60	4.86	4.90	5.04	7.00	4.96	5.02	4.90
4713	2.31, 8	5	+	150	0.16	63	87	.70	4.04	5.56	3.98	3.00	3.02	4.82	4.12	2.82	2.82	5.12
4714	2.31, 8	5	-	150	0.16	87	63	.70	8.76	5.56	9.94	8.02	8.08	4.88	10.38	7.96	8.06	5.24
4715	2.31, 8	5	+	150	0.33	50	100	.70	1.96	4.80	2.14	1.38	1.38	4.94	2.08	1.34	1.36	5.08
4716	2.31, 8	5	-	150	0.33	100	50	.70	12.50	6.10	15.34	12.56	12.58	4.84	15.06	12.34	12.40	4.92
4717	2.31, 8	5	+	150	0.50	37	113	.70	.94	4.70	.92	.56	.56	5.06	.94	.64	.66	4.70
4718	2.31, 8	5	-	150	0.50	113	37	.70	17.20	6.88	21.52	18.06	18.08	5.16	21.54	17.80	17.94	5.18
4719	2.31, 8	5	0	200	0	100	100	.70	5.70	5.34	6.34	5.08	5.08	4.96	6.52	4.82	4.82	4.96
4720	2.31, 8	5	+	200	0.16	84	116	.70	3.26	5.08	3.86	2.72	2.74	4.80	4.26	2.88	2.90	4.96
4721	2.31, 8	5	-	200	0.16	116	84	.70	8.10	5.00	10.04	7.80	7.84	4.92	10.50	8.26	8.32	5.32
4722	2.31, 8	5	+	200	0.33	67	133	.70	1.96	4.72	2.06	1.36	1.36	4.98	2.02	1.40	1.44	5.38
4723	2.31, 8	5	-	200	0.33	133	67	.70	12.22	5.78	15.54	12.10	12.18	4.78	15.22	12.38	12.44	4.98
4724	2.31, 8	5	+	200	0.50	50	150	.70	.68	4.76	.88	.38	.40	5.16	.94	.46	.46	5.52
4725	2.31, 8	5	-	200	0.50	150	50	.70	17.46	6.54	22.18	18.92	19.02	5.24	21.58	18.26	18.30	5.38
4726	2.31, 8	1	0	20	0	10	10	.80	3.80	2.80	5.64	4.20	4.46	3.28	5.96	4.20	4.54	2.98
4727	2.31, 8	1	0	20	0.16	8	12	.80	4.38	3.68	5.82	4.18	4.48	3.32	5.42	3.84	4.12	2.86
4728	2.31, 8	1	0	20	0.33	7	13	.80	4.54	4.62	5.80	4.30	4.62	3.68	5.72	4.20	4.38	3.40
4729	2.31, 8	1	0	20	0.50	5	15	.80	4.34	5.74	6.74	5.04	5.44	4.02	5.98	4.46	4.72	4.08
4730	2.31, 8	1	0	40	0	20	20	.80	4.78	4.06	5.60	4.30	4.50	3.84	5.68	4.36	4.54	3.76
4731	2.31, 8	1	0	40	0.16	17	23	.80	4.80	4.52	5.74	4.52	4.68	4.02	5.70	4.60	4.84	4.12
4732	2.31, 8	1	0	40	0.33	13	27	.80	4.42	4.66	6.08	4.72	4.96	4.20	6.42	4.84	5.12	4.42
4733	2.31, 8	1	0	40	0.50	10	30	.80	4.26	5.90	5.78	4.66	4.78	4.38	6.22	4.92	5.12	5.36
4734	2.31, 8	1	0	60	0	30	30	.80	4.74	4.30	5.98	4.68	4.82	4.34	5.54	4.28	4.38	4.18

	D	VR	P	N	Δn	n1	n2	ε	F g	B-F g	F t	F-GG t	F-HF t	B-F t	F g x t	F-GG g x t	F-HF g x t	B-F g x t
4735	2.31, 8	1	0	60	0.16	25	35	.80	4.84	4.58	5.86	4.98	5.10	4.68	5.52	4.20	4.28	4.08
4736	2.31, 8	1	0	60	0.33	20	40	.80	4.60	5.00	6.16	4.76	4.88	4.76	5.86	4.44	4.54	4.40
4737	2.31, 8	1	0	60	0.50	15	45	.80	4.86	6.18	5.96	4.82	4.86	4.68	6.04	4.88	4.98	5.02
4738	2.31, 8	1	0	80	0	40	40	.80	4.50	4.48	6.64	5.50	5.54	5.22	5.36	4.38	4.44	4.30
4739	2.31, 8	1	0	80	0.16	34	46	.80	4.44	4.70	6.08	4.98	5.16	5.02	5.68	4.92	5.02	4.86
4740	2.31, 8	1	0	80	0.33	27	53	.80	5.04	5.24	5.86	4.86	4.90	5.14	5.50	4.36	4.44	4.44
4741	2.31, 8	1	0	80	0.50	20	60	.80	4.96	6.18	6.12	5.10	5.18	4.96	6.14	4.88	4.92	4.58
4742	2.31, 8	1	0	100	0	50	50	.80	5.14	4.86	6.10	4.96	5.02	5.18	5.80	4.62	4.66	4.68
4743	2.31, 8	1	0	100	0.16	42	58	.80	5.00	4.68	6.10	4.76	4.90	4.88	5.56	4.54	4.54	4.78
4744	2.31, 8	1	0	100	0.33	33	67	.80	4.66	5.22	5.94	4.82	4.84	4.86	5.78	4.72	4.82	4.98
4745	2.31, 8	1	0	100	0.50	25	75	.80	4.42	5.78	5.68	4.72	4.80	4.80	5.10	4.22	4.26	4.48
4746	2.31, 8	1	0	150	0	75	75	.80	5.46	5.46	6.46	5.28	5.34	5.42	6.58	5.58	5.64	5.68
4747	2.31, 8	1	0	150	0.16	63	87	.80	5.68	5.62	6.44	5.24	5.30	5.42	5.94	4.80	4.80	4.78
4748	2.31, 8	1	0	150	0.33	50	100	.80	4.96	5.46	6.22	5.16	5.16	5.14	5.86	4.62	4.66	4.78
4749	2.31, 8	1	0	150	0.50	37	113	.80	5.06	6.06	6.04	4.94	5.04	5.22	5.62	4.76	4.80	5.06
4750	2.31, 8	1	0	200	0	100	100	.80	5.06	5.10	6.52	5.28	5.28	5.48	6.34	5.00	5.04	5.42
4751	2.31, 8	1	0	200	0.16	84	116	.80	5.36	5.22	6.48	5.58	5.60	5.60	6.48	5.52	5.58	5.54
4752	2.31, 8	1	0	200	0.33	67	133	.80	4.66	5.06	6.48	5.38	5.40	5.50	6.10	5.06	5.08	5.02
4753	2.31, 8	1	0	200	0.50	50	150	.80	4.86	5.42	6.42	5.24	5.34	5.30	5.70	4.54	4.60	4.84
4754	2.31, 8	1.5	0	20	0	10	10	.80	3.88	2.90	5.78	4.34	4.62	3.48	5.98	4.20	4.54	3.16
4755	2.31, 8	1.5	+	20	0.16	8	12	.80	4.12	3.30	4.88	3.40	3.70	3.24	4.38	3.28	3.50	3.02
4756	2.31, 8	1.5	-	20	0.16	12	8	.80	5.16	3.82	6.86	5.20	5.36	3.62	6.82	4.98	5.24	3.64
4757	2.31, 8	1.5	+	20	0.33	7	13	.80	3.82	3.64	4.40	3.12	3.42	3.56	4.28	3.06	3.28	3.32
4758	2.31, 8	1.5	-	20	0.33	13	7	.80	5.22	4.80	6.92	5.22	5.58	3.62	7.48	5.40	5.88	3.34
4759	2.31, 8	1.5	+	20	0.50	5	15	.80	3.26	4.56	4.42	3.06	3.22	3.90	3.92	2.78	3.00	3.86
4760	2.31, 8	1.5	-	20	0.50	15	5	.80	6.88	8.78	8.86	6.82	7.28	4.42	9.50	7.22	7.66	4.52
4761	2.31, 8	1.5	0	40	0	20	20	.80	4.90	4.06	5.42	4.18	4.36	3.70	5.40	4.06	4.16	3.60
4762	2.31, 8	1.5	+	40	0.16	17	23	.80	4.50	4.30	4.98	3.60	3.72	4.10	4.90	3.78	4.04	3.88
4763	2.31, 8	1.5	-	40	0.16	23	17	.80	5.34	4.66	6.20	4.74	5.02	3.96	6.44	5.00	5.14	3.98
4764	2.31, 8	1.5	+	40	0.33	13	27	.80	3.04	3.98	4.24	3.30	3.42	3.88	4.46	3.14	3.24	4.46
4765	2.31, 8	1.5	-	40	0.33	27	13	.80	6.44	5.96	8.12	6.28	6.52	4.46	7.46	5.76	5.92	4.10
4766	2.31, 8	1.5	+	40	0.50	10	30	.80	2.64	4.78	3.78	2.78	2.86	4.34	3.70	3.04	3.16	5.22

	D	VR	P	N	Δn	n1	n2	ε	F g	B-F g	F t	F-GG t	F-HF t	B-F t	F g x t	F-GG g x t	F-HF g x t	B-F g x t
4767	2.31, 8	1.5	-	40	0.50	30	10	.80	7.14	7.02	9.22	7.24	7.38	4.70	8.76	6.94	7.20	4.46
4768	2.31, 8	1.5	0	60	0	30	30	.80	4.84	4.48	6.12	4.78	4.96	4.50	5.50	4.30	4.42	4.22
4769	2.31, 8	1.5	+	60	0.16	25	35	.80	4.42	4.26	4.90	4.06	4.12	4.46	4.70	3.60	3.62	4.30
4770	2.31, 8	1.5	-	60	0.16	35	25	.80	5.54	4.72	7.22	5.68	5.84	4.96	6.76	5.56	5.68	4.66
4771	2.31, 8	1.5	+	60	0.33	20	40	.80	3.26	4.48	4.42	3.58	3.68	4.62	4.08	3.24	3.30	4.24
4772	2.31, 8	1.5	-	60	0.33	40	20	.80	6.24	5.50	8.12	6.58	6.76	4.38	7.40	5.88	5.96	4.32
4773	2.31, 8	1.5	+	60	0.50	15	45	.80	3.28	5.10	3.74	2.74	2.80	4.66	2.80	2.84	2.90	5.06
4774	2.31, 8	1.5	-	60	0.50	45	15	.80	7.14	6.72	8.98	7.36	7.52	4.90	8.86	7.30	7.42	4.80
4775	2.31, 8	1.5	0	80	0	40	40	.80	4.82	4.54	6.60	5.48	5.62	5.22	5.54	4.26	4.34	4.24
4776	2.31, 8	1.5	+	80	0.16	34	46	.80	3.84	4.36	5.54	4.56	4.58	5.42	5.26	4.24	4.32	4.96
4777	2.31, 8	1.5	-	80	0.16	46	34	.80	5.38	4.74	7.62	6.30	6.38	5.40	6.38	5.16	5.32	4.44
4778	2.31, 8	1.5	+	80	0.33	27	53	.80	3.92	4.88	4.44	3.34	3.46	5.22	4.08	3.14	3.20	4.38
4779	2.31, 8	1.5	-	80	0.33	53	27	.80	6.70	5.40	8.68	7.40	7.42	5.20	8.20	6.62	6.76	5.68
4780	2.31, 8	1.5	+	80	0.50	20	60	.80	2.96	5.72	3.94	2.86	2.94	5.28	3.50	2.76	2.78	4.90
4781	2.31, 8	1.5	-	80	0.50	60	20	.80	6.98	6.28	10.16	8.42	8.52	5.06	9.18	7.56	7.74	4.96
4782	2.31, 8	1.5	0	100	0	50	50	.80	4.78	4.52	6.14	4.98	5.04	5.10	5.80	4.58	4.70	4.82
4783	2.31, 8	1.5	+	100	0.16	42	58	.80	4.38	4.62	5.22	4.14	4.20	4.90	4.66	3.76	3.78	4.62
4784	2.31, 8	1.5	-	100	0.16	58	42	.80	5.68	5.10	6.88	5.72	5.78	5.02	6.86	5.78	5.84	4.86
4785	2.31, 8	1.5	+	100	0.33	33	67	.80	3.52	4.82	4.32	3.22	3.30	4.94	4.06	3.24	3.26	4.84
4786	2.31, 8	1.5	-	100	0.33	67	33	.80	7.16	6.10	8.36	6.80	6.86	5.10	8.44	6.90	7.00	4.94
4787	2.31, 8	1.5	+	100	0.50	25	75	.80	2.84	5.14	3.40	2.72	2.74	4.74	3.10	2.36	2.38	4.34
4788	2.31, 8	1.5	-	100	0.50	75	25	.80	8.02	6.30	8.94	7.40	7.52	4.52	9.30	7.94	8.00	4.96
4789	2.31, 8	1.5	0	150	0	75	75	.80	5.32	5.20	6.54	5.20	5.22	5.40	6.70	5.60	5.70	5.70
4790	2.31, 8	1.5	+	150	0.16	63	87	.80	4.72	5.62	5.40	4.42	4.46	5.40	5.00	3.98	4.00	4.86
4791	2.31, 8	1.5	-	150	0.16	87	63	.80	6.02	5.12	7.40	6.14	6.18	5.16	7.00	5.98	6.00	5.12
4792	2.31, 8	1.5	+	150	0.33	50	100	.80	3.68	5.08	4.72	3.62	3.68	5.48	4.12	3.24	3.26	5.06
4793	2.31, 8	1.5	-	150	0.33	100	50	.80	6.60	5.32	8.52	7.30	7.36	5.36	8.20	6.82	6.86	5.34
4794	2.31, 8	1.5	+	150	0.50	37	113	.80	3.08	5.58	3.54	2.82	2.82	5.28	3.36	2.46	2.48	4.96
4795	2.31, 8	1.5	-	150	0.50	113	37	.80	8.04	6.24	9.70	8.16	8.16	5.24	9.52	7.92	7.98	5.14
4796	2.31, 8	1.5	0	200	0	100	100	.80	5.30	5.14	6.26	5.20	5.24	5.32	6.12	5.16	5.16	5.26
4797	2.31, 8	1.5	+	200	0.16	84	116	.80	4.40	5.22	5.64	4.70	4.74	5.52	5.68	4.62	4.64	5.60
4798	2.31, 8	1.5	-	200	0.16	116	84	.80	5.80	5.30	7.60	6.08	6.14	4.98	7.12	6.02	6.08	5.16

	D	VR	P	N	Δn	n1	n2	ε	F g	B-F g	F t	F-GG t	F-HF t	B-F t	F g x t	F-GG g x t	F-HF g x t	B-F g x t
4799	2.31, 8	1.5	+	200	0.33	67	133	.80	3.42	4.88	4.68	3.88	3.90	5.48	4.28	3.44	3.48	4.94
4800	2.31, 8	1.5	-	200	0.33	133	67	.80	6.54	5.38	8.78	7.36	7.38	5.36	8.32	7.06	7.10	5.32
4801	2.31, 8	1.5	+	200	0.50	50	150	.80	2.82	5.08	3.96	3.18	3.18	5.40	3.48	2.54	2.62	5.04
4802	2.31, 8	1.5	-	200	0.50	150	50	.80	7.58	5.74	9.94	8.54	8.60	5.76	9.46	7.94	7.98	5.02
4803	2.31, 8	2	0	20	0	10	10	.80	4.50	3.54	6.08	4.34	4.80	3.52	6.00	4.16	4.58	3.20
4804	2.31, 8	2	+	20	0.16	8	12	.80	4.14	3.52	4.30	2.98	3.18	3.32	3.98	2.94	3.08	2.96
4805	2.31, 8	2	-	20	0.16	12	8	.80	6.52	4.98	7.54	5.52	5.94	3.68	7.64	5.62	5.90	3.64
4806	2.31, 8	2	+	20	0.33	7	13	.80	3.52	3.56	3.60	2.50	2.70	3.44	3.52	2.62	2.82	3.18
4807	2.31, 8	2	-	20	0.33	13	7	.80	6.84	5.82	8.38	6.16	6.66	3.56	8.78	6.58	7.06	3.66
4808	2.31, 8	2	+	20	0.50	5	15	.80	2.80	3.92	3.24	2.02	2.28	3.88	2.66	1.86	2.06	3.78
4809	2.31, 8	2	-	20	0.50	15	5	.80	9.64	10.14	11.70	9.06	9.64	4.48	12.00	9.48	9.90	4.58
4810	2.31, 8	2	0	40	0	20	20	.80	5.18	4.52	5.34	4.14	4.32	3.64	5.36	4.10	4.24	3.58
4811	2.31, 8	2	+	40	0.16	17	23	.80	4.06	4.16	4.38	3.24	3.44	3.90	4.62	3.32	3.48	3.92
4812	2.31, 8	2	-	40	0.16	23	17	.80	6.10	5.20	6.92	5.20	5.46	4.00	6.98	5.52	5.74	3.84
4813	2.31, 8	2	+	40	0.33	13	27	.80	2.60	3.74	3.26	2.24	2.32	4.00	3.24	2.56	2.62	4.36
4814	2.31, 8	2	-	40	0.33	27	13	.80	7.96	6.38	9.76	7.84	8.06	4.46	9.06	7.16	7.34	4.28
4815	2.31, 8	2	+	40	0.50	10	30	.80	2.20	4.32	2.64	1.78	1.90	4.24	2.76	1.92	1.98	5.16
4816	2.31, 8	2	-	40	0.50	30	10	.80	9.36	7.92	12.04	9.80	10.08	4.76	11.06	9.38	9.64	4.42
4817	2.31, 8	2	0	60	0	30	30	.80	4.98	4.58	6.14	4.90	4.98	4.46	5.72	4.34	4.54	4.24
4818	2.31, 8	2	+	60	0.16	25	35	.80	4.02	4.46	4.64	3.48	3.58	4.54	4.12	3.16	3.28	4.34
4819	2.31, 8	2	-	60	0.16	35	25	.80	6.30	5.04	7.88	6.26	6.40	4.92	7.24	6.08	6.24	4.80
4820	2.31, 8	2	+	60	0.33	20	40	.80	3.00	4.26	3.58	2.80	2.88	4.64	3.16	2.34	2.44	4.28
4821	2.31, 8	2	-	60	0.33	40	20	.80	7.90	6.00	9.58	7.94	8.02	4.34	9.18	7.48	7.62	4.16
4822	2.31, 8	2	+	60	0.50	15	45	.80	2.46	4.84	2.60	1.76	1.84	4.60	2.58	1.96	2.04	4.96
4823	2.31, 8	2	-	60	0.50	45	15	.80	9.38	7.32	11.60	9.54	9.66	5.02	11.54	9.50	9.66	4.68
4824	2.31, 8	2	0	80	0	40	40	.80	4.78	4.52	6.70	5.52	5.56	5.30	5.60	4.46	4.48	4.14
4825	2.31, 8	2	+	80	0.16	34	46	.80	3.48	4.36	5.36	4.22	4.26	5.62	4.76	3.74	3.78	5.02
4826	2.31, 8	2	-	80	0.16	46	34	.80	6.04	4.92	8.34	6.82	6.92	5.24	7.20	5.76	5.82	4.44
4827	2.31, 8	2	+	80	0.33	27	53	.80	3.12	4.66	3.60	2.80	2.82	5.22	3.24	2.50	2.52	4.42
4828	2.31, 8	2	-	80	0.33	53	27	.80	8.04	5.82	10.34	8.58	8.74	5.28	10.04	8.16	8.28	4.38
4829	2.31, 8	2	+	80	0.50	20	60	.80	2.38	5.14	2.38	1.82	1.86	5.34	2.46	1.84	1.92	4.90
4830	2.31, 8	2	-	80	0.50	60	20	.80	9.36	6.74	12.74	11.14	11.24	5.04	11.74	10.02	10.12	4.84

	D	VR	P	N	Δn	n1	n2	ε	F g	B-F g	F t	F-GG t	F-HF t	B-F t	F g x t	F-GG g x t	F-HF g x t	B-F g x t
4831	2.31, 8	2	0	100	0	50	50	.80	4.92	4.60	6.26	5.08	5.12	4.98	5.80	4.68	4.72	4.66
4832	2.31, 8	2	+	100	0.16	42	58	.80	4.02	4.94	4.82	3.76	3.80	4.80	4.20	3.52	3.58	4.58
4833	2.31, 8	2	-	100	0.16	58	42	.80	6.44	5.32	7.62	6.40	6.50	5.00	7.36	6.10	6.20	4.86
4834	2.31, 8	2	+	100	0.33	33	67	.80	2.72	4.66	3.22	2.30	2.38	4.98	2.94	2.20	2.20	4.82
4835	2.31, 8	2	-	100	0.33	67	33	.80	8.42	6.06	10.32	8.56	8.62	4.64	9.98	8.50	8.58	4.62
4836	2.31, 8	2	+	100	0.50	25	75	.80	2.08	4.86	2.36	1.70	1.72	5.00	1.78	1.36	1.40	4.48
4837	2.31, 8	2	-	100	0.50	75	25	.80	10.22	6.68	11.48	9.80	9.86	4.30	12.26	10.50	10.54	4.98
4838	2.31, 8	2	0	150	0	75	75	.80	5.16	4.82	6.38	5.18	5.18	5.16	6.76	5.42	5.54	5.42
4839	2.31, 8	2	+	150	0.16	63	87	.80	4.28	5.56	5.04	4.02	4.10	5.38	4.42	3.68	3.72	4.76
4840	2.31, 8	2	-	150	0.16	87	63	.80	6.56	5.18	8.06	6.70	6.74	5.08	7.68	6.30	6.34	5.16
4841	2.31, 8	2	+	150	0.33	50	100	.80	3.08	4.90	3.76	2.78	2.80	5.28	3.18	2.46	2.50	5.14
4842	2.31, 8	2	-	150	0.33	100	50	.80	7.86	5.48	10.44	8.72	8.80	5.48	10.04	8.42	8.50	5.30
4843	2.31, 8	2	+	150	0.50	37	113	.80	2.04	5.42	2.40	1.82	1.86	5.20	2.04	1.54	1.54	4.80
4844	2.31, 8	2	-	150	0.50	113	37	.80	10.64	6.28	12.60	10.56	10.66	4.88	12.44	10.60	10.64	5.10
4845	2.31, 8	2	0	200	0	100	100	.80	5.36	5.18	6.20	5.22	5.26	5.06	6.30	5.06	5.08	5.28
4846	2.31, 8	2	+	200	0.16	84	116	.80	3.80	5.02	5.02	4.04	4.06	5.28	5.12	4.12	4.12	5.48
4847	2.31, 8	2	-	200	0.16	116	84	.80	6.56	5.38	8.30	6.86	6.96	5.02	7.88	6.72	6.74	5.02
4848	2.31, 8	2	+	200	0.33	67	133	.80	2.86	4.82	3.84	3.00	3.02	5.40	3.44	2.62	2.66	5.30
4849	2.31, 8	2	-	200	0.33	133	67	.80	7.94	5.60	10.56	9.06	9.08	5.64	10.52	8.48	8.50	5.20
4850	2.31, 8	2	+	200	0.50	50	150	.80	2.00	5.08	2.66	2.00	2.04	5.54	2.10	1.56	1.56	5.00
4851	2.31, 8	2	-	200	0.50	150	50	.80	9.78	5.80	12.94	11.12	11.18	5.74	12.42	10.68	10.78	5.20
4852	2.31, 8	5	0	20	0	10	10	.80	6.56	5.88	6.14	4.52	4.78	3.24	5.96	4.16	4.60	2.80
4853	2.31, 8	5	+	20	0.16	8	12	.80	4.82	5.20	3.00	1.92	2.10	3.24	3.00	2.02	2.18	2.84
4854	2.31, 8	5	-	20	0.16	12	8	.80	11.12	7.68	10.14	7.78	8.12	3.20	10.12	7.76	8.20	3.16
4855	2.31, 8	5	+	20	0.33	7	13	.80	3.78	4.80	2.02	1.34	1.46	3.22	1.96	1.44	1.56	2.84
4856	2.31, 8	5	-	20	0.33	13	7	.80	13.14	8.76	13.34	9.80	10.26	3.66	13.56	10.30	10.84	3.40
4857	2.31, 8	5	+	20	0.50	5	15	.80	2.12	3.98	.74	.48	.52	3.28	.84	.56	.66	3.22
4858	2.31, 8	5	-	20	0.50	15	5	.80	19.50	13.26	20.90	16.80	17.48	4.54	21.06	17.22	17.92	4.48
4859	2.31, 8	5	0	40	0	20	20	.80	6.06	5.24	5.72	4.40	4.52	3.64	5.68	4.52	4.64	3.80
4860	2.31, 8	5	+	40	0.16	17	23	.80	4.26	4.68	3.04	2.44	2.54	3.40	3.56	2.46	2.56	3.70
4861	2.31, 8	5	-	40	0.16	23	17	.80	9.08	6.42	9.08	7.02	7.32	3.94	9.20	7.22	7.52	3.66
4862	2.31, 8	5	+	40	0.33	13	27	.80	2.16	4.04	1.36	.88	1.00	3.52	1.46	1.06	1.06	3.76

	D	VR	P	N	Δn	n1	n2	ε	F g	B-F g	F t	F-GG t	F-HF t	B-F t	F g x t	F-GG g x t	F-HF g x t	B-F g x t
4863	2.31, 8	5	-	40	0.33	27	13	.80	13.48	7.68	16.28	13.40	13.80	4.18	14.44	11.92	12.22	3.96
4864	2.31, 8	5	+	40	0.50	10	30	.80	1.58	3.76	.54	.38	.38	3.92	.82	.56	.58	4.32
4865	2.31, 8	5	-	40	0.50	30	10	.80	18.06	9.58	21.92	19.16	19.40	4.60	21.28	17.84	18.18	4.26
4866	2.31, 8	5	0	60	0	30	30	.80	6.02	5.64	6.24	5.06	5.24	4.64	5.76	4.54	4.64	4.22
4867	2.31, 8	5	+	60	0.16	25	35	.80	3.84	4.64	3.40	2.52	2.58	4.28	3.12	2.38	2.38	4.02
4868	2.31, 8	5	-	60	0.16	35	25	.80	8.78	5.56	10.08	8.12	8.26	4.36	10.06	8.12	8.32	4.60
4869	2.31, 8	5	+	60	0.33	20	40	.80	2.18	4.44	1.84	1.36	1.44	4.28	1.62	1.02	1.08	4.04
4870	2.31, 8	5	-	60	0.33	40	20	.80	12.18	6.74	14.74	12.58	12.74	4.22	14.48	12.24	12.32	4.14
4871	2.31, 8	5	+	60	0.50	15	45	.80	1.22	4.56	.62	.42	.42	4.64	.70	.60	.60	4.78
4872	2.31, 8	5	-	60	0.50	45	15	.80	18.12	8.26	21.50	18.06	18.28	4.56	21.18	18.38	18.58	4.50
4873	2.31, 8	5	0	80	0	40	40	.80	5.44	4.96	6.52	5.38	5.46	5.08	5.86	4.48	4.54	4.10
4874	2.31, 8	5	+	80	0.16	34	46	.80	3.68	4.56	4.26	3.24	3.26	5.40	3.66	2.74	2.80	4.40
4875	2.31, 8	5	-	80	0.16	46	34	.80	8.50	5.86	10.00	8.34	8.40	4.86	9.34	7.36	7.50	4.36
4876	2.31, 8	5	+	80	0.33	27	53	.80	2.16	4.56	1.88	1.22	1.24	5.38	1.64	1.14	1.22	4.42
4877	2.31, 8	5	-	80	0.33	53	27	.80	12.14	6.38	15.08	12.98	13.12	5.02	15.66	13.38	13.50	4.44
4878	2.31, 8	5	+	80	0.50	20	60	.80	1.18	4.64	.68	.50	.50	5.58	.54	.34	.38	4.62
4879	2.31, 8	5	-	80	0.50	60	20	.80	17.52	7.22	22.50	19.98	20.10	4.58	21.38	18.70	18.90	4.62
4880	2.31, 8	5	0	100	0	50	50	.80	5.46	5.00	6.42	4.90	4.94	4.62	5.74	4.70	4.76	4.68
4881	2.31, 8	5	+	100	0.16	42	58	.80	3.18	4.72	3.68	2.74	2.78	5.08	3.36	2.54	2.60	4.44
4882	2.31, 8	5	-	100	0.16	58	42	.80	8.46	5.66	9.88	8.22	8.26	4.66	9.26	7.82	7.94	4.74
4883	2.31, 8	5	+	100	0.33	33	67	.80	1.74	4.30	1.56	1.14	1.20	4.96	1.48	1.16	1.18	4.50
4884	2.31, 8	5	-	100	0.33	67	33	.80	13.10	6.40	15.30	13.30	13.48	4.58	15.30	13.20	13.26	4.04
4885	2.31, 8	5	+	100	0.50	25	75	.80	1.06	4.12	.68	.44	.44	5.02	.54	.34	.36	4.16
4886	2.31, 8	5	-	100	0.50	75	25	.80	18.16	7.06	21.12	18.46	18.56	4.34	20.52	18.50	18.60	4.58
4887	2.31, 8	5	0	150	0	75	75	.80	5.38	4.86	6.42	5.22	5.28	5.00	6.80	5.26	5.38	5.06
4888	2.31, 8	5	+	150	0.16	63	87	.80	3.64	5.28	3.86	2.90	2.94	5.36	3.70	2.72	2.76	4.78
4889	2.31, 8	5	-	150	0.16	87	63	.80	8.20	5.30	10.14	8.60	8.64	4.92	9.84	8.14	8.18	4.88
4890	2.31, 8	5	+	150	0.33	50	100	.80	1.72	4.68	1.88	1.36	1.36	5.32	1.54	1.04	1.06	4.96
4891	2.31, 8	5	-	150	0.33	100	50	.80	12.22	5.62	15.84	13.54	13.60	5.48	14.90	12.86	13.00	5.26
4892	2.31, 8	5	+	150	0.50	37	113	.80	.74	4.44	.76	.56	.56	5.40	.44	.34	.34	4.66
4893	2.31, 8	5	-	150	0.50	113	37	.80	17.88	6.56	22.78	20.00	20.04	4.86	22.28	19.76	19.78	4.80
4894	2.31, 8	5	0	200	0	100	100	.80	5.56	5.18	6.18	5.02	5.12	5.06	6.32	5.22	5.24	5.24

	D	VR	P	N	Δn	n1	n2	ε	F g	B-F g	F t	F-GG t	F-HF t	B-F t	F g x t	F-GG g x t	F-HF g x t	B-F g x t
4895	2.31, 8	5	+	200	0.16	84	116	.80	2.84	4.88	3.62	2.94	2.98	5.20	3.66	2.90	2.92	5.12
4896	2.31, 8	5	-	200	0.16	116	84	.80	8.54	5.38	10.34	8.76	8.78	5.32	10.46	8.82	8.88	5.02
4897	2.31, 8	5	+	200	0.33	67	133	.80	1.60	4.78	1.82	1.44	1.44	5.24	1.72	1.38	1.38	5.16
4898	2.31, 8	5	-	200	0.33	133	67	.80	12.30	5.66	15.76	13.68	13.70	5.24	15.42	13.58	13.60	5.46
4899	2.31, 8	5	+	200	0.50	50	150	.80	.76	4.60	.68	.48	.48	5.44	.44	.28	.28	4.72
4900	2.31, 8	5	-	200	0.50	150	50	.80	17.36	6.32	22.30	20.00	20.06	5.48	22.48	20.16	20.18	4.96
4901	2.31, 8	1	0	20	0	10	10	.90	3.72	2.84	6.00	4.66	4.96	3.66	4.74	3.54	3.98	2.52
4902	2.31, 8	1	0	20	0.16	8	12	.90	4.04	3.44	6.00	4.56	5.04	3.72	4.82	3.62	3.86	2.66
4903	2.31, 8	1	0	20	0.33	7	13	.90	4.52	4.38	5.98	4.66	5.00	4.06	4.74	3.86	4.18	2.88
4904	2.31, 8	1	0	20	0.50	5	15	.90	4.36	6.24	5.96	4.74	5.18	4.94	5.22	4.16	4.64	4.14
4905	2.31, 8	1	0	40	0	20	20	.90	5.04	4.18	5.64	4.48	4.74	3.88	5.28	4.50	4.64	3.68
4906	2.31, 8	1	0	40	0.16	17	23	.90	4.44	4.04	5.72	4.50	4.72	4.10	4.82	3.88	4.18	3.66
4907	2.31, 8	1	0	40	0.33	13	27	.90	4.64	4.32	5.86	4.64	4.88	4.54	5.08	4.10	4.28	3.76
4908	2.31, 8	1	0	40	0.50	10	30	.90	3.86	5.90	5.82	4.88	5.04	5.16	5.48	4.50	4.74	4.36
4909	2.31, 8	1	0	60	0	30	30	.90	4.52	4.20	5.56	4.88	5.00	4.68	5.06	4.28	4.36	4.20
4910	2.31, 8	1	0	60	0.16	25	35	.90	4.64	4.32	5.80	5.04	5.10	4.68	5.02	4.28	4.44	3.92
4911	2.31, 8	1	0	60	0.33	20	40	.90	4.68	5.08	5.90	5.04	5.28	5.04	5.26	4.42	4.52	4.12
4912	2.31, 8	1	0	60	0.50	15	45	.90	4.82	6.14	5.98	5.38	5.48	5.12	5.20	4.68	4.80	5.00
4913	2.31, 8	1	0	80	0	40	40	.90	4.34	4.34	6.78	5.70	5.76	5.38	5.14	4.58	4.70	4.62
4914	2.31, 8	1	0	80	0.16	34	46	.90	4.72	4.74	6.40	5.64	5.74	5.48	5.46	4.70	4.84	4.70
4915	2.31, 8	1	0	80	0.33	27	53	.90	4.56	4.66	6.22	5.54	5.58	5.34	5.28	4.56	4.66	4.14
4916	2.31, 8	1	0	80	0.50	20	60	.90	4.96	6.46	6.16	5.32	5.36	5.54	5.54	5.08	5.28	4.64
4917	2.31, 8	1	0	100	0	50	50	.90	4.76	4.78	6.40	5.48	5.56	5.36	4.74	4.22	4.32	4.16
4918	2.31, 8	1	0	100	0.16	42	58	.90	4.80	4.60	6.24	5.50	5.56	5.36	5.26	4.68	4.76	4.56
4919	2.31, 8	1	0	100	0.33	33	67	.90	4.44	4.98	5.70	5.18	5.26	5.38	5.42	4.86	5.00	4.96
4920	2.31, 8	1	0	100	0.50	25	75	.90	4.36	5.80	5.98	5.22	5.30	5.44	4.86	4.38	4.42	4.60
4921	2.31, 8	1	0	150	0	75	75	.90	5.38	5.32	5.78	5.18	5.30	5.20	6.10	5.38	5.48	5.48
4922	2.31, 8	1	0	150	0.16	63	87	.90	5.38	5.42	5.86	5.16	5.24	5.30	5.48	4.94	4.98	5.16
4923	2.31, 8	1	0	150	0.33	50	100	.90	4.74	5.10	5.88	5.16	5.18	5.12	5.24	4.64	4.66	5.10
4924	2.31, 8	1	0	150	0.50	37	113	.90	4.58	5.86	5.92	5.18	5.20	5.70	5.20	4.88	4.94	5.36
4925	2.31, 8	1	0	200	0	100	100	.90	4.94	5.04	5.68	5.12	5.16	5.26	5.86	5.26	5.30	5.38
4926	2.31, 8	1	0	200	0.16	84	116	.90	5.18	5.22	5.98	5.54	5.54	5.42	5.82	5.20	5.20	5.32

	D	VR	P	N	Δn	n1	n2	ε	F g	B-F g	F t	F-GG t	F-HF t	B-F t	F g x t	F-GG g x t	F-HF g x t	B-F g x t
4927	2.31, 8	1	0	200	0.33	67	133	.90	4.86	5.22	6.04	5.22	5.22	5.38	5.82	5.26	5.28	5.22
4928	2.31, 8	1	0	200	0.50	50	150	.90	4.54	5.56	5.88	5.08	5.12	5.70	5.04	4.60	4.62	4.92
4929	2.31, 8	1.5	0	20	0	10	10	.90	4.00	2.98	6.20	4.78	5.04	3.90	4.90	3.78	4.12	2.68
4930	2.31, 8	1.5	+	20	0.16	8	12	.90	3.74	2.84	5.10	3.80	4.14	3.84	3.94	3.02	3.22	2.60
4931	2.31, 8	1.5	-	20	0.16	12	8	.90	5.30	4.24	7.16	5.48	5.78	3.90	5.86	4.68	5.06	2.90
4932	2.31, 8	1.5	+	20	0.33	7	13	.90	3.82	4.04	4.42	3.44	3.64	3.82	3.78	2.88	3.16	2.82
4933	2.31, 8	1.5	-	20	0.33	13	7	.90	5.40	5.14	7.92	5.98	6.52	4.30	6.74	5.26	5.60	3.46
4934	2.31, 8	1.5	+	20	0.50	5	15	.90	3.32	5.02	3.92	3.04	3.10	4.42	3.18	2.50	2.82	3.74
4935	2.31, 8	1.5	-	20	0.50	15	5	.90	6.82	8.24	9.08	7.28	7.68	5.94	8.64	7.18	7.60	4.60
4936	2.31, 8	1.5	0	40	0	20	20	.90	5.10	4.14	5.48	4.50	4.58	4.00	5.22	4.48	4.78	3.82
4937	2.31, 8	1.5	+	40	0.16	17	23	.90	3.84	3.44	4.68	3.90	4.04	3.92	3.98	3.26	3.40	3.44
4938	2.31, 8	1.5	-	40	0.16	23	17	.90	5.42	4.60	6.48	5.52	5.78	4.24	5.84	5.14	5.30	4.08
4939	2.31, 8	1.5	+	40	0.33	13	27	.90	3.32	4.00	3.84	3.22	3.34	4.34	3.50	2.92	3.08	3.62
4940	2.31, 8	1.5	-	40	0.33	27	13	.90	6.06	5.54	8.14	6.90	7.06	4.72	6.86	6.16	6.32	4.02
4941	2.31, 8	1.5	+	40	0.50	10	30	.90	2.94	4.78	3.46	2.96	3.04	5.00	3.10	2.58	2.72	3.98
4942	2.31, 8	1.5	-	40	0.50	30	10	.90	7.10	7.44	9.38	8.14	8.46	5.38	8.04	6.92	7.20	4.88
4943	2.31, 8	1.5	0	60	0	30	30	.90	4.38	4.12	5.62	4.82	4.94	4.72	5.14	4.42	4.60	4.32
4944	2.31, 8	1.5	+	60	0.16	25	35	.90	3.88	4.28	4.78	4.18	4.20	4.64	4.20	3.64	3.64	4.10
4945	2.31, 8	1.5	-	60	0.16	35	25	.90	5.42	4.84	7.06	5.88	6.08	4.70	6.50	5.66	5.88	4.86
4946	2.31, 8	1.5	+	60	0.33	20	40	.90	3.82	4.94	4.38	3.58	3.78	4.72	3.66	3.12	3.20	3.94
4947	2.31, 8	1.5	-	60	0.33	40	20	.90	6.56	5.52	8.20	7.18	7.42	4.70	7.56	6.62	6.86	4.70
4948	2.31, 8	1.5	+	60	0.50	15	45	.90	3.28	5.24	3.76	3.12	3.18	5.16	3.16	2.74	2.82	4.68
4949	2.31, 8	1.5	-	60	0.50	45	15	.90	7.52	6.48	8.96	7.74	7.90	5.00	8.88	7.78	7.94	5.18
4950	2.31, 8	1.5	0	80	0	40	40	.90	4.66	4.60	6.66	5.76	5.90	5.30	5.18	4.76	4.78	4.30
4951	2.31, 8	1.5	+	80	0.16	34	46	.90	3.96	4.20	5.52	4.82	4.88	5.50	4.52	3.84	3.96	4.58
4952	2.31, 8	1.5	-	80	0.16	46	34	.90	5.88	5.02	7.66	7.00	7.10	5.50	6.20	5.46	5.62	4.40
4953	2.31, 8	1.5	+	80	0.33	27	53	.90	3.82	4.36	4.70	4.04	4.14	5.28	3.54	3.04	3.14	4.22
4954	2.31, 8	1.5	-	80	0.33	53	27	.90	6.68	5.48	9.12	8.18	8.28	5.38	6.84	6.14	6.32	4.24
4955	2.31, 8	1.5	+	80	0.50	20	60	.90	3.16	5.74	4.00	3.42	3.48	5.76	3.30	2.86	2.94	4.58
4956	2.31, 8	1.5	-	80	0.50	60	20	.90	7.40	6.42	10.34	9.00	9.16	5.76	8.76	7.92	8.10	4.98
4957	2.31, 8	1.5	0	100	0	50	50	.90	4.98	4.76	6.14	5.42	5.52	5.36	4.74	4.20	4.26	4.28
4958	2.31, 8	1.5	+	100	0.16	42	58	.90	4.30	4.66	5.30	4.48	4.62	5.32	4.48	3.90	3.90	4.52

	D	VR	P	N	Δn	n1	n2	ε	F g	B-F g	F t	F-GG t	F-HF t	B-F t	F g x t	F-GG g x t	F-HF g x t	B-F g x t
4959	2.31, 8	1.5	-	100	0.16	58	42	.90	5.74	5.00	6.84	5.92	5.96	5.08	6.24	5.62	5.72	4.70
4960	2.31, 8	1.5	+	100	0.33	33	67	.90	3.24	4.46	4.38	3.80	3.86	5.30	3.56	3.22	3.26	4.60
4961	2.31, 8	1.5	-	100	0.33	67	33	.90	7.20	5.80	7.90	6.80	6.86	5.32	7.72	6.86	6.94	4.98
4962	2.31, 8	1.5	+	100	0.50	25	75	.90	2.92	5.24	3.22	2.78	2.88	5.46	2.82	2.50	2.54	4.56
4963	2.31, 8	1.5	-	100	0.50	75	25	.90	8.02	6.72	8.84	7.82	7.94	4.94	9.34	8.36	8.44	4.88
4964	2.31, 8	1.5	0	150	0	75	75	.90	5.56	5.42	5.86	5.28	5.28	5.20	5.82	5.22	5.24	5.26
4965	2.31, 8	1.5	+	150	0.16	63	87	.90	4.86	5.52	4.94	4.30	4.30	5.26	4.80	4.30	4.34	5.34
4966	2.31, 8	1.5	-	150	0.16	87	63	.90	6.00	5.24	7.08	6.10	6.16	5.06	6.28	5.52	5.58	4.72
4967	2.31, 8	1.5	+	150	0.33	50	100	.90	3.42	4.98	4.02	3.62	3.62	5.20	3.48	3.06	3.08	5.06
4968	2.31, 8	1.5	-	150	0.33	100	50	.90	6.74	5.38	7.84	7.02	7.12	5.00	7.40	6.66	6.74	4.98
4969	2.31, 8	1.5	+	150	0.50	37	113	.90	2.80	5.56	3.32	2.96	2.96	5.62	2.98	2.66	2.66	5.04
4970	2.31, 8	1.5	-	150	0.50	113	37	.90	7.80	6.32	9.38	8.42	8.46	5.08	8.98	8.10	8.24	5.18
4971	2.31, 8	1.5	0	200	0	100	100	.90	5.04	5.14	5.88	5.02	5.08	5.16	5.72	5.02	5.08	5.20
4972	2.31, 8	1.5	+	200	0.16	84	116	.90	4.32	5.18	4.98	4.52	4.60	5.34	4.88	4.34	4.36	5.46
4973	2.31, 8	1.5	-	200	0.16	116	84	.90	5.62	5.06	6.54	5.86	5.88	5.14	6.40	5.56	5.64	5.08
4974	2.31, 8	1.5	+	200	0.33	67	133	.90	3.60	5.02	4.28	3.72	3.74	5.30	3.96	3.56	3.60	5.28
4975	2.31, 8	1.5	-	200	0.33	133	67	.90	6.48	5.46	8.02	7.04	7.14	5.04	8.16	7.38	7.42	5.52
4976	2.31, 8	1.5	+	200	0.50	50	150	.90	2.68	5.26	3.32	2.94	2.94	5.58	3.04	2.70	2.74	4.82
4977	2.31, 8	1.5	-	200	0.50	150	50	.90	7.66	5.98	9.10	8.14	8.18	5.06	9.00	8.16	8.30	5.60
4978	2.31, 8	2	0	20	0	10	10	.90	4.46	3.54	6.28	4.98	5.18	3.84	4.94	3.98	4.16	2.82
4979	2.31, 8	2	+	20	0.16	8	12	.90	3.76	3.10	4.56	3.36	3.56	3.94	3.72	2.86	3.06	2.64
4980	2.31, 8	2	-	20	0.16	12	8	.90	6.42	4.92	8.02	5.94	6.40	4.02	6.98	5.44	5.96	3.24
4981	2.31, 8	2	+	20	0.33	7	13	.90	3.62	3.86	3.80	2.88	3.12	3.84	3.06	2.46	2.60	2.68
4982	2.31, 8	2	-	20	0.33	13	7	.90	6.72	6.00	9.48	7.36	7.98	4.52	8.02	6.32	6.80	3.62
4983	2.31, 8	2	+	20	0.50	5	15	.90	2.78	4.34	2.86	2.32	2.50	4.40	2.34	1.70	1.90	3.46
4984	2.31, 8	2	-	20	0.50	15	5	.90	9.48	9.80	11.70	9.70	10.24	5.98	11.10	9.18	9.94	5.06
4985	2.31, 8	2	0	40	0	20	20	.90	5.06	4.14	5.68	4.52	4.68	3.82	5.22	4.44	4.72	3.74
4986	2.31, 8	2	+	40	0.16	17	23	.90	3.94	3.82	4.10	3.30	3.50	3.94	3.52	2.90	3.02	3.46
4987	2.31, 8	2	-	40	0.16	23	17	.90	6.18	5.04	7.12	6.06	6.30	4.18	6.70	5.64	5.88	4.12
4988	2.31, 8	2	+	40	0.33	13	27	.90	2.88	3.84	3.06	2.54	2.62	4.14	2.60	2.20	2.28	3.62
4989	2.31, 8	2	-	40	0.33	27	13	.90	7.50	5.96	10.10	8.84	9.10	4.46	8.80	7.36	7.70	4.16
4990	2.31, 8	2	+	40	0.50	10	30	.90	2.32	4.24	2.52	2.14	2.20	4.66	2.22	1.82	1.86	3.92

	D	VR	P	N	Δn	n1	n2	ε	F g	B-F g	F t	F-GG t	F-HF t	B-F t	F g x t	F-GG g x t	F-HF g x t	B-F g x t
4991	2.31, 8	2	-	40	0.50	30	10	.90	9.46	8.10	11.88	10.40	10.78	5.50	11.10	9.56	9.80	5.16
4992	2.31, 8	2	0	60	0	30	30	.90	4.70	4.32	5.68	5.02	5.06	4.66	5.34	4.48	4.60	4.50
4993	2.31, 8	2	+	60	0.16	25	35	.90	3.82	4.34	4.36	3.76	3.86	4.64	3.74	3.38	3.44	4.04
4994	2.31, 8	2	-	60	0.16	35	25	.90	5.98	4.98	7.84	6.72	6.84	4.92	7.20	6.32	6.40	4.80
4995	2.31, 8	2	+	60	0.33	20	40	.90	3.20	4.70	3.16	2.70	2.80	4.58	2.86	2.48	2.64	4.10
4996	2.31, 8	2	-	60	0.33	40	20	.90	7.60	5.60	9.82	8.80	8.96	4.78	9.20	8.24	8.38	4.64
4997	2.31, 8	2	+	60	0.50	15	45	.90	2.58	4.80	2.46	1.84	2.00	4.84	2.18	1.88	1.94	4.50
4998	2.31, 8	2	-	60	0.50	45	15	.90	9.76	7.04	11.82	10.50	10.60	5.16	11.74	10.44	10.72	5.32
4999	2.31, 8	2	0	80	0	40	40	.90	4.82	4.62	6.72	5.88	5.98	5.28	5.34	4.76	4.92	4.44
5000	2.31, 8	2	+	80	0.16	34	46	.90	3.70	4.16	5.08	4.44	4.48	5.52	4.10	3.52	3.58	4.54
5001	2.31, 8	2	-	80	0.16	46	34	.90	6.42	5.16	8.56	7.56	7.68	5.48	6.88	5.86	6.06	4.28
5002	2.31, 8	2	+	80	0.33	27	53	.90	3.06	4.30	3.70	3.24	3.28	5.40	2.74	2.30	2.40	4.04
5003	2.31, 8	2	-	80	0.33	53	27	.90	7.68	5.74	11.00	9.66	9.82	5.40	8.68	7.70	7.96	4.20
5004	2.31, 8	2	+	80	0.50	20	60	.90	2.48	5.40	2.52	2.02	2.04	5.66	2.30	1.84	1.88	4.54
5005	2.31, 8	2	-	80	0.50	60	20	.90	9.46	6.88	13.66	12.10	12.32	5.76	11.96	10.60	10.76	5.18
5006	2.31, 8	2	0	100	0	50	50	.90	5.20	5.02	6.08	5.22	5.28	5.30	4.70	4.22	4.28	4.34
5007	2.31, 8	2	+	100	0.16	42	58	.90	3.94	4.74	4.62	4.02	4.10	5.32	3.90	3.34	3.50	4.50
5008	2.31, 8	2	-	100	0.16	58	42	.90	6.38	5.14	7.68	6.66	6.74	5.04	6.92	6.22	6.36	4.68
5009	2.31, 8	2	+	100	0.33	33	67	.90	2.64	4.48	3.24	2.78	2.82	5.26	2.72	2.32	2.36	4.48
5010	2.31, 8	2	-	100	0.33	67	33	.90	8.78	5.64	9.84	8.58	8.66	5.06	9.48	8.62	8.72	4.46
5011	2.31, 8	2	+	100	0.50	25	75	.90	2.00	4.68	2.40	1.96	2.00	5.38	1.92	1.66	1.66	4.16
5012	2.31, 8	2	-	100	0.50	75	25	.90	10.36	7.08	12.08	10.66	10.80	4.96	12.00	10.88	11.08	4.88
5013	2.31, 8	2	0	150	0	75	75	.90	5.68	5.68	5.82	5.12	5.20	5.08	5.78	5.06	5.16	5.22
5014	2.31, 8	2	+	150	0.16	63	87	.90	4.52	5.56	4.68	4.04	4.06	5.28	4.22	3.70	3.76	5.08
5015	2.31, 8	2	-	150	0.16	87	63	.90	6.46	5.30	8.02	6.94	7.02	4.94	7.38	6.34	6.50	4.62
5016	2.31, 8	2	+	150	0.33	50	100	.90	3.02	4.76	3.14	2.76	2.80	5.10	2.64	2.32	2.38	4.94
5017	2.31, 8	2	-	150	0.33	100	50	.90	8.18	5.72	9.84	8.72	8.86	4.94	9.26	8.40	8.52	5.12
5018	2.31, 8	2	+	150	0.50	37	113	.90	2.18	5.22	2.30	1.86	1.88	5.64	2.08	1.80	1.80	4.88
5019	2.31, 8	2	-	150	0.50	113	37	.90	9.98	6.54	11.44	10.86	10.86	5.10	11.88	10.86	10.90	5.30
5020	2.31, 8	2	0	200	0	100	100	.90	5.14	5.20	5.90	5.06	5.16	5.26	5.66	5.04	5.06	5.08
5021	2.31, 8	2	+	200	0.16	84	116	.90	3.74	4.98	4.40	3.92	3.98	5.30	4.30	3.84	3.86	5.20
5022	2.31, 8	2	-	200	0.16	116	84	.90	6.30	5.06	7.02	6.30	6.36	4.74	6.94	6.34	6.38	4.84

	D	VR	P	N	Δn	n1	n2	ε	F g	B-F g	F t	F-GG t	F-HF t	B-F t	F g x t	F-GG g x t	F-HF g x t	B-F g x t
5023	2.31, 8	2	+	200	0.33	67	133	.90	2.92	5.00	3.38	2.92	2.92	5.06	3.04	2.60	2.64	5.04
5024	2.31, 8	2	-	200	0.33	133	67	.90	7.72	5.72	9.66	8.72	8.80	5.06	9.76	8.90	8.96	5.56
5025	2.31, 8	2	+	200	0.50	50	150	.90	2.04	5.14	2.18	1.90	1.90	5.52	1.98	1.80	1.80	4.68
5026	2.31, 8	2	-	200	0.50	150	50	.90	10.10	6.06	12.64	11.62	11.66	5.16	12.14	11.10	11.18	5.30
5027	2.31, 8	5	0	20	0	10	10	.90	6.98	6.06	6.74	5.18	5.54	4.14	5.86	4.66	4.98	3.48
5028	2.31, 8	5	+	20	0.16	8	12	.90	4.64	4.80	3.36	2.30	2.56	3.64	2.70	2.02	2.24	2.74
5029	2.31, 8	5	-	20	0.16	12	8	.90	11.20	7.92	11.26	8.76	9.42	4.40	10.60	8.14	8.88	3.80
5030	2.31, 8	5	+	20	0.33	7	13	.90	3.74	4.28	2.18	1.48	1.76	3.14	1.90	1.44	1.52	2.10
5031	2.31, 8	5	-	20	0.33	13	7	.90	13.18	8.92	14.48	11.90	12.54	4.24	13.54	11.30	11.86	4.04
5032	2.31, 8	5	+	20	0.50	5	15	.90	1.98	4.18	1.00	.66	.82	3.90	.74	.56	.60	3.14
5033	2.31, 8	5	-	20	0.50	15	5	.90	19.18	12.80	22.10	18.40	19.22	6.30	21.28	18.36	19.06	5.76
5034	2.31, 8	5	0	40	0	20	20	.90	6.00	5.46	5.84	4.48	4.74	3.86	5.54	4.70	4.86	3.96
5035	2.31, 8	5	+	40	0.16	17	23	.90	4.16	4.84	3.06	2.44	2.54	3.58	2.84	2.44	2.52	3.44
5036	2.31, 8	5	-	40	0.16	23	17	.90	8.86	6.54	9.36	7.96	8.28	3.98	9.42	7.86	8.20	4.44
5037	2.31, 8	5	+	40	0.33	13	27	.90	2.34	4.10	1.26	.98	1.04	3.92	1.30	1.02	1.08	3.68
5038	2.31, 8	5	-	40	0.33	27	13	.90	12.80	7.12	16.44	14.36	14.68	4.34	14.70	12.88	13.22	4.38
5039	2.31, 8	5	+	40	0.50	10	30	.90	1.42	3.98	.66	.44	.52	4.12	.72	.60	.64	3.86
5040	2.31, 8	5	-	40	0.50	30	10	.90	17.20	9.14	22.58	19.60	20.06	5.22	21.56	19.08	19.58	5.56
5041	2.31, 8	5	0	60	0	30	30	.90	5.52	4.78	5.94	4.86	5.04	4.36	5.62	4.78	4.84	4.04
5042	2.31, 8	5	+	60	0.16	25	35	.90	3.56	4.80	3.18	2.56	2.56	4.56	2.70	2.26	2.40	3.88
5043	2.31, 8	5	-	60	0.16	35	25	.90	8.56	5.42	10.48	8.90	9.06	4.34	10.04	8.56	8.82	4.36
5044	2.31, 8	5	+	60	0.33	20	40	.90	2.30	4.52	1.52	1.38	1.40	4.52	1.36	1.04	1.10	4.36
5045	2.31, 8	5	-	60	0.33	40	20	.90	12.14	6.12	15.76	14.08	14.30	4.52	15.58	13.70	14.00	4.60
5046	2.31, 8	5	+	60	0.50	15	45	.90	1.36	4.32	.46	.40	.42	4.58	.68	.56	.58	4.06
5047	2.31, 8	5	-	60	0.50	45	15	.90	18.58	7.82	22.92	20.56	20.74	4.70	22.40	20.18	20.52	5.12
5048	2.31, 8	5	0	80	0	40	40	.90	5.42	4.76	6.72	5.54	5.72	5.12	5.62	4.80	4.96	4.52
5049	2.31, 8	5	+	80	0.16	34	46	.90	3.50	4.40	3.94	3.18	3.26	5.40	3.30	2.86	2.92	4.46
5050	2.31, 8	5	-	80	0.16	46	34	.90	8.22	5.48	10.52	8.94	9.12	5.06	8.98	7.78	7.98	4.42
5051	2.31, 8	5	+	80	0.33	27	53	.90	2.34	4.50	1.82	1.54	1.54	5.40	1.42	1.16	1.18	4.12
5052	2.31, 8	5	-	80	0.33	53	27	.90	12.14	6.34	16.38	14.86	15.16	4.96	15.02	13.02	13.26	4.42
5053	2.31, 8	5	+	80	0.50	20	60	.90	1.36	4.68	.52	.34	.34	5.36	.46	.40	.42	4.26
5054	2.31, 8	5	-	80	0.50	60	20	.90	16.92	7.16	24.02	22.16	22.32	5.98	22.78	20.76	21.02	5.34

	D	VR	P	N	Δn	n1	n2	ε	F g	B-F g	F t	F-GG t	F-HF t	B-F t	F g x t	F-GG g x t	F-HF g x t	B-F g x t
5055	2.31, 8	5	0	100	0	50	50	.90	5.18	5.18	6.12	5.48	5.54	4.98	5.06	4.26	4.42	4.26
5056	2.31, 8	5	+	100	0.16	42	58	.90	3.24	4.66	3.46	2.90	3.00	4.80	2.78	2.30	2.32	4.26
5057	2.31, 8	5	-	100	0.16	58	42	.90	8.80	5.58	9.76	8.50	8.62	5.14	9.20	8.14	8.20	4.46
5058	2.31, 8	5	+	100	0.33	33	67	.90	1.78	4.16	1.50	1.18	1.20	5.22	1.08	.88	.88	4.36
5059	2.31, 8	5	-	100	0.33	67	33	.90	12.90	6.18	16.00	14.08	14.22	4.70	15.76	14.10	14.26	4.48
5060	2.31, 8	5	+	100	0.50	25	75	.90	.88	4.24	.50	.42	.42	5.32	.50	.38	.38	3.98
5061	2.31, 8	5	-	100	0.50	75	25	.90	17.84	7.18	22.18	20.50	20.74	4.62	22.70	20.74	20.98	4.96
5062	2.31, 8	5	0	150	0	75	75	.90	5.90	5.58	5.80	5.08	5.12	4.88	6.20	5.42	5.50	5.10
5063	2.31, 8	5	+	150	0.16	63	87	.90	3.86	5.52	3.16	2.76	2.78	5.24	3.32	2.74	2.78	5.34
5064	2.31, 8	5	-	150	0.16	87	63	.90	8.44	5.72	10.34	9.24	9.32	5.04	9.42	8.46	8.52	4.86
5065	2.31, 8	5	+	150	0.33	50	100	.90	2.00	4.70	1.50	1.32	1.34	5.18	1.32	1.12	1.14	4.84
5066	2.31, 8	5	-	150	0.33	100	50	.90	12.32	5.92	15.62	14.28	14.42	4.86	15.10	13.92	13.96	5.06
5067	2.31, 8	5	+	150	0.50	37	113	.90	.96	4.84	.56	.48	.50	5.10	.56	.50	.50	4.60
5068	2.31, 8	5	-	150	0.50	113	37	.90	17.60	6.74	22.38	20.60	20.78	5.08	22.24	20.72	20.90	5.24
5069	2.31, 8	5	0	200	0	100	100	.90	5.56	5.22	5.50	4.78	4.78	4.66	5.76	5.12	5.18	5.00
5070	2.31, 8	5	+	200	0.16	84	116	.90	3.22	4.90	3.16	2.74	2.74	4.86	3.04	2.64	2.66	5.14
5071	2.31, 8	5	-	200	0.16	116	84	.90	8.20	4.82	9.36	8.50	8.54	4.76	9.72	8.88	8.94	4.60
5072	2.31, 8	5	+	200	0.33	67	133	.90	1.86	4.96	1.38	1.12	1.14	4.96	1.32	1.12	1.12	5.18
5073	2.31, 8	5	-	200	0.33	133	67	.90	12.20	6.00	15.66	14.28	14.34	5.10	15.42	14.18	14.26	5.38
5074	2.31, 8	5	+	200	0.50	50	150	.90	.72	4.62	.42	.34	.34	5.28	.48	.30	.30	4.82
5075	2.31, 8	5	-	200	0.50	150	50	.90	17.26	6.38	23.24	21.66	21.76	5.10	22.46	20.66	20.80	5.28
5076	2.31, 8	1	0	20	0	10	10	1	3.58	2.72	4.28	3.20	3.36	2.54	4.00	3.36	3.68	2.40
5077	2.31, 8	1	0	20	0.16	8	12	1	4.24	3.34	4.44	3.30	3.66	2.62	4.28	3.56	3.84	2.76
5078	2.31, 8	1	0	20	0.33	7	13	1	4.64	4.20	4.52	3.68	4.06	2.96	4.44	3.46	3.78	3.02
5079	2.31, 8	1	0	20	0.50	5	15	1	4.54	6.10	5.44	4.38	4.72	3.48	5.12	4.00	4.50	3.58
5080	2.31, 8	1	0	40	0	20	20	1	5.04	4.06	4.80	4.14	4.34	3.58	4.22	3.68	3.94	3.28
5081	2.31, 8	1	0	40	0.16	17	23	1	4.46	4.08	4.80	4.16	4.32	3.98	4.52	4.08	4.24	3.68
5082	2.31, 8	1	0	40	0.33	13	27	1	4.50	4.46	5.08	4.54	4.68	3.84	4.52	4.06	4.28	3.98
5083	2.31, 8	1	0	40	0.50	10	30	1	4.14	5.76	5.30	4.54	4.64	4.36	5.08	4.56	4.72	4.42
5084	2.31, 8	1	0	60	0	30	30	1	4.66	4.20	4.92	4.42	4.58	4.36	5.04	4.68	4.80	4.46
5085	2.31, 8	1	0	60	0.16	25	35	1	4.66	4.36	5.20	4.66	4.78	4.60	4.52	4.14	4.34	3.82
5086	2.31, 8	1	0	60	0.33	20	40	1	4.94	5.32	5.42	5.08	5.22	4.52	4.88	4.54	4.60	3.76

	D	VR	P	N	Δn	n1	n2	ε	F g	B-F g	F t	F-GG t	F-HF t	B-F t	F g x t	F-GG g x t	F-HF g x t	B-F g x t
5087	2.31, 8	1	0	60	0.50	15	45	1	5.06	6.10	5.40	5.06	5.14	4.42	4.74	4.48	4.58	4.40
5088	2.31, 8	1	0	80	0	40	40	1	4.68	4.52	5.58	5.18	5.22	5.00	4.84	4.58	4.68	4.30
5089	2.31, 8	1	0	80	0.16	34	46	1	4.62	4.56	5.20	4.84	4.98	5.04	5.12	4.94	5.00	4.94
5090	2.31, 8	1	0	80	0.33	27	53	1	4.78	4.66	5.30	4.80	4.90	4.54	4.90	4.58	4.66	4.46
5091	2.31, 8	1	0	80	0.50	20	60	1	4.78	6.40	5.54	5.38	5.42	4.94	5.00	4.80	4.90	4.28
5092	2.31, 8	1	0	100	0	50	50	1	5.04	4.90	5.10	4.68	4.76	5.02	4.70	4.48	4.54	4.56
5093	2.31, 8	1	0	100	0.16	42	58	1	4.88	4.74	5.42	5.02	5.16	5.02	4.84	4.58	4.64	4.74
5094	2.31, 8	1	0	100	0.33	33	67	1	4.74	5.10	5.22	4.90	4.98	4.88	5.32	5.06	5.12	4.72
5095	2.31, 8	1	0	100	0.50	25	75	1	4.36	5.84	5.20	4.86	4.96	4.70	4.92	4.68	4.72	4.40
5096	2.31, 8	1	0	150	0	75	75	1	5.46	5.38	5.04	4.74	4.84	5.00	5.42	5.26	5.34	5.56
5097	2.31, 8	1	0	150	0.16	63	87	1	5.36	5.40	4.94	4.74	4.76	4.78	5.10	4.90	4.94	5.00
5098	2.31, 8	1	0	150	0.33	50	100	1	4.96	5.14	5.24	5.04	5.06	5.02	4.92	4.70	4.80	4.96
5099	2.31, 8	1	0	150	0.50	37	113	1	4.68	5.94	5.38	5.08	5.14	5.04	5.04	4.82	4.96	5.02
5100	2.31, 8	1	0	200	0	100	100	1	5.18	5.18	4.98	4.72	4.78	4.64	5.12	5.04	5.12	5.36
5101	2.31, 8	1	0	200	0.16	84	116	1	5.04	5.18	5.12	4.98	5.02	5.02	5.34	5.26	5.30	5.22
5102	2.31, 8	1	0	200	0.33	67	133	1	4.92	5.08	5.62	5.38	5.42	5.64	5.32	5.26	5.26	5.10
5103	2.31, 8	1	0	200	0.50	50	150	1	4.82	5.42	5.32	5.18	5.22	5.26	5.14	4.96	4.98	4.88
5104	2.31, 8	1.5	0	20	0	10	10	1	3.94	2.98	4.30	3.28	3.52	2.64	4.10	3.40	3.68	2.44
5105	2.31, 8	1.5	+	20	0.16	8	12	1	4.00	3.10	3.64	2.86	3.02	2.44	3.38	2.72	2.92	2.52
5106	2.31, 8	1.5	-	20	0.16	12	8	1	5.36	4.18	5.28	4.00	4.32	2.30	5.24	4.34	4.66	2.66
5107	2.31, 8	1.5	+	20	0.33	7	13	1	3.82	3.84	3.34	2.62	2.82	2.76	3.00	2.34	2.58	2.84
5108	2.31, 8	1.5	-	20	0.33	13	7	1	5.40	4.82	6.36	4.48	5.18	2.42	6.22	5.06	5.42	2.66
5109	2.31, 8	1.5	+	20	0.50	5	15	1	3.46	4.74	3.26	2.64	2.90	3.38	2.98	2.46	2.62	3.48
5110	2.31, 8	1.5	-	20	0.50	15	5	1	6.86	8.52	8.24	6.50	7.04	3.88	8.44	7.22	7.78	3.72
5111	2.31, 8	1.5	0	40	0	20	20	1	5.00	4.32	4.68	3.94	4.22	3.54	4.24	3.84	3.96	3.32
5112	2.31, 8	1.5	+	40	0.16	17	23	1	3.88	4.08	4.18	3.54	3.72	3.90	3.78	3.34	3.44	3.84
5113	2.31, 8	1.5	-	40	0.16	23	17	1	5.40	4.72	5.54	5.00	5.14	3.98	5.28	4.72	4.94	3.62
5114	2.31, 8	1.5	+	40	0.33	13	27	1	3.44	4.14	3.52	2.86	3.06	4.02	2.84	2.62	2.66	3.98
5115	2.31, 8	1.5	-	40	0.33	27	13	1	6.32	5.58	7.10	6.38	6.54	4.10	6.26	5.50	5.88	3.66
5116	2.31, 8	1.5	+	40	0.50	10	30	1	2.84	5.22	2.82	2.56	2.62	4.52	3.04	2.70	2.88	4.24
5117	2.31, 8	1.5	-	40	0.50	30	10	1	7.00	6.94	8.46	7.58	7.86	4.06	7.78	6.84	7.20	3.98
5118	2.31, 8	1.5	0	60	0	30	30	1	4.60	4.20	4.88	4.26	4.48	4.14	5.06	4.72	4.86	4.42

	D	VR	P	N	Δn	n1	n2	ε	F g	B-F g	F t	F-GG t	F-HF t	B-F t	F g x t	F-GG g x t	F-HF g x t	B-F g x t
5119	2.31, 8	1.5	+	60	0.16	25	35	1	4.12	4.18	4.18	3.80	3.96	4.46	3.80	3.42	3.50	3.80
5120	2.31, 8	1.5	-	60	0.16	35	25	1	5.64	4.64	6.26	5.60	5.70	4.28	6.34	5.84	6.02	4.46
5121	2.31, 8	1.5	+	60	0.33	20	40	1	3.68	4.84	3.66	3.14	3.32	4.56	3.32	3.10	3.12	4.08
5122	2.31, 8	1.5	-	60	0.33	40	20	1	6.28	5.32	7.26	6.72	6.84	4.24	6.86	6.34	6.46	4.26
5123	2.31, 8	1.5	+	60	0.50	15	45	1	3.38	5.42	3.04	2.76	2.84	4.44	2.62	2.36	2.50	4.46
5124	2.31, 8	1.5	-	60	0.50	45	15	1	7.14	6.58	8.32	7.74	7.94	4.22	8.54	8.14	8.26	4.80
5125	2.31, 8	1.5	0	80	0	40	40	1	4.50	4.36	5.64	5.12	5.20	4.96	4.80	4.58	4.72	4.54
5126	2.31, 8	1.5	+	80	0.16	34	46	1	3.70	4.36	4.54	4.08	4.16	5.14	4.44	4.16	4.26	5.14
5127	2.31, 8	1.5	-	80	0.16	46	34	1	5.38	4.90	6.42	5.82	5.90	4.78	5.56	5.16	5.30	4.40
5128	2.31, 8	1.5	+	80	0.33	27	53	1	3.74	4.52	3.56	3.40	3.40	4.82	3.06	2.92	2.92	4.52
5129	2.31, 8	1.5	-	80	0.33	53	27	1	6.32	5.42	7.94	7.36	7.50	4.68	6.86	6.58	6.64	4.46
5130	2.31, 8	1.5	+	80	0.50	20	60	1	3.10	5.92	3.14	2.86	2.92	5.44	2.74	2.48	2.56	4.40
5131	2.31, 8	1.5	-	80	0.50	60	20	1	7.16	6.36	9.12	8.72	8.80	4.72	8.56	8.10	8.28	4.52
5132	2.31, 8	1.5	0	100	0	50	50	1	5.10	5.00	5.14	4.78	4.88	4.96	4.86	4.62	4.70	4.66
5133	2.31, 8	1.5	+	100	0.16	42	58	1	4.32	4.72	4.40	4.18	4.26	5.08	3.78	3.66	3.70	4.66
5134	2.31, 8	1.5	-	100	0.16	58	42	1	5.82	5.16	6.34	6.04	6.16	5.02	5.88	5.60	5.68	4.88
5135	2.31, 8	1.5	+	100	0.33	33	67	1	3.48	4.72	3.62	3.42	3.46	5.06	3.52	3.30	3.38	4.70
5136	2.31, 8	1.5	-	100	0.33	67	33	1	7.12	5.74	7.18	6.76	6.82	4.30	7.26	7.02	7.10	4.46
5137	2.31, 8	1.5	+	100	0.50	25	75	1	2.82	5.18	2.90	2.60	2.68	4.96	2.52	2.38	2.40	4.20
5138	2.31, 8	1.5	-	100	0.50	75	25	1	7.98	6.76	8.52	8.04	8.16	4.16	8.70	8.28	8.38	4.26
5139	2.31, 8	1.5	0	150	0	75	75	1	5.28	5.40	5.20	4.84	4.92	4.82	5.42	5.14	5.20	5.26
5140	2.31, 8	1.5	+	150	0.16	63	87	1	4.80	5.38	4.12	3.88	3.94	4.96	4.08	3.78	3.86	5.02
5141	2.31, 8	1.5	-	150	0.16	87	63	1	5.98	5.34	6.08	5.86	5.88	4.80	6.16	5.96	6.00	5.04
5142	2.31, 8	1.5	+	150	0.33	50	100	1	3.44	4.88	3.44	3.26	3.26	4.96	3.54	3.32	3.36	4.84
5143	2.31, 8	1.5	-	150	0.33	100	50	1	6.32	5.44	7.20	6.80	6.94	4.88	7.18	6.92	7.00	4.90
5144	2.31, 8	1.5	+	150	0.50	37	113	1	3.00	5.38	2.82	2.68	2.70	4.98	2.70	2.52	2.56	4.98
5145	2.31, 8	1.5	-	150	0.50	113	37	1	7.88	6.28	9.30	8.92	9.02	4.84	8.94	8.64	8.72	5.06
5146	2.31, 8	1.5	0	200	0	100	100	1	5.24	5.26	5.10	4.86	4.92	4.72	5.30	5.14	5.20	5.10
5147	2.31, 8	1.5	+	200	0.16	84	116	1	4.38	5.24	4.18	4.10	4.10	5.00	4.52	4.38	4.42	5.10
5148	2.31, 8	1.5	-	200	0.16	116	84	1	5.82	5.00	5.98	5.78	5.82	4.62	6.40	6.24	6.34	5.02
5149	2.31, 8	1.5	+	200	0.33	67	133	1	3.64	5.14	3.78	3.52	3.58	5.12	3.44	3.40	3.42	4.94
5150	2.31, 8	1.5	-	200	0.33	133	67	1	6.54	5.74	6.82	6.70	6.72	4.78	7.50	7.36	7.40	5.30

	D	VR	P	N	Δn	n1	n2	ε	F g	B-F g	F t	F-GG t	F-HF t	B-F t	F g x t	F-GG g x t	F-HF g x t	B-F g x t
5151	2.31, 8	1.5	+	200	0.50	50	150	1	2.76	5.04	2.96	2.84	2.86	5.20	2.50	2.42	2.42	5.22
5152	2.31, 8	1.5	-	200	0.50	150	50	1	7.66	5.66	8.60	8.34	8.36	4.80	9.02	8.82	8.90	4.88
5153	2.31, 8	2	0	20	0	10	10	1	4.78	3.60	4.44	3.38	3.76	2.56	4.14	3.32	3.70	2.42
5154	2.31, 8	2	+	20	0.16	8	12	1	4.06	3.18	3.12	2.38	2.48	2.58	2.88	2.36	2.56	2.62
5155	2.31, 8	2	-	20	0.16	12	8	1	6.40	4.80	6.22	4.54	5.06	2.30	6.12	4.94	5.46	2.56
5156	2.31, 8	2	+	20	0.33	7	13	1	3.60	3.80	2.54	2.10	2.34	2.70	2.30	1.74	1.94	2.66
5157	2.31, 8	2	-	20	0.33	13	7	1	7.04	6.08	7.90	5.90	6.36	2.66	7.78	6.16	6.70	2.80
5158	2.31, 8	2	+	20	0.50	5	15	1	3.02	4.30	2.26	1.86	2.00	3.14	1.96	1.44	1.64	3.42
5159	2.31, 8	2	-	20	0.50	15	5	1	9.24	9.70	10.92	9.00	9.48	3.74	10.80	9.30	9.88	3.64
5160	2.31, 8	2	0	40	0	20	20	1	5.02	4.50	4.70	3.92	4.16	3.62	4.12	3.64	3.92	3.36
5161	2.31, 8	2	+	40	0.16	17	23	1	3.94	4.36	3.58	2.88	3.12	3.94	3.28	2.88	3.08	3.88
5162	2.31, 8	2	-	40	0.16	23	17	1	6.32	5.30	6.28	5.40	5.66	3.90	6.06	5.28	5.66	3.62
5163	2.31, 8	2	+	40	0.33	13	27	1	3.00	3.82	2.58	2.16	2.24	4.04	2.16	1.88	1.96	3.84
5164	2.31, 8	2	-	40	0.33	27	13	1	7.66	6.20	9.22	8.14	8.40	3.86	8.00	7.20	7.34	3.62
5165	2.31, 8	2	+	40	0.50	10	30	1	2.40	4.50	1.98	1.78	1.88	4.46	1.98	1.68	1.78	4.26
5166	2.31, 8	2	-	40	0.50	30	10	1	9.26	7.92	11.20	9.92	10.36	4.26	10.56	9.68	10.02	4.10
5167	2.31, 8	2	0	60	0	30	30	1	4.78	4.18	4.90	4.32	4.48	4.12	4.96	4.66	4.78	4.26
5168	2.31, 8	2	+	60	0.16	25	35	1	4.00	4.14	3.70	3.20	3.30	4.26	3.18	3.06	3.12	3.92
5169	2.31, 8	2	-	60	0.16	35	25	1	6.32	4.60	6.98	6.30	6.38	4.40	6.98	6.50	6.58	4.28
5170	2.31, 8	2	+	60	0.33	20	40	1	3.02	4.62	2.56	2.24	2.28	4.44	2.52	2.32	2.32	3.96
5171	2.31, 8	2	-	60	0.33	40	20	1	7.42	5.48	8.84	8.18	8.32	4.18	8.20	7.74	7.90	4.10
5172	2.31, 8	2	+	60	0.50	15	45	1	2.68	5.02	1.80	1.68	1.72	4.48	1.72	1.60	1.68	4.40
5173	2.31, 8	2	-	60	0.50	45	15	1	9.58	7.02	11.20	10.48	10.62	4.02	11.24	10.56	10.68	4.66
5174	2.31, 8	2	0	80	0	40	40	1	4.70	4.56	5.44	4.90	5.10	4.56	4.92	4.64	4.76	4.38
5175	2.31, 8	2	+	80	0.16	34	46	1	3.58	4.18	3.86	3.60	3.66	5.24	3.84	3.58	3.66	5.00
5176	2.31, 8	2	-	80	0.16	46	34	1	6.22	5.04	7.22	6.50	6.64	4.66	6.16	5.84	5.90	4.36
5177	2.31, 8	2	+	80	0.33	27	53	1	3.30	4.56	2.70	2.44	2.56	5.00	2.38	2.24	2.26	4.54
5178	2.31, 8	2	-	80	0.33	53	27	1	7.76	5.64	9.82	9.24	9.34	4.60	8.98	8.46	8.68	4.54
5179	2.31, 8	2	+	80	0.50	20	60	1	2.40	5.74	1.92	1.74	1.80	5.36	1.40	1.34	1.38	4.36
5180	2.31, 8	2	-	80	0.50	60	20	1	9.58	6.74	12.44	11.80	11.96	4.80	11.96	11.22	11.54	4.48
5181	2.31, 8	2	0	100	0	50	50	1	5.24	5.02	4.96	4.52	4.60	4.66	4.98	4.70	4.84	4.62
5182	2.31, 8	2	+	100	0.16	42	58	1	4.12	4.88	3.78	3.46	3.52	4.72	3.12	2.90	2.98	4.48

	D	VR	P	N	Δn	n1	n2	ε	F g	B-F g	F t	F-GG t	F-HF t	B-F t	F g x t	F-GG g x t	F-HF g x t	B-F g x t
5183	2.31, 8	2	-	100	0.16	58	42	1	6.42	5.30	6.90	6.50	6.60	4.74	6.66	6.32	6.42	4.88
5184	2.31, 8	2	+	100	0.33	33	67	1	2.72	4.56	2.64	2.40	2.48	4.84	2.48	2.32	2.38	4.56
5185	2.31, 8	2	-	100	0.33	67	33	1	8.60	6.42	9.38	8.80	8.86	4.60	9.26	8.88	9.04	4.96
5186	2.31, 8	2	+	100	0.50	25	75	1	1.94	4.76	1.74	1.58	1.60	4.84	1.56	1.44	1.50	4.20
5187	2.31, 8	2	-	100	0.50	75	25	1	10.24	7.02	11.42	10.72	10.84	4.18	11.62	11.30	11.46	4.22
5188	2.31, 8	2	0	150	0	75	75	1	5.60	5.42	5.02	4.86	4.90	4.84	5.40	5.14	5.22	5.20
5189	2.31, 8	2	+	150	0.16	63	87	1	4.46	5.46	3.72	3.44	3.56	5.02	3.66	3.56	3.56	5.10
5190	2.31, 8	2	-	150	0.16	87	63	1	6.38	5.26	6.90	6.68	6.74	4.82	6.64	6.44	6.48	4.90
5191	2.31, 8	2	+	150	0.33	50	100	1	2.94	4.74	2.64	2.46	2.52	4.86	2.44	2.26	2.28	4.72
5192	2.31, 8	2	-	150	0.33	100	50	1	8.04	5.66	8.88	8.66	8.74	4.88	8.88	8.66	8.76	5.04
5193	2.31, 8	2	+	150	0.50	37	113	1	2.16	5.06	1.62	1.54	1.56	5.30	1.62	1.60	1.60	4.92
5194	2.31, 8	2	-	150	0.50	113	37	1	10.30	6.44	12.28	11.82	11.92	4.86	12.34	12.04	12.10	5.10
5195	2.31, 8	2	0	200	0	100	100	1	5.28	5.16	5.16	4.90	5.02	4.94	5.26	5.10	5.18	5.20
5196	2.31, 8	2	+	200	0.16	84	116	1	3.90	5.20	3.74	3.56	3.60	4.66	3.92	3.82	3.86	5.24
5197	2.31, 8	2	-	200	0.16	116	84	1	6.44	5.40	6.46	6.26	6.28	4.70	6.98	6.84	6.90	5.32
5198	2.31, 8	2	+	200	0.33	67	133	1	2.98	5.00	2.68	2.56	2.58	5.12	2.56	2.56	2.56	5.18
5199	2.31, 8	2	-	200	0.33	133	67	1	8.00	5.48	8.80	8.56	8.60	4.56	9.08	8.88	8.98	5.04
5200	2.31, 8	2	+	200	0.50	50	150	1	1.92	4.86	1.76	1.68	1.68	5.20	1.40	1.34	1.36	5.18
5201	2.31, 8	2	-	200	0.50	150	50	1	9.82	6.04	11.68	11.28	11.46	4.58	11.78	11.58	11.62	4.96
5202	2.31, 8	5	0	20	0	10	10	1	7.00	5.96	4.56	3.24	3.56	2.38	4.32	3.24	3.52	2.10
5203	2.31, 8	5	+	20	0.16	8	12	1	4.82	5.00	1.96	1.46	1.56	2.42	1.72	1.20	1.36	2.12
5204	2.31, 8	5	-	20	0.16	12	8	1	11.36	7.88	9.34	7.18	7.80	2.32	8.72	6.98	7.34	2.34
5205	2.31, 8	5	+	20	0.33	7	13	1	3.60	4.66	1.30	.88	.98	2.42	.92	.70	.80	2.32
5206	2.31, 8	5	-	20	0.33	13	7	1	13.22	9.22	13.14	10.30	11.10	2.56	12.64	10.10	10.96	2.64
5207	2.31, 8	5	+	20	0.50	5	15	1	2.24	4.18	.50	.36	.42	2.54	.36	.24	.30	2.64
5208	2.31, 8	5	-	20	0.50	15	5	1	19.30	13.20	20.58	17.24	18.16	3.62	20.42	17.64	18.52	3.76
5209	2.31, 8	5	0	40	0	20	20	1	6.16	5.20	4.54	3.64	3.78	3.12	4.24	3.60	3.74	3.18
5210	2.31, 8	5	+	40	0.16	17	23	1	4.10	4.82	2.56	2.16	2.34	3.44	2.60	2.14	2.32	3.78
5211	2.31, 8	5	-	40	0.16	23	17	1	9.14	6.60	8.18	7.08	7.46	3.36	8.10	7.28	7.48	3.40
5212	2.31, 8	5	+	40	0.33	13	27	1	2.44	4.28	.86	.70	.74	3.64	.90	.80	.80	3.58
5213	2.31, 8	5	-	40	0.33	27	13	1	13.10	7.60	15.50	13.88	14.24	3.74	14.04	12.52	12.92	3.48
5214	2.31, 8	5	+	40	0.50	10	30	1	1.60	4.06	.38	.24	.28	4.16	.30	.28	.28	4.06

	D	VR	P	N	Δn	n1	n2	ε	F g	B-F g	F t	F-GG t	F-HF t	B-F t	F g x t	F-GG g x t	F-HF g x t	B-F g x t
5215	2.31, 8	5	-	40	0.50	30	10	1	17.62	9.54	22.02	19.82	20.20	3.74	20.92	19.12	19.42	3.72
5216	2.31, 8	5	0	60	0	30	30	1	5.56	5.12	5.20	4.40	4.56	4.36	4.92	4.60	4.66	4.14
5217	2.31, 8	5	+	60	0.16	25	35	1	3.62	4.90	2.40	2.14	2.16	4.02	2.08	1.84	1.88	4.18
5218	2.31, 8	5	-	60	0.16	35	25	1	8.60	5.46	8.96	8.08	8.30	4.34	8.96	8.26	8.42	4.34
5219	2.31, 8	5	+	60	0.33	20	40	1	2.26	4.46	1.12	.94	.98	3.96	.92	.72	.76	4.00
5220	2.31, 8	5	-	60	0.33	40	20	1	12.40	6.32	14.46	13.06	13.44	3.46	14.24	13.00	13.30	3.44
5221	2.31, 8	5	+	60	0.50	15	45	1	1.46	4.46	.36	.32	.36	4.44	.36	.30	.32	4.26
5222	2.31, 8	5	-	60	0.50	45	15	1	17.64	8.00	21.42	20.08	20.40	3.80	22.02	21.04	21.30	4.26
5223	2.31, 8	5	0	80	0	40	40	1	5.42	4.92	5.38	4.94	5.06	4.44	4.74	4.32	4.36	4.12
5224	2.31, 8	5	+	80	0.16	34	46	1	3.44	4.46	2.68	2.36	2.42	4.96	2.78	2.56	2.58	4.74
5225	2.31, 8	5	-	80	0.16	46	34	1	8.48	5.50	9.14	8.44	8.50	4.48	8.36	7.62	7.76	4.08
5226	2.31, 8	5	+	80	0.33	27	53	1	2.36	4.58	1.14	.92	.94	4.94	.90	.82	.84	4.34
5227	2.31, 8	5	-	80	0.33	53	27	1	12.00	5.96	15.40	14.38	14.56	4.02	14.94	14.16	14.32	4.14
5228	2.31, 8	5	+	80	0.50	20	60	1	1.20	4.62	.30	.28	.28	5.32	.18	.18	.18	4.48
5229	2.31, 8	5	-	80	0.50	60	20	1	16.64	7.20	23.66	22.60	22.86	4.20	23.02	21.80	22.02	4.20
5230	2.31, 8	5	0	100	0	50	50	1	5.60	5.14	4.92	4.52	4.58	4.10	5.00	4.56	4.68	4.34
5231	2.31, 8	5	+	100	0.16	42	58	1	3.36	4.66	2.44	2.16	2.16	4.50	2.14	1.90	1.94	4.22
5232	2.31, 8	5	-	100	0.16	58	42	1	8.74	5.76	9.18	8.68	8.78	4.56	8.92	8.30	8.40	4.62
5233	2.31, 8	5	+	100	0.33	33	67	1	1.66	4.34	.98	.82	.82	4.36	.94	.90	.92	4.24
5234	2.31, 8	5	-	100	0.33	67	33	1	13.16	6.72	15.62	14.92	15.04	4.38	14.84	14.04	14.20	4.54
5235	2.31, 8	5	+	100	0.50	25	75	1	.86	3.86	.40	.38	.38	4.86	.22	.22	.22	4.00
5236	2.31, 8	5	-	100	0.50	75	25	1	17.70	7.32	22.28	21.36	21.58	3.80	21.62	20.56	20.80	4.06
5237	2.31, 8	5	0	150	0	75	75	1	5.62	5.38	4.92	4.54	4.56	4.84	5.12	4.88	4.90	4.98
5238	2.31, 8	5	+	150	0.16	63	87	1	3.74	5.22	2.48	2.34	2.42	4.84	2.50	2.36	2.40	4.74
5239	2.31, 8	5	-	150	0.16	87	63	1	8.50	5.34	8.92	8.48	8.56	4.64	8.32	7.98	8.18	4.52
5240	2.31, 8	5	+	150	0.33	50	100	1	1.96	4.74	1.02	.98	1.00	4.76	1.10	1.00	1.04	4.80
5241	2.31, 8	5	-	150	0.33	100	50	1	12.10	5.94	15.26	14.72	14.80	4.60	15.28	14.54	14.62	4.58
5242	2.31, 8	5	+	150	0.50	37	113	1	.88	4.52	.36	.28	.30	4.72	.28	.26	.26	4.82
5243	2.31, 8	5	-	150	0.50	113	37	1	18.12	6.60	22.94	22.22	22.36	4.60	22.96	22.46	22.58	4.66
5244	2.31, 8	5	0	200	0	100	100	1	5.44	5.18	4.82	4.70	4.74	4.70	5.34	5.18	5.20	5.18
5245	2.31, 8	5	+	200	0.16	84	116	1	2.96	4.90	2.40	2.26	2.26	4.72	2.66	2.40	2.48	5.02
5246	2.31, 8	5	-	200	0.16	116	84	1	8.38	5.24	8.98	8.50	8.56	4.58	9.30	8.94	9.04	5.12

	D	VR	P	N	Δn	n1	n2	ε	F g	B-F g	F t	F-GG t	F-HF t	B-F t	F g x t	F-GG g x t	F-HF g x t	B-F g x t
5247	2.31, 8	5	+	200	0.33	67	133	1	1.62	5.02	.92	.82	.84	4.78	.96	.94	.96	5.32
5248	2.31, 8	5	-	200	0.33	133	67	1	12.40	5.82	14.60	14.14	14.24	4.58	15.06	14.56	14.64	5.02
5249	2.31, 8	5	+	200	0.50	50	150	1	.70	4.56	.30	.30	.30	4.80	.26	.26	.26	4.86
5250	2.31, 8	5	-	200	0.50	150	50	1	17.54	6.32	23.16	22.62	22.70	4.66	23.00	22.42	22.54	4.82
